# Insight into the phylogeny and metabolic divergence of *Monascus* species (*M. pilosus*, *M. ruber*, and *M. purpureus*) at the genome level

**DOI:** 10.3389/fmicb.2023.1199144

**Published:** 2023-05-25

**Authors:** Zhiyu Zhang, Mengfei Cui, Panting Chen, Juxing Li, Zhitao Mao, Yufeng Mao, Zhenjing Li, Qingbin Guo, Changlu Wang, Xiaoping Liao, Huanhuan Liu

**Affiliations:** ^1^State Key Laboratory of Food Nutrition and Safety, Tianjin University of Science and Technology, Tianjin, China; ^2^State Key Laboratory of Food Nutrition and Safety, Tianjin University of Science and Technology, Ministry of Education, Tianjin, China; ^3^Biodesign Center, Key Laboratory of Engineering Biology for Low-Carbon Manufacturing, Tianjin Institute of Industrial Biotechnology, Chinese Academy of Sciences, Tianjin, China; ^4^Haihe Laboratory of Synthetic Biology, Tianjin, China

**Keywords:** *Monascus*, phylogenetic analysis, taxonomic divergence, secondary metabolite gene clusters, pan-genome

## Abstract

**Background:**

Species of the genus *Monascus* are economically important and widely used in the production of food colorants and monacolin K. However, they have also been known to produce the mycotoxin citrinin. Currently, taxonomic knowledge of this species at the genome level is insufficient.

**Methods:**

This study presents genomic similarity analyses through the analysis of the average nucleic acid identity of the genomic sequence and the whole genome alignment. Subsequently, the study constructed a pangenome of *Monascus* by reannotating all the genomes and identifying a total of 9,539 orthologous gene families. Two phylogenetic trees were constructed based on 4,589 single copy orthologous protein sequences and all the 5,565 orthologous proteins, respectively. In addition, carbohydrate active enzymes, secretome, allergic proteins, as well as secondary metabolite gene clusters were compared among the included 15 *Monascus* strains.

**Results:**

The results clearly revealed a high homology between *M. pilosus* and *M. ruber*, and their distant relationship with *M. purpureus*. Accordingly, all the included 15 *Monascus* strains should be classified into two distinctly evolutionary clades, namely the *M. purpureus* clade and the *M. pilosus*-*M. ruber* clade. Moreover, gene ontology enrichment showed that the *M. pilosus*-*M. ruber* clade had more orthologous genes involved with environmental adaptation than the *M. purpureus* clade. Compared to *Aspergillus oryzae*, all the *Monascus* species had a substantial gene loss of carbohydrate active enzymes. Potential allergenic and fungal virulence factor proteins were also found in the secretome of *Monascus*. Furthermore, this study identified the pigment synthesis gene clusters present in all included genomes, but with multiple nonessential genes inserted in the gene cluster of *M. pilosus* and *M. ruber* compared to *M. purpureus*. The citrinin gene cluster was found to be intact and highly conserved only among *M. purpureus* genomes. The monacolin K gene cluster was found only in the genomes of *M. pilosus* and *M. ruber*, but the sequence was more conserved in *M. ruber.*

**Conclusion:**

This study provides a paradigm for phylogenetic analysis of the genus *Monascus*, and it is believed that this report will lead to a better understanding of these food microorganisms in terms of classification, metabolic differentiation, and safety.

## 1. Introduction

*Monascus* spp. is a highly valuable edible fungus that has been traditionally consumed in China and other Asian countries such as Japan, Republic of Korea, and the Philippines. It has high nutritional value due to the synthesis of a variety of beneficial secondary metabolites, including *Monascus* azaphilone pigments (MonAzPs), monacolin K (MK), aminobutyric acid, ergosterol, and Hong Qu polysaccharide (Wang et al., 2021), with MonAzPs and MK being of special concern. MonAzPs are a type of chromogenic chemical consisting of a chromophore with a polyketide structure and medium- or long-chain fatty acids. They are highly promising in the food, healthcare, and cosmetic industries due to their excellent coloring properties, good biological efficacy (antioxidant, anti-inflammatory, hypolipidemic, and anti-tumor properties), and non-toxic side effects as natural food additives (Chen et al., 2019). Another important metabolite, MK, is an inhibitor of 3-hydroxy-3-methylglutaryl-coenzyme A (HMG-CoA) reductase, a critical enzyme in endogenous cholesterol production (Zhang et al., 2020). It is a prescription drug with brand names Mevinoline, Lovastatin, or Mevalonate used to treat excessive cholesterol, coronary heart disease, and other disorders (Karthikeyan and Dharumadurai, 2023). However, concerns were raised regarding the safety of *Monascus* products when citrinin, a mycotoxin hazardous to both humans and animals, was detected in *Monascus* products as early as 1995 (Shao et al., 2014). Citrinin is nephrotoxic and can cause kidney enlargement, renal tubule expansion, and renal epithelial cell degeneration and necrosis. Fortunately, some citrinin-free *Monascus* strains and production techniques have been developed recently, including metabolic engineering to eliminate the production of citrinin by disrupting the polyketide synthase gene *pksCT* (Jia et al., 2010) or dehydrogenase gene *citE* (Ning et al., 2017), natural screening (Feng et al., 2016), mutagenesis (Kalaivani and Rajasekaran, 2014), genome shuffling (Ghosh and Dam, 2020), low pH (Kang et al., 2014), and genistein addition (Ouyang et al., 2021). With the increased safety of *Monascus*, people are becoming more interested in the health benefits of this edible fungus.

Despite the economic importance of *Monascus*, taxonomic research on this genus remains limited (Barbosa et al., 2017). *Monascus* spp. are categorized under the phylum Eumycota, subphylum Ascomycota, class Plectomycetes, order Eurotiales, and family *Monascaceae*. Morphologically, this genus generates non-porous perithecia at the top of the stem-like hypha, ascospores distributed throughout the hypha, virtually spherical to wide spherical ascospores, and transparent and oval ascospores detached from the closed capsule. In 1983 Hawksworth and Pitt updated the genus based on physiological and morphological criteria, reducing the number of recognized species to three: *M. pilosus*, *M. ruber*, and *M. purpureus* (Barbosa et al., 2017). Following the discovery of new species, the NCBI taxonomy database now contains more than twenty records on *Monascus* species.^1^ Among these, *M. purpureus* has the most heterotypic synonyms, such as *M. albidus*, *M. anka*, *M. erroneous*, and *M. rubiginosus*.

More recently, DNA sequencing and molecular phylogenetics have become essential in microbiological taxonomy. ITS (Dai et al., 2021), LSU (He et al., 2020; Higa et al., 2020), β-tubulin (Tong et al., 2022), calmodulin (He et al., 2020; Ruiz and Radwan, 2021) are frequently used for accurate species identification of close relatives among *Monascus* spp. However, phylogenetic delineation of this genus was complicated by gene region inconsistency and low support for internal nodes. Phenotype-based identification schemes in *Monascus* have been difficult to reconcile with the results obtained by ITS, partial LSU and/or β-tubulin gene sequencing (Park and Jong, 2003; Park et al., 2004; Shao et al., 2011, 2014), and the genetic identities of *Monascus* species are still under debate or are even confusing. Back in Park and Jong (2003) investigated the phylogenetic relationships among the species using sequences from the D1/D2 region of the large subunit (LSU) rRNA genes. They found that *M. ruber*, *M. pilosus*, and *M. purpureus* were closely related and clustered into the same subgroup, but *M. ruber* and *M. pilosus* were unable to be distinguished from each other. In Park et al. (2004) amplified and sequenced the ITS and partial β-tubulin genes of 17 ATCC reference strains of *Monascus* species. Still, they found that *M. pilosus* and *M. ruber* could not be differentiated using these sequences (Park et al., 2004), implying that species boundaries in *Monascus* should be reexamined. In fact, although ITS is the formally recognized fungal barcode, it sometimes does not distinguish among closely related phylogenetic species (Ruiz and Radwan, 2021). Due to insufficient phylogenetic information and gene-specific noise, one or a few loci usually yield incongruent phylogenies, resulting in several weakly supported nodes.

To advance the understanding of phylogeny on these edible fungi, a deeper sampling of larger and identical gene sets across the genome is required (Binder et al., 2013; Ruiz and Radwan, 2021). Whole-genome sequencing (WGS) has provided a significant benefit in establishing phylogenetic relationships, genetic diversity, virulence-related components, and biotechnological features (Carrillo and Blais, 2021). The widespread availability of WGS effectively eliminated data availability as a limiting factor for inferring phylogenetic trees, offering hundreds to thousands of loci for analysis, and eventually replacing molecular phylogenetics with phylogenomics (Nagy and Szollosi, 2017). As the available genomes of *Monascus* spp., the first WGS project (*M. ruber* NRRL 1597, SRR1800507, Illumina HiSeq 2000 platform) was published in 2015 as part of the JGI 1,000 Fungal Genomes Project to represent members of the ascomycete family Monascaceae, while the first sequence assembly was deposited in NCBI database in 2017 (*M. ruber* ASM90018405v1) (Chen et al., 2015), currently up to more than 10 deposits. Only a few have been assembled to the chromosomal level and functionally annotated (Yang et al., 2015).^2^ In the research conducted by Yang et al. (2015) they used 2,053 single-copy orthologs across the genome of *M. purpureus* YY-1 and other 17 genomes from the class Eurotiomycetes and Sordariomycetes to create a phylogenetic tree by the maximum-likelihood method, revealing the *M. purpureus* YY-1’s close relationship with the family Aspergillaceae. In another study, Higa et al. (2020) compared the biosynthetic gene clusters (BGCs) of MK, citrinin, and MonAzPs using WGS of *M. pilosus* NBRC 4520, *M. purpureus* NBRC 4478, and *M. ruber* NBRC 4483. They found that *M. pilosus* and *M. purpureus* are chemotaxonomically distinct while *M. ruber* has similar secondary metabolite BGCs to *M. pilosus*. The genome-scale approach to microbial taxonomy obviously offers improved resolution, stability, and reliability in evolutionary analyses compared to established methods of identifying physiologically and biochemically changeable and monogenic markers. Unfortunately, no phylogenomics studies on *Monascus* have been conducted within this genus.

On the other hand, WGS enables us to more fully comprehend the intrinsic mechanisms that underlie the phenotypic variations in nutritional profile, pathogenicity, host specificity, secondary metabolite synthesis and so on (San et al., 2019). Genomes within a species or all strains within a clade frequently have a core/conserved component as well as a variable set of genetic material that is referred to as a “pan-genome” among individuals or populations (Barber et al., 2021). The core genome comprises sequences present in all strains and is typically linked to biological functions and key phenotypic traits of the species. The variable/accessory/dispensable genomes contain sequences that are unique to one strain or subset of strains and are related to the adaptability of the species to specific environments or unique biological characteristics, reflecting the characteristics of the individuals. The size of the pan-genome is determined by the effective population size, lifestyle, and niche heterogeneity of the species. The gene pool of a species largely controls its ecological interactions and adaptive capacity, with core and variable genes contributing to the presence-absence variations (PAVs) (Siren et al., 2021). Therefore, a pan-genome of *Monascus* can allow us to better understand the relationship between individual characteristics and genetic variation within this species.

To expand the understanding of the phylogenetic relationships and metabolic divergence in *Monascus* species, this study collected 15 genome assemblies from the genus. They were compared at the whole-genome level using average nucleotide identity (ANI), whole-genome alignment (WGA), PAVs of the pan-genome, species tree inferred from all orthologous gene sequences (STAG), and species tree from single-copy orthologous genes (SCOG). Additionally, this study investigated noteworthy characteristics of *Monascus*, such as secondary metabolite biosynthetic gene clusters (BGCs), carbohydrate active enzymes, secretome, and pathogenicity that occur in this genus’ genomes. This approach moves away from a single reference genome that may not necessarily represent the species as a whole, and allows for better understanding of its metabolic versatility, ultimately leading to better management of these food microorganisms.

## 2. Materials and methods

### 2.1. Collection of genome assemblies

All available sequenced *Monascus* genomes defined by the taxonomically united genome database in NCBI^3^ were collected, resulting in a collection of 15 genomes (July 2022). The collection included a complete assembly (GCA_003184285.1 of *M. purpureus* YY-1) and 14 assemblies labeled as scaffold or contig. Table 1 summarized several key features for the 15 *Monascus* genomes. The completeness of genome assemblies were assessed by BUSCO (version 5.4.3) (Manni et al., 2021) with a reference set of single-copy orthologs of fungi (fungi_odb10)^4^ and default parameters.

**TABLE 1:** Genome assembly information of *Monascus* spp. included in this study.

Strain	Accession	Num_contigs	Length (bp)	N50	L50	N90	L90	GC_content (%)
*M. purpureus* CSU M183	GCA_019320005.1	69	23,752,195	1,018,695	8	272,101	24	49.43
*M. purpureus* PF1702S	GCA_023624875.1	341	23,230,699	132,158	46	42,757	166	48.99
*M. purpureus* GB01	GCA_004359145.1	122	24,325,354	327,499	19	91,302	70	48.94
*M. purpureus* P7048 × 2	GCA_023624895.1	300	23,295,031	145,302	48	44,858	160	49.03
*M. purpureus* HQ1	GCA_006542485.1	578	23,216,438	90,089	77	25,331	252	49.02
*M. purpureus* YJX8	GCA_011319195.1	14	24,529,005	3,318,727	3	1,996,896	7	48.86
*M. purpureus* YY-1	GCA_003184285.1	8	24,147,356	2,821,034	4	2,248,774	7	45.23
*M. purpureus* RP2	GCA_023935125.1	24	24,403,261	3,297,839	4	975,033	8	48.84
*M. ruber* KACC 46666	GCA_024449045.1	13	25,909,023	3,185,132	4	1,567,545	9	48.84
*M. ruber* FWB13	GCA_002976275.1	23	26,287,179	3,364,862	4	1,128,020	9	48.91
*M. ruber* GA^a^	GCA_900184055.1	198	24,882,894	314,215	24	96,185	74	47.93
*M. pilosus* YDJ-1	GCA_018806905.1	11	26,137,741	3,368,137	4	2,535,095	7	48.9
*M. pilosus* YDJ-2	GCA_018806955.1	8	26,144,475	3,479,861	4	2,422,675	7	48.89
*M. pilosus* K104061	GCA_018806895.1	10	26,125,137	3,364,842	4	2,535,067	7	48.87
*M. pilosus* MS-1	GCA_018806995.1	11	26,196,030	3,510,661	4	2,260,563	8	48.89

^a^Original record as Monascus ruber genome assembly (GCA_900184055.1).

### 2.2. ANI and WGA

Average nucleotide identity is a metric used to compare genetic relatedness of two genomes at the nucleotide level, particularly among strains that belong to the same species or a close phylogenetic clade (Yoon et al., 2017). The ANI values of *Monascus* genomes were calculated using fastANI (version 1.33) (Jain et al., 2018) with the parameter -fragLen set to 500 bp. The resulting ANI matrix was subjected to clustering analysis using the method of compete with squared Euclidean distance metrics in R. Minimap2 is a tool for fast and accurate pairwise alignment of nucleotide sequences. It can align short reads, long reads, assemble contigs, and complete genomes (Li, 2018). To perform assembly level alignments, we used minimap2 with the parameters -c, -cx, and asm5, and visualized the results using the R package pafr.

### 2.3. Genome annotation

Funannotate (version 1.8.14) (Palmer and Stajich, 2020) was used to perform genome cleaning, FASTA header sorting, repeat sequence masking, and gene prediction on *Monascus* assemblies. The reference protein sequences were collected by integrating the Funannotate protein models and the *Monascus* proteomes from UniProt. The weight of *ab initio* gene predictors, including Augustus, snap, glimmerHMM, and GeneMark-ES/ET, was set to 1:1:8:8.

### 2.4. Construction of *Monascus* pan-genome

The construction of *Monascus* pan-genome was implemented as follows. OrthoFinder (version 2.5.4) (Emms and Kelly, 2019) was used to perform all-against-all sequence similarity searches using Blastp among the predicted protein sequences generated by Funannotate. In the OrthoFinder workflow, orthogroups were generated with Markov Cluster Algorithm clustering method. OrthoFinder output files (Orthogroups folder) were used to extract the pan-genome (the total orthogroups across strains), core genome (orthogroups present at all strains), and accessory genome (orthogroups present at more than one strain but not all). The pan-genome’s gene presence-absence variation (PAV) matrix was then subjected to hierarchical clustering in R using the complete method and squared Euclidean distance metrics.

Two strategies were utilized to construct genome-wide phylogenetic trees based on orthologous proteins. The initial approach involved using the STAG algorithm, integrated within OrthoFinder (Emms and Kelly, 2018), with all orthologous protein sequences and default settings. For the second strategy, a species tree was constructed using SCOG protein sequences, which involved the following steps: (1) aligning the sequences in the OrthoFinder output folder (single_copy_orthologous) using muscle (version 5.1) (Edgar, 2004); (2) extracting conserved sequences with Gblocks^5^; (3) converted.fa format to.phy using MEGA (version 7) (Kumar et al., 2016); (4) predicting a suitable amino acid substitution model with ProtTest (version 3) (Darriba et al., 2011) and finally constructing the maximum likelihood phylogenetic tree with RAxML (version 8.2.12) (Stamatakis, 2014).

### 2.5. Functional gene annotation

#### 2.5.1. Carbohydrate-active enzymes (CAZymes)

Carbohydrate-active enzymes (Cantarel et al., 2009) describes the families of structurally related catalytic and carbohydrate-binding modules (or functional domains) of enzymes involved in the synthesis and degradation of complex carbohydrates and glycoconjugates. To perform the CAZy annotation, a local dbCAN (Yin et al., 2012) was employed using HMMER (version 3.2.2) searching against a hidden Markov model database of CAZyme domains derived from CDD and CAZy (Potter et al., 2018).

#### 2.5.2. Prediction of secreted proteins

Secreted proteins are proteins exported from the cell by a specific pathway. A secreted protein was defined in this study as a protein with a secretory signal peptide but no transmembrane domains, mitochondrial localization, or chloroplast localization. The prediction of secreted protein was as follows. To predict secreted proteins, we used SignalP6 (Teufel et al., 2022) to identify the presence and location of signal peptides in protein sequences, TMHMM (Sonnhammer et al., 1998) to predict transmembrane domains, and TargetP (Emanuelsson et al., 2007) to determine the subcellular localization of proteins based on their N-terminal sequence features and to provide a potential cleavage site.

#### 2.5.3. Virulence factor annotation

Fungal VFDF database (Lu et al., 2012) describes the virulence factors in fungal pathogens that contains information on the genes, proteins, functions, mechanisms, and pathways of virulence factors from various fungal species that cause human and plant diseases. To perform VFDF prediction, all the proteins in the DFVF database were downloaded and aligned them with the secreted protein sequences using Blastp program.

#### 2.5.4. Prediction of the BGCs of secondary metabolites

The BGCs of citrinin, MK, and the MonAzPs was identified and annotated using the tool antisMASH (Blin et al., 2021). The visualization of genetic cluster was implemented with R package genes.

#### 2.5.5. Gene Ontology (GO) annotation and enrichment analysis

Gene Ontology annotation was performed using the online webtool eggnog-mapper^6^ (Cantalapiedra et al., 2021). GO enrichment and visualization were implement in Cytoscape (version 3.8.3) (Shannon et al., 2003) using BiNGO plugin (Maere et al., 2005).

Unless otherwise specified, a 40% sequence identity cutoff was used for protein functional annotation.

## 3. Results

### 3.1. Genome information and assessment of assembly quality

**FIGURE 1:**
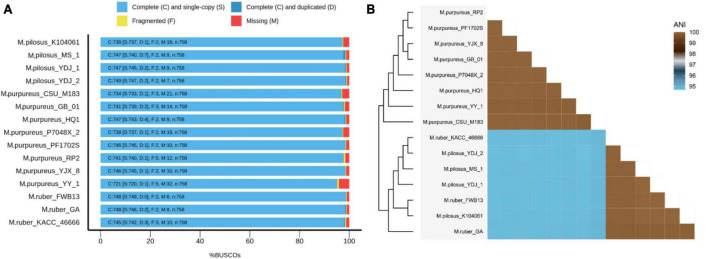
BUSCO evaluation on the genome assembly quality **(A)** and ANI-based genomic relatedness among *Monascus* spp. **(B)**.

Fifteen representative genomic assemblies of the *Monascus* genus were downloaded from the NCBI assembly database^7^ and classified into three species, namely, *M. ruber*, *M. pilosus*, and *M. purpureus* (Table 1). The sizes of the assemblies ranged from 23.2 to 26.3 Mb, with GC contents varying from 45.23 to 49.43%. The BUSCO evaluation indicated that more than 95% of the 758 single-copy gene homologs were completely assembled in these genomes (Figure 1), indicating high quality of genome assembly (Manni et al., 2021).

To reannotate these fungal genome assemblies, the Funannotate script (Palmer and Stajich, 2020), a well-streamlined annotation tool, was used. The resulting annotations (Table 2) revealed that each genome encodes 8,103–9,030 genes, with an average protein length of 484.33–495.67 amino acids. Interestingly, the total number of genes encoded by the *M. pilosus* or *M. ruber* genomes was higher than 8,800, more than those of *M. purpureus*.

**TABLE 2:** *Monascus* annotation information.

Strain	Num_genes	Num_mRNA	Num_tRNA	Avg_gene length	Total exons	Avg_exon length	Avg_protein length
*M. purpureus* CSU M183	8,498	8,365	133	1603.21	24,434	442.64	488.7
*M. purpureus* PF1702S	8,391	8,255	136	1608.37	24,303	441.44	490.7
*M. purpureus* GB01	8,480	8,339	141	1627.85	25,039	438.75	495.4
*M. purpureus* P7048 × 2	8,377	8,250	127	1629.75	24,845	437.07	495.67
*M. purpureus* HQ1	8,441	8,320	121	1598.75	24,280	441.17	487.08
*M. purpureus* YJX8	8,633	8,482	151	1602.5	24,831	442.07	489.54
*M. purpureus* YY-1	8,103	7,984	119	1629.66	23,986	438.32	491.45
*M. purpureus* RP2	8,462	8,311	151	1629.64	25,178	437.68	495.51
*M. ruber* KACC 46666	8,896	8,745	151	1595.31	25,763	436.85	484.79
*M. ruber* FWB13	9,030	8,867	163	1591.81	26,269	436.52	485.13
*M. ruber* GA	8,807	8689	118	1602.66	25,568	437.2	485.78
*M. pilosus* YDJ-1	8,954	8,814	140	1589.74	25,599	440.86	484.33
*M. pilosus* YDJ-2	8,838	8,704	134	1618.85	25,975	436.7	491.18
*M. pilosus* K104061	8,931	8,771	160	1592.98	25,610	440.66	486.15
*M. pilosus* MS-1	8,853	8,705	148	1619.35	26,023	437.84	492.41

### 3.2. ANI analysis

The whole-genome average nucleotide identity (ANI) is a reliable method to determine the genetic relatedness of two genomes and evaluate the boundaries between species with prokaryotic organisms from the same species typically showing 95% ANI among themselves (Jain et al., 2018). Recently, ANI analysis has also been used to assess relationships between eukaryotic genomes, such as yeasts (Lachance et al., 2020), microsporidia (de Albuquerque and Haag, 2023), and plankton species (Delmont et al., 2022). In this study, ANI analysis revealed that the 15 *Monascus* strains could be delineated into two distinct clades, the *M. purpureus* clade and the *M. ruber-M. pilosus* clade (Figure 1B). ANI values within each clade were greater than 99.86%, while those from different clades were less than 94.85%. Although 95% ANI is not yet accepted as the species boundary in eukaryotes, an ANI close to 100% suggested a high degree of overall genomic sequence identity and indicates that two genomes share a large proportion of similar DNA sequences.

### 3.3. Whole-genome alignment (WGA)

**FIGURE 2:**
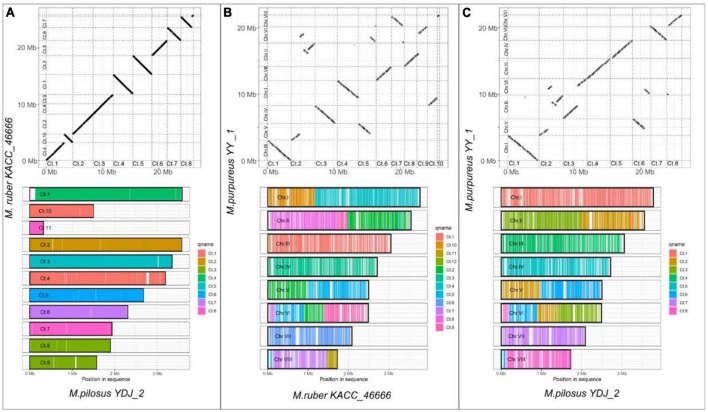
Whole-genome alignment on *Monascus ruber* KACC 46666, *M. pilosus* YDJ2 and *M. purpureus* YY1. **(A)** *M. ruber* KACC 46666 vs. *M. pilosus* YDJ2; **(B)** *M. ruber* KACC 46666 vs. *M. purpureus* YY1; **(C)** *M. pilosus* YDJ2 vs. *M. purpureus* YY1. The upper panel is the scatter diagram of genome collinearity and the lower panel describes the chromosome coverage. Other whole-genome alignments are supplied in Supplementary Material 1.

In contrast to ANI, which measures similarity of query genome fragments are to their homologous counterparts in the subject genome (Yoon et al., 2017), WGA focuses on predicting evolutionarily related sequence positions and identifying large-scale structural changes, such as duplications and rearrangements (Dewey, 2012). For WGA analysis in this study, the less fragmented genomes from three species with high assembly integrity were chosen, i.e., *M. purpureus* YY-1, *M. pilosus* YDJ2, and *M. ruber* KACC 46666. The remaining WGA analysis outputs were included in the Supplementary Material 1. Figure 2 illustrated the homologous regions that had undergone DNA translocation, rearrangement, or recombination, resulting in them being scrambled or inverted. Missing genome regions were represented by the gaps in the alignments, and Figures 2B, C clearly showed chromosome rearrangements. Genomes of *M. ruber* KACC 46666 and *M. pilosus* YDJ2 exhibited an incredibly strong collinearity, as shown in Figure 2A, with no significant genomic insertions, conversions, or translocations found in either strain, except for 5 fragment deletions and inversions. Conversely, numerous fragment inversions and DNA translocations were found between *M. purpureus* YY-1 and *M. pilosus* YDJ2, as well as *M. purpureus* YY-1 and *M. ruber* KACC 46666, resulting in low similarity and poor linear match. Distinct chromosomal rearrangement events were detected in chromosomes 1, 2, 5, 6, and 8 between *M. purpureus* YY-1 and *M. ruber* KACC 46666, while chromosome 1 of *M. purpureus* YY-1 was a fusion of the inverted Ct.4 of *M. ruber* KACC 46666 and the translocated Ct.10. Overall, WGA revealed that *M. ruber* KACC 46666 had better collinearity with *M. pilosus* YDJ2, and they were more closely related.

### 3.4. *Monascus*’ pan-genome

**FIGURE 3:**
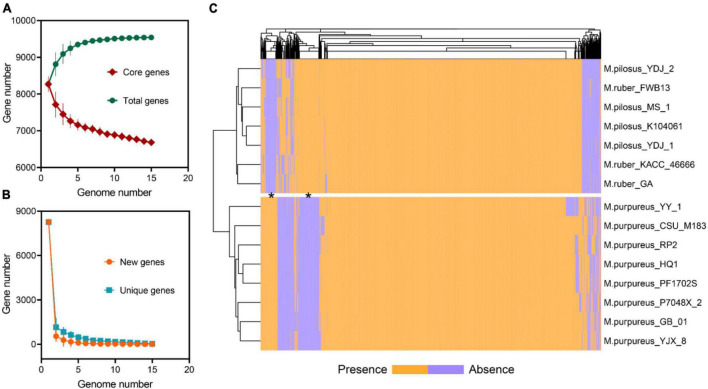
Pan-genome of *Monascus spp*. **(A)** Total and core orthogroups along with the increase of *Monascus* genome number; **(B)** new and unique orthogroups; **(C)** visualization of PAVs and hierarchical clustering.

The pan-genome of *Monascus* spp. was constructed using OrthoFinder (Emms and Kelly, 2019). All proteins identified from the 15 genomes were used to infer orthologous protein clusters (orthogroups) (Figure 3A). With the addition of more genome assemblies, the core genome size decreased while the pan-genome size increased. The “open form” idea of Heap law (Kadiri et al., 2023) was reflected as more orthologous families were included in the analysis (Figure 3B). On the whole, the pan-genome encompassed 9,539 orthogroups, of which 6,683 (70.06%) had been converged as the core genome, while the remaining were variable and exhibited the PAV feature, which was an important source of genetic divergence and diversity, as well as having profound effects on phenotypic variations (Wang et al., 2014). Visualization of PAV and hierarchical clustering revealed that the 15 *Monascus* strains could be divided into two major groups, the *M. purpureus* clade and the *M. ruber*-*M. pilosus* clade (Figure 3C), consistent with ANI-based clustering.

**FIGURE 4:**
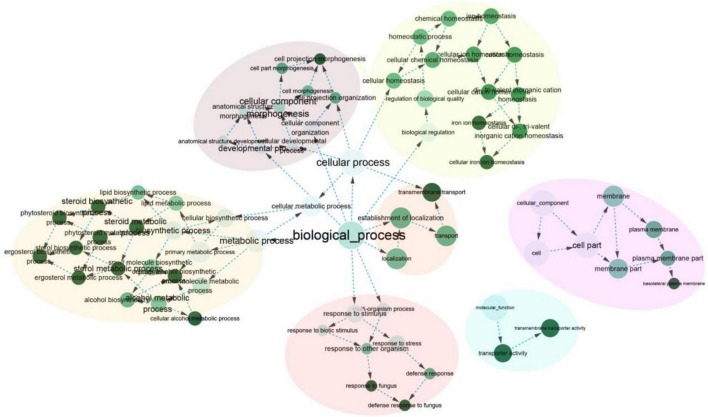
GO enrichment of the unique orthogroups in *M. ruber*-*M. pilosus* clade. The analysis was based on the genome of *M. ruber* FWB13 (GCA_002976275.1) as a representative. Additional GO enrichment outputs of the other strains were available in Supplementary Material 2.

Furthermore, 277 orthogroups were found only in *M. purpureus* strains, while 546 were found only in *M. pilosus*-*M. ruber* strains marked by asterisk in Figure 3C. Using the unique orthogroups from the *M. ruber*-*M. pilosus* clade and the *M. purpureus* clade, we conducted GO enrichment analysis on each strain separately. However, the unique orthogroups associated with the *M. purpureus* clade did not enrich into any GO categories (corrected *P* > 0.05), indicating a more stochastic and discrete occurrence of these genes (Supplementary Material 2). In contrast, the *M. ruber*-*M. pilosus* clade’s unique genes were mainly involved in three categories: as shown in Figure 4 (1) Biological processes, such as transport and localization, stimulus response, cellular component organization, and regulation of cellular homeostasis; (2) Molecular functions, such as transport activities; (3) Cellular components, such as plasma membrane. Based on these findings, it can be concluded that the strains from *M. ruber*-*M. pilosus* clade had a stronger ability to transport and maintain cellular homeostasis than the strains from the *M. purpureus* clade, enabling them to better adapt to changing living environments.

### 3.5. Phylogenetic analysis at the genome level

**FIGURE 5:**
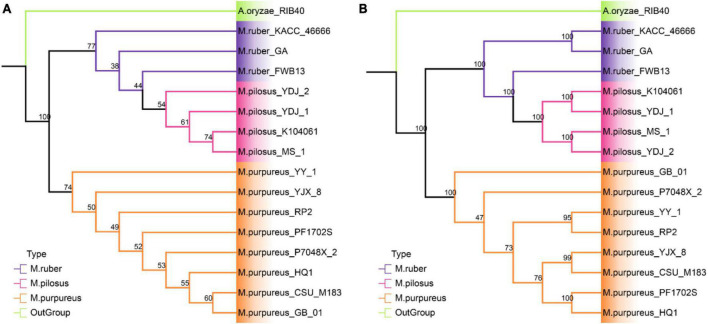
Phylogenetic tree at genome level. **(A)** STAG tree with 5565 orthologous protein sequences; **(B)** SCOG tree with 4589 single-copy orthologous sequences.

The phylogenetic relationships among the 15 strains in *Monascus* species were investigated using two different approaches (Figure 5). *A. oryzae* RIB40 was the only outgroup species included because both *Aspergillus* and *Monascus* belong to the Aspergillaceae family, and more distant outgroup taxa would likely further reduce the percent coverage of orthogroups present in all species. The first approach, STAG created a rooted phylogenomic tree using OrthoFinder, which utilized 5.565 single- and multi-copy orthologous protein sequences found in all the genomes (Figure 5A). The support values for each bipartition in a consensus STAG tree are the proportion of times that the bipartition is seen in each of the individual species tree estimates (Emms and Kelly, 2018). Meanwhile, the second approach, SCOG, employed RAxML to construct another phylogenetic tree using 4,589 single-copy orthologs (Figure 5B) by JTT + I + G + F amino acid substitution model. Node support was estimated with 1,000 bootstrap replicates.

The STAG phylogenetic tree identified two distinct binary clades with a support value of 100%, the *M. purpureus* clade and the *M. ruber-M. pilosus* clade (Figure 5A). Similarly, the SCOG tree demonstrated a comparable topology with a 100% support for these two clades (Figure 5B), indicating that species trees inferred from all orthologous proteins were as accurate as those inferred from single-copy orthologs. However, the SCOG tree showed significant advantages in supporting sub-branches due to higher support values. In the STAG tree, most of the nodes within both the *M. purpureus* and *M. ruber-M. pilosus* clades had low supports (<70%), which made the topology of the sub-branches unreliable. In contrast, only one node within the *M. purpureus* clade in the SCOG tree was unsound. Moreover, *M. ruber* FWB13 was a unique presence in the *M. ruber-M. pilosus clade*, forming a monophyletic group with four *M. pilosus* species but a paraphyletic group with the other two *M. ruber* species. This topology was also evident in the STAG tree, where *M. ruber* FWB13 occupied a similar phylogenetic position.

Overall, the phylogenomic trees based on these two approaches confirmed the results of ANI and PAV clustering as well as verifying the deduction from genome collinearity analysis.

### 3.6. Comparative functional genome within the *Monascus* species

To further compare the metabolic divergences among the 15 strains, the protein sequences of all strains were annotated with CAZy, and secretome. And the BGCs of second metabolite in the genome were identified and characterized.

#### 3.6.1. CAZyme annotation

**FIGURE 6:**
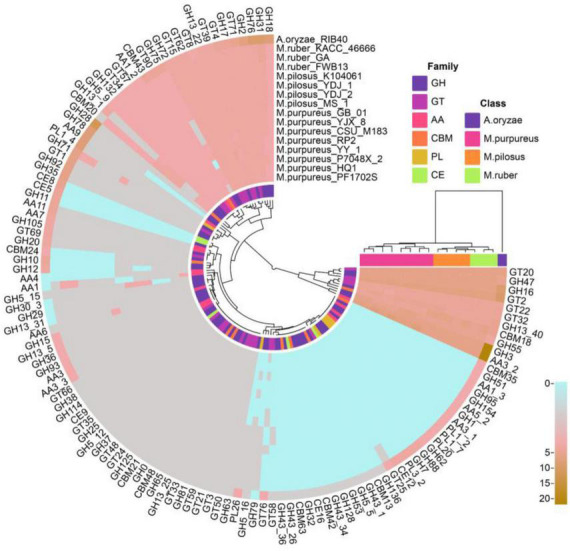
CAZY annotation of *Monascus* proteomes. Proteins of sequence identity < 40% were filtered.

A total of 2,542 coding genes in these 15 genomes were annotated as CAZymes, including 246 auxiliary activity proteins (AAs), 172 carbohydrate-binding modules (CBMs), 15 carbohydrate esterases (CEs), 1,241 glycoside hydrolases (GHs), 846 glycosyltransferases (GTs), and 22 polysaccharide lyases (PLs). Compared to *A. oryzae*, *Monascus* showed a weaker carbohydrate utilization capacity due to the absence or fewer copies of CAZymes. Although *Monascus* species are able to use starch substrates, such as rice, for growth and metabolism, only α-amylase of GH13 family (EC 3.2.1.1, splitting the α-1,4 glycosidic linkages in amylose to yield maltose and glucose) was found, while β-amylases, which cleave β-maltose at the non-reducing end of starch, were absent in all genomes, as shown in Figure 6.

Moreover, *M. purpureus* genomes contained more copies of endoglucanases (EC 3.2.1.4, GH5∼GH10) and β-1,3-glucosidase (EC 3.2.1.-, GH132) than *M. ruber* or *M. pilosus*. Endoglucanase is a cellulase family member that has a higher affinity for cellulose and also acts on xylan and mixed β- (1–3, 1–4)-glucan, while β-1,3-glucosidase catalyzes the hydrolysis of β(1→3)-glucosidic linkages in β(1→3)-d-glucan, which is the main constituent of fungal cell walls (Ramos and Malcata, 2011).

Auxiliary activity proteins family members, such as AA4 vanillin oxidase (VAO, EC 1.1.3.38) were only found in the *M. purpureus* genome. VAO is a fungal flavoenzyme that converts a wide range of para-substituted phenols and is the only known fungal member of the 4-phenol oxidizing subgroup of the VAO/PCMH flavoprotein family (Gygli et al., 2018).

In addition, GH5_16, GH78, and GH79 family members were only found in *M. ruber*-*M. pilosus* genomes. GH5_16 is an endo-1,6-β-galactanase (EC 3.2.1.164) that hydrolyzes 1,6-β-D-galactooligosaccharides with a polymerization degree of more than three and their acidic derivatives with 4-*O*-methylglucosyluronate or glucosyluronate groups at the non-reducing ends (Zhang et al., 2022). GH78 glycoside hydrolases hydrolyze α-L-rhamnosides (EC 3.2.1.40) and degrade flavonoid glycosides that are common in human diets and have important applications in food and medicine industries (O’Neill et al., 2015). GH79 glycoside hydrolases are widely distributed in eukaryotes such as fungi, plants, and animals as well as bacteria and their known members include β-glucuronidase (EC 3.2.1.31), baicalin β-glucuronidase (EC 3.2.1.167), and heparanase (EC 3.2.1.166) (Zhu and Tang, 2021).

#### 3.6.2. *Monascus*’ secretome and allergen proteins

**FIGURE 7:**
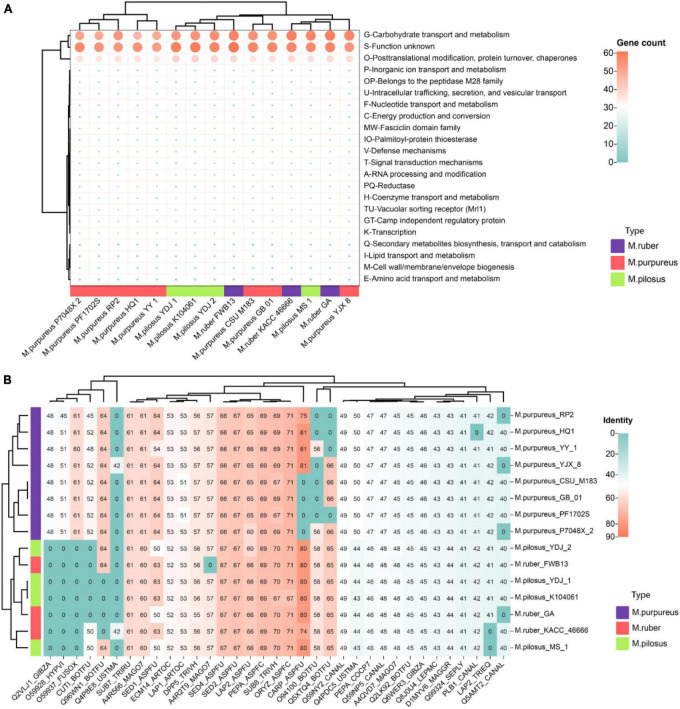
*Monascus*’ secretome **(A)** and fungal virulence proteins **(B)**. Proteins of sequence identity < 40% were filtered.

The “secretome” refers to the complete collection of proteins secreted by microorganisms that perform various functions such as digestion, signaling, and defense (Caccia et al., 2013). Notably, the top-ranked annotation items for *Monascus* covered carbohydrate transport and metabolism (G), post-translational modification, protein turnover, chaperones (O), and function unknown (S) (Figure 7A), including lipase, acid phosphatase, glycoside hydrolases, aspartic-type endopeptidase activity (GO:0004190), and protein hydrolysis serine-type endopeptidase activity (GO:0006508). Some secreted proteins allergenic, such as allergen (orthologous to CADAFLAP00008692), allergen Asp (orthologous to CADAFUBP00002039, Asp F4), allergen Asp F7 (orthologous to V5GFQ9). Of them, CADAFLAP00008692 is a putative allergen from *A. flavus.* Asp F4 is associated with allergic bronchopulmonary aspergillosis (Ramachandran et al., 2004), while Asp F7 from *A. fumigatus* is a peroxiredoxin, a major fungal allergen known for its function as a virulence factor candidate vaccine and reactive oxygen scavenger (Blanco-Ulate et al., 2013). Additionally, all 15 genomes had a defensive secreted β-lactamase, an enzyme that confers resistance to penicillins, cephalosporins, and monobactams (Livermore, 1998).

Given the presence of allergens in secreted proteins and the food safety of *Monascus* products, these proteins were further annotated for fungal pathogens using the DFVF database. It’s worth noting that 35 secreted proteins (7.8% of total secretome) in *Monascus* were predicted to be involved in virulence and pathogenicity (Figure 7B). Among them, CARP_ASPFU had high identity ≥ 80% present in the genomes of *M. pilosus*, *M. ruber* (except KACC 46666), *M. purpureus* HQ1, *M. purpureus* YY-1, and *M. purpureus* YJX8. This protein is a secreted vacuolar aspartic endopeptidase with broad specificity for peptide bonds protein hydrolysis, and has an important function in allergen processing and causing various rare human infections such as lung aspergillosis and mycotic keratitis (Jehangir and Ahmad, 2004).

#### 3.6.3. Secondary metabolic synthetic genetic cluster

**FIGURE 8:**
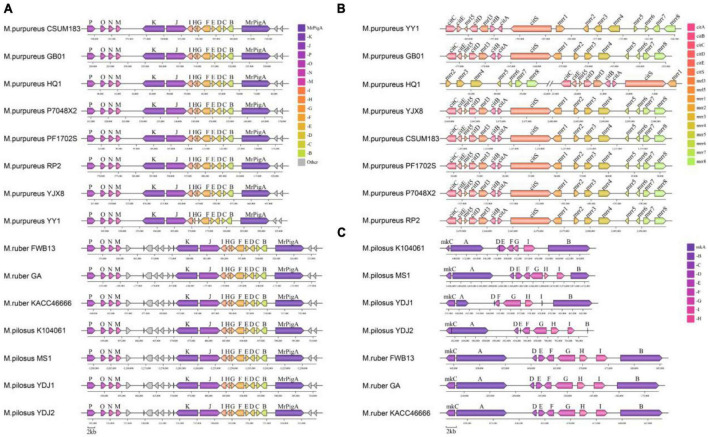
Organization of the secondary metabolic synthetic genetic cluster. **(A)** MonAzPs BGCs; **(B)** citrinin BGCs; **(C)** MK BGCs. Negative coordinate values represent the reversed redirection of BGCs.

Monascus azaphilone pigments, MK and citrinin are the most concerned metabolites produced by *Monascus* spp. and their primary structures are synthesized by type I polyketide synthase (T1PKS). According to antiSMASH search results, the BGC responsible for producing MonAzPs was found present in each genome (reference BGC: BGC0000027.1) (Chen et al., 2019; Figure 8A). Despite this, the MonAzPs BGC in *M. purpureus* was approximately 56 kb in length, whereas those in *M. pilosus* and *M. ruber* were 65 kb and had seven non-essential genes inserted into the gene cluster. The sequences of the 15 core genes from each BGC did not differ considerably from the reference sequence (identity > 90%, Supplementary Material 3).

The reference citrinin BGC (BGC0001338) from *M. ruber* M7 contains 16 genes, including the essential genes *citS* (polyketide synthase), *citA* (serine hydrolase), *citB* [Fe(II) oxidoreductase], *citC* (oxidoreductase), *citD* (aldehyde dehydrogenase), and *citE* (short-chain dehydrogenase) (He and Cox, 2016). In this study, only eight *M. purpureus* strains had the complete citrinin gene cluster sequence (Figure 8B). Except for *M. purpureus* HQ1, the citrinin gene cluster size was approximately 42 kb, and the core gene cluster (total length of the key sequences responsible for citrinin synthesis) was around 20 kb. The gene cluster from *M. purpureus* HQ1 was split into two fragments, with *citS-citA-citB-mrr3-citD-mrr5-citE-citC* present in the contig of VIFY01000224.1 and *mrr-8-mrr7-mrr6-mrr5-mrr-4-mrr3-mrr2* in VIFY01000166.1. Nevertheless, the sequence between these eight gene clusters and the reference gene cluster was highly conservative (identity > 95% for pairwise homologous genes). Additionally, several non-core genes, including *mrr2*, *mrr3*, *mrr4*, *mrr5*, *mrr6*, *mrr7*, and *mrr8*, were discovered in *M. ruber* and *M. pilosus* genomes, but all the core genes were absent (Supplementary Material 3).

The MK BGC, which typically contains nine genes (*mokA* to *mokI*) identified in BGC0000098 from *M. pilosus* based on sequence similarity (Zhang et al., 2020). In this study, the MK BGC was identified in seven genomes of *M. ruber* and *M. pilosus*, with a size of approximately 41 kb in *M. ruber* and 27 kb in *M. pilosus*. The gene sequences of the BGCs in *M. ruber* FWB13, *M. ruber* KACC 46666, *M. ruber* GA, and *M. pilosus* MS-1 were found to be highly similar to the reference BGC. *M. pilosus* YDJ1, *M. pilosus* YDJ2, and *M. pilosus* K104061 were isolated from commercial MK products and found to be capable of producing MK (Dai et al., 2021), but antiSMASH or BLAST searches revealed incomplete BGC sequences in their genomes. *MokH* was absent in *M. pilosus* K104061, and *MokE* was missing from *M. pilosus* YDJ1’s BGC. Furthermore, *MokB* and *MokE* from *M. pilosus* YDJ2, as well as *MokD* and *MokI* from *M. pilosus* YDJ1, were truncated compared to the other six homologous genes. These findings implied that MK synthesis might have diverged in different strains, and additional evidence for critical enzymes in MK synthesis was required in *Monascus.*

## 4. Discussion

*Monascus* species are widely found in various habitats such as soil, starch, grain, dried fish, surface sediments of rivers, and roots of pine trees (Mohan Kumari, 2009). Due to the production of MonAzPs and MK, these filamentous fungi are frequently used in food and medicine. However, the taxonomy of *Monascus* has long been a matter of confusion. The phenotype-based identification schemes in *Monascus* were difficult to match with the results obtained by ITS, partial LSU and/or β-tubulin gene sequencing (Patakova, 2013), especially in the case of single-gene locus-based phylogenetics. For example, Dai et al. (2021) inferred a Neighbor-Joining tree using ITS sequences, which revealed that several isolates from *M. pilosus*, *M. fuliginosus*, *M. barkeri*, *M. paxii*, *M. albidulus*, *M. ruber*, *M. purpureus*, and *M. fumeus* were evolutionarily close in the same clade with high bootstrap values. In another clade, several strains from *M. purpureus*, *M. rutilus*, *M. aurantiacus*, and *M. kaoliang* were clustered. Within these two main clades, the strain-level division was still vague due to the low support level.

To overcome inappropriate taxonomy of fungi caused by a single locus, the genealogical concordance phylogenetic species recognition concept (GCPSR) was proposed as an empirical method for recognizing cryptic speciation (Taylor et al., 2000). GCPSR involves sequencing multiple genes that are then combined in phylogenetic analyses. In a report conducted by He et al. (2020) a phylogenetic tree was constructed using concatenated sequences of five protein genes (*BenA*, *CaM*, *RPB2*, *pksKS*, and *MAT1-1*) and two ribosomal RNA genes (ITS and LSU) with a total length of 6,983 bp. Strains from *M. ruber* and *M. purpureus* were clustered into different species clades, respectively, with a high Bayesian analysis/bootstrap percentage. Unfortunately, *M. pilous* was not included in their phylogenetic analysis. In 2017, Patakova divided the *Monascus* spp. into section Floridani and Section Rubri by concatenated phylogeny based on the sequences of ITS + BenA + CaM + LSU + RPB2 with Bayes/RAXML method (Patakova, 2013). *M. ruber*, *M. pilous* and *M. purpureus* were all in the section Rubri, and *M. purpureus* was located in a separate subclade from *M. ruber* and *M. pilous* with high Bayes/RAXML support. *M. ruber* and *M. pilous* were clustered into the same evolutionary branch.

Recently, modern phylogenetic analyses utilize hundreds to thousands of sites from throughout the genome, which are orders of magnitude larger than traditional sequencing datasets. Thus, the size of these datasets significantly reduces the impact of random error and data availability, making them promising for addressing historically recalcitrant nodes in the tree of life. In this study, using the *Monascus* assembly deposits from the NCBI genome database, a genome-level phylogenetic analysis was conducted. These 15 genomes were predominantly descended from two clades, the *M. purpureus* clade and the *M. ruber-M. pilous* clade, from either the SCOG tree according to the concatenated phylogeny based on 4,589 single copy protein orthologs or the STAG analysis with all the 5,565 single-/multiple- copy protein orthologs. Both of the phylogenomic analysis strategies were reliable with 100% support values for the two major clades. Furthermore, evidence from the nucleic acid level was proposed to support this conclusion, including analyses of average nucleotide identity and genomic collinearity. In particular, there was considerable genomic collinearity between *M. ruber* and *M. pilous*, indicating a high degree of similarity between these two species, rather than with *M. purpureus*. However, genome collinearity analysis, which frequently relies on genome assembly quality, makes it difficult to distinguish between strains when using fragmented genome assemblies. Hence, it is essential to employ a combination of several methods from various perspectives for evaluating the similarity between genomes.

Through identification and characterization of the BGCs of *Monascus*, it was found that all genomes contain the MonAzPs BGCs. However, the citrinin BGCs were only discovered in *M. purpureus*, while the BGCs of MK were only present in *M. pilosus* and *M. ruber*. The production of the mycotoxin citrinin, was originally described in *M. purpureus* and *M. ruber* in Blanc et al. (1995). It was subsequently shown that the *M. ruber* used in that study was, in fact, *M. purpureus* (Chen et al., 2008) because the *pksCT* gene for citrinin polyketide synthase was only present in *M. purpureus* and *M. kaoliang* (a synonym for *M. purpureus*), but not in *M. pilosus*, *M. ruber*, *M. floridanus*, *M. sanguineus*, *M. barkeri*, or *M. lunisporas*. Despite this, citrinin production in *M. ruber* was later demonstrated by other authors (Li et al., 2010, 2021). However, whether or not this was due to incorrect strain classification requires additional validation. Another possibility is that this strain-specific citrinin synthesis might originate from a horizontal gene transfer of the BGC among fungi, because the citrinin pathway belongs to the general pathway shared by many *Penicillium*, *Aspergillus*, and *Monascus* species (Higa et al., 2020).

Additionally, when compared to the reference BGCs, the BGC sequences of MonAzPs and citrinin revealed the highest degree of conservation. Interestingly, seven additional inserted genes in the MonAzPs BGCs could well differentiate *M. purpureus* apart from *M. pilosus* or *M. ruber*. Furthermore, the BGCs of MK were more varied in *M. pilosus* but were conserved in *M. ruber*. Although this suggested that the three strain classes might be distinguished by divergence from the MK gene cluster sequence, further sequencing evidence from more strains is necessary to support this.

According to the findings of this study, the investigated *Monascus* species can be classified into two groups: the *M. pilosus*-*M. ruber* clade and the *M. purpureus* clade. This classification may have significant implications in *Monascus*-related industries. Typically, commercial *Monascus* products are divided into two categories, those intended for the production of MonAzPs for food coloring, and those for the production of MK, a secondary metabolite to lower cholesterol and treat hypolipidemia. Among them, *M. purpureus* is a prominent red-colored mold species (Yang et al., 2015), but a number of strains including *M. purpureus*, *M. pilosus, M. sanguineus* and *M. ruber* were reported to produce MK (Wen et al., 2020). This is confusing because it is unclear whether the synthesis of MK is due to individual differences among *Monascus* strains or incorrect classification, as the genome of all *M. purpureus* strains used in this study lacked the complete gene cluster for MK biosynthesis. Therefore, further identification of these strains at the phenotypic and genotypic levels is necessary. For example, in a recent study by Higa et al. (2020) the metabolite analysis based on liquid chromatography-mass spectrometry revealed significant differences in the MonAzPs and related metabolites produced by the three species (*M. pilosus*, *M. ruber*, and *M. purpureus*) in liquid media, despite *M. ruber* had similar biosynthetic and secondary metabolite BGCs to *M. pilosus*.

Moreover, genome-level analysis provides insights into metabolic differences among individual strains. Comparative genomic analyses showed that the genome size of *M. purpureus* was smaller than that of *M. pilosus/M. ruber* and had undergone significant gene losses, particularly in cellular transport and maintaining homeostasis, as a result of specialized adaptation to the environment. Compared to *A. oryzae*, *Monascus* also displayed gene losses in both enzyme species and quantities involved in carbohydrate metabolism, which might be due to strain degradation resulting from prolonged domestication of the starch-rich matrix (Dufossé, 2018). Furthermore, *Monascus*’ genomes were predicted to contain a number of secreted proteins that could act as allergens or be involved in virulence and pathogenicity, although they exhibit interindividual variability. One such protein is CARP_ASPFU, which is present in most of the genomes and has a high identity ≥ 80%, and may play a crucial role in processing or signaling to allergens, causing rare human infections such as lung aspergillosis and mycotic keratitis. Given the importance of food safety, it is necessary to confirm whether the toxins produced by particular *Monascus* strains are actually produced by gene expression or just exist in the genome.

## 5. Conclusion

In conclusion, *Monascus* is a widely consumed commodity strain due to its good coloring and health care efficacy. However, its taxonomic profile remains under debate and is difficult to classify using a single locus sequence alignment and comparison, as is the case with other fungi. This study utilized phylogenomics, which draws information from comparing entire or large portions of genomes, to gain more detailed insights into evolutionary relationships, as compared to traditional phylogenetic methods that rely on a smaller number of genetic markers. Our findings clearly demonstrate differences between *M. purpureus* and *M. pilosus*/*M. ruber*, as well as a high degree of similarity between the genomes of *M. pilosus* and *M. ruber*. By comparing the genomes of different *Monascus* strains, we were able to identify differences in metabolism for environmental adaptation, carbohydrate-active enzymes, secretome, fungal pathogens, as well as in secondary metabolite gene clusters. Genome-level research provides insights into the species classification of *Monascus*, and as the cost of genome sequencing decreases, this high-resolution phylogenetic method will become an important means for evaluating the safety of *Monascus* and other edible fungi.

## Data availability statement

The datasets presented in this study can be found in online repositories. The names of the repository/repositories and accession number(s) can be found in the article/Supplementary material.

## Author contributions

ZZ, HL, and XL conceived and designed the study. ZZ, MC, PC, JL, ZM, YM, ZL, QG, and CW performed the data analysis. All authors wrote the manuscript and approved the final manuscript.

## Footnotes

1
https://www.ncbi.nlm.nih.gov/Taxonomy/Browser/wwwtax.cgi?id=5097

2
https://www.ncbi.nlm.nih.gov/data-hub/genome/?taxon=5097

3
https://www.ncbi.nlm.nih.gov/genome/?term=Monascus

4
https://busco-data.ezlab.org/v4/data/lineages/

5
http://phylogeny.lirmm.fr/phylo_cgi/one_task.cgi?task_type=gblocks

6
http://eggnog-mapper.embl.de/

7
https://www.ncbi.nlm.nih.gov/data-hub/taxonomy/tree/?taxon=5097

## Supplementary material

Supplementary material 1 Whole-genome alignment on all the genomes from *Monascus* spp.

Supplementary material 2 GO enrichment of the unique orthogroups in each *Monascus* genome.

Supplementary material 3 Secondary metabolite synthetic genetic clusters in *Monascus* genome.
