# Supplementary material for: Insight into the phylogeny and metabolic divergence of Monascus species (M. pilosus, M. ruber, and M. purpureus) at the genome level
Source: Front Microbiol. 2023 May 25;14:1199144. doi: 10.3389/fmicb.2023.1199144 (PMC10249731; doi:10.3389/fmicb.2023.1199144)
Supplement: Supplementary material 1 — Whole-genome alignment on all the genomes from Monascus spp. [file Data_Sheet_1.PDF]

query.M.pilosus_K104061.fasta.ref.M.pilosus_YDJ_1.minimap

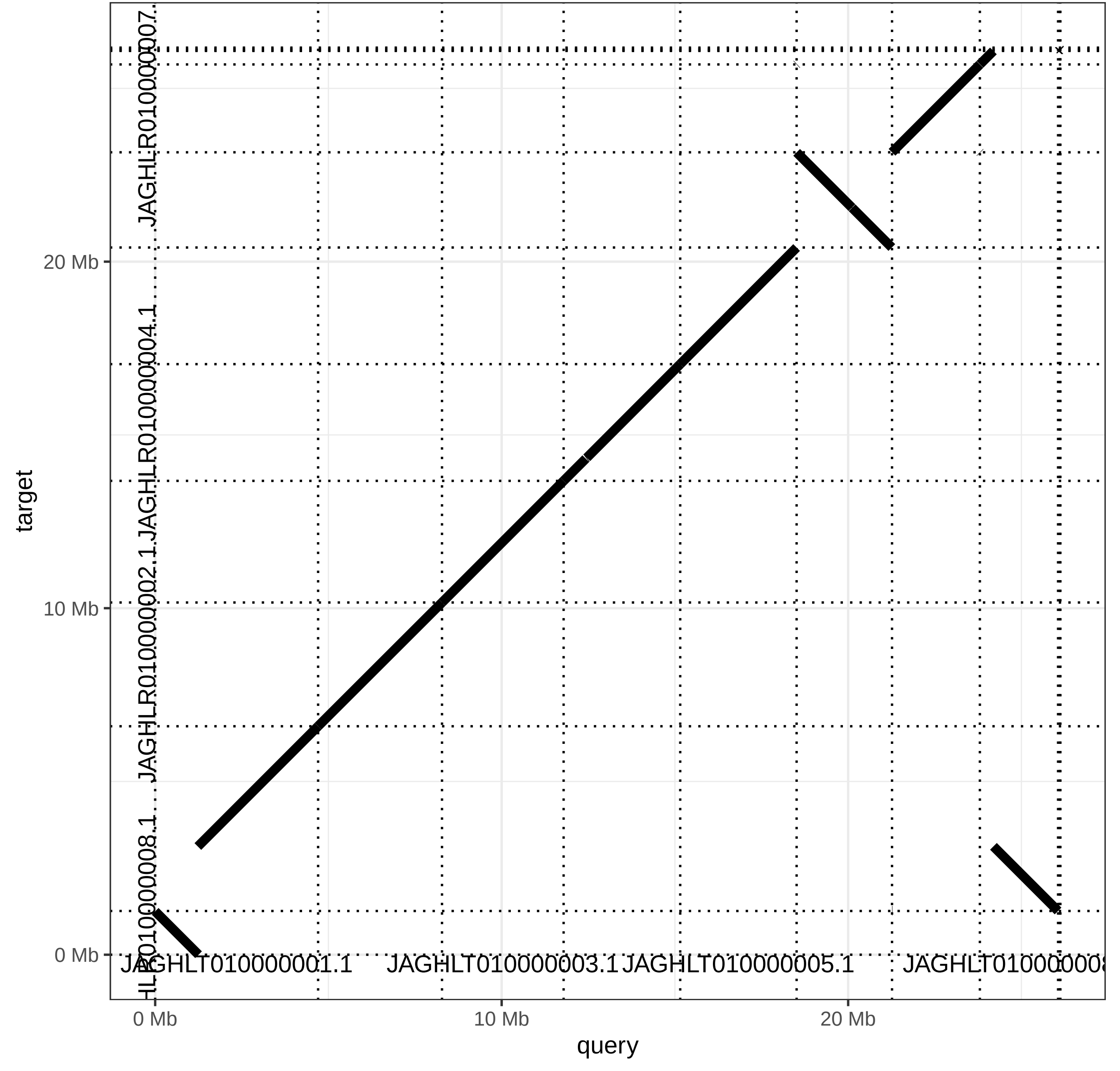

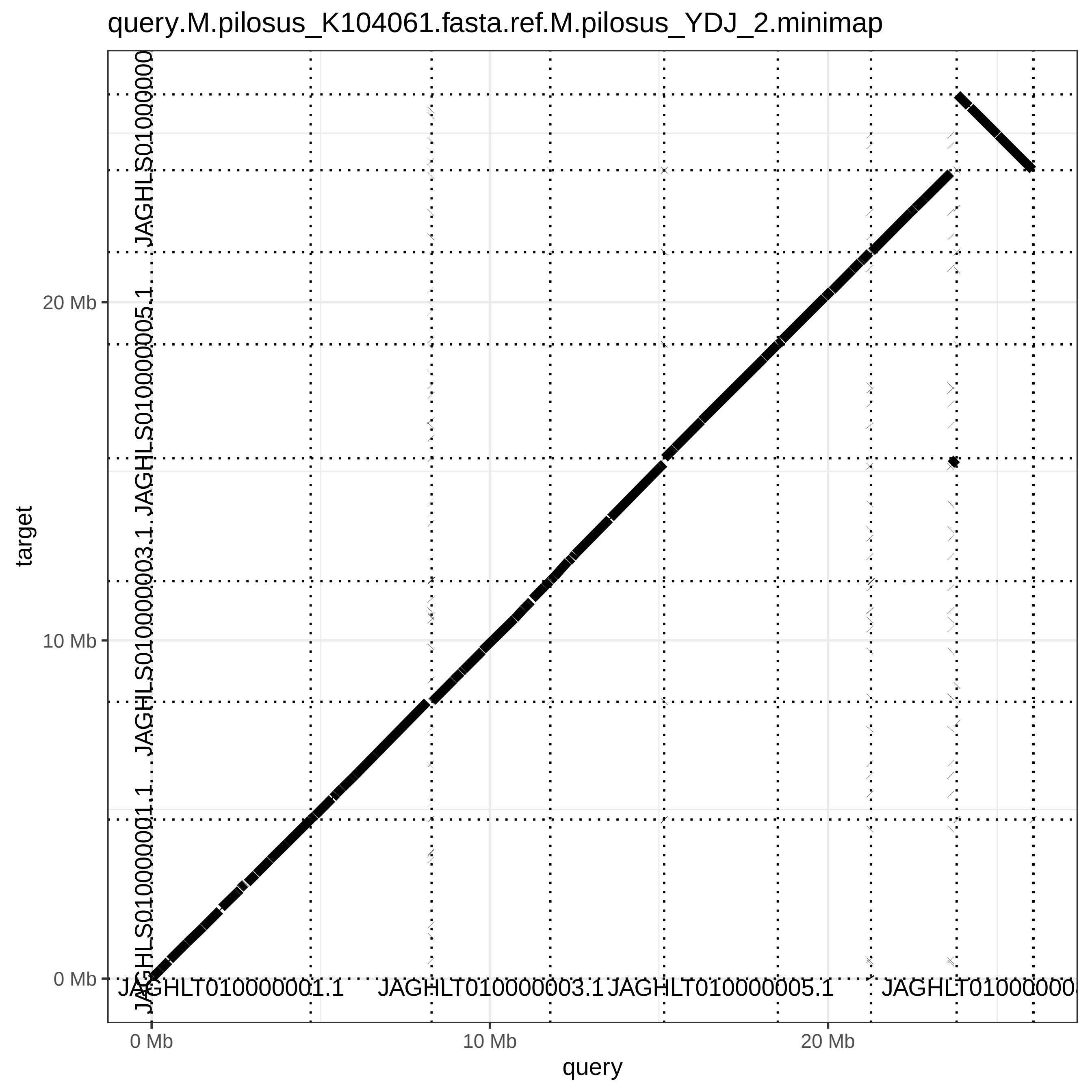

query.M.pilosus_K104061.fasta.ref.M.pilosus_YDJ_2.minimap
target
query
0 Mb
10 Mb
20 Mb
JAGHLS010000001.1
JAGHLS010000003.1
JAGHLS010000005.1
JAGHLT010000001.1
JAGHLT010000003.1
JAGHLT010000005.1

query.M.pilosus_K104061.fasta.ref.M.purpureus_CSU_M183.minimap

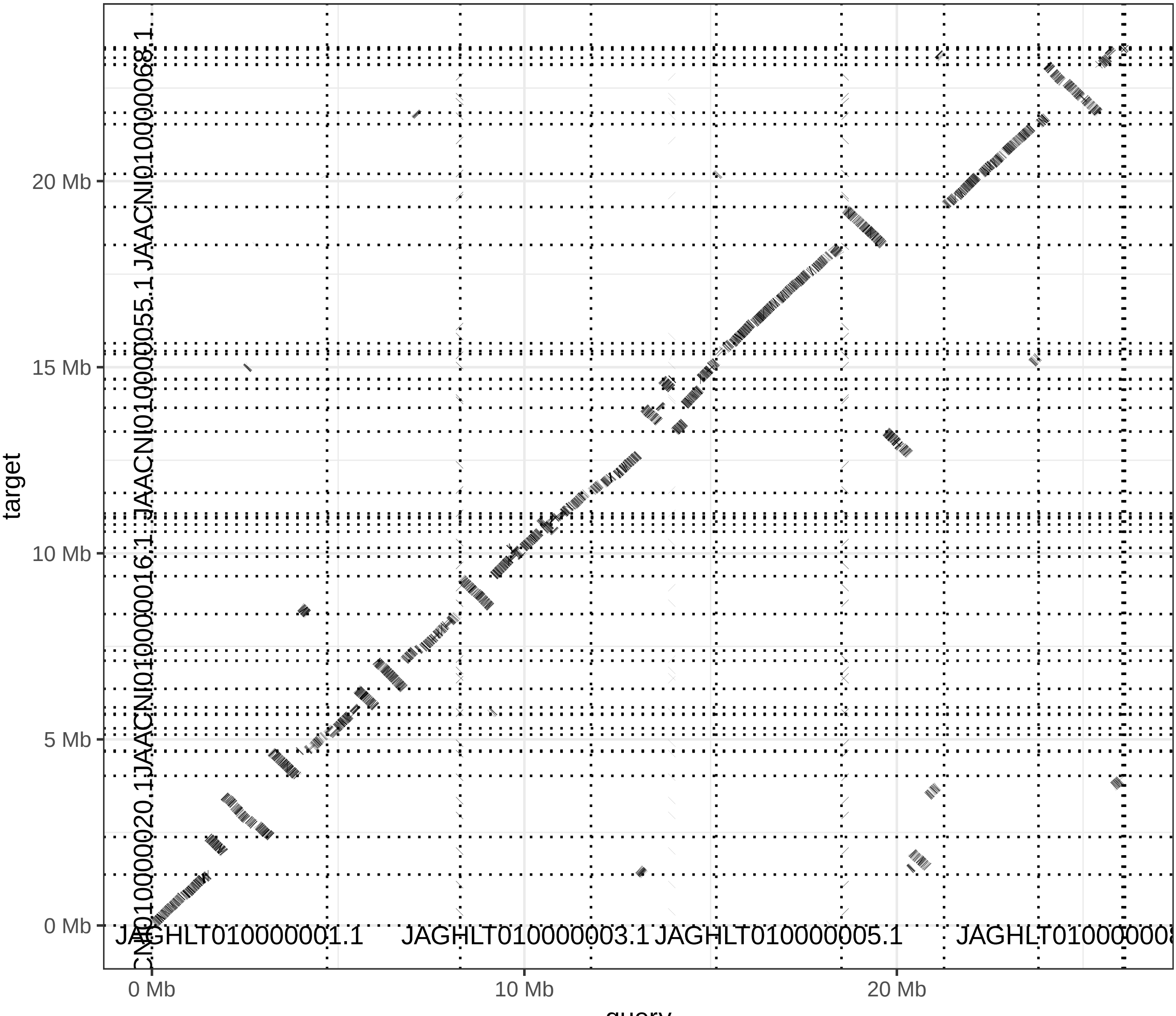

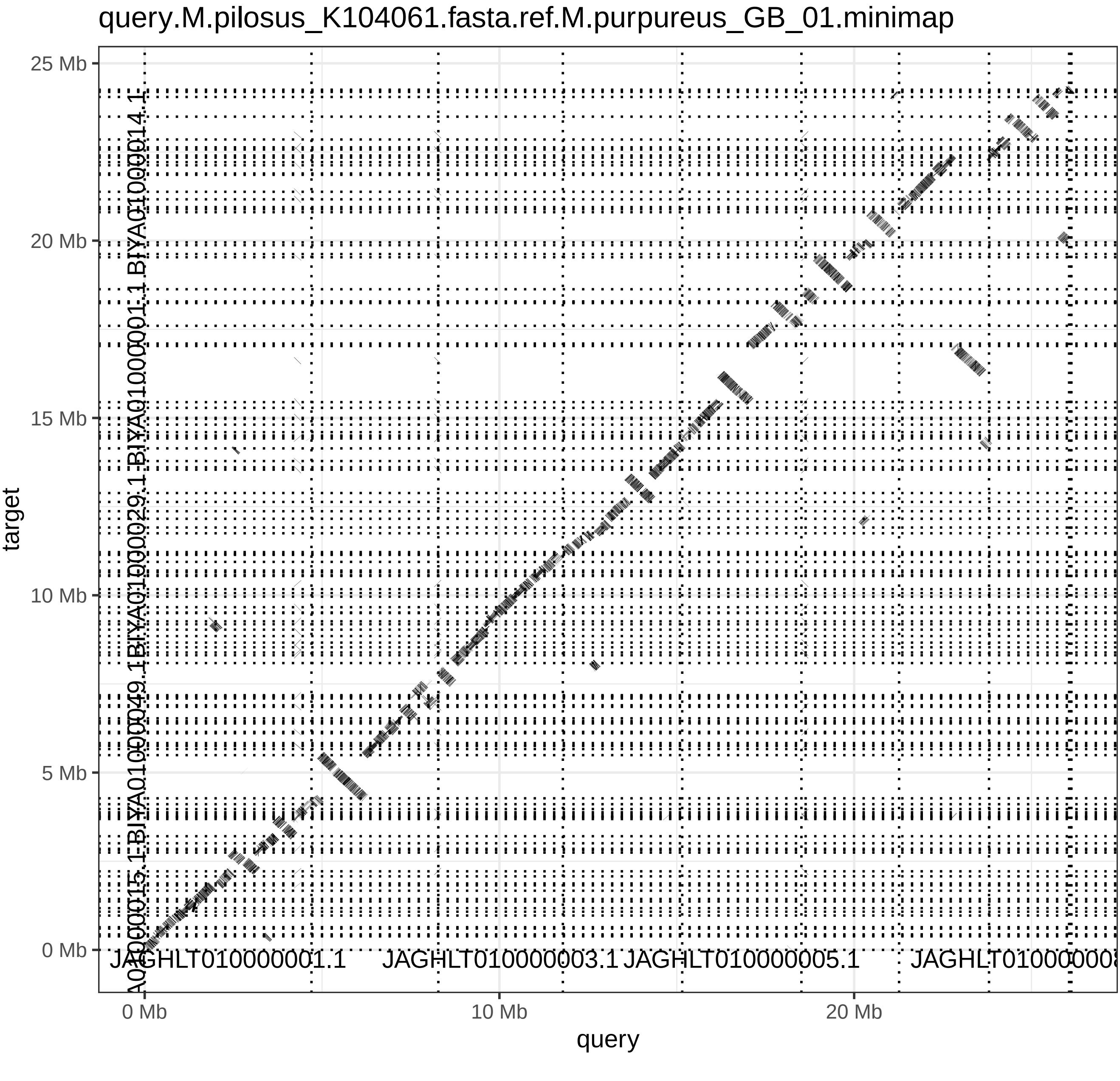

query.M.pilosus_K104061.fasta.ref.M.purpureus_GB_01.minimap
target
25 Mb
20 Mb
15 Mb
10 Mb
5 Mb
0 Mb
query
0 Mb
10 Mb
20 Mb
JAGHLT010000001.1
JAGHLT010000003.1
JAGHLT010000005.1

query.M.pilosus_K104061.fasta.ref.M.purpureus_HQ1.minimap

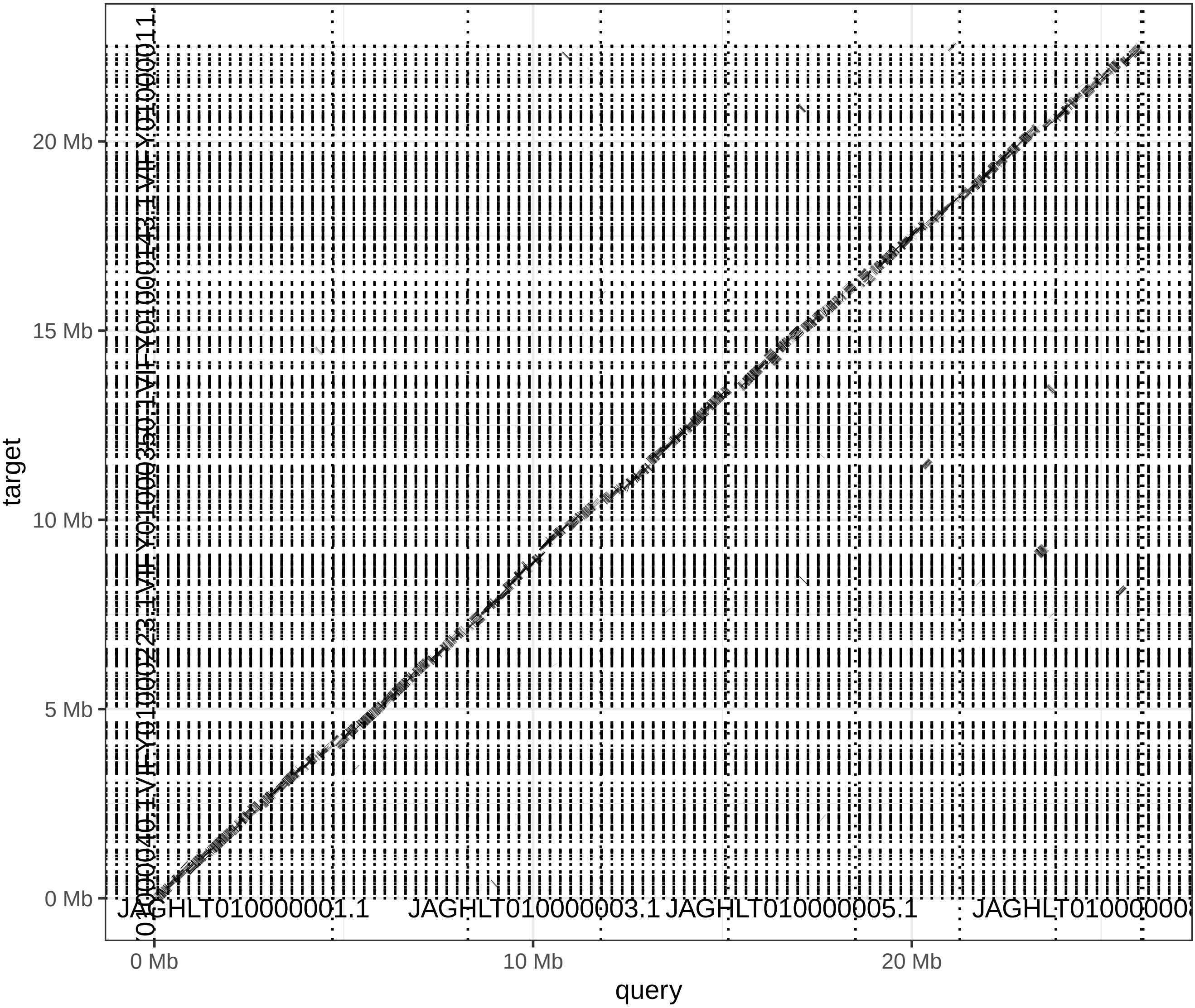

query.M.pilosus_K104061.fasta.ref.M.purpureus_P7048X_2.minimap

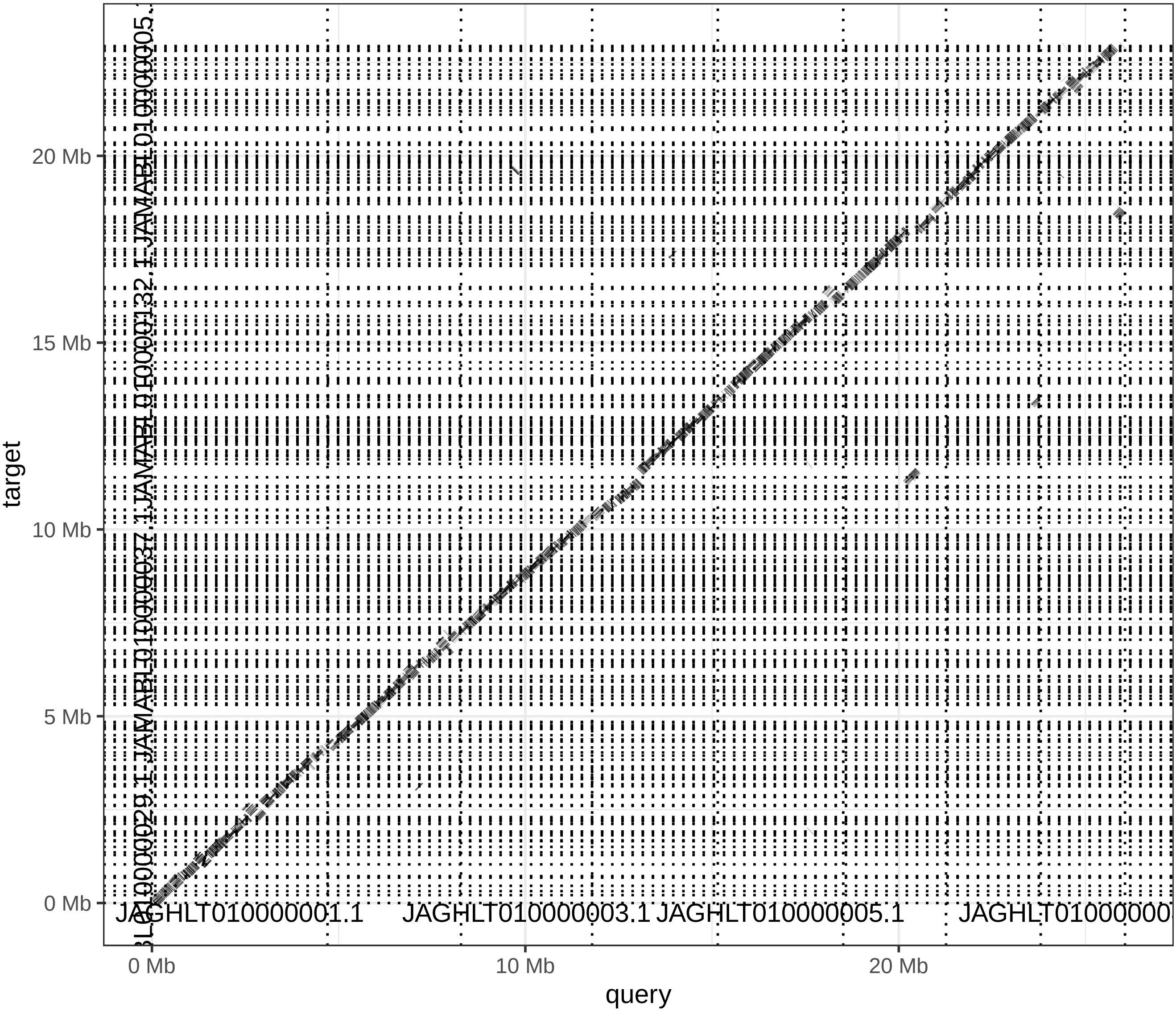

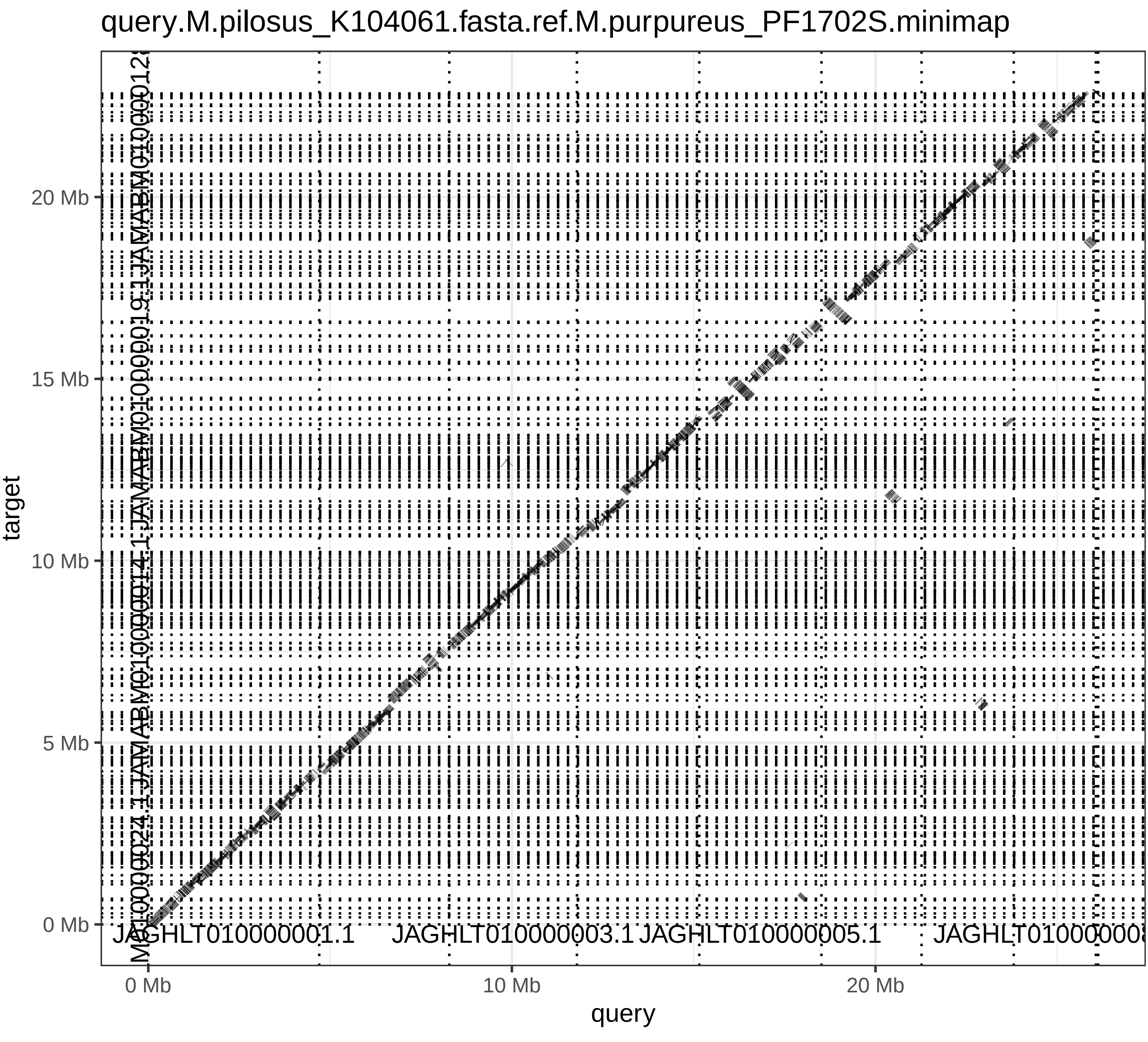

query.M.pilosus_K104061.fasta.ref.M.purpureus_PF1702S.minimap
target
query
0 Mb
5 Mb
10 Mb
15 Mb
20 Mb
0 Mb
10 Mb
20 Mb
JAGHLT010000001.1
JAGHLT010000003.1
JAGHLT010000005.1

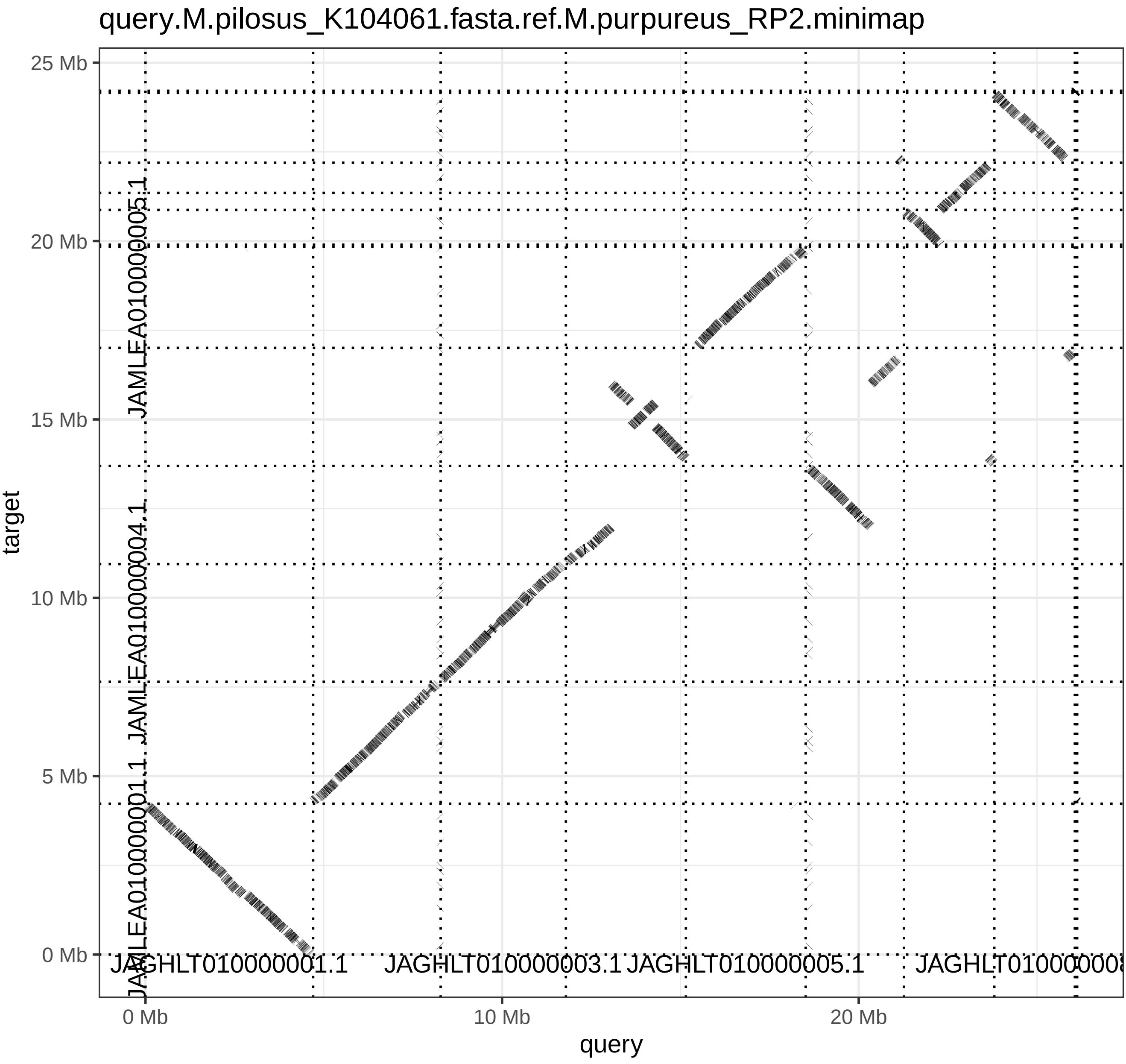

query.M.pilosus_K104061.fasta.ref.M.purpureus_RP2.minimap
target
query
25 Mb
20 Mb
15 Mb
10 Mb
5 Mb
0 Mb
0 Mb
10 Mb
20 Mb
JAMLEA010000005.1
JAMLEA010000004.1
JAMLEA010000001.1
JAGHLT010000001.1
JAGHLT010000003.1
JAGHLT010000005.1

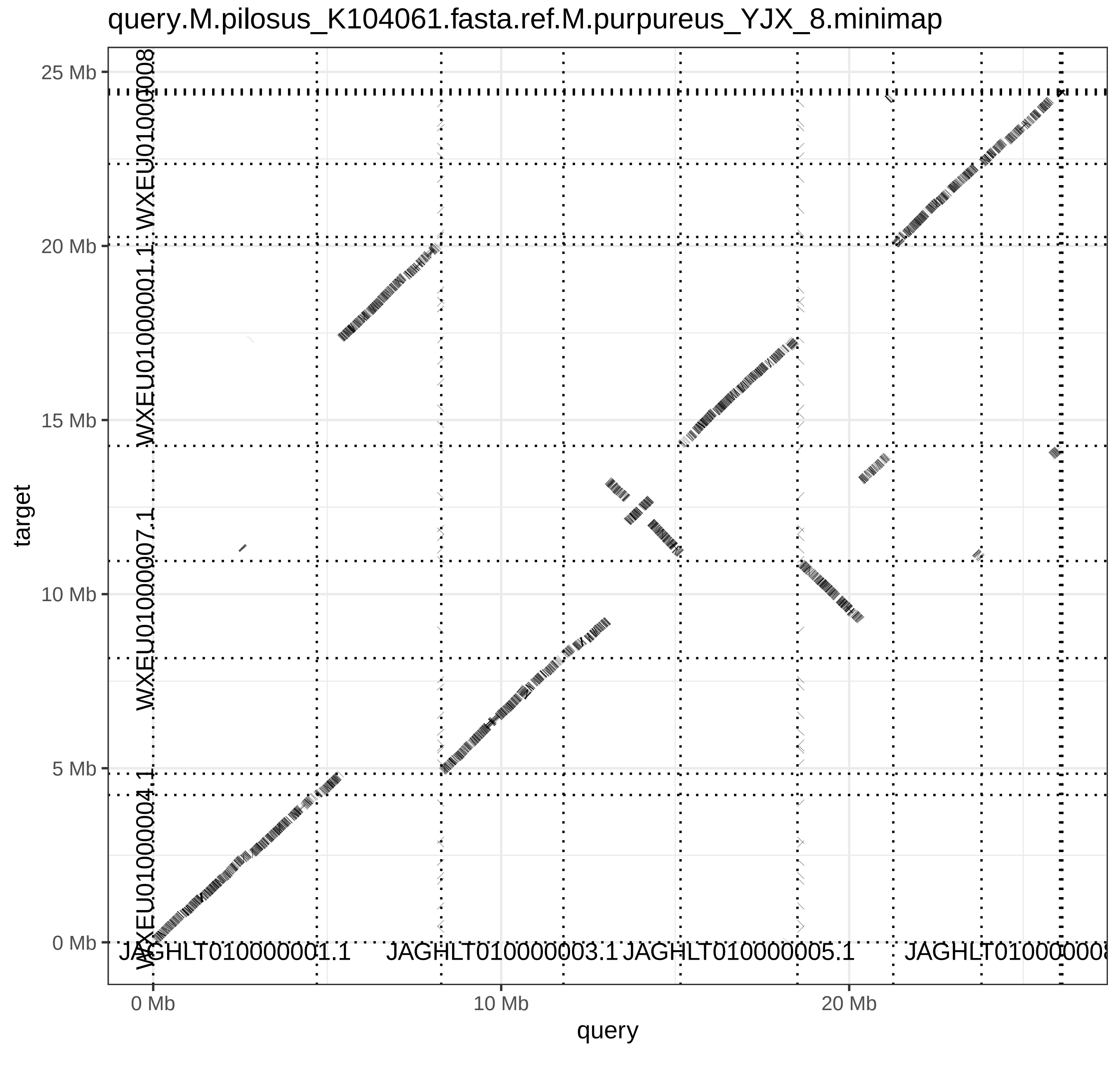

query.M.pilosus_K104061.fasta.ref.M.purpureus_YJX_8.minimap
target
25 Mb
20 Mb
15 Mb
10 Mb
5 Mb
0 Mb
WXEU01000004.1
WXEU01000007.1
WXEU01000001.1
WXEU01000008
JAGHLT010000001.1
JAGHLT010000003.1
JAGHLT010000005.1
JAGHLT01000008
0 Mb
10 Mb
20 Mb
query

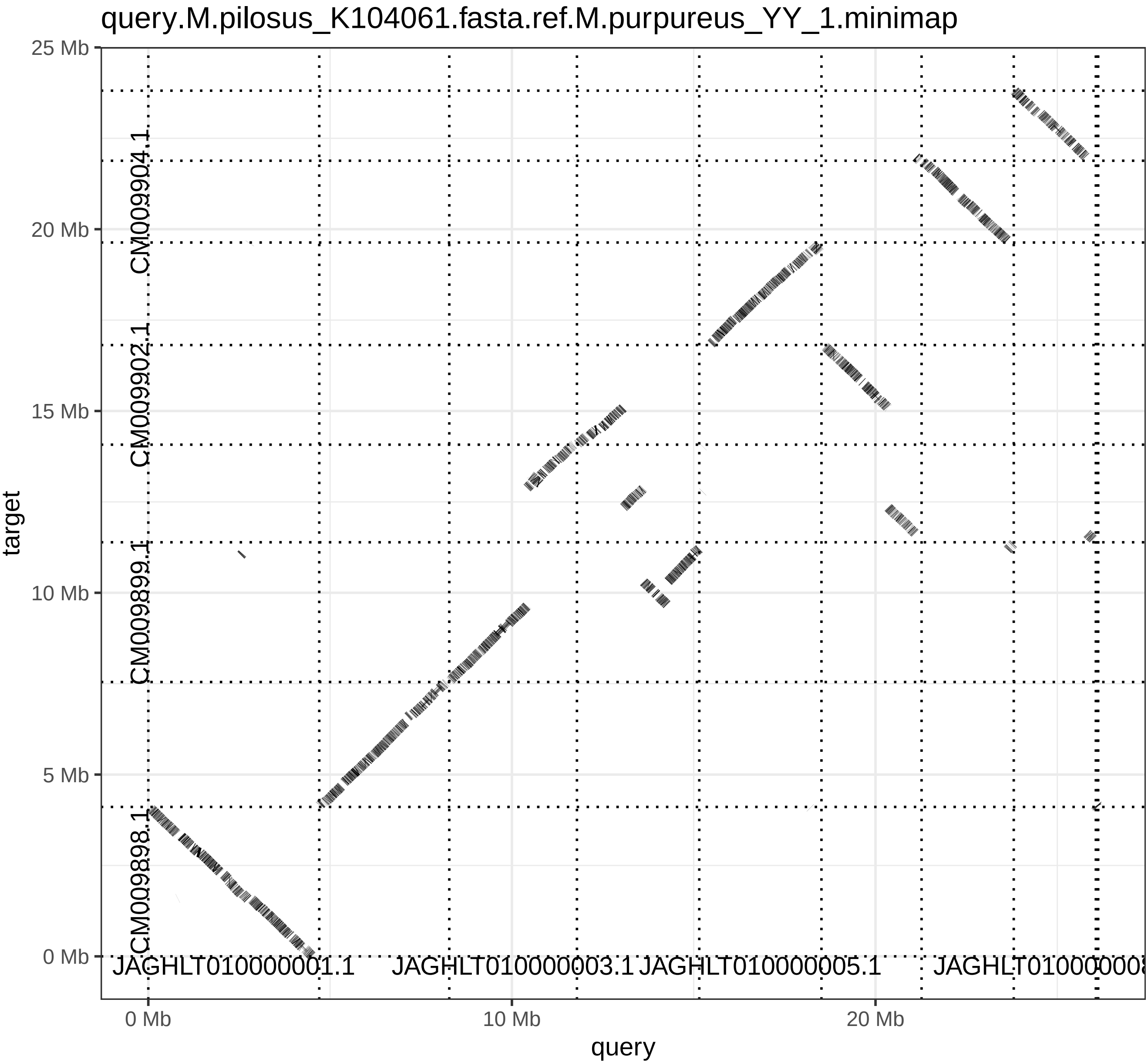
query.M.pilosus_K104061.fasta.ref.M.purpureus_YY_1.minimap
target
query
25 Mb
20 Mb
15 Mb
10 Mb
5 Mb
0 Mb
0 Mb
10 Mb
20 Mb
CM009904.1
CM009902.1
CM009899.1
CM009898.1
JAGHLT010000001.1
JAGHLT010000003.1
JAGHLT010000005.1

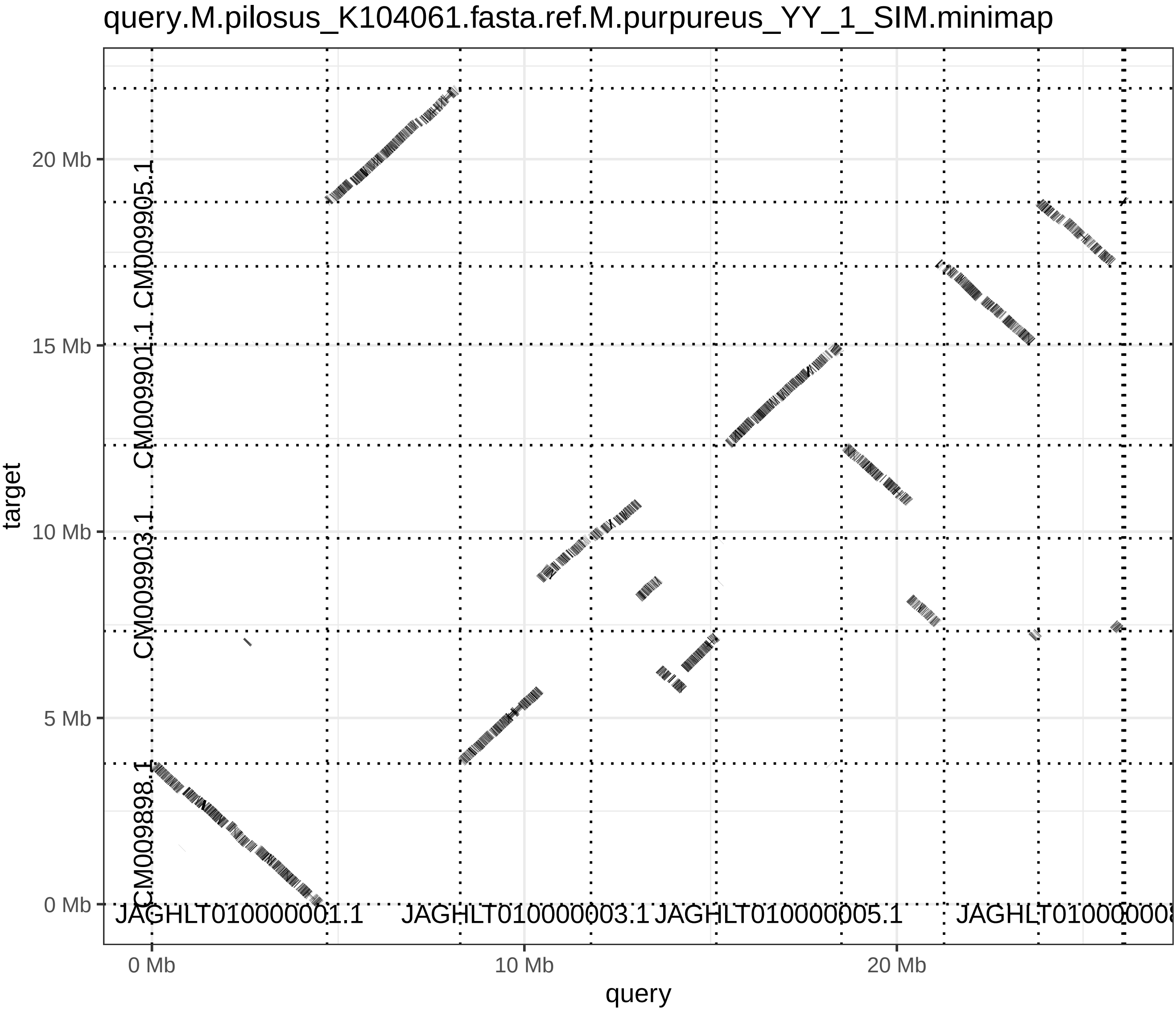

query.M.pilosus_K104061.fasta.ref.M.purpureus_YY_1_SIM.minimap
target
20 Mb
15 Mb
10 Mb
5 Mb
0 Mb
CM009905.1
CM009901.1
CM009903.1
CM009898.1
JAGHLT010000001.1
JAGHLT010000003.1
JAGHLT010000005.1
0 Mb
10 Mb
20 Mb
query

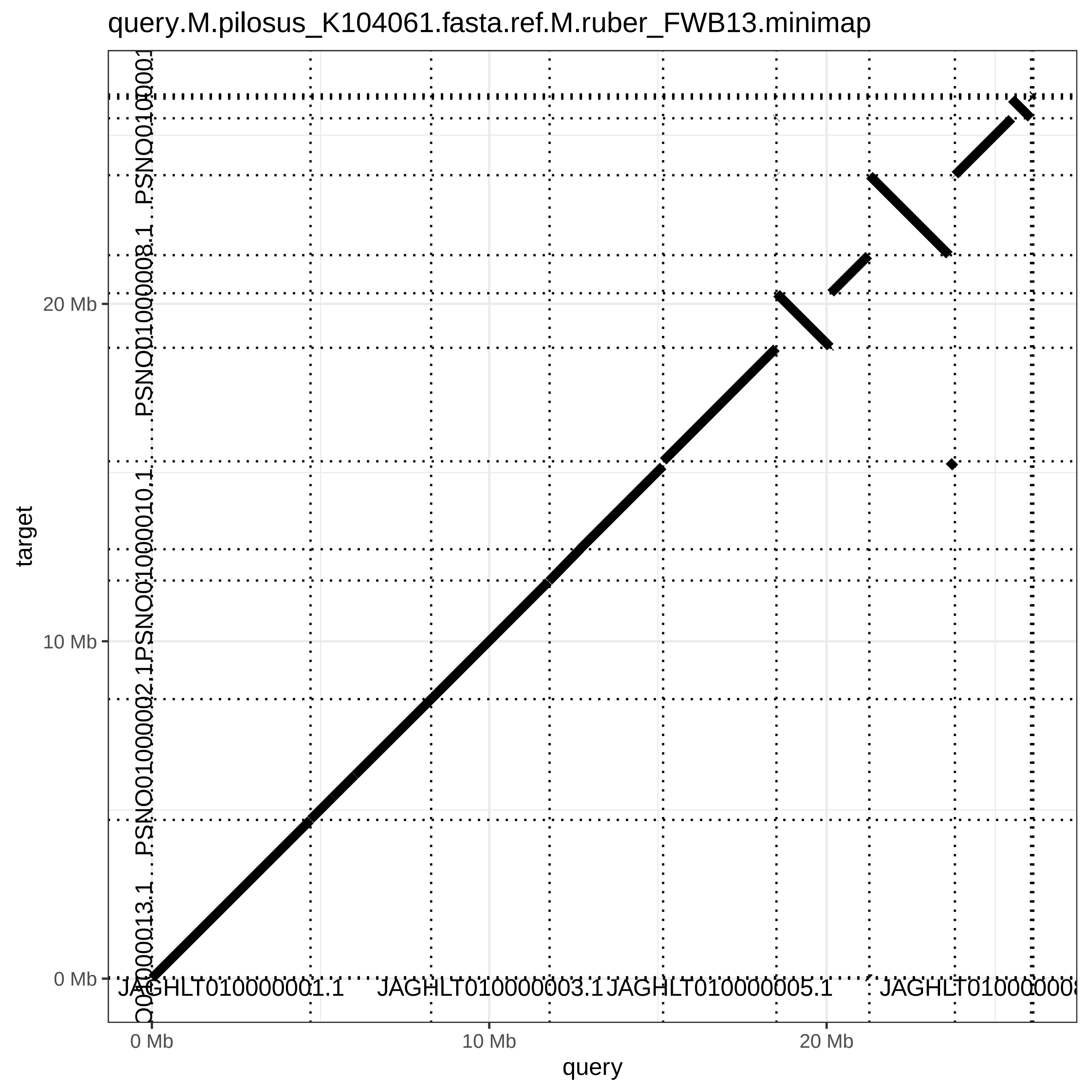

query.M.pilosus_K104061.fasta.ref.M.ruber_FWB13.minimap
target
query
0 Mb
10 Mb
20 Mb
JAGHLT010000001.1
JAGHLT010000003.1
JAGHLT010000005.1
PSNO010000013.1
PSNO010000002.1
PSNO010000010.1
PSNO010000008.1

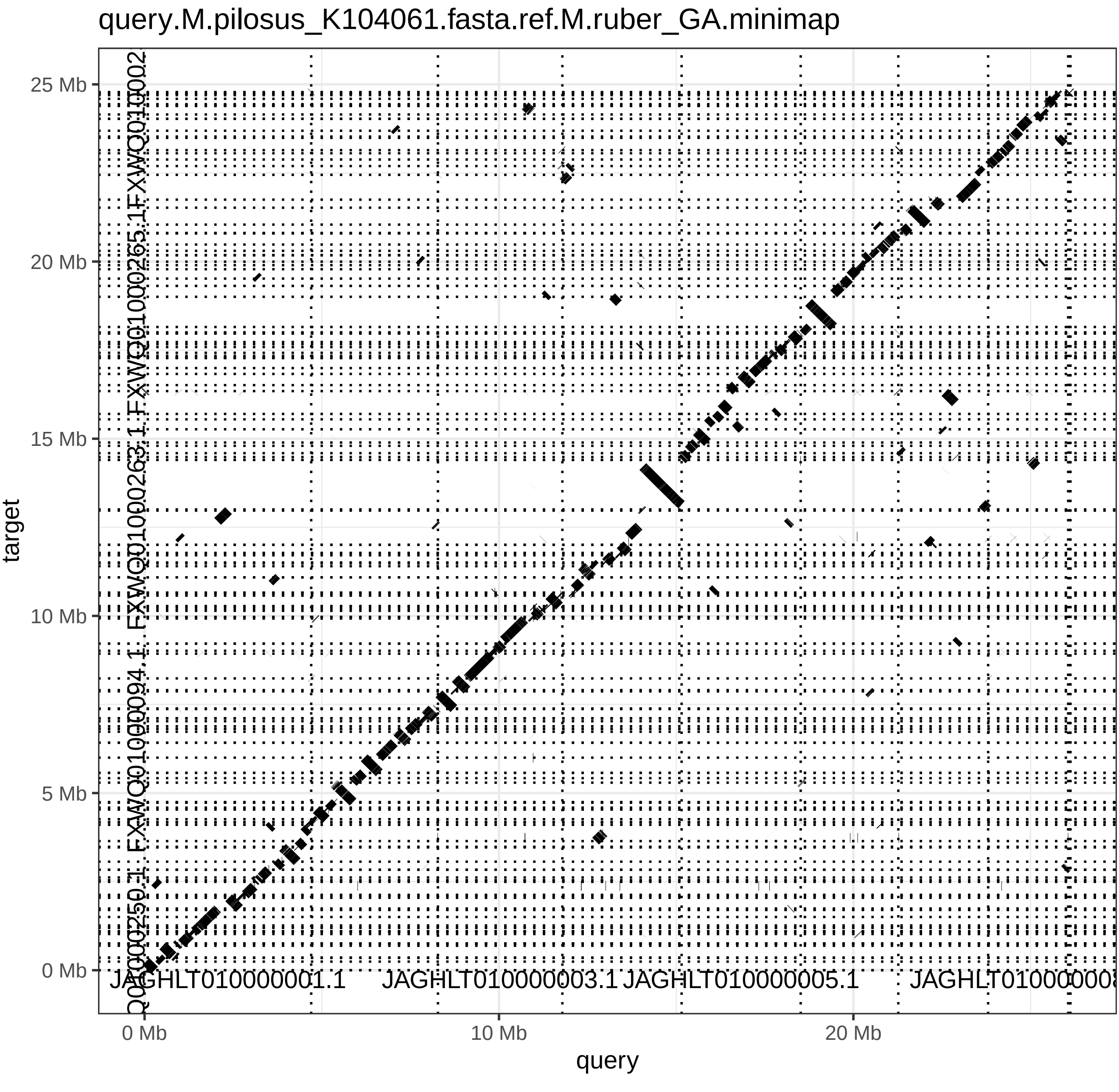
query.M.pilosus_K104061.fasta.ref.M.ruber_GA.minimap
target
query
25 Mb
20 Mb
15 Mb
10 Mb
5 Mb
0 Mb
0 Mb
10 Mb
20 Mb
JAGHLT010000001.1
JAGHLT010000003.1
JAGHLT010000005.1
FXWQ01000094.1
FXWQ010000263.1
FXWQ010000265.1

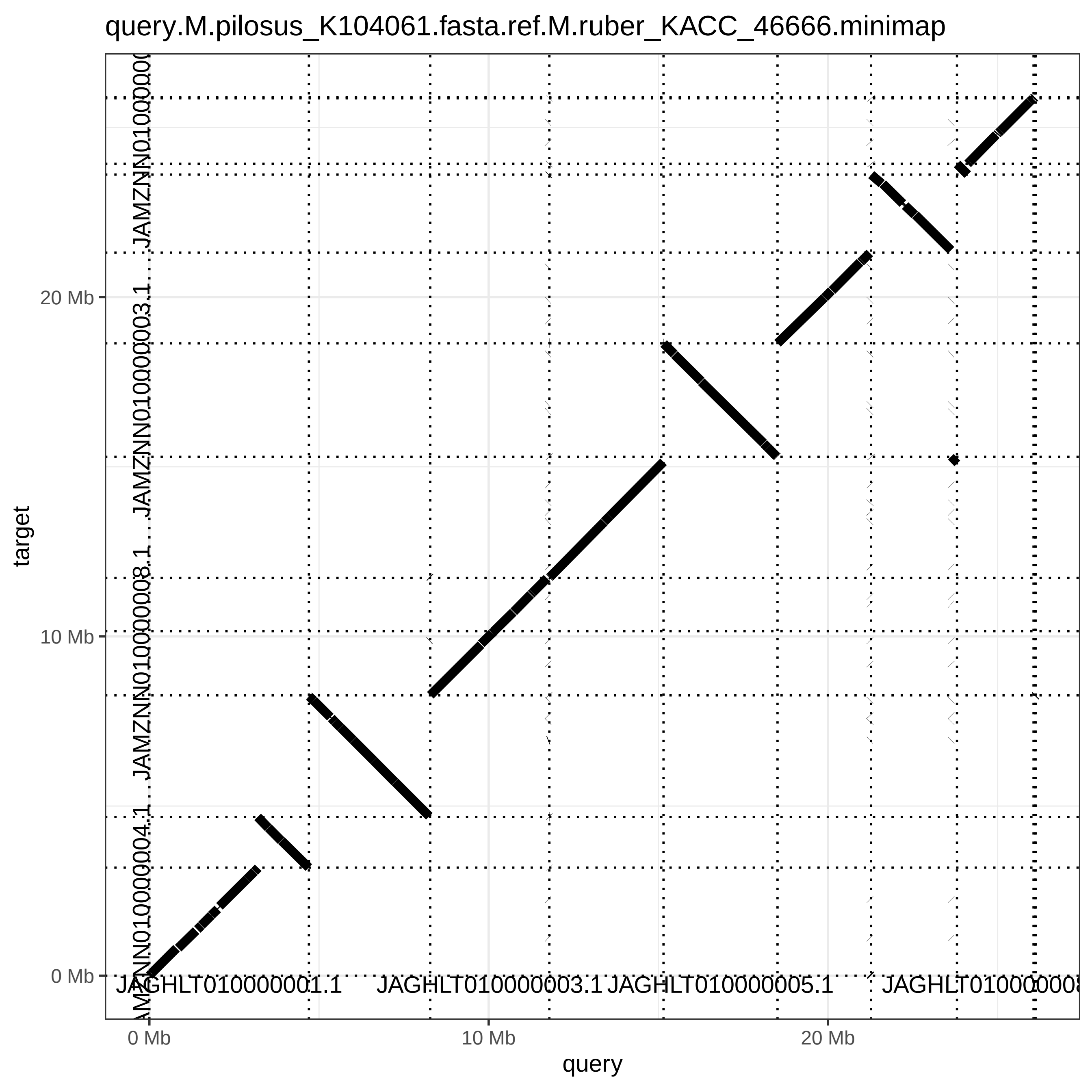
query.M.pilosus_K104061.fasta.ref.M.ruber_KACC_46666.minimap
target
query
0 Mb
10 Mb
20 Mb
JAGHLT010000001.1
JAGHLT010000003.1
JAGHLT010000005.1
JAGHLT01000008
JAMZNN010000004.1
JAMZNN010000008.1
JAMZNN010000003.1
JAMZNN0100000

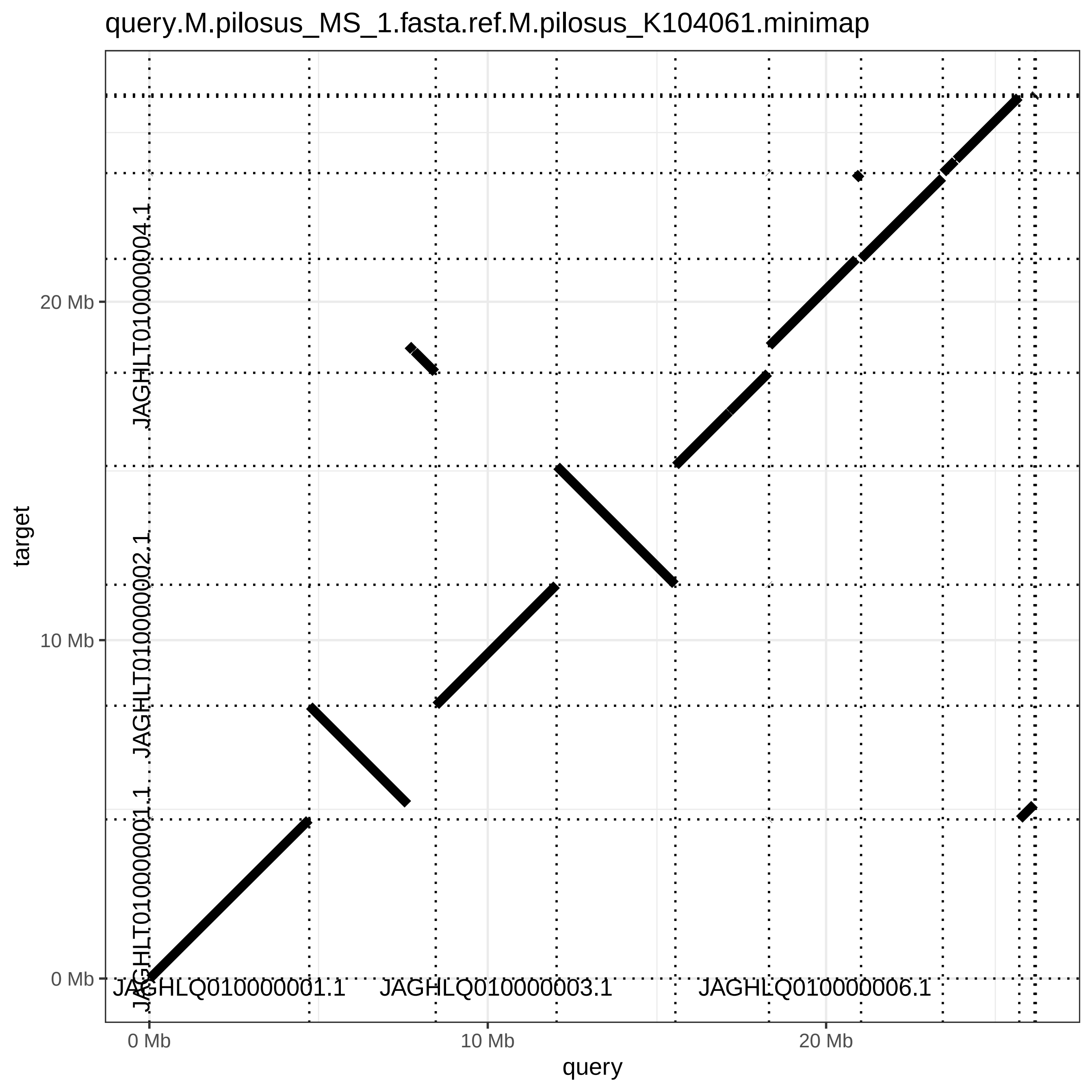
query.M.pilosus_MS_1.fasta.ref.M.pilosus_K104061.minimap
target
query
JAGHLT010000001.1
JAGHLT010000002.1
JAGHLT010000004.1
JAGHLQ010000001.1
JAGHLQ010000003.1
JAGHLQ010000006.1
0 Mb
10 Mb
20 Mb
0 Mb
10 Mb
20 Mb

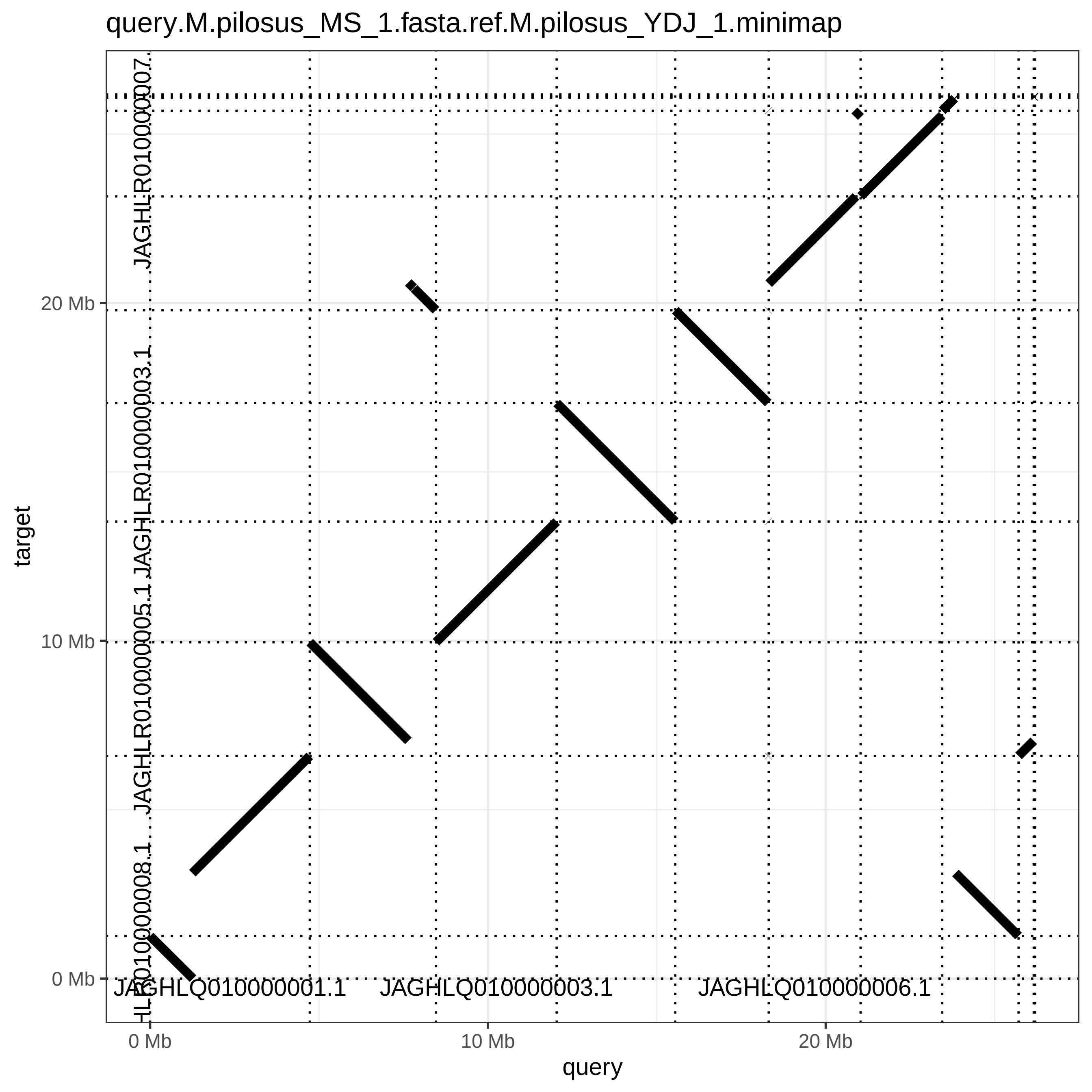

query.M.pilosus_MS_1.fasta.ref.M.pilosus_YDJ_1.minimap
target
query
0 Mb
10 Mb
20 Mb
JAGHLQ010000001.1
JAGHLQ010000003.1
JAGHLQ010000006.1
JAGHLR010000008.1
JAGHLR010000005.1
JAGHLR010000003.1
JAGHLR010000007.

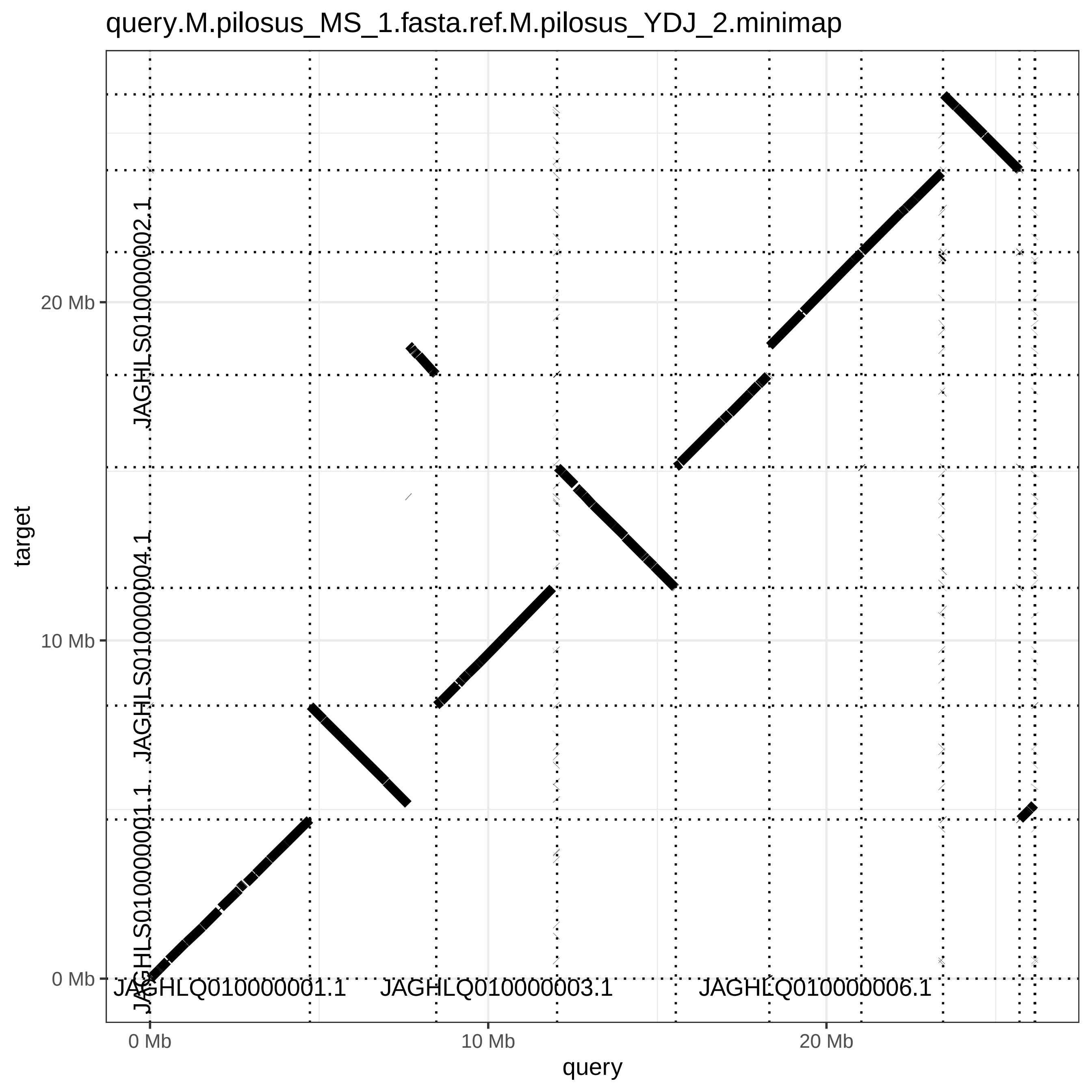

query.M.pilosus_MS_1.fasta.ref.M.pilosus_YDJ_2.minimap
target
20 Mb
10 Mb
0 Mb
JAGHLS010000002.1
JAGHLS010000004.1
JAGHLS010000001.1
JAGHLQ010000001.1
JAGHLQ010000003.1
JAGHLQ010000006.1
0 Mb
10 Mb
20 Mb
query

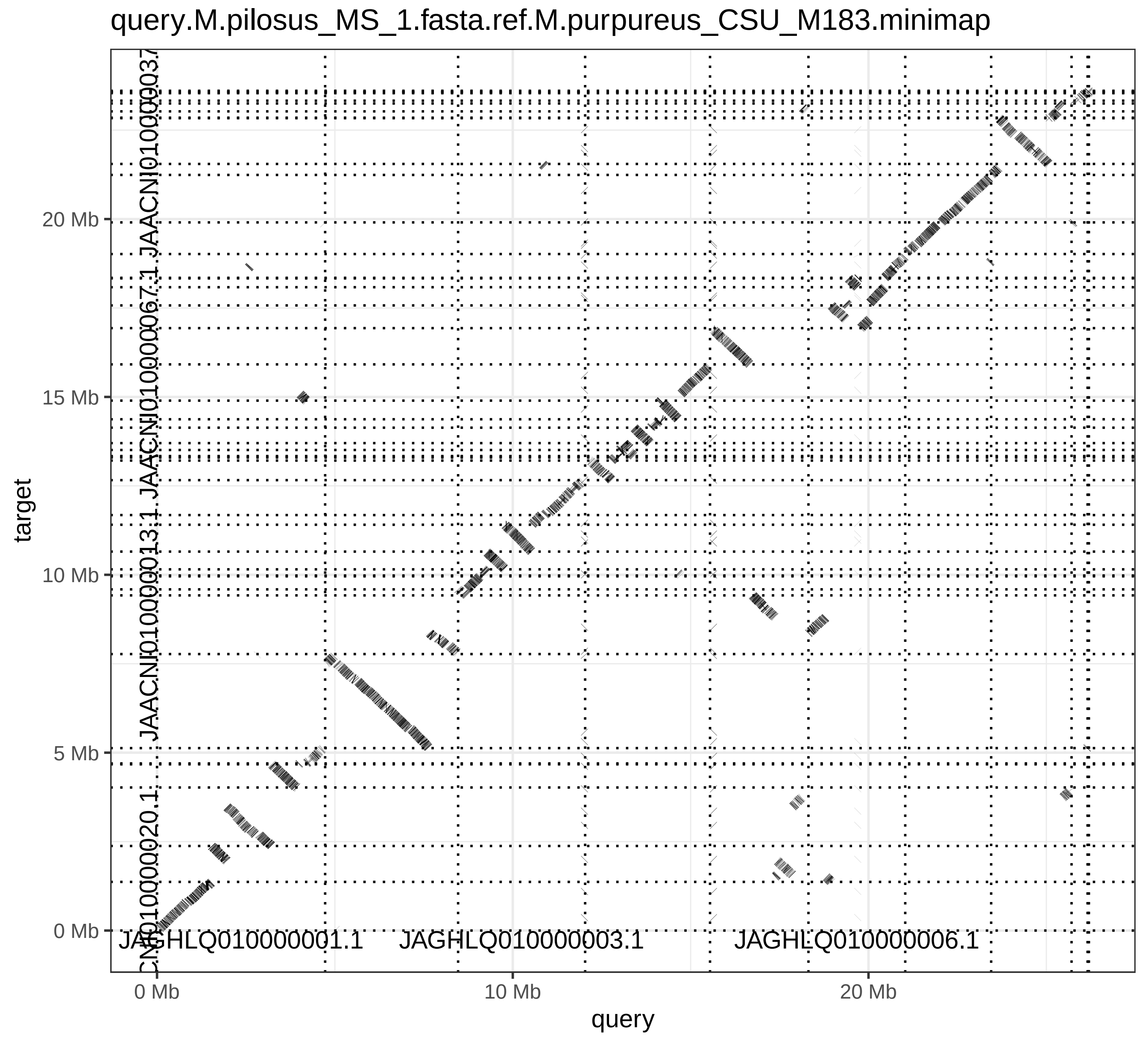

query.M.pilosus_MS_1.fasta.ref.M.purpureus_CSU_M183.minimap
target
query
20 Mb
15 Mb
10 Mb
5 Mb
0 Mb
0 Mb
10 Mb
20 Mb
JAGHLQ010000001.1
JAGHLQ010000003.1
JAGHLQ010000006.1

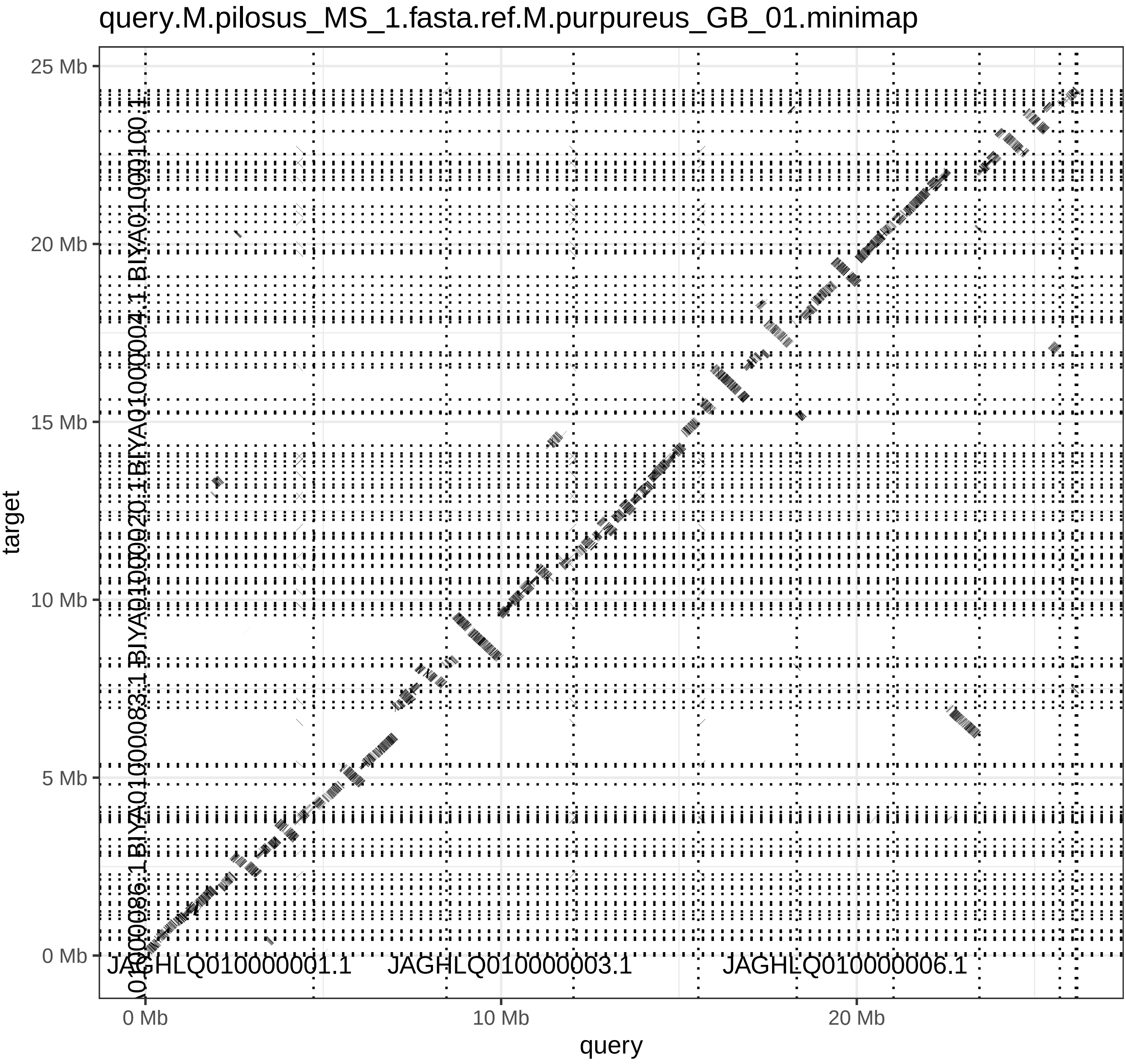
query.M.pilosus_MS_1.fasta.ref.M.purpureus_GB_01.minimap
target
query
25 Mb
20 Mb
15 Mb
10 Mb
5 Mb
0 Mb
0 Mb
10 Mb
20 Mb
JAGHLQ010000001.1
JAGHLQ010000003.1
JAGHLQ010000006.1

query.M.pilosus_MS_1.fasta.ref.M.purpureus_HQ1.minimap

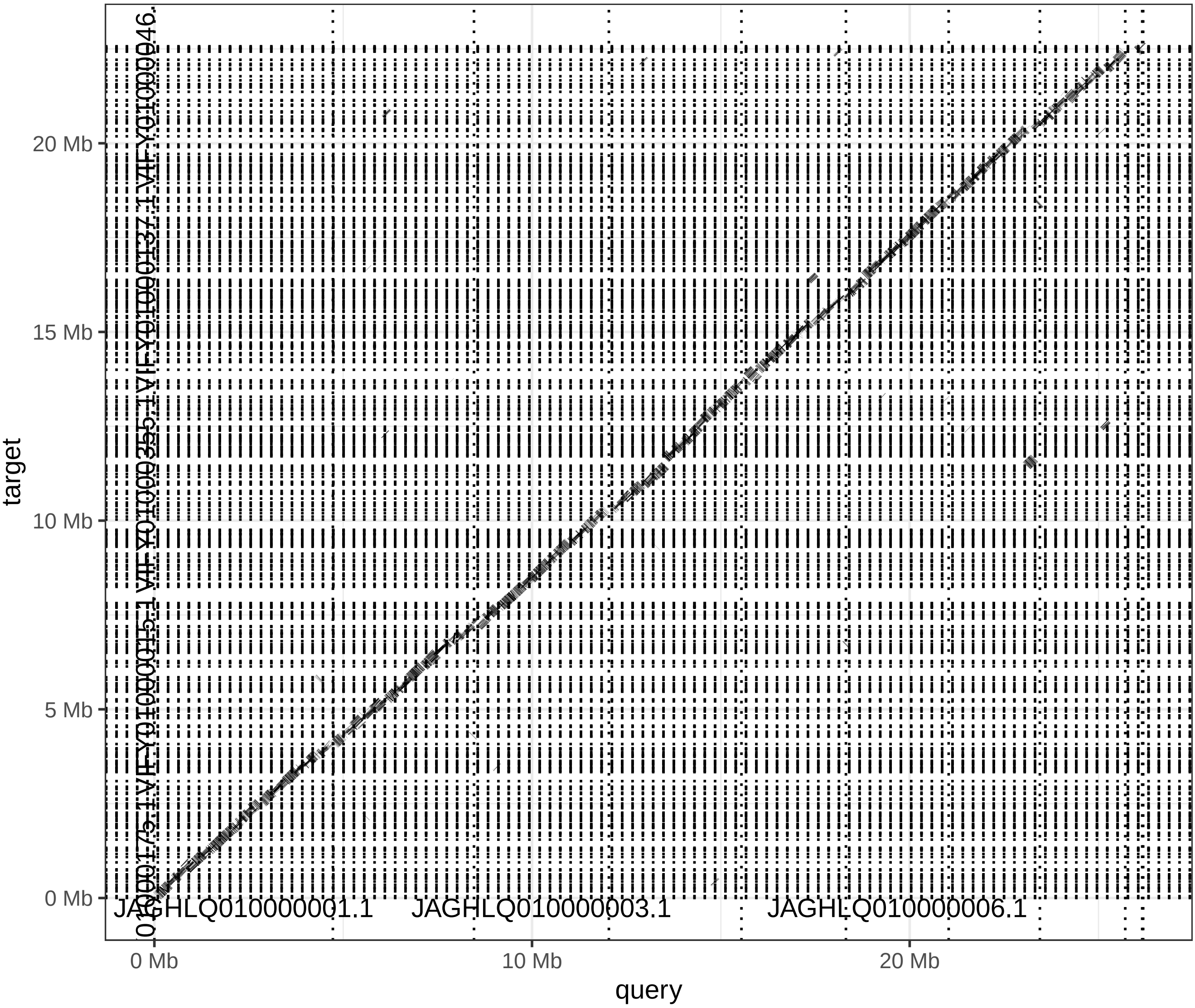

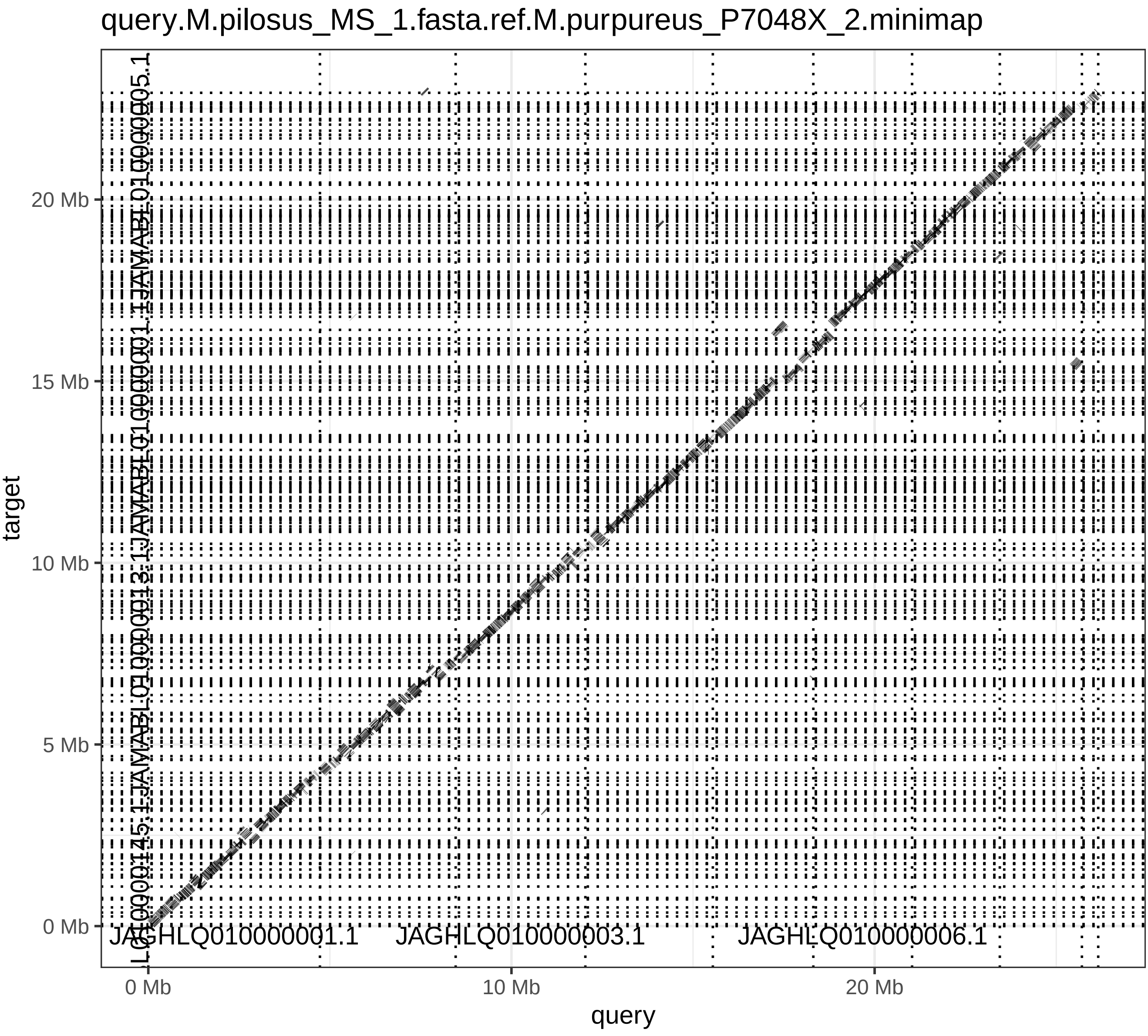
query.M.pilosus_MS_1.fasta.ref.M.purpureus_P7048X_2.minimap
target
20 Mb
15 Mb
10 Mb
5 Mb
0 Mb
JAGHLQ010000001.1
JAGHLQ010000003.1
JAGHLQ010000006.1
0 Mb
10 Mb
20 Mb
query

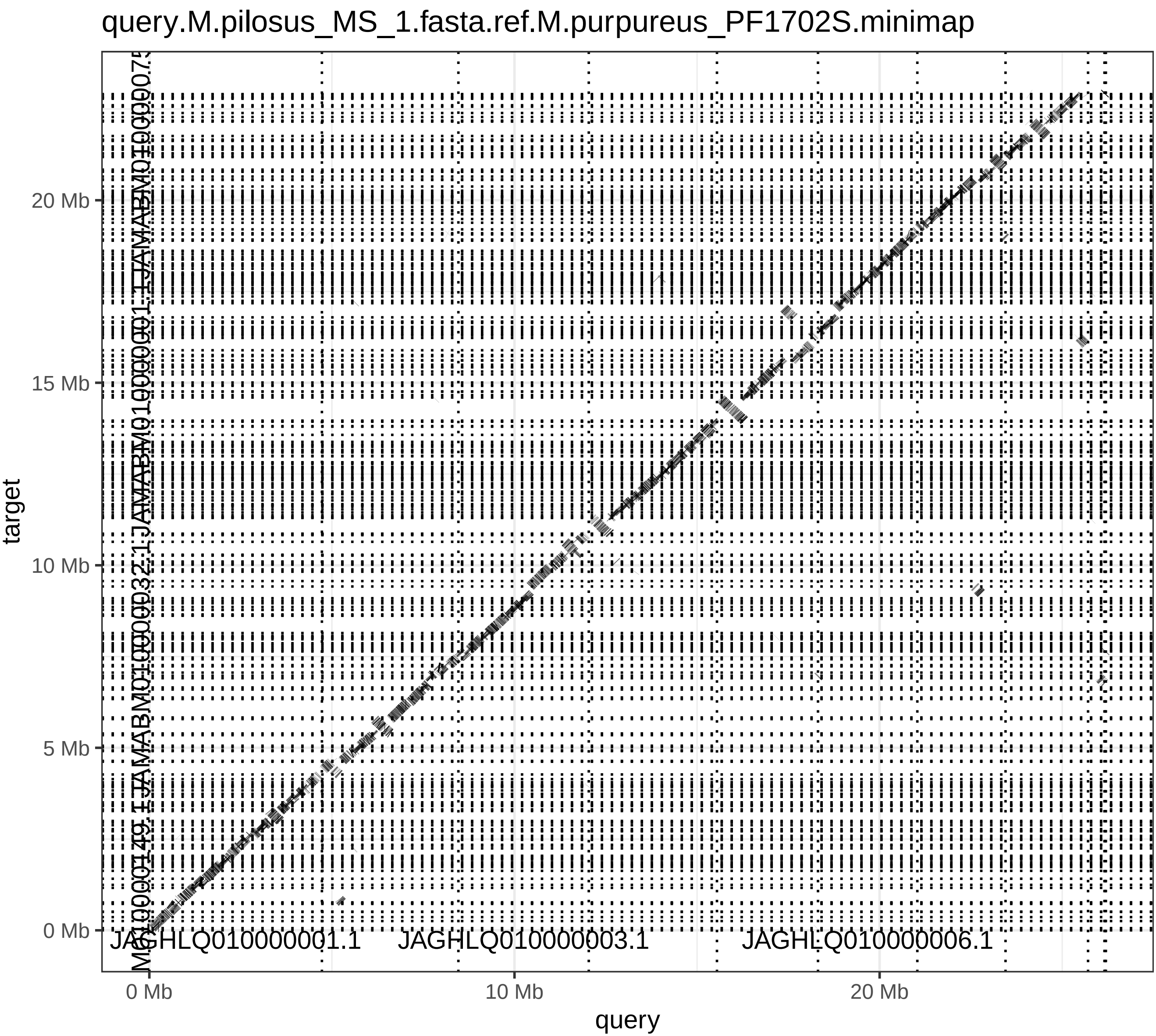
query.M.pilosus_MS_1.fasta.ref.M.purpureus_PF1702S.minimap
target
query
0 Mb
5 Mb
10 Mb
15 Mb
20 Mb
0 Mb
10 Mb
20 Mb
JAGHLQ010000001.1
JAGHLQ010000003.1
JAGHLQ010000006.1

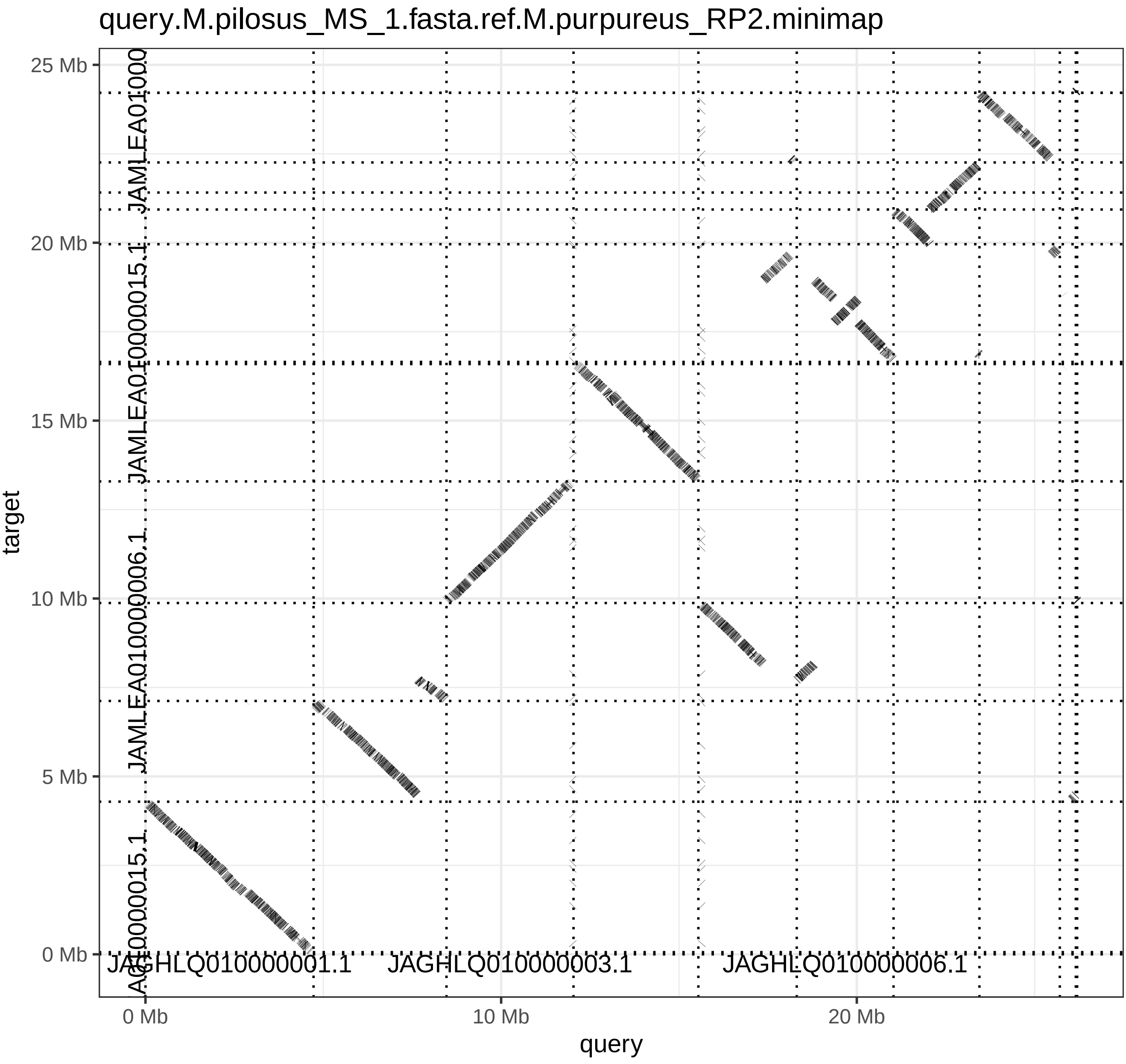

query.M.pilosus_MS_1.fasta.ref.M.purpureus_RP2.minimap
target
25 Mb
20 Mb
15 Mb
10 Mb
5 Mb
0 Mb
JAMLEA01000
JAMLEA010000015.1
JAMLEA010000006.1
JAMLEA010000015.1
JAGHLQ010000001.1
JAGHLQ010000003.1
JAGHLQ010000006.1
0 Mb
10 Mb
20 Mb
query

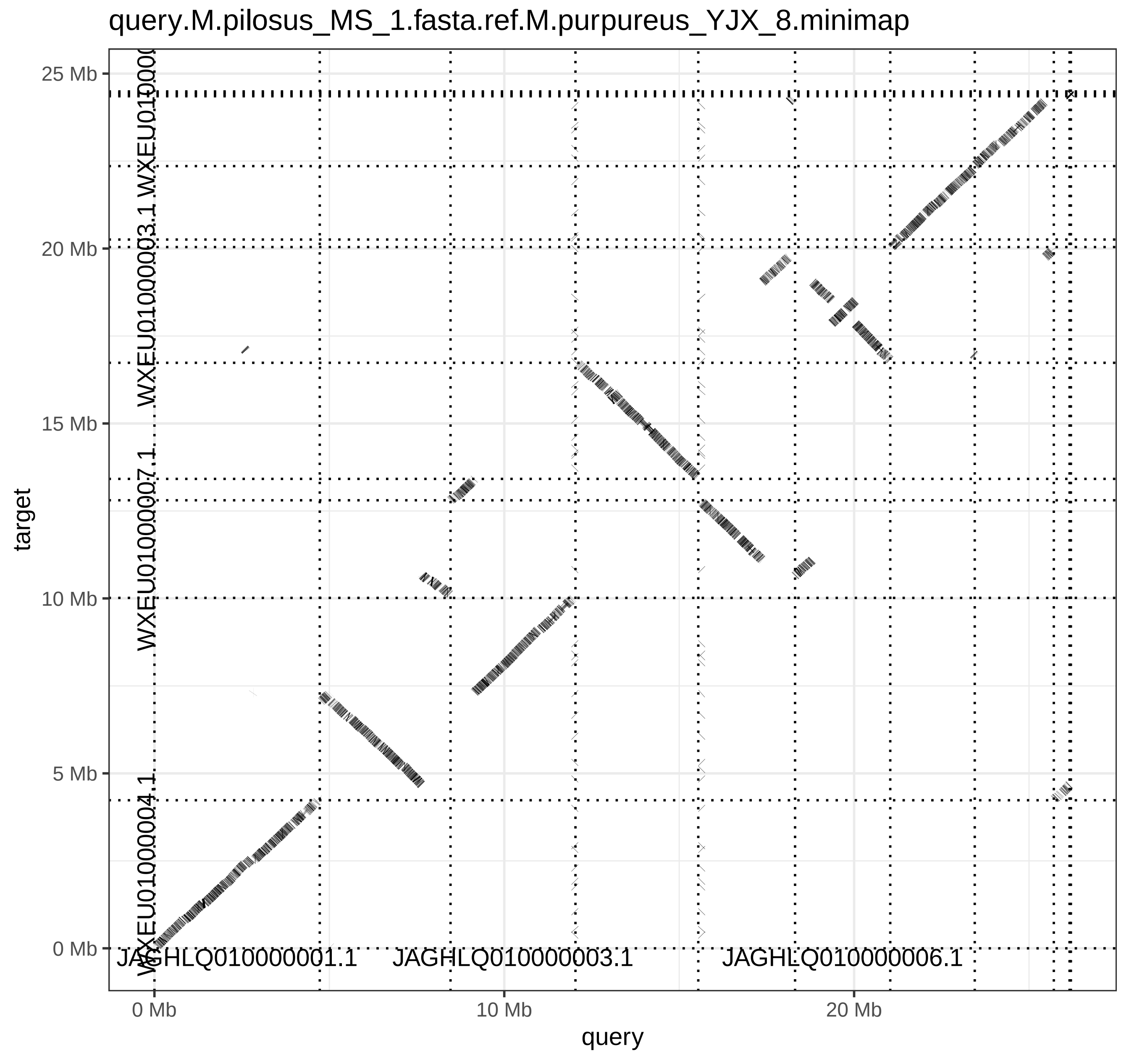

query.M.pilosus_MS_1.fasta.ref.M.purpureus_YJX_8.minimap
target
query
25 Mb
20 Mb
15 Mb
10 Mb
5 Mb
0 Mb
0 Mb
10 Mb
20 Mb
WXEU010000004.1
WXEU010000007.1
WXEU010000003.1
JAGHLQ010000001.1
JAGHLQ010000003.1
JAGHLQ010000006.1

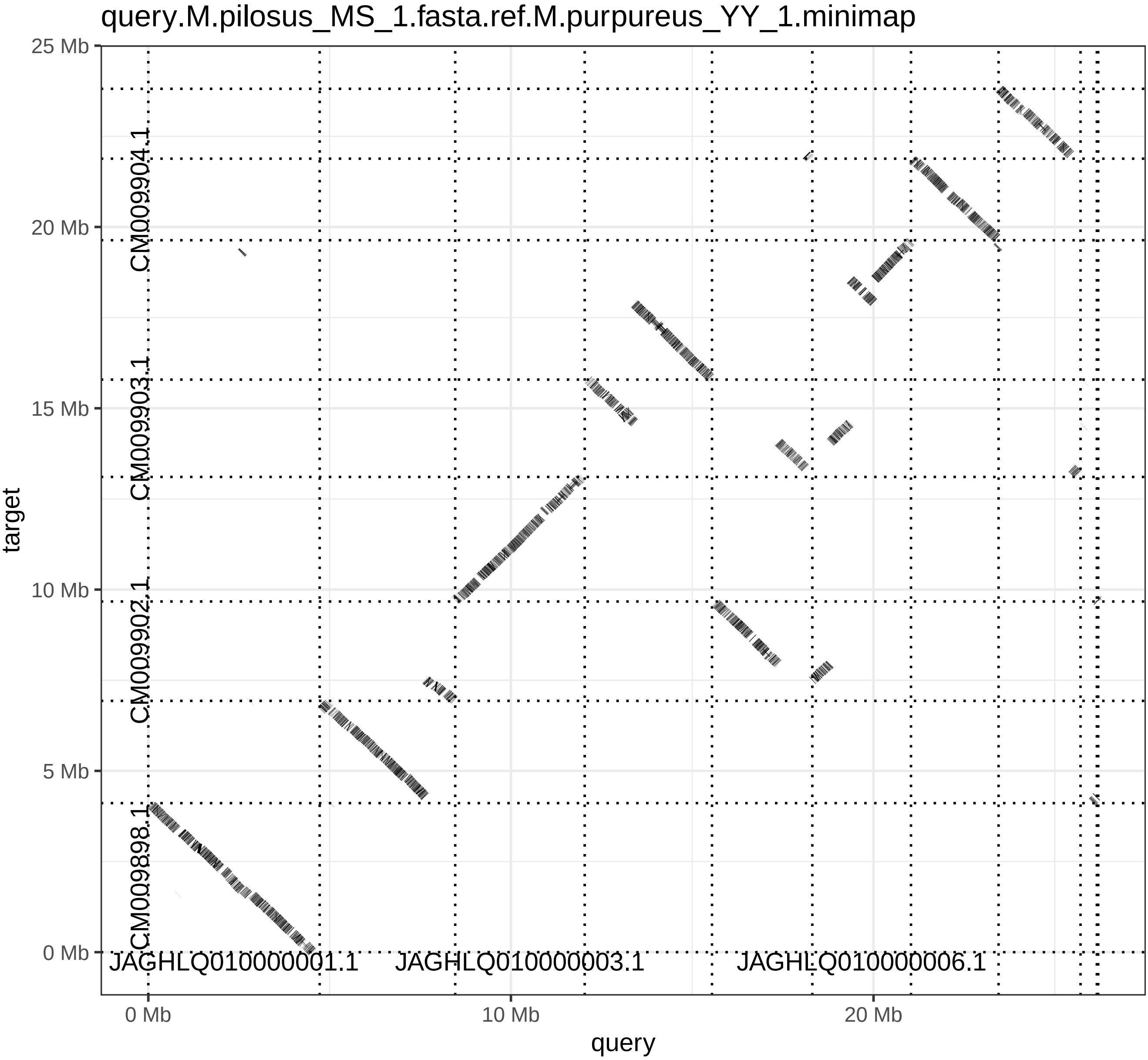

query.M.pilosus_MS_1.fasta.ref.M.purpureus_YY_1.minimap
25 Mb
20 Mb
15 Mb
10 Mb
5 Mb
0 Mb
target
CM009904.1
CM009903.1
CM009902.1
CM009898.1
JAGHLQ010000001.1
JAGHLQ010000003.1
JAGHLQ010000006.1
0 Mb
10 Mb
20 Mb
query

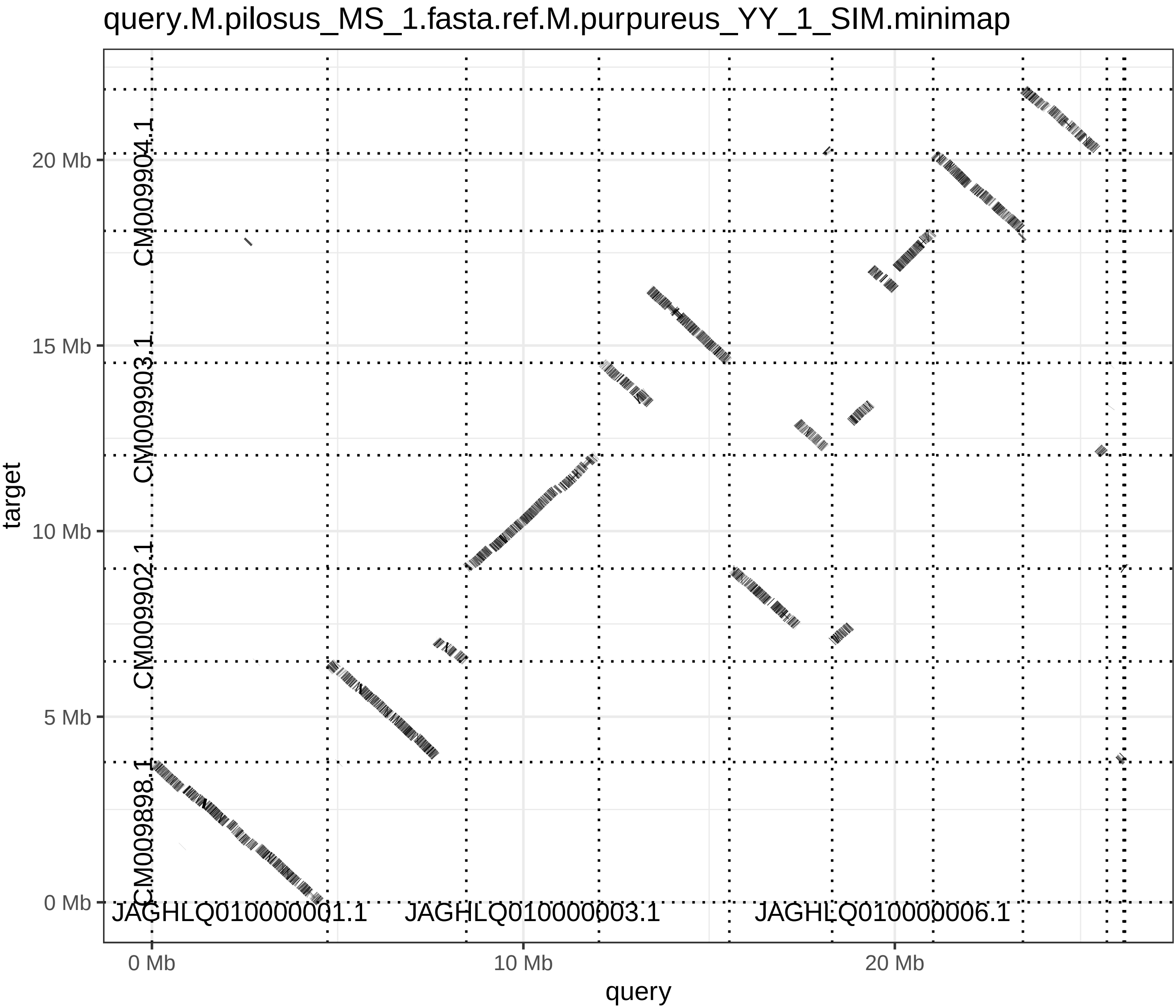
query.M.pilosus_MS_1.fasta.ref.M.purpureus_YY_1_SIM.minimap
target
query
CM009904.1
CM009903.1
CM009902.1
CM009898.1
JAGHLQ010000001.1
JAGHLQ010000003.1
JAGHLQ010000006.1
20 Mb
15 Mb
10 Mb
5 Mb
0 Mb
0 Mb
10 Mb
20 Mb

query.M.pilosus_MS_1.fasta.ref.M.ruber_FWB13.minimap

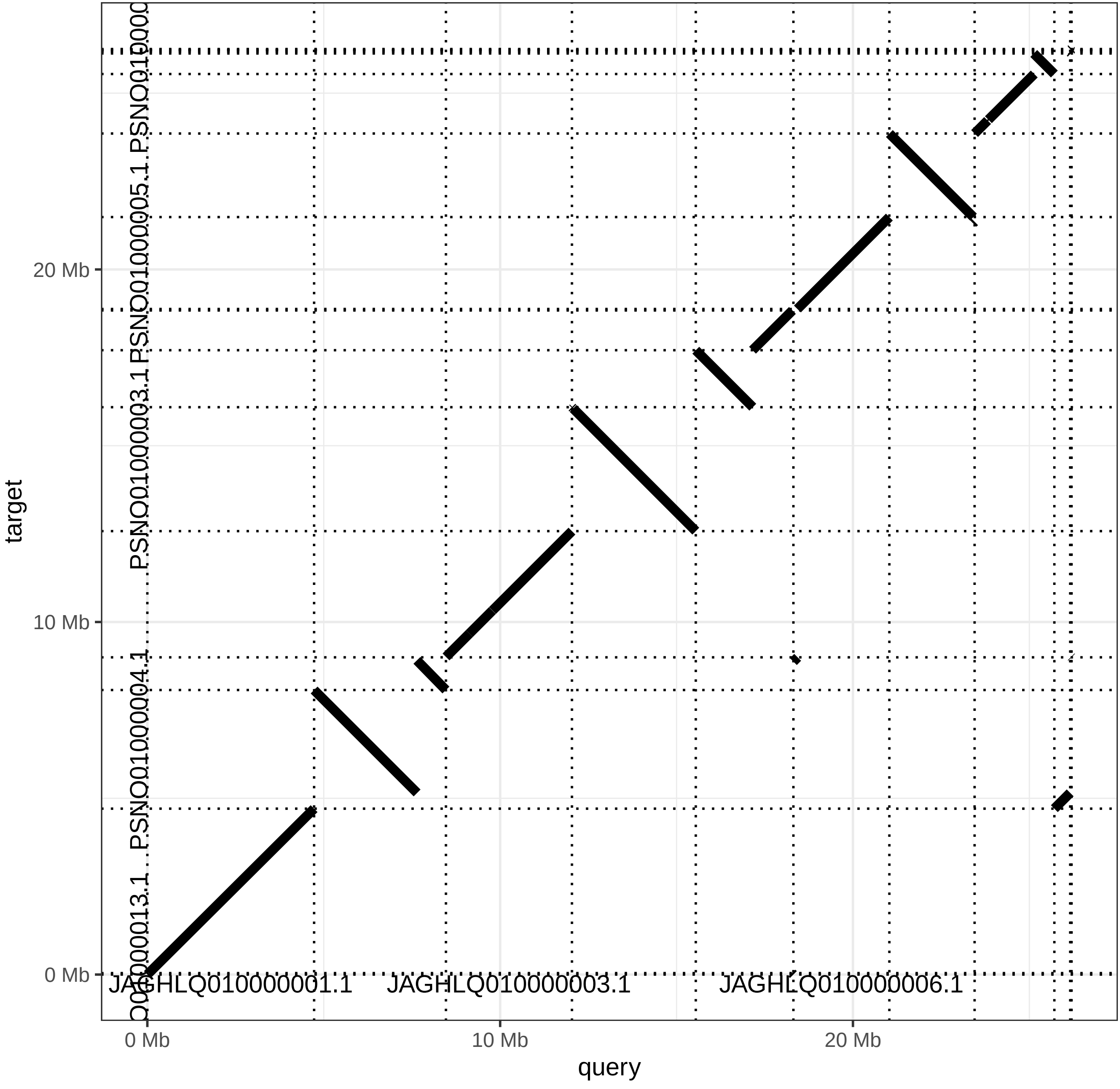

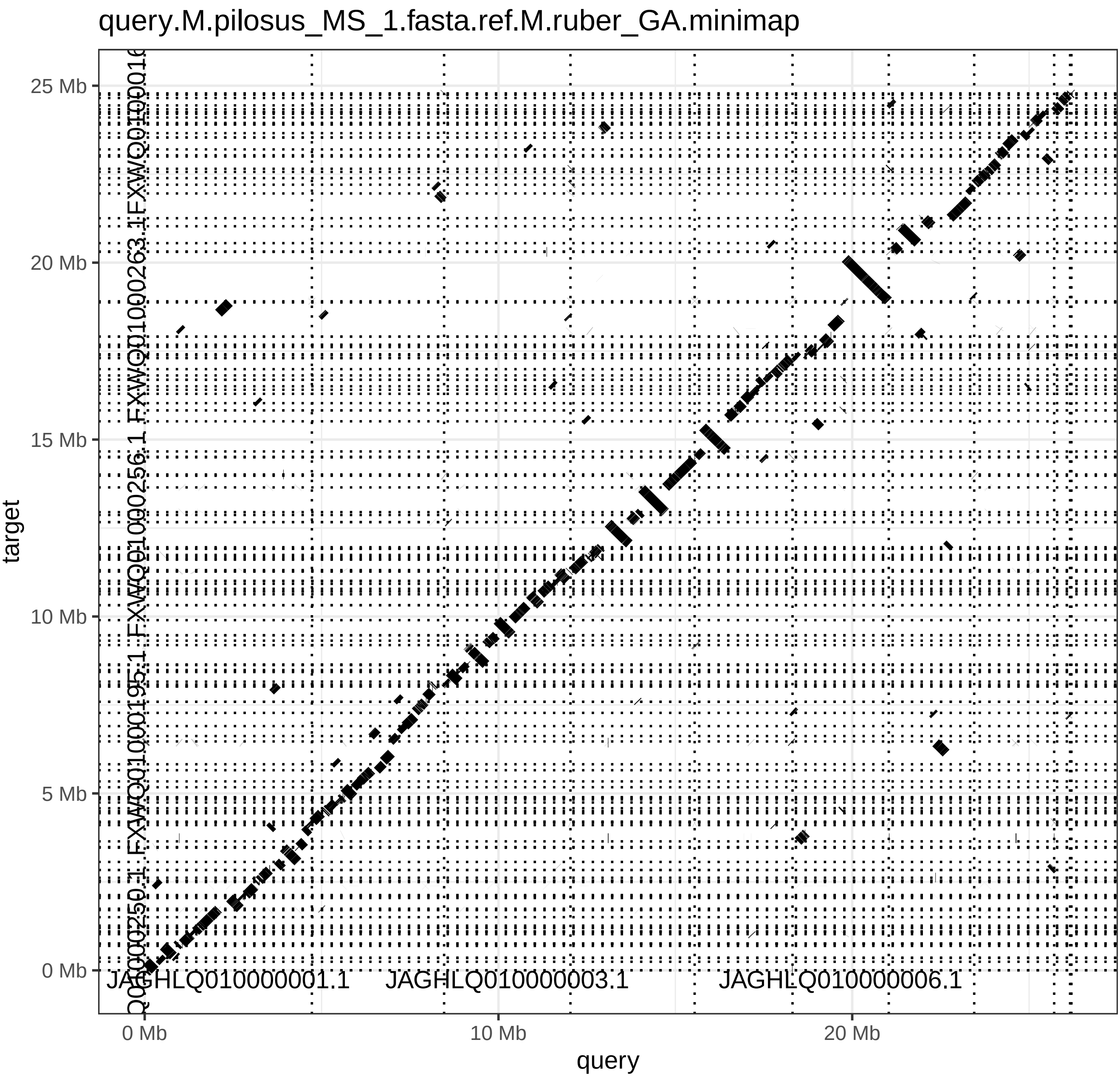

query.M.pilosus_MS_1.fasta.ref.M.ruber_GA.minimap
target
query
25 Mb
20 Mb
15 Mb
10 Mb
5 Mb
0 Mb
0 Mb
10 Mb
20 Mb
JAGHLQ010000001.1
JAGHLQ010000003.1
JAGHLQ010000006.1

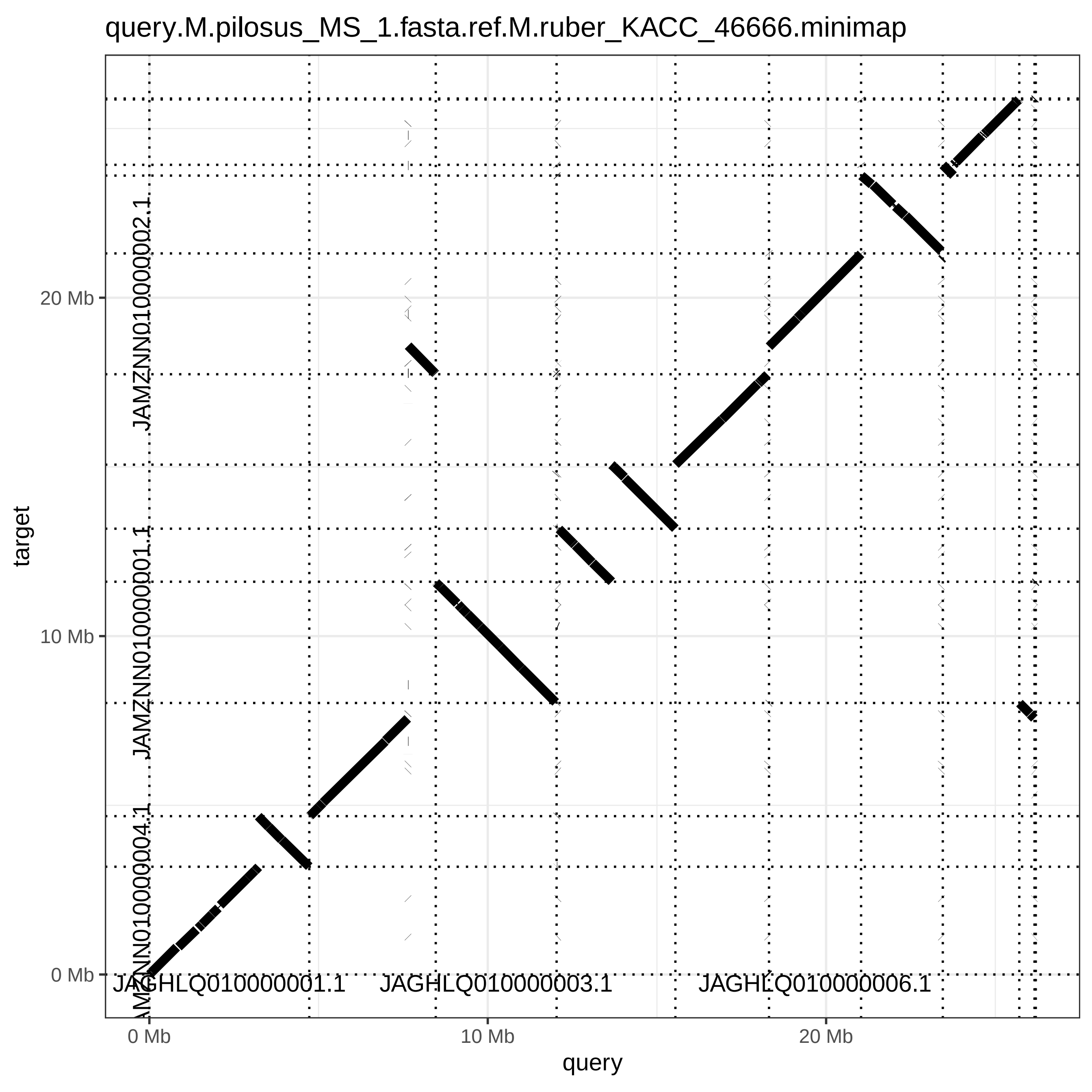

query.M.pilosus_MS_1.fasta.ref.M.ruber_KACC_46666.minimap
target
20 Mb
10 Mb
0 Mb
JAMZNN010000002.1
JAMZNN010000001.1
JAMZNN010000004.1
JAGHLQ010000001.1
JAGHLQ010000003.1
JAGHLQ010000006.1
0 Mb
10 Mb
20 Mb
query

query.M.pilosus_YDJ_1.fasta.ref.M.pilosus_K104061.minimap

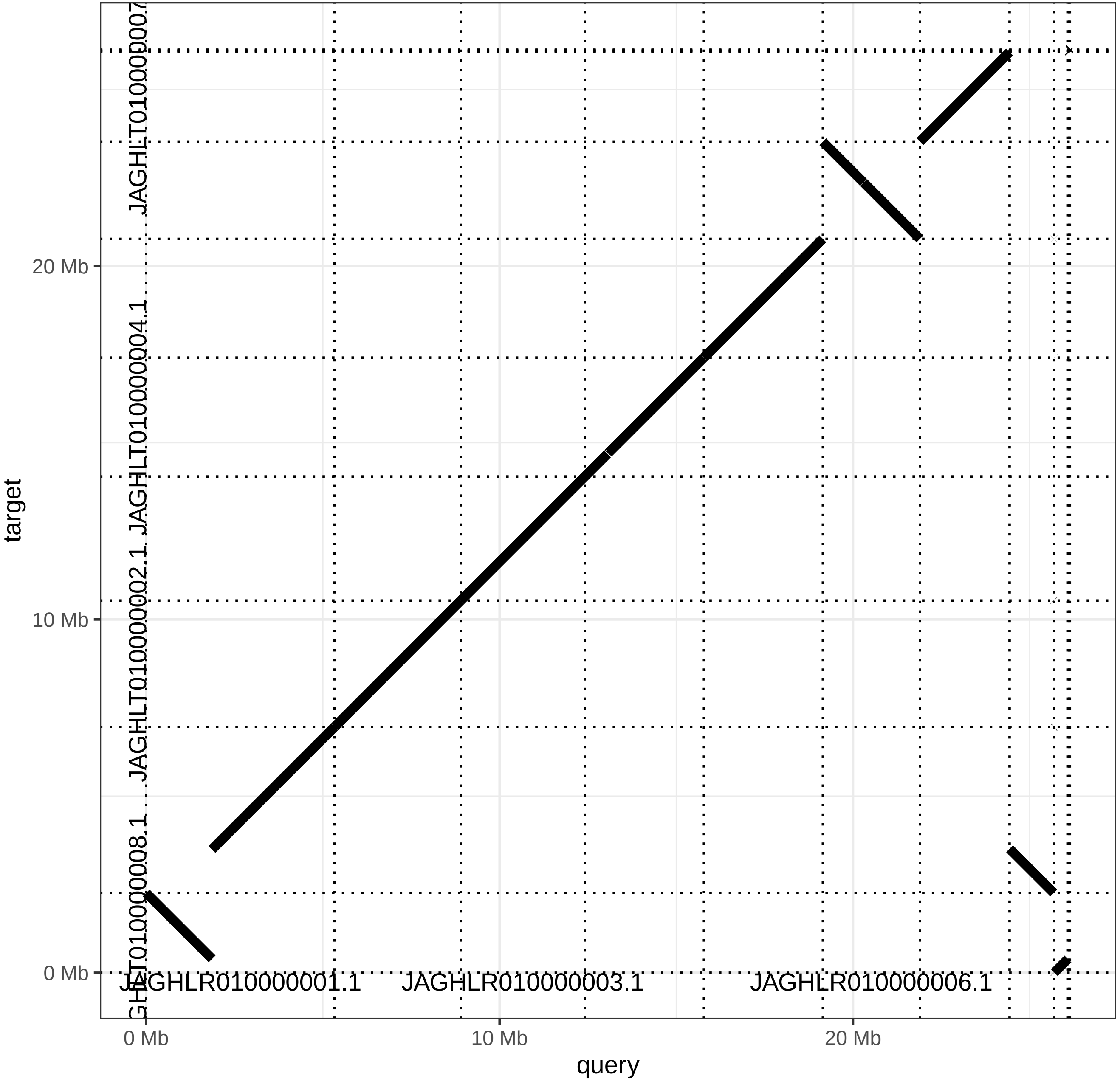

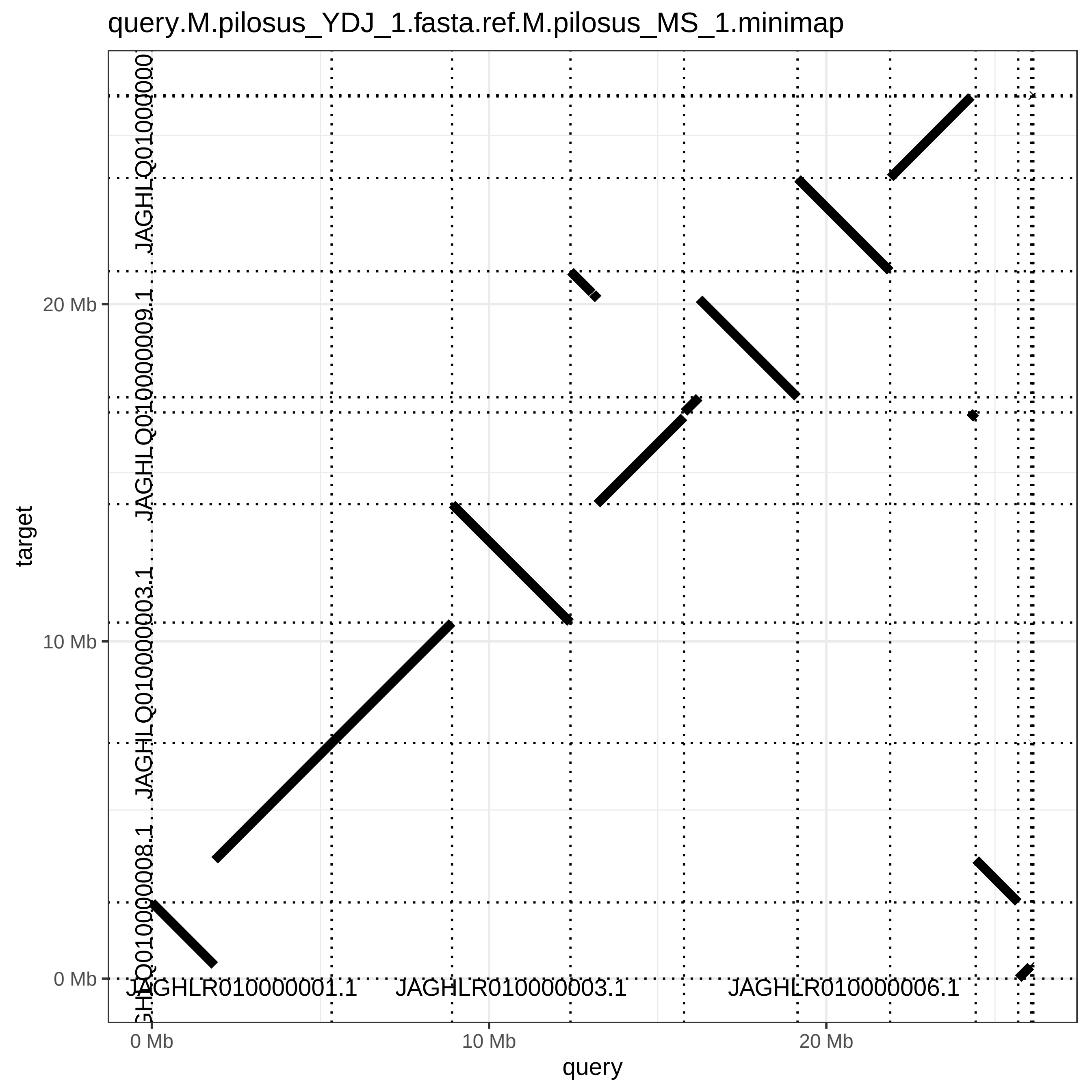
query.M.pilosus_YDJ_1.fasta.ref.M.pilosus_MS_1.minimap
target
query
0 Mb
10 Mb
20 Mb
JAGHLR010000001.1
JAGHLR010000003.1
JAGHLR010000006.1
JAGHLQ010000003.1
JAGHLQ010000009.1

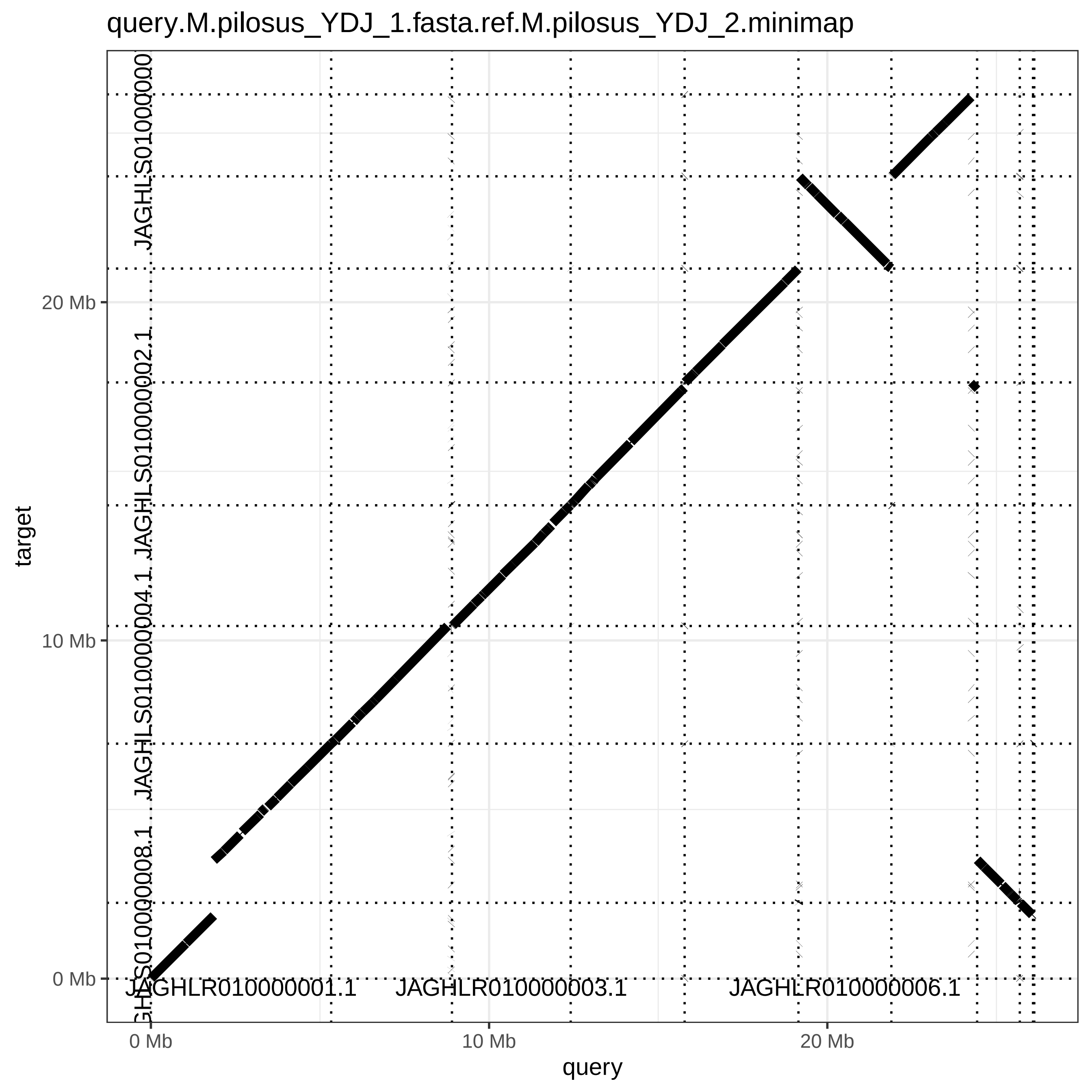
query.M.pilosus_YDJ_1.fasta.ref.M.pilosus_YDJ_2.minimap
target
query
0 Mb
10 Mb
20 Mb
JAGHLR010000001.1
JAGHLR010000003.1
JAGHLR010000006.1
JAGHLS010000002.1
JAGHLS010000004.1
JAGHLS010000008.1

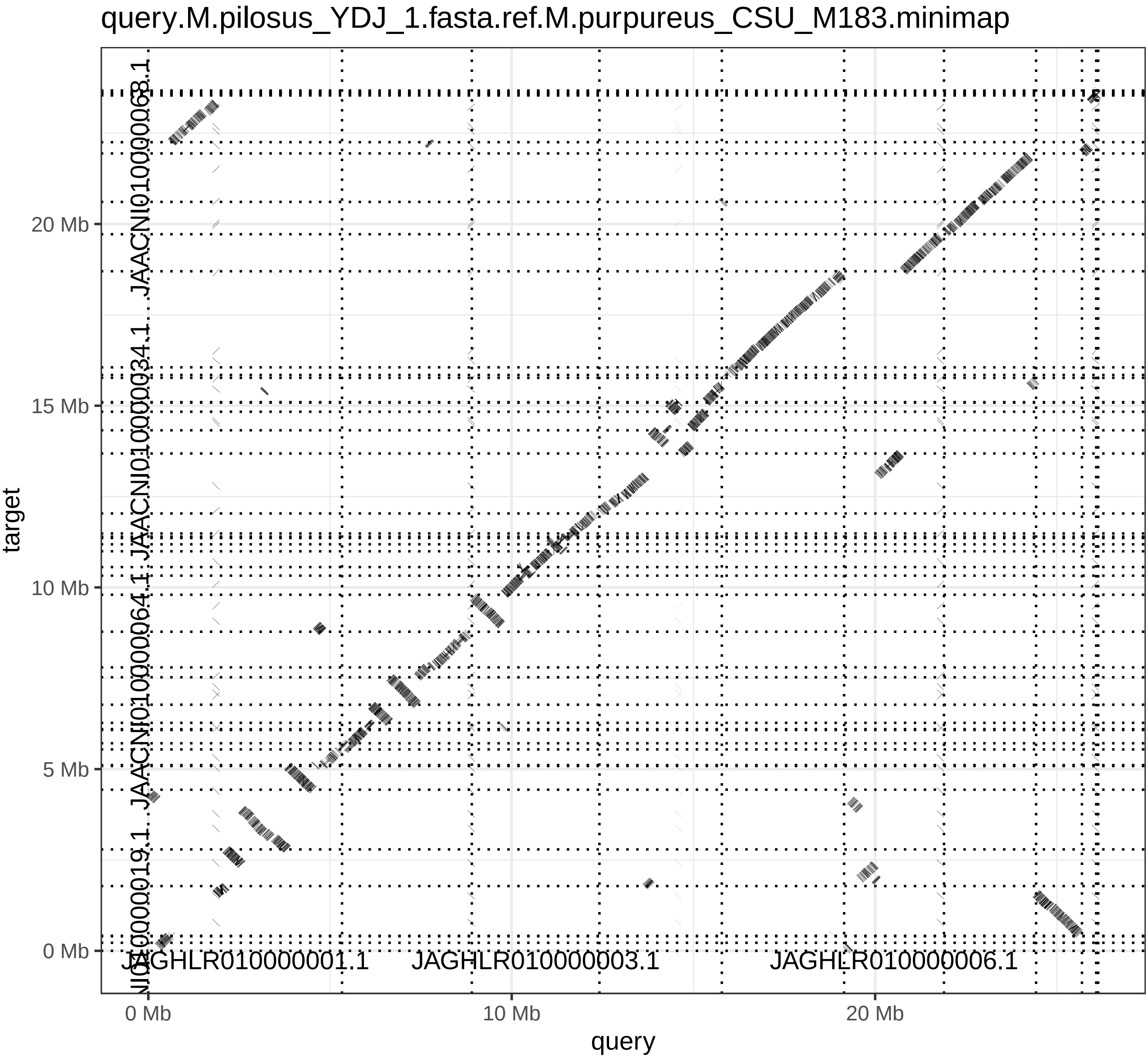
query.M.pilosus_YDJ_1.fasta.ref.M.purpureus_CSU_M183.minimap
target
query
0 Mb
5 Mb
10 Mb
15 Mb
20 Mb
0 Mb
10 Mb
20 Mb
JAGHLR010000001.1
JAGHLR010000003.1
JAGHLR010000006.1

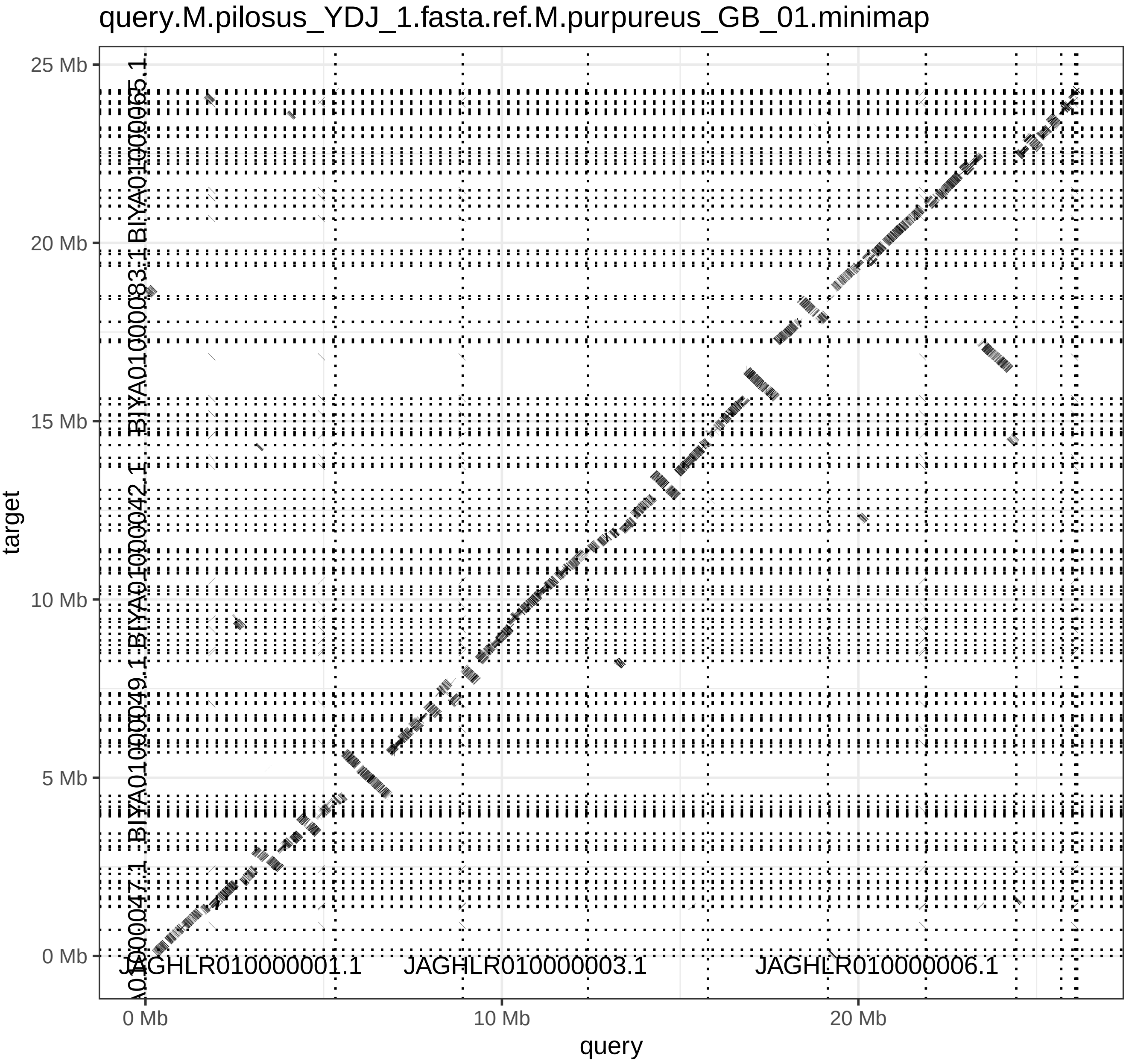
query.M.pilosus_YDJ_1.fasta.ref.M.purpureus_GB_01.minimap
target
query
25 Mb
20 Mb
15 Mb
10 Mb
5 Mb
0 Mb
0 Mb
10 Mb
20 Mb
JAGHLR010000001.1
JAGHLR010000003.1
JAGHLR010000006.1
BIYA010000065.1
BIYA010000083.1
BIYA010000042.1
BIYA010000049.1
BIYA010000047.1

query.M.pilosus_YDJ_1.fasta.ref.M.purpureus_HQ1.minimap

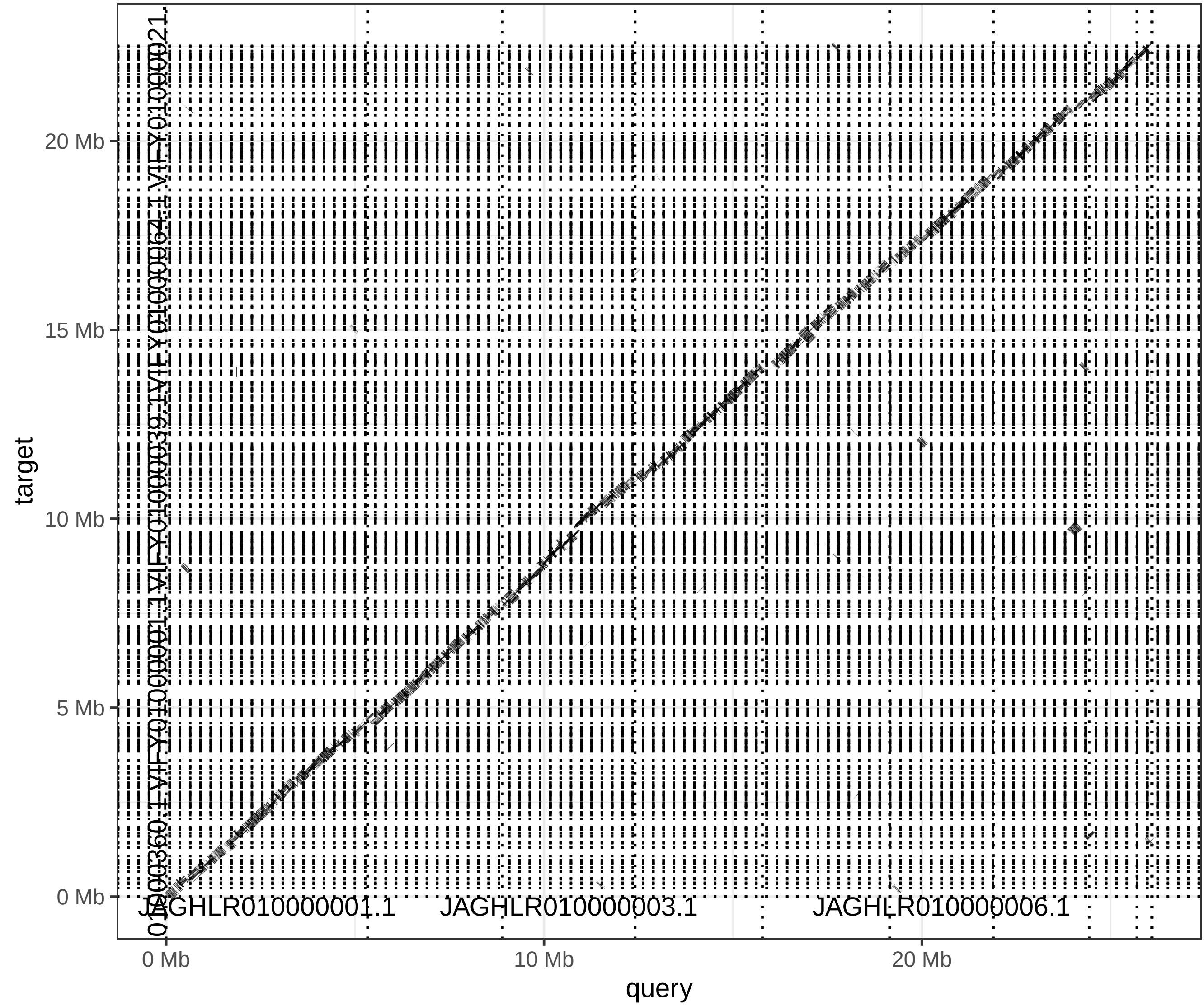

query.M.pilosus_YDJ_1.fasta.ref.M.purpureus_P7048X_2.minimap

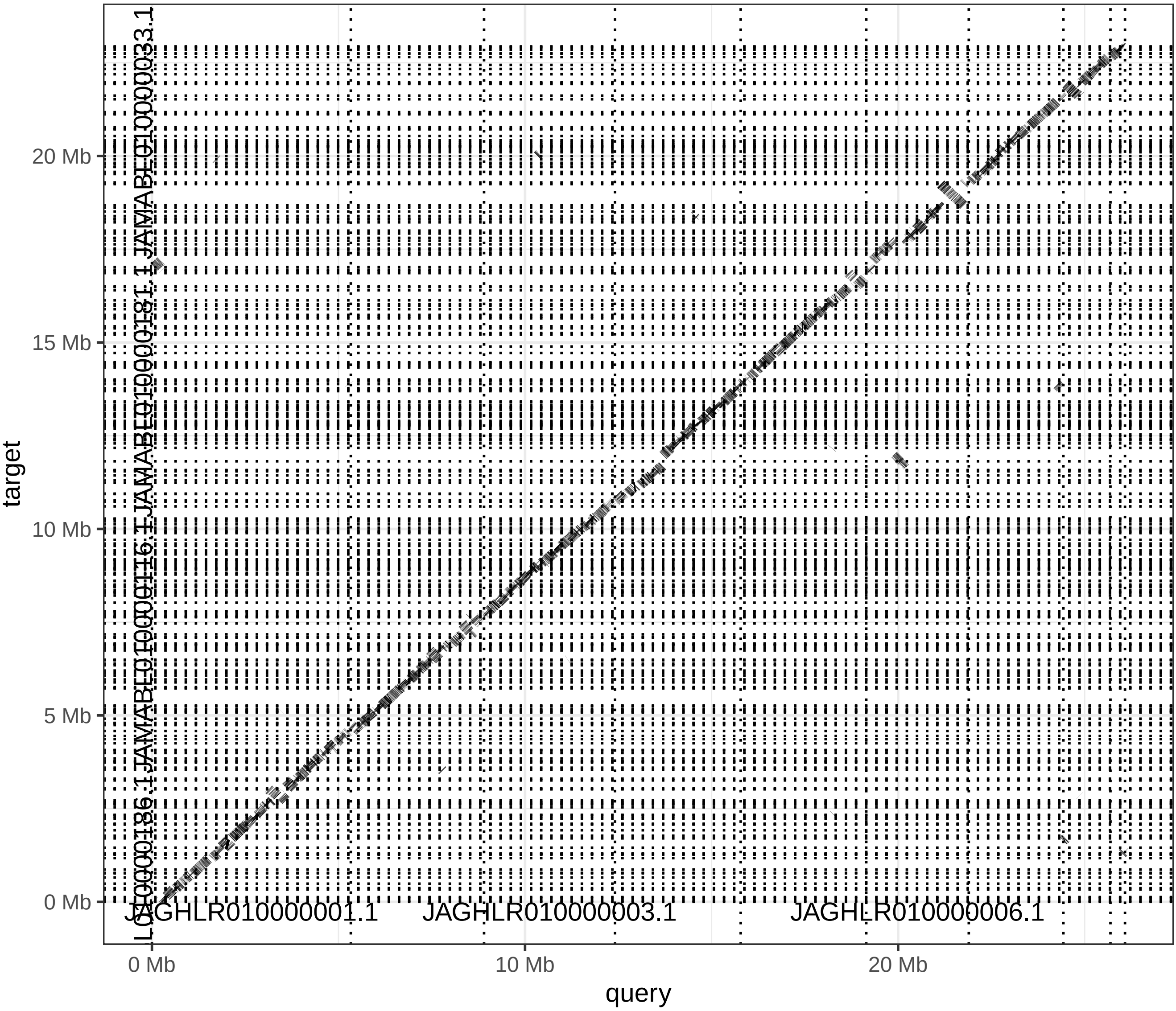

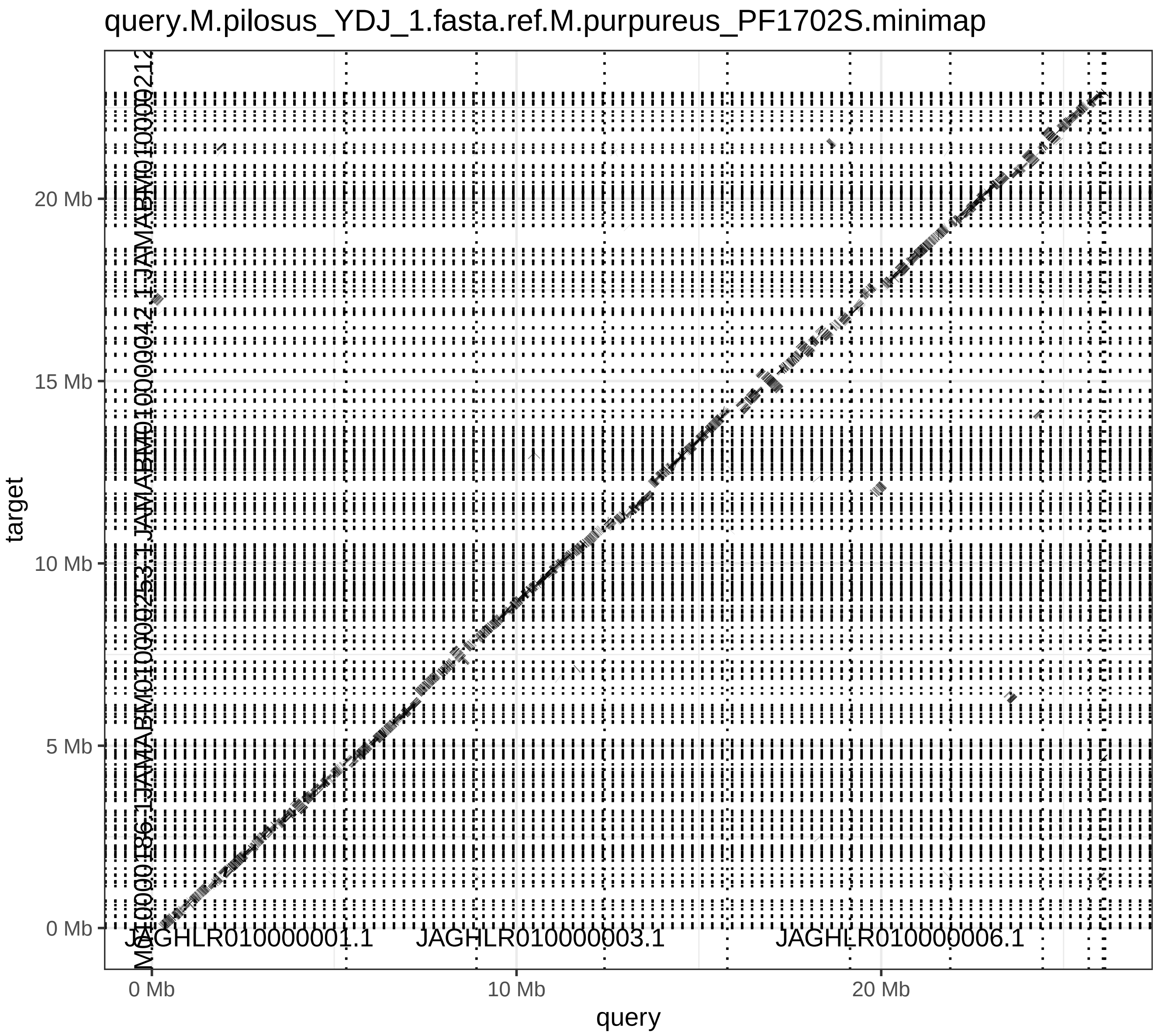
query.M.pilosus_YDJ_1.fasta.ref.M.purpureus_PF1702S.minimap
target
20 Mb
15 Mb
10 Mb
5 Mb
0 Mb
JAGHLR010000001.1
JAGHLR010000003.1
JAGHLR010000006.1
0 Mb
10 Mb
20 Mb
query

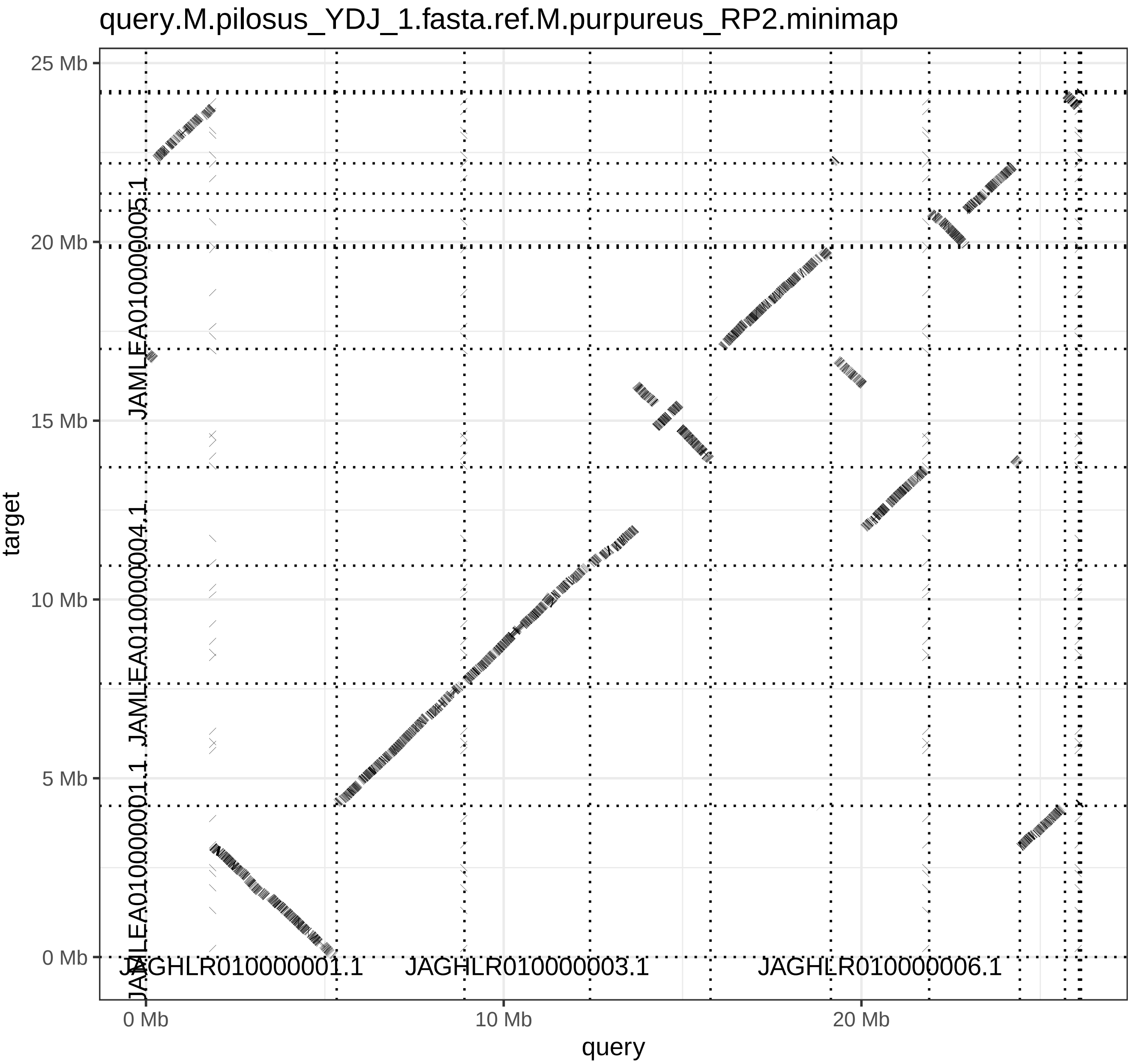
query.M.pilosus_YDJ_1.fasta.ref.M.purpureus_RP2.minimap
25 Mb
20 Mb
15 Mb
10 Mb
5 Mb
0 Mb
target
JAMLEA010000005.1
JAMLEA010000004.1
JAMLEA010000001.1
JAMLEA010000001.1
JAGHLR010000001.1
JAGHLR010000003.1
JAGHLR010000006.1
0 Mb
10 Mb
20 Mb
query

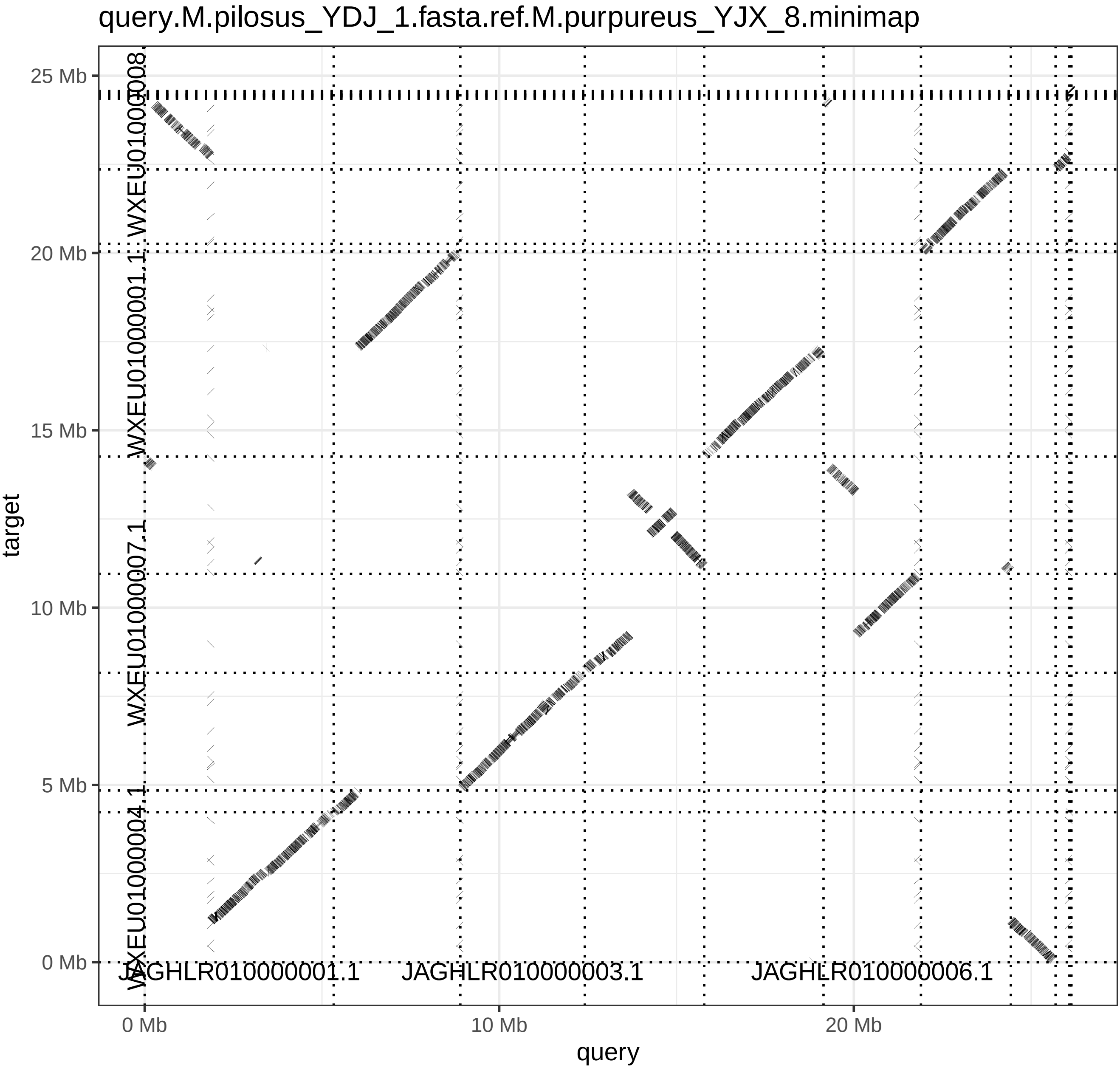
query.M.pilosus_YDJ_1.fasta.ref.M.purpureus_YJX_8.minimap
target
query
0 Mb
5 Mb
10 Mb
15 Mb
20 Mb
25 Mb
0 Mb
10 Mb
20 Mb
JAGHLR010000001.1
JAGHLR010000003.1
JAGHLR010000006.1
WXEU01000004.1
WXEU01000007.1
WXEU01000001.1
WXEU01000008

query.M.pilosus_YDJ_1.fasta.ref.M.purpureus_YY_1.minimap

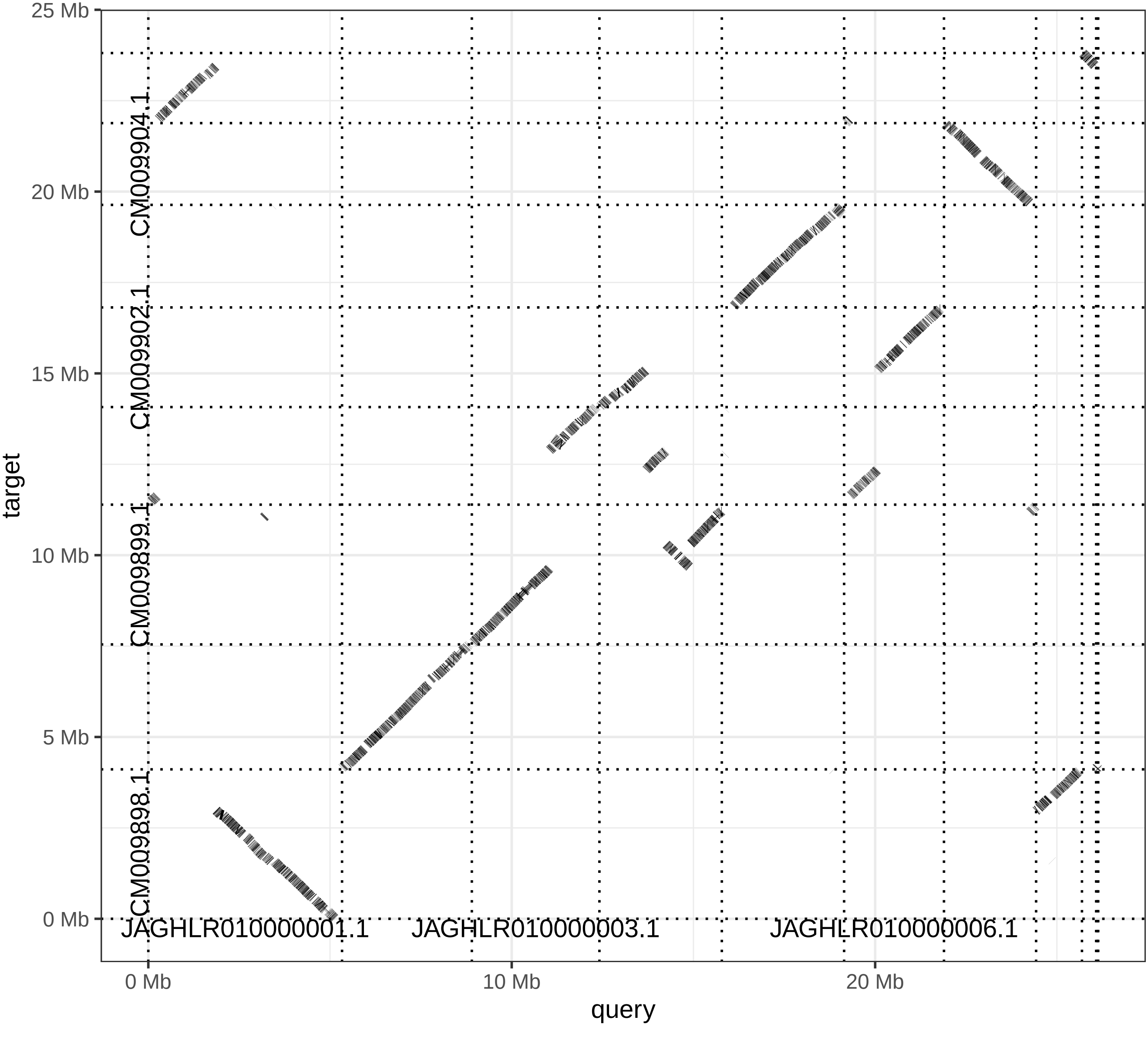

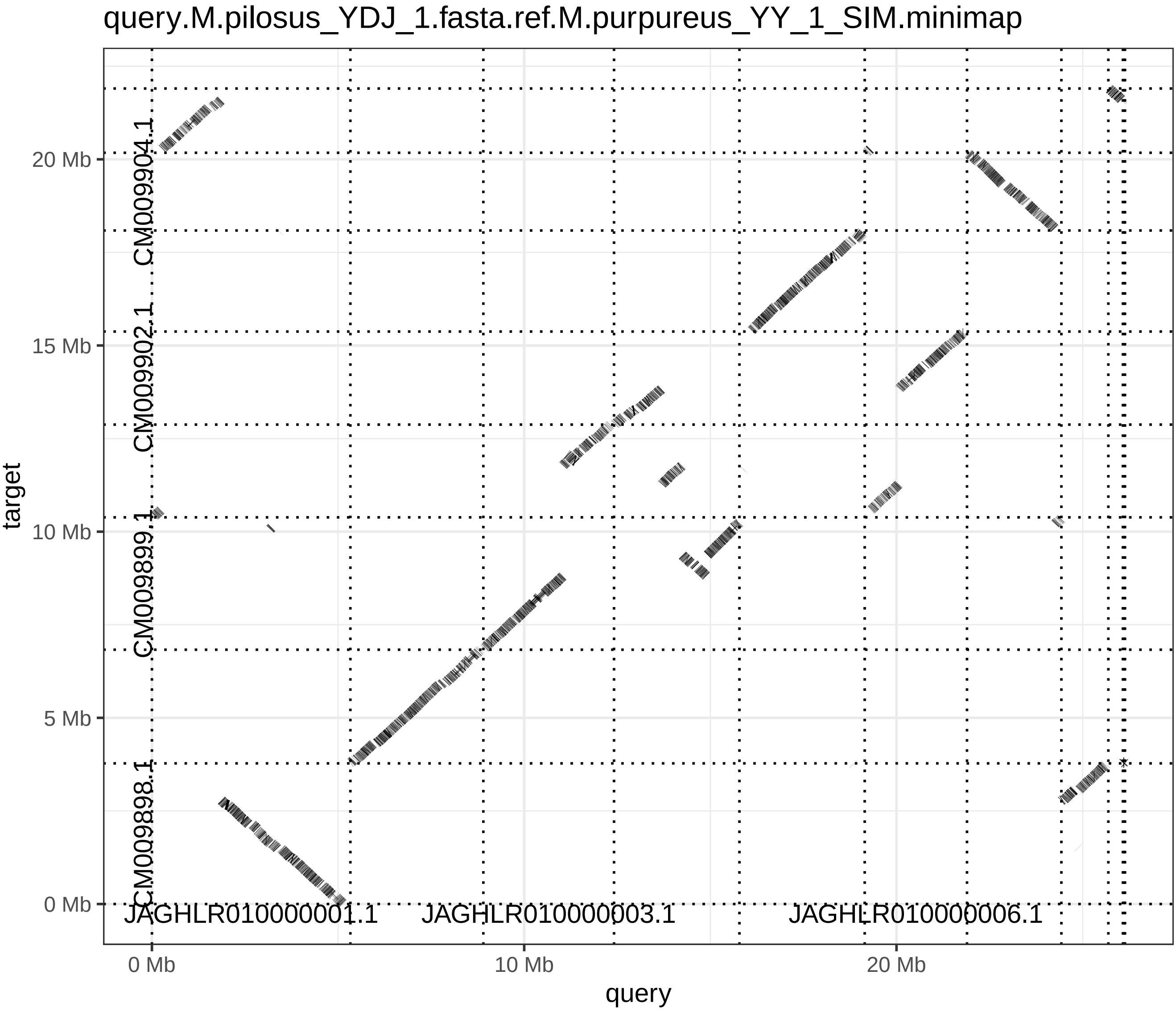
query.M.pilosus_YDJ_1.fasta.ref.M.purpureus_YY_1_SIM.minimap
target
query
CM009904.1
CM009902.1
CM009899.1
CM009898.1
20 Mb
15 Mb
10 Mb
5 Mb
0 Mb
JAGHLR010000001.1
JAGHLR010000003.1
JAGHLR010000006.1
0 Mb
10 Mb
20 Mb

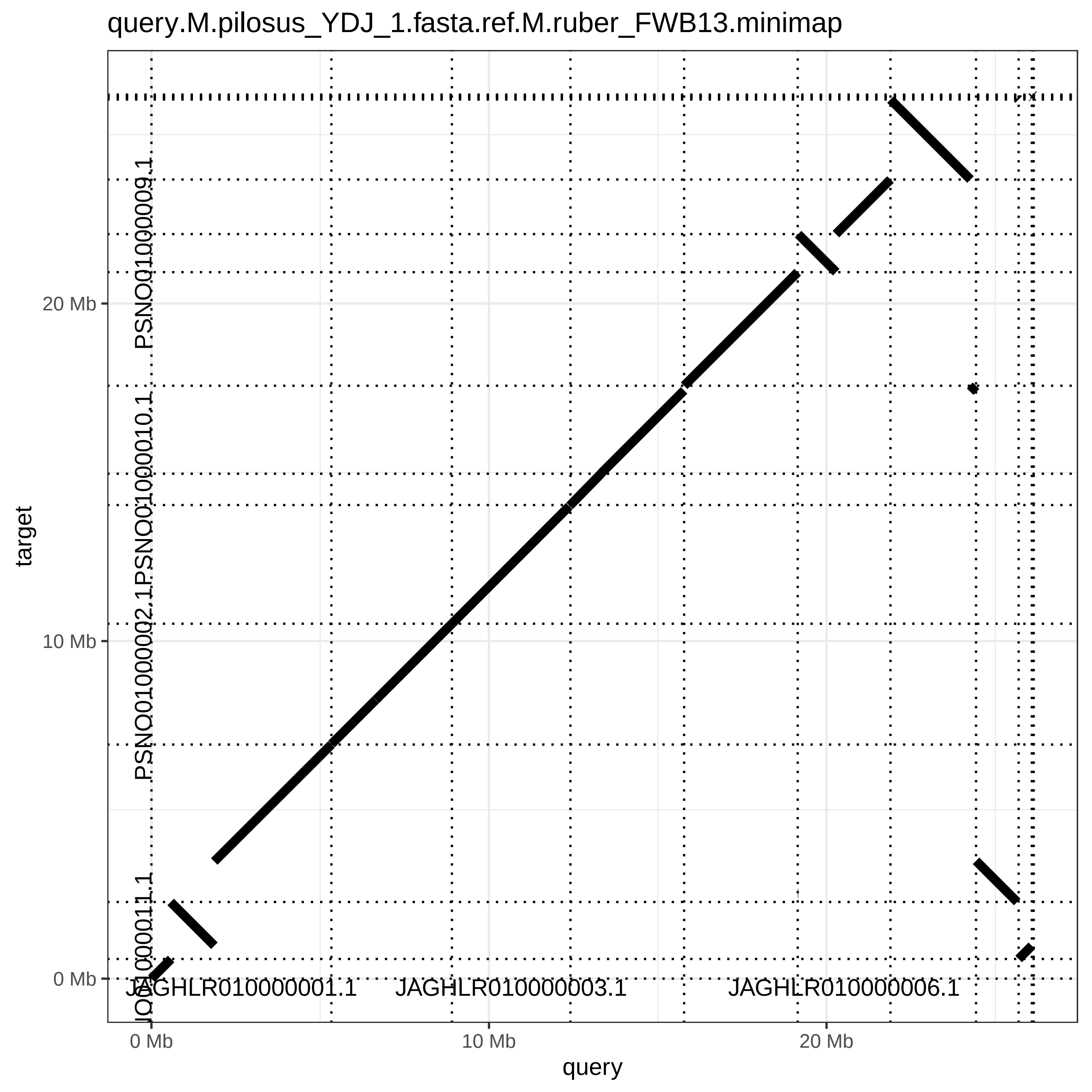
query.M.pilosus_YDJ_1.fasta.ref.M.ruber_FWB13.minimap
target
PSNO010000009.1
PSNO010000010.1
PSNO010000002.1
20 Mb
10 Mb
0 Mb
JAGHLR010000001.1
JAGHLR010000003.1
JAGHLR010000006.1
0 Mb
10 Mb
20 Mb
query

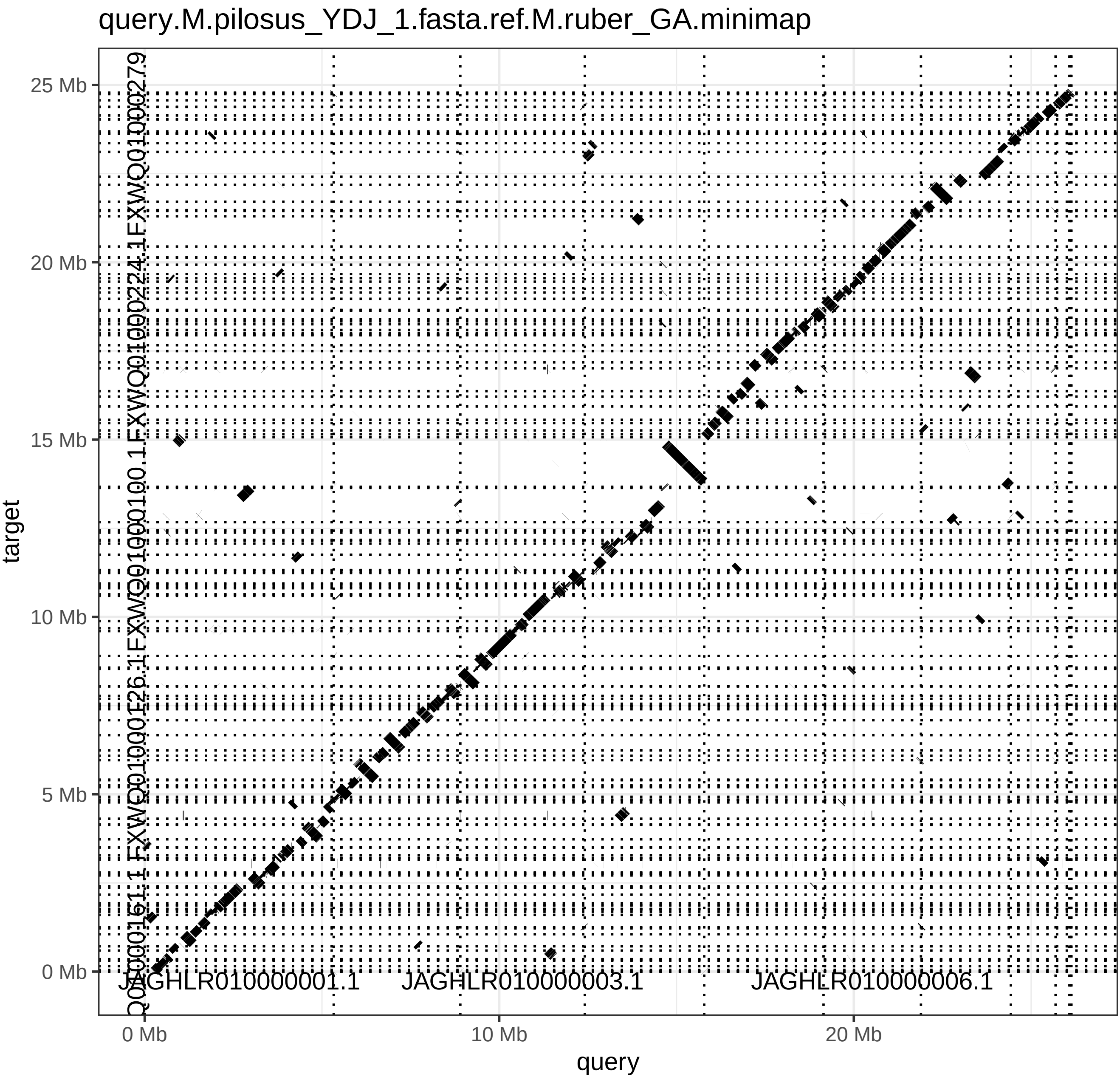
query.M.pilosus_YDJ_1.fasta.ref.M.ruber_GA.minimap
target
25 Mb
20 Mb
15 Mb
10 Mb
5 Mb
0 Mb
JAGHLR010000001.1
JAGHLR010000003.1
JAGHLR010000006.1
0 Mb
10 Mb
20 Mb
query

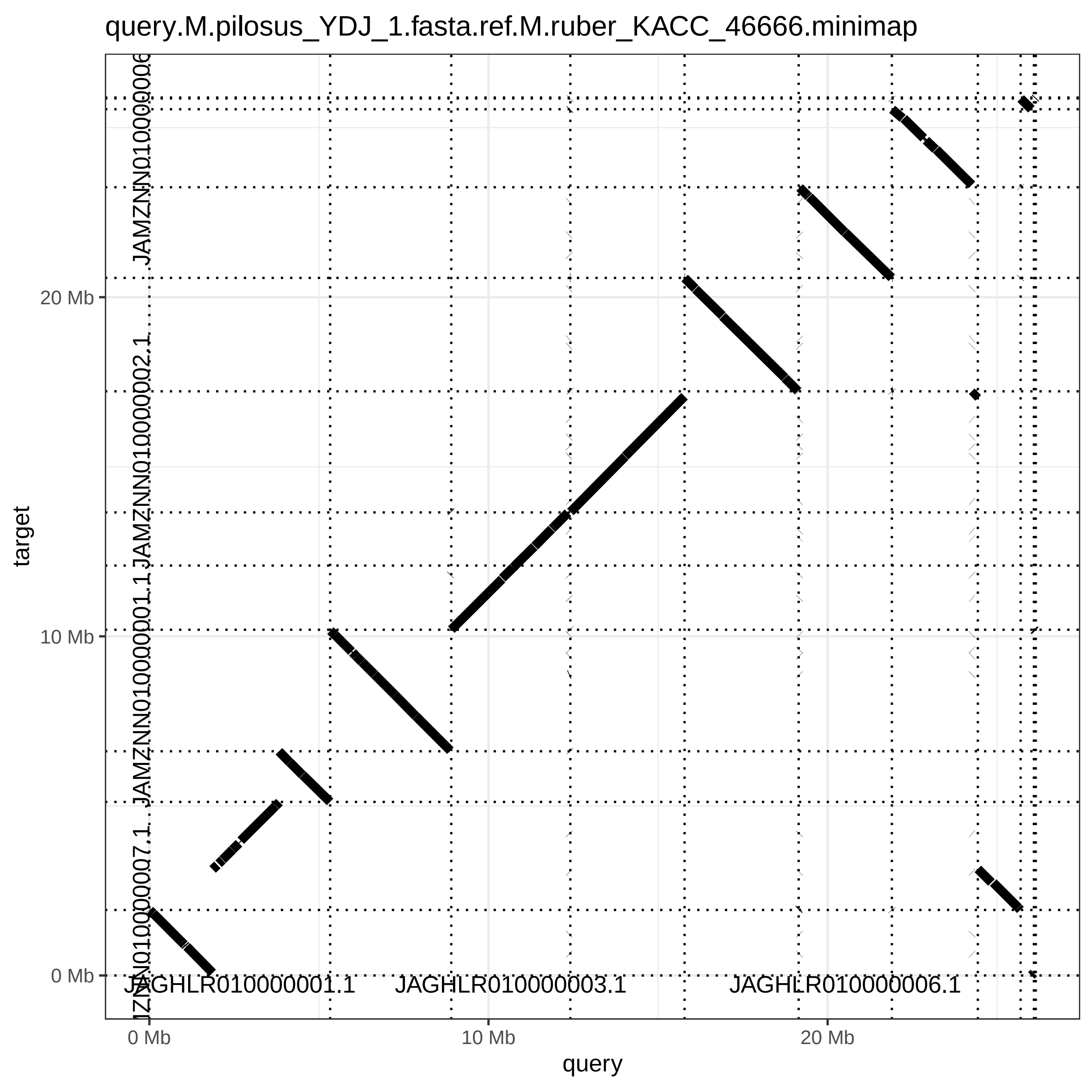

query.M.pilosus_YDJ_1.fasta.ref.M.ruber_KACC_46666.minimap
target
query
20 Mb
10 Mb
0 Mb
0 Mb
10 Mb
20 Mb
JAGHLR010000001.1
JAGHLR010000003.1
JAGHLR010000006.1
JAMZNN010000006
JAMZNN010000002.1
JAMZNN010000001.1
JAMZNN010000007.1

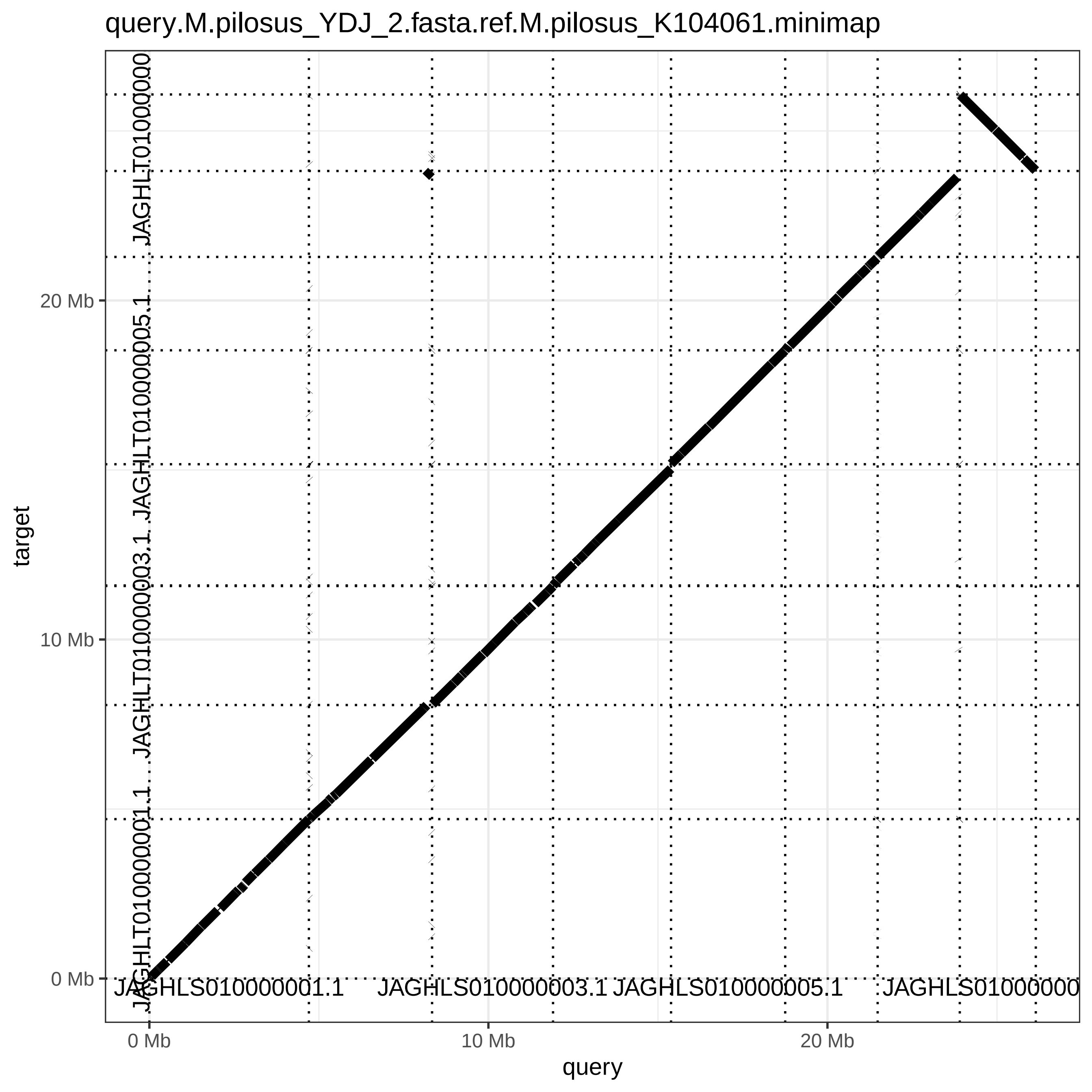

query.M.pilosus_YDJ_2.fasta.ref.M.pilosus_K104061.minimap
target
query
0 Mb
10 Mb
20 Mb
JAGHLS010000001.1
JAGHLS010000003.1
JAGHLS010000005.1
JAGHLT010000001.1
JAGHLT010000003.1
JAGHLT010000005.1

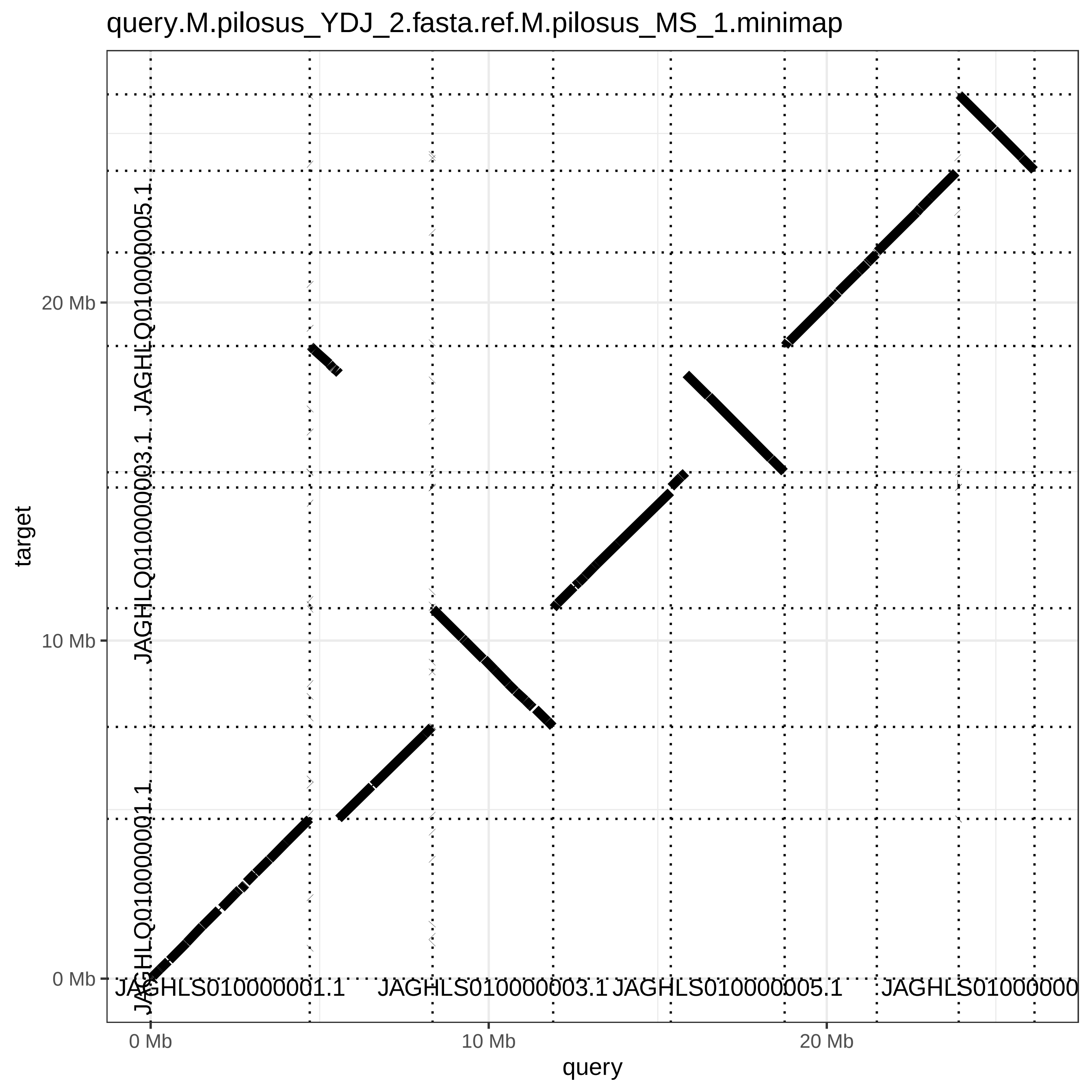
query.M.pilosus_YDJ_2.fasta.ref.M.pilosus_MS_1.minimap
target
query
0 Mb
10 Mb
20 Mb
JAGHLQ010000001.1
JAGHLQ010000003.1
JAGHLQ010000005.1
JAGHLS010000001.1
JAGHLS010000003.1
JAGHLS010000005.1
JAGHLS01000000

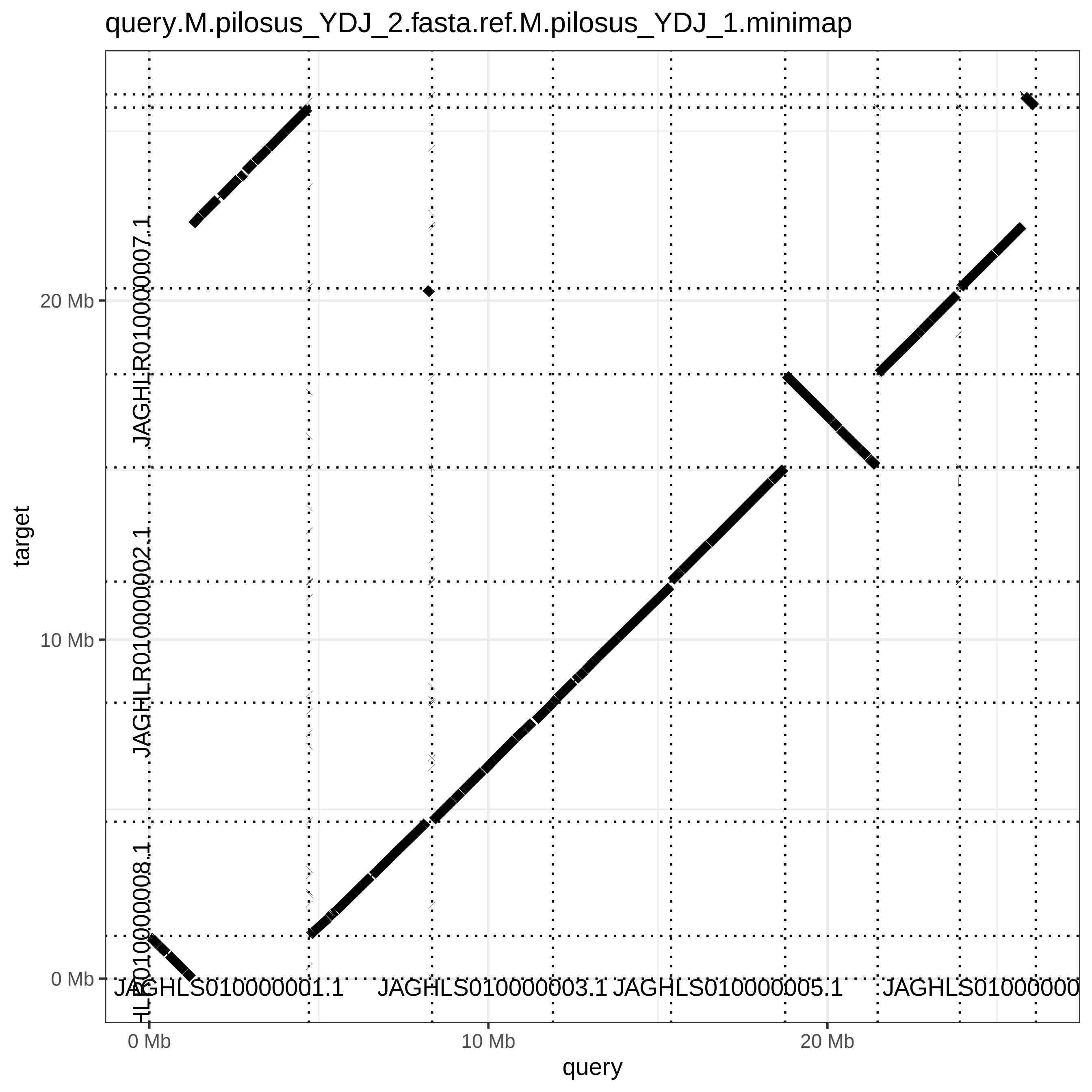
query.M.pilosus_YDJ_2.fasta.ref.M.pilosus_YDJ_1.minimap
target
query
0 Mb
10 Mb
20 Mb
JAGHLR010000007.1
JAGHLR010000002.1
JAGHLR010000008.1
JAGHLS010000001.1
JAGHLS010000003.1
JAGHLS010000005.1
JAGHLS01000000

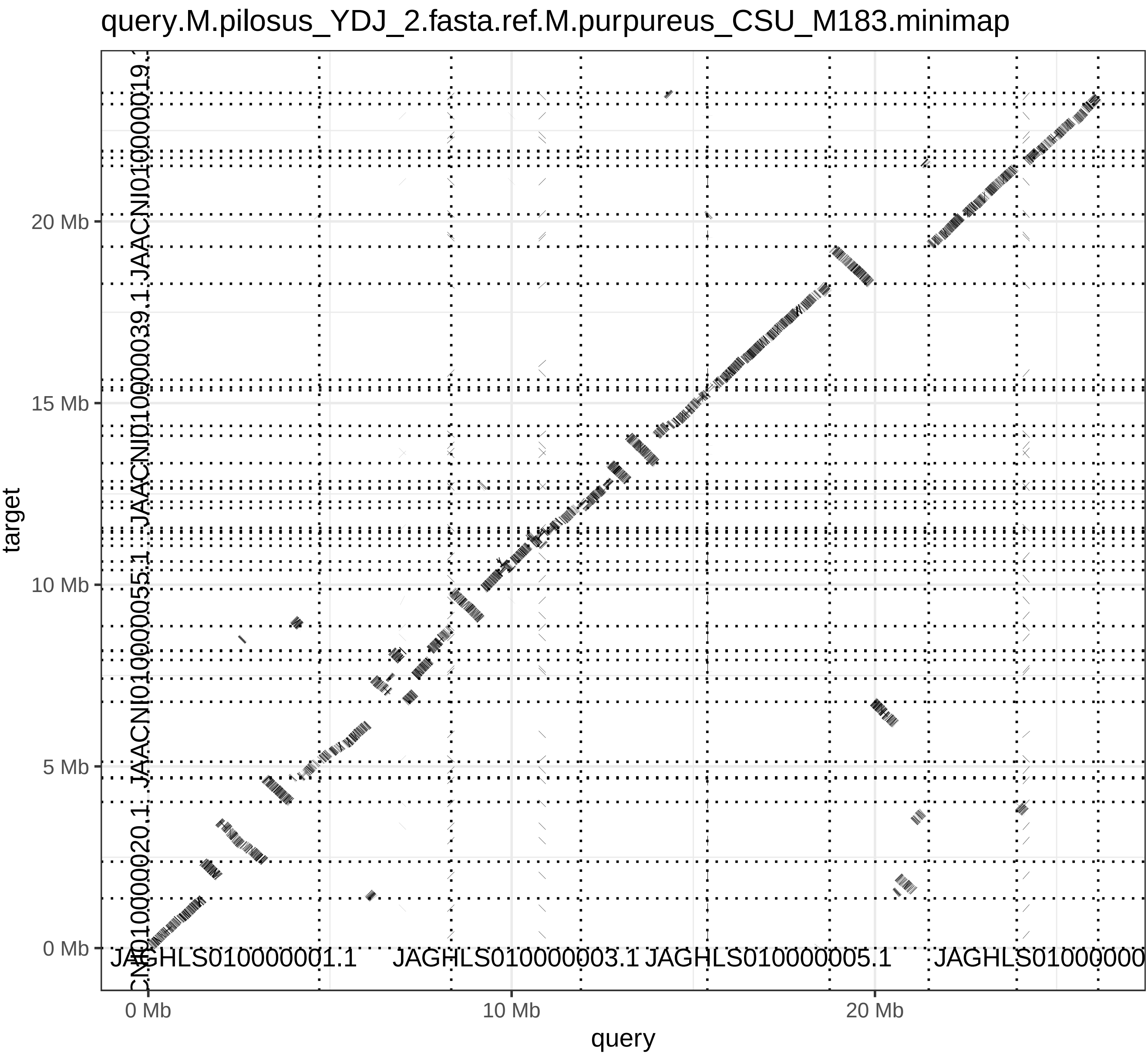
query.M.pilosus_YDJ_2.fasta.ref.M.purpureus_CSU_M183.minimap
target
query
0 Mb
5 Mb
10 Mb
15 Mb
20 Mb
0 Mb
10 Mb
20 Mb
JAGHLS010000001.1
JAGHLS010000003.1
JAGHLS010000005.1
JAACNI010000019.1
JAACNI010000039.1
JAACNI010000055.1
JAACNI010000020.1

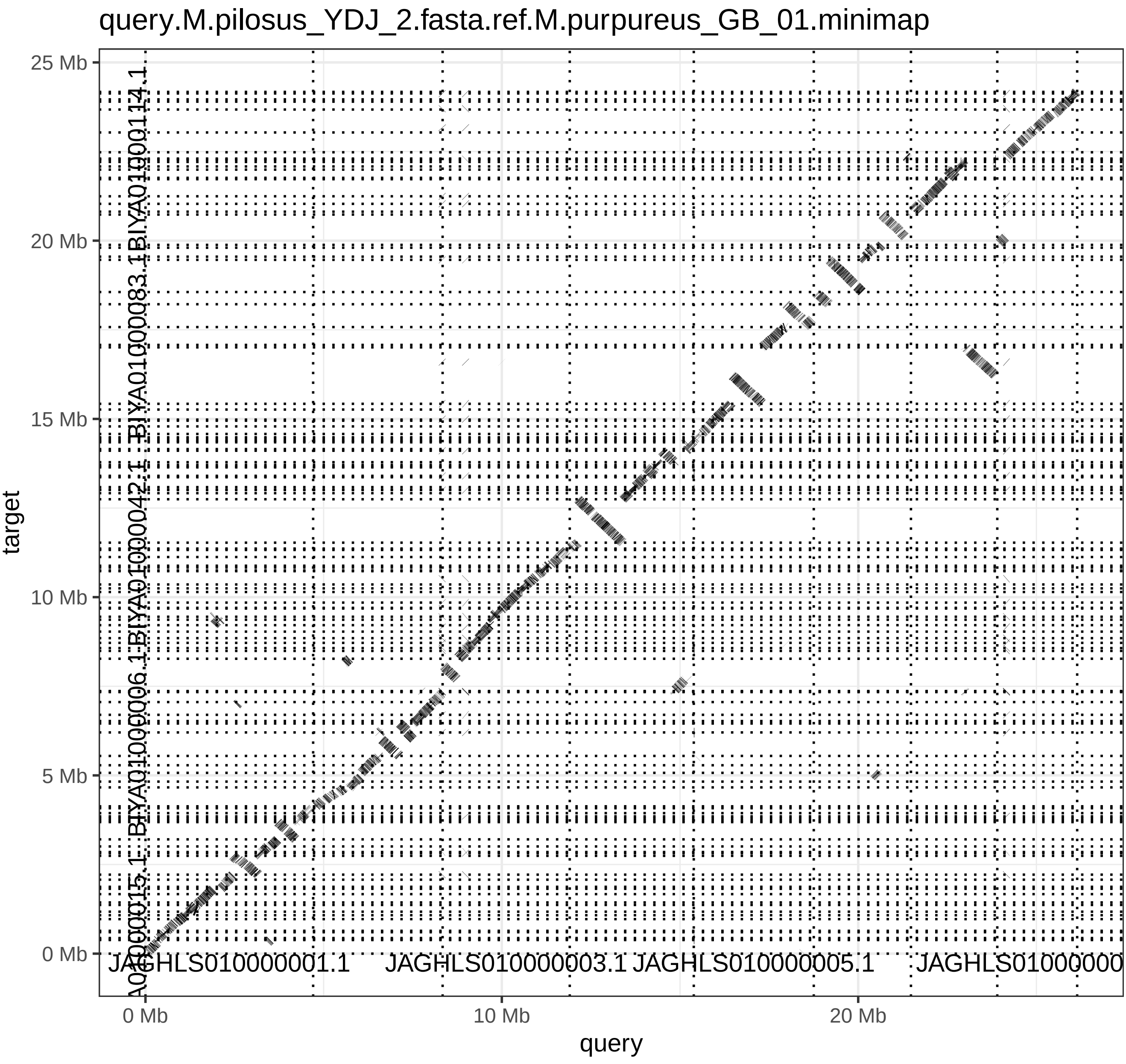
query.M.pilosus_YDJ_2.fasta.ref.M.purpureus_GB_01.minimap
target
query
25 Mb
20 Mb
15 Mb
10 Mb
5 Mb
0 Mb
0 Mb
10 Mb
20 Mb
JAGHLS010000001.1
JAGHLS010000003.1
JAGHLS010000005.1
BIYA010000083.1
BIYA010000042.1

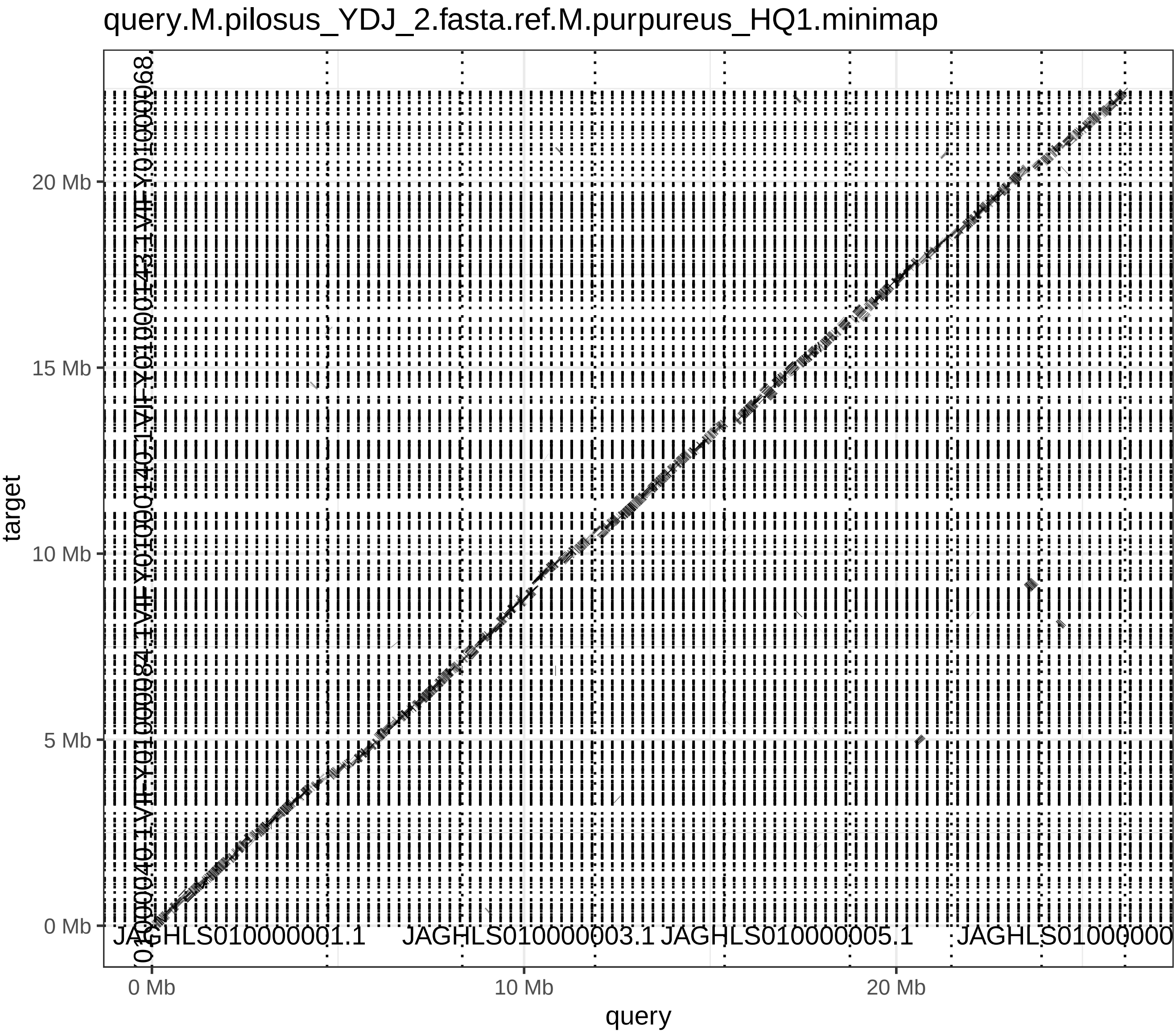

query.M.pilosus_YDJ_2.fasta.ref.M.purpureus_HQ1.minimap
target
query
20 Mb
15 Mb
10 Mb
5 Mb
0 Mb
0 Mb
10 Mb
20 Mb
JAGHLS010000001.1
JAGHLS010000003.1
JAGHLS010000005.1

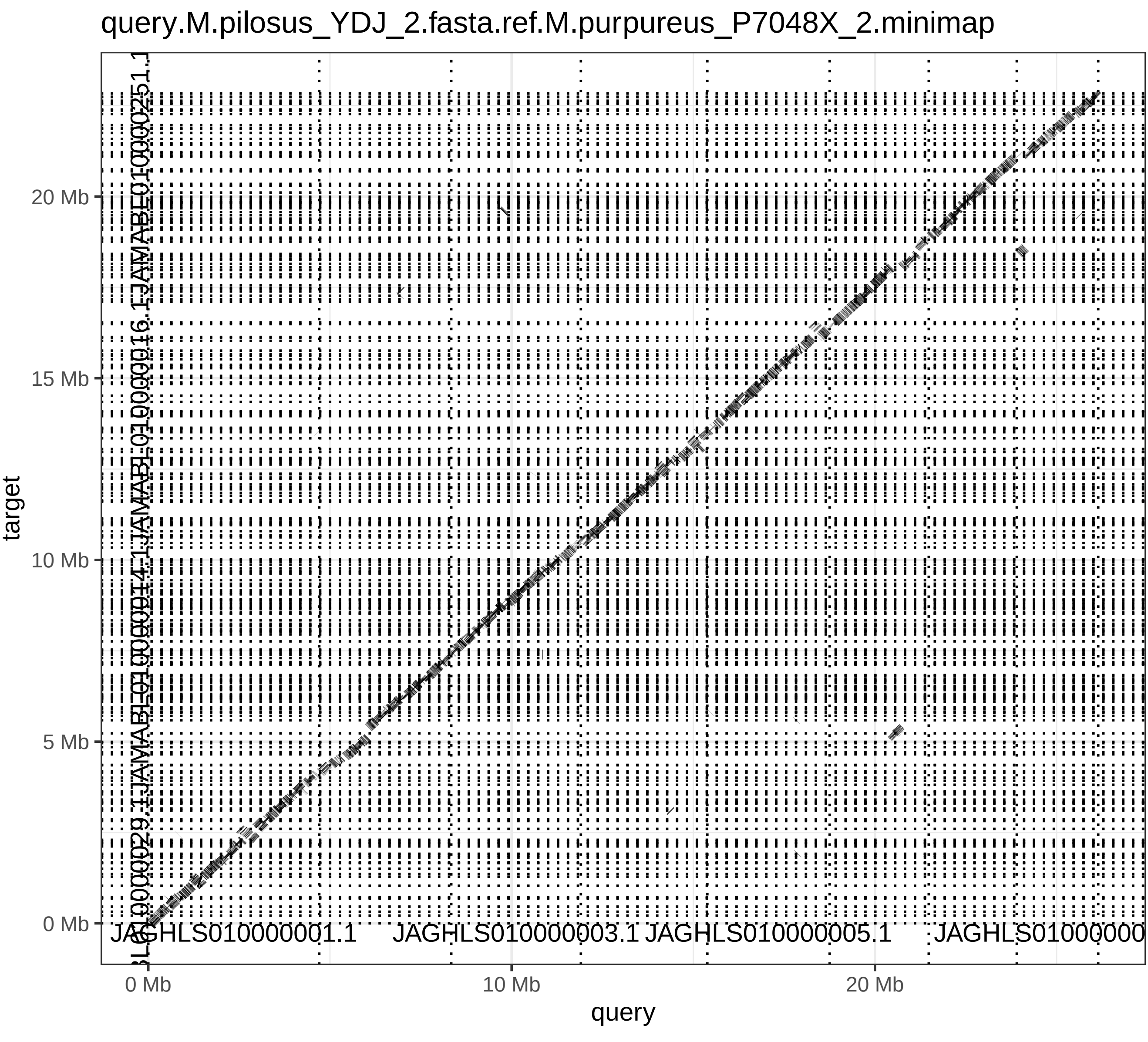
query.M.pilosus_YDJ_2.fasta.ref.M.purpureus_P7048X_2.minimap
target
20 Mb
15 Mb
10 Mb
5 Mb
0 Mb
query
0 Mb
10 Mb
20 Mb
JAGHLS010000001.1
JAGHLS010000003.1
JAGHLS010000005.1

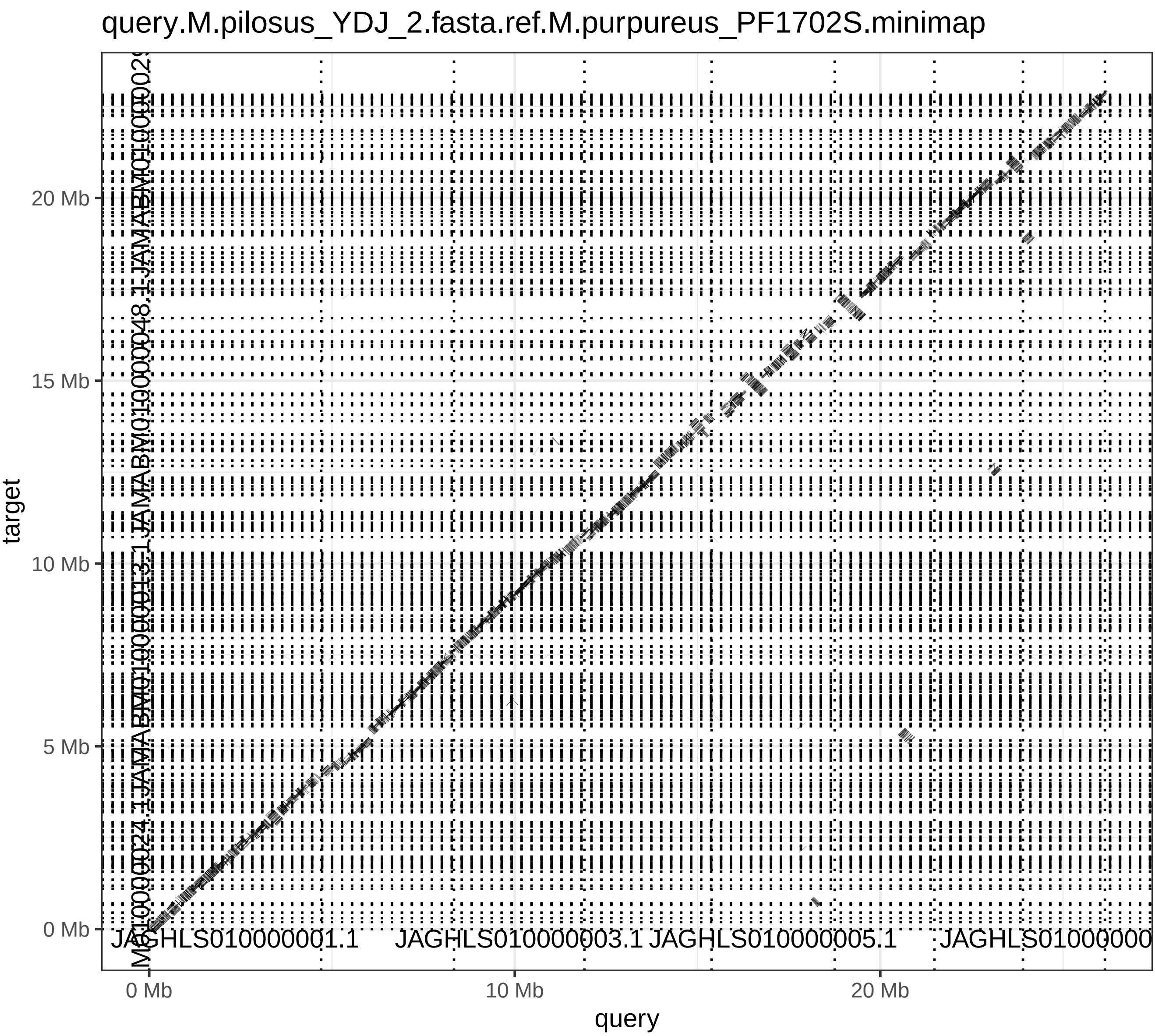
query.M.pilosus_YDJ_2.fasta.ref.M.purpureus_PF1702S.minimap
target
20 Mb
15 Mb
10 Mb
5 Mb
0 Mb
JAGHLS010000001.1
JAGHLS010000003.1
JAGHLS010000005.1
0 Mb
10 Mb
20 Mb
query

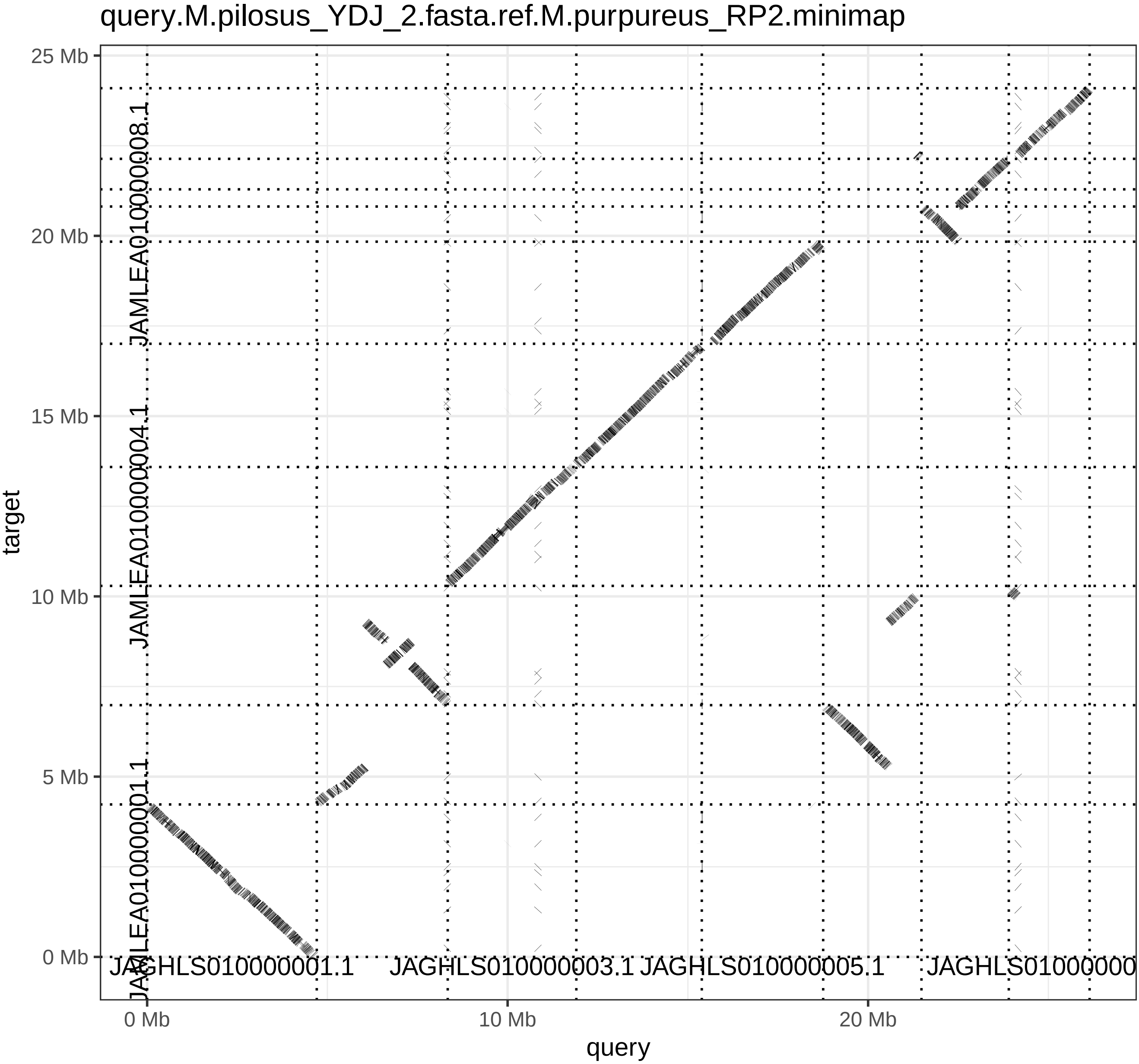
query.M.pilosus_YDJ_2.fasta.ref.M.purpureus_RP2.minimap
target
query
25 Mb
20 Mb
15 Mb
10 Mb
5 Mb
0 Mb
0 Mb
10 Mb
20 Mb
JAMLEA010000008.1
JAMLEA010000004.1
JAMLEA010000001.1
JAGHLS010000001.1
JAGHLS010000003.1
JAGHLS010000005.1
JAGHLS01000000

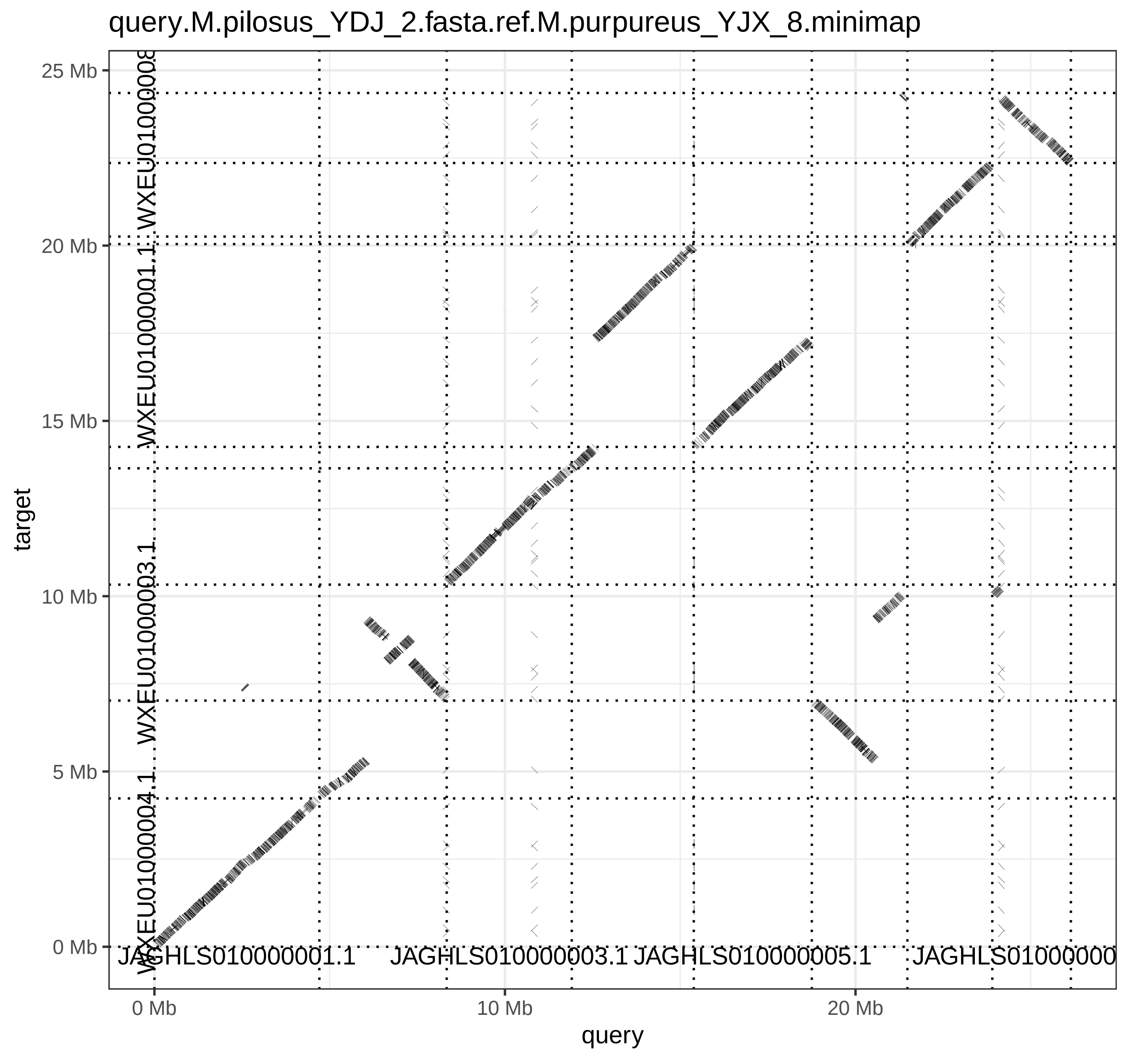
query.M.pilosus_YDJ_2.fasta.ref.M.purpureus_YJX_8.minimap
target
query
25 Mb
20 Mb
15 Mb
10 Mb
5 Mb
0 Mb
0 Mb
10 Mb
20 Mb
JAGHLS010000001.1
JAGHLS010000003.1
JAGHLS010000005.1
JAGHLS01000000
WXEU01000008
WXEU010000001.1
WXEU010000003.1
WXEU010000004.1

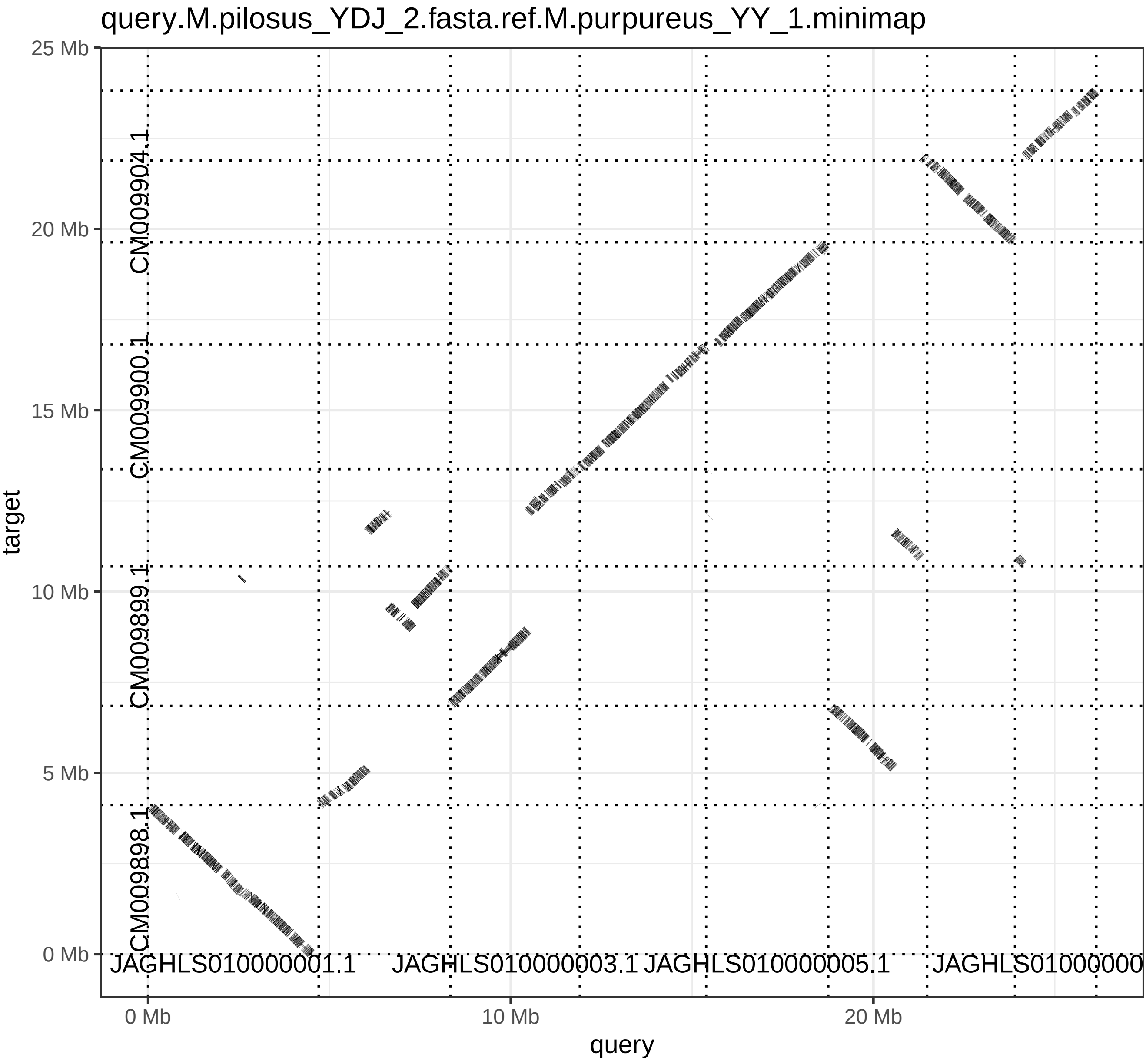
query.M.pilosus_YDJ_2.fasta.ref.M.purpureus_YY_1.minimap
25 Mb
20 Mb
15 Mb
10 Mb
5 Mb
0 Mb
target
CM009904.1
CM009900.1
CM009899.1
CM009898.1
JAGHLS010000001.1
JAGHLS010000003.1
JAGHLS010000005.1
JAGHLS01000000
0 Mb
10 Mb
20 Mb
query

query.M.pilosus_YDJ_2.fasta.ref.M.purpureus_YY_1_SIM.minimap

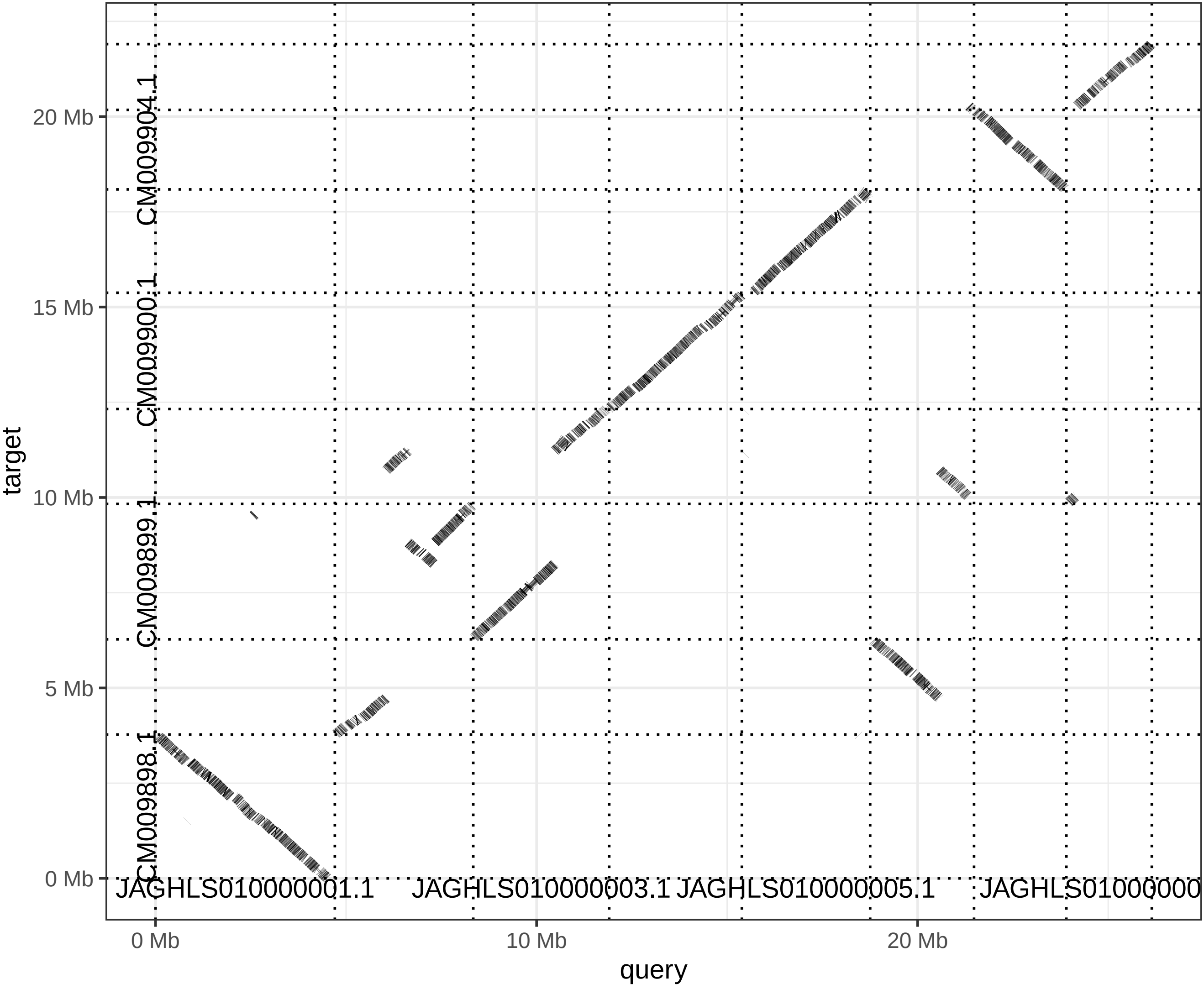

query.M.pilosus_YDJ_2.fasta.ref.M.ruber_FWB13.minimap

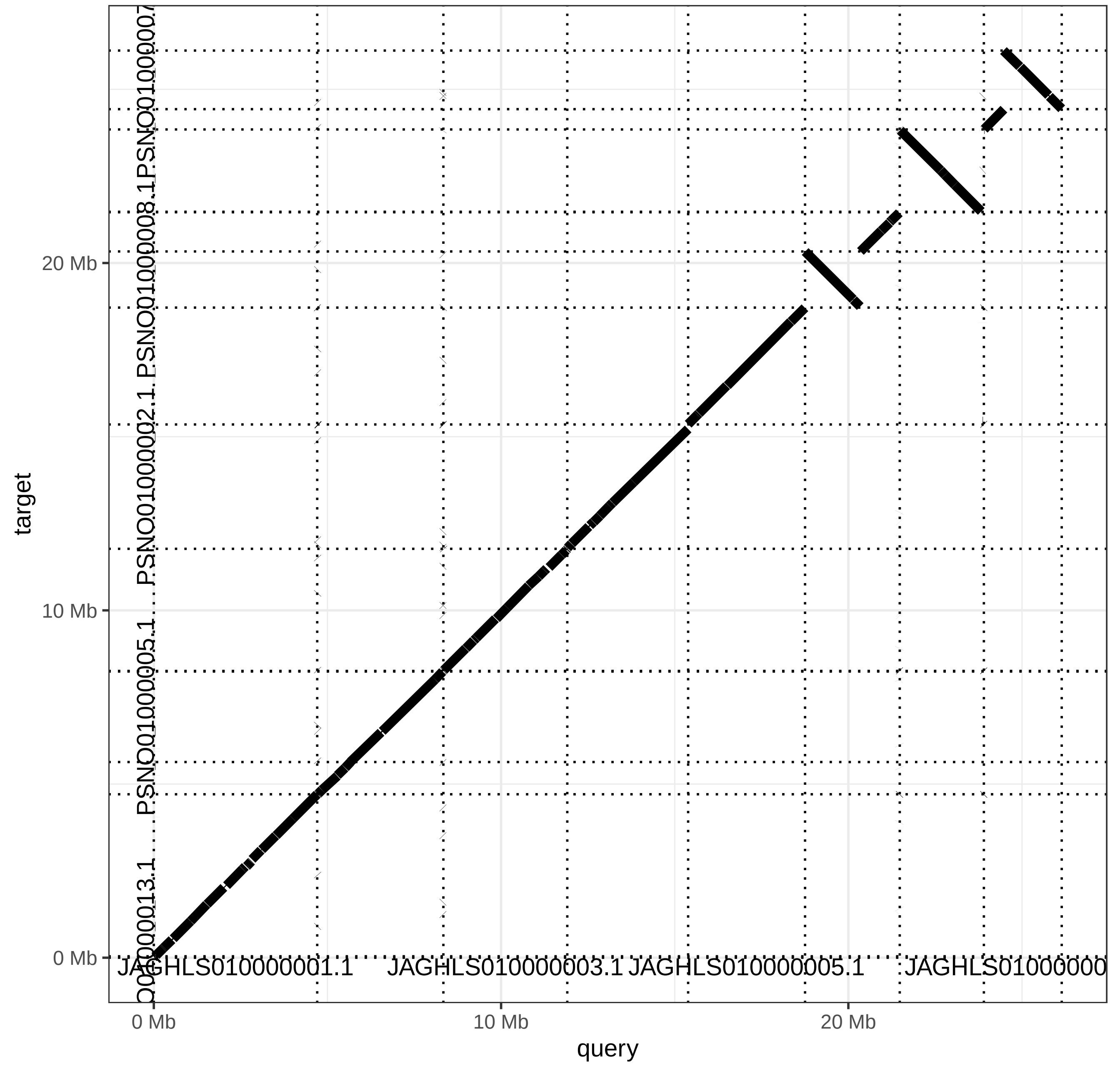

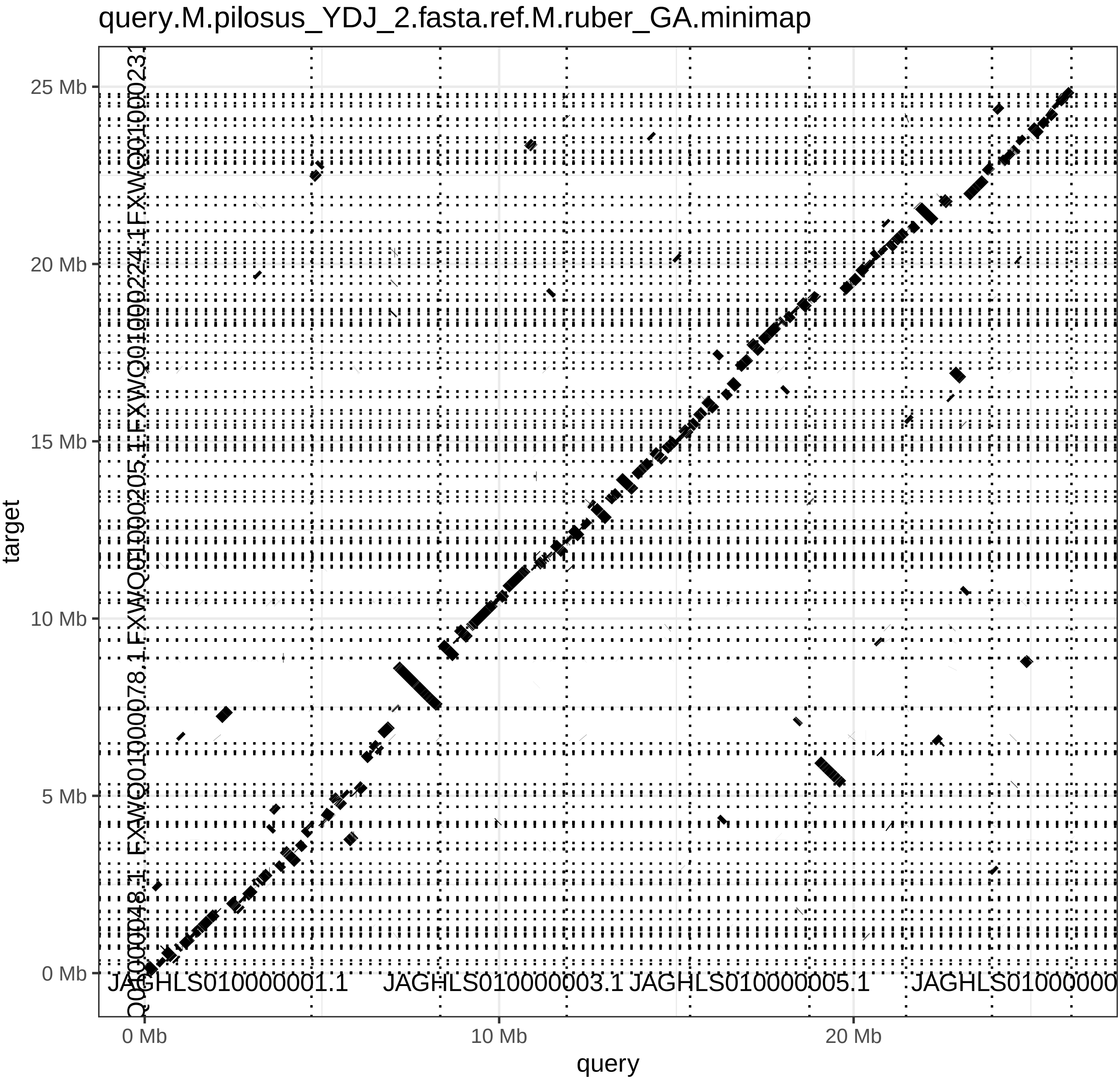

query.M.pilosus_YDJ_2.fasta.ref.M.ruber_GA.minimap
target
25 Mb
20 Mb
15 Mb
10 Mb
5 Mb
0 Mb
query
0 Mb
10 Mb
20 Mb
JAGHLS010000001.1
JAGHLS010000003.1
JAGHLS010000005.1

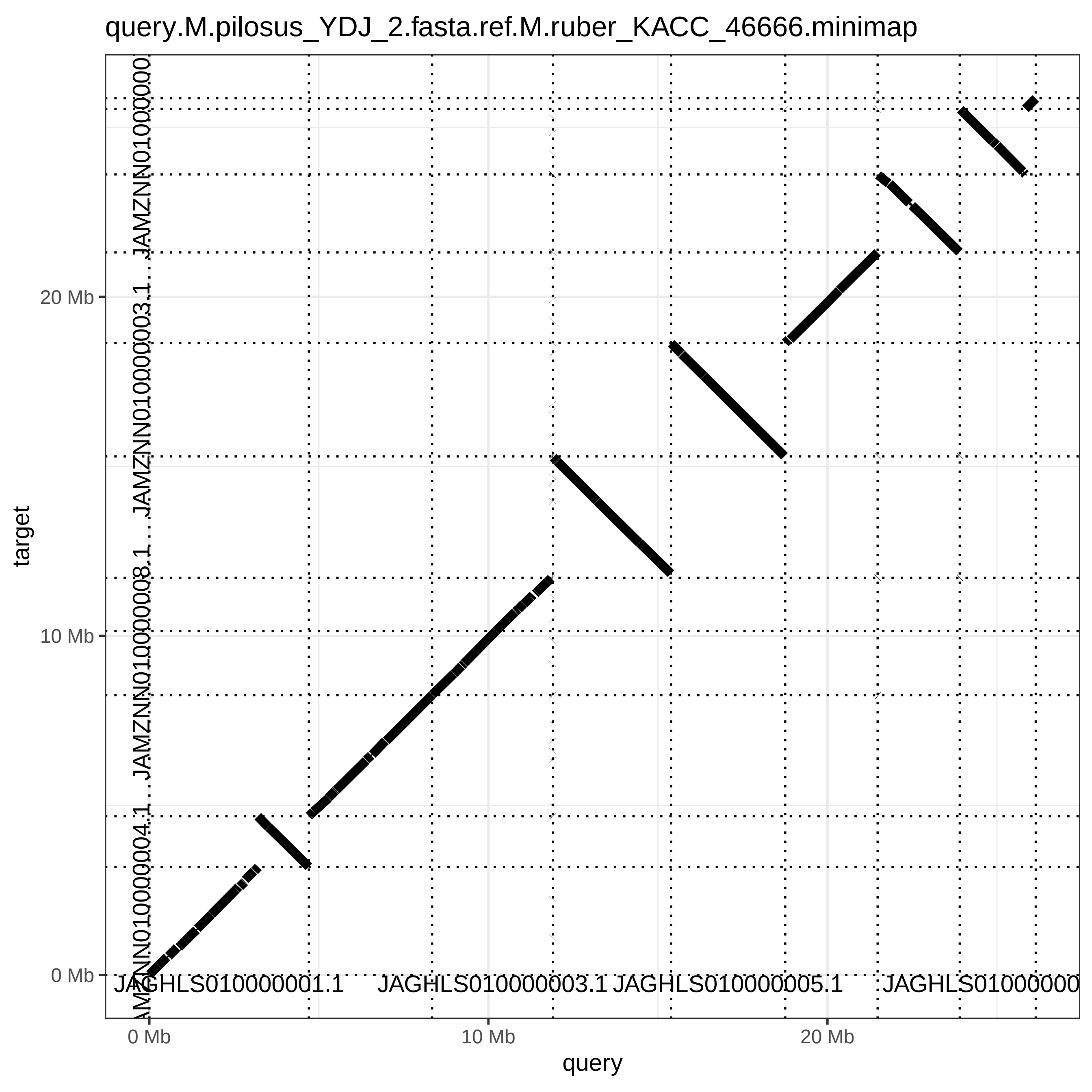

query.M.pilosus_YDJ_2.fasta.ref.M.ruber_KACC_46666.minimap
target
query
0 Mb
10 Mb
20 Mb
JAGHLS010000001.1
JAGHLS010000003.1
JAGHLS010000005.1
JAGHLS01000000
JAMZNN010000004.1
JAMZNN010000008.1
JAMZNN010000003.1
JAMZNN0100000

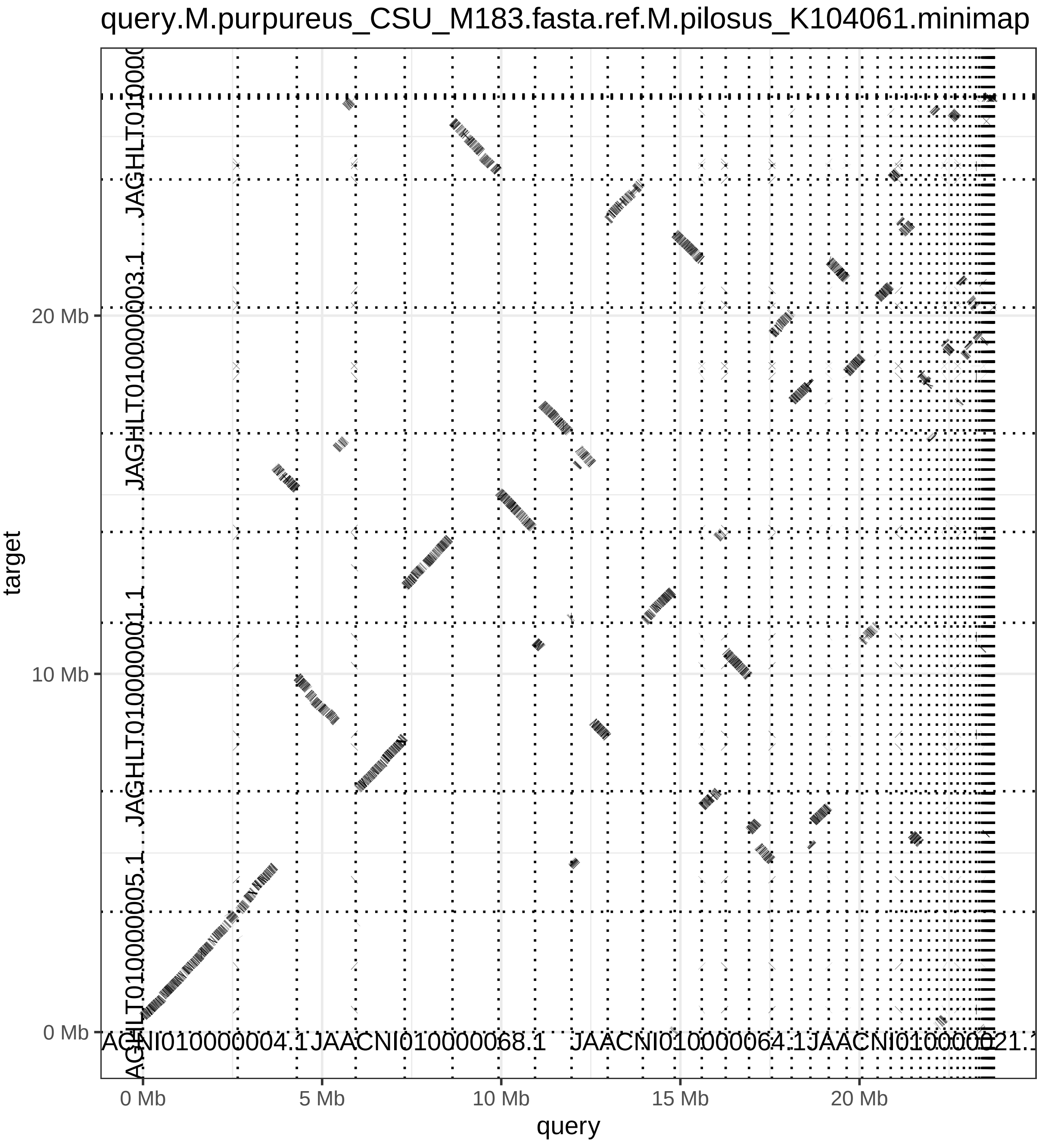
query.M.purpureus_CSU_M183.fasta.ref.M.pilosus_K104061.minimap
target
query
0 Mb
5 Mb
10 Mb
15 Mb
20 Mb
0 Mb
10 Mb
20 Mb
JAGHLT010000005.1
JAGHLT010000001.1
JAGHLT010000003.1
JAACNI010000004.1
JAACNI010000068.1
JAACNI010000064.1

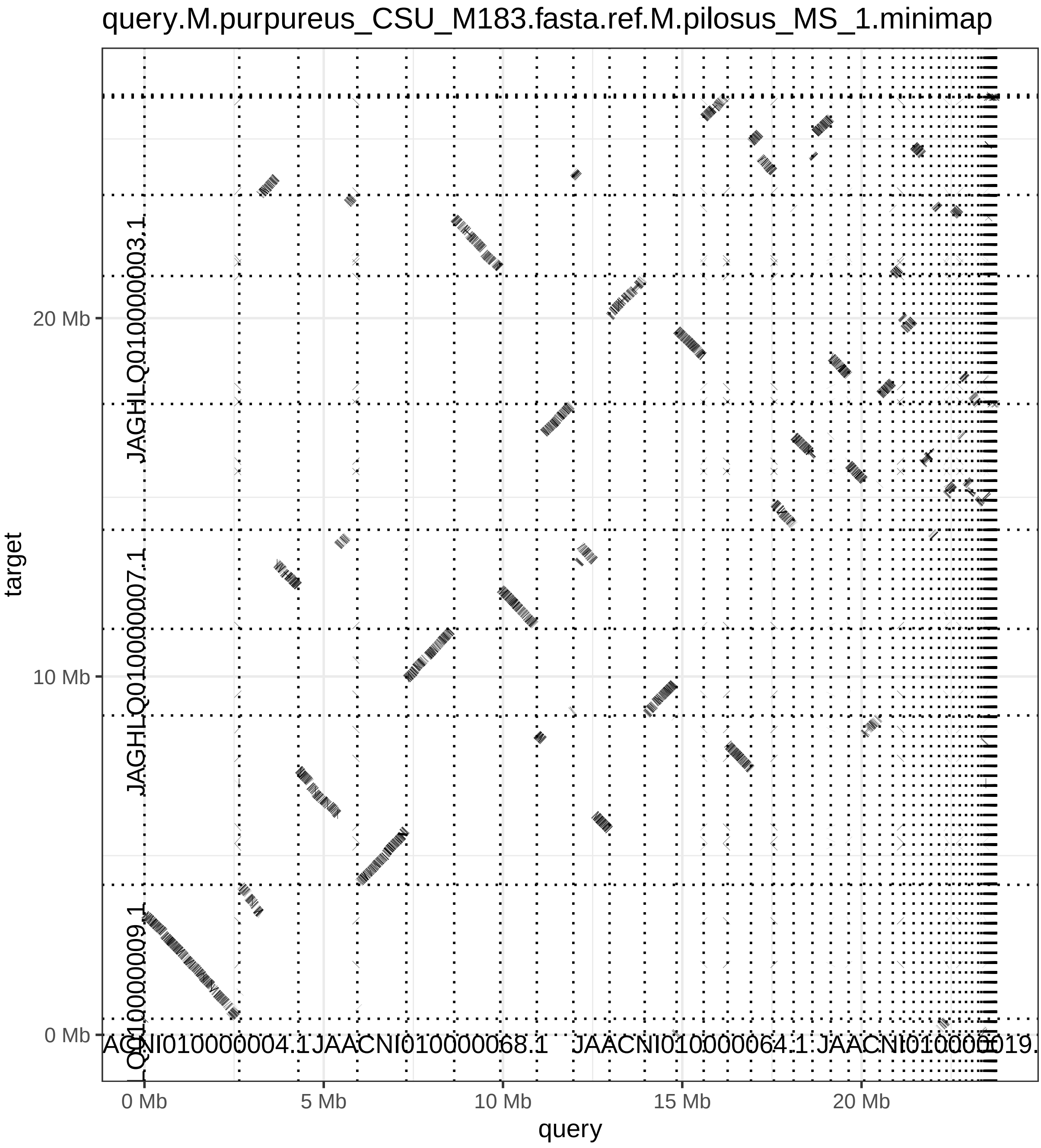
query.M.purpureus_CSU_M183.fasta.ref.M.pilosus_MS_1.minimap
target
20 Mb
10 Mb
0 Mb
JAGHLQ010000003.1
JAGHLQ010000007.1
LQ010000009.1
ACNI010000004.1
JAACNI010000068.1
JAACNI010000064.1
JAACNI01000019.
0 Mb
5 Mb
10 Mb
15 Mb
20 Mb
query

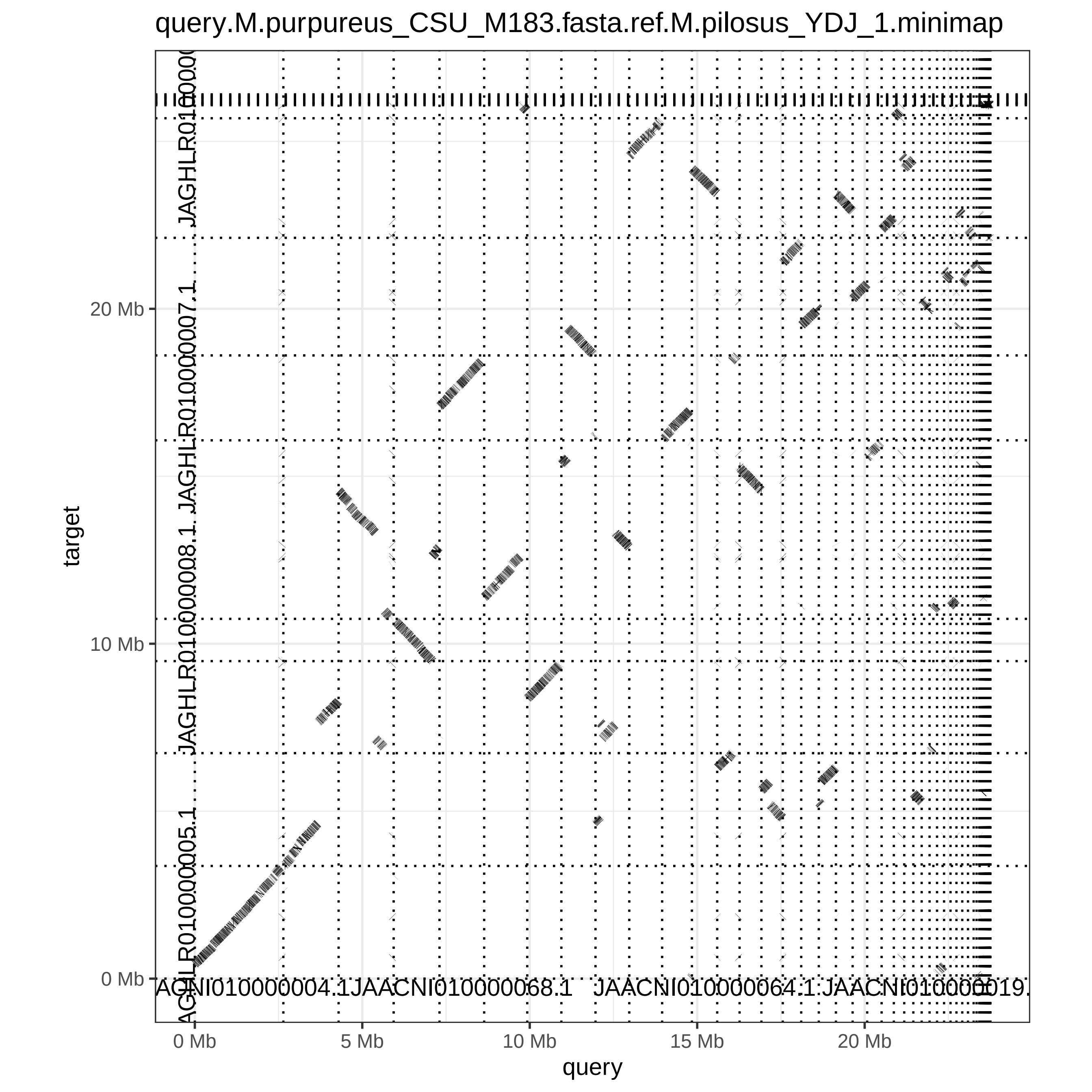
query.M.purpureus_CSU_M183.fasta.ref.M.pilosus_YDJ_1.minimap
target
query
0 Mb
5 Mb
10 Mb
15 Mb
20 Mb
0 Mb
10 Mb
20 Mb
JAGHLR010000007.1
JAGHLR010000008.1
JAGHLR010000005.1
JAACNI010000004.1
JAACNI010000068.1
JAACNI010000064.1

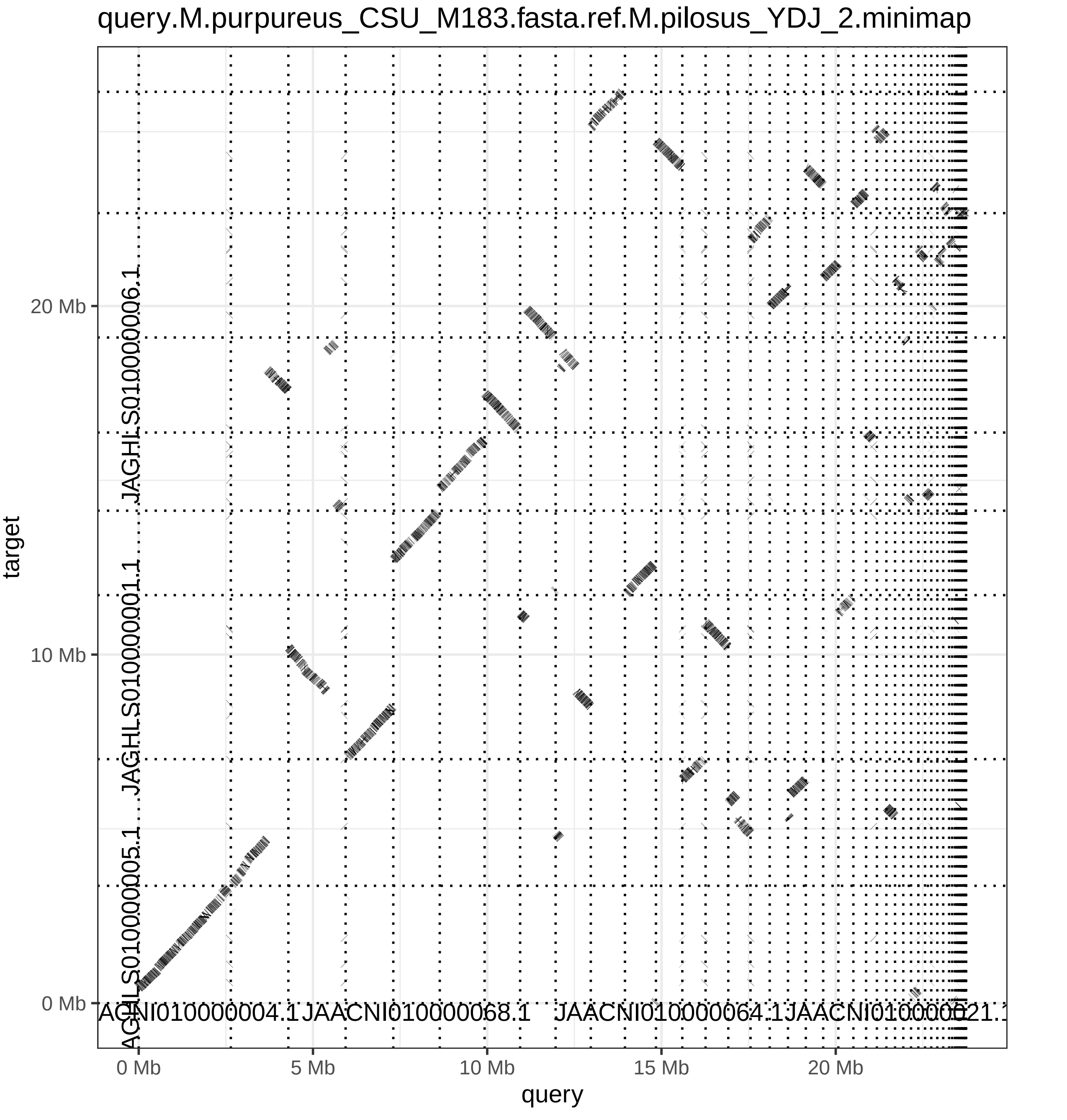
query.M.purpureus_CSU_M183.fasta.ref.M.pilosus_YDJ_2.minimap
target
query
0 Mb
10 Mb
20 Mb
0 Mb
5 Mb
10 Mb
15 Mb
20 Mb
JAGHLS010000006.1
JAGHLS010000001.1
JAGHLS010000005.1
JAACNI010000004.1
JAACNI010000068.1
JAACNI010000064.1
JAACNI010000021.1

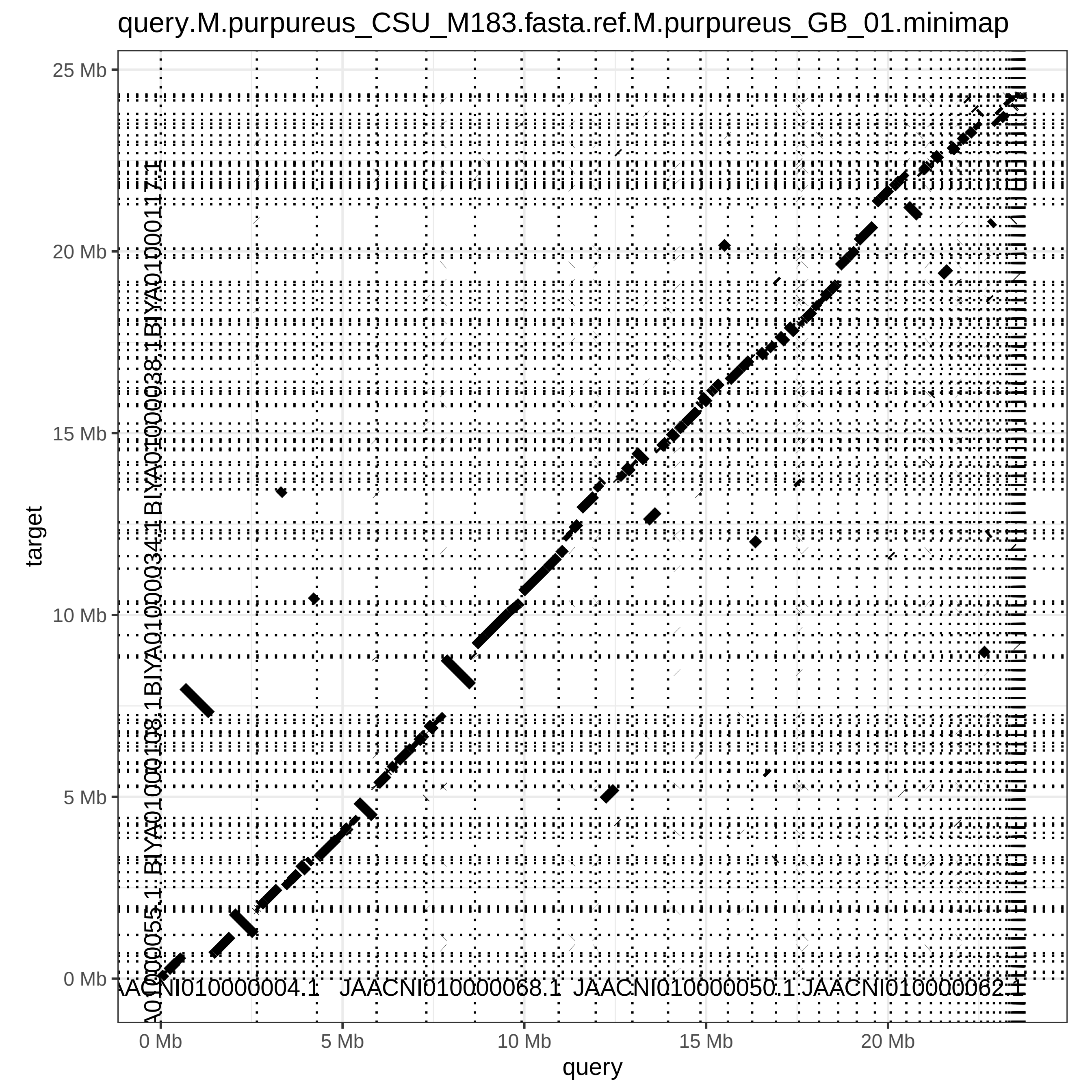

query.M.purpureus_CSU_M183.fasta.ref.M.purpureus_GB_01.minimap
target
query
25 Mb
20 Mb
15 Mb
10 Mb
5 Mb
0 Mb
0 Mb
5 Mb
10 Mb
15 Mb
20 Mb
JAACNI010000068.1
JAACNI010000062.1

query.M.purpureus_CSU_M183.fasta.ref.M.purpureus_HQ1.minimap

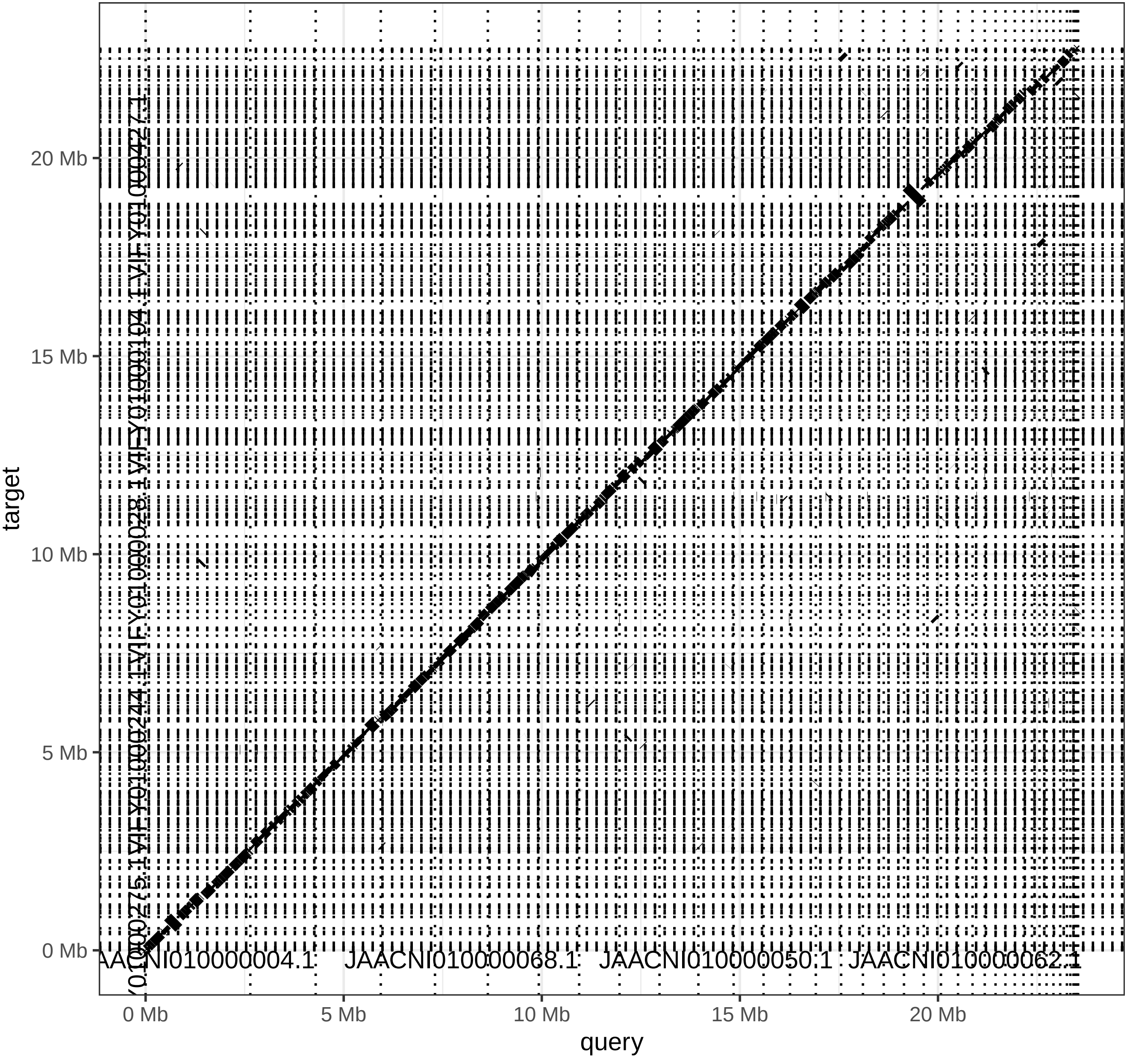

query.M.purpureus_CSU_M183.fasta.ref.M.purpureus_P7048X_2.minimap

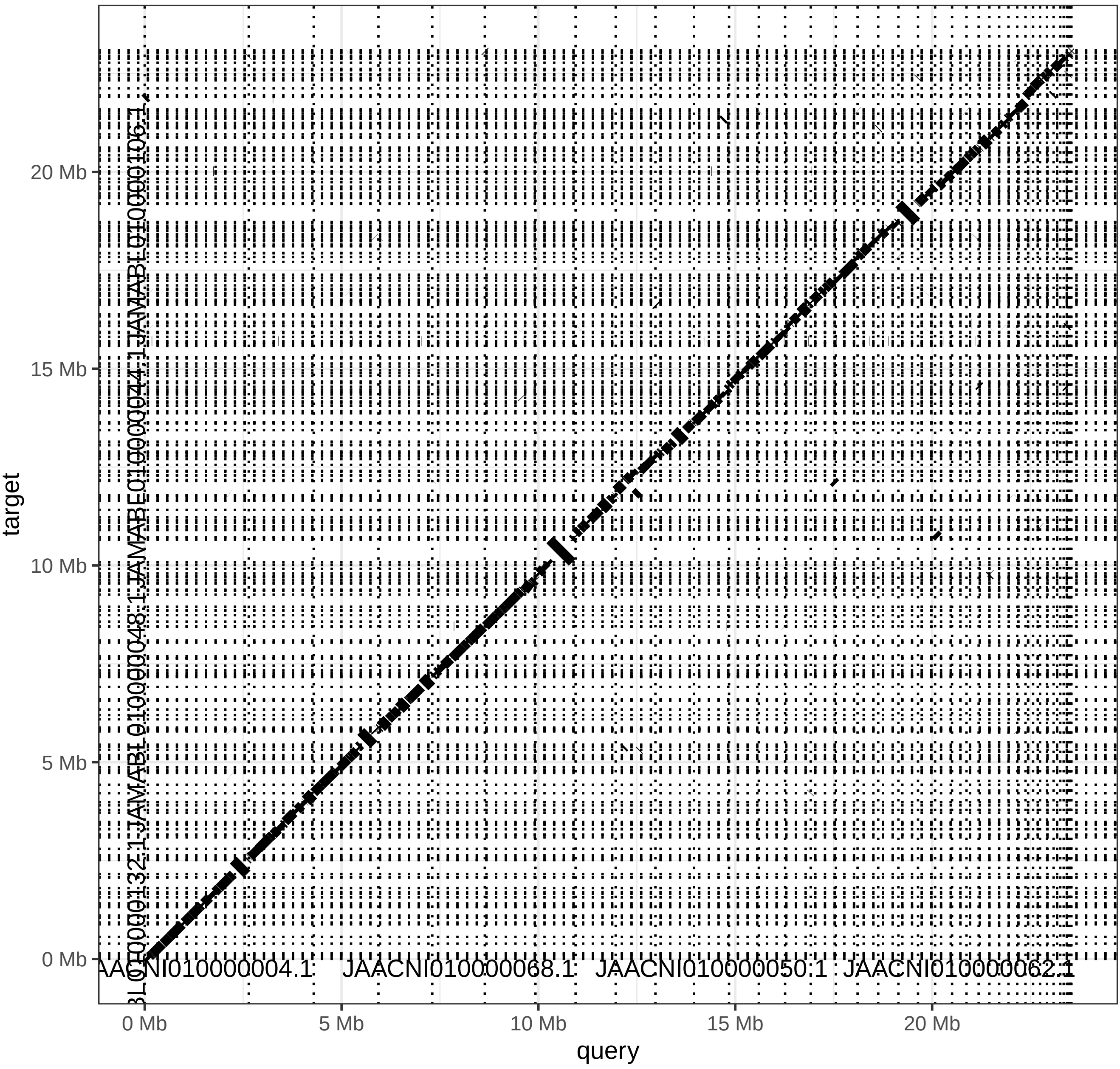

query.M.purpureus_CSU_M183.fasta.ref.M.purpureus_PF1702S.minimap

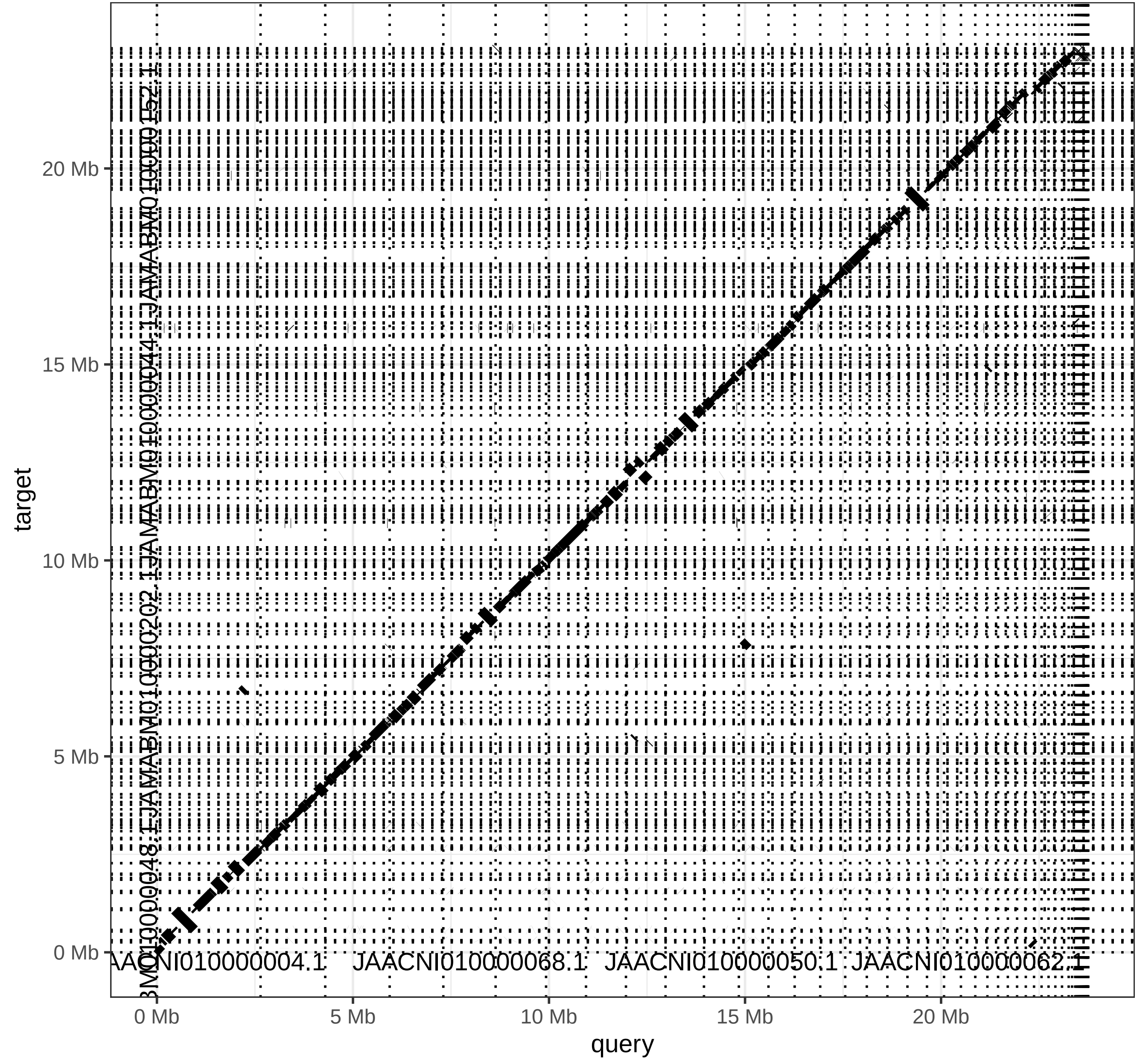

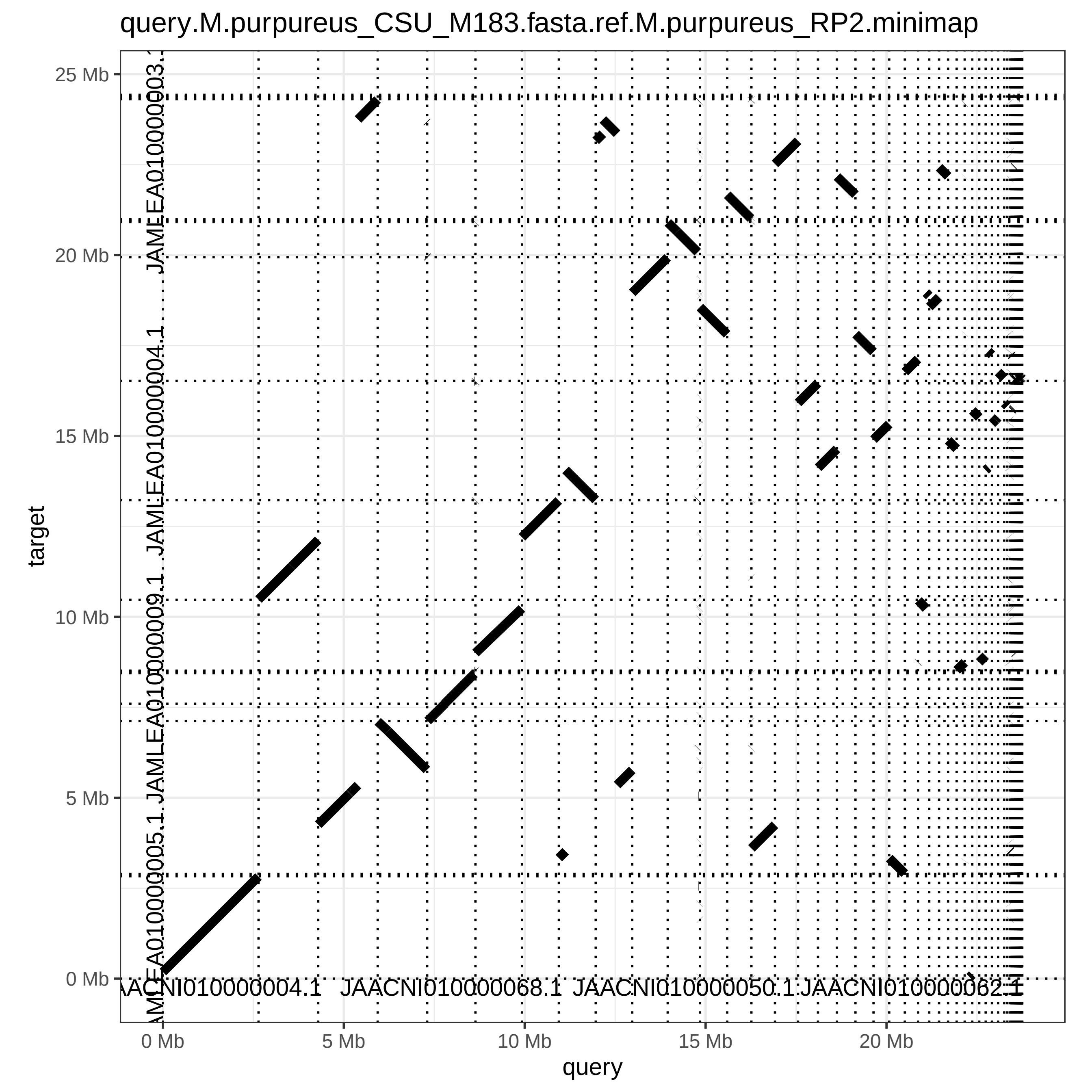

query.M.purpureus_CSU_M183.fasta.ref.M.purpureus_RP2.minimap
target
query
25 Mb
20 Mb
15 Mb
10 Mb
5 Mb
0 Mb
0 Mb
5 Mb
10 Mb
15 Mb
20 Mb
JAMLEA010000003.1
JAMLEA010000004.1
JAMLEA010000009.1
JAMLEA010000005.1
JAACNI010000004.1
JAACNI010000068.1
JAACNI010000050.1
JAACNI010000062.1

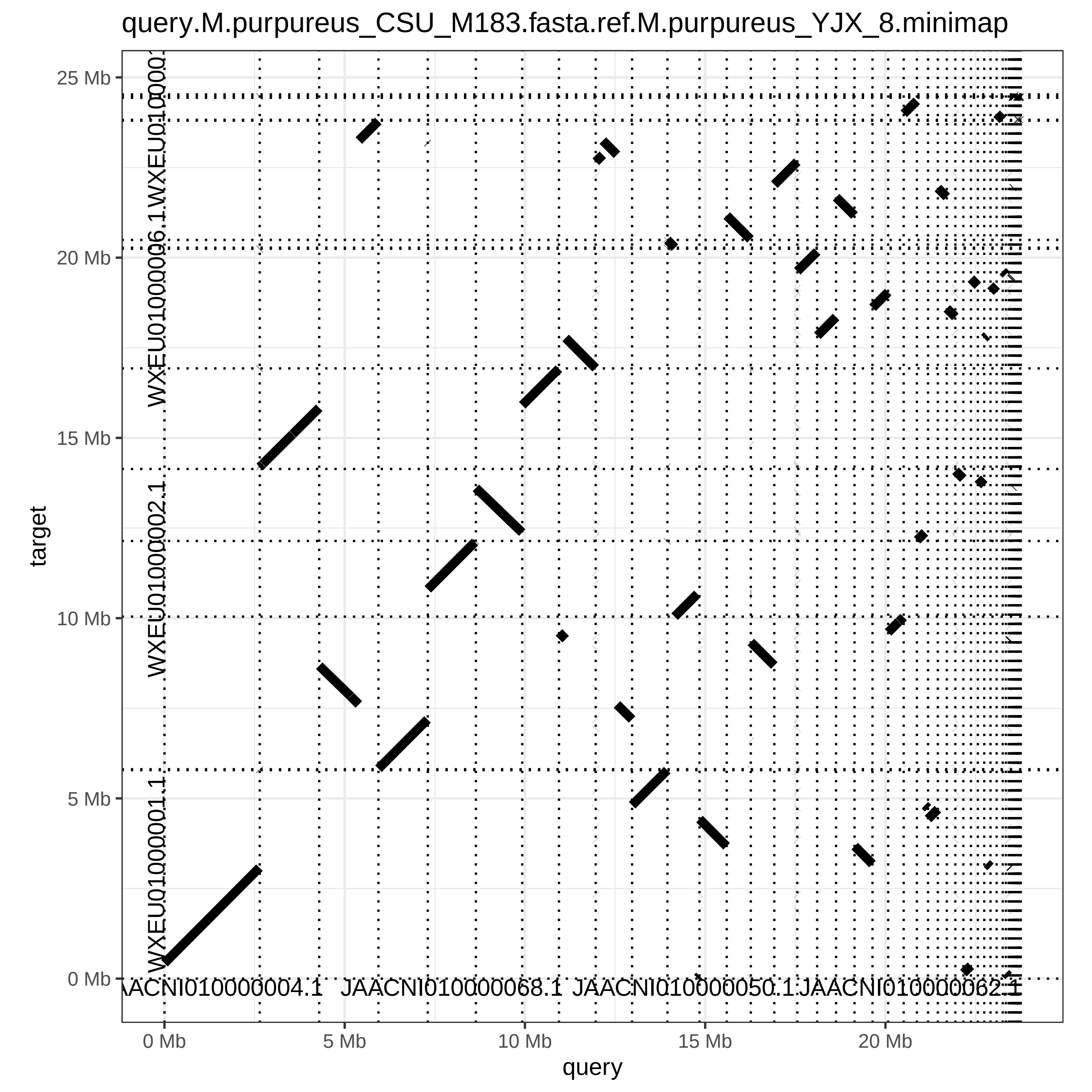
query.M.purpureus_CSU_M183.fasta.ref.M.purpureus_YJX_8.minimap
target
25 Mb
20 Mb
15 Mb
10 Mb
5 Mb
0 Mb
WXEU01000001.1
WXEU01000002.1
0 Mb
5 Mb
10 Mb
15 Mb
20 Mb
JAACNI010000068.1
JAACNI010000062.1
query

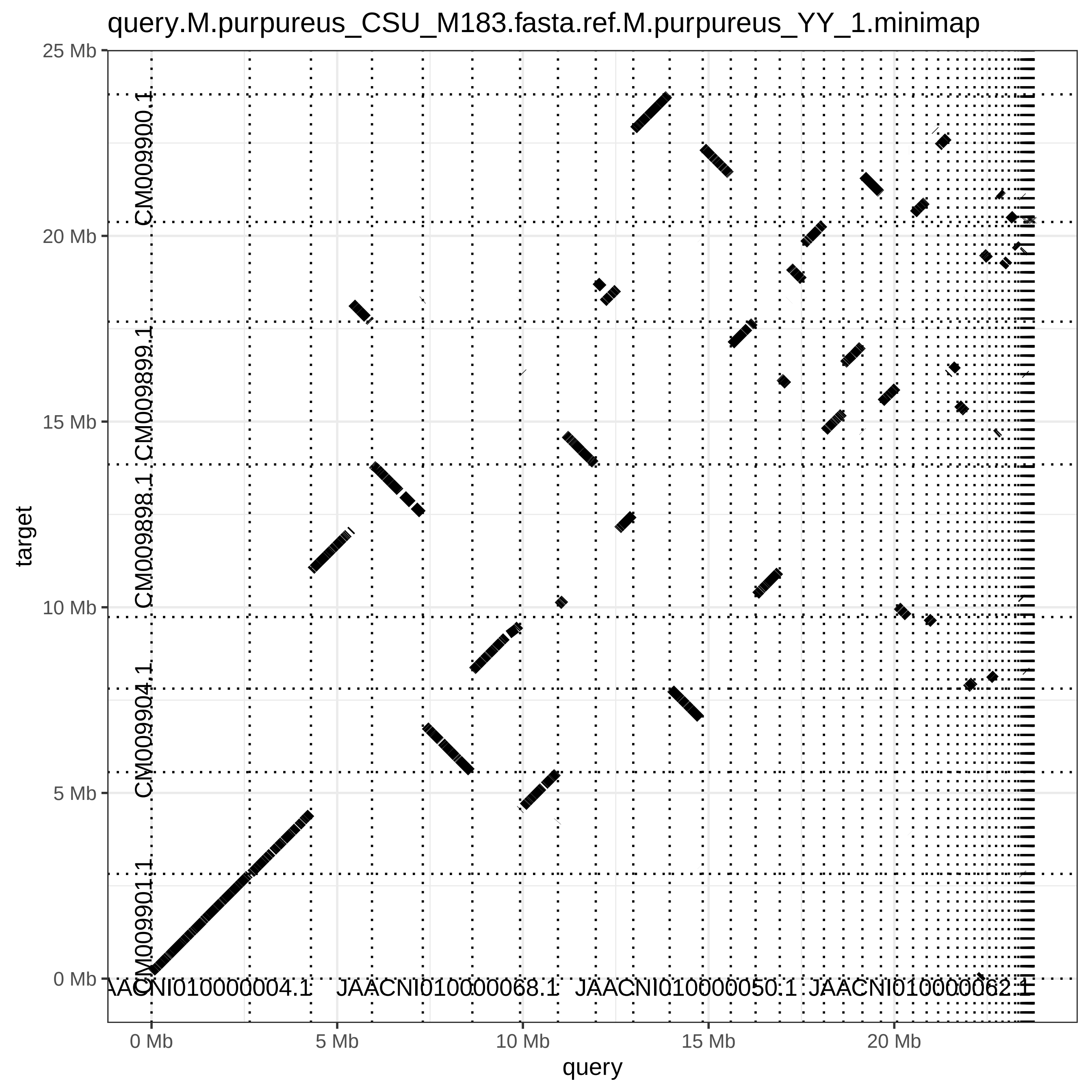
query.M.purpureus_CSU_M183.fasta.ref.M.purpureus_YY_1.minimap
target
query
25 Mb
20 Mb
15 Mb
10 Mb
5 Mb
0 Mb
0 Mb
5 Mb
10 Mb
15 Mb
20 Mb
CM009900.1
CM009899.1
CM009898.1
CM009904.1
CM009901.1
JAACNI010000068.1
JAACNI010000050.1
JAACNI010000062.1

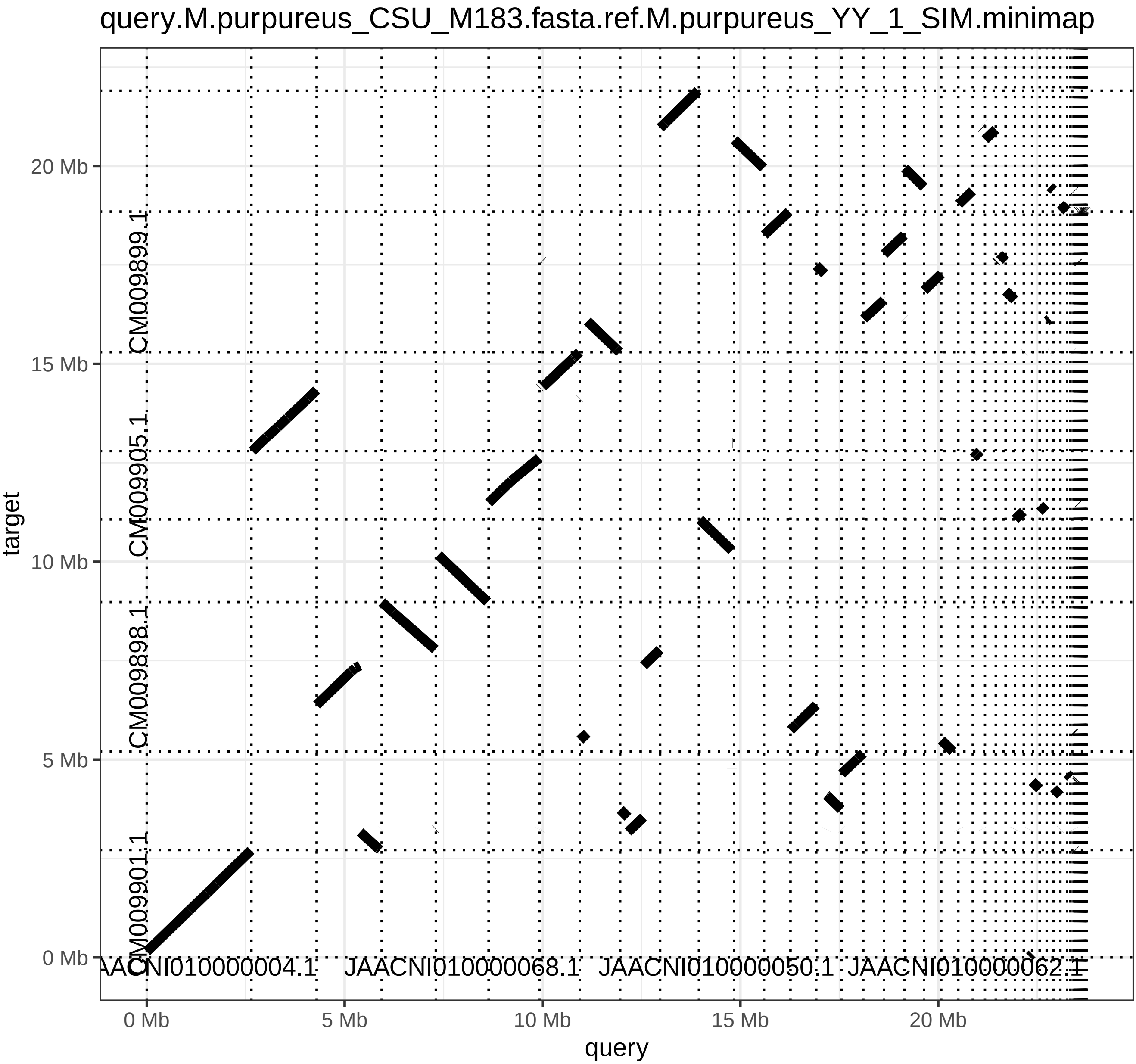
query.M.purpureus_CSU_M183.fasta.ref.M.purpureus_YY_1_SIM.minimap
target
20 Mb
15 Mb
10 Mb
5 Mb
0 Mb
CM009899.1
CM009905.1
CM009898.1
CM009901.1
JAACNI010000004.1
JAACNI010000068.1
JAACNI010000050.1
JAACNI010000062.1
0 Mb
5 Mb
10 Mb
15 Mb
20 Mb
query

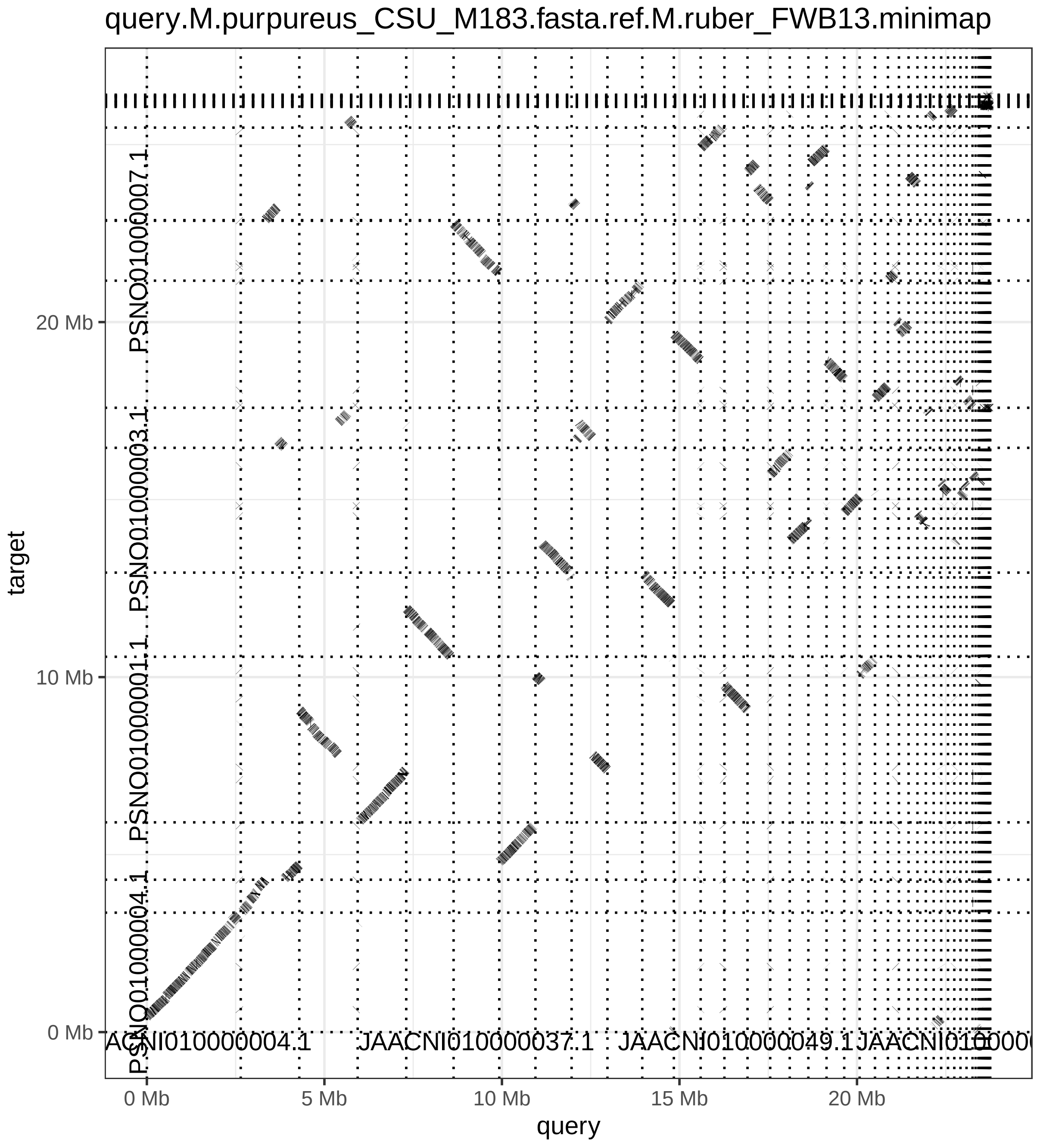
query.M.purpureus_CSU_M183.fasta.ref.M.ruber_FWB13.minimap
target
query
20 Mb
10 Mb
0 Mb
0 Mb
5 Mb
10 Mb
15 Mb
20 Mb
PSNO01000007.1
PSNO01000003.1
PSNO01000001.1
PSNO01000004.1
JAACNI010000037.1
JAACNI010000049.1

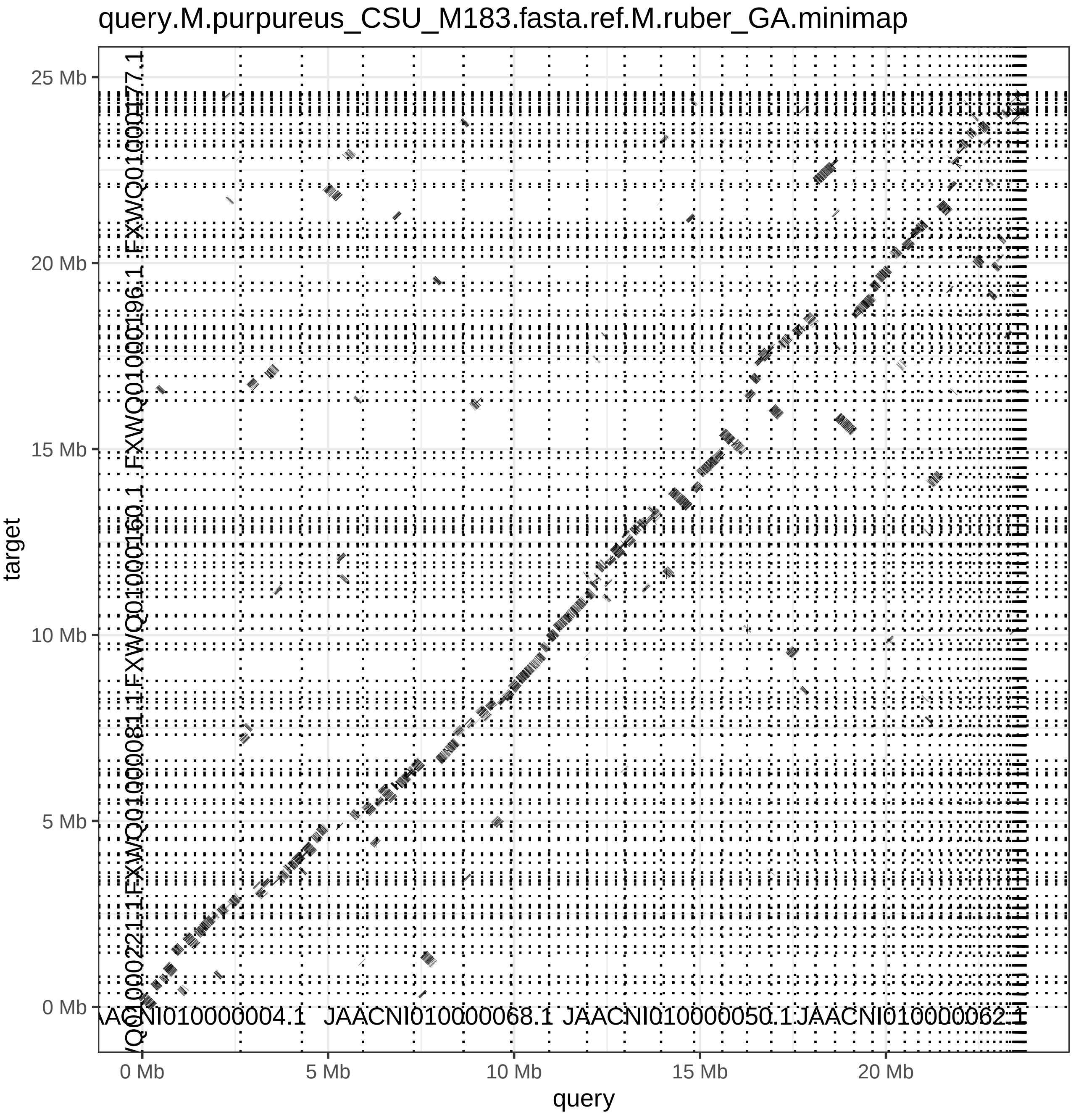
query.M.purpureus_CSU_M183.fasta.ref.M.ruber_GA.minimap
target
query
0 Mb
5 Mb
10 Mb
15 Mb
20 Mb
25 Mb
JAACNI010000068.1
JAACNI010000050.1
JAACNI010000062.1
FXWQ01000177.1
FXWQ01000196.1
FXWQ01000160.1

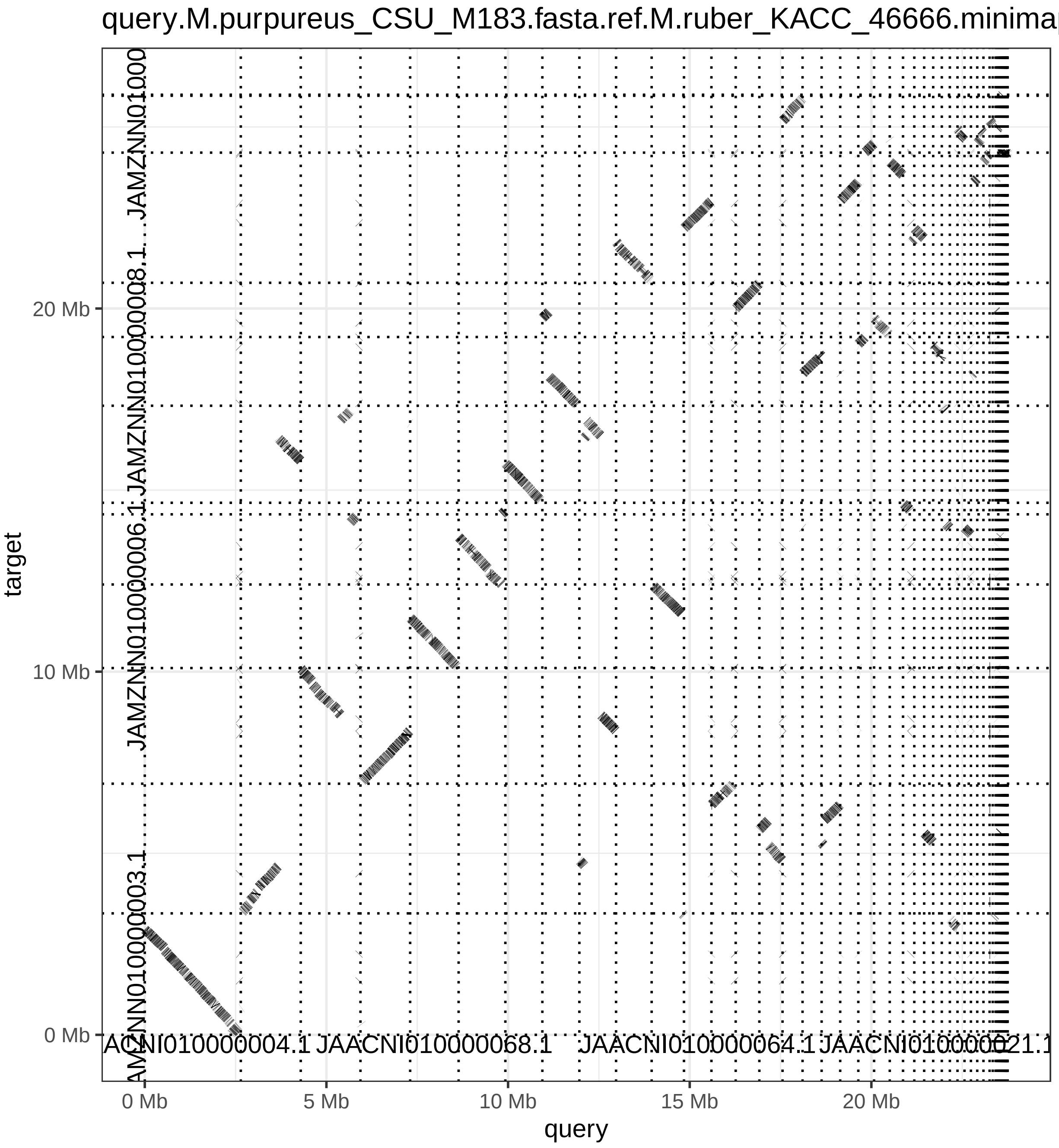

query.M.purpureus_CSU_M183.fasta.ref.M.ruber_KACC_46666.minimap
target
20 Mb
10 Mb
0 Mb
JAMZNN010000006.1
JAMZNN010000008.1
ACNI010000004.1
JAACNI010000068.1
JAACNI010000064.1
0 Mb
5 Mb
10 Mb
15 Mb
20 Mb
query

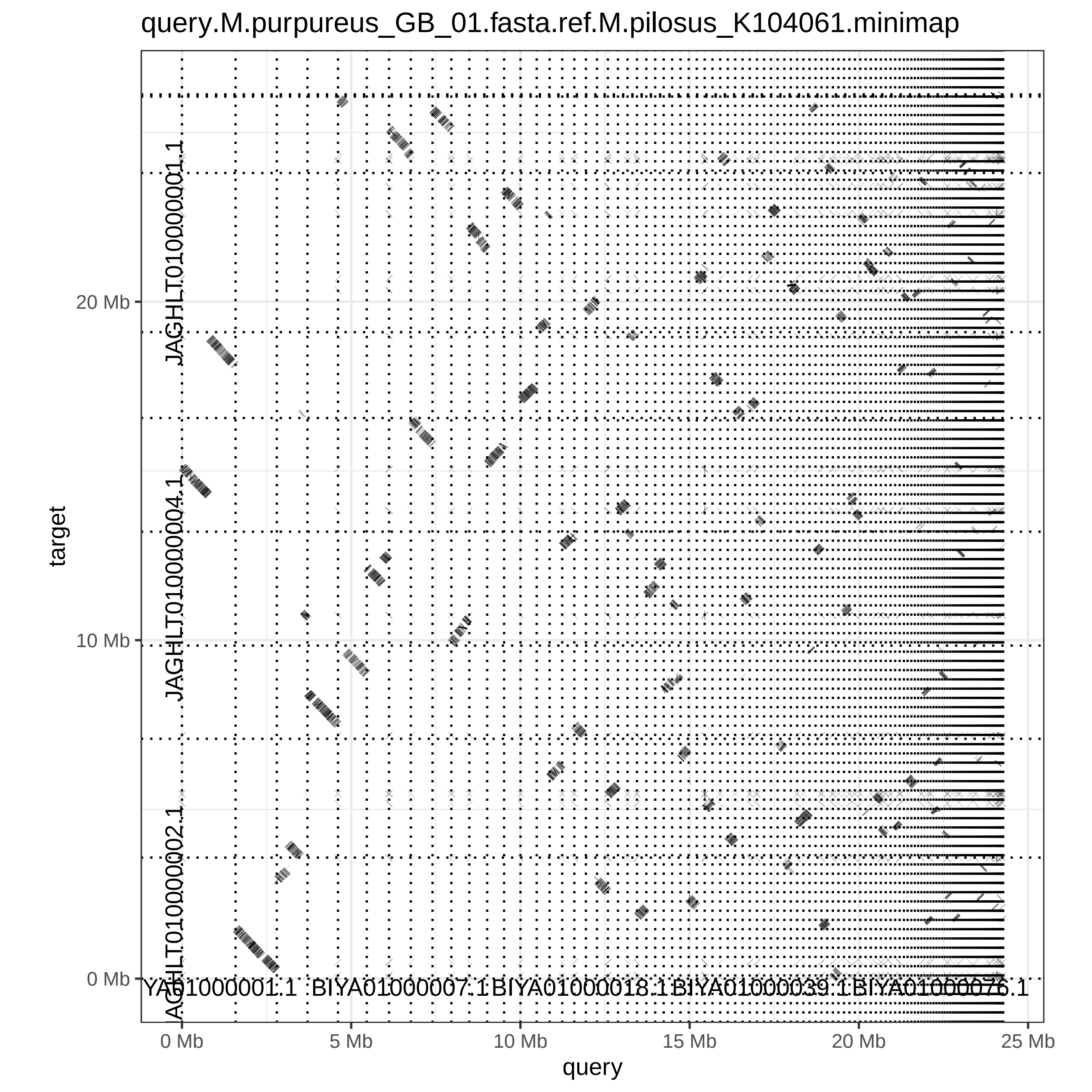

query.M.purpureus_GB_01.fasta.ref.M.pilosus_K104061.minimap
target
JAGHLT010000001.1
JAGHLT010000004.1
JAGHLT010000002.1
20 Mb
10 Mb
0 Mb
YA01000001.1
BIYA01000007.1
BIYA01000018.1
BIYA01000039.1
BIYA01000076.1
0 Mb
5 Mb
10 Mb
15 Mb
20 Mb
25 Mb
query

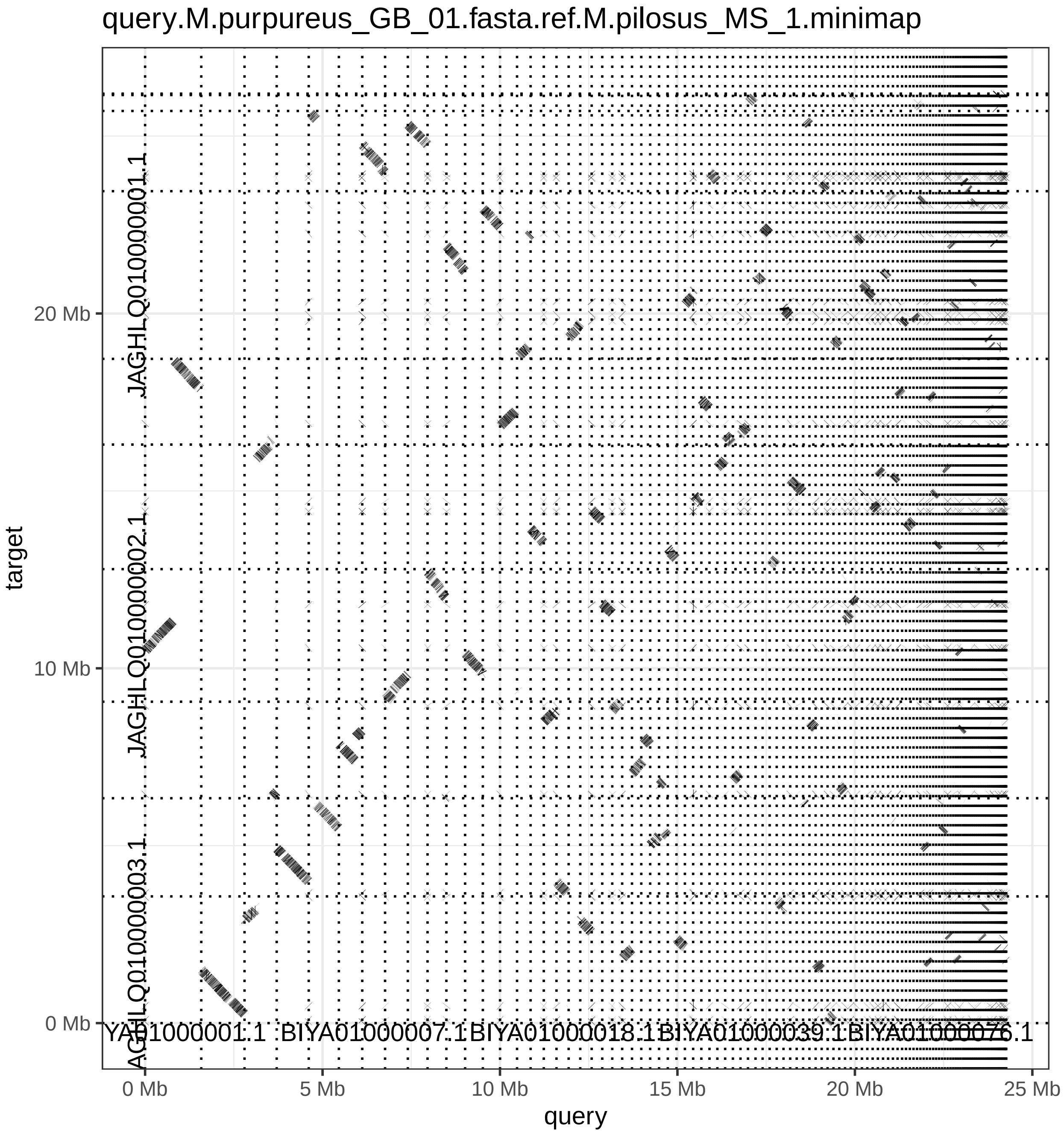
query.M.purpureus_GB_01.fasta.ref.M.pilosus_MS_1.minimap
target
20 Mb
10 Mb
0 Mb
JAGHLQ010000001.1
JAGHLQ010000002.1
JAGHLQ010000003.1
YA01000001.1
BIYA01000007.1
BIYA01000018.1
BIYA01000039.1
BIYA01000076.1
0 Mb
5 Mb
10 Mb
15 Mb
20 Mb
25 Mb
query

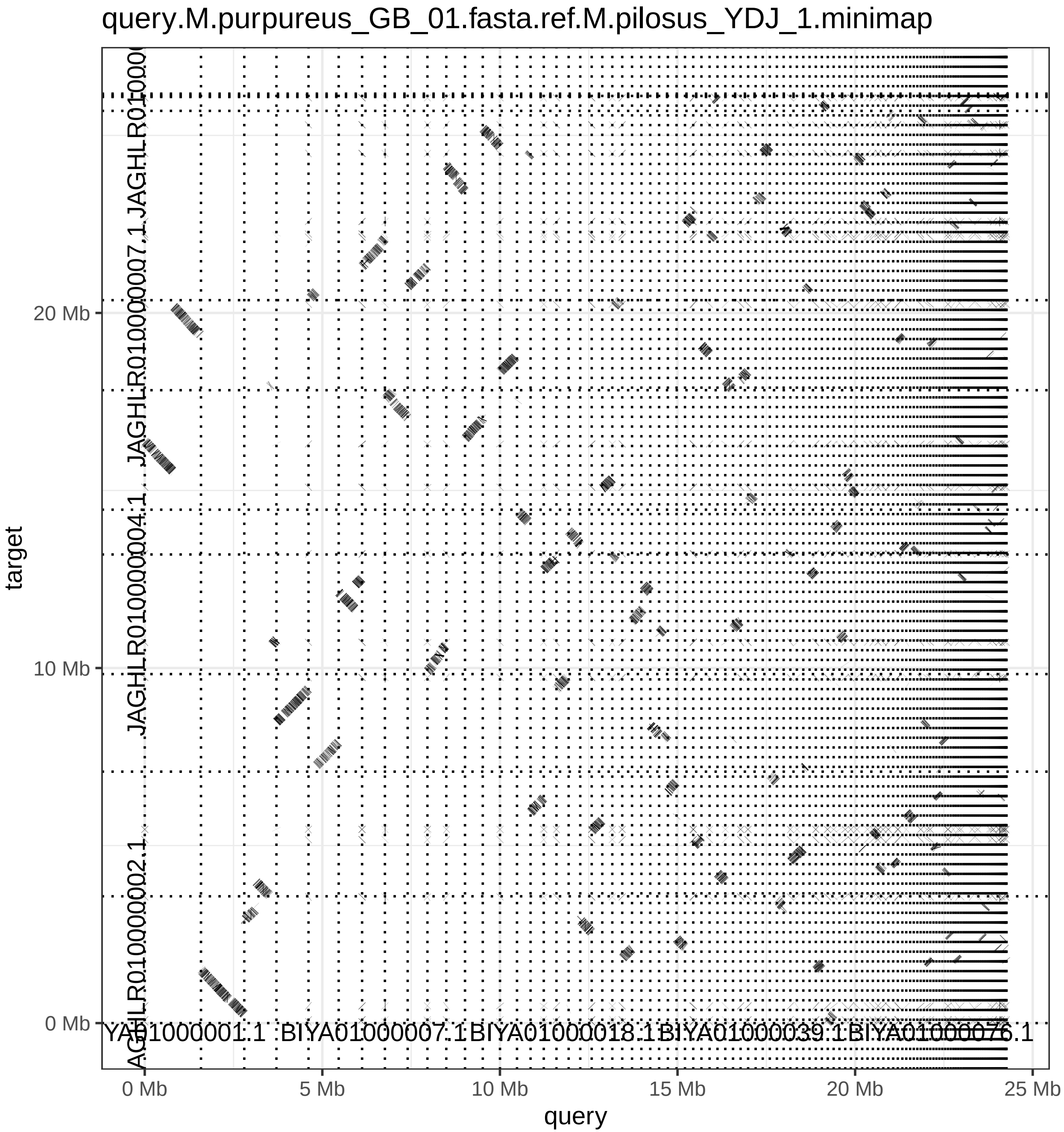

query.M.purpureus_GB_01.fasta.ref.M.pilosus_YDJ_1.minimap
target
query
20 Mb
10 Mb
0 Mb
0 Mb
5 Mb
10 Mb
15 Mb
20 Mb
25 Mb
YA01000001.1
BIYA01000007.1
BIYA01000018.1
BIYA01000039.1
BIYA01000076.1
JAGHLR010000007.1
JAGHLR010000004.1
JAGHLR010000002.1

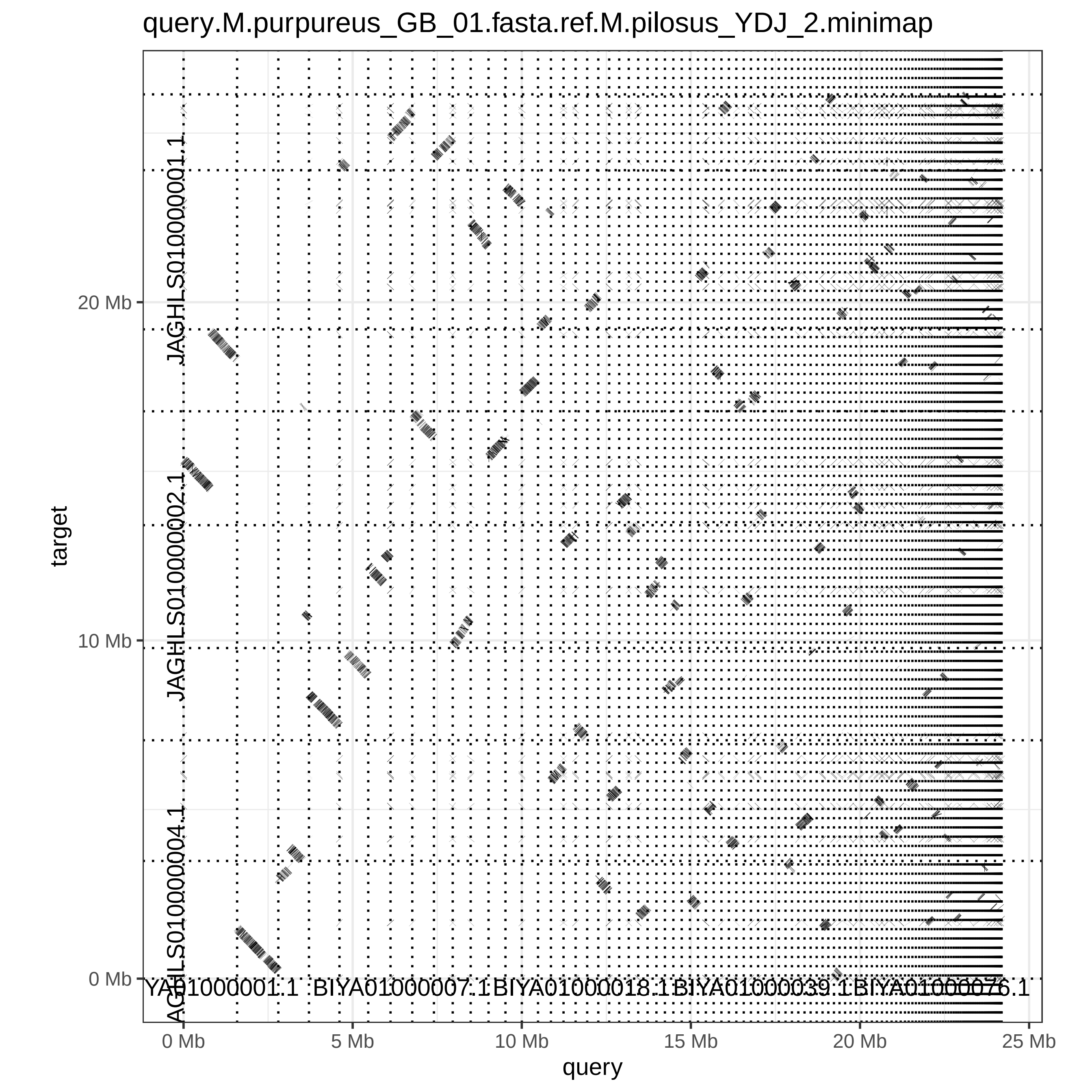

query.M.purpureus_GB_01.fasta.ref.M.pilosus_YDJ_2.minimap
target
JAGHLS010000001.1
JAGHLS010000002.1
JAGHLS010000004.1
20 Mb
10 Mb
0 Mb
YA01000001.1
BIYA01000007.1
BIYA01000018.1
BIYA01000039.1
BIYA01000076.1
0 Mb
5 Mb
10 Mb
15 Mb
20 Mb
25 Mb
query

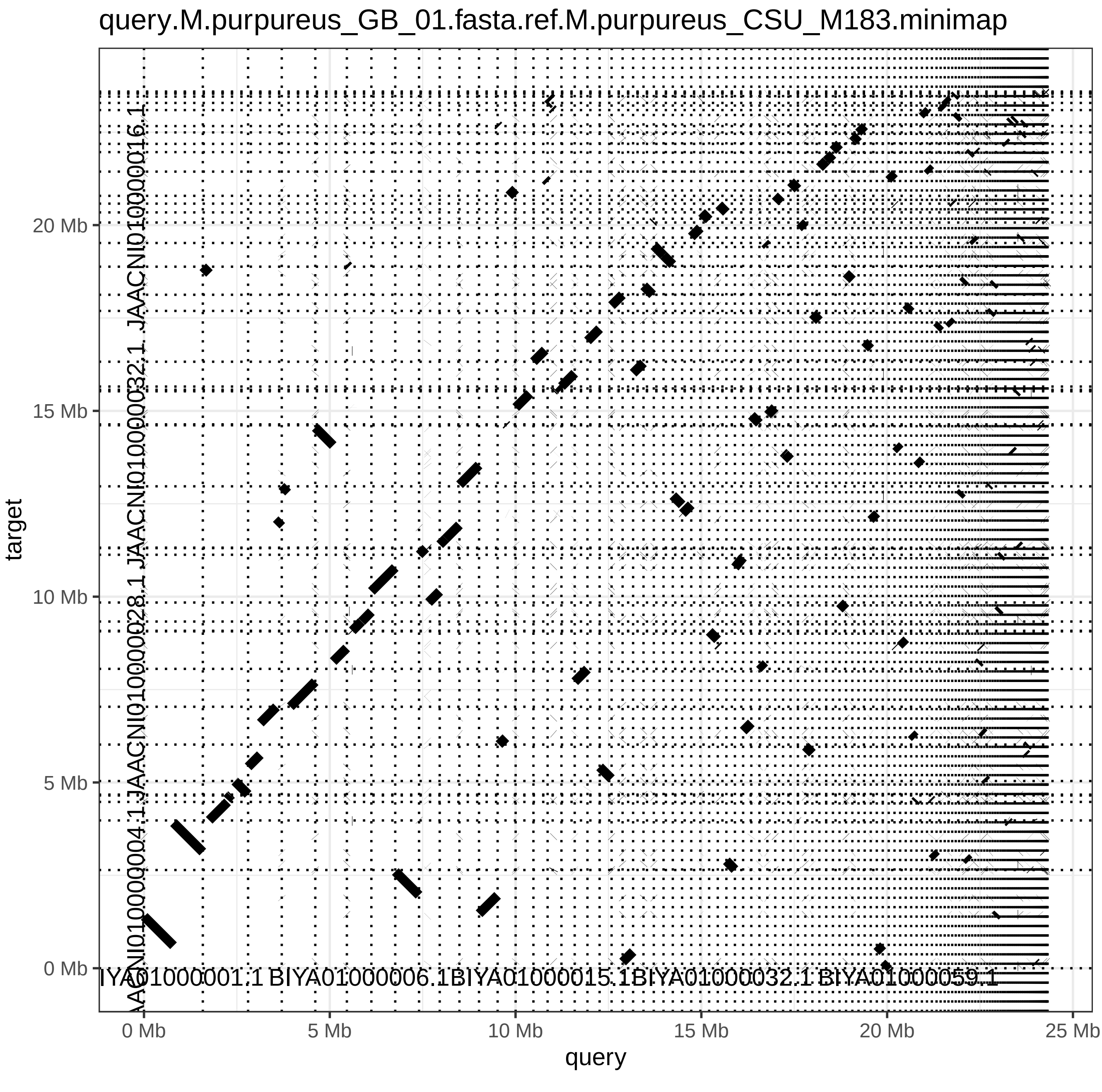
query.M.purpureus_GB_01.fasta.ref.M.purpureus_CSU_M183.minimap
target
20 Mb
15 Mb
10 Mb
5 Mb
0 Mb
JAACNI01000004.1
JAACNI01000028.1
JAACNI01000032.1
JAACNI01000016.1
BIYA01000006.1
BIYA01000015.1
BIYA01000032.1
BIYA01000059.1
0 Mb
5 Mb
10 Mb
15 Mb
20 Mb
25 Mb
query

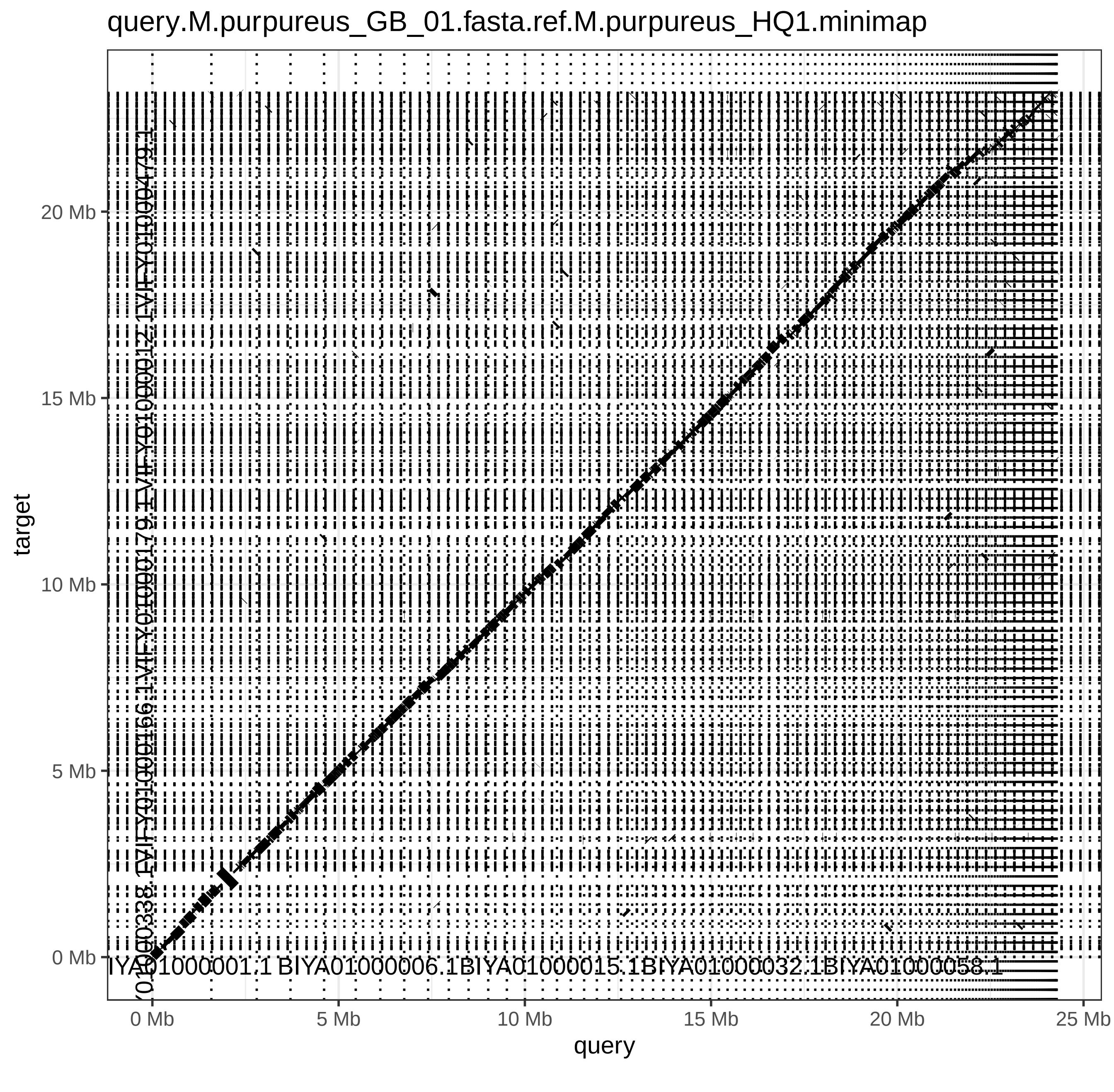
query.M.purpureus_GB_01.fasta.ref.M.purpureus_HQ1.minimap
target
0 Mb
5 Mb
10 Mb
15 Mb
20 Mb
25 Mb
query
BIYA01000006.1
BIYA01000015.1
BIYA01000032.1
BIYA01000058.1

query.M.purpureus_GB_01.fasta.ref.M.purpureus_P7048X_2.minimap

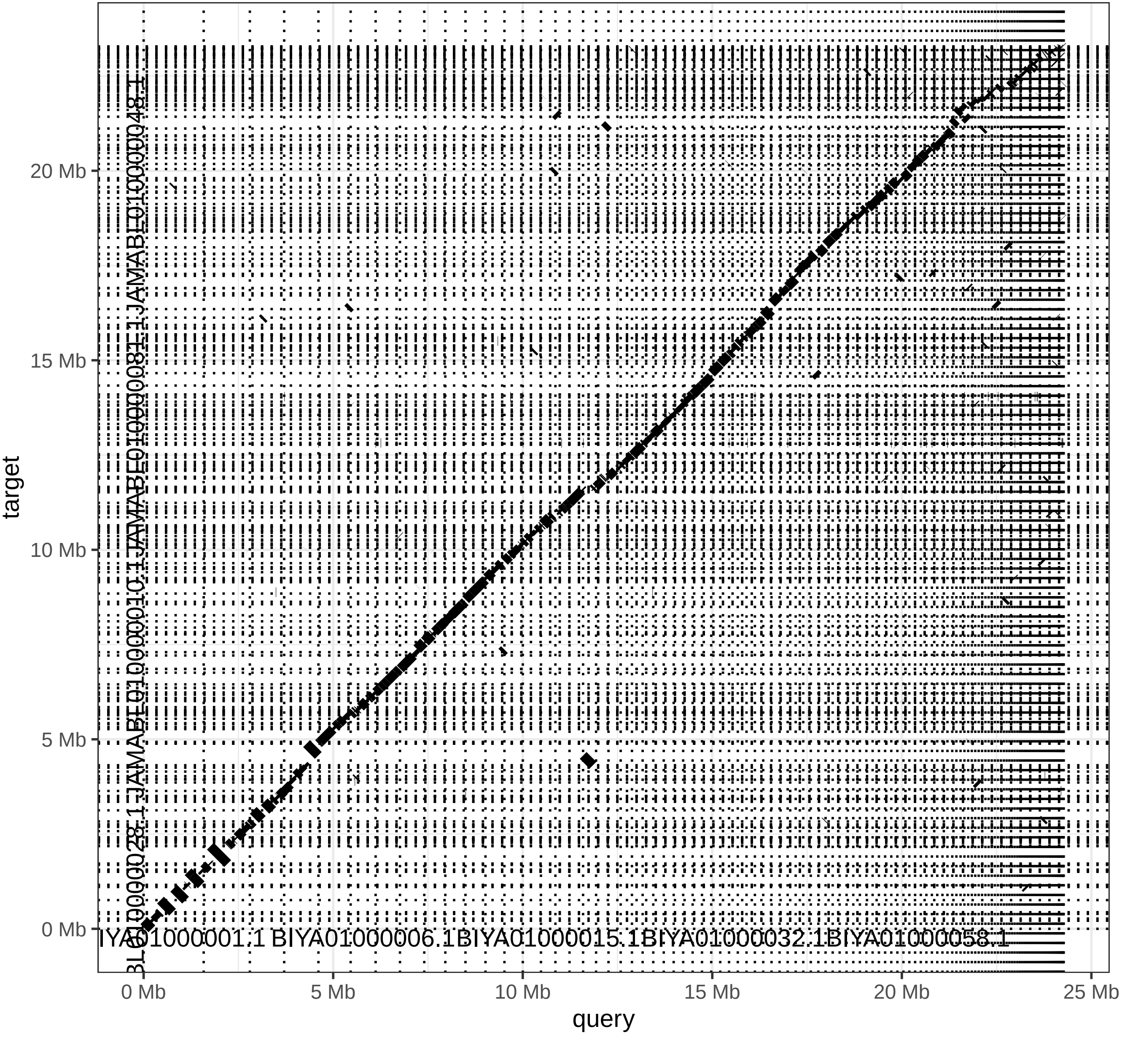

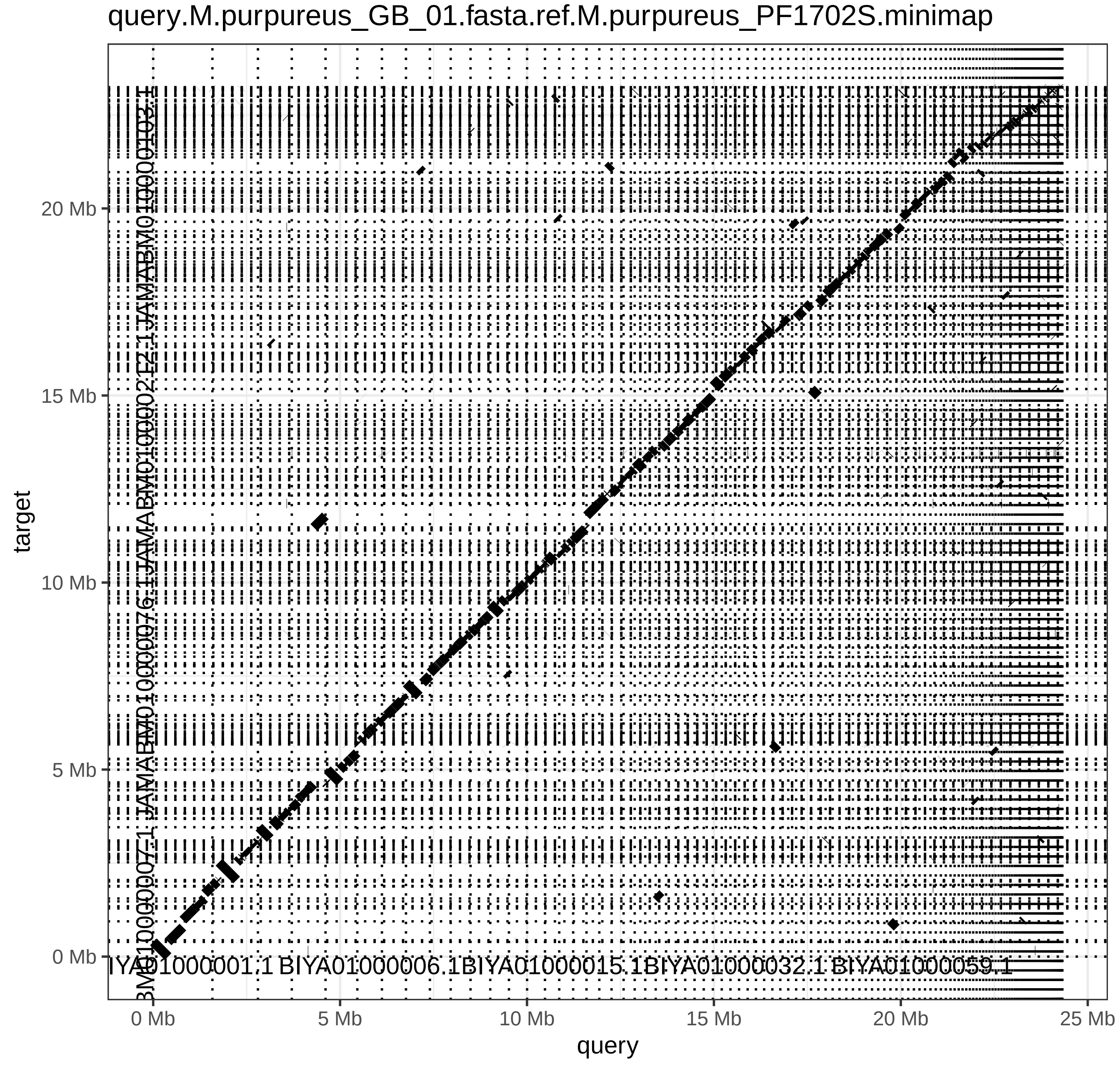
query.M.purpureus_GB_01.fasta.ref.M.purpureus_PF1702S.minimap
target
query
0 Mb
5 Mb
10 Mb
15 Mb
20 Mb
25 Mb
BIYA01000006.1
BIYA01000015.1
BIYA01000032.1
BIYA01000059.1

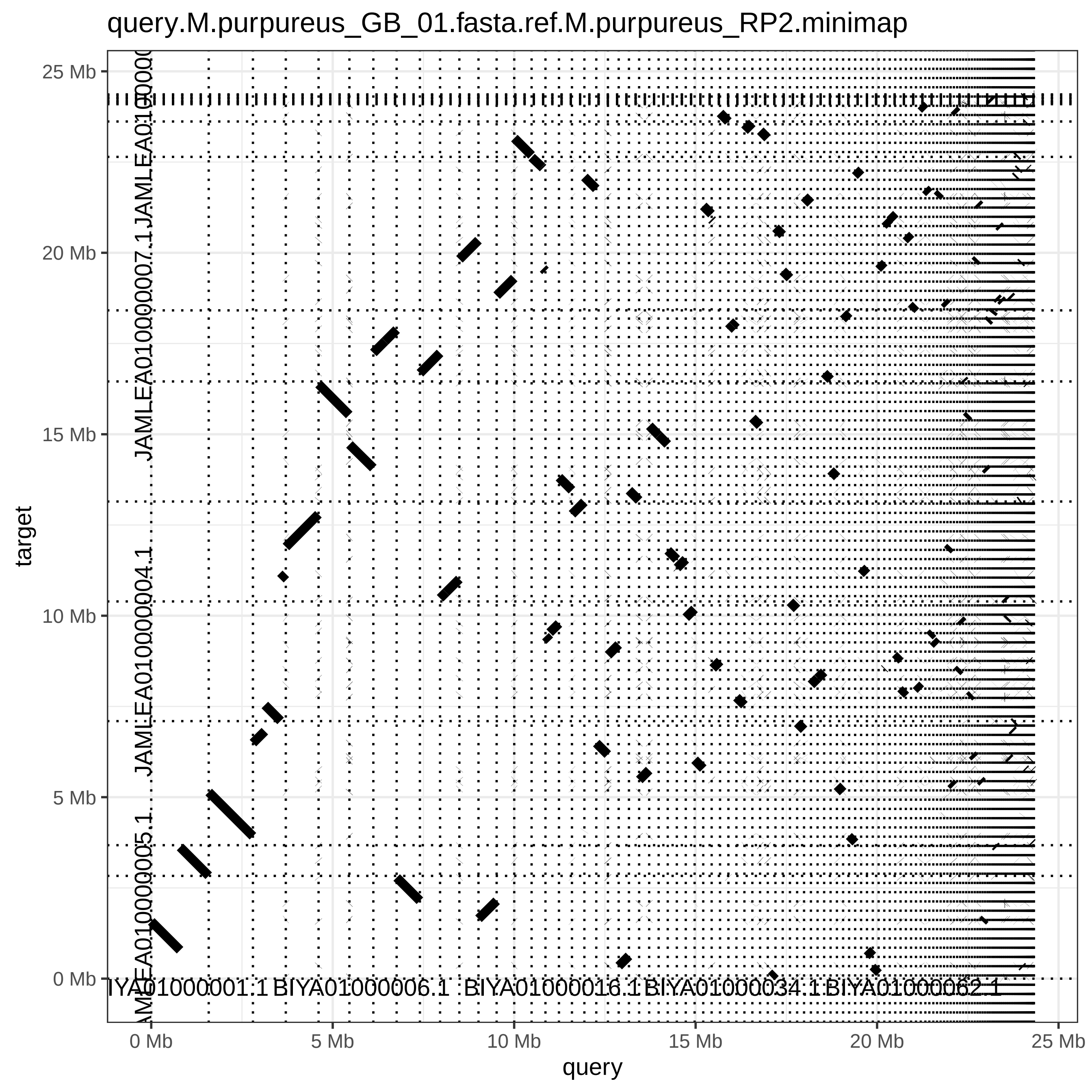
query.M.purpureus_GB_01.fasta.ref.M.purpureus_RP2.minimap
target
query
25 Mb
20 Mb
15 Mb
10 Mb
5 Mb
0 Mb
0 Mb
5 Mb
10 Mb
15 Mb
20 Mb
25 Mb
JAMLEA010000007.1
JAMLEA010000004.1
BIYA01000006.1
BIYA01000016.1
BIYA01000034.1
BIYA01000062.1

query.M.purpureus_GB_01.fasta.ref.M.purpureus_YJX_8.minimap

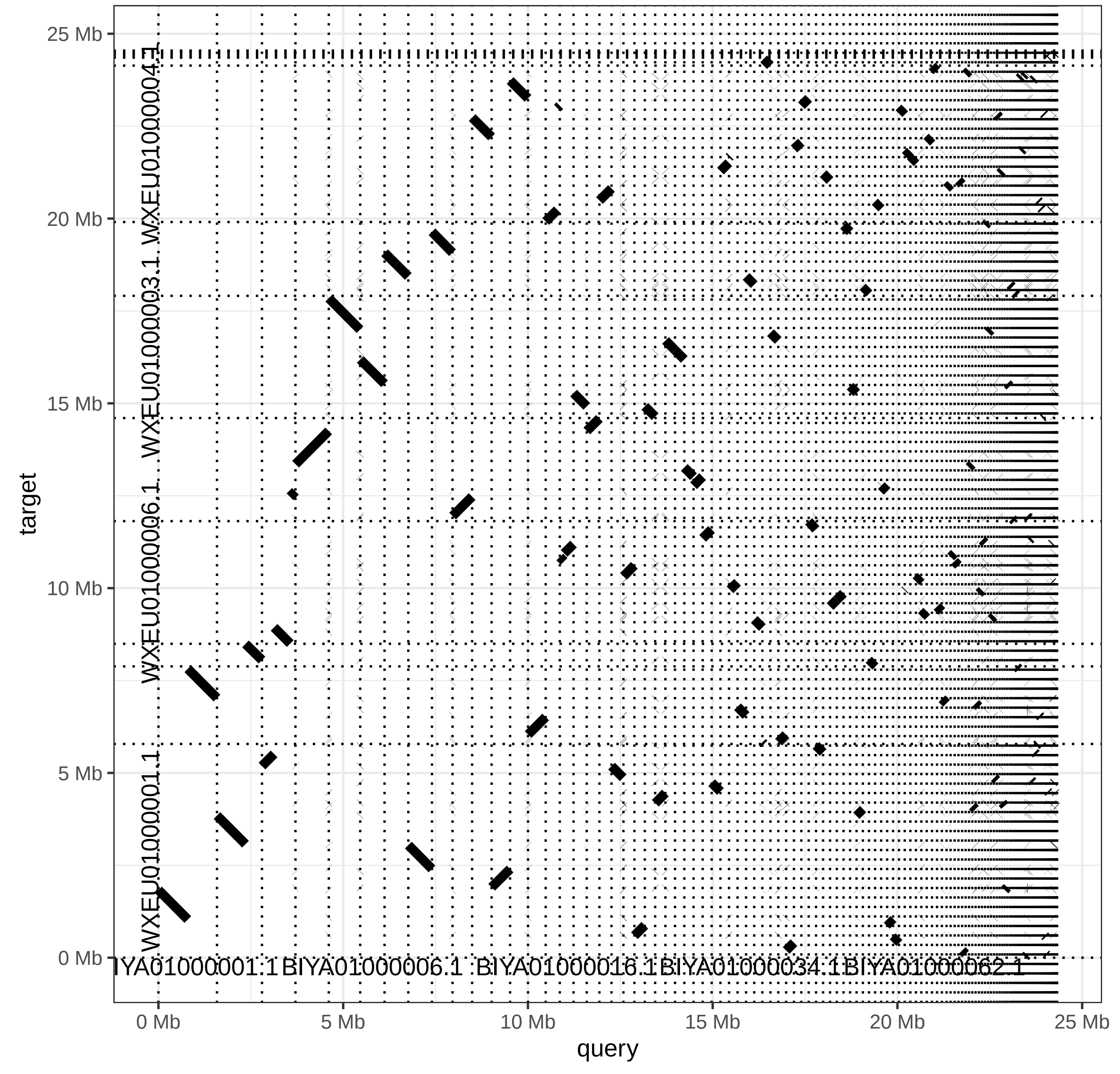

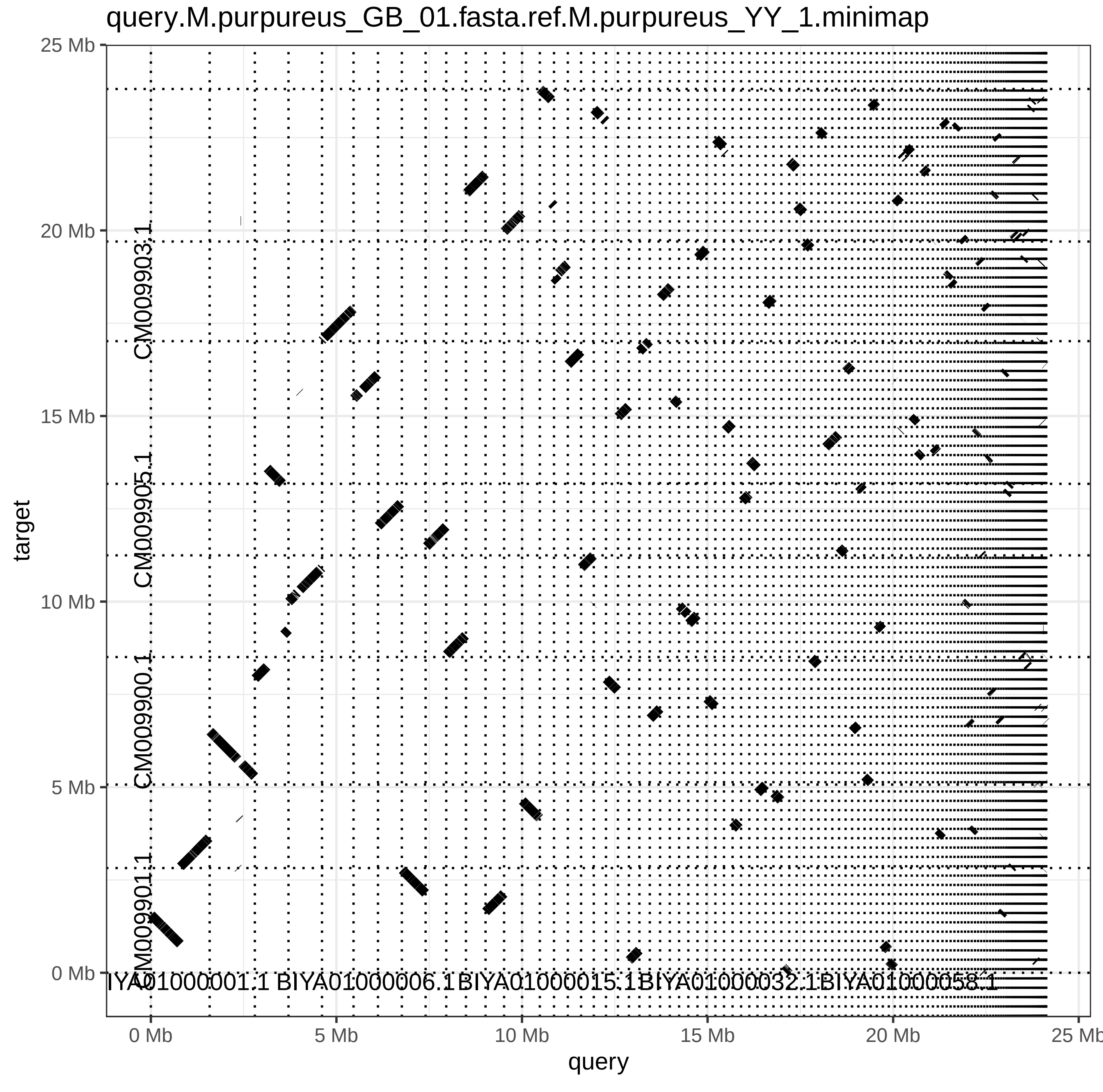

query.M.purpureus_GB_01.fasta.ref.M.purpureus_YY_1.minimap
25 Mb
20 Mb
15 Mb
10 Mb
5 Mb
0 Mb
target
CM009903.1
CM009905.1
CM009900.1
CM009901.1
BIYA01000001.1
BIYA01000006.1
BIYA01000015.1
BIYA01000032.1
BIYA01000058.1
0 Mb
5 Mb
10 Mb
15 Mb
20 Mb
25 Mb
query

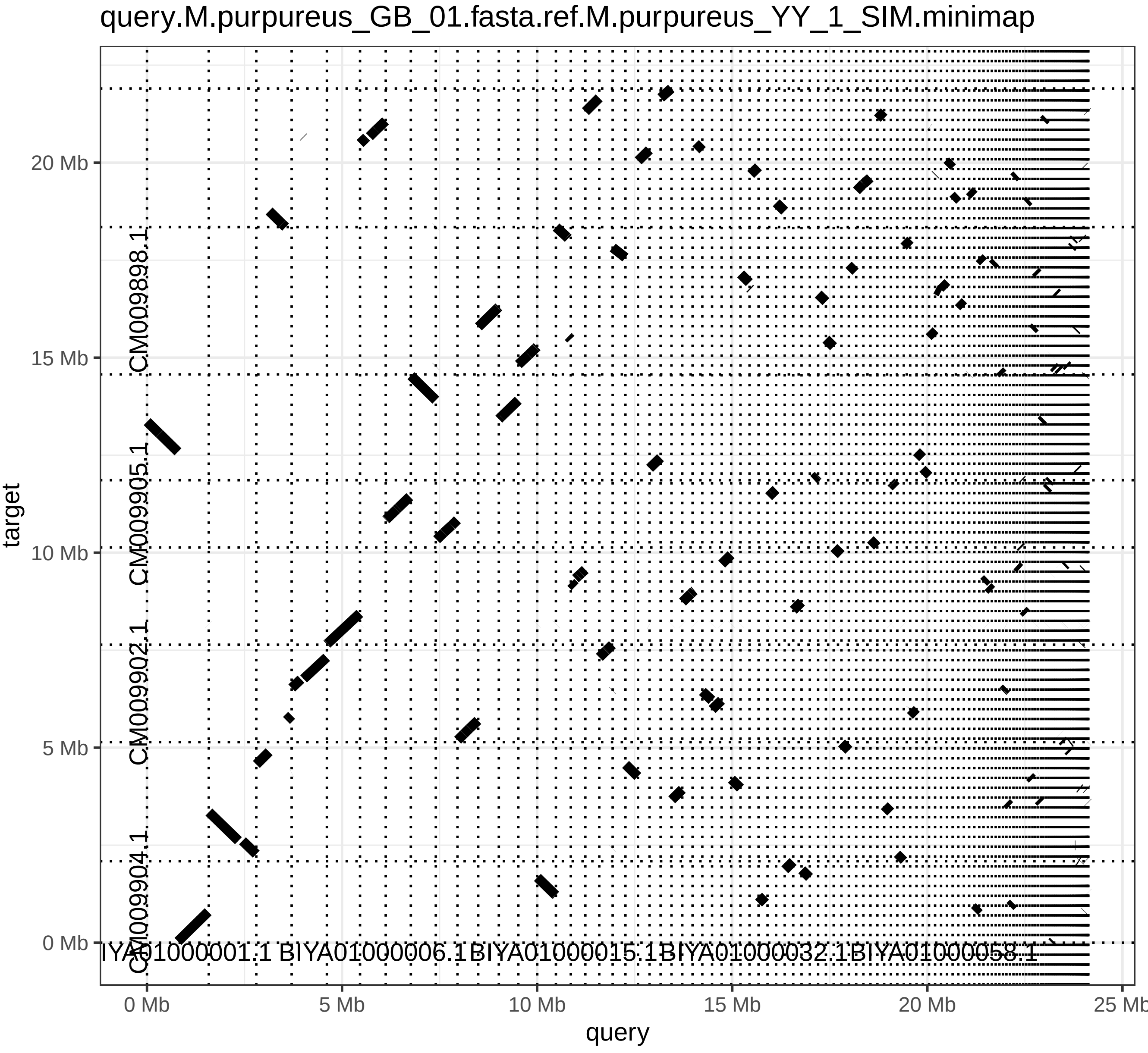

query.M.purpureus_GB_01.fasta.ref.M.purpureus_YY_1_SIM.minimap
target
query
0 Mb
5 Mb
10 Mb
15 Mb
20 Mb
25 Mb
CM009904.1
CM009902.1
CM009905.1
CM009898.1
IYA01000001.1
BIYA01000006.1
BIYA01000015.1
BIYA01000032.1
BIYA01000058.1

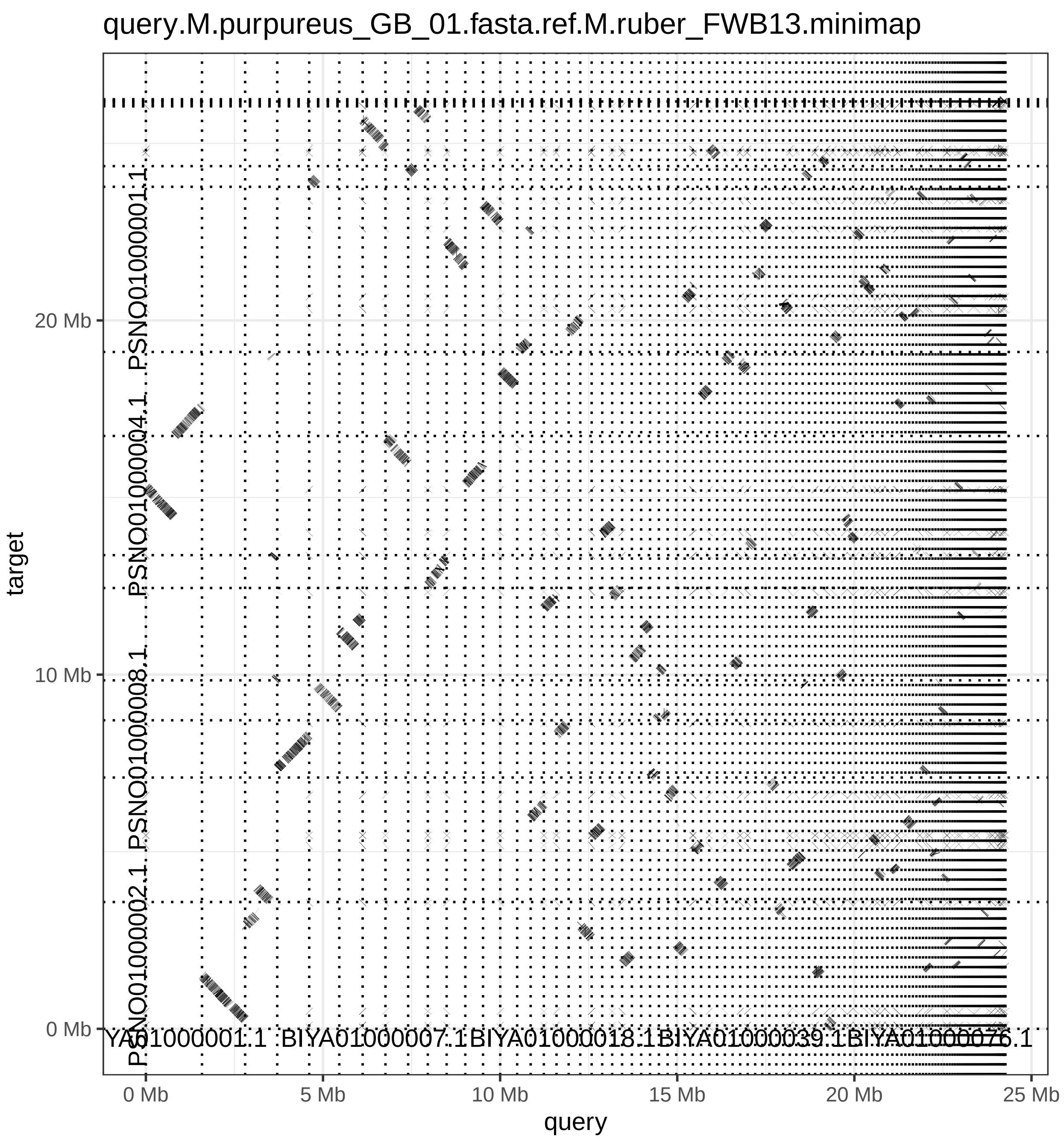
query.M.purpureus_GB_01.fasta.ref.M.ruber_FWB13.minimap
target
20 Mb
10 Mb
0 Mb
PSNO01000001.1
PSNO01000004.1
PSNO01000008.1
PSNO01000002.1
PSNO01000
YA01000001.1
BIYA01000007.1
BIYA01000018.1
BIYA01000039.1
BIYA01000076.1
0 Mb
5 Mb
10 Mb
15 Mb
20 Mb
25 Mb
query

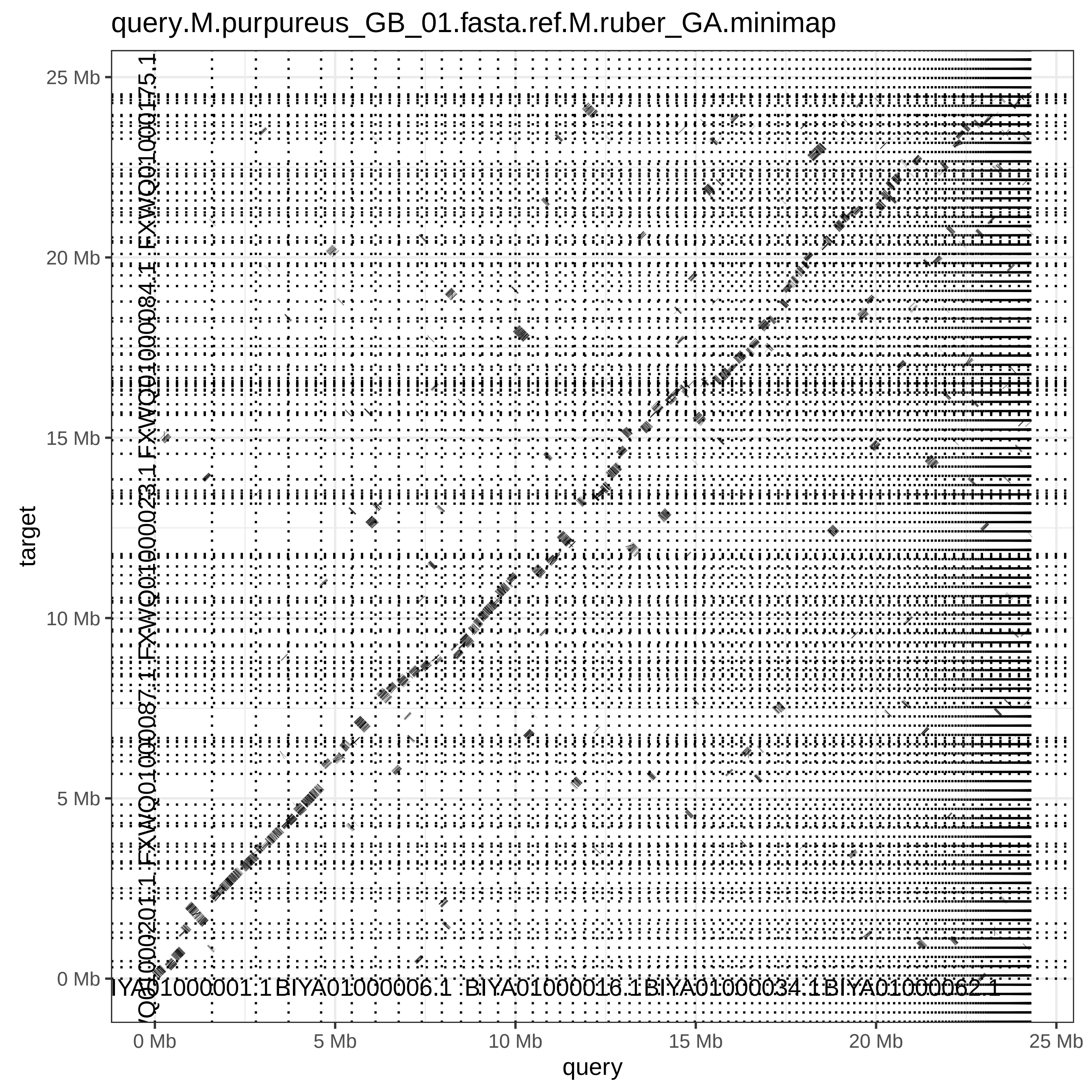
query.M.purpureus_GB_01.fasta.ref.M.ruber_GA.minimap
target
query
0 Mb
5 Mb
10 Mb
15 Mb
20 Mb
25 Mb
BIYA01000001.1
BIYA01000006.1
BIYA01000016.1
BIYA01000034.1
BIYA01000062.1
FXWQ01000201.1
FXWQ01000087.1
FXWQ01000023.1
FXWQ01000084.1
FXWQ01000175.1

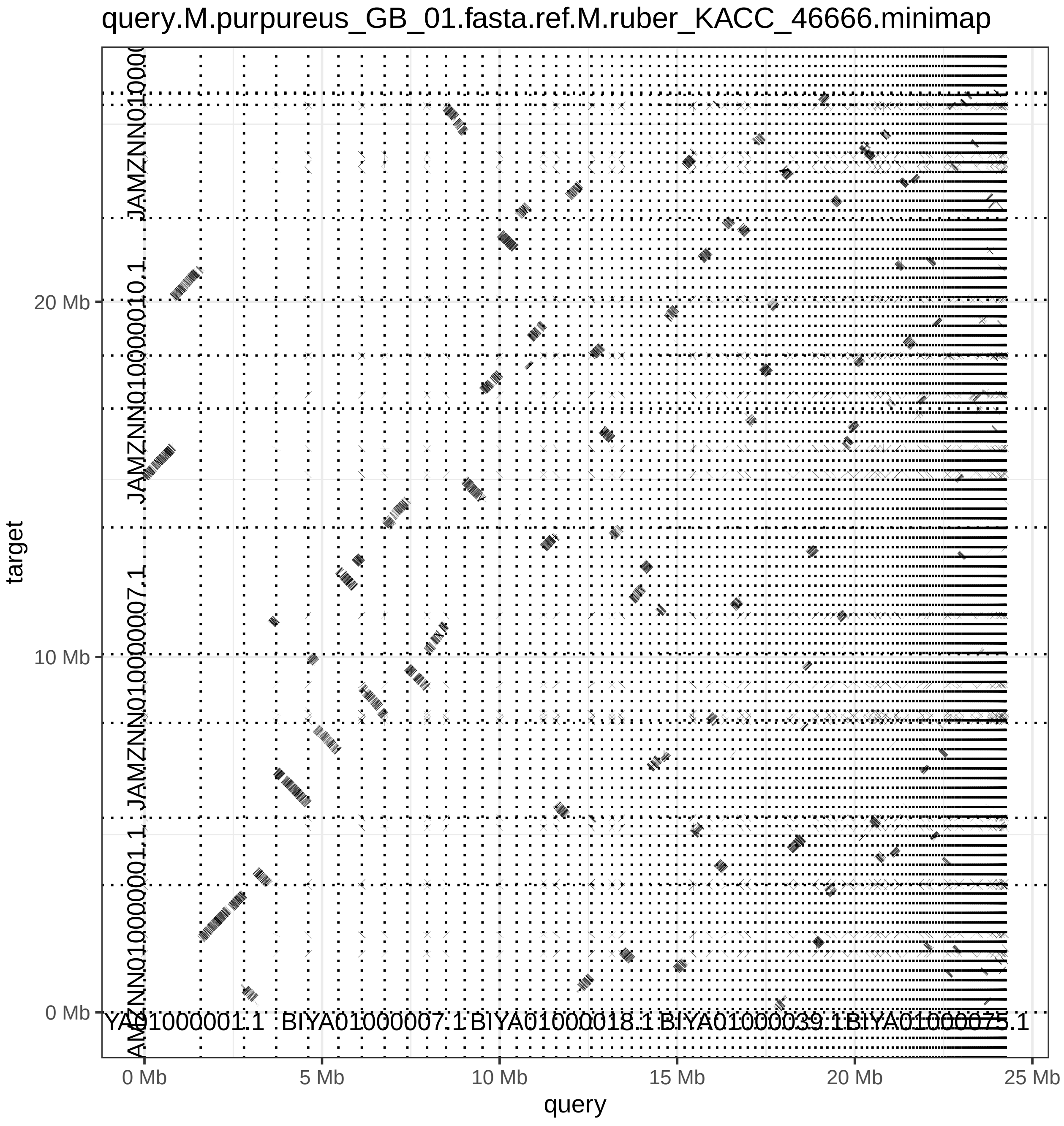

query.M.purpureus_GB_01.fasta.ref.M.ruber_KACC_46666.minimap
target
query
0 Mb
5 Mb
10 Mb
15 Mb
20 Mb
25 Mb
10 Mb
20 Mb
YA01000001.1
BIYA01000007.1
BIYA01000018.1
BIYA01000039.1
BIYA01000075.1
JAMZNN0100001
JAMZNN010000010.1
JAMZNN010000007.1
AMZNN010000001.1

query.M.purpureus_HQ1.fasta.ref.M.pilosus_K104061.minimap

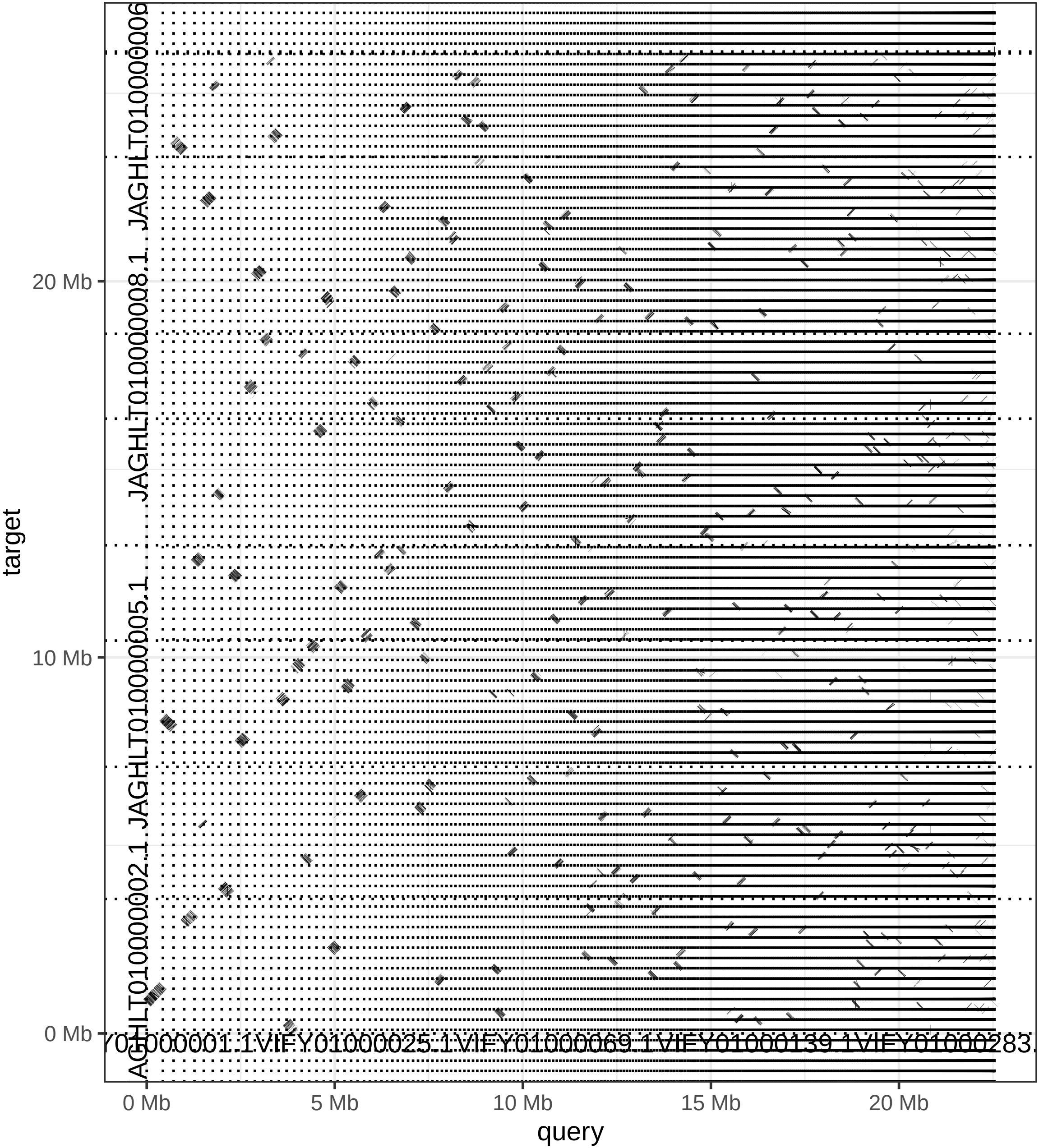

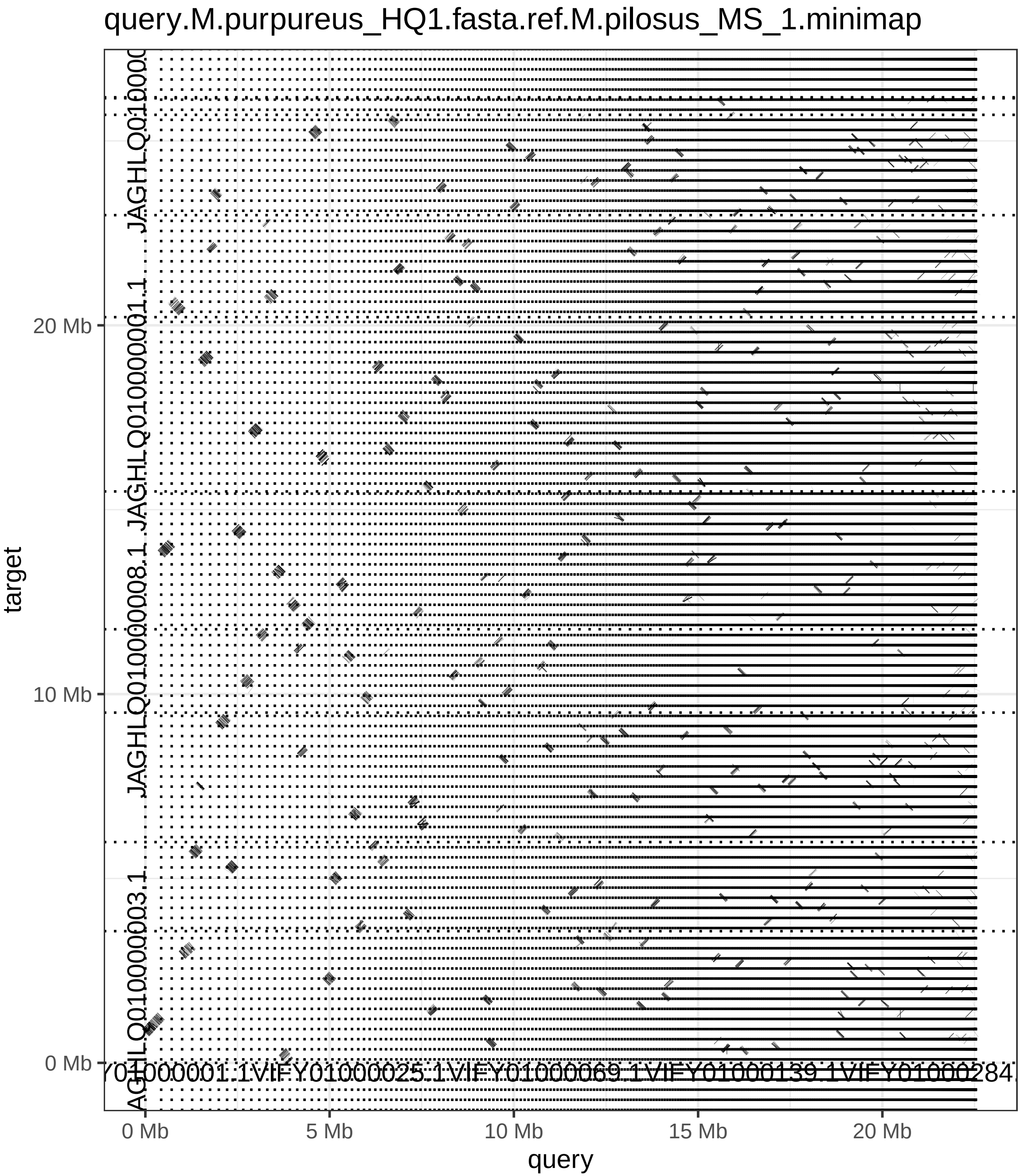

query.M.purpureus_HQ1.fasta.ref.M.pilosus_MS_1.minimap
target
query
0 Mb
5 Mb
10 Mb
15 Mb
20 Mb
10 Mb
20 Mb
JAGHLQ010000008.1

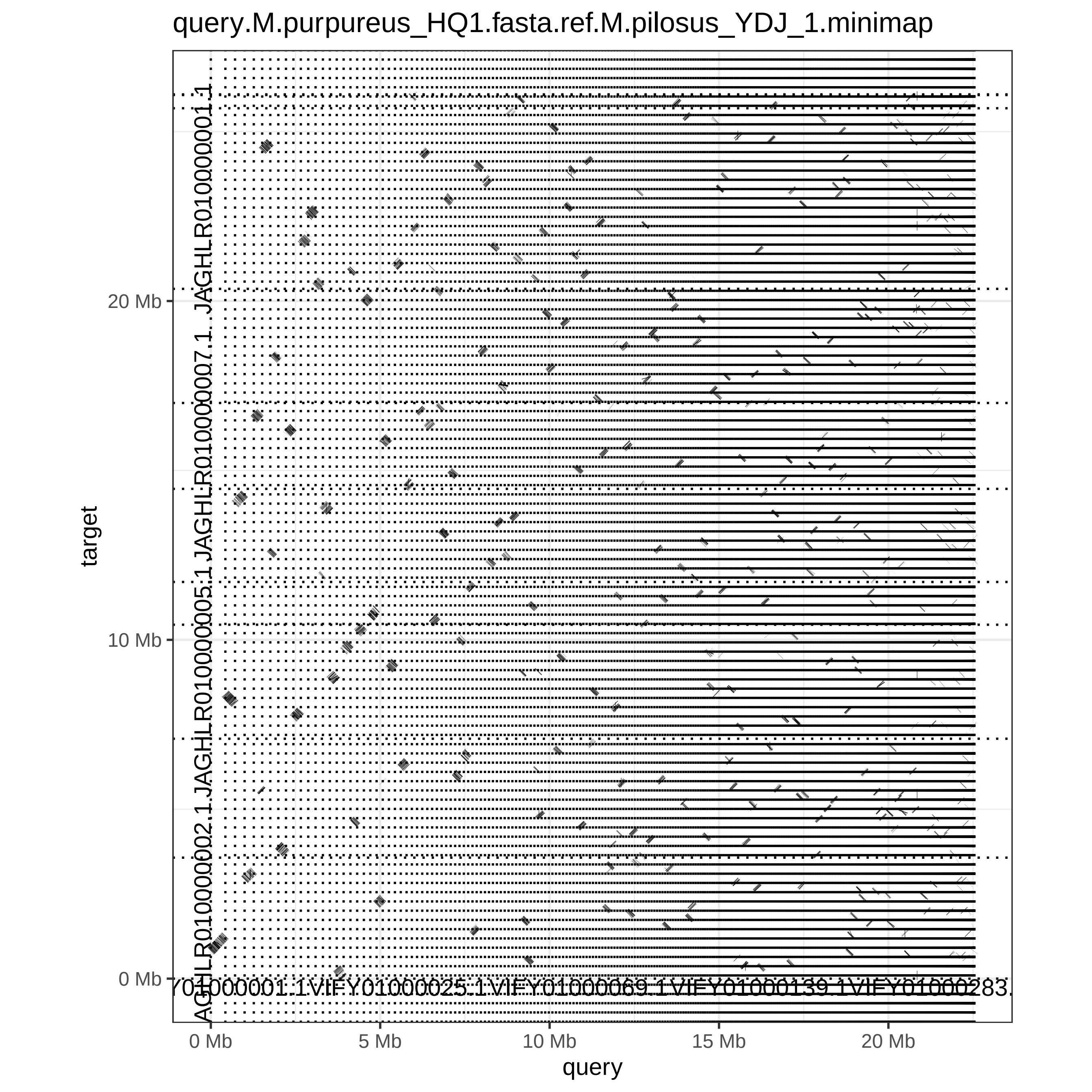
query.M.purpureus_HQ1.fasta.ref.M.pilosus_YDJ_1.minimap
target
20 Mb
10 Mb
0 Mb
0 Mb
5 Mb
10 Mb
15 Mb
20 Mb
query

query.M.purpureus_HQ1.fasta.ref.M.pilosus_YDJ_2.minimap

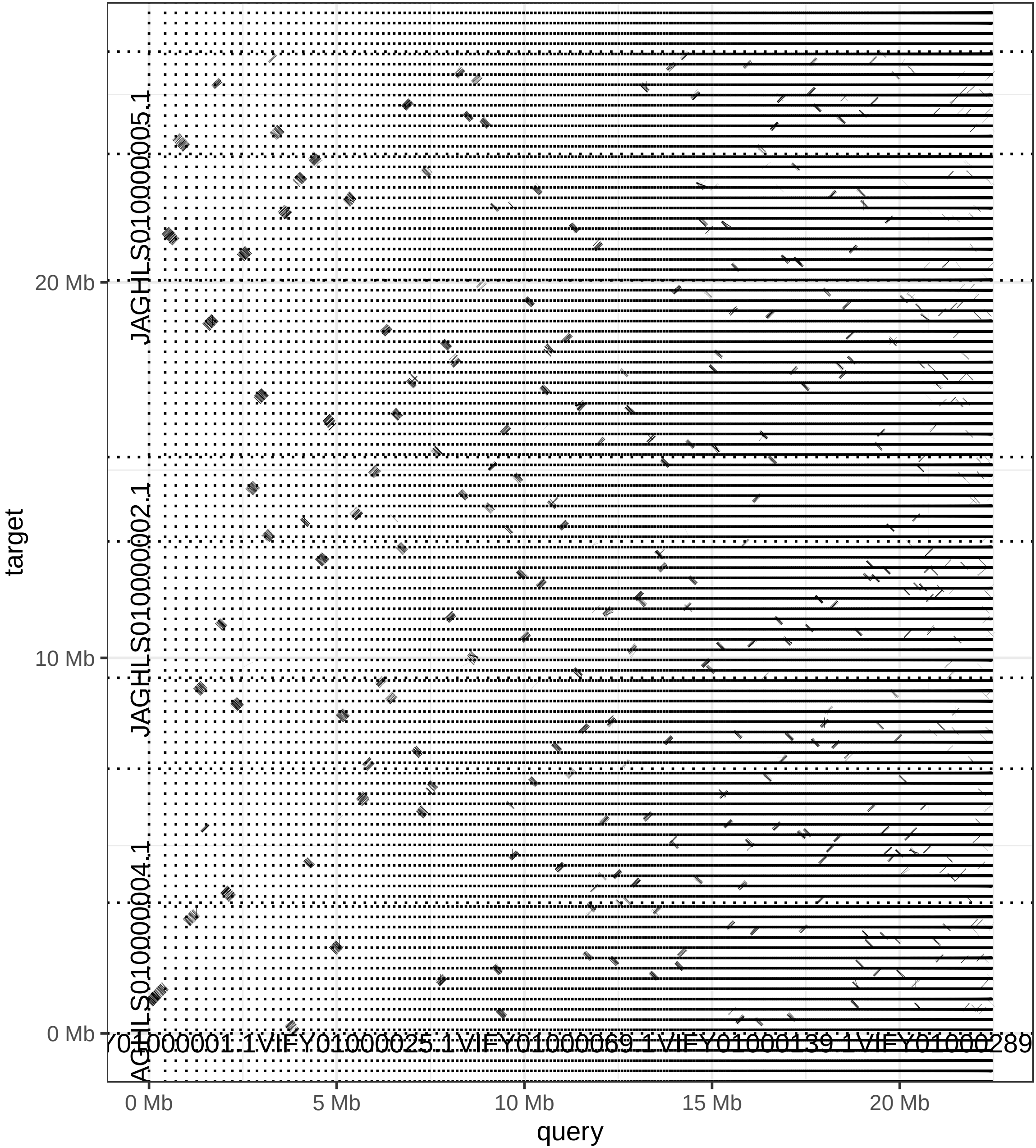

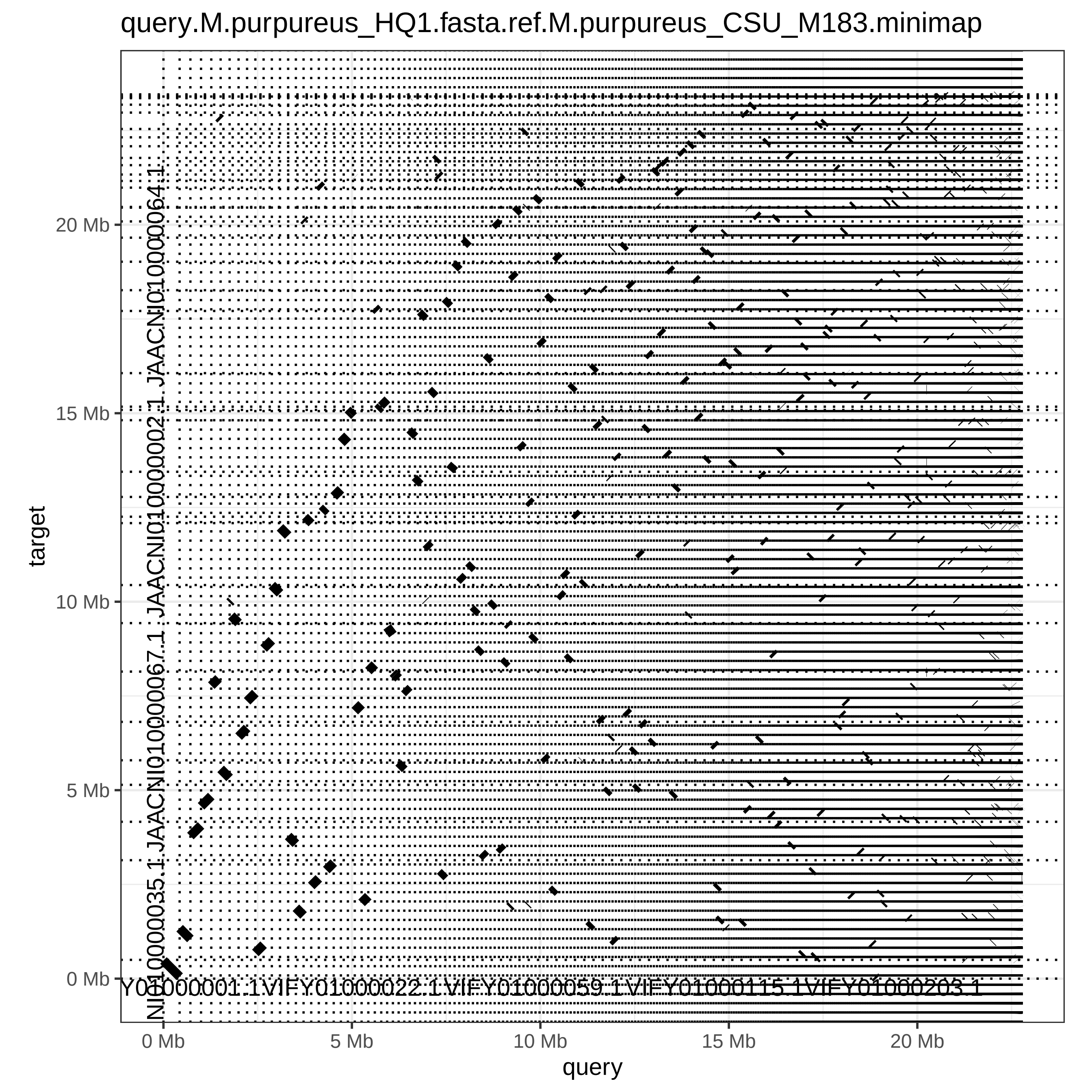

query.M.purpureus_HQ1.fasta.ref.M.purpureus_CSU_M183.minimap
target
query
0 Mb
5 Mb
10 Mb
15 Mb
20 Mb
0 Mb
5 Mb
10 Mb
15 Mb
20 Mb

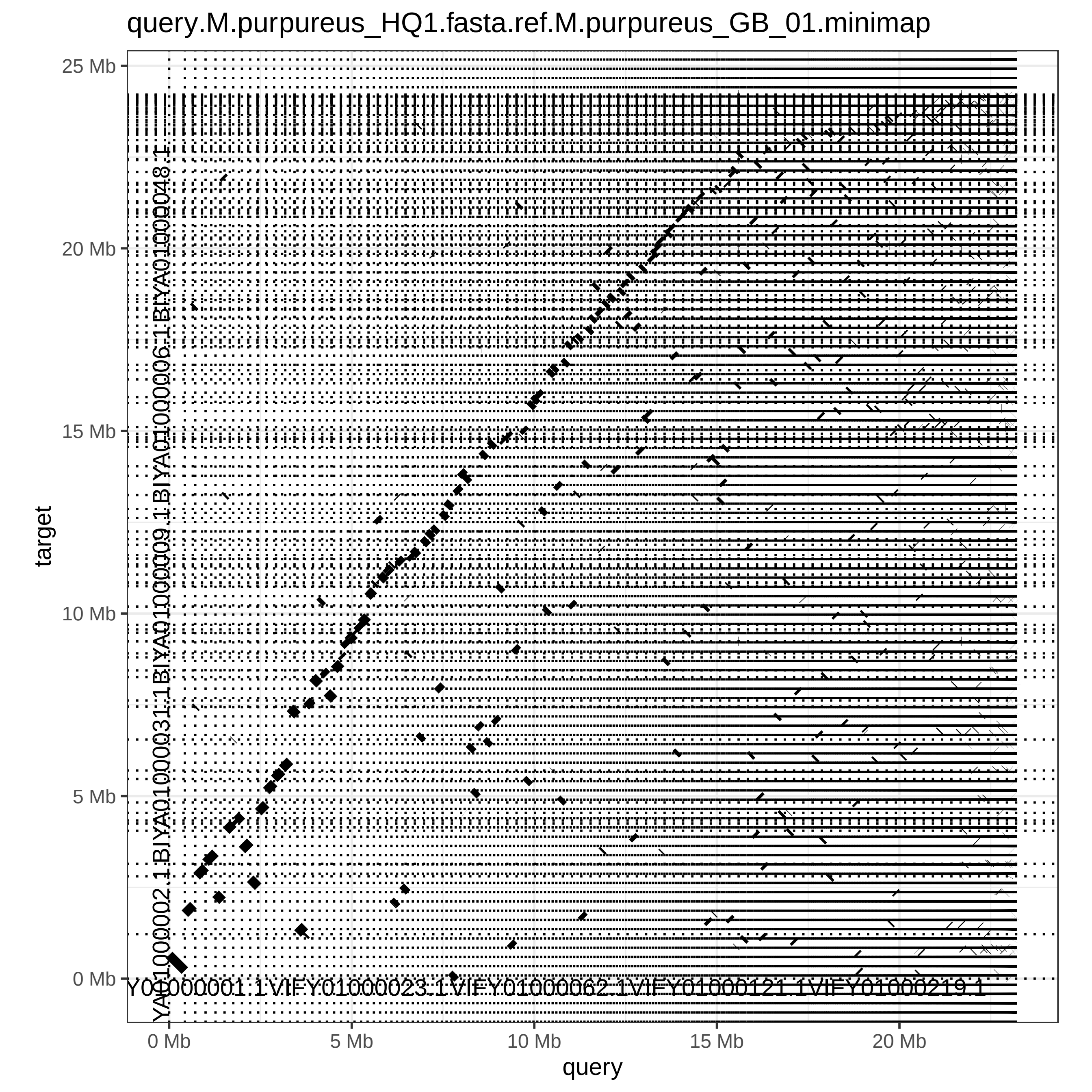

query.M.purpureus_HQ1.fasta.ref.M.purpureus_GB_01.minimap
target
25 Mb
20 Mb
15 Mb
10 Mb
5 Mb
0 Mb
query
0 Mb
5 Mb
10 Mb
15 Mb
20 Mb

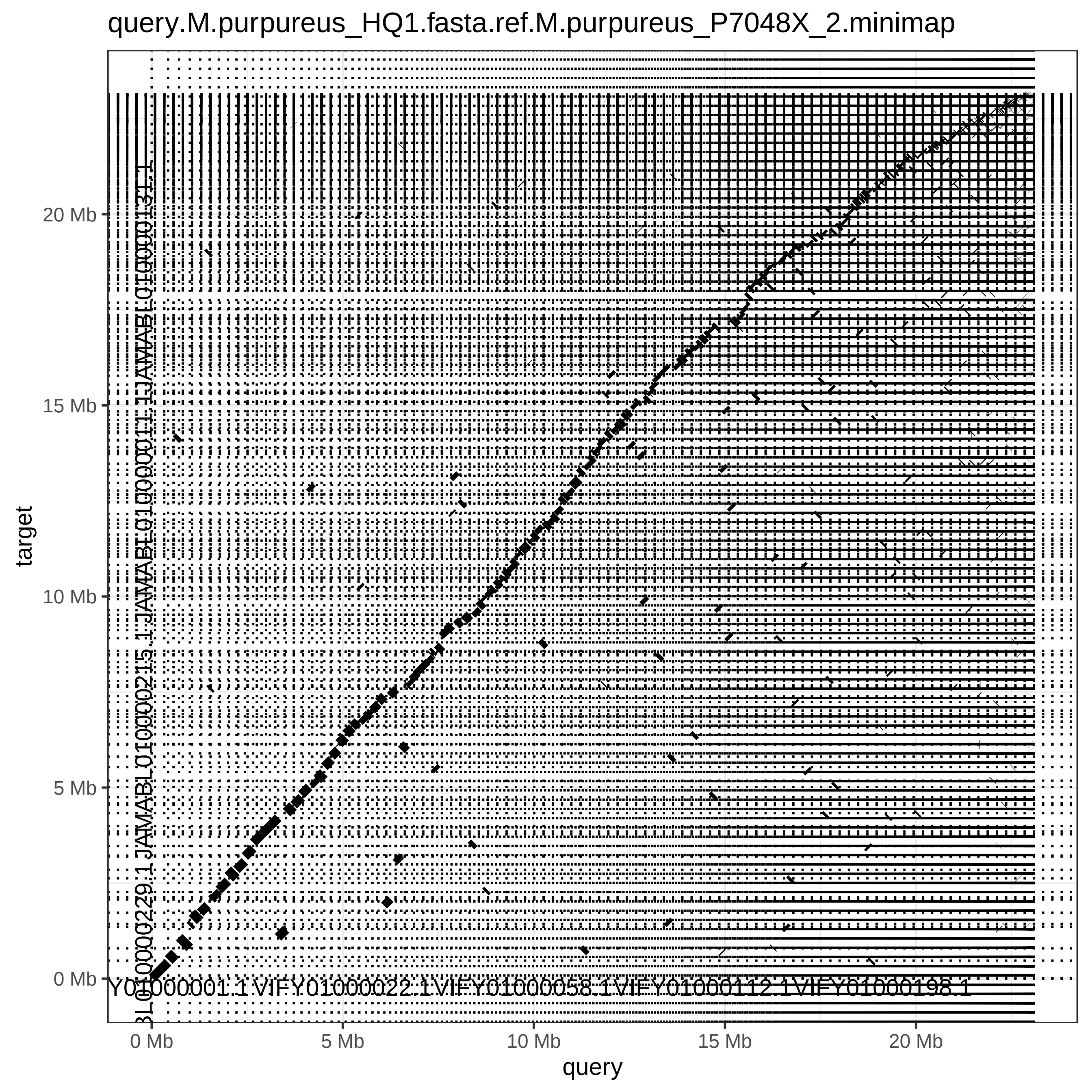

query.M.purpureus_HQ1.fasta.ref.M.purpureus_P7048X_2.minimap
target
20 Mb
15 Mb
10 Mb
5 Mb
0 Mb
VIFY01000022.1
VIFY01000058.1
VIFY01000112.1
VIFY01000198.1
0 Mb
5 Mb
10 Mb
15 Mb
20 Mb
query

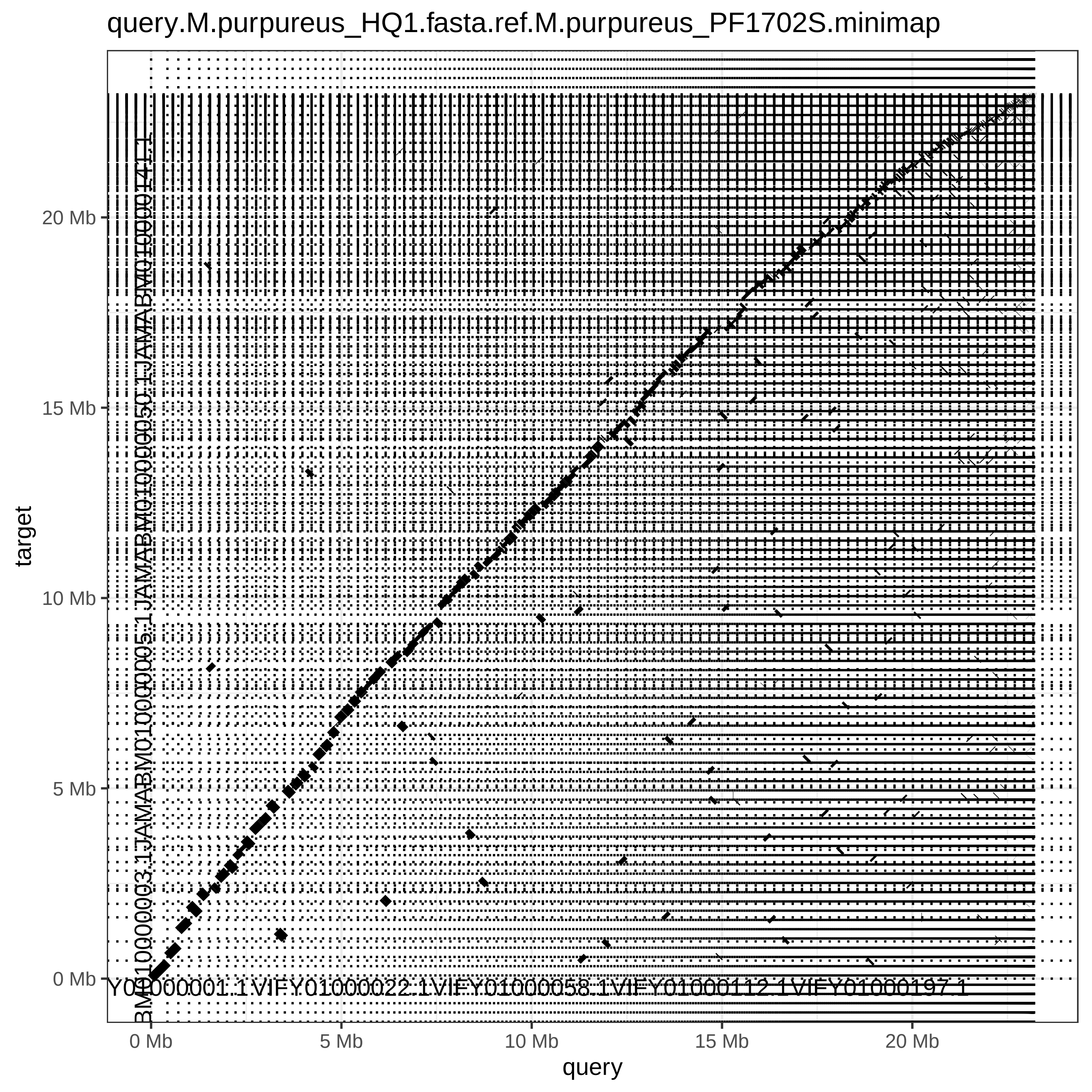
query.M.purpureus_HQ1.fasta.ref.M.purpureus_PF1702S.minimap
target
20 Mb
15 Mb
10 Mb
5 Mb
0 Mb
Y01000001.1VIFY01000022.1VIFY01000058.1VIFY01000112.1VIFY01000197.1
0 Mb
5 Mb
10 Mb
15 Mb
20 Mb
query

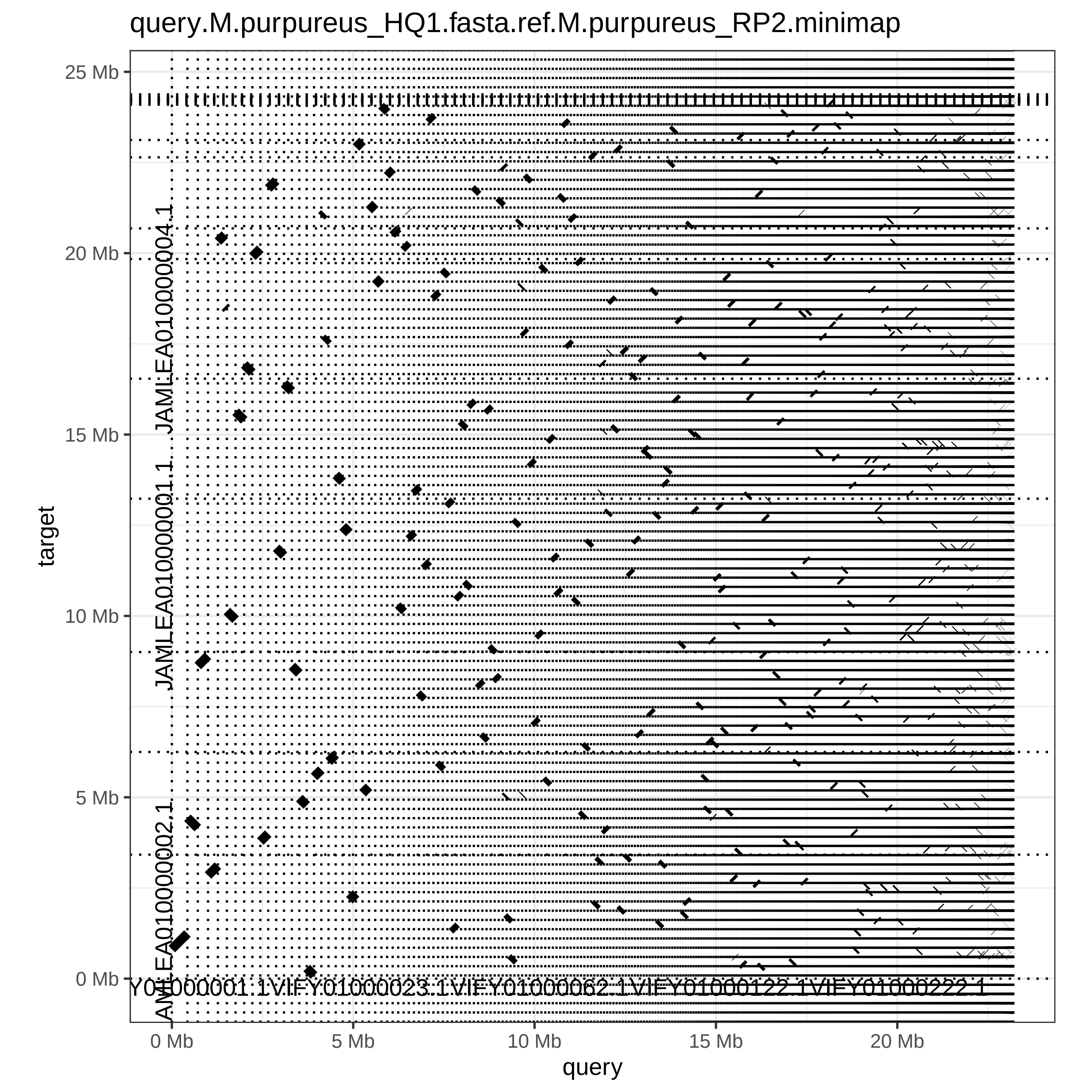
query.M.purpureus_HQ1.fasta.ref.M.purpureus_RP2.minimap
target
query
0 Mb
5 Mb
10 Mb
15 Mb
20 Mb
25 Mb
JAMLEA010000004.1
JAMLEA010000001.1
JAMLEA010000002.1
Y01000001.1VIFY01000023.1VIFY01000062.1VIFY01000122.1VIFY01000222.1

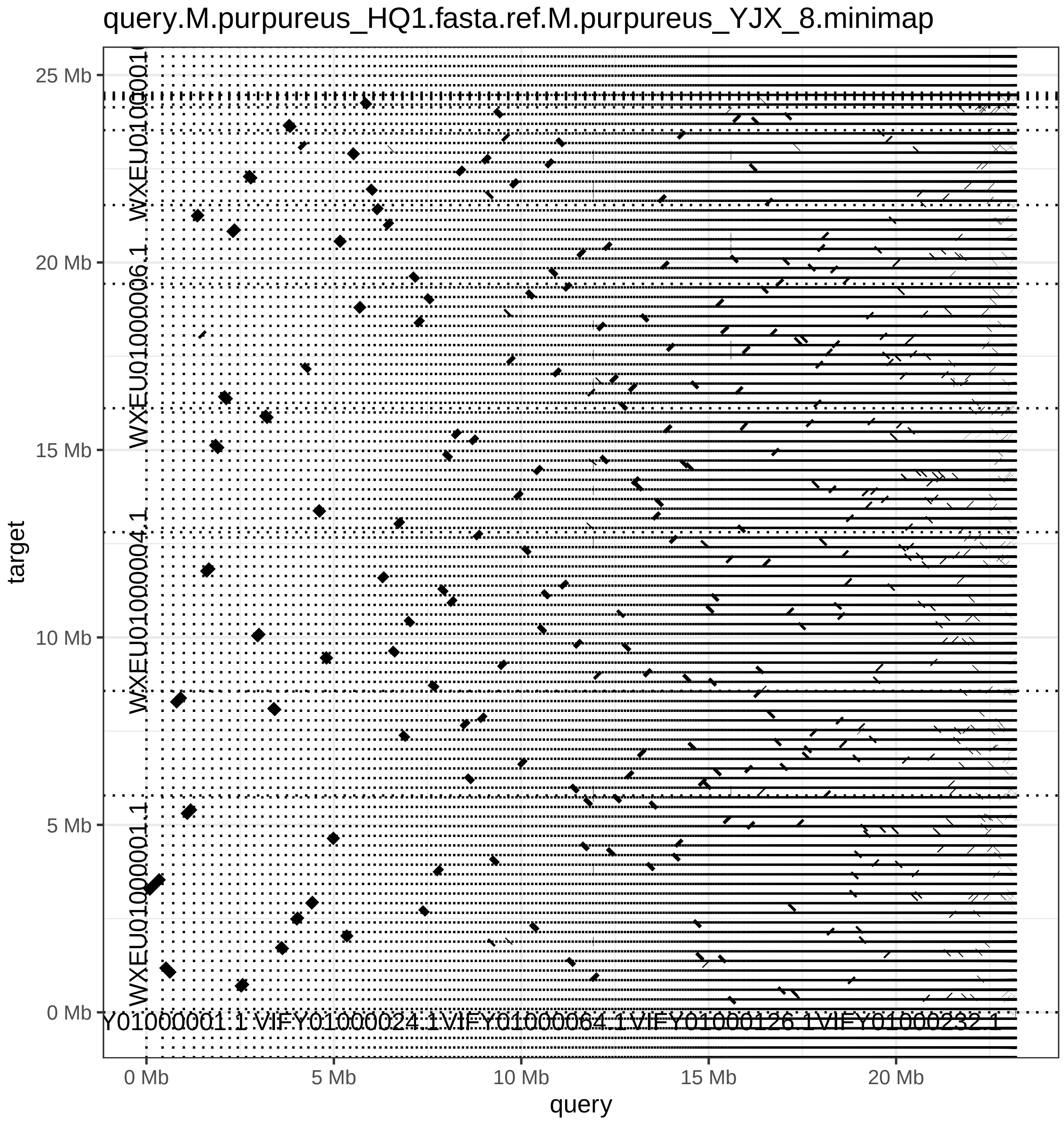

query.M.purpureus_HQ1.fasta.ref.M.purpureus_YJX_8.minimap
target
query
0 Mb
5 Mb
10 Mb
15 Mb
20 Mb
25 Mb
WXEU01000001.1
WXEU01000004.1
WXEU01000006.1
Y01000001.1
VIFY01000024.1
VIFY01000064.1
VIFY01000126.1
VIFY01000232.1

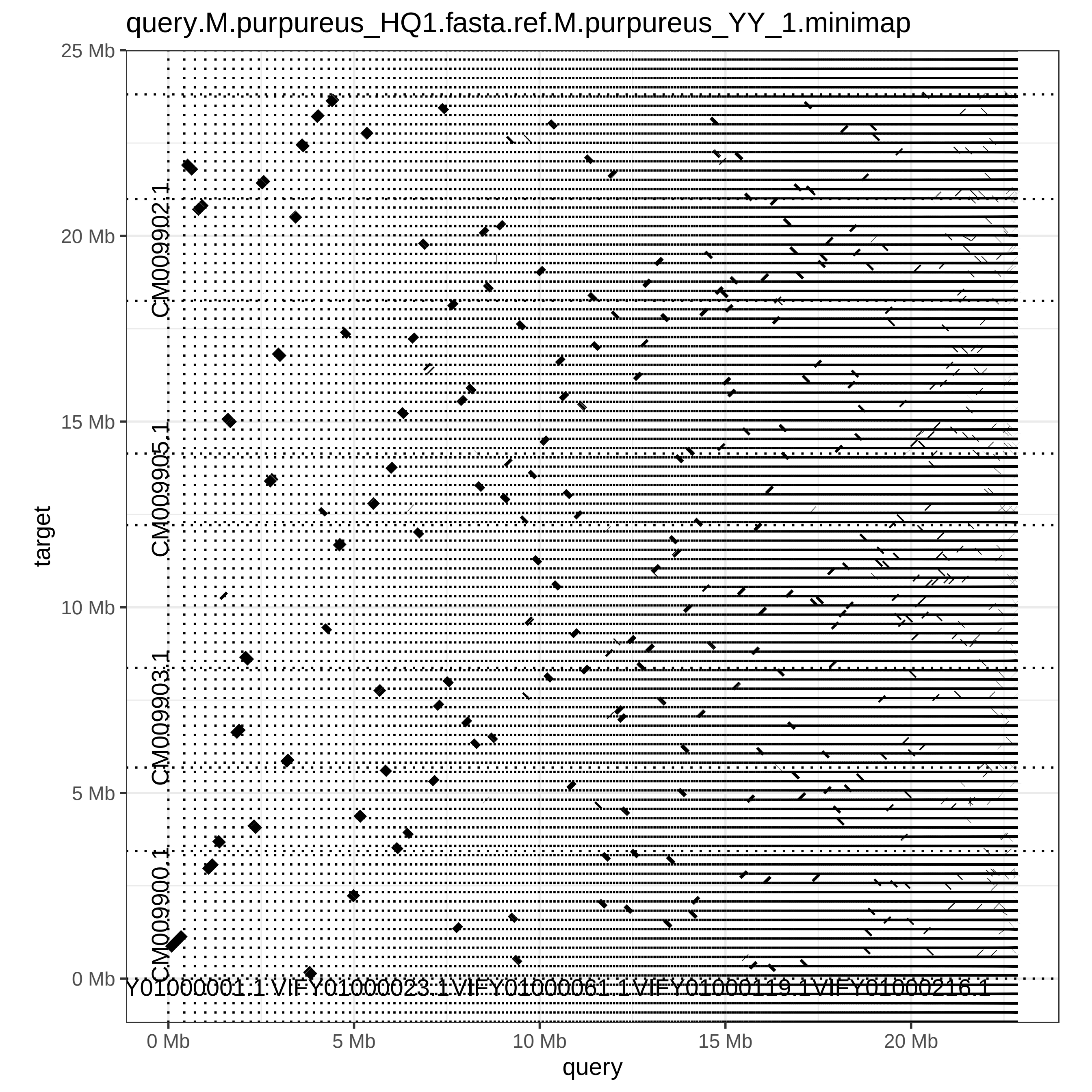

query.M.purpureus_HQ1.fasta.ref.M.purpureus_YY_1.minimap
target
25 Mb
20 Mb
15 Mb
10 Mb
5 Mb
0 Mb
CM009902.1
CM009905.1
CM009903.1
CM009900.1
Y01000001.1 VIFY01000023.1VIFY01000061.1VIFY01000119.1VIFY01000216.1
0 Mb
5 Mb
10 Mb
15 Mb
20 Mb
query

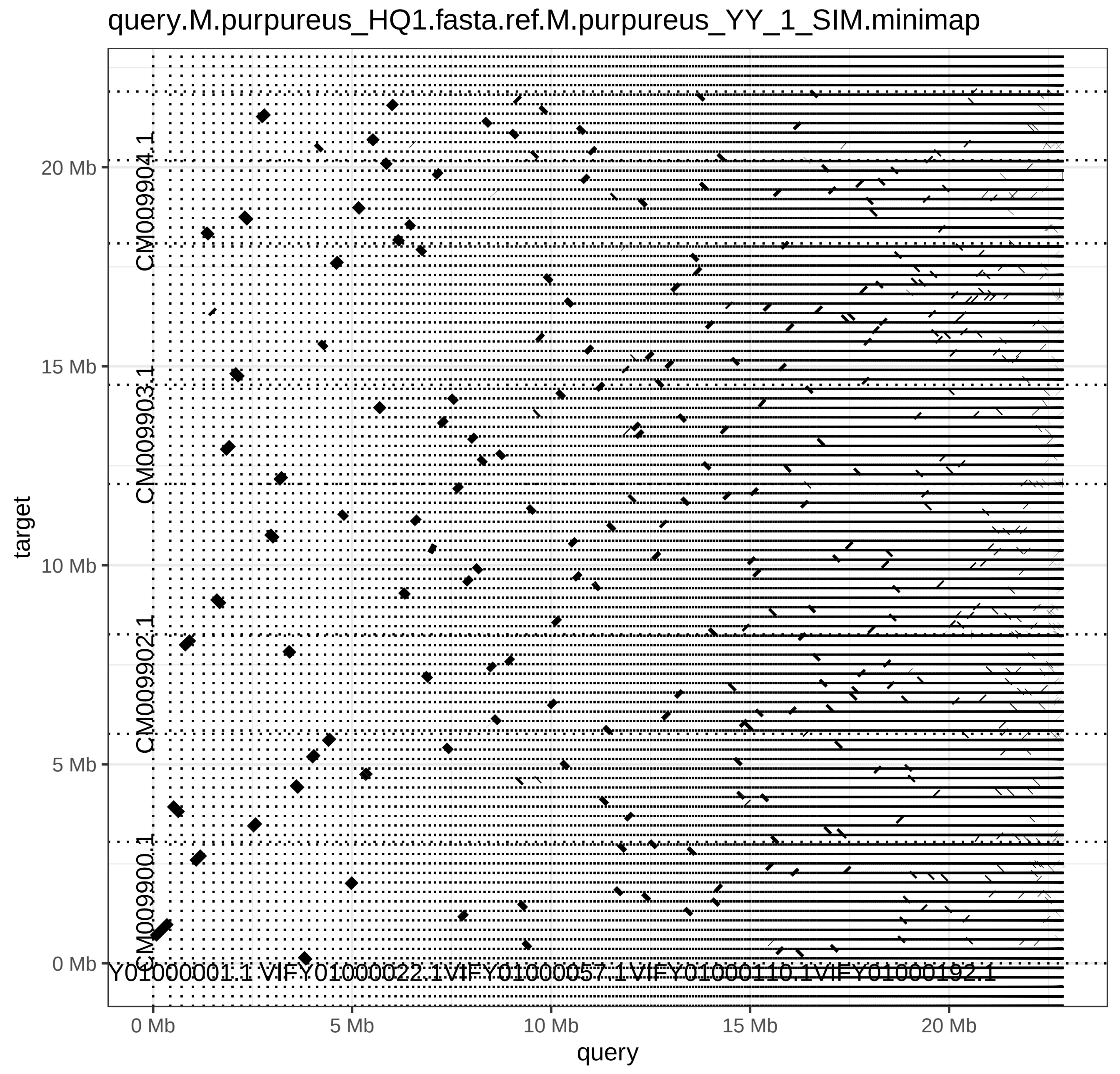
query.M.purpureus_HQ1.fasta.ref.M.purpureus_YY_1_SIM.minimap
target
query
0 Mb
5 Mb
10 Mb
15 Mb
20 Mb
CM009900.1
CM009902.1
CM009903.1
CM009904.1
Y01000001.1
VIFY01000022.1
VIFY01000057.1
VIFY01000110.1
VIFY01000192.1

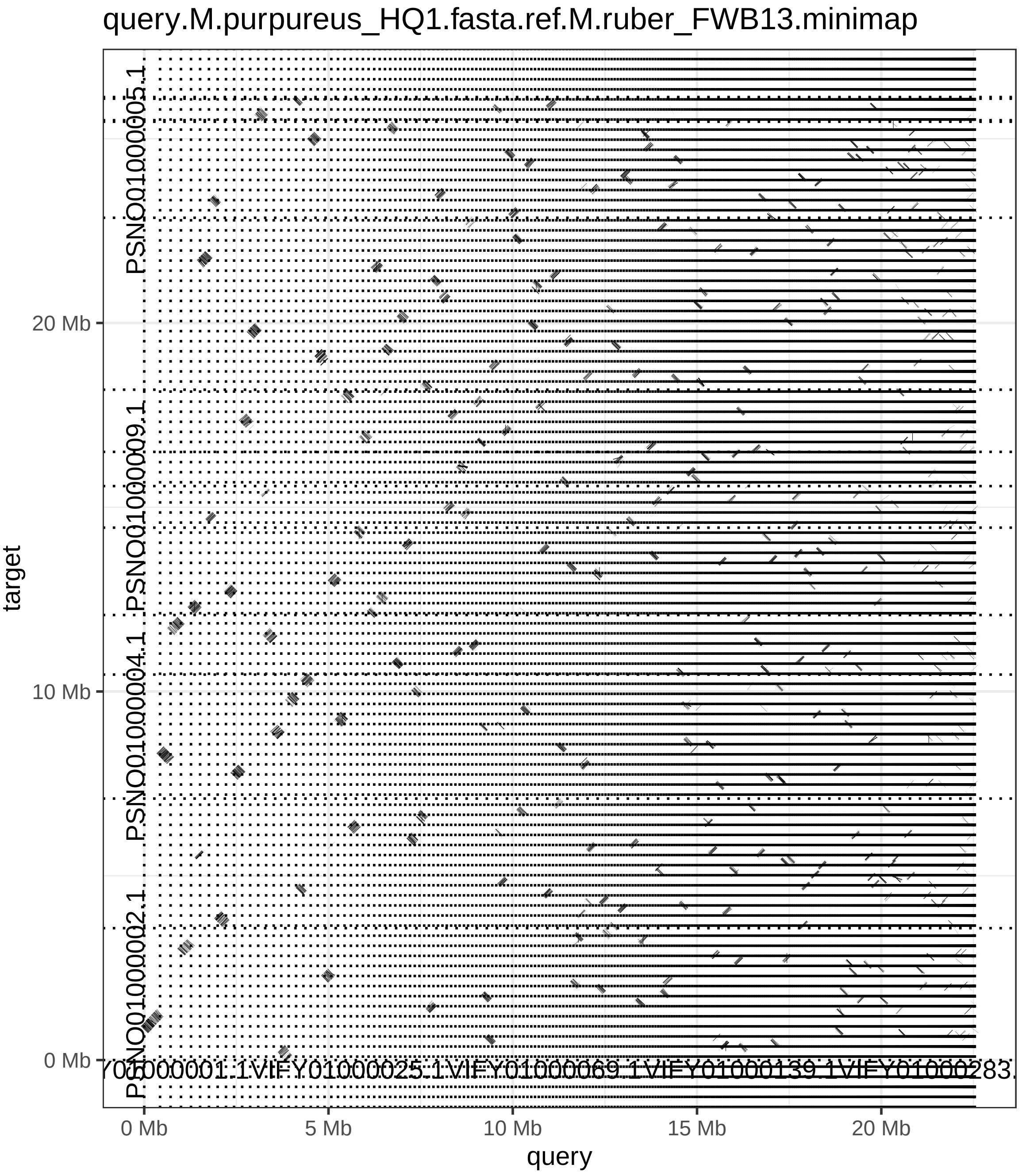
query.M.purpureus_HQ1.fasta.ref.M.ruber_FWB13.minimap
PSNO01000005.1
PSNO01000009.1
PSNO01000004.1
PSNO01000002.1
20 Mb
10 Mb
0 Mb
target
VIFY01000025.1
VIFY01000069.1
VIFY01000139.1
0 Mb
5 Mb
10 Mb
15 Mb
20 Mb
query

query.M.purpureus_HQ1.fasta.ref.M.ruber_GA.minimap

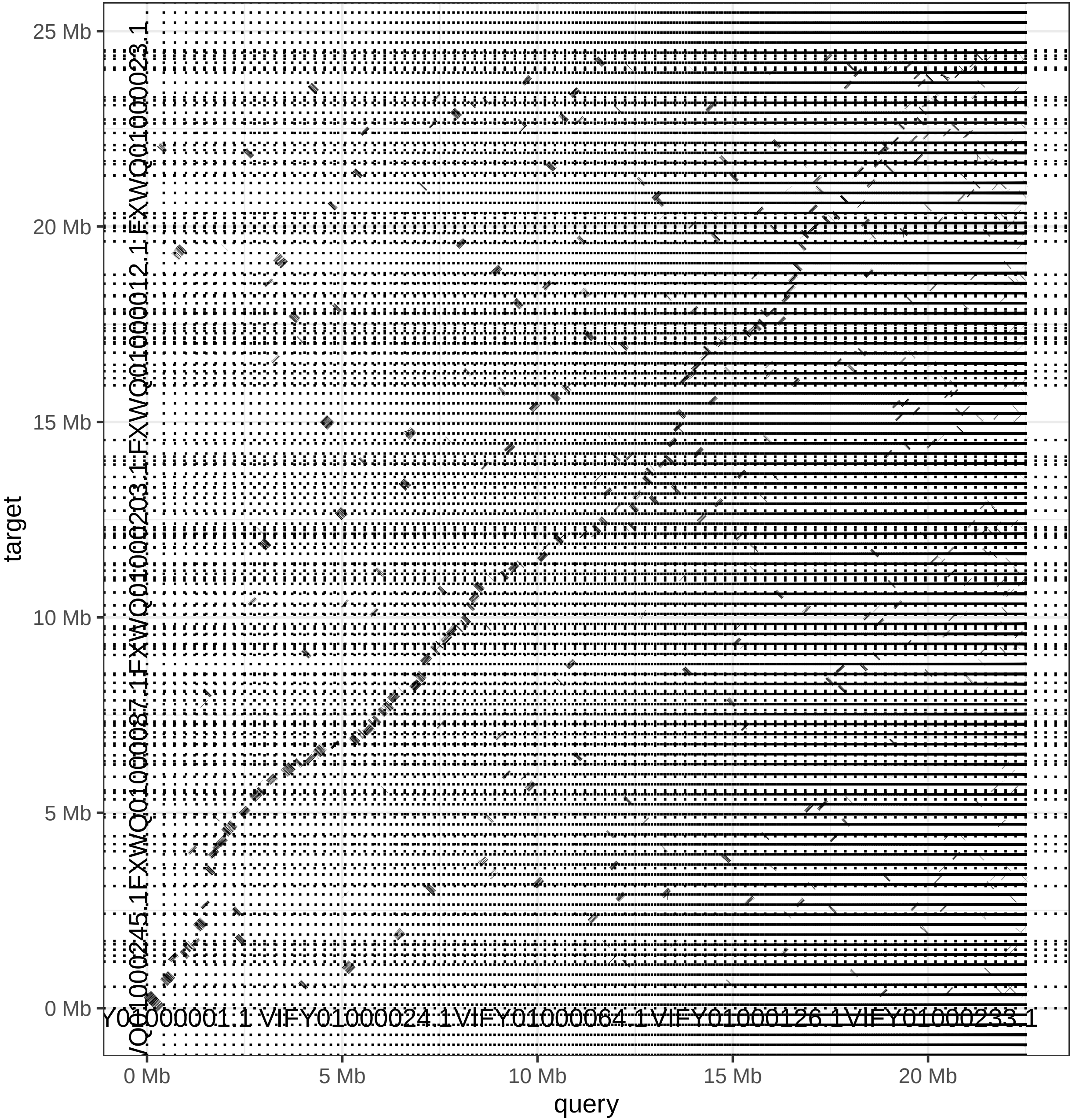

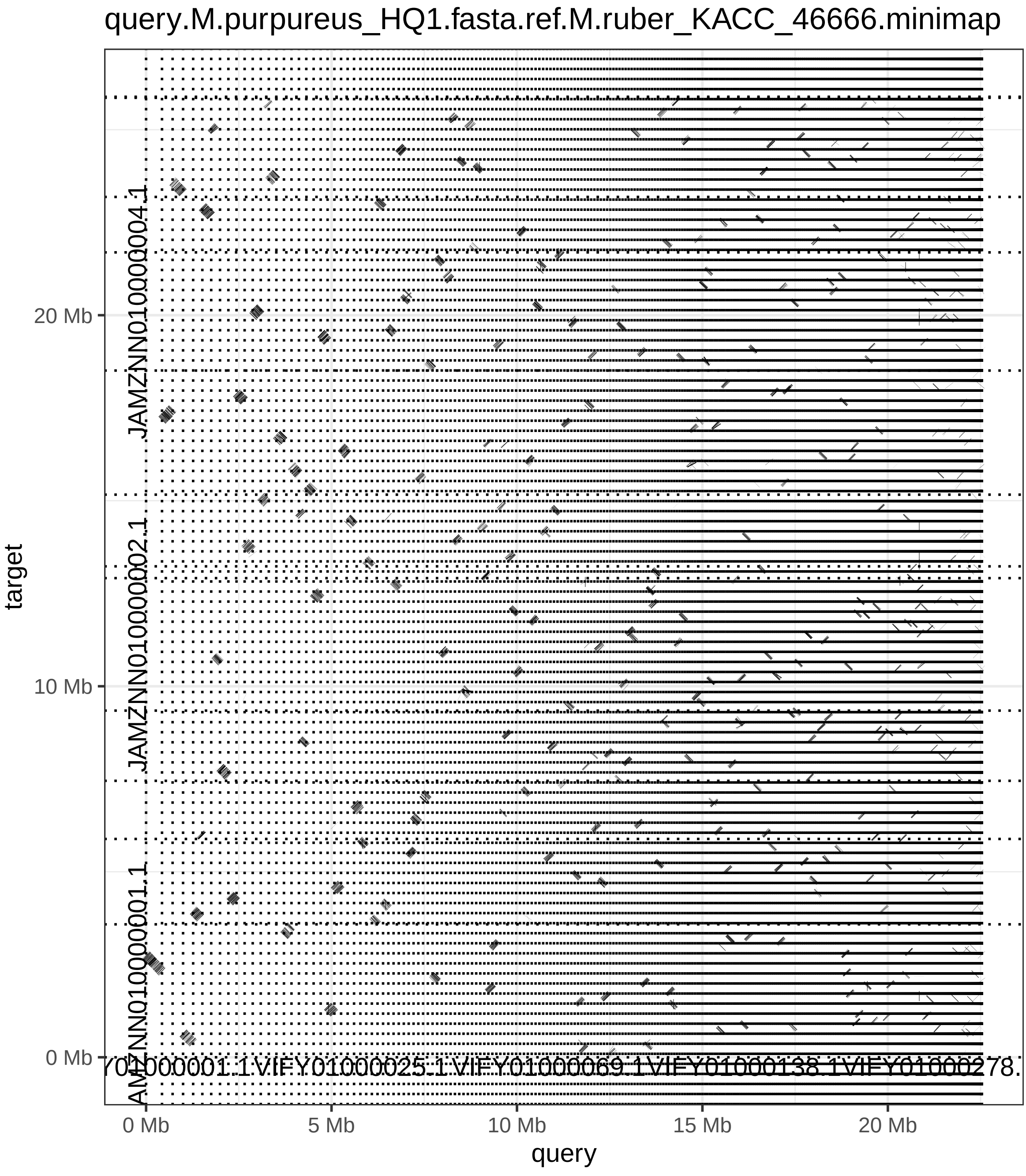
query.M.purpureus_HQ1.fasta.ref.M.ruber_KACC_46666.minimap
target
20 Mb
10 Mb
0 Mb
JAMZNN010000004.1
JAMZNN010000002.1
JAMZNN010000001.1
query
0 Mb
5 Mb
10 Mb
15 Mb
20 Mb

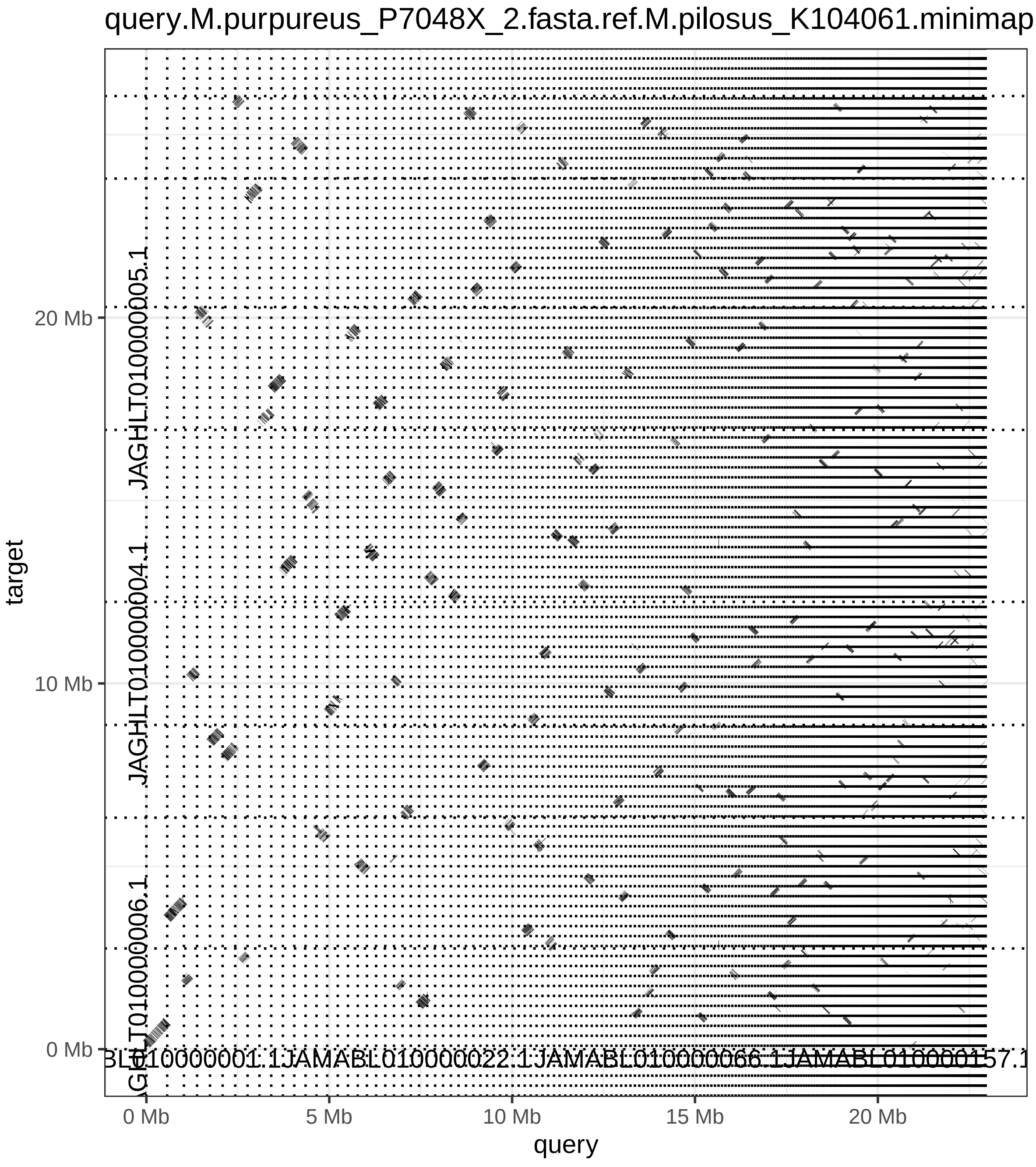
query.M.purpureus_P7048X_2.fasta.ref.M.pilosus_K104061.minimap
target
query
0 Mb
5 Mb
10 Mb
15 Mb
20 Mb
0 Mb
10 Mb
20 Mb
JAGHLT010000005.1
JAGHLT010000004.1
JAGHLT010000006.1
JAMABL010000022.1
JAMABL010000066.1
JAMABL010000157.1

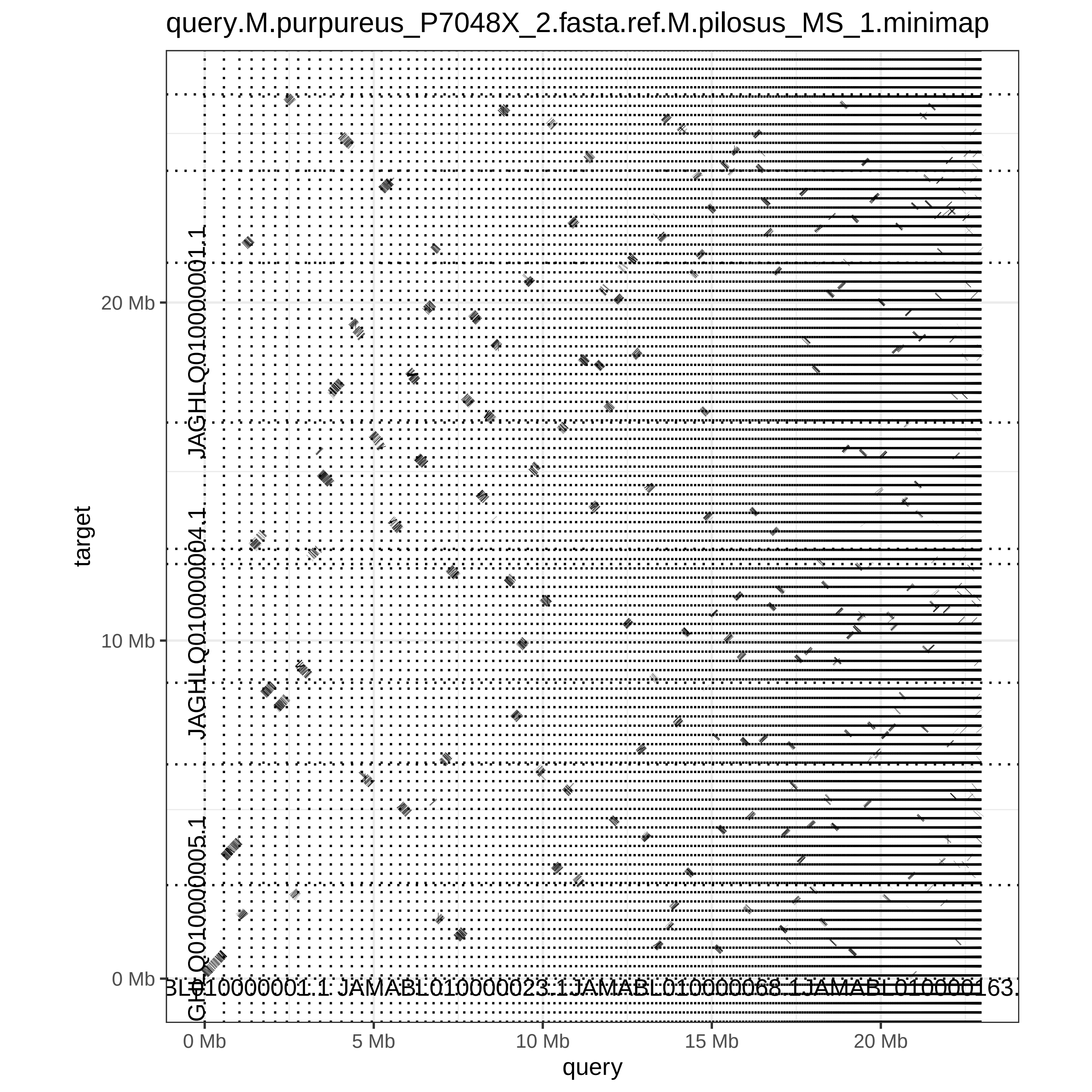
query.M.purpureus_P7048X_2.fasta.ref.M.pilosus_MS_1.minimap
target
20 Mb
10 Mb
0 Mb
JAGHLQ010000001.1
JAGHLQ010000004.1
JAGHLQ010000005.1
0 Mb
5 Mb
10 Mb
15 Mb
20 Mb
query
JAMABL010000023.1
JAMABL010000068.1

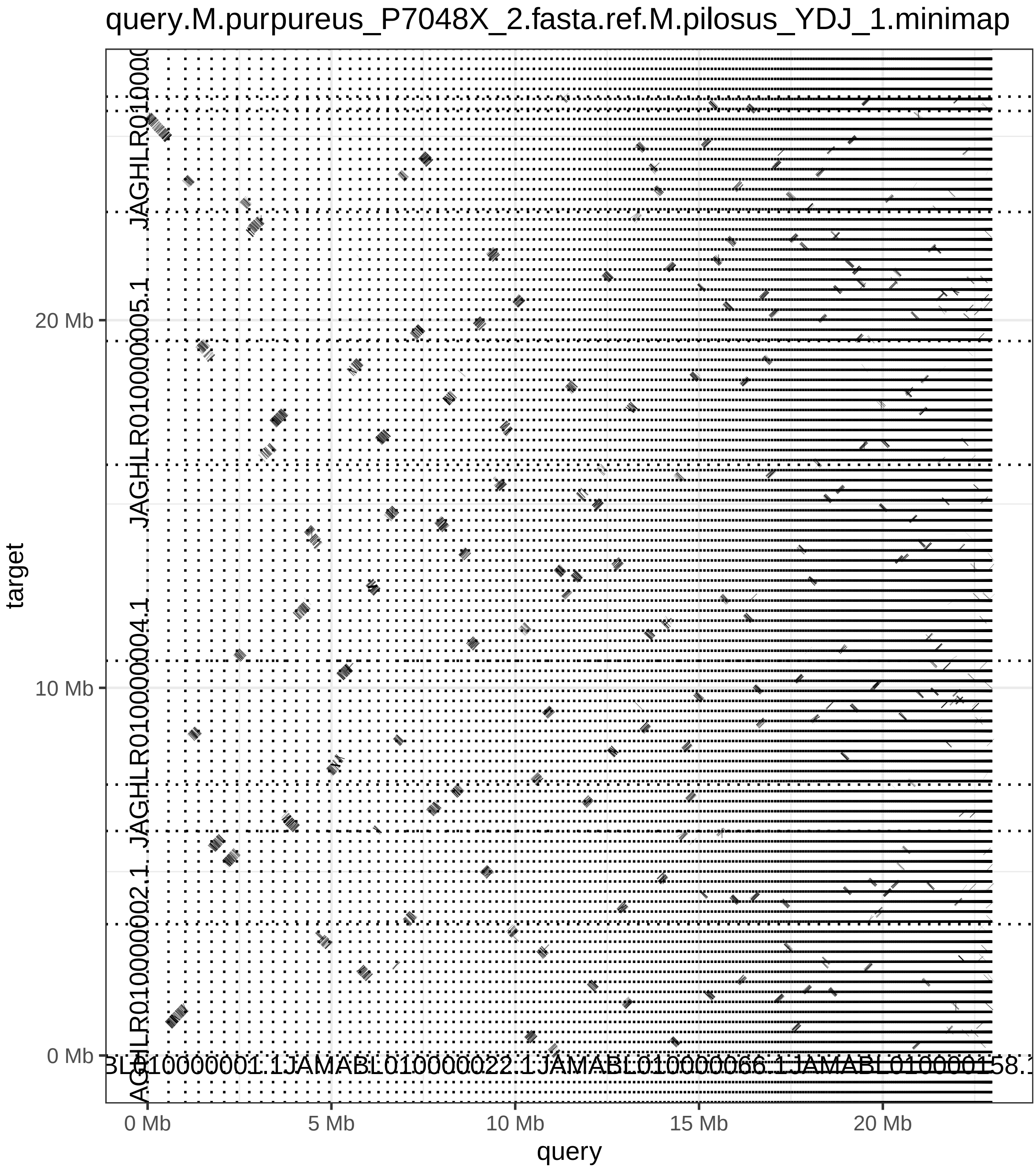
query.M.purpureus_P7048X_2.fasta.ref.M.pilosus_YDJ_1.minimap
target
query
0 Mb
10 Mb
20 Mb
0 Mb
5 Mb
10 Mb
15 Mb
20 Mb
JAGHLR010000005.1
JAGHLR010000004.1
JAGHLR010000002.1
JAMABL010000022.1
JAMABL010000066.1
JAMABL010000158.1

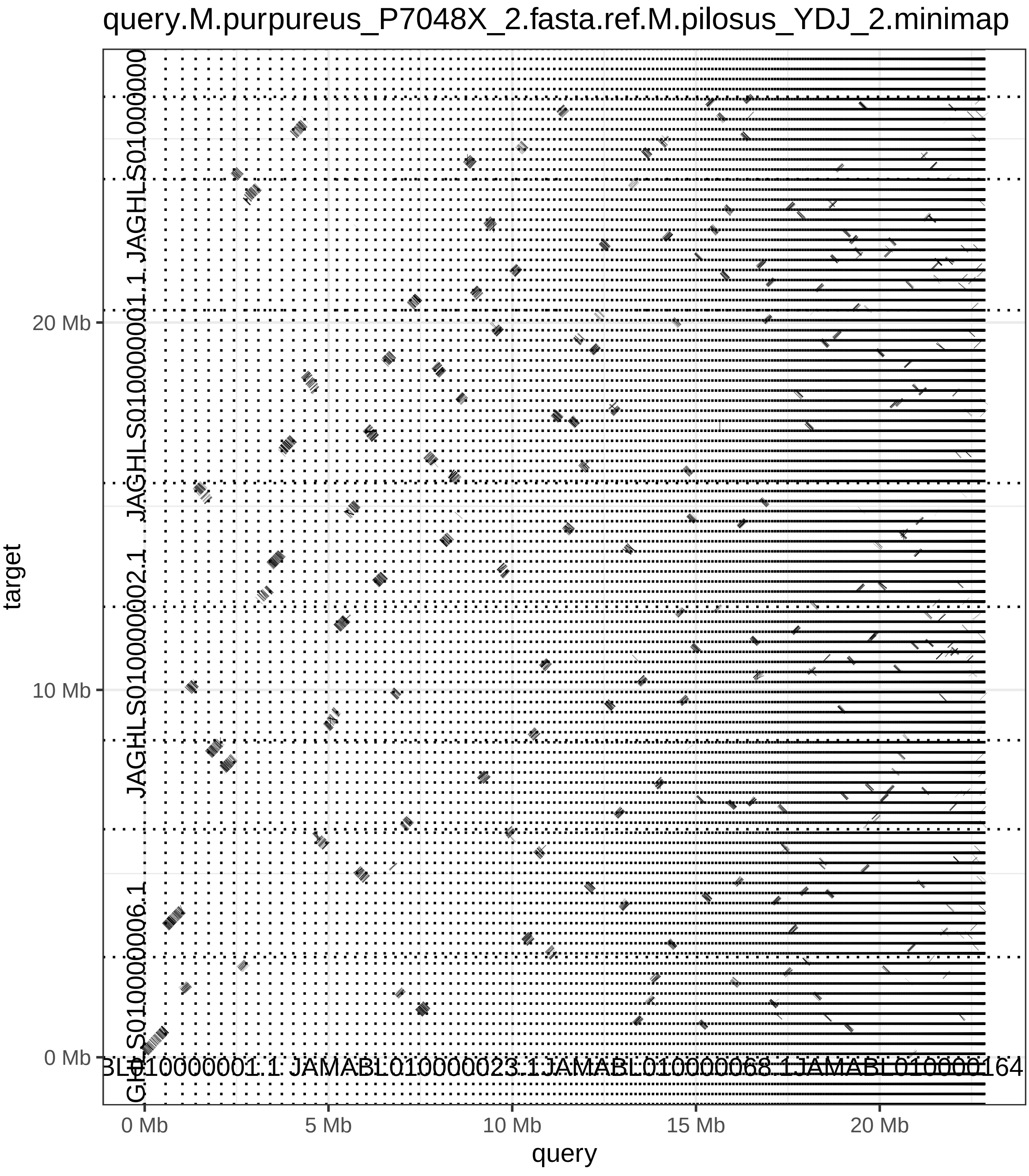
query.M.purpureus_P7048X_2.fasta.ref.M.pilosus_YDJ_2.minimap
target
20 Mb
10 Mb
0 Mb
0 Mb
5 Mb
10 Mb
15 Mb
20 Mb
query

query.M.purpureus_P7048X_2.fasta.ref.M.purpureus_CSU_M183.minimap

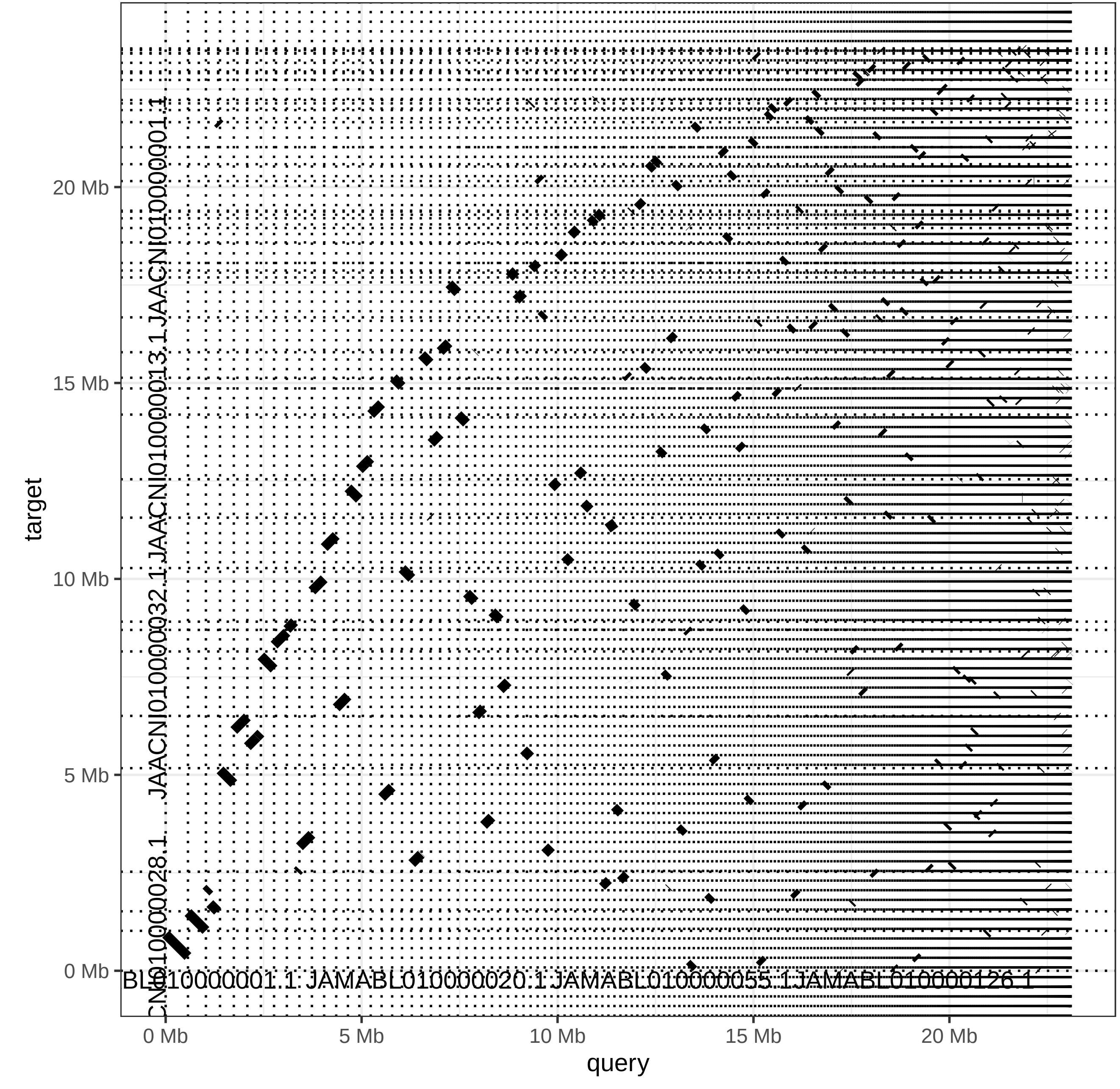

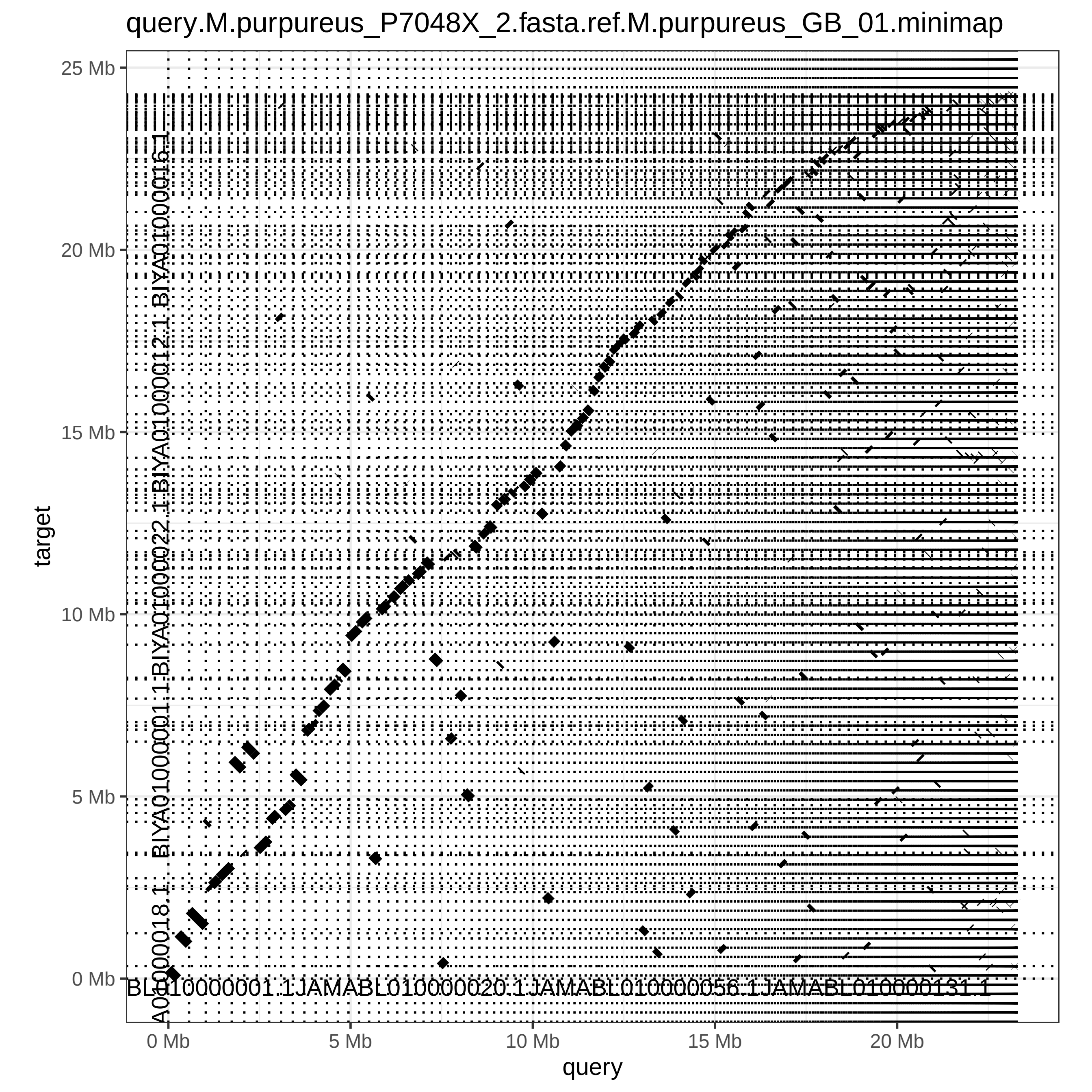

query.M.purpureus_P7048X_2.fasta.ref.M.purpureus_GB_01.minimap
target
query
0 Mb
5 Mb
10 Mb
15 Mb
20 Mb
25 Mb
BL010000001.1JAMABL010000020.1JAMABL010000056.1JAMABL010000131.1

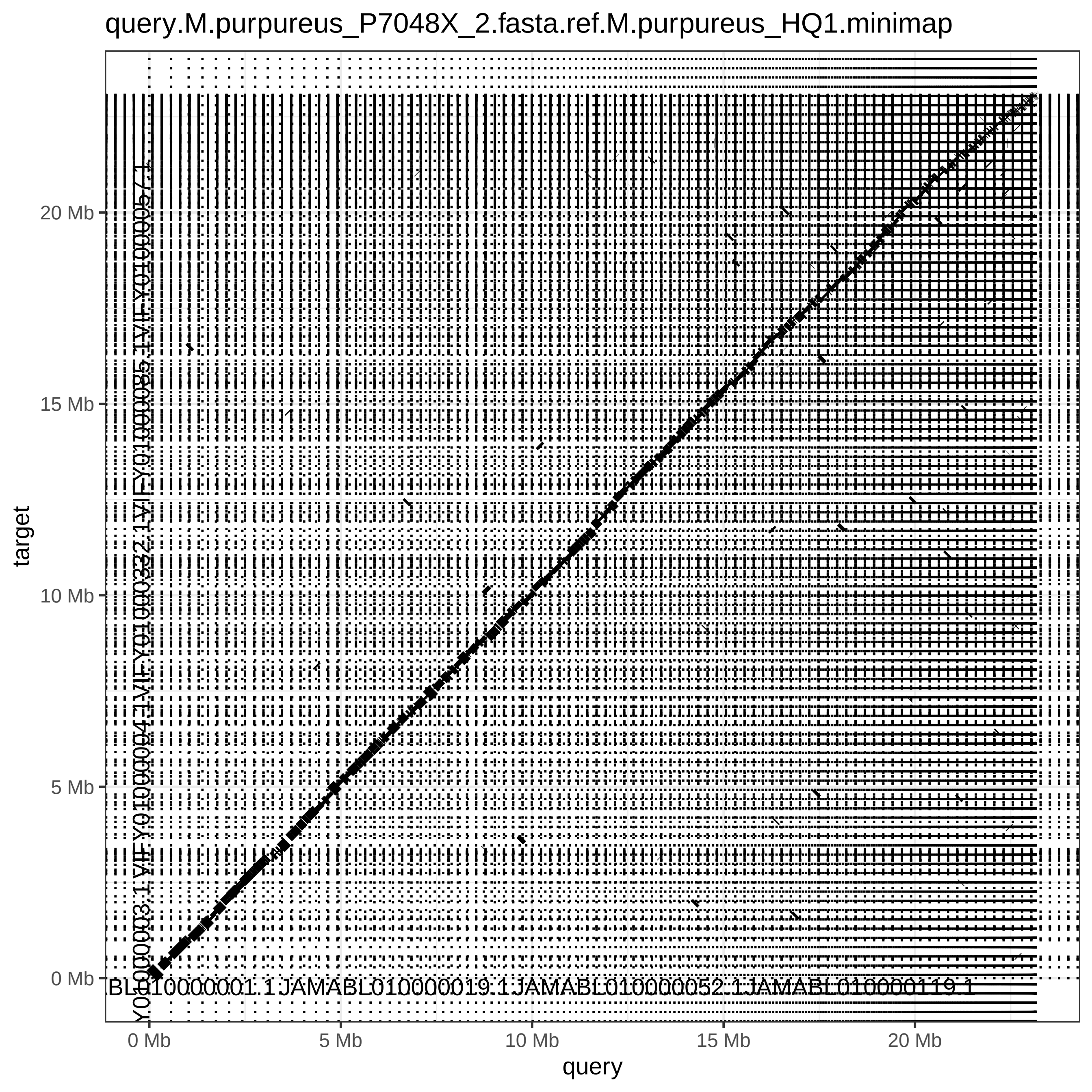
query.M.purpureus_P7048X_2.fasta.ref.M.purpureus_HQ1.minimap
target
20 Mb
15 Mb
10 Mb
5 Mb
0 Mb
JAMABL010000019.1
JAMABL010000052.1
JAMABL010000119.1
0 Mb
5 Mb
10 Mb
15 Mb
20 Mb
query

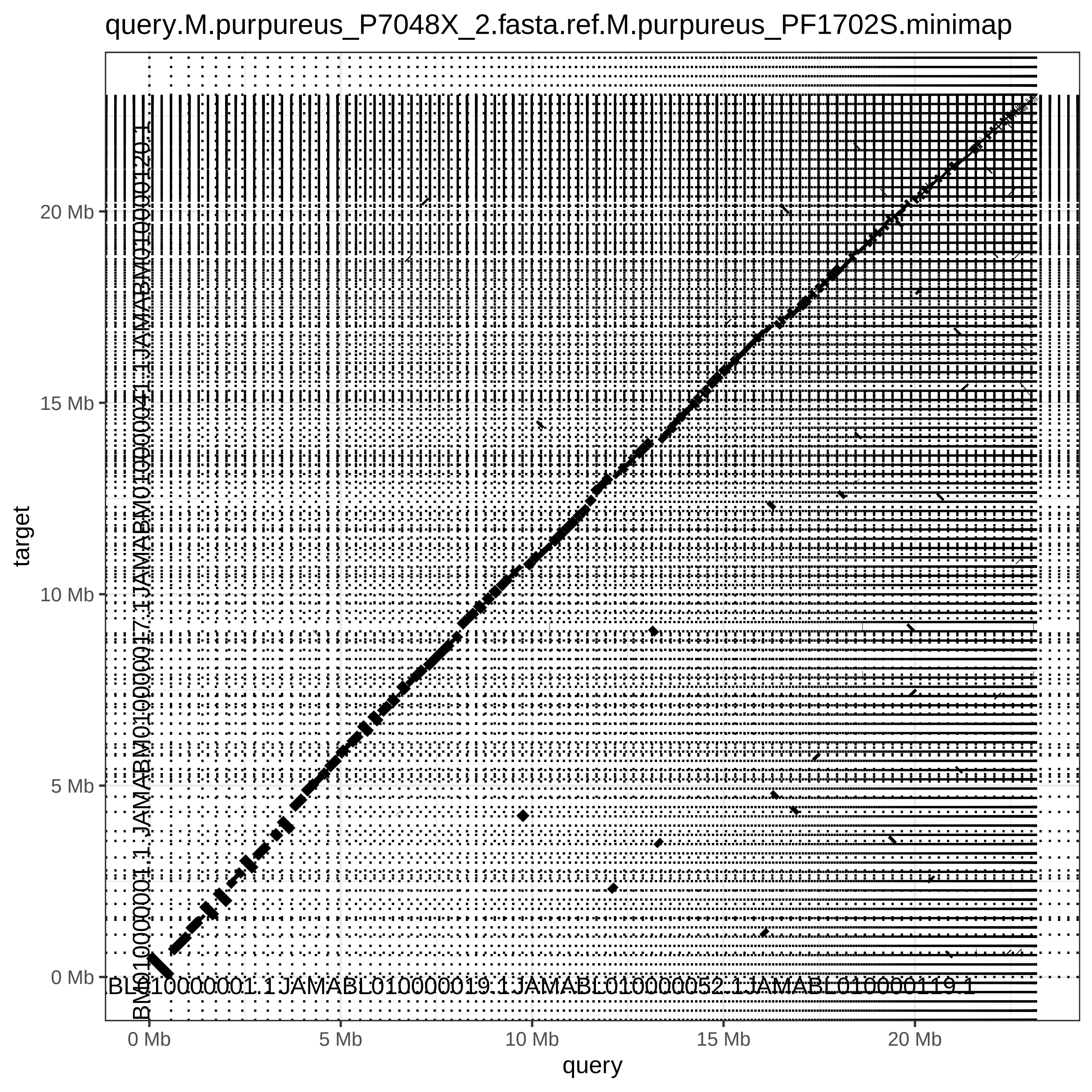

query.M.purpureus_P7048X_2.fasta.ref.M.purpureus_PF1702S.minimap
target
query
0 Mb
5 Mb
10 Mb
15 Mb
20 Mb
JAMABL010000019.1
JAMABL010000052.1
JAMABL010000119.1

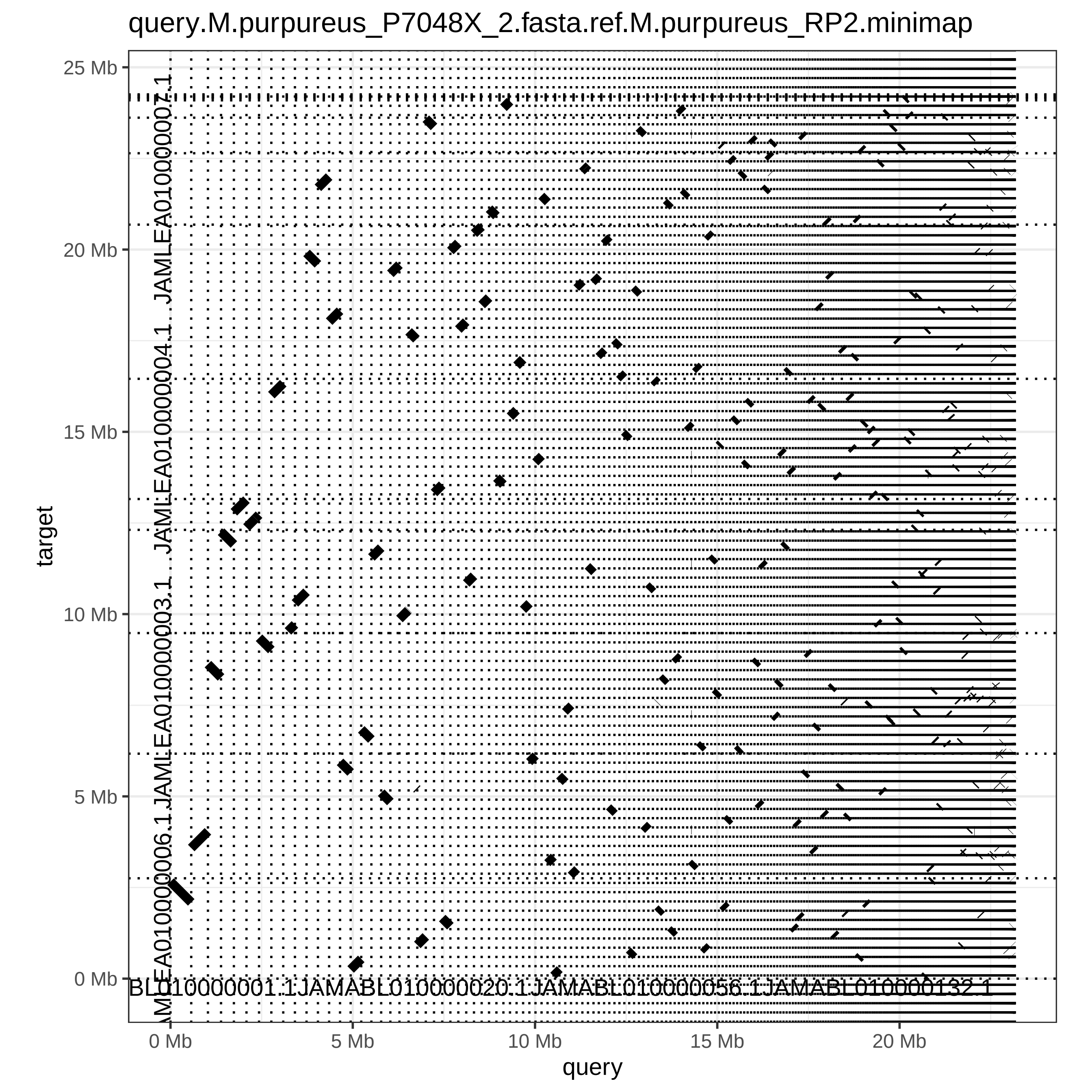
query.M.purpureus_P7048X_2.fasta.ref.M.purpureus_RP2.minimap
target
25 Mb
20 Mb
15 Mb
10 Mb
5 Mb
0 Mb
JAMLEA010000007.1
JAMLEA010000004.1
JAMLEA010000003.1
JAMLEA010000006.1
JAMLEA010000
BL010000001.1JAMABL010000020.1JAMABL010000056.1JAMABL010000132.1
0 Mb
5 Mb
10 Mb
15 Mb
20 Mb
query

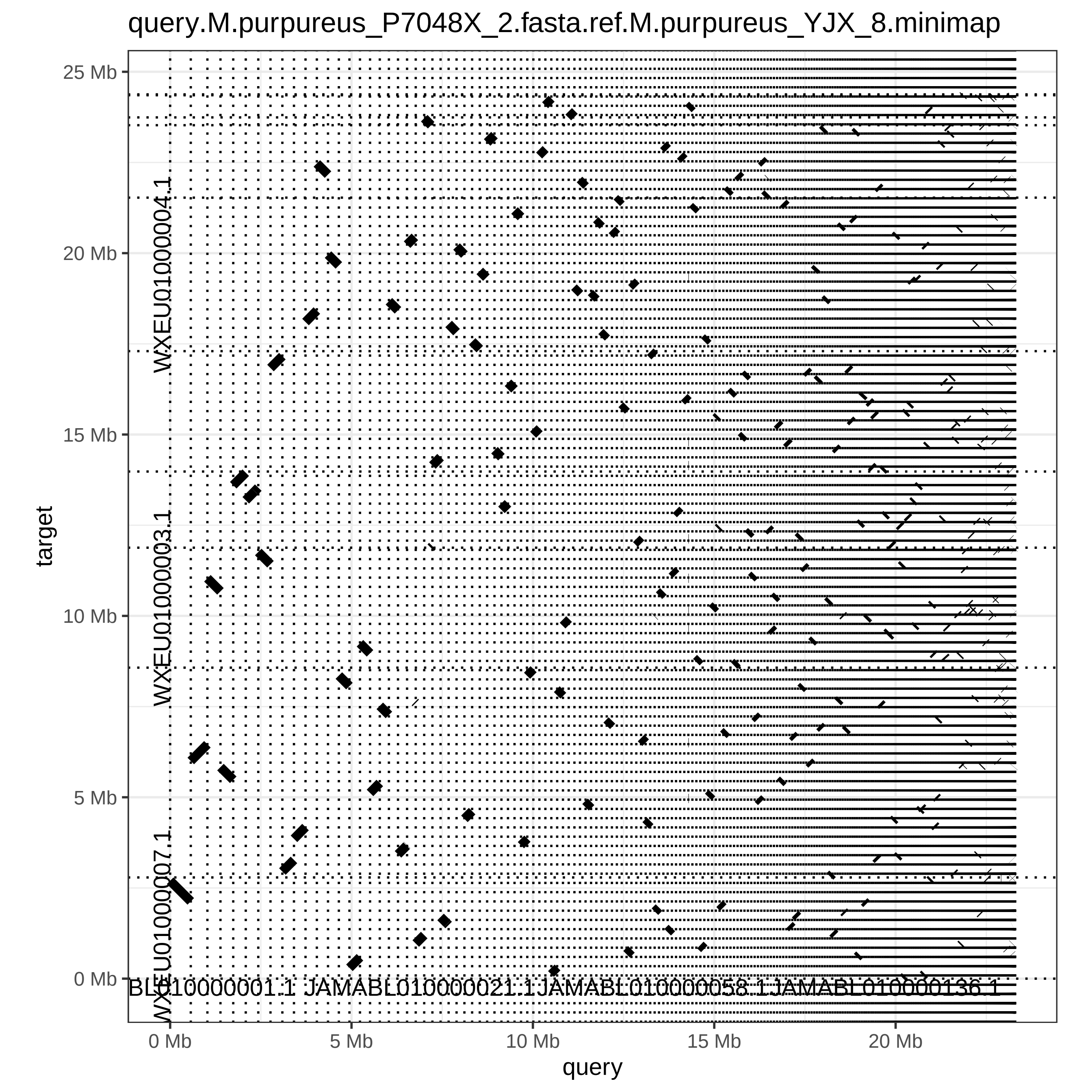
query.M.purpureus_P7048X_2.fasta.ref.M.purpureus_YJX_8.minimap
target
query
25 Mb
20 Mb
15 Mb
10 Mb
5 Mb
0 Mb
0 Mb
5 Mb
10 Mb
15 Mb
20 Mb
WXEU01000004.1
WXEU01000003.1
WXEU01000007.1
BL010000001.1
JAMABL010000021.1
JAMABL010000058.1
JAMABL010000136.1

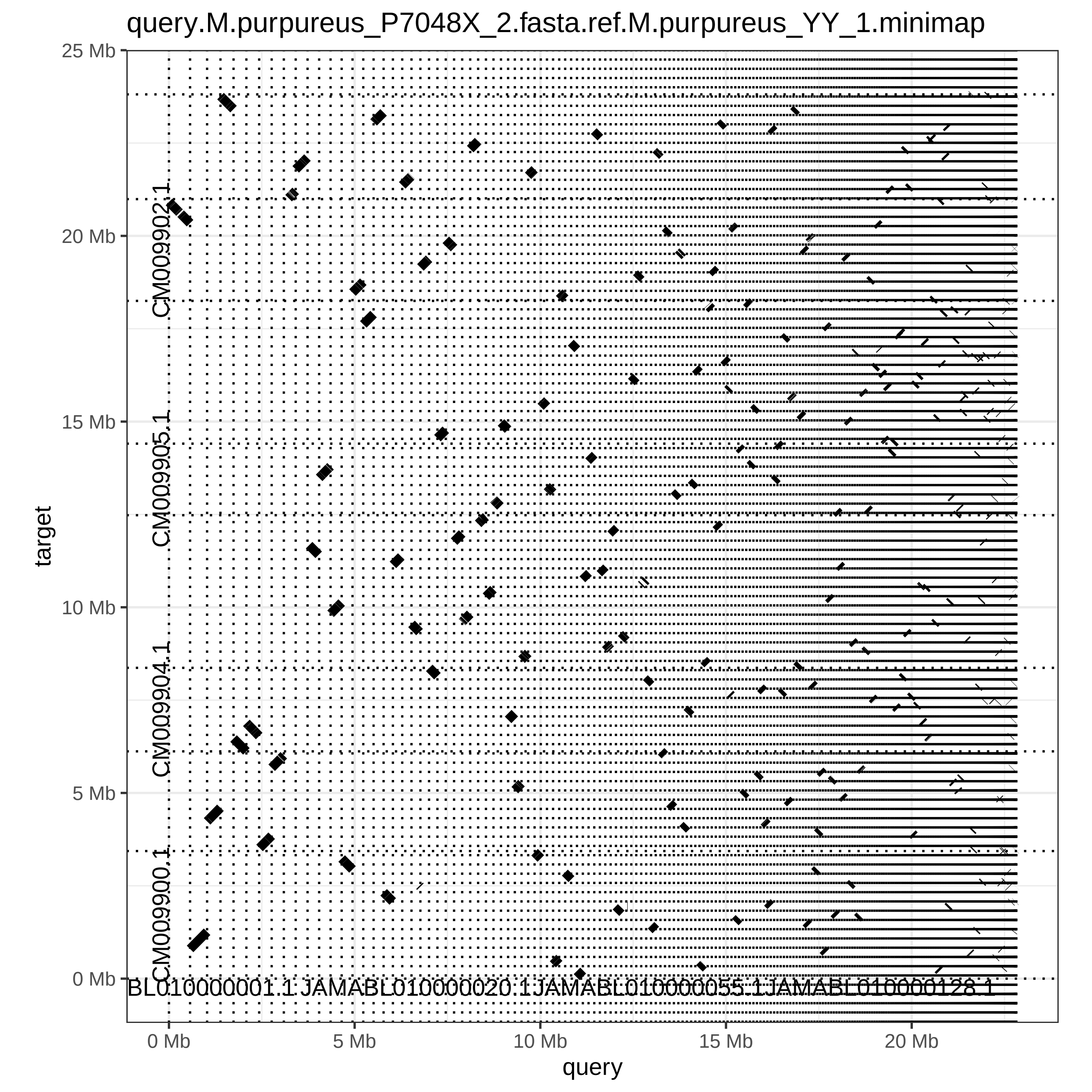
query.M.purpureus_P7048X_2.fasta.ref.M.purpureus_YY_1.minimap
target
25 Mb
20 Mb
15 Mb
10 Mb
5 Mb
0 Mb
CM009902.1
CM009905.1
CM009904.1
CM009900.1
BL010000001.1
JAMABL010000020.1
JAMABL010000055.1
JAMABL010000128.1
0 Mb
5 Mb
10 Mb
15 Mb
20 Mb
query

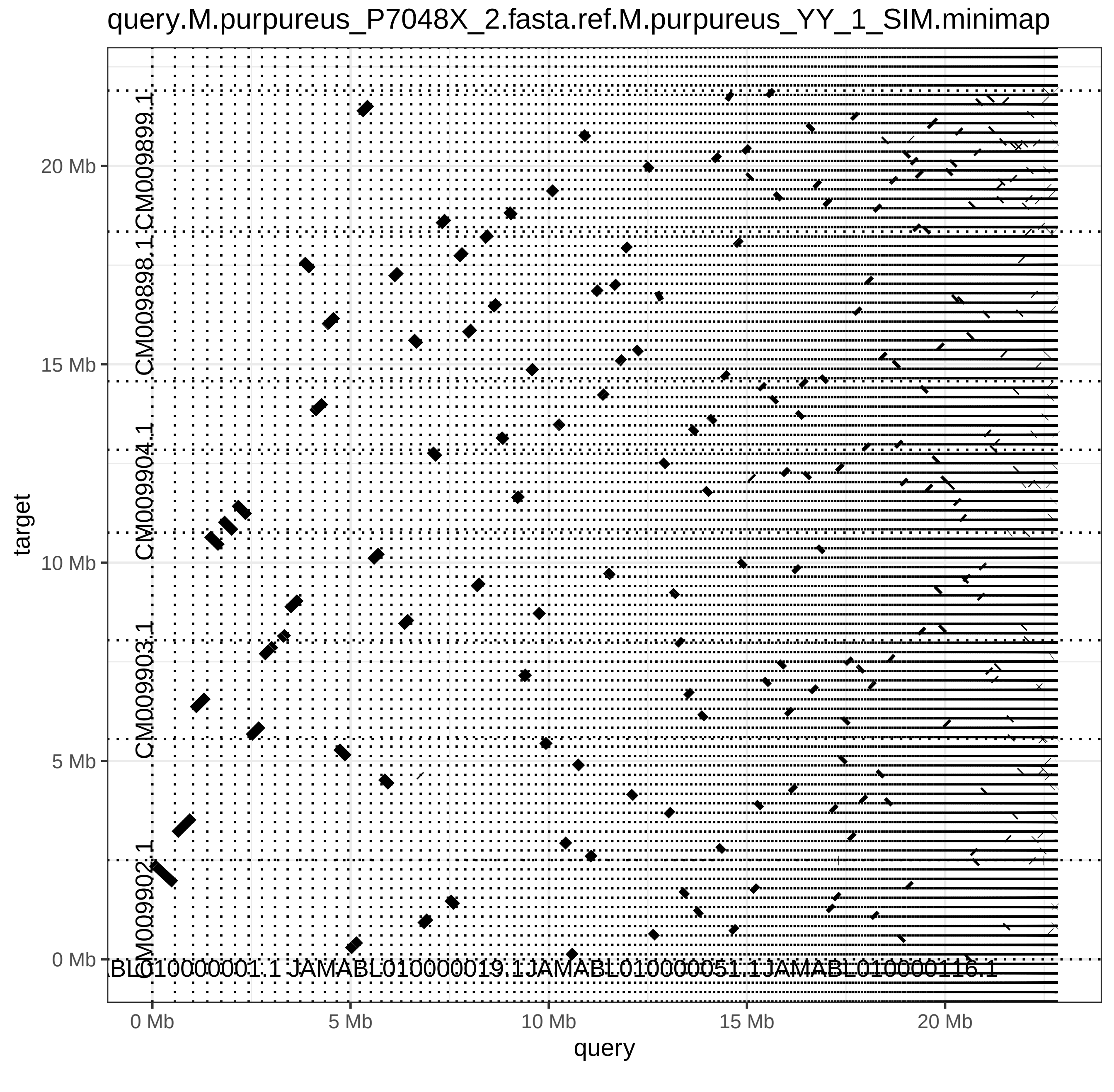
query.M.purpureus_P7048X_2.fasta.ref.M.purpureus_YY_1_SIM.minimap
target
query
0 Mb
5 Mb
10 Mb
15 Mb
20 Mb
CM009899.1
CM009898.1
CM009904.1
CM009903.1
CM009902.1
JAMABL010000019.1
JAMABL010000051.1
JAMABL010000116.1

query.M.purpureus_P7048X_2.fasta.ref.M.ruber_FWB13.minimap

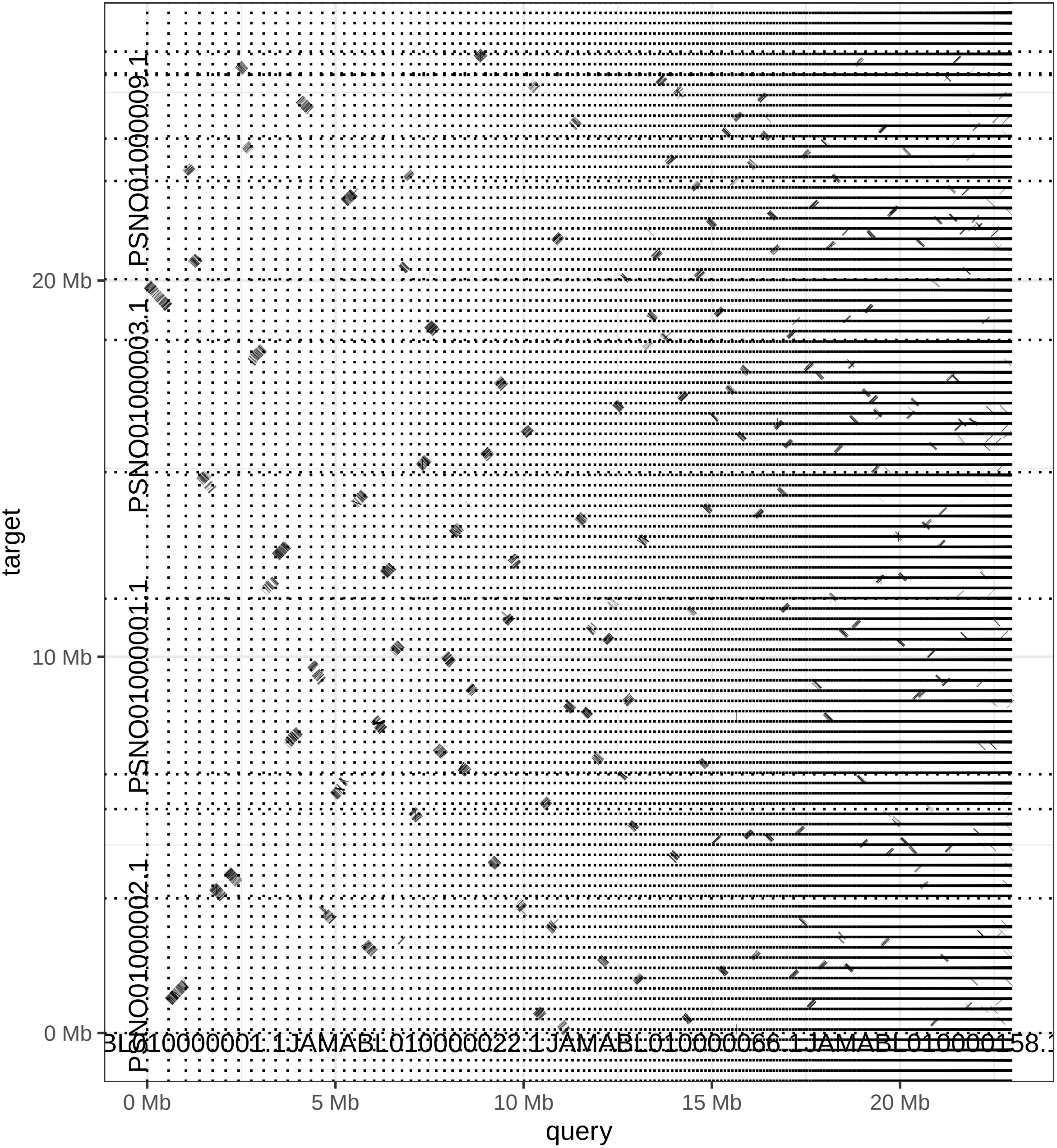

query.M.purpureus_P7048X_2.fasta.ref.M.ruber_GA.minimap

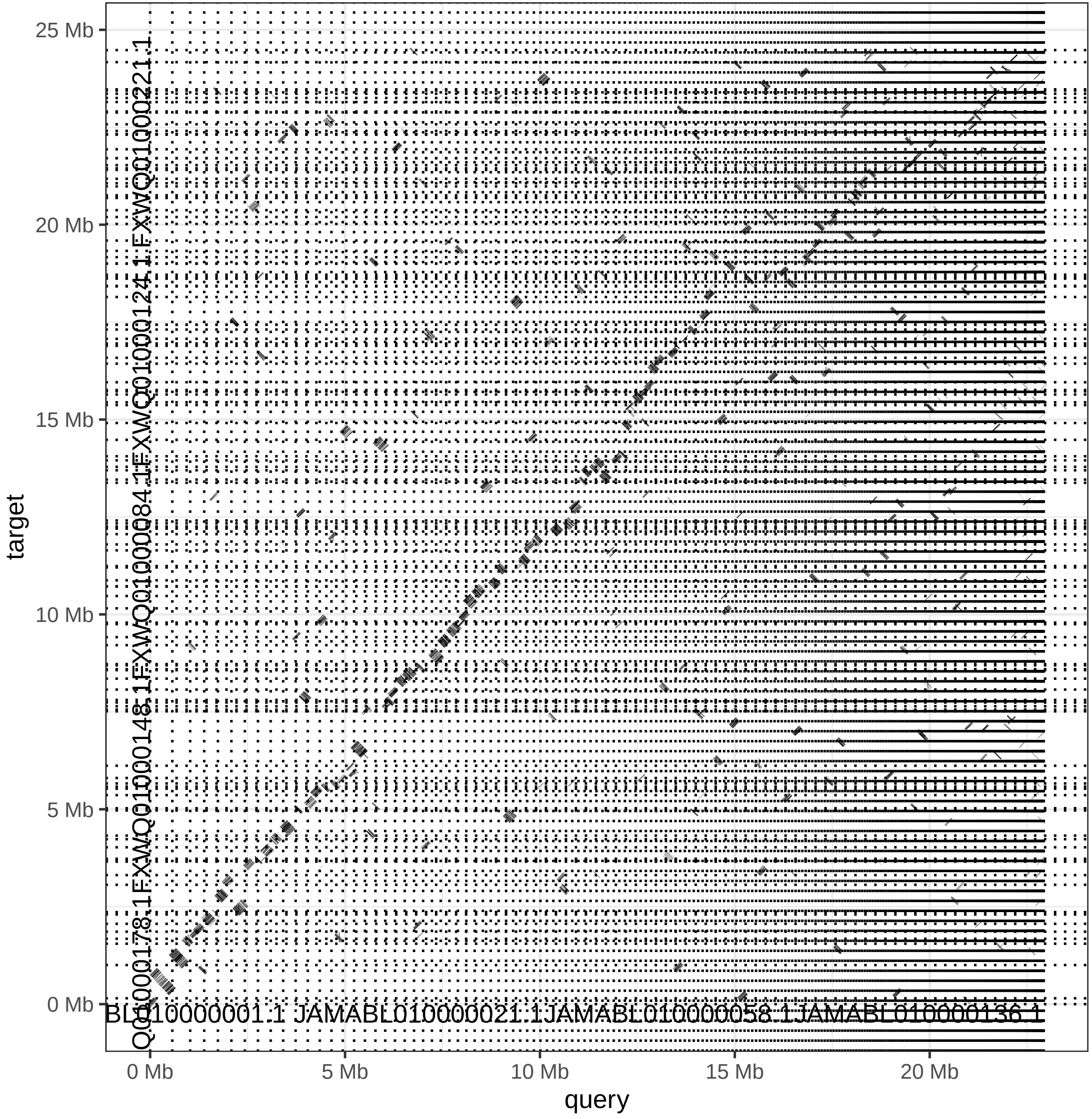

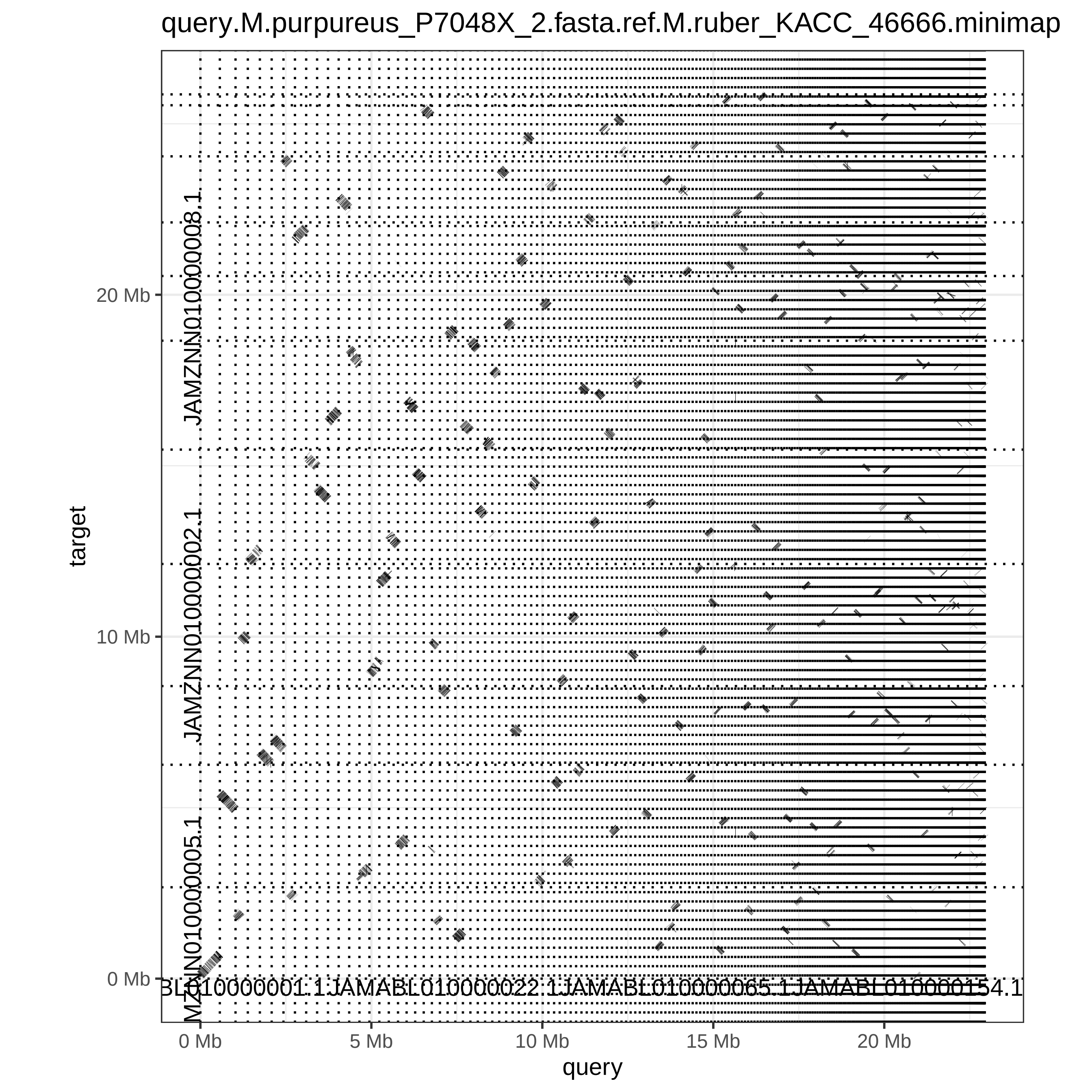

query.M.purpureus_P7048X_2.fasta.ref.M.ruber_KACC_46666.minimap
target
20 Mb
10 Mb
0 Mb
JAMZNN010000008.1
JAMZNN010000002.1
JAMZNN010000005.1
BL010000001.1JAMABL010000022.1JAMABL010000065.1JAMABL010000154.1
0 Mb
5 Mb
10 Mb
15 Mb
20 Mb
query

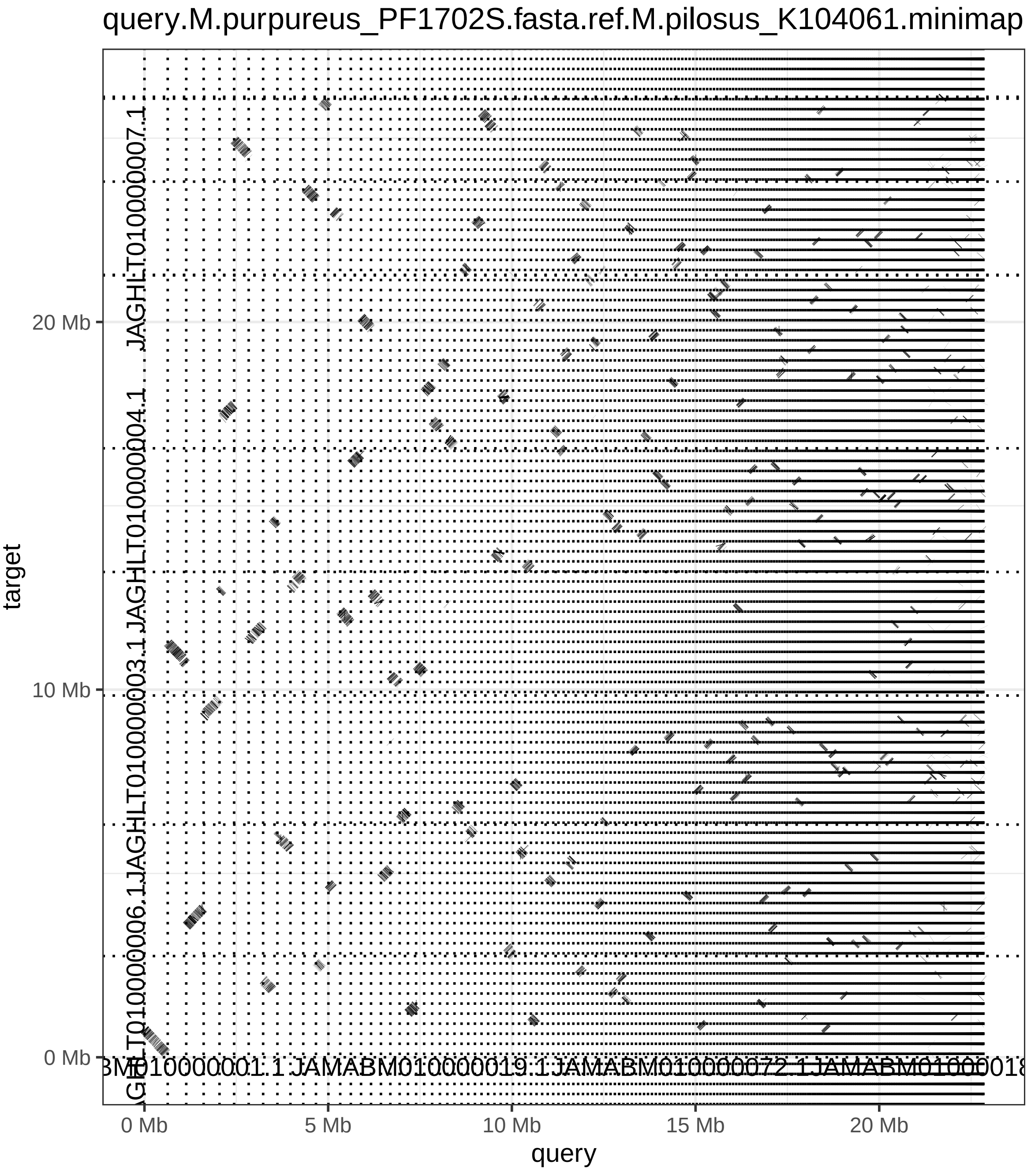

query.M.purpureus_PF1702S.fasta.ref.M.pilosus_K104061.minimap
target
query
0 Mb
5 Mb
10 Mb
15 Mb
20 Mb
0 Mb
10 Mb
20 Mb
JAGHLT010000007.1
JAGHLT010000004.1
JAGHLT010000003.1
JAGHLT010000006.1
JAMABM010000019.1
JAMABM010000072.1

query.M.purpureus_PF1702S.fasta.ref.M.pilosus_MS_1.minimap

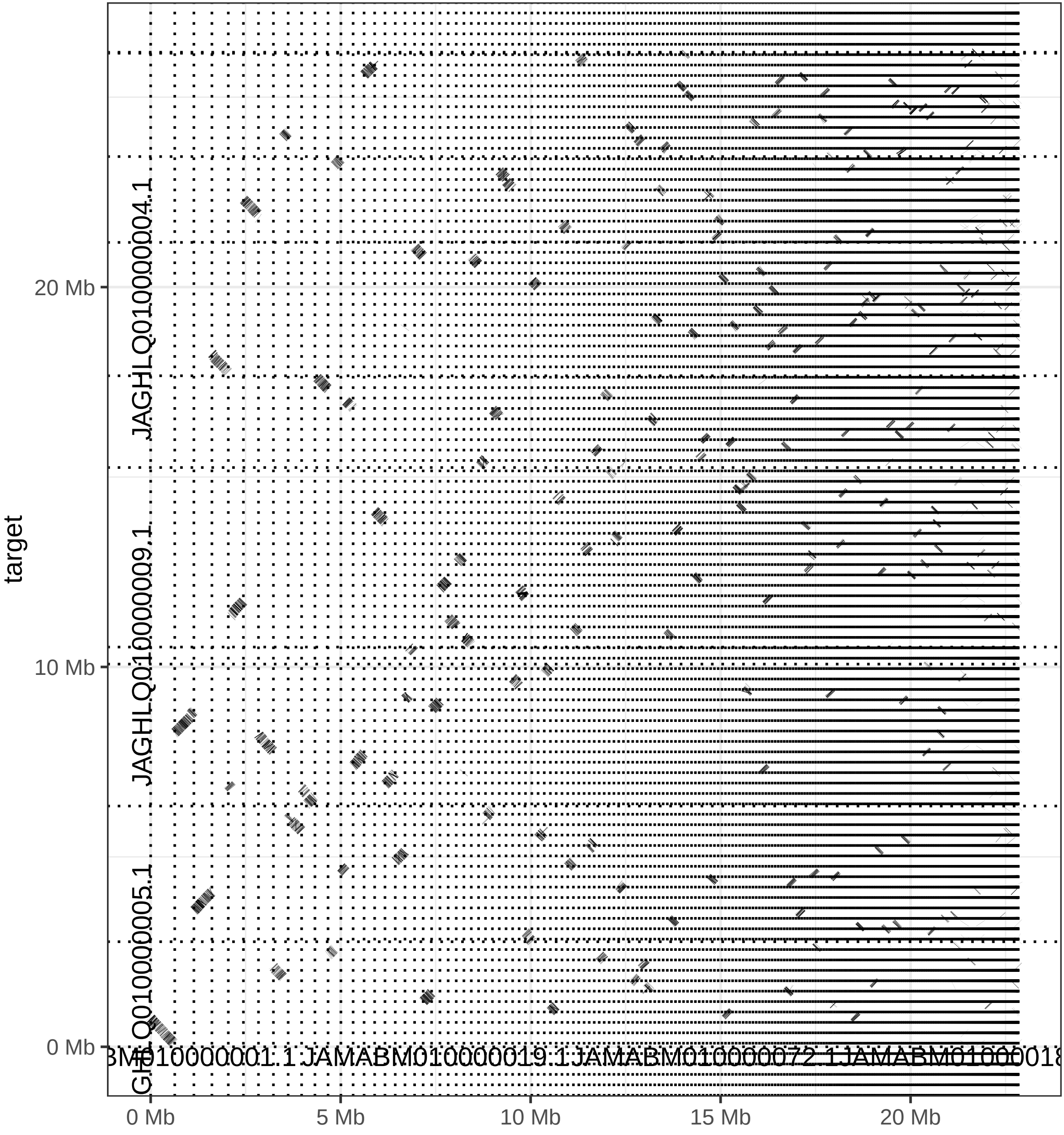

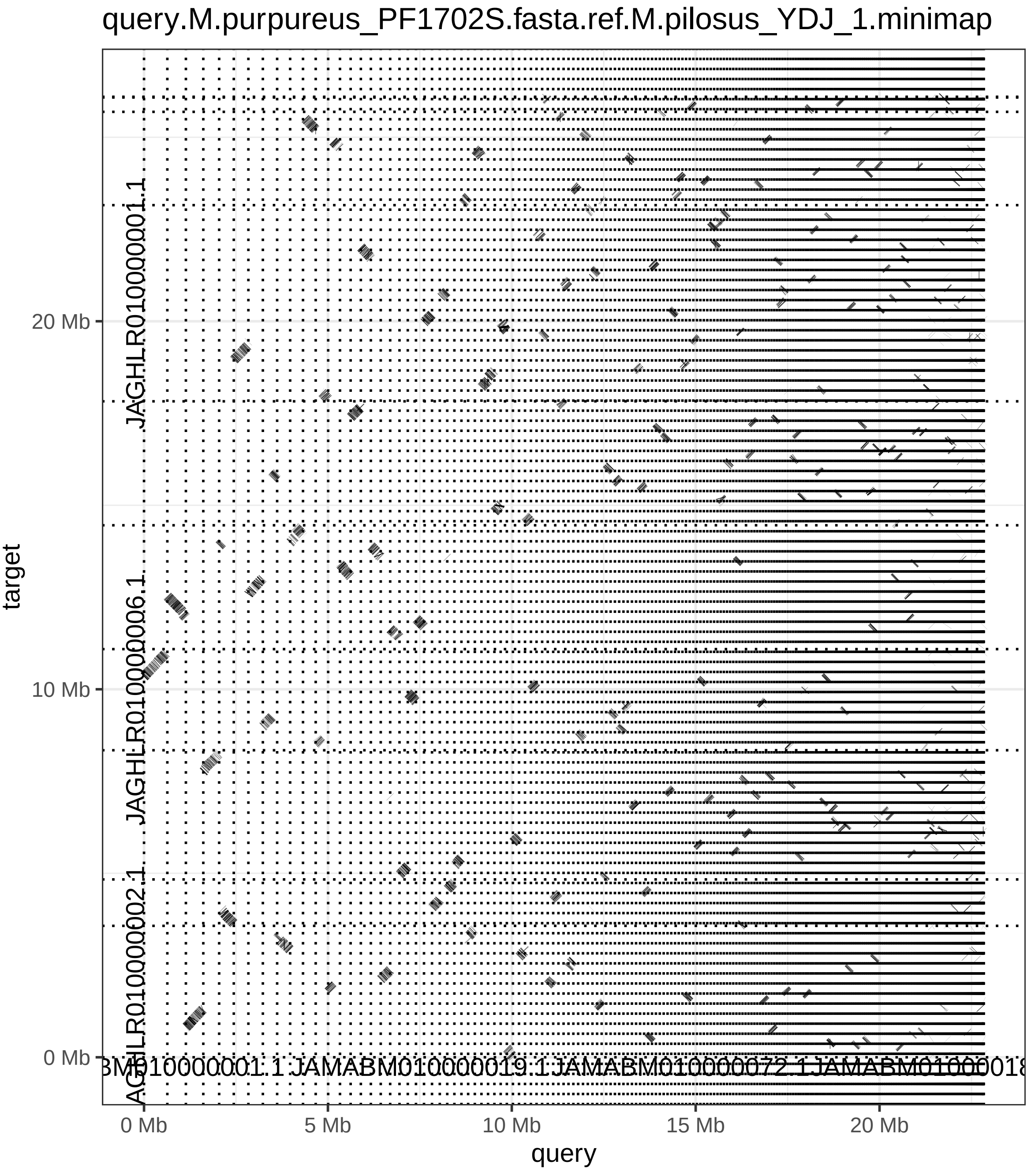
query.M.purpureus_PF1702S.fasta.ref.M.pilosus_YDJ_1.minimap
target
query
0 Mb
10 Mb
20 Mb
0 Mb
5 Mb
10 Mb
15 Mb
20 Mb
JAGHLR010000001.1
JAGHLR010000006.1
JAGHLR010000002.1
JAMABM010000019.1
JAMABM010000072.1

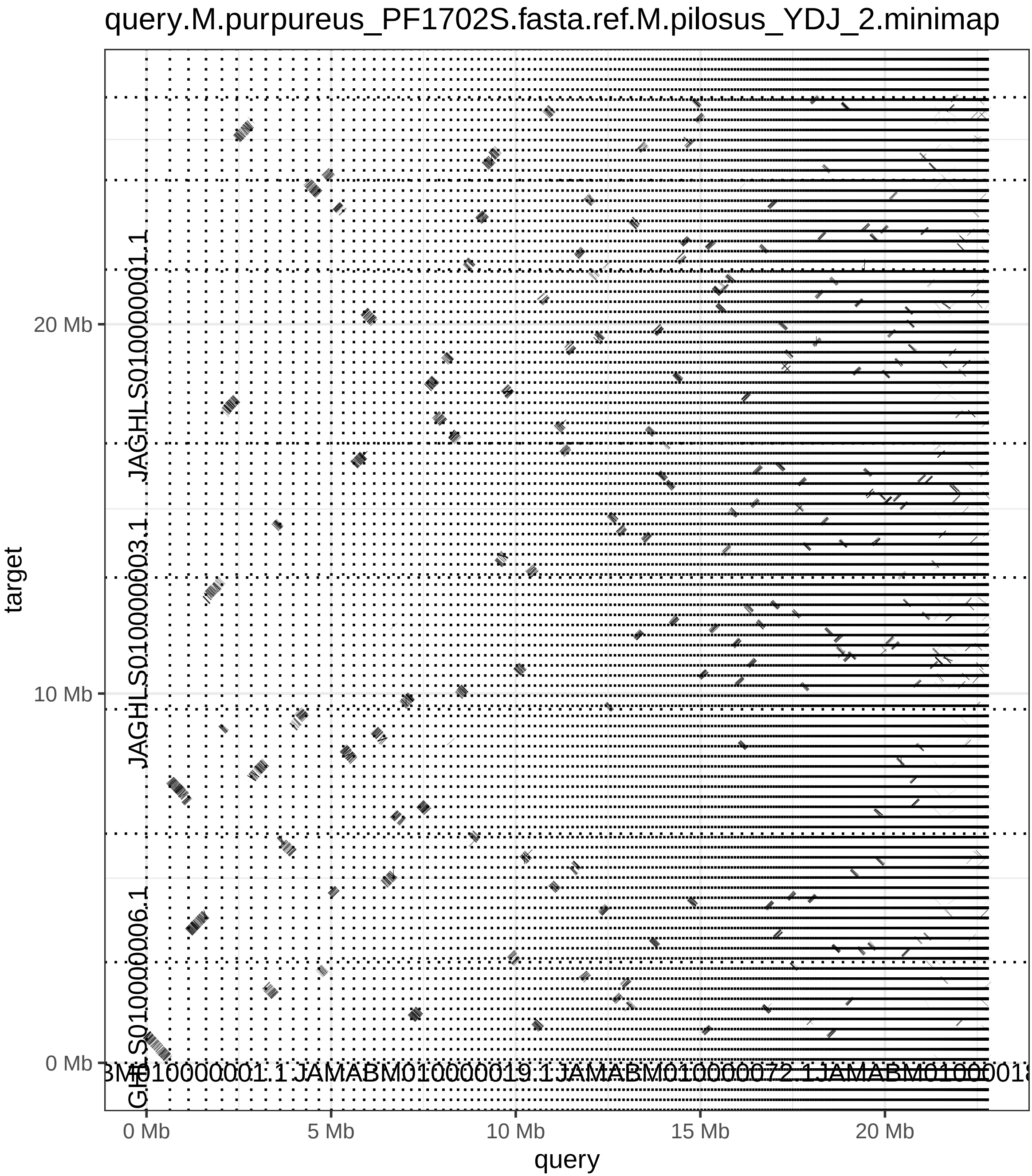
query.M.purpureus_PF1702S.fasta.ref.M.pilosus_YDJ_2.minimap
target
query
0 Mb
5 Mb
10 Mb
15 Mb
20 Mb
10 Mb
20 Mb
JAGHLS010000001.1
JAGHLS010000003.1
JAGHLS010000006.1
JAMABM010000019.1
JAMABM010000072.1

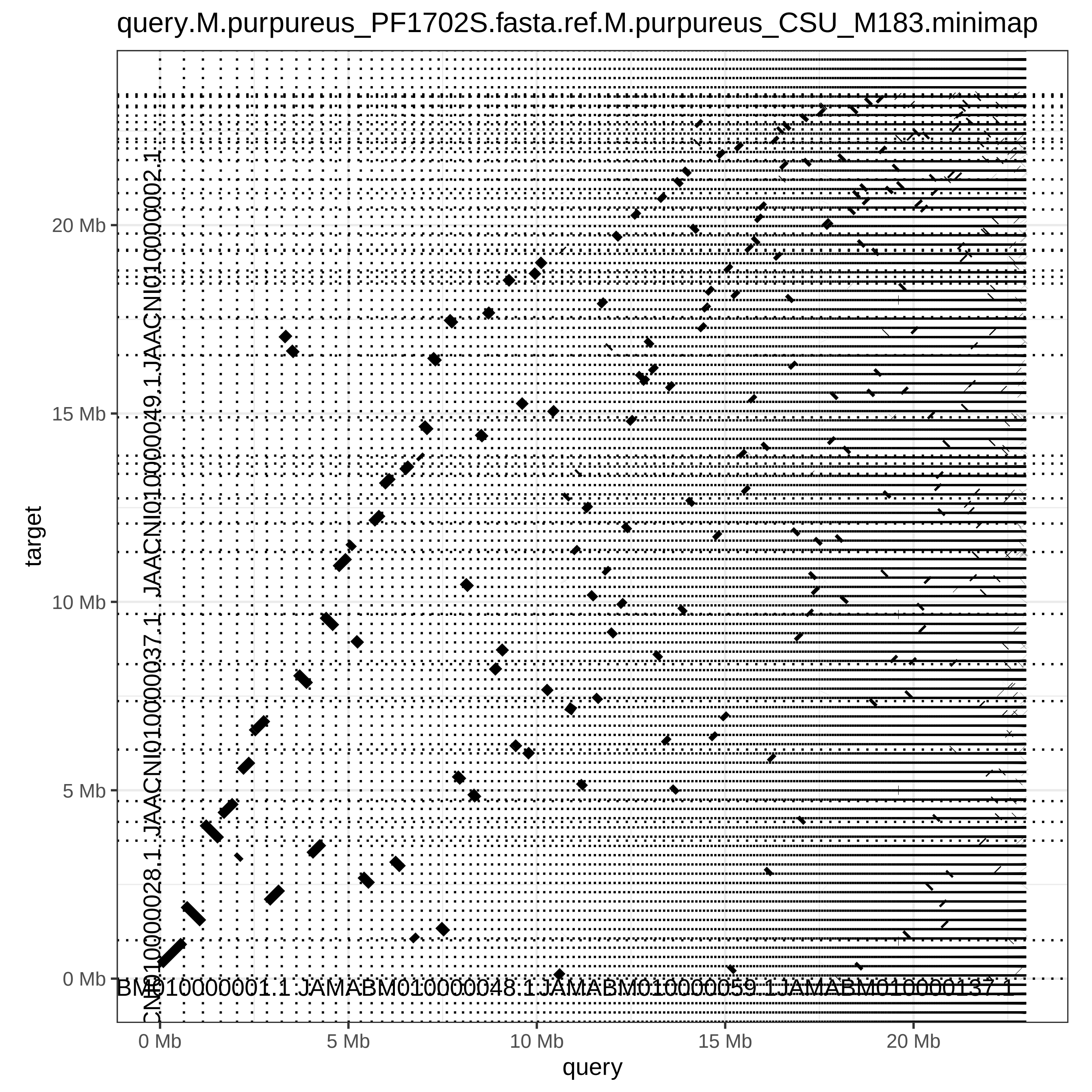

query.M.purpureus_PF1702S.fasta.ref.M.purpureus_CSU_M183.minimap
target
query
0 Mb
5 Mb
10 Mb
15 Mb
20 Mb

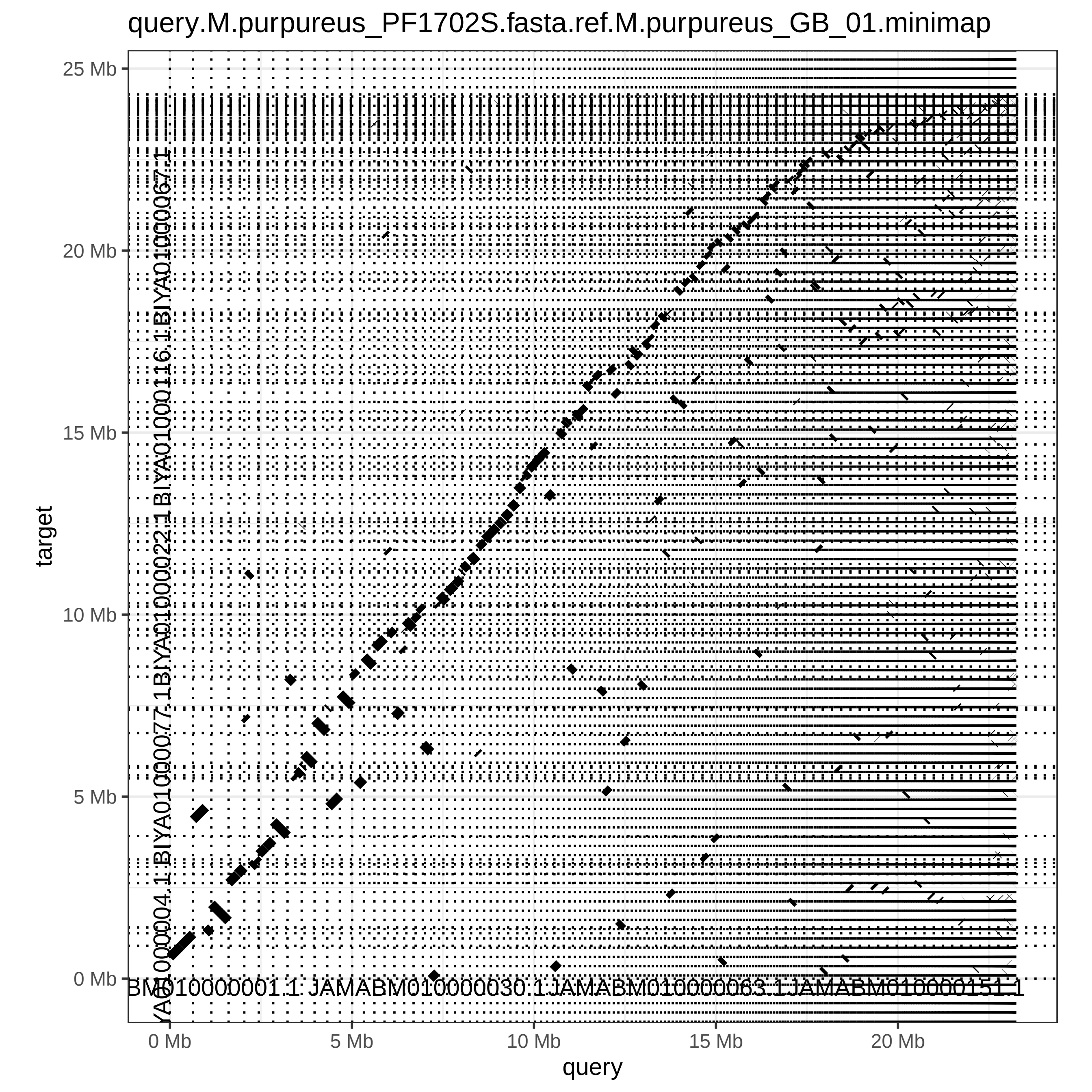
query.M.purpureus_PF1702S.fasta.ref.M.purpureus_GB_01.minimap
target
query
0 Mb
5 Mb
10 Mb
15 Mb
20 Mb
25 Mb
JAMABM010000030.1
JAMABM010000063.1
JAMABM010000151.1

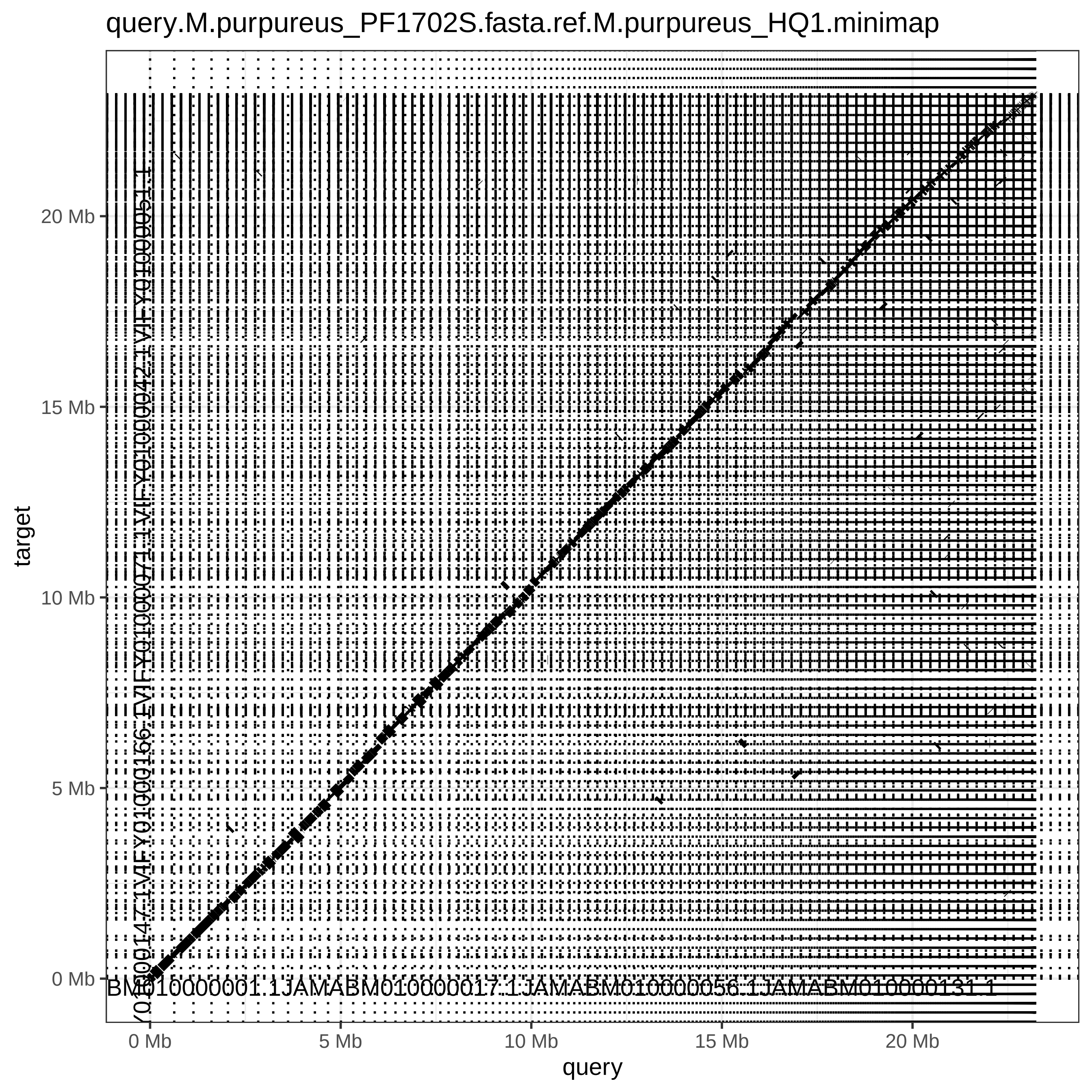
query.M.purpureus_PF1702S.fasta.ref.M.purpureus_HQ1.minimap
20 Mb
15 Mb
10 Mb
5 Mb
0 Mb
target
BM010000001.1JAMABM010000017.1JAMABM010000056.1JAMABM010000131.1
0 Mb
5 Mb
10 Mb
15 Mb
20 Mb
query

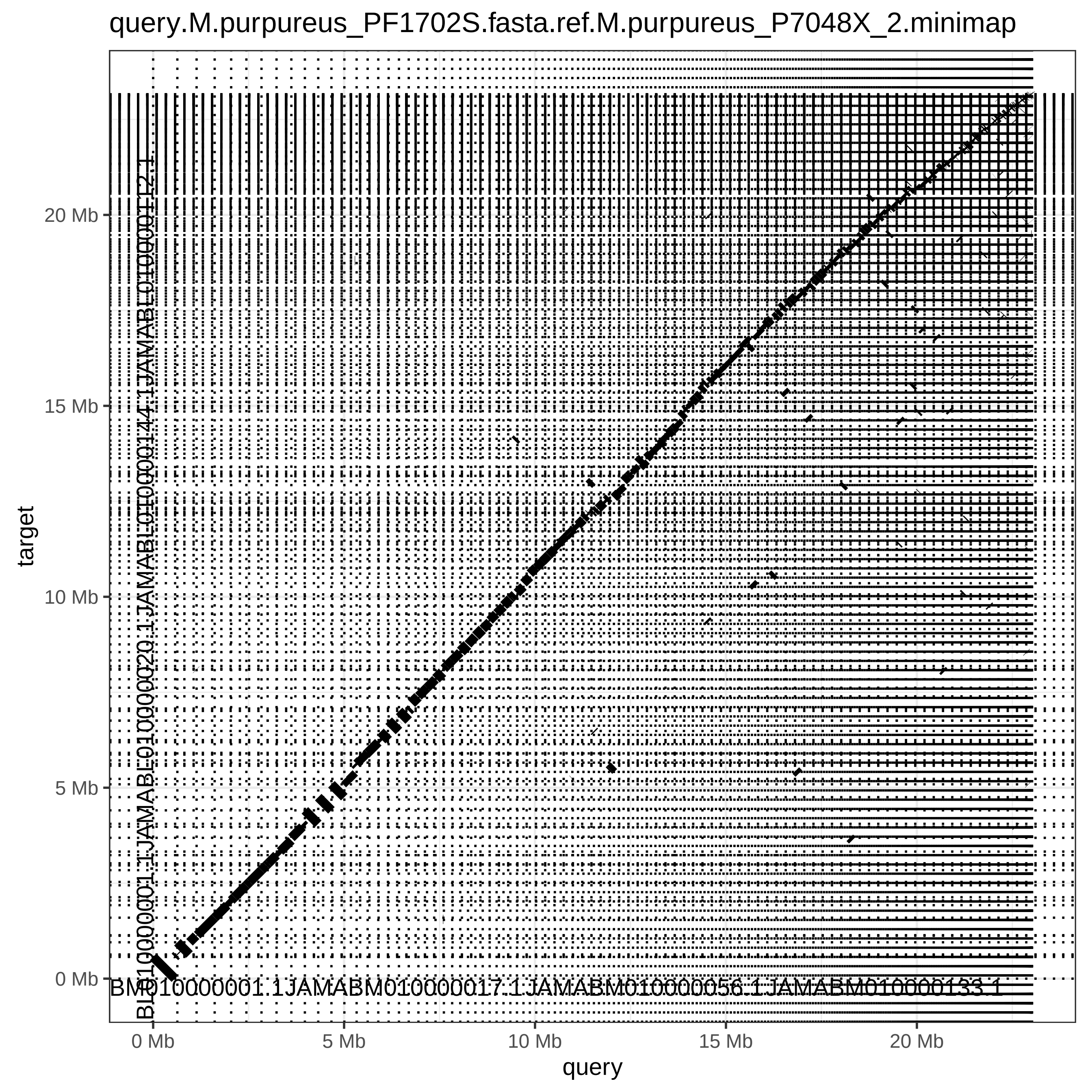
query.M.purpureus_PF1702S.fasta.ref.M.purpureus_P7048X_2.minimap
target
20 Mb
15 Mb
10 Mb
5 Mb
0 Mb
query
0 Mb
5 Mb
10 Mb
15 Mb
20 Mb
BM010000001.1JAMABM010000017.1JAMABM010000056.1JAMABM010000133.1
BL010000001.1JAMABL010000020.1JAMABL010000144.1JAMABL010000121.1

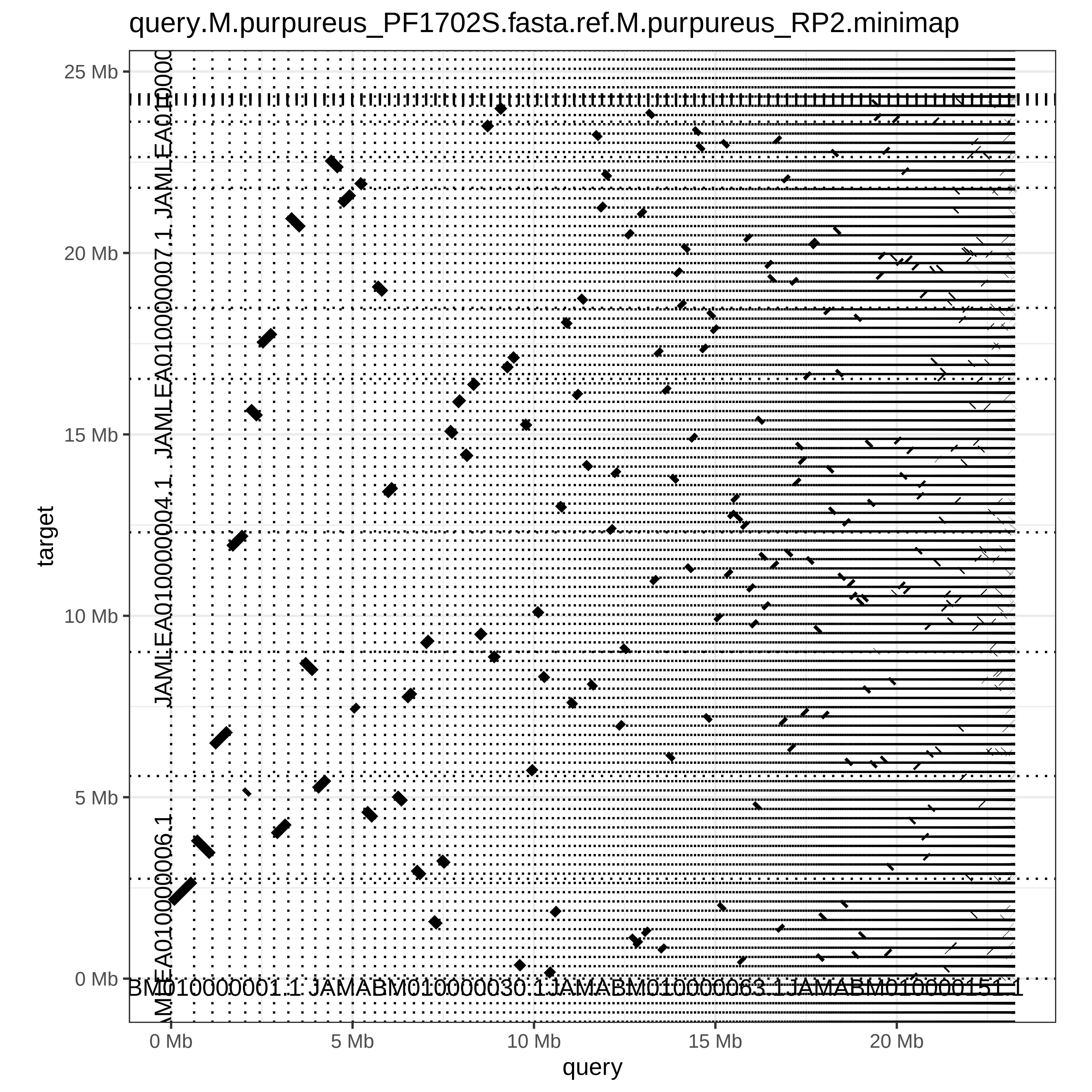
query.M.purpureus_PF1702S.fasta.ref.M.purpureus_RP2.minimap
target
25 Mb
20 Mb
15 Mb
10 Mb
5 Mb
0 Mb
JAMLEA010000007.1
JAMLEA010000004.1
JAMLEA010000006.1
BM010000001.1
JAMABM010000030.1
JAMABM010000063.1
JAMABM010000151.1
0 Mb
5 Mb
10 Mb
15 Mb
20 Mb
query

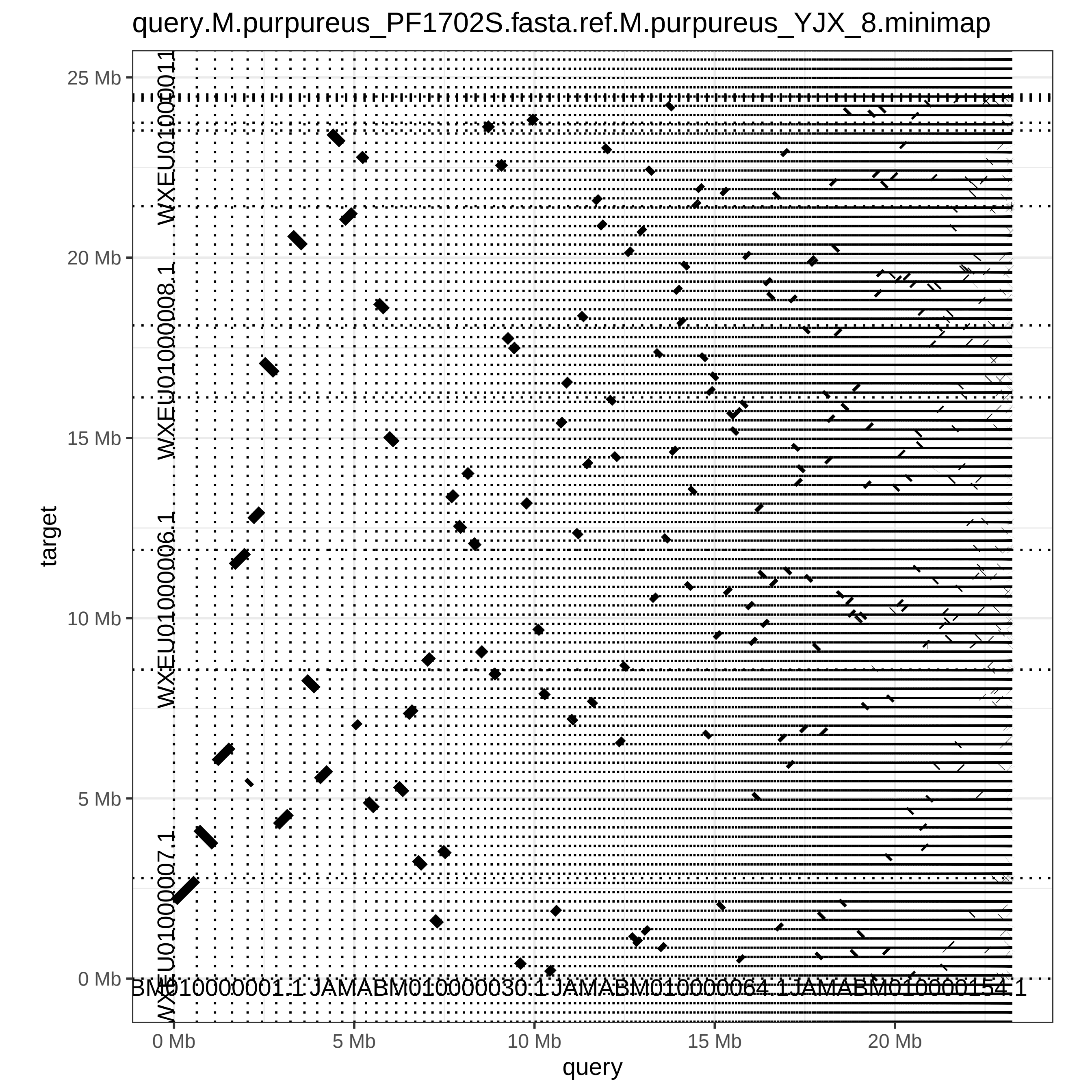

query.M.purpureus_PF1702S.fasta.ref.M.purpureus_YJX_8.minimap
target
query
25 Mb
20 Mb
15 Mb
10 Mb
5 Mb
0 Mb
0 Mb
5 Mb
10 Mb
15 Mb
20 Mb
WXEU01000011.1
WXEU01000008.1
WXEU01000006.1
WXEU01000007.1
JAMABM010000030.1
JAMABM010000064.1
JAMABM010000154.1

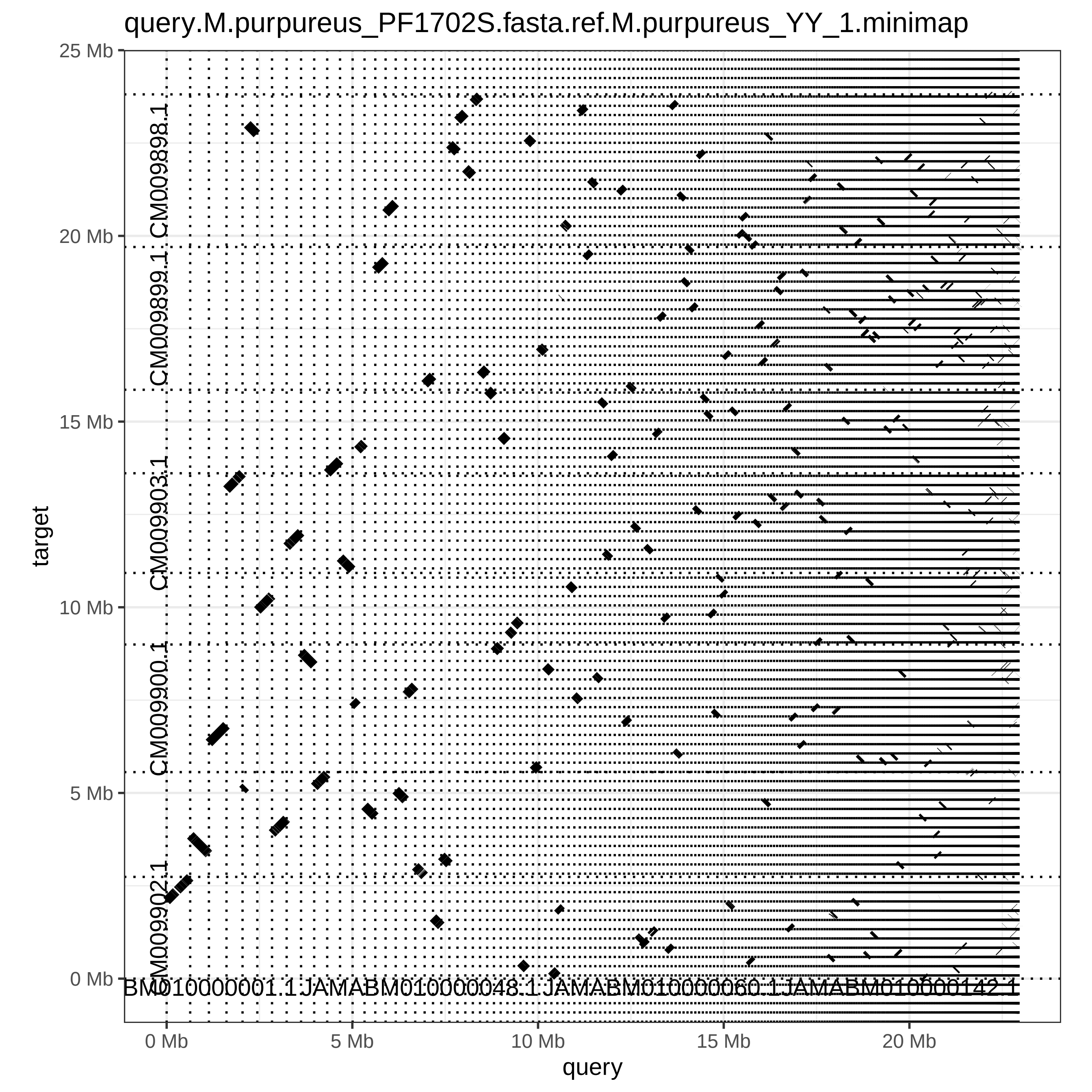

query.M.purpureus_PF1702S.fasta.ref.M.purpureus_YY_1.minimap
target
25 Mb
20 Mb
15 Mb
10 Mb
5 Mb
0 Mb
CM009898.1
CM009899.1
CM009903.1
CM009900.1
CM009902.1
BM010000001.1
JAMABM010000048.1
JAMABM010000060.1
JAMABM010000142.1
0 Mb
5 Mb
10 Mb
15 Mb
20 Mb
query

query.M.purpureus_PF1702S.fasta.ref.M.purpureus_YY_1_SIM.minimap

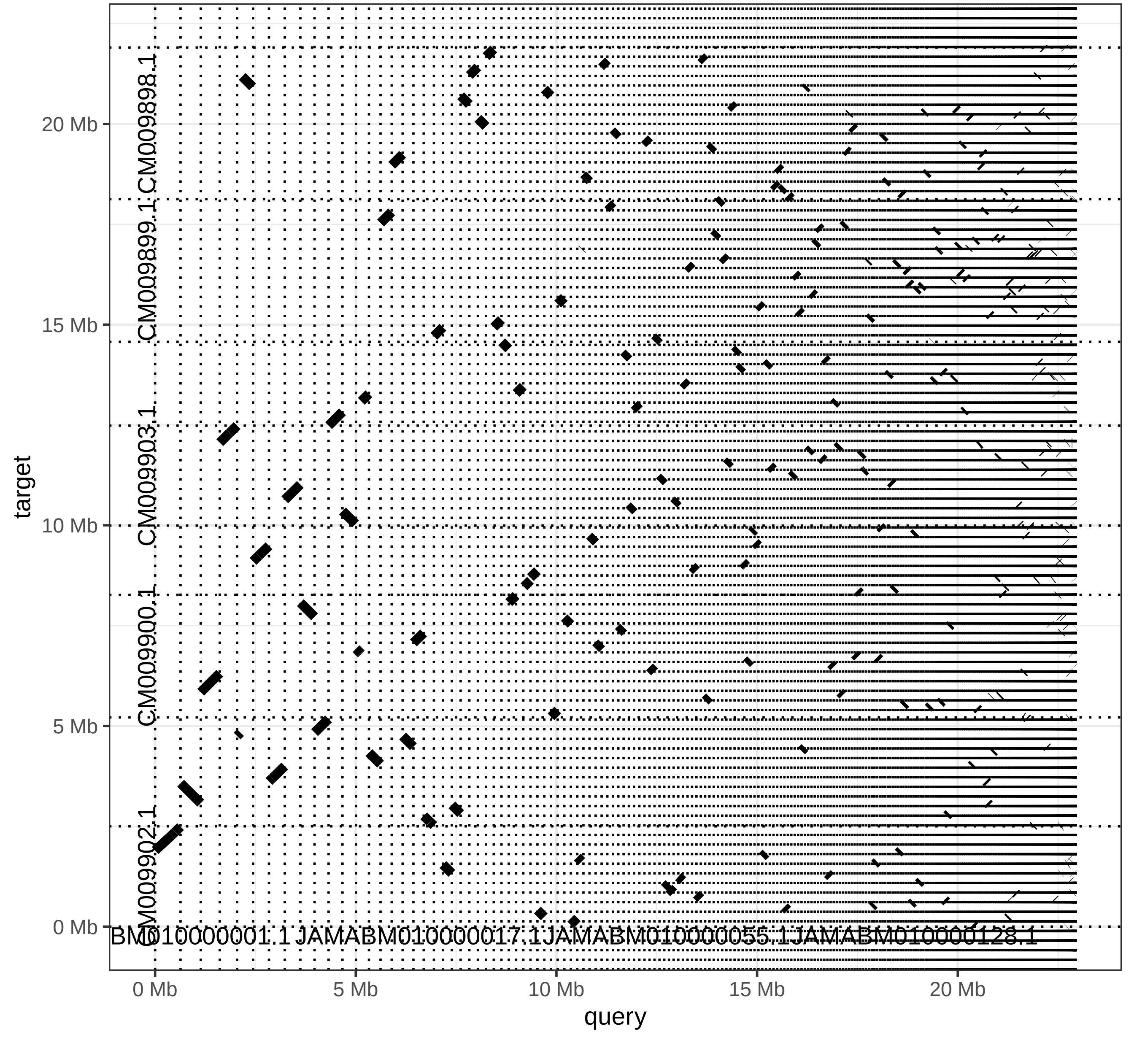

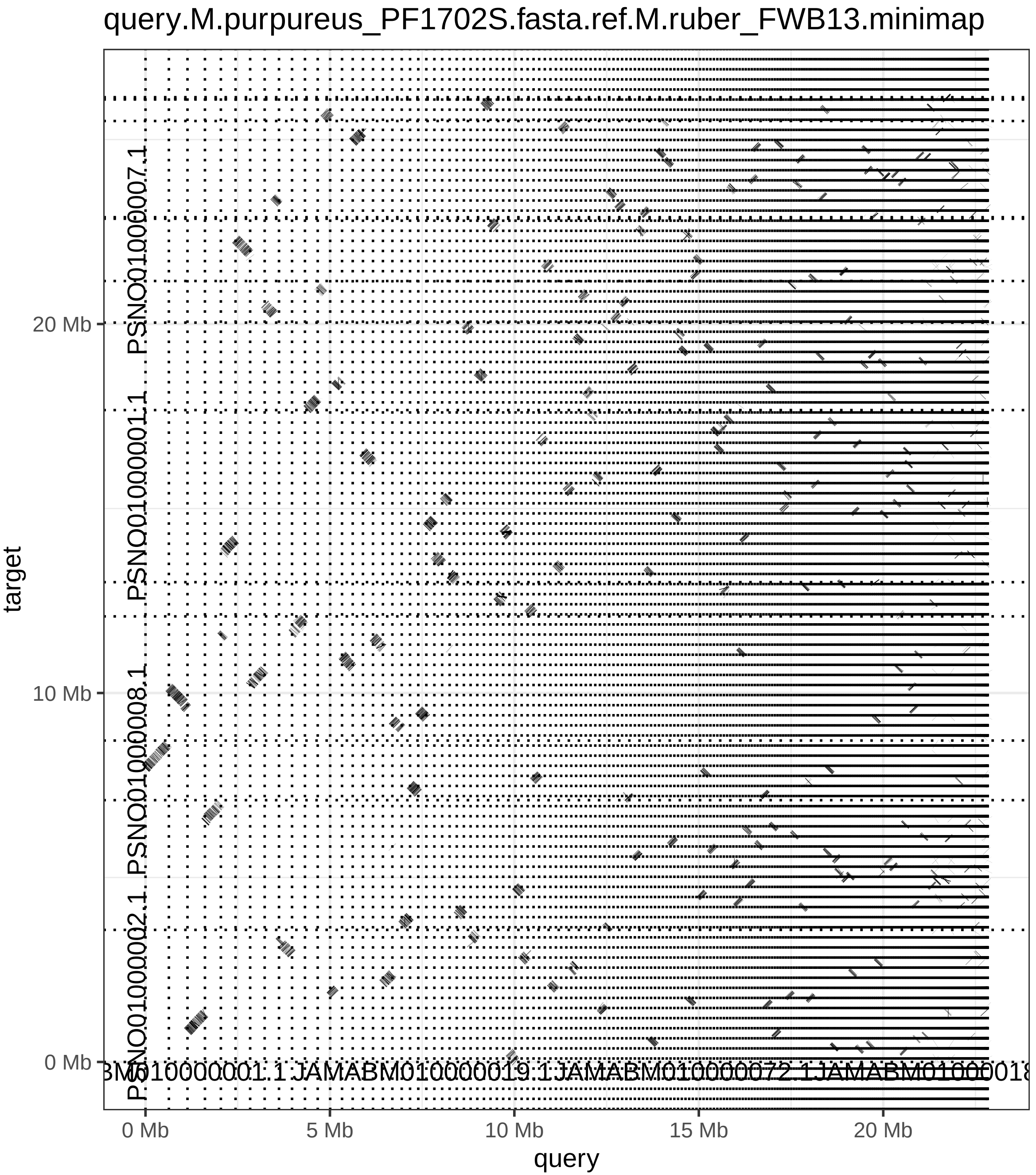
query.M.purpureus_PF1702S.fasta.ref.M.ruber_FWB13.minimap
target
query
0 Mb
5 Mb
10 Mb
15 Mb
20 Mb
0 Mb
10 Mb
20 Mb
PSNO01000007.1
PSNO01000001.1
PSNO01000008.1
PSNO01000002.1
JAMABM010000019.1
JAMABM010000072.1

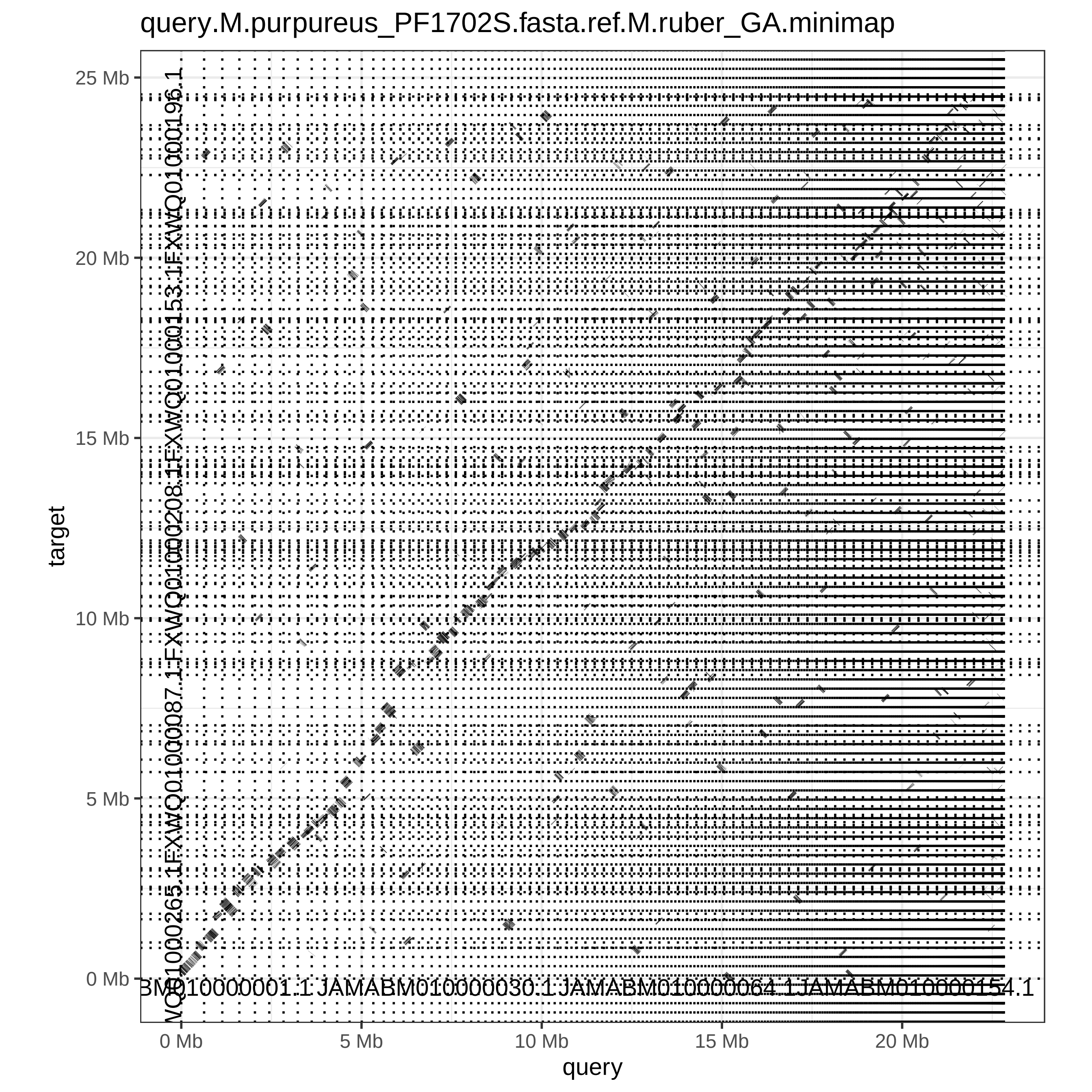
query.M.purpureus_PF1702S.fasta.ref.M.ruber_GA.minimap
target
25 Mb
20 Mb
15 Mb
10 Mb
5 Mb
0 Mb
query
0 Mb
5 Mb
10 Mb
15 Mb
20 Mb
JAMABM010000030.1
JAMABM010000064.1
JAMABM010000154.1

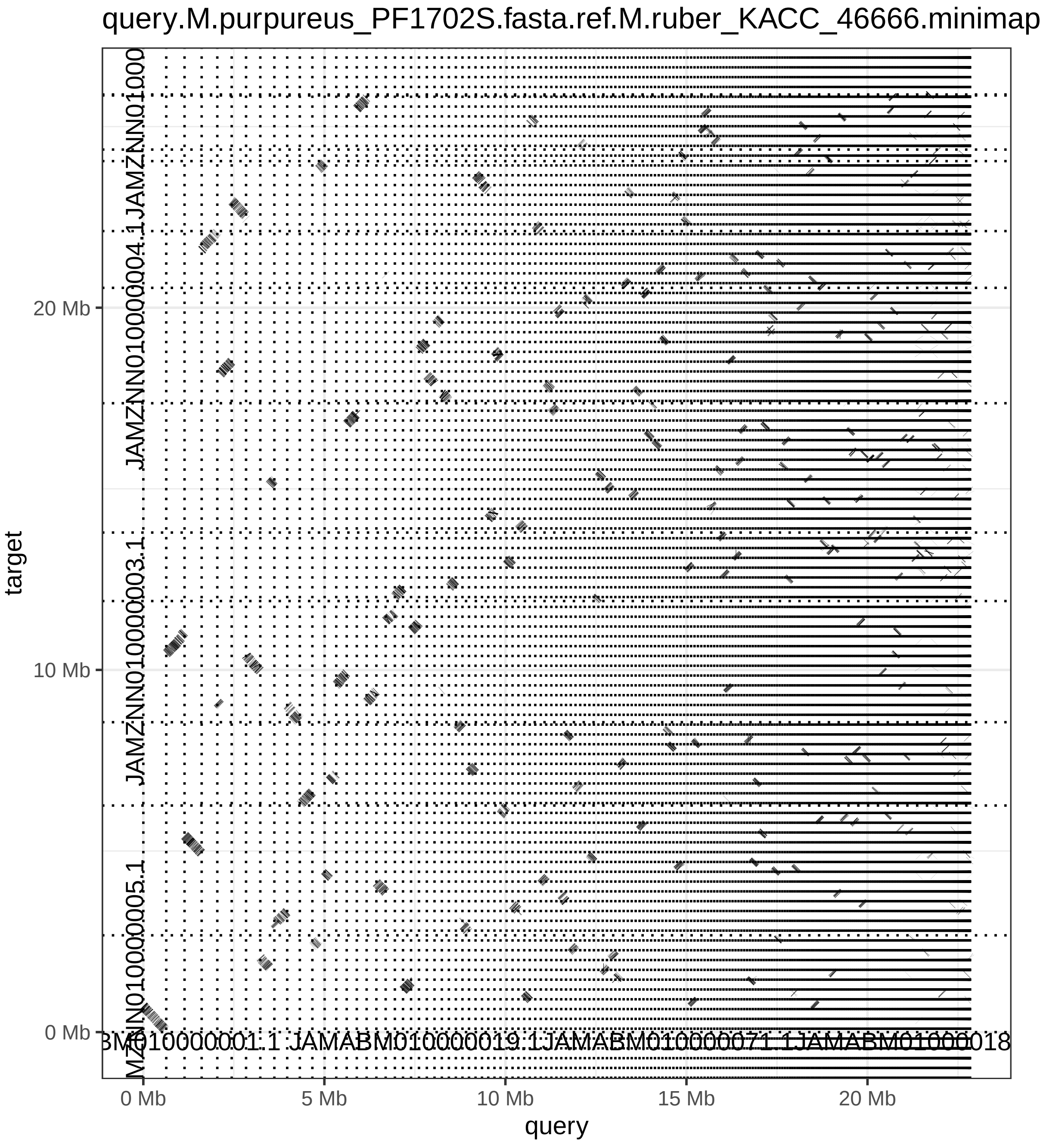
query.M.purpureus_PF1702S.fasta.ref.M.ruber_KACC_46666.minimap
target
query
0 Mb
10 Mb
20 Mb
0 Mb
5 Mb
10 Mb
15 Mb
20 Mb

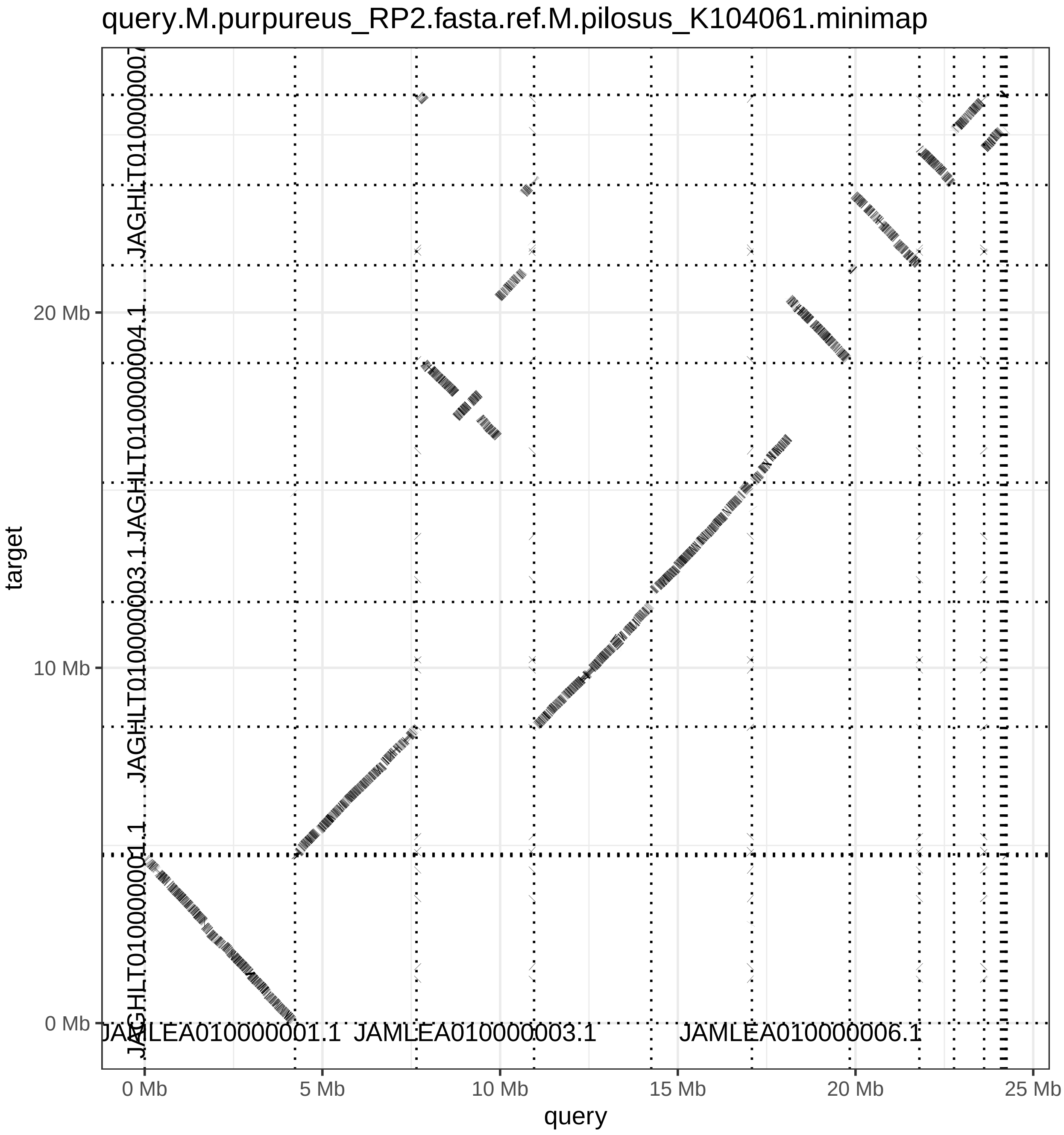
query.M.purpureus_RP2.fasta.ref.M.pilosus_K104061.minimap
target
query
0 Mb
5 Mb
10 Mb
15 Mb
20 Mb
25 Mb
0 Mb
10 Mb
20 Mb
JAMLEA010000001.1
JAMLEA010000003.1
JAMLEA010000006.1
JAGHLT010000001.1
JAGHLT010000003.1
JAGHLT010000004.1
JAGHLT01000000

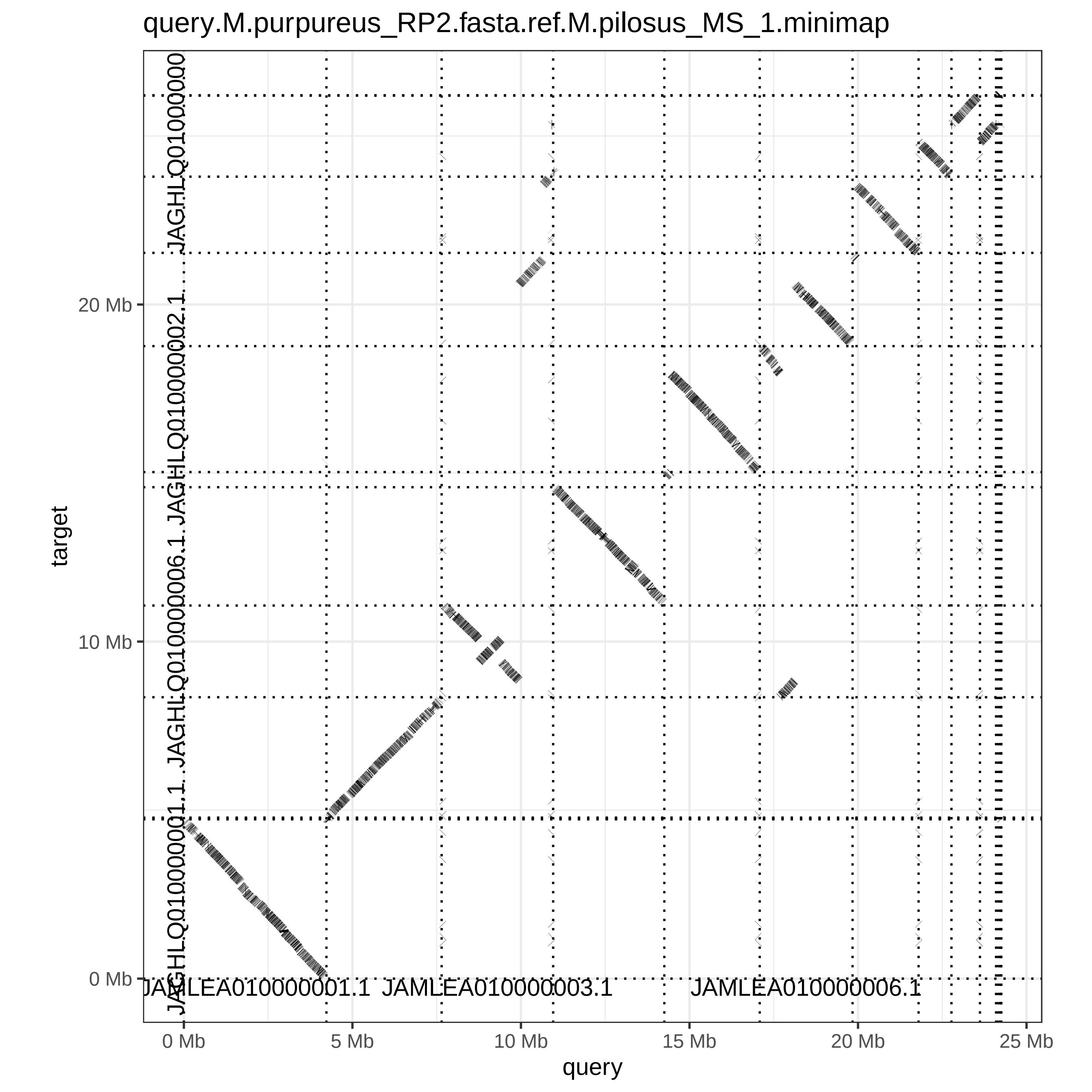
query.M.purpureus_RP2.fasta.ref.M.pilosus_MS_1.minimap
target
query
0 Mb
10 Mb
20 Mb
0 Mb
5 Mb
10 Mb
15 Mb
20 Mb
25 Mb
JAMLEA010000001.1
JAMLEA010000003.1
JAMLEA010000006.1
JAGHLQ010000001.1
JAGHLQ010000006.1
JAGHLQ010000002.1
JAGHLQ01000000

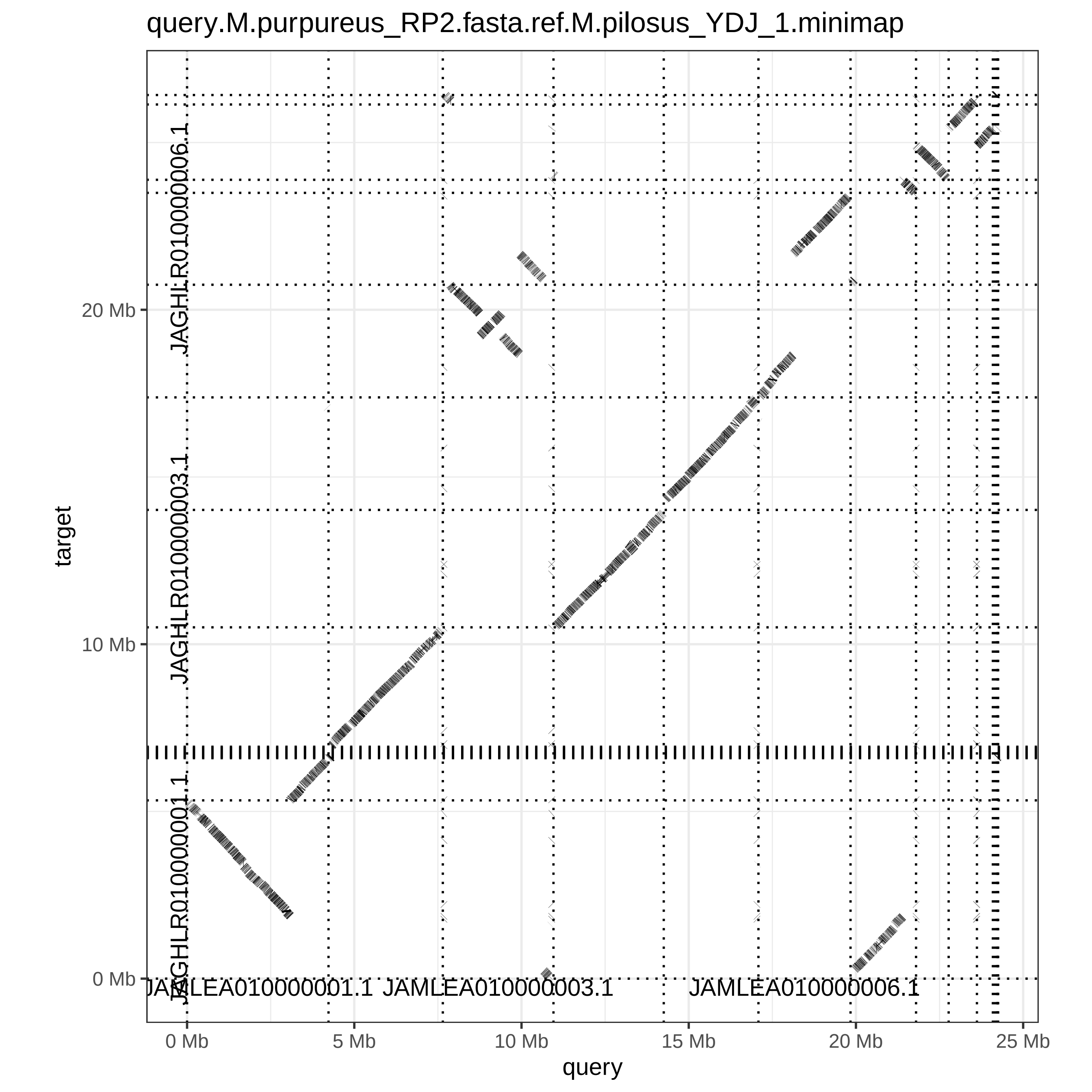
query.M.purpureus_RP2.fasta.ref.M.pilosus_YDJ_1.minimap
target
query
JAGHLR010000006.1
JAGHLR010000003.1
JAGHLR010000001.1
JAMLEA010000001.1
JAMLEA010000003.1
JAMLEA010000006.1
0 Mb
10 Mb
20 Mb
0 Mb
5 Mb
10 Mb
15 Mb
20 Mb
25 Mb

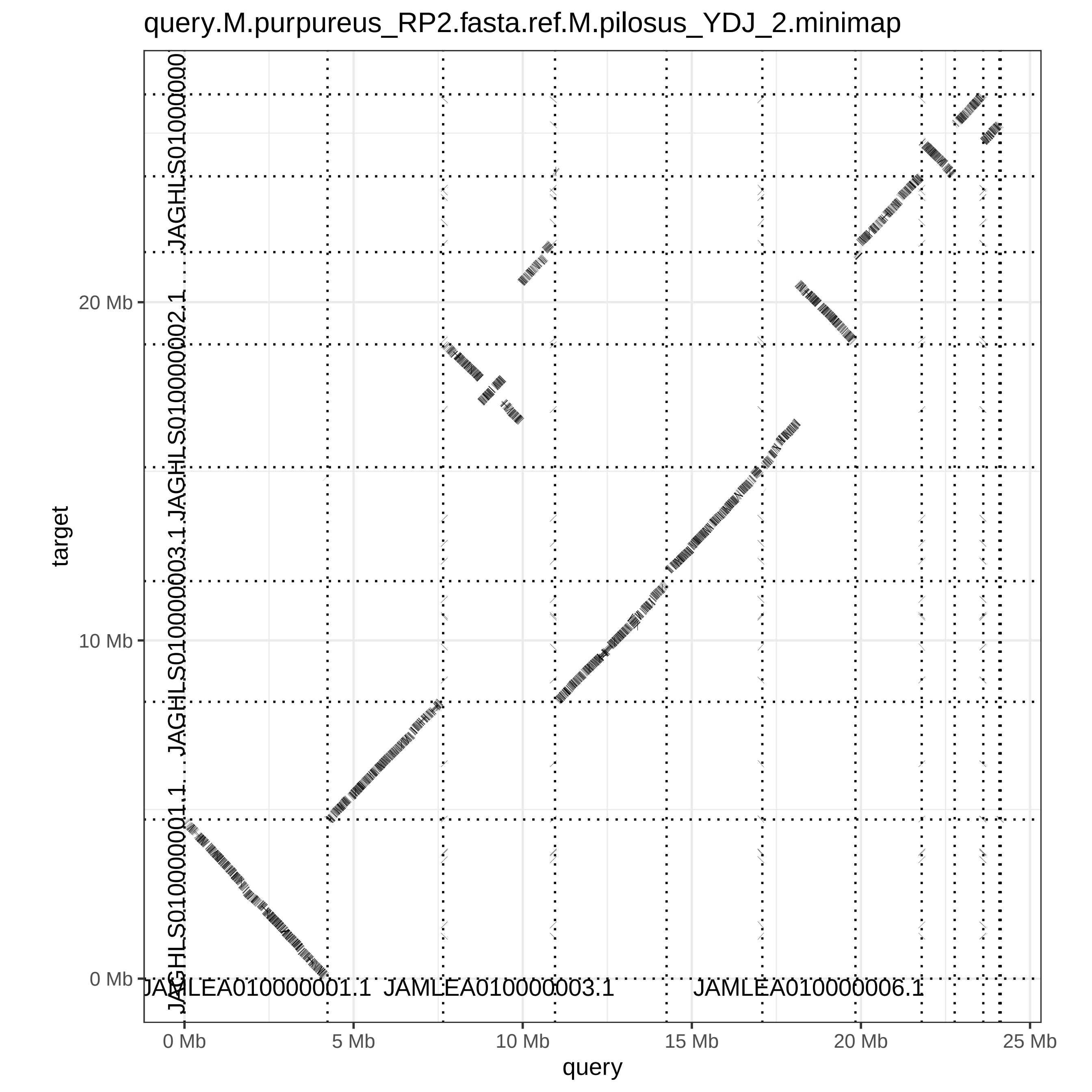
query.M.purpureus_RP2.fasta.ref.M.pilosus_YDJ_2.minimap
target
query
0 Mb
10 Mb
20 Mb
0 Mb
5 Mb
10 Mb
15 Mb
20 Mb
25 Mb
JAMLEA010000001.1
JAMLEA010000003.1
JAMLEA010000006.1
JAGHLS010000001.1
JAGHLS010000002.1
JAGHLS010000003.1
JAGHLS010000000

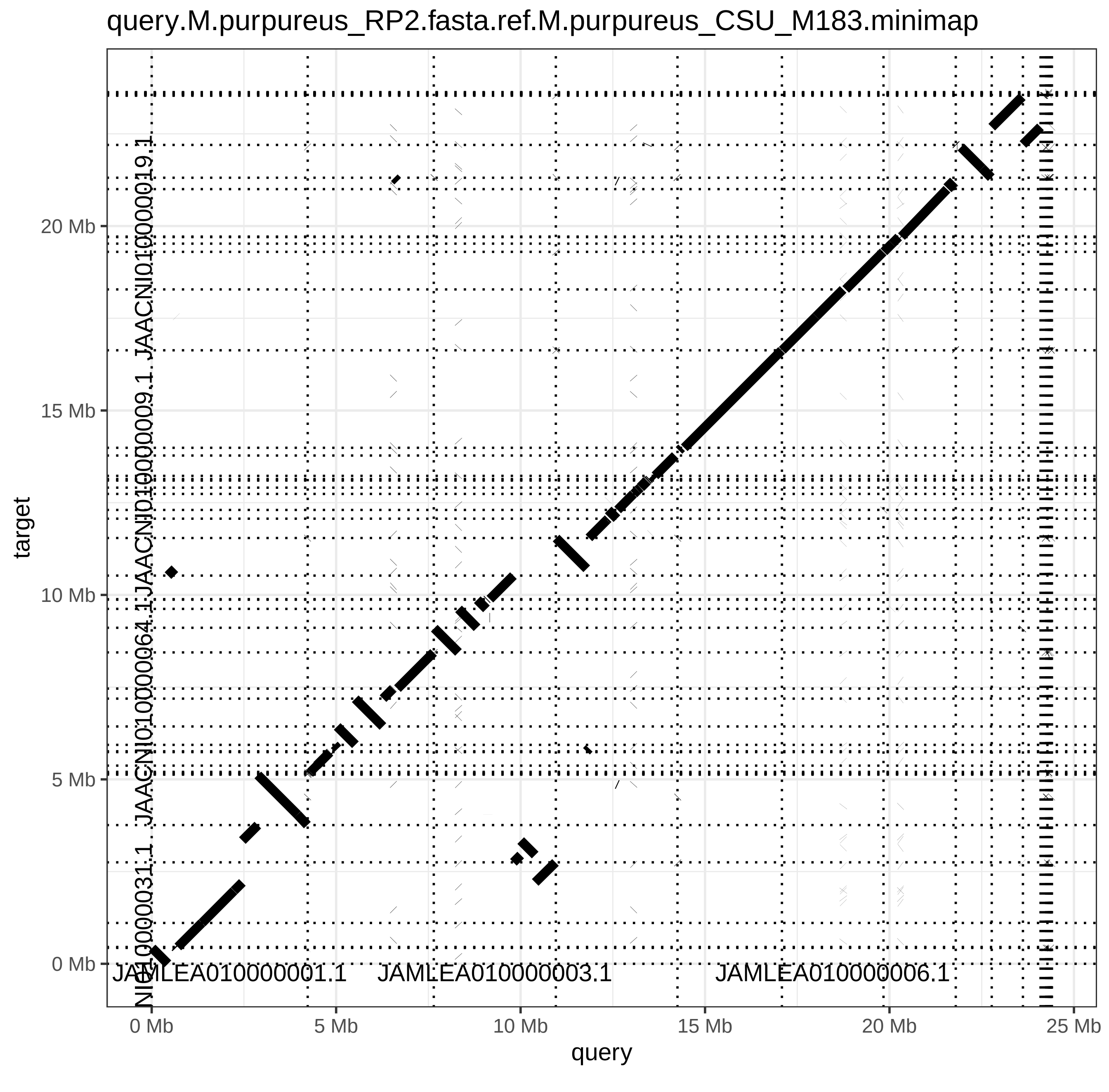
query.M.purpureus_RP2.fasta.ref.M.purpureus_CSU_M183.minimap
target
query
0 Mb
5 Mb
10 Mb
15 Mb
20 Mb
25 Mb
JAMLEA010000001.1
JAMLEA010000003.1
JAMLEA010000006.1
JAACNI010000031.1
JAACNI010000064.1
JAACNI010000009.1
JAACNI010000019.1

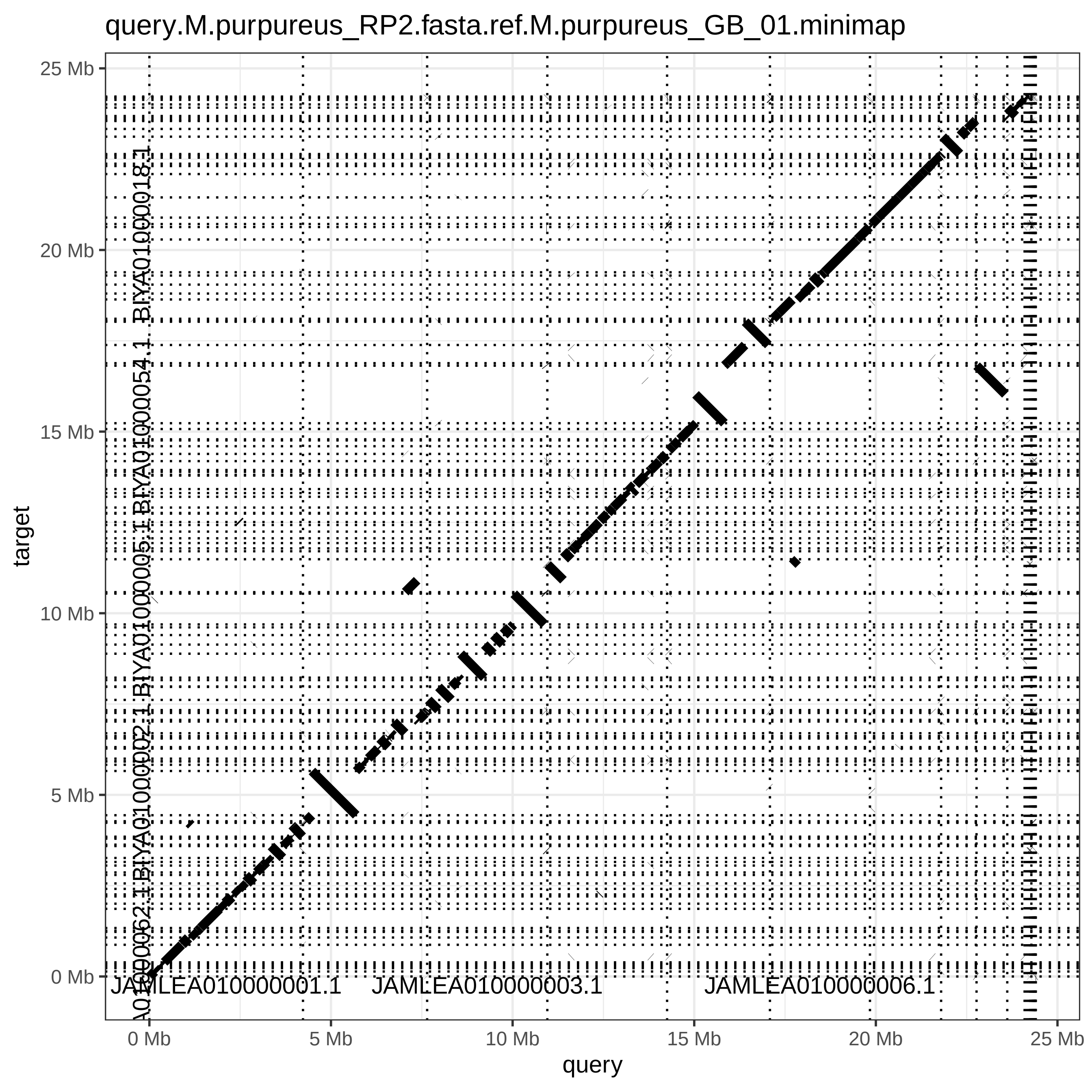

query.M.purpureus_RP2.fasta.ref.M.purpureus_GB_01.minimap
target
query
25 Mb
20 Mb
15 Mb
10 Mb
5 Mb
0 Mb
0 Mb
5 Mb
10 Mb
15 Mb
20 Mb
25 Mb
JAMLEA010000001.1
JAMLEA010000003.1
JAMLEA010000006.1
BIYA010000018.1
BIYA010000054.1
BIYA010000005.1
BIYA010000002.1
BIYA010000062.1

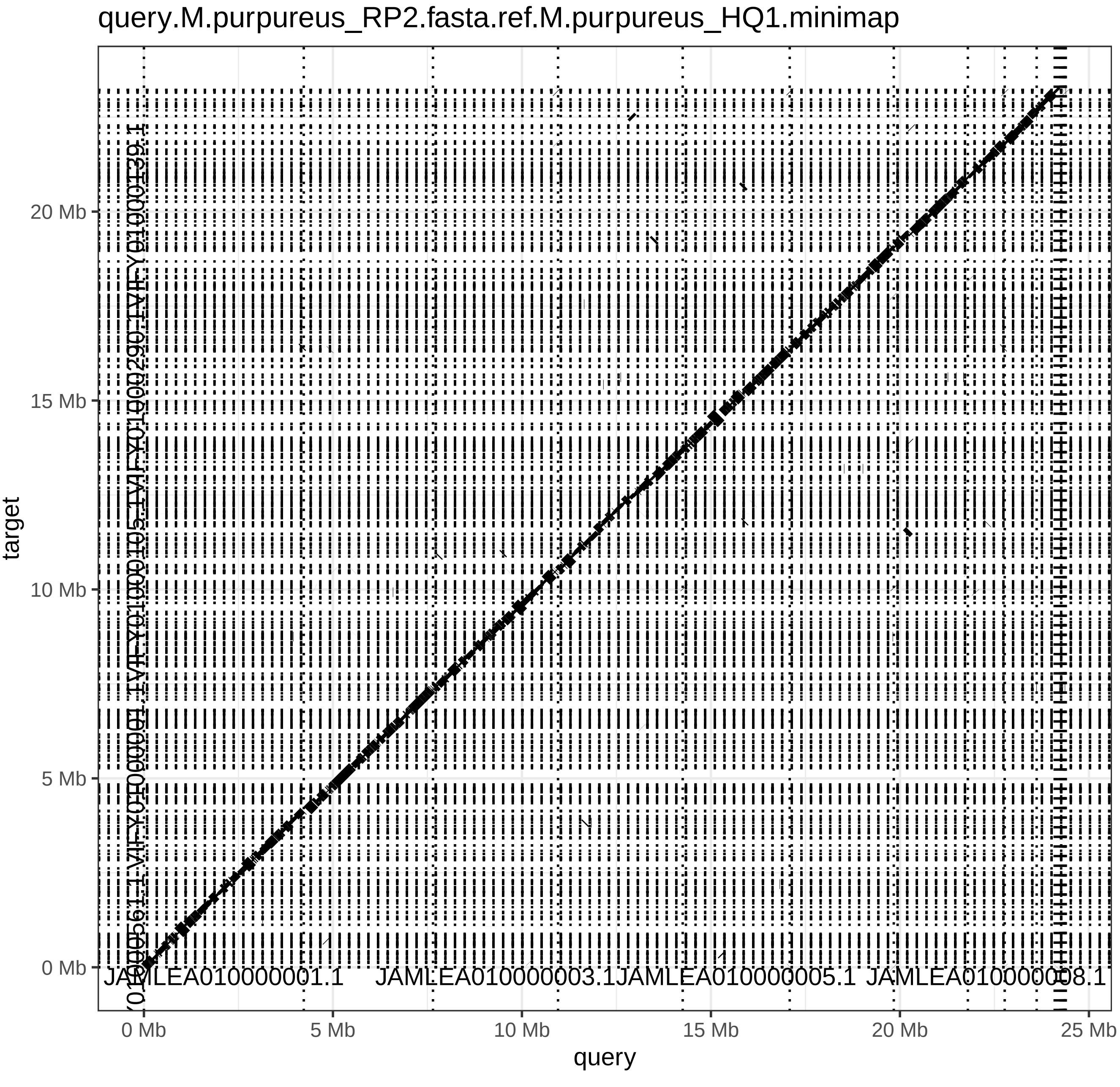
query.M.purpureus_RP2.fasta.ref.M.purpureus_HQ1.minimap
target
20 Mb
15 Mb
10 Mb
5 Mb
0 Mb
JAMLEA010000001.1
JAMLEA010000003.1
JAMLEA010000005.1
JAMLEA010000008.1
0 Mb
5 Mb
10 Mb
15 Mb
20 Mb
25 Mb
query

query.M.purpureus_RP2.fasta.ref.M.purpureus_P7048X_2.minimap

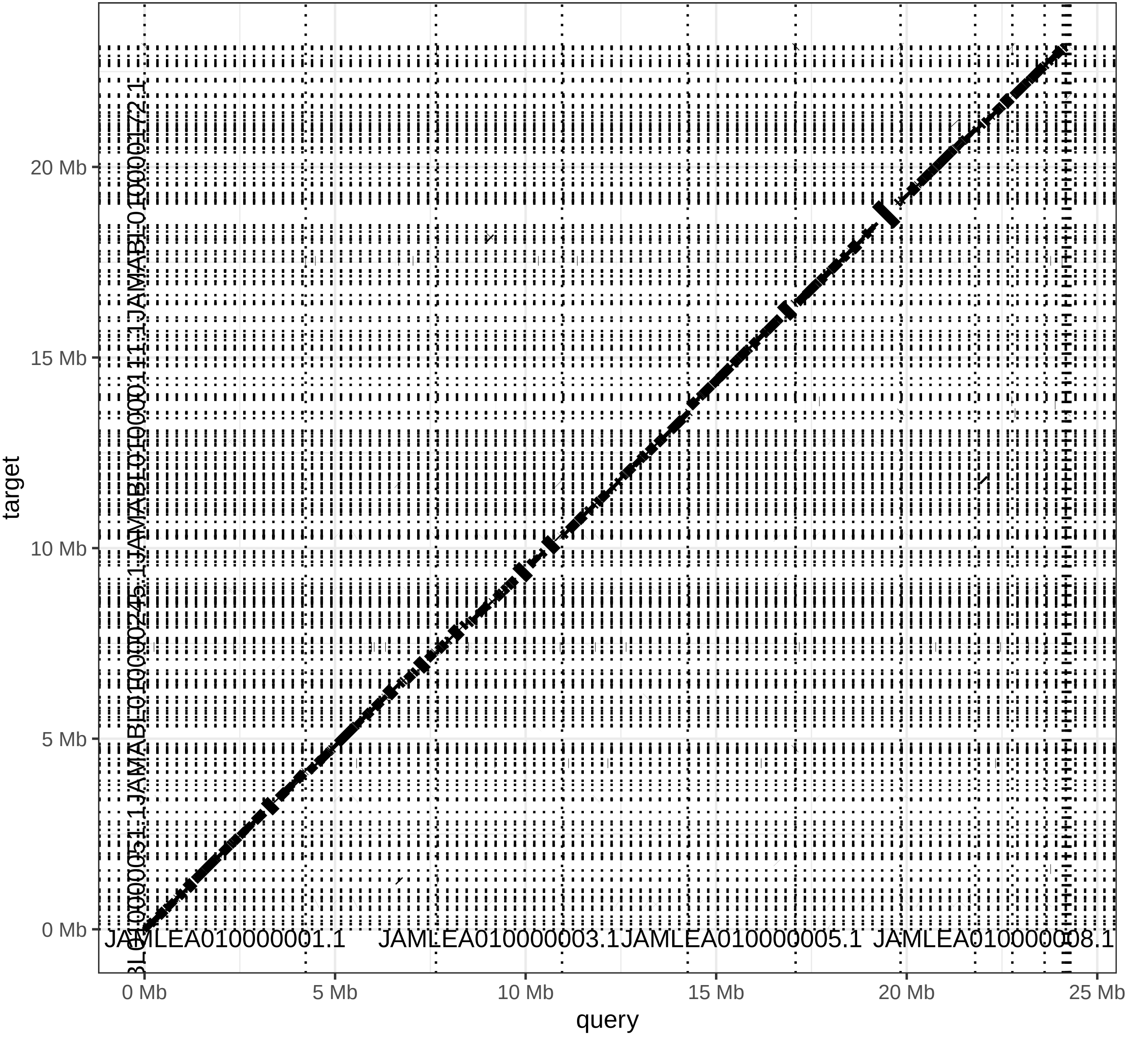

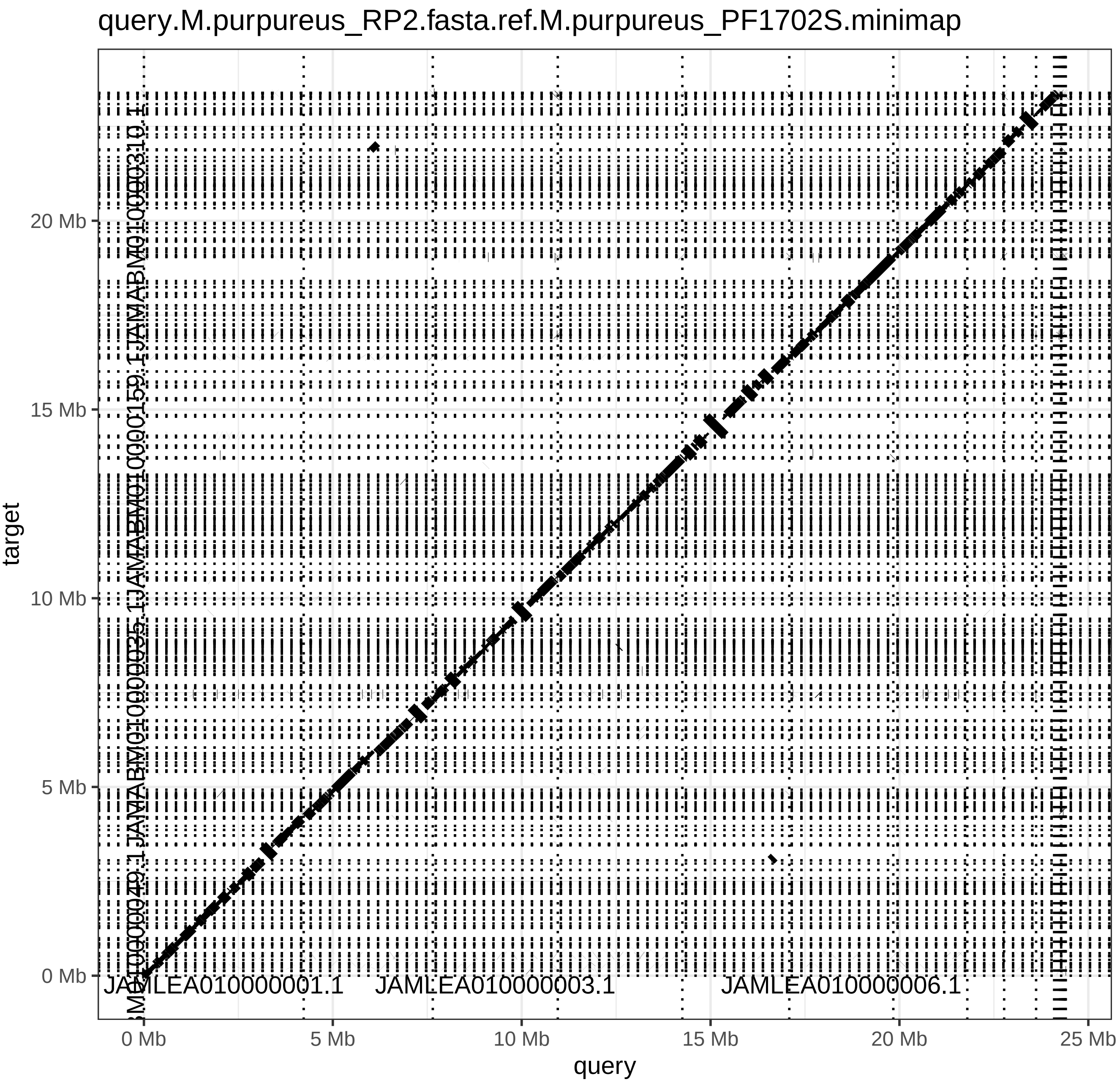
query.M.purpureus_RP2.fasta.ref.M.purpureus_PF1702S.minimap
target
0 Mb
5 Mb
10 Mb
15 Mb
20 Mb
query
0 Mb
5 Mb
10 Mb
15 Mb
20 Mb
25 Mb
JAMLEA010000001.1
JAMLEA010000003.1
JAMLEA010000006.1

query.M.purpureus_RP2.fasta.ref.M.purpureus_YJX_8.minimap

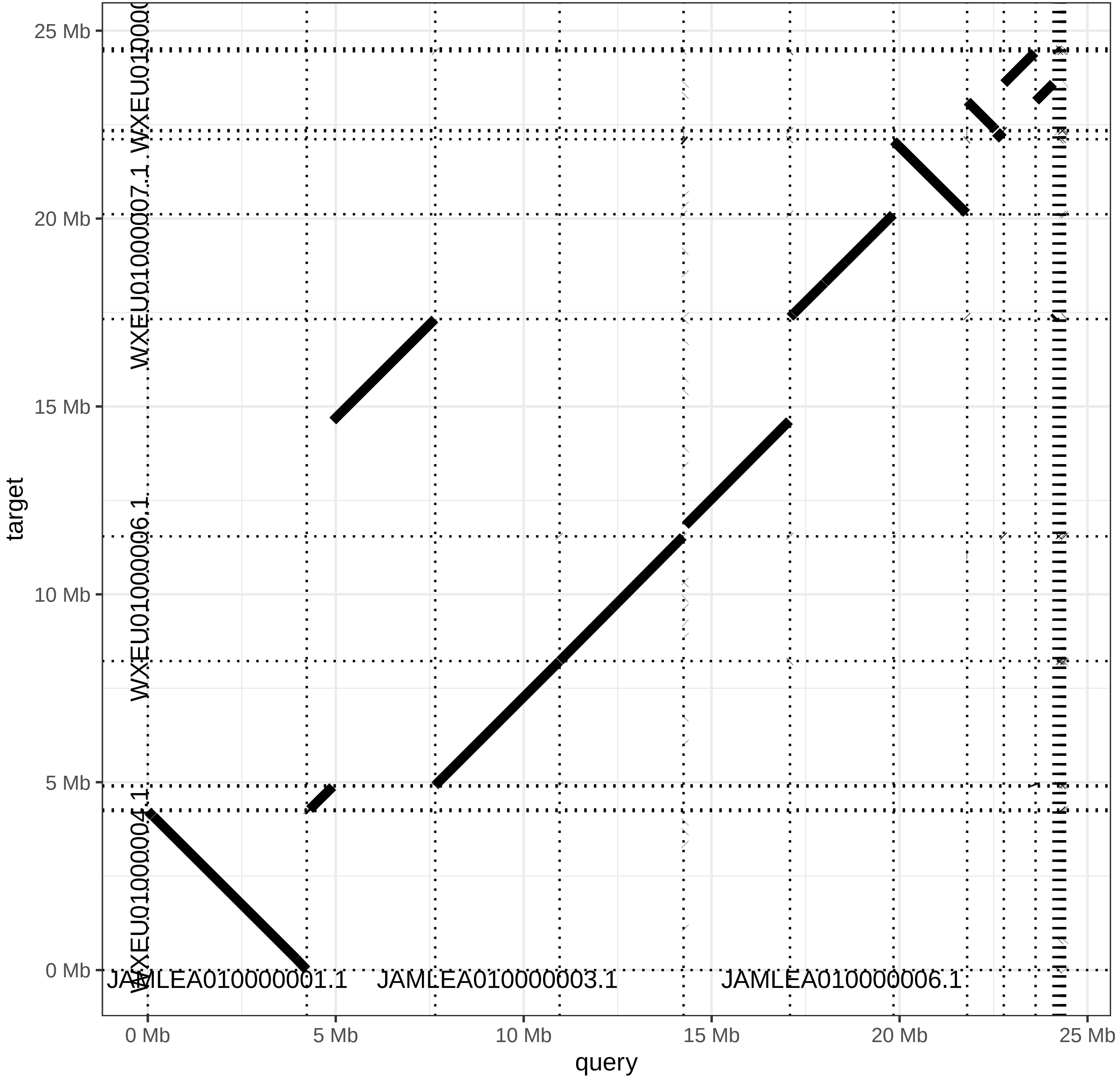

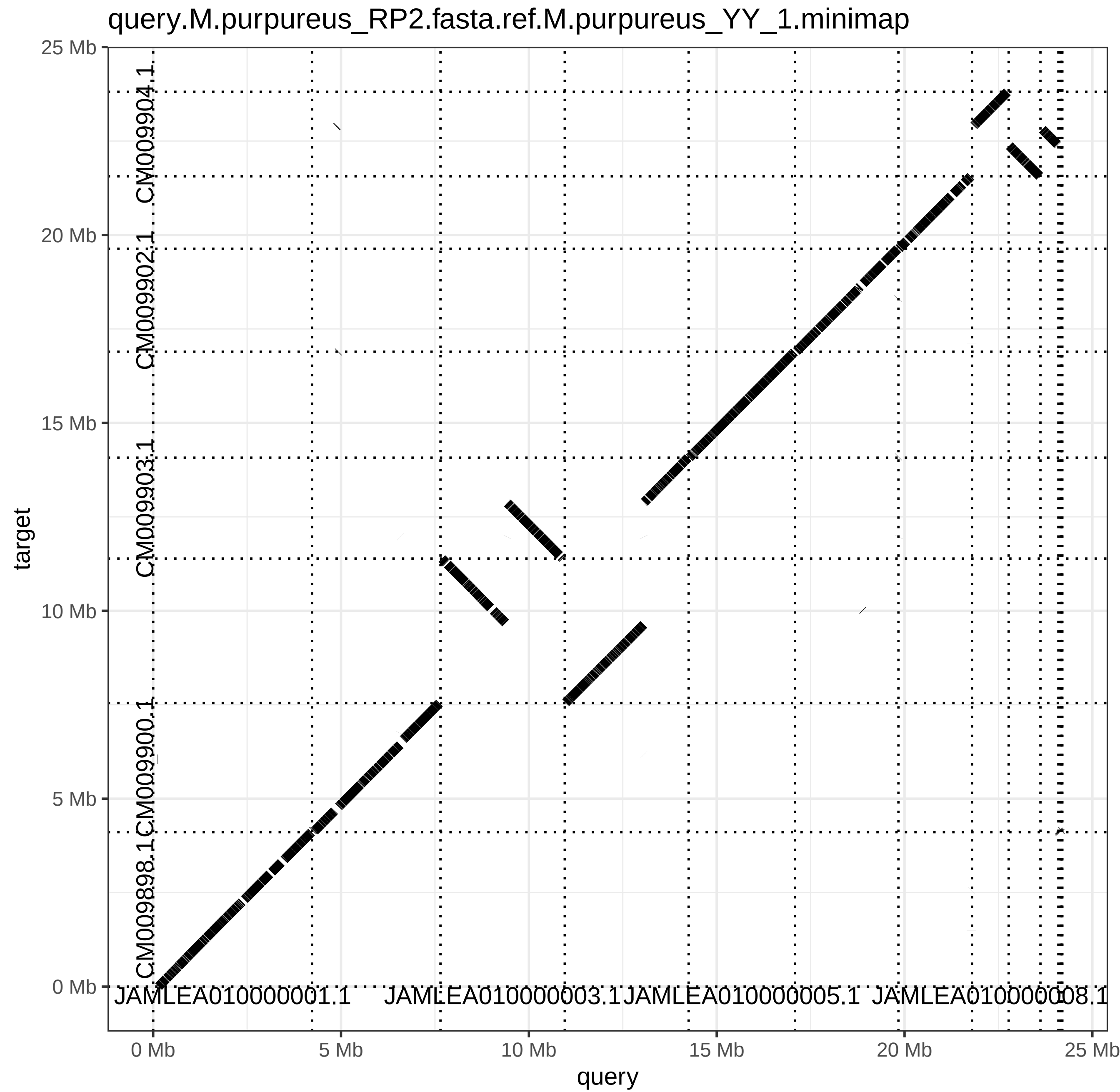

query.M.purpureus_RP2.fasta.ref.M.purpureus_YY_1.minimap
target
query
25 Mb
20 Mb
15 Mb
10 Mb
5 Mb
0 Mb
0 Mb
5 Mb
10 Mb
15 Mb
20 Mb
25 Mb
CM009904.1
CM009902.1
CM009903.1
CM009900.1
CM009898.1
JAMLEA010000001.1
JAMLEA010000003.1
JAMLEA010000005.1
JAMLEA010000008.1

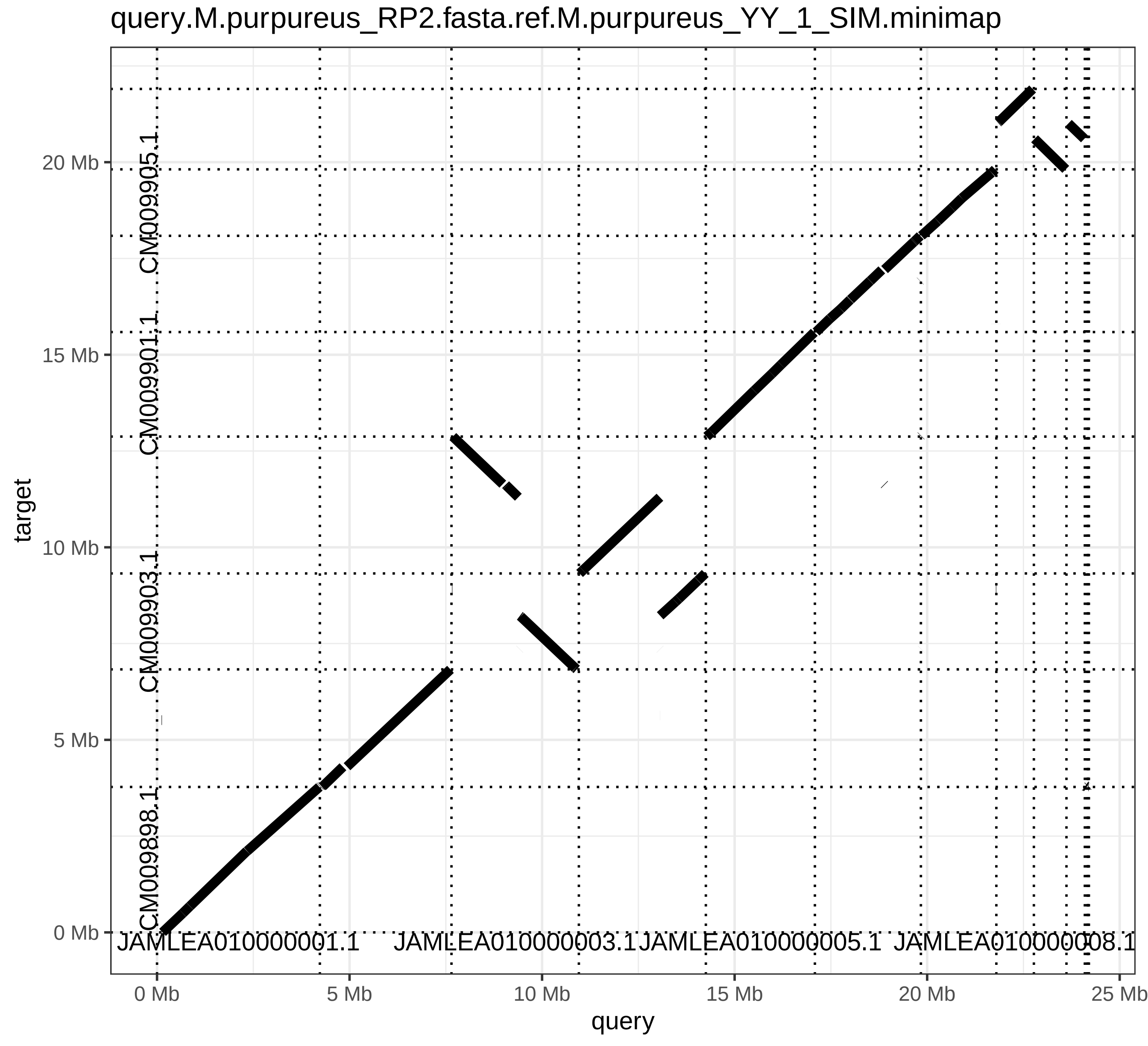
query.M.purpureus_RP2.fasta.ref.M.purpureus_YY_1_SIM.minimap
target
20 Mb
15 Mb
10 Mb
5 Mb
0 Mb
CM009905.1
CM009901.1
CM009903.1
CM009898.1
JAMLEA010000001.1
JAMLEA010000003.1
JAMLEA010000005.1
JAMLEA010000008.1
0 Mb
5 Mb
10 Mb
15 Mb
20 Mb
25 Mb
query

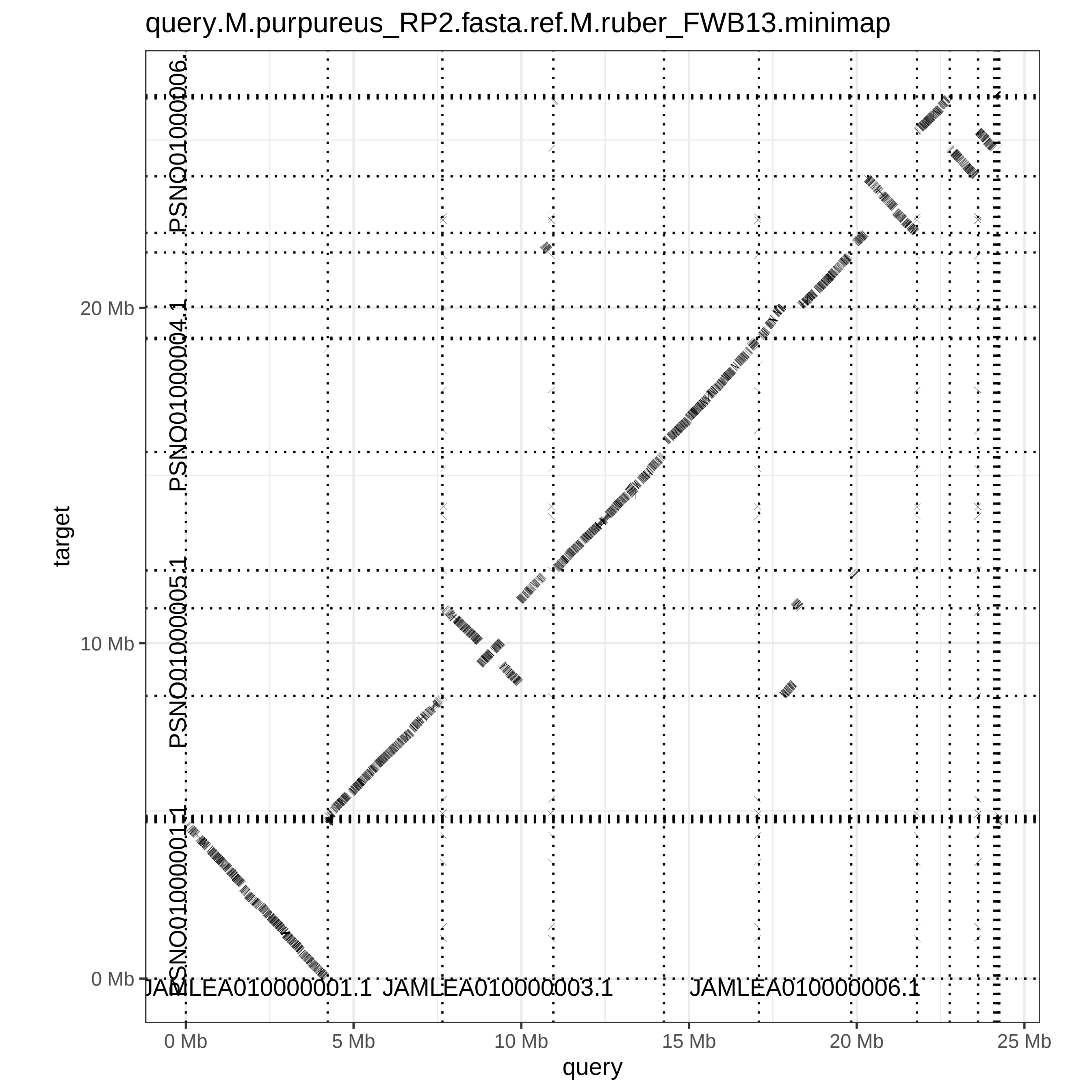
query.M.purpureus_RP2.fasta.ref.M.ruber_FWB13.minimap
target
query
0 Mb
10 Mb
20 Mb
5 Mb
15 Mb
25 Mb
PSNO01000006.1
PSNO01000004.1
PSNO01000005.1
PSNO01000001.1
JAMLEA010000001.1
JAMLEA010000003.1
JAMLEA010000006.1

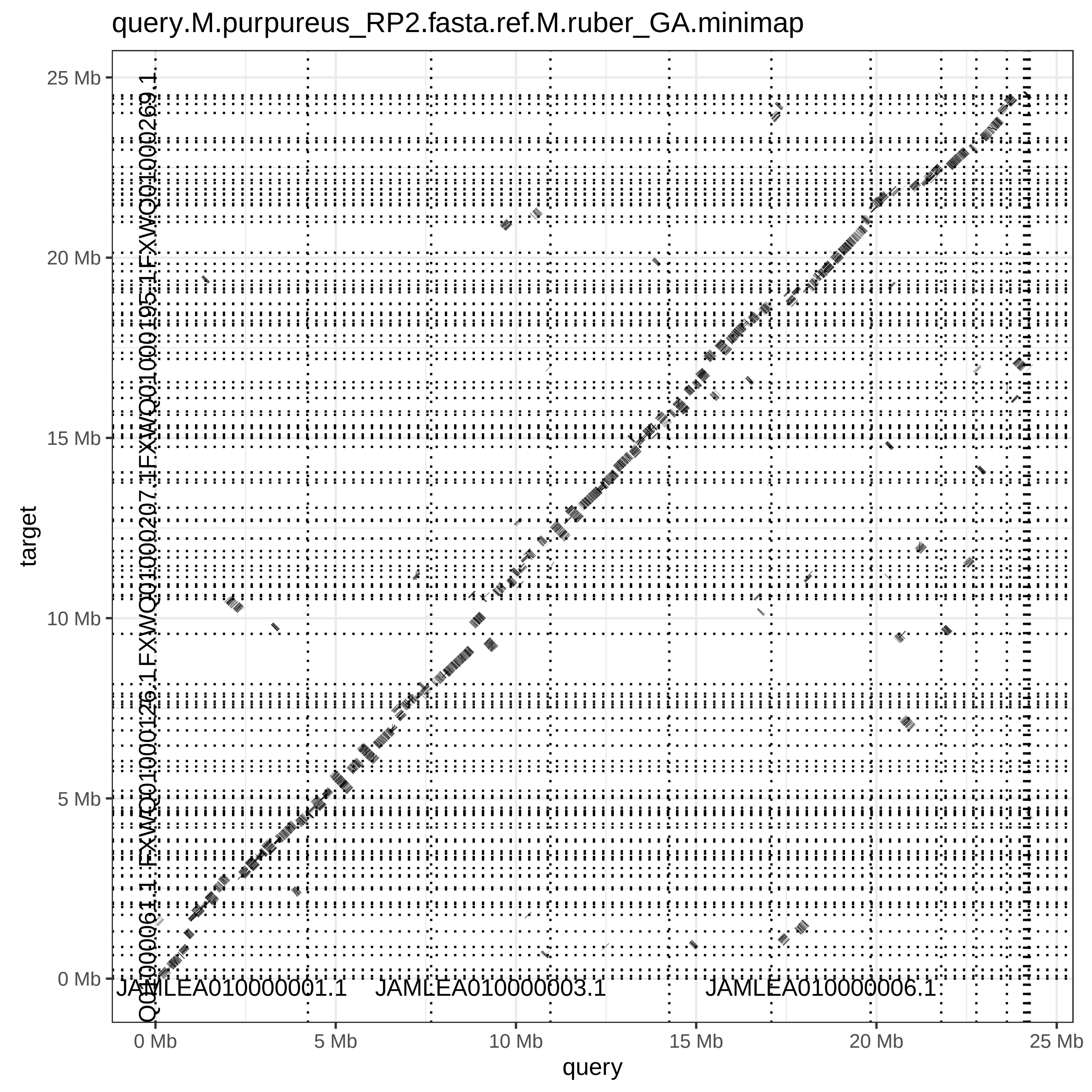
query.M.purpureus_RP2.fasta.ref.M.ruber_GA.minimap
target
query
25 Mb
20 Mb
15 Mb
10 Mb
5 Mb
0 Mb
0 Mb
5 Mb
10 Mb
15 Mb
20 Mb
25 Mb
JAMLEA010000001.1
JAMLEA010000003.1
JAMLEA010000006.1

query.M.purpureus_RP2.fasta.ref.M.ruber_KACC_46666.minimap

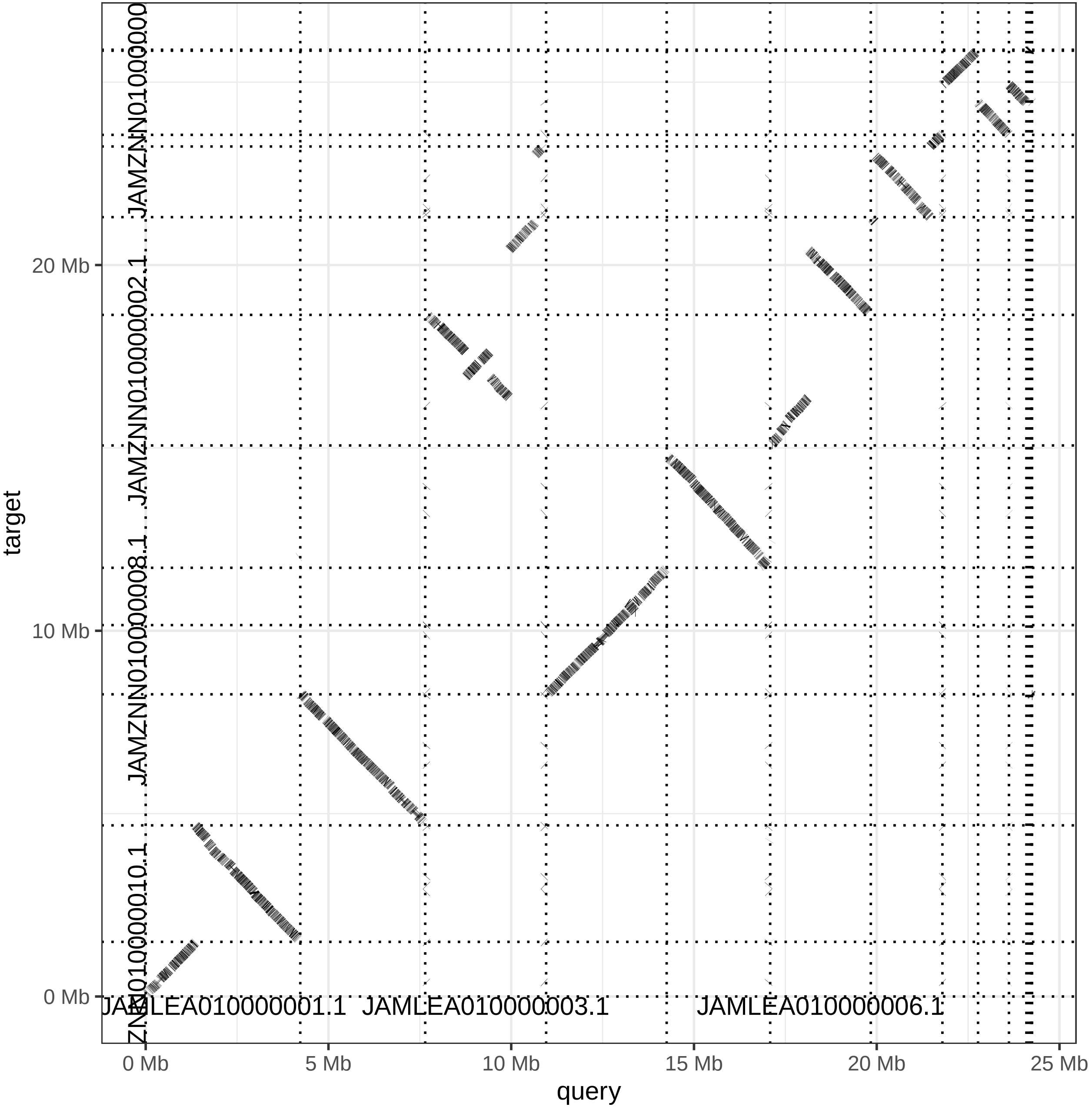

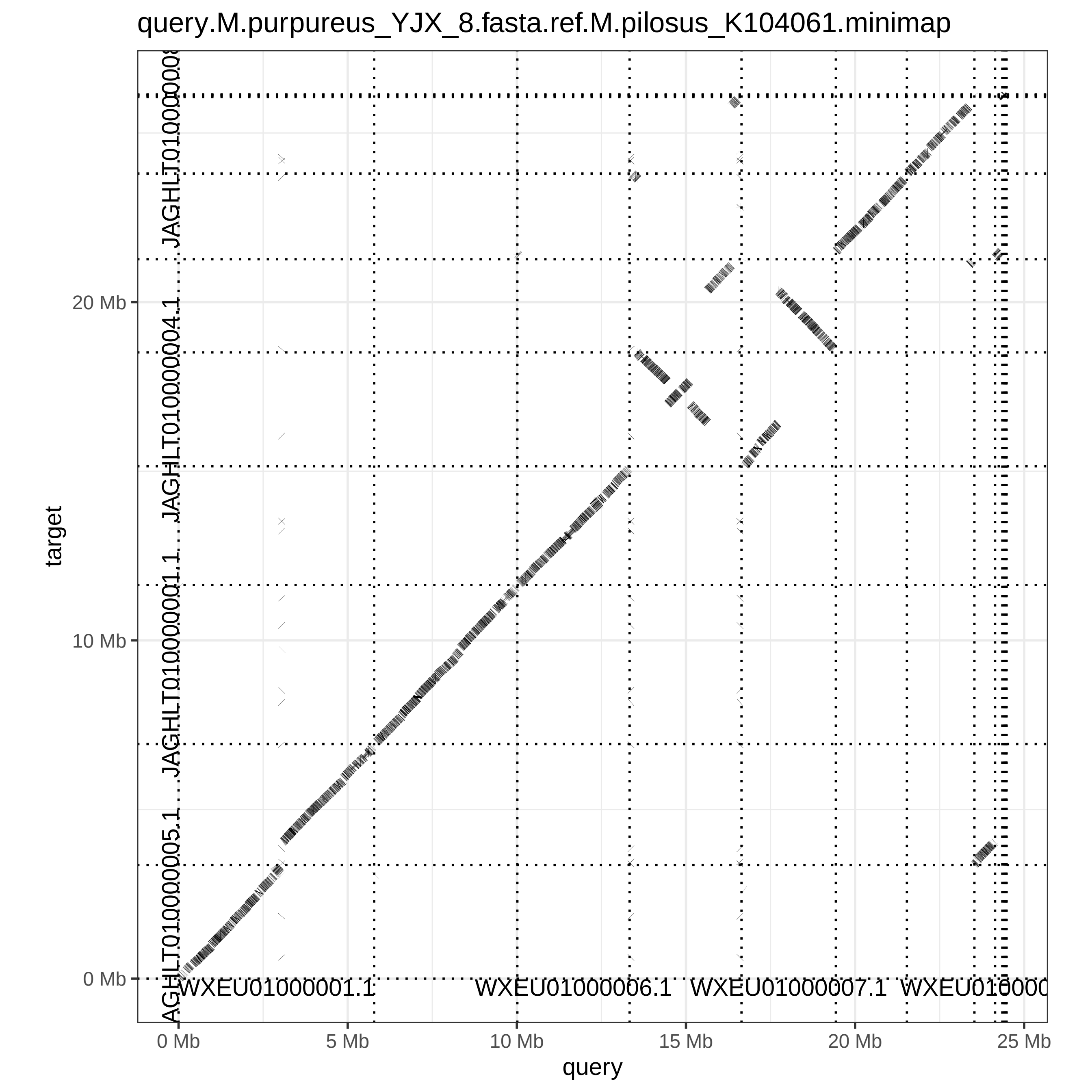

query.M.purpureus_YJX_8.fasta.ref.M.pilosus_K104061.minimap
target
query
0 Mb
10 Mb
20 Mb
5 Mb
15 Mb
25 Mb
WXEU01000001.1
WXEU01000006.1
WXEU01000007.1
JAGHLT010000005.1
JAGHLT010000001.1
JAGHLT010000004.1

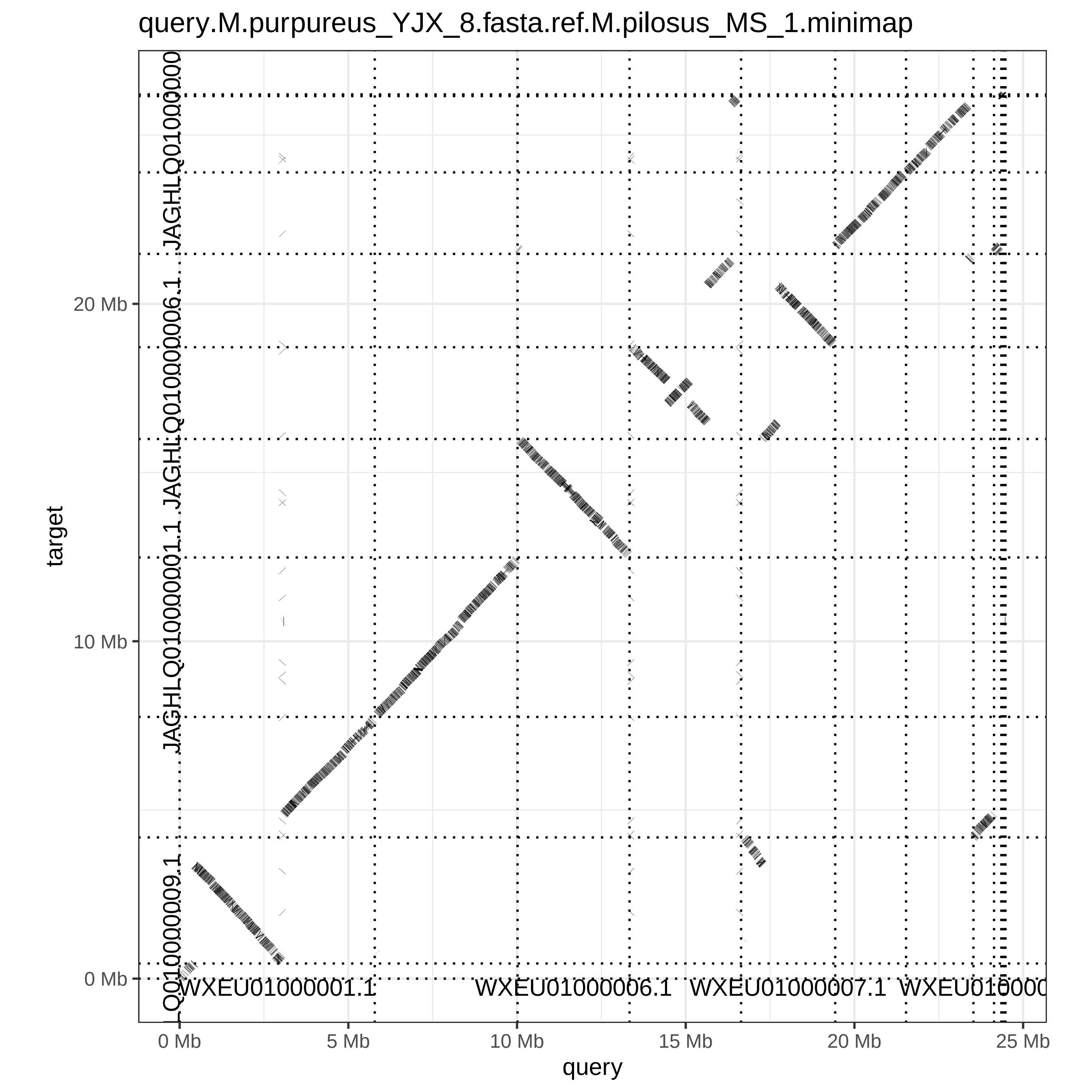

query.M.purpureus_YJX_8.fasta.ref.M.pilosus_MS_1.minimap
target
query
0 Mb
10 Mb
20 Mb
0 Mb
5 Mb
10 Mb
15 Mb
20 Mb
25 Mb
WXEU01000001.1
WXEU01000006.1
WXEU01000007.1
JAGHLQ010000006.1
JAGHLQ010000001.1

query.M.purpureus_YJX_8.fasta.ref.M.pilosus_YDJ_1.minimap

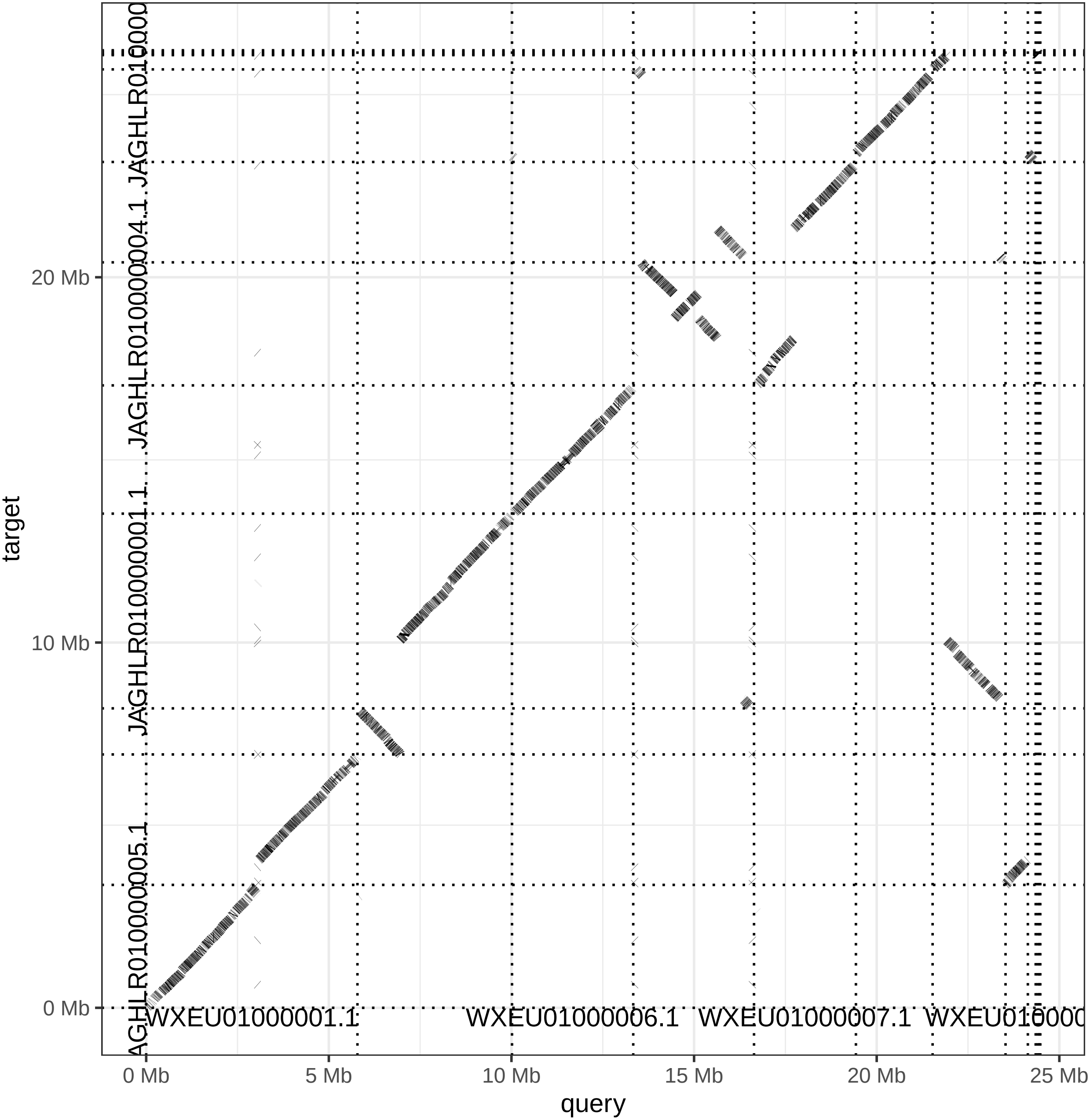

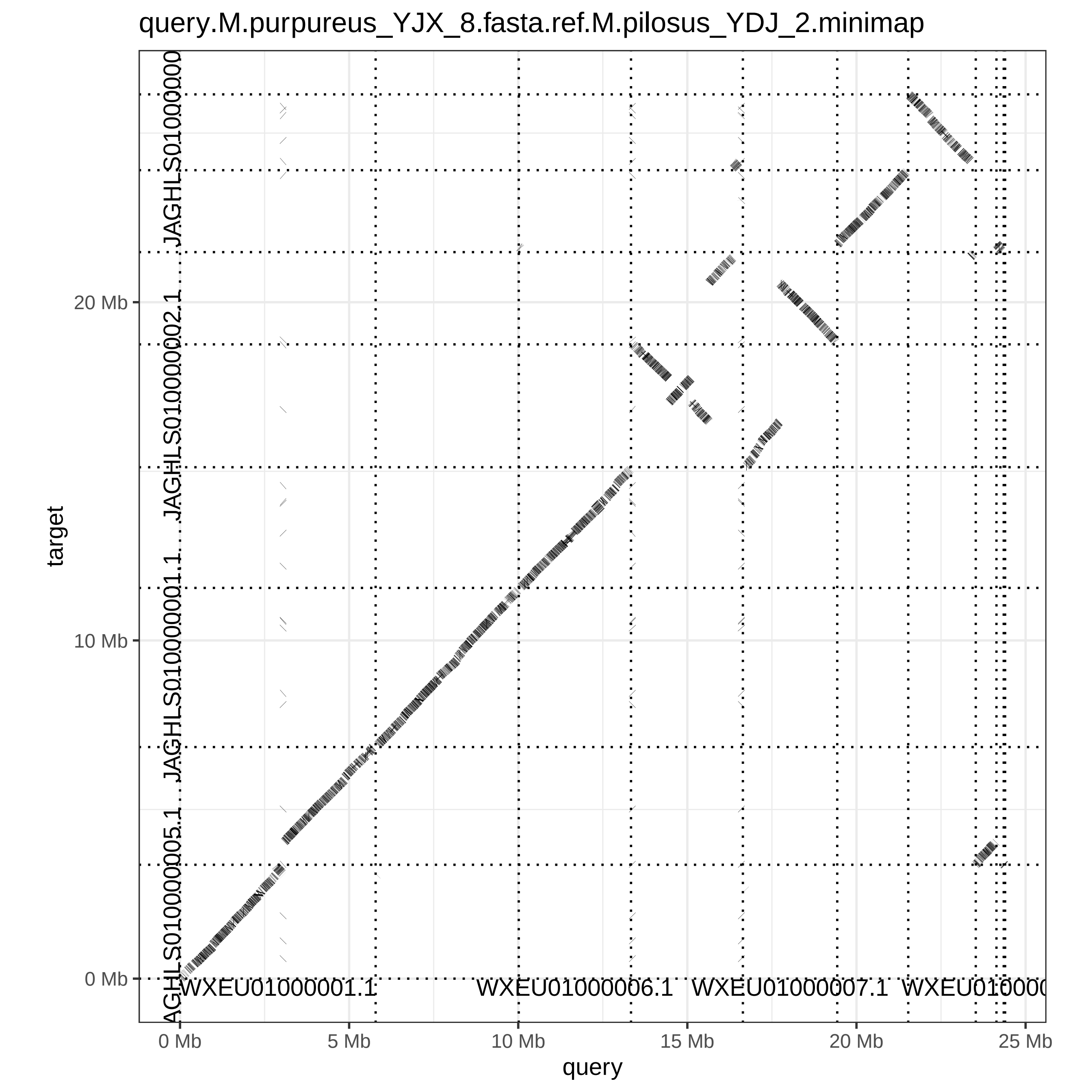
query.M.purpureus_YJX_8.fasta.ref.M.pilosus_YDJ_2.minimap
target
20 Mb
10 Mb
0 Mb
JAGHLS01000000
JAGHLS010000002.1
JAGHLS010000001.1
JAGHLS010000005.1
JAGHLS010000000
WXEU01000001.1
WXEU01000006.1
WXEU01000007.1
0 Mb
5 Mb
10 Mb
15 Mb
20 Mb
25 Mb
query

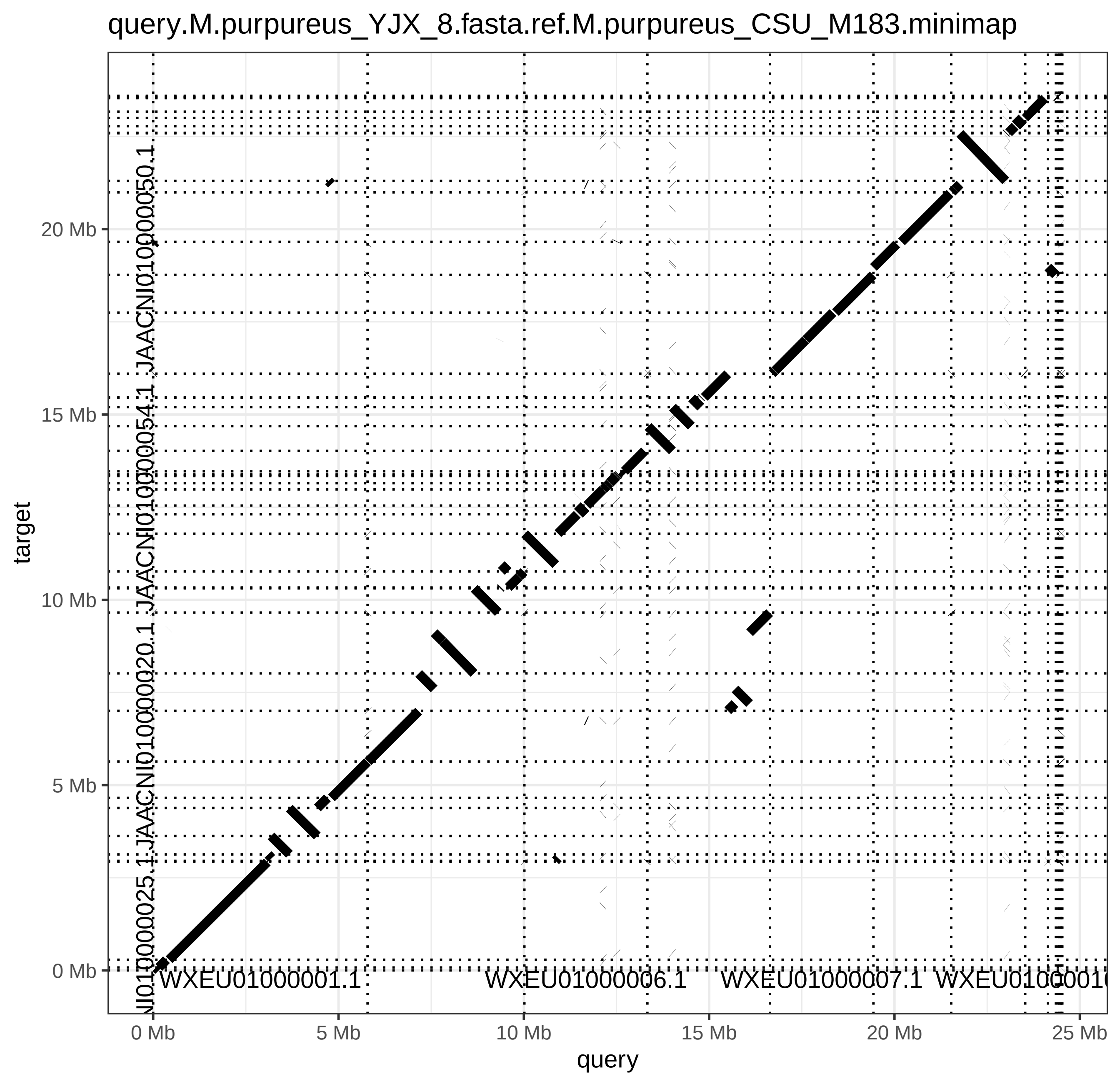

query.M.purpureus_YJX_8.fasta.ref.M.purpureus_CSU_M183.minimap
target
query
0 Mb
5 Mb
10 Mb
15 Mb
20 Mb
25 Mb
WXEU01000001.1
WXEU01000006.1
WXEU01000007.1
JAACNI010000050.1
JAACNI010000054.1
JAACNI010000020.1
JAACNI010000025.1

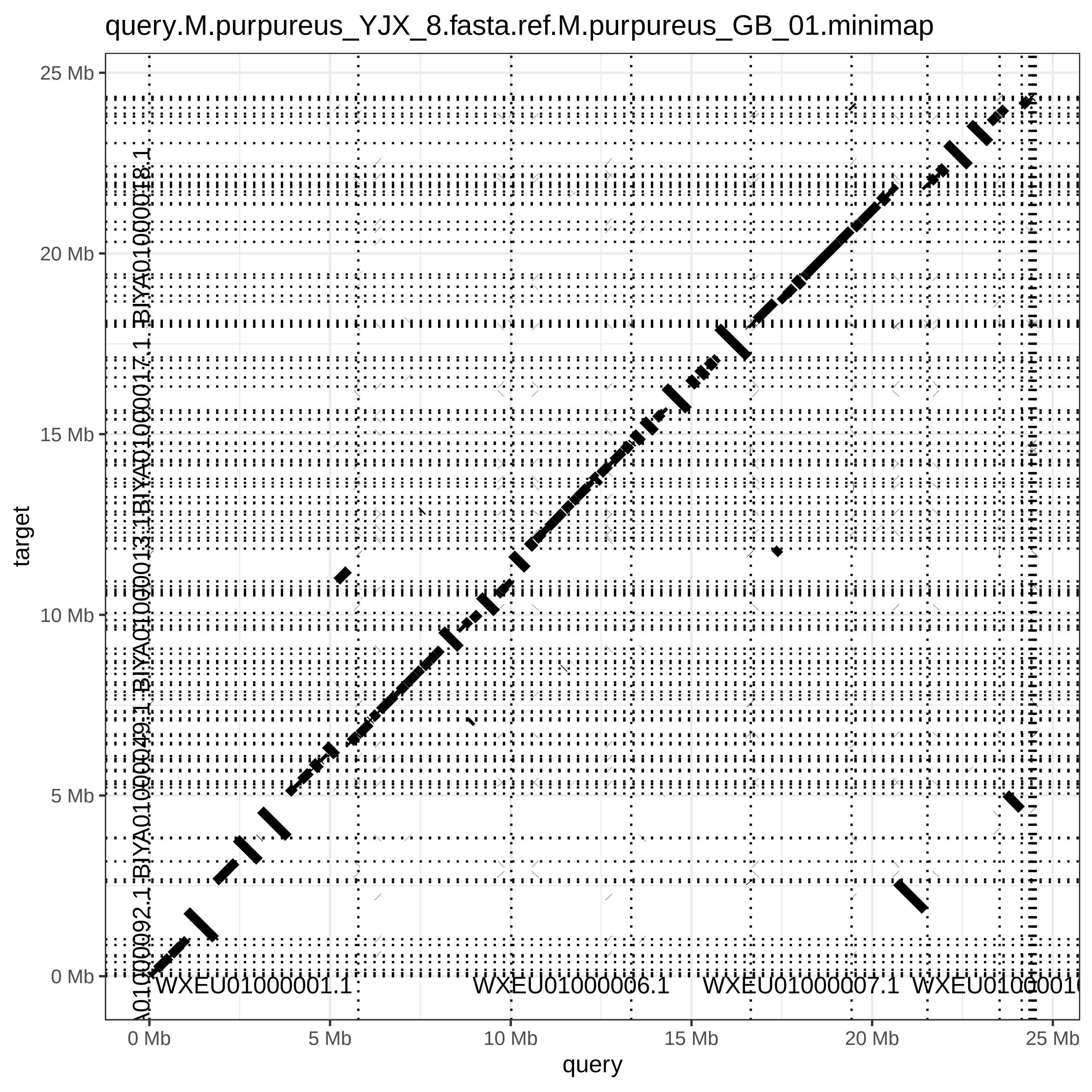
query.M.purpureus_YJX_8.fasta.ref.M.purpureus_GB_01.minimap
25 Mb
20 Mb
15 Mb
10 Mb
5 Mb
0 Mb
target
BIYA01000018.1
WXEU01000001.1
WXEU01000006.1
WXEU01000007.1
0 Mb
5 Mb
10 Mb
15 Mb
20 Mb
25 Mb
query

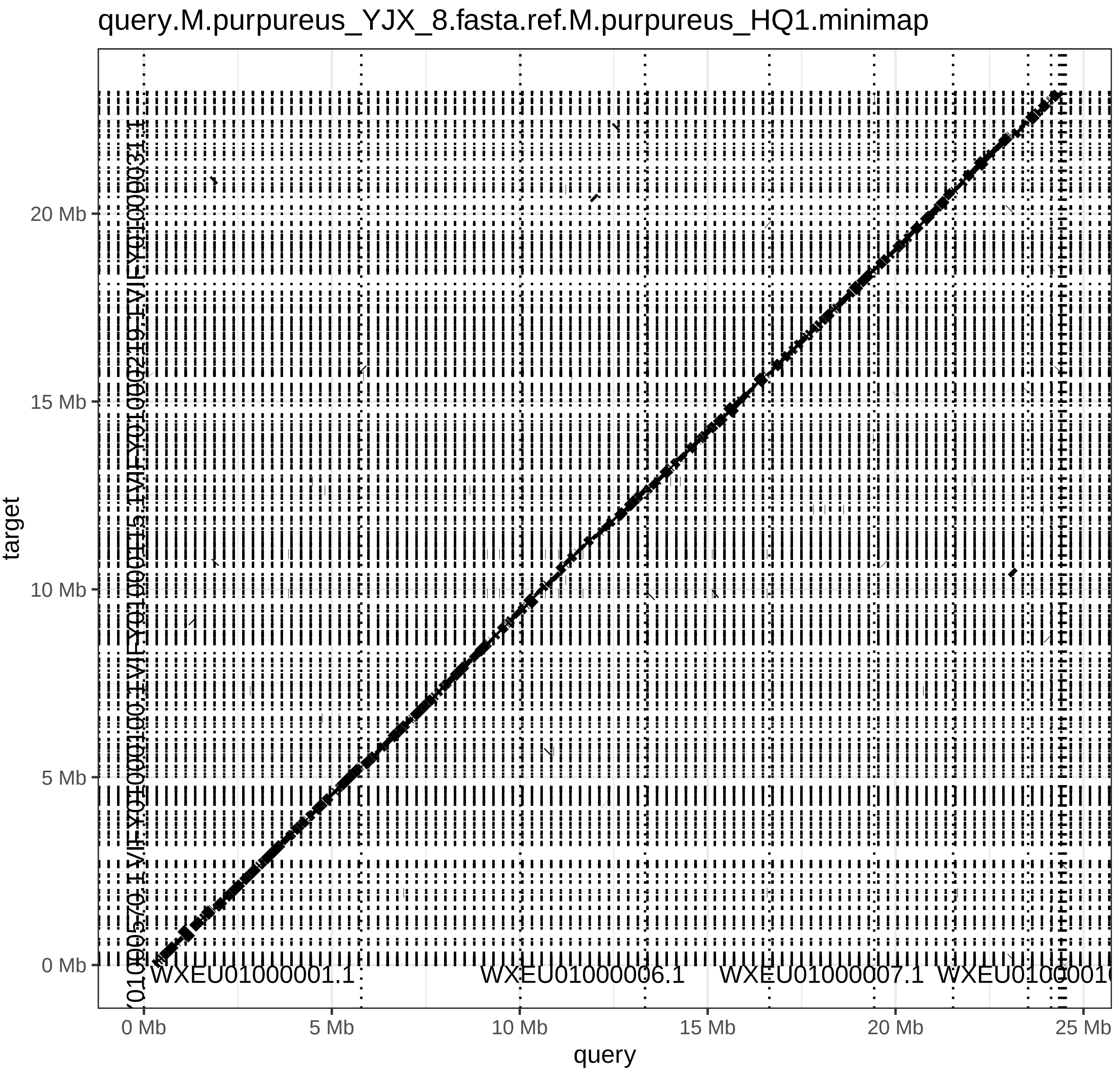

query.M.purpureus_YJX_8.fasta.ref.M.purpureus_HQ1.minimap
target
query
0 Mb
5 Mb
10 Mb
15 Mb
20 Mb
25 Mb
WXEU01000001.1
WXEU01000006.1
WXEU01000007.1

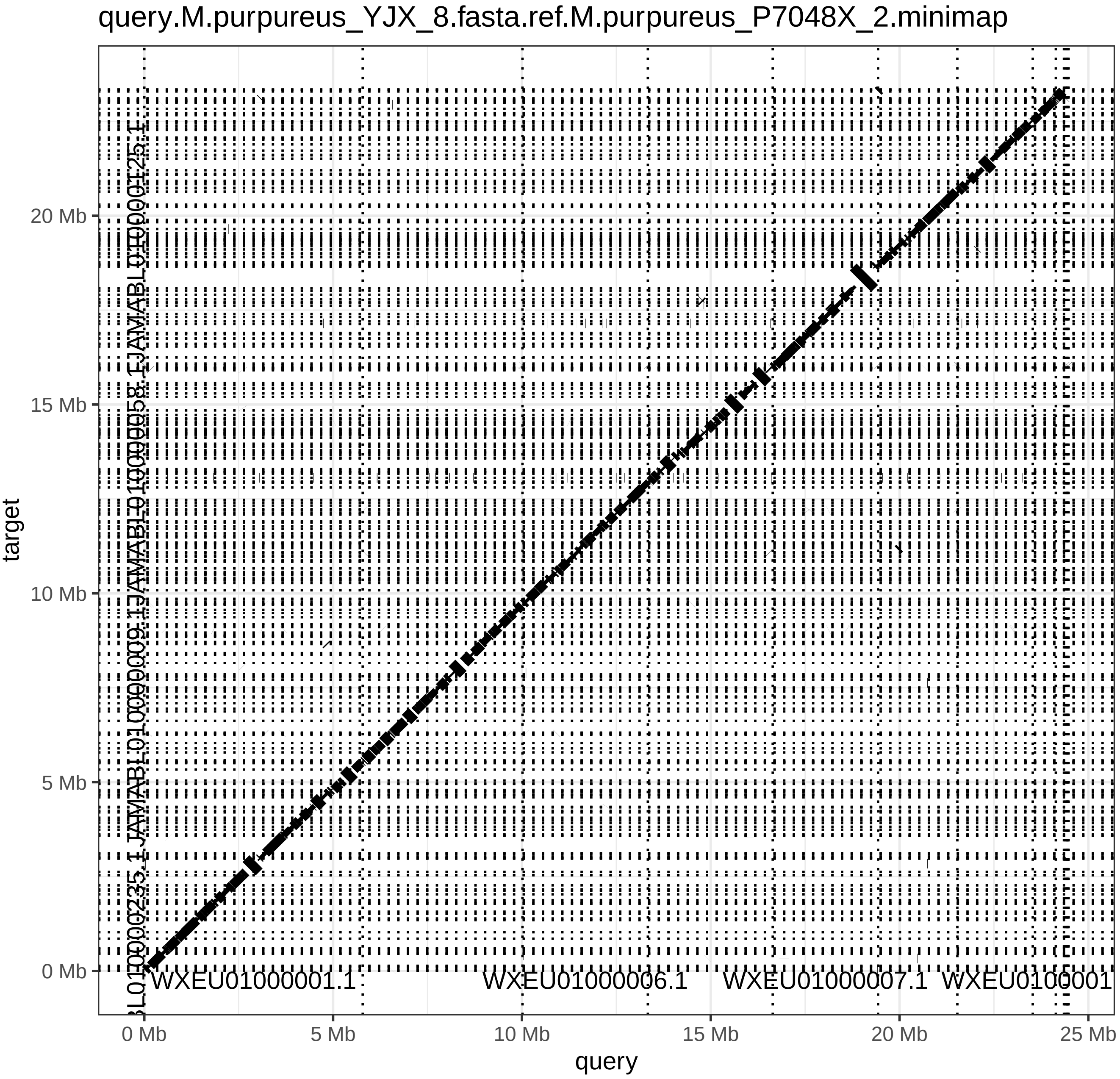

query.M.purpureus_YJX_8.fasta.ref.M.purpureus_P7048X_2.minimap
target
20 Mb
15 Mb
10 Mb
5 Mb
0 Mb
query
0 Mb
5 Mb
10 Mb
15 Mb
20 Mb
25 Mb
WXEU01000001.1
WXEU01000006.1
WXEU01000007.1

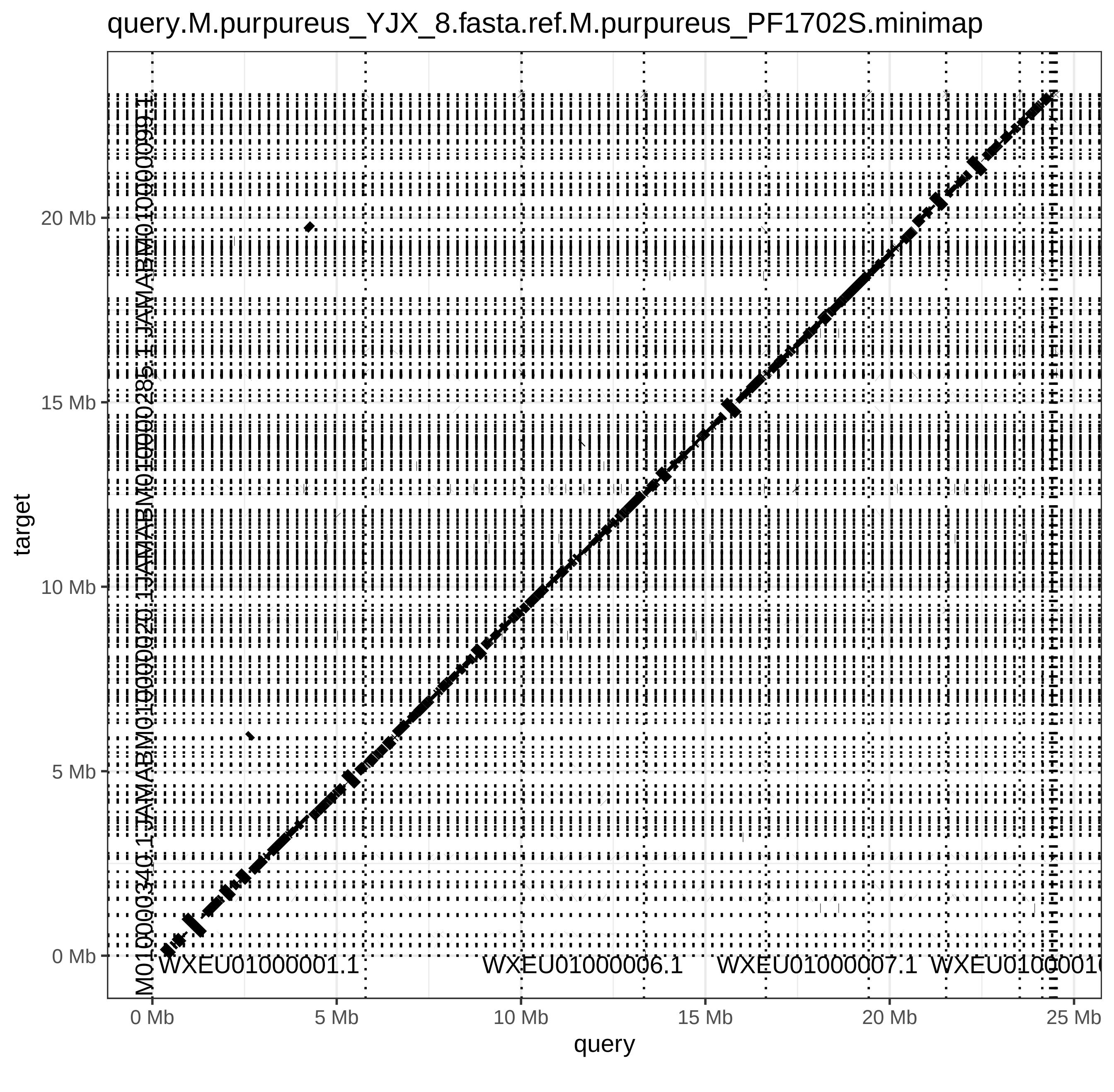
query.M.purpureus_YJX_8.fasta.ref.M.purpureus_PF1702S.minimap
target
query
0 Mb
5 Mb
10 Mb
15 Mb
20 Mb
25 Mb
WXEU01000001.1
WXEU01000006.1
WXEU01000007.1

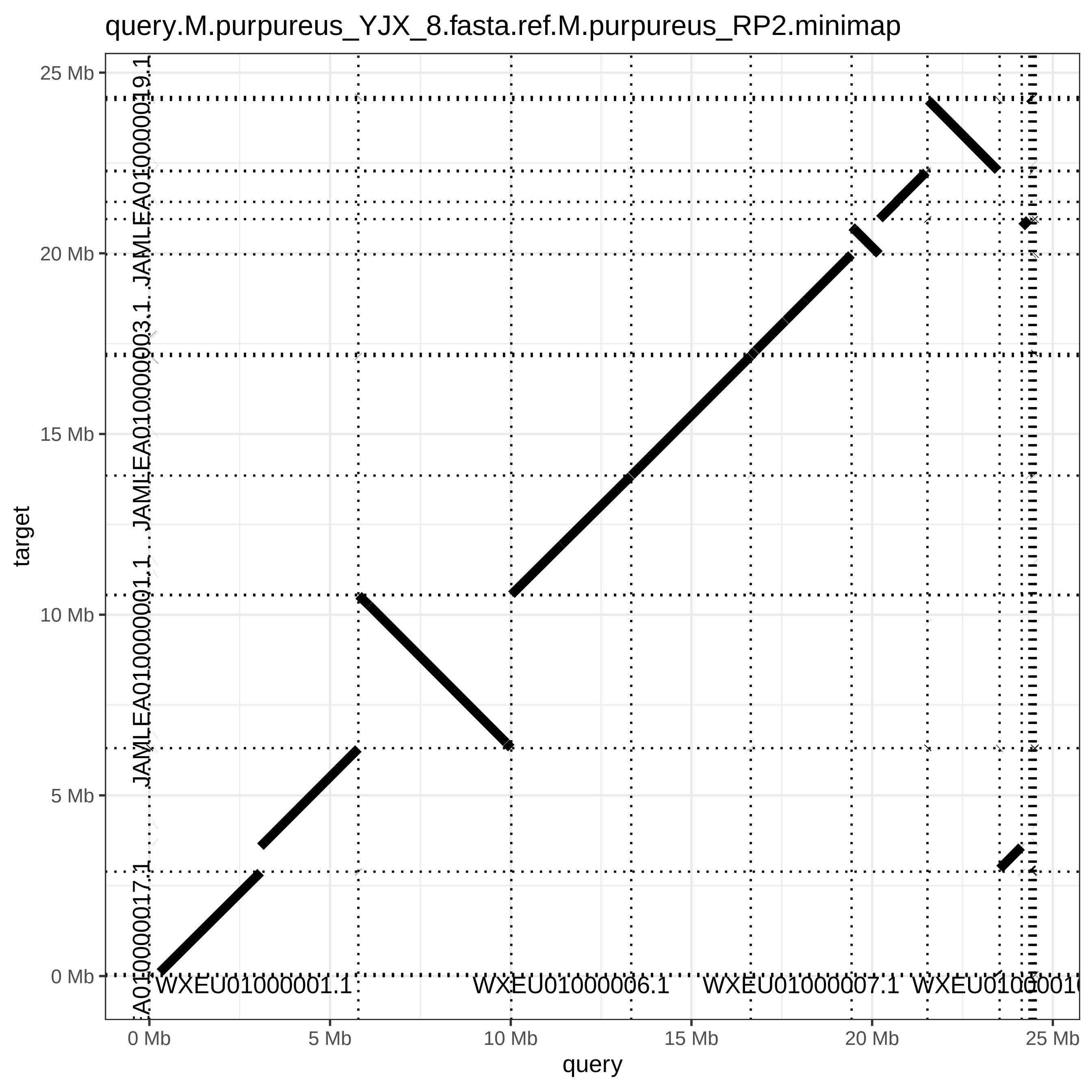
query.M.purpureus_YJX_8.fasta.ref.M.purpureus_RP2.minimap
target
query
0 Mb
5 Mb
10 Mb
15 Mb
20 Mb
25 Mb
WXEU01000001.1
WXEU01000006.1
WXEU01000007.1
JAMLEA010000017.1
JAMLEA010000001.1
JAMLEA010000003.1
JAMLEA010000019.1

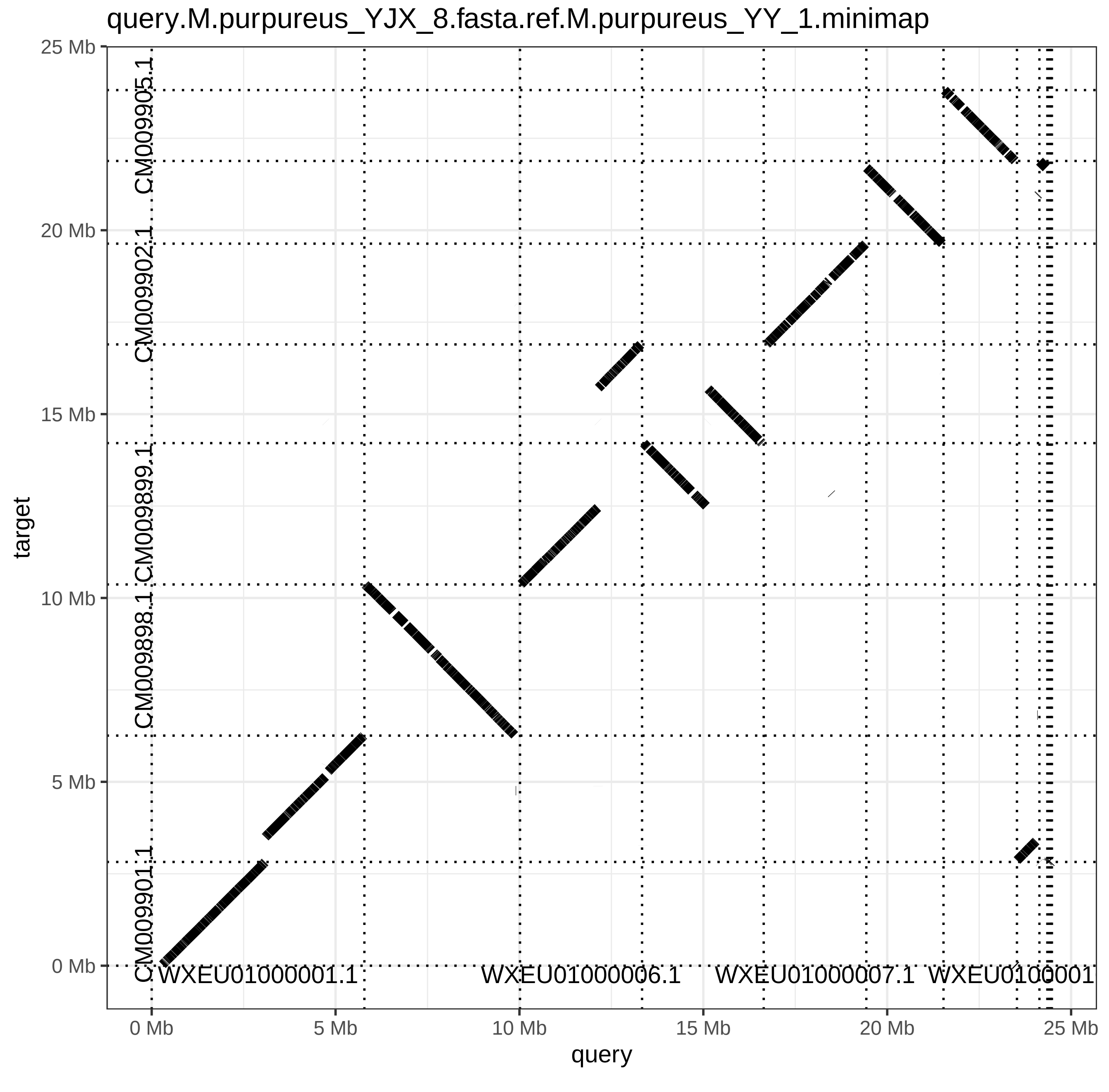

query.M.purpureus_YJX_8.fasta.ref.M.purpureus_YY_1.minimap
target
query
0 Mb
5 Mb
10 Mb
15 Mb
20 Mb
25 Mb
CM009905.1
CM009902.1
CM009899.1
CM009898.1
CM009901.1
WXEU01000001.1
WXEU01000006.1
WXEU01000007.1
WXEU0100001

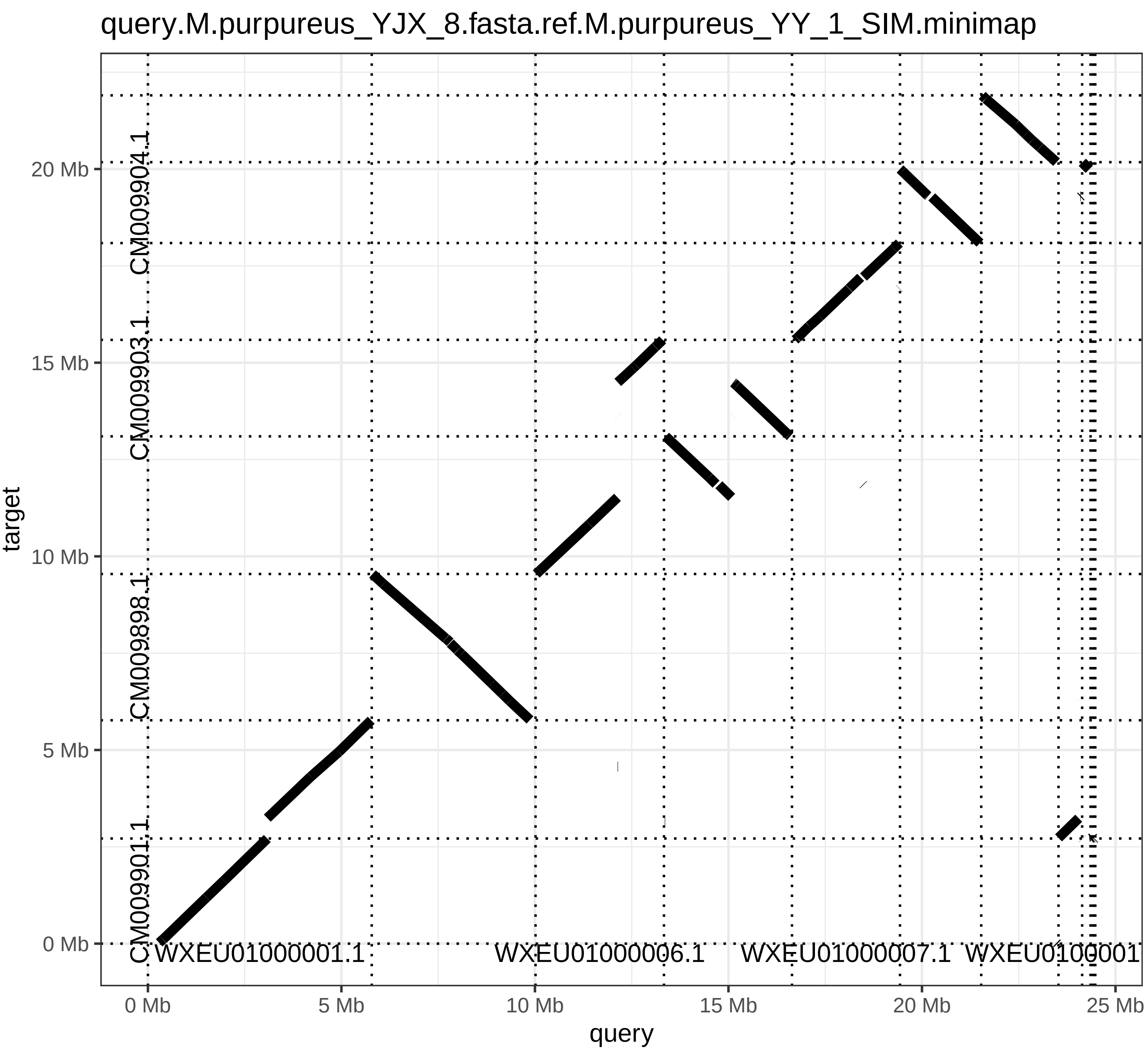
query.M.purpureus_YJX_8.fasta.ref.M.purpureus_YY_1_SIM.minimap
target
query
0 Mb
5 Mb
10 Mb
15 Mb
20 Mb
25 Mb
CM009901.1
CM009898.1
CM009903.1
CM009904.1
WXEU01000001.1
WXEU01000006.1
WXEU01000007.1

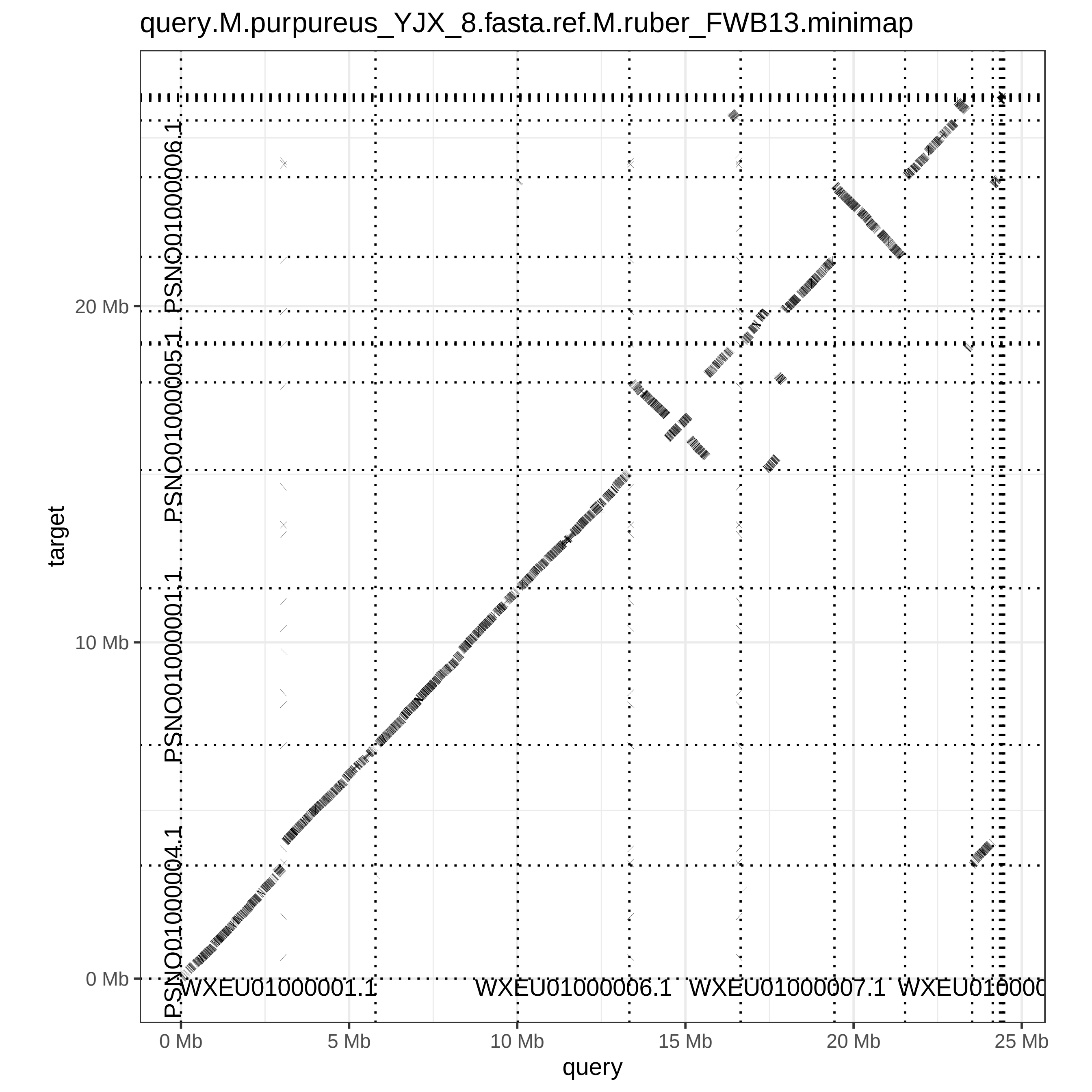
query.M.purpureus_YJX_8.fasta.ref.M.ruber_FWB13.minimap
target
query
0 Mb
10 Mb
20 Mb
5 Mb
15 Mb
25 Mb
PSNO01000006.1
PSNO01000005.1
PSNO01000001.1
PSNO01000004.1
WXEU01000001.1
WXEU01000006.1
WXEU01000007.1
WXEU010000

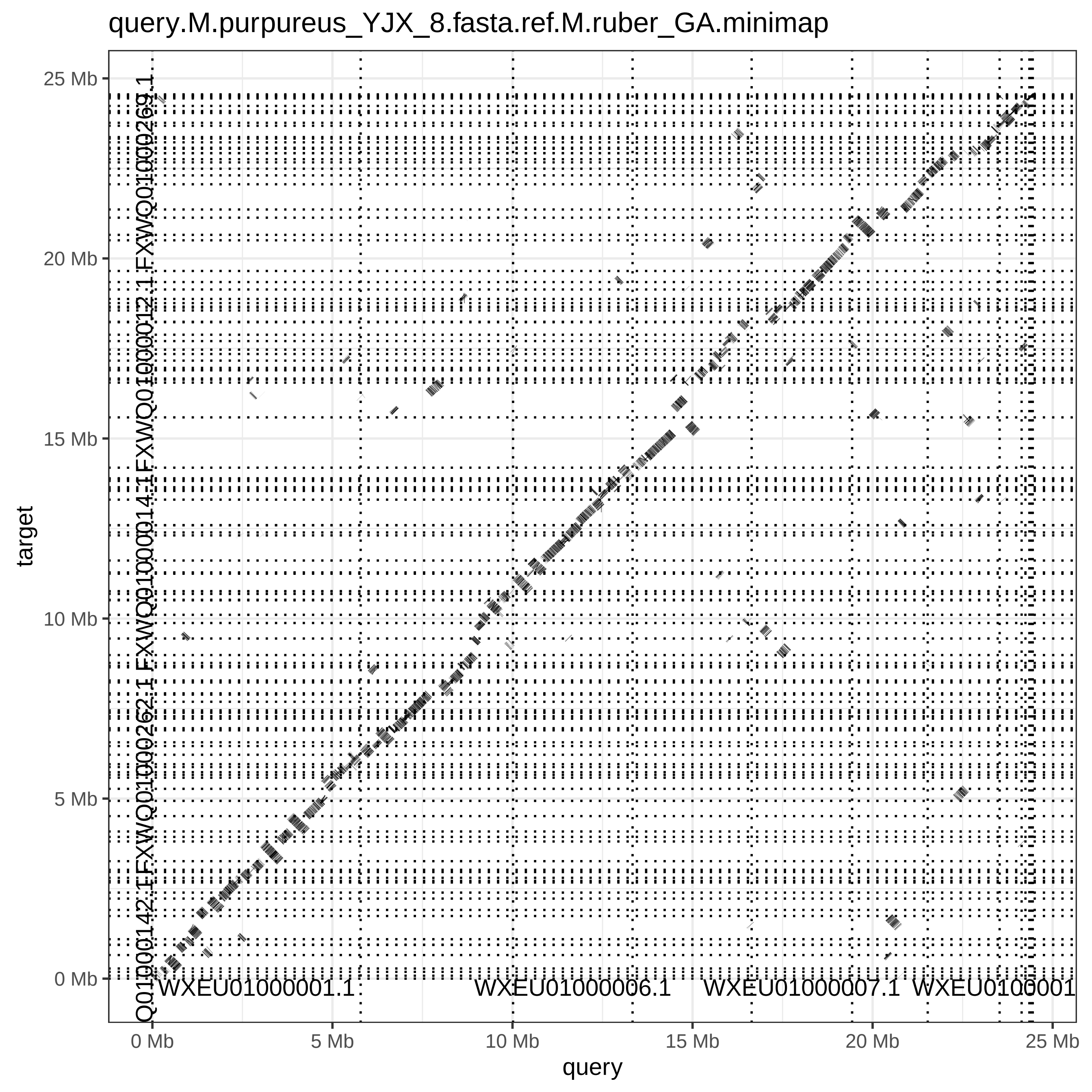
query.M.purpureus_YJX_8.fasta.ref.M.ruber_GA.minimap
target
query
25 Mb
20 Mb
15 Mb
10 Mb
5 Mb
0 Mb
0 Mb
5 Mb
10 Mb
15 Mb
20 Mb
25 Mb
WXEU01000001.1
WXEU01000006.1
WXEU01000007.1
WXEU0100001

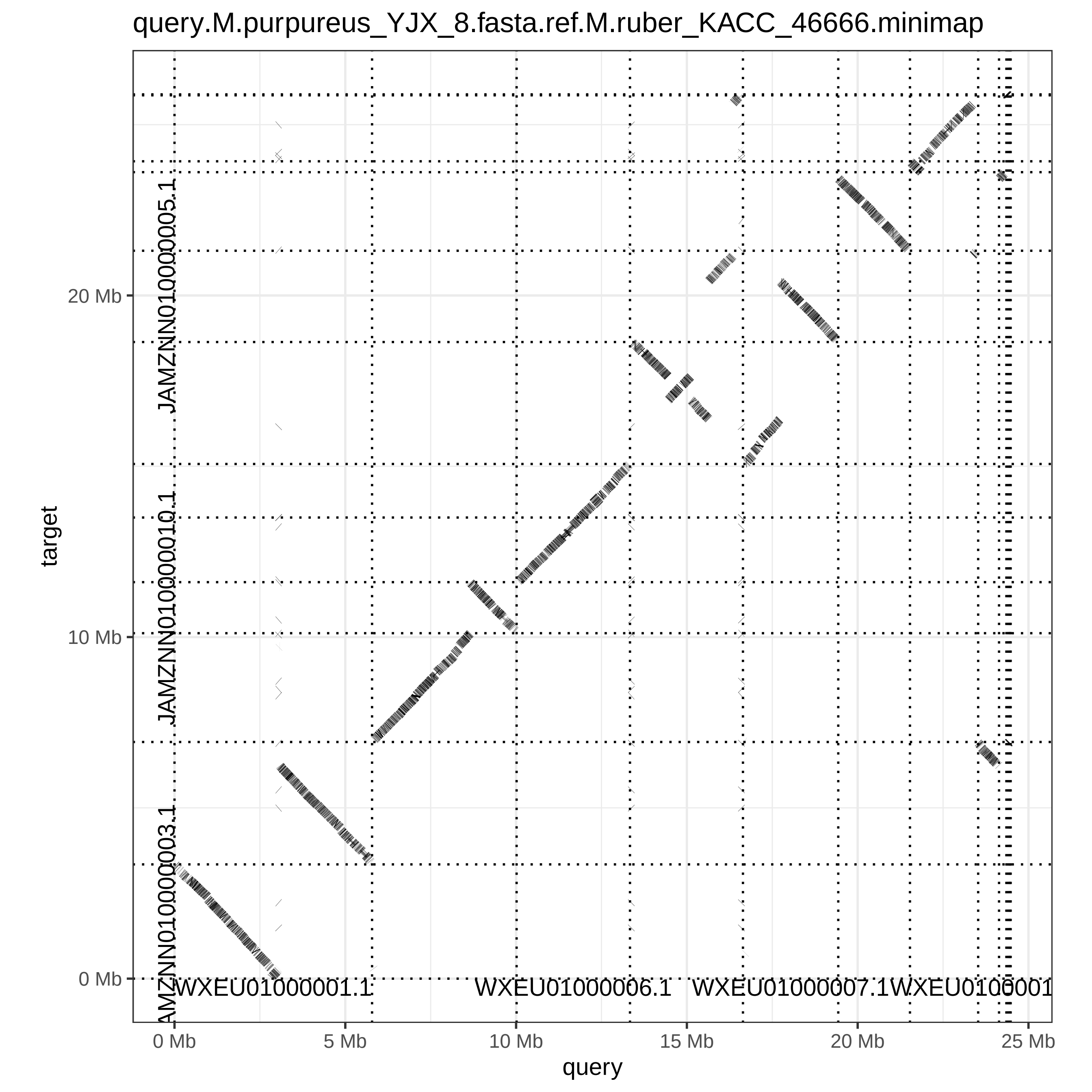

query.M.purpureus_YJX_8.fasta.ref.M.ruber_KACC_46666.minimap
target
query
0 Mb
5 Mb
10 Mb
15 Mb
20 Mb
25 Mb
0 Mb
10 Mb
20 Mb
JAMZNN010000005.1
JAMZNN010000010.1
JAMZNN010000003.1
WXEU01000001.1
WXEU01000006.1
WXEU01000007.1

query.M.purpureus_YY_1.fasta.ref.M.pilosus_K104061.minimap

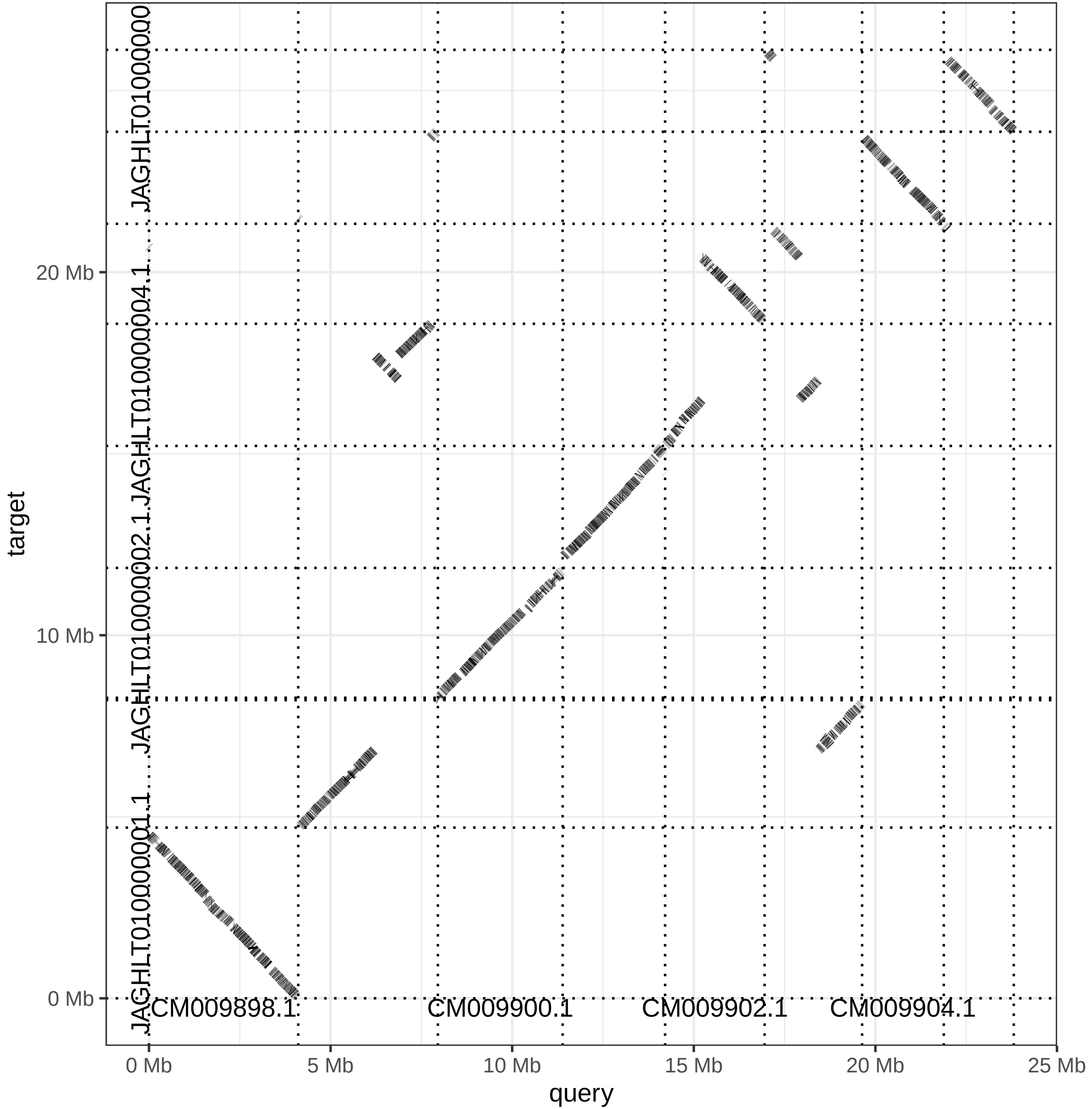

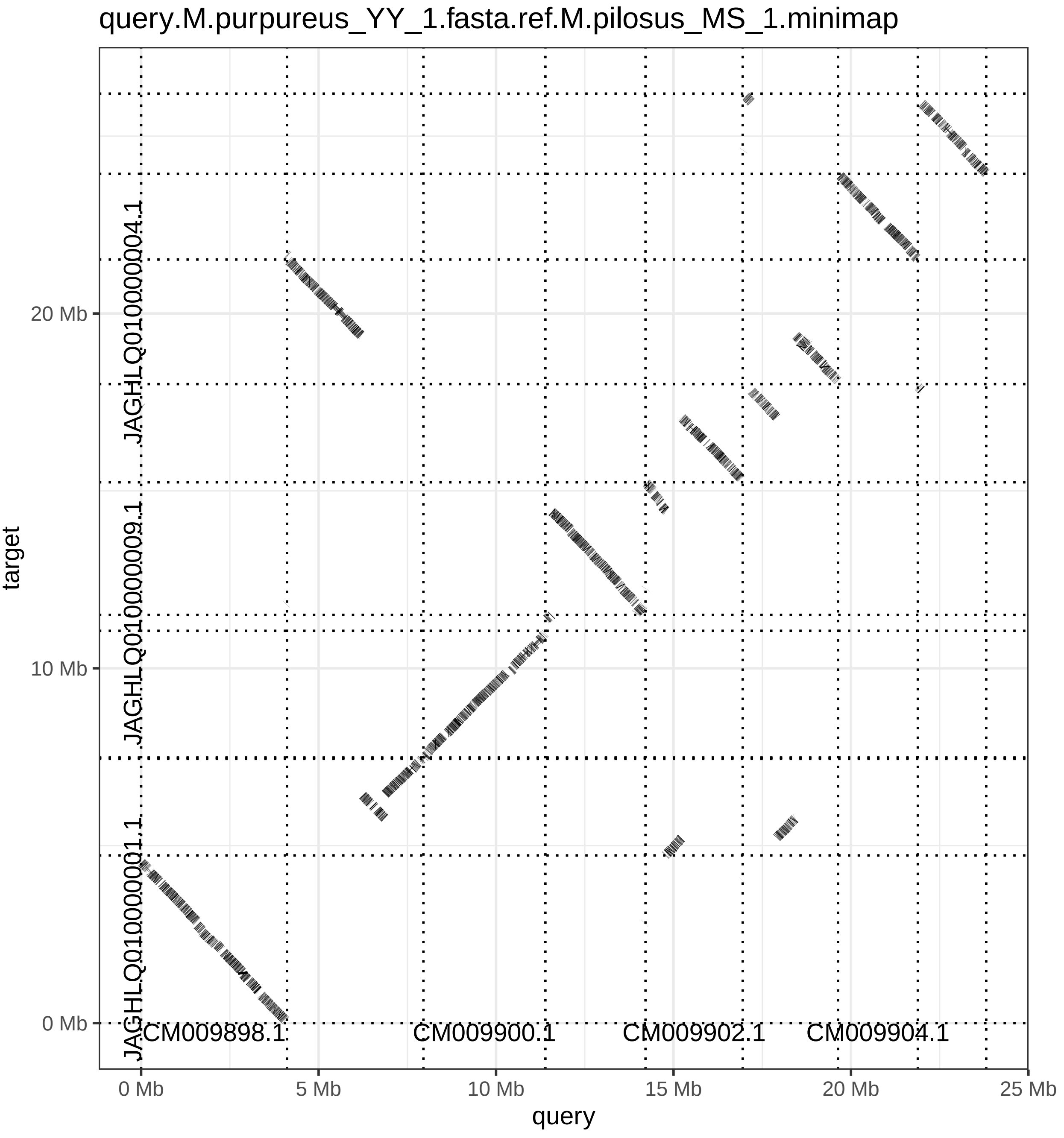

query.M.purpureus_YY_1.fasta.ref.M.pilosus_MS_1.minimap
target
query
0 Mb
10 Mb
20 Mb
0 Mb
5 Mb
10 Mb
15 Mb
20 Mb
25 Mb
JAGHLQ010000001.1
JAGHLQ010000009.1
JAGHLQ010000004.1
CM009898.1
CM009900.1
CM009902.1
CM009904.1

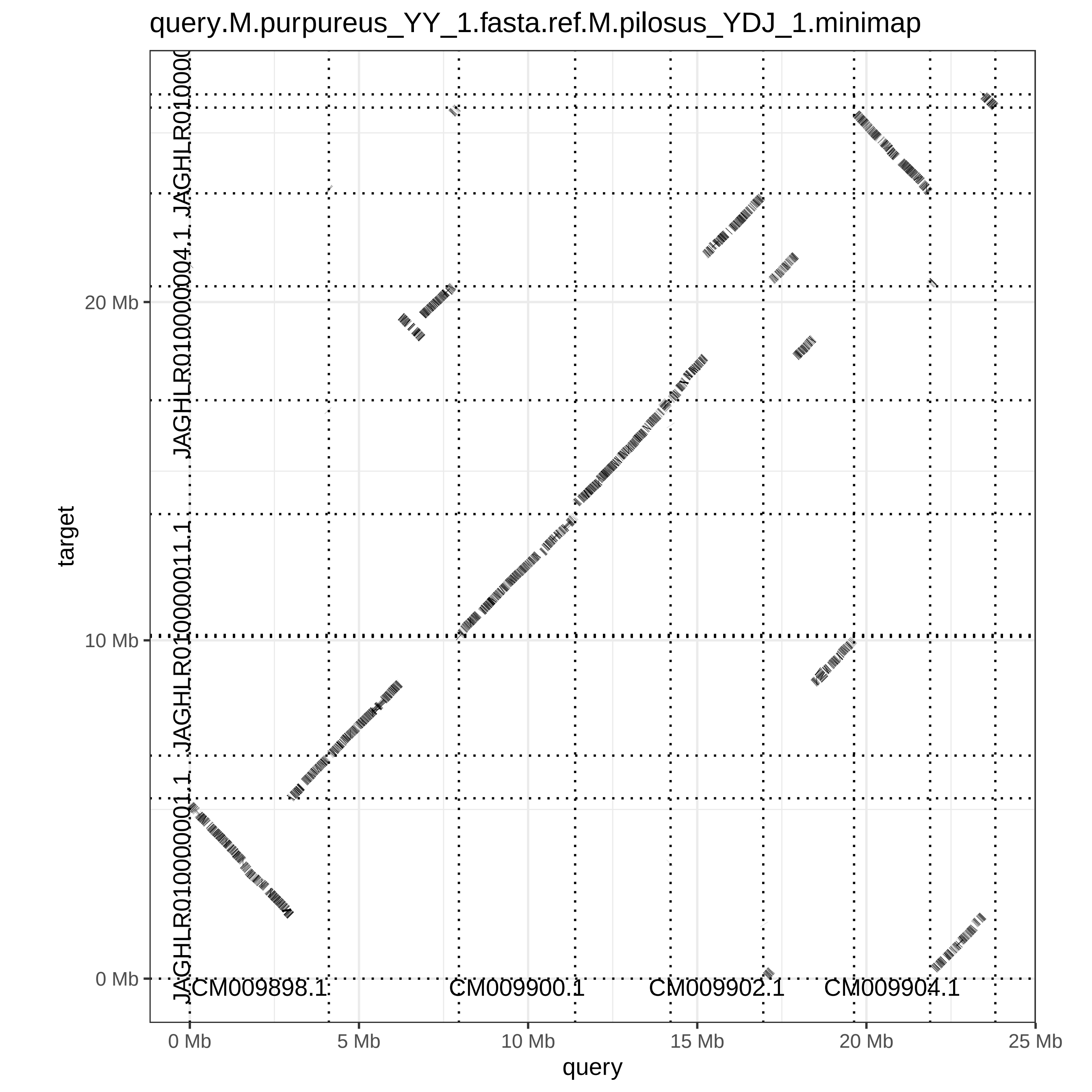

query.M.purpureus_YY_1.fasta.ref.M.pilosus_YDJ_1.minimap
target
query
0 Mb
5 Mb
10 Mb
15 Mb
20 Mb
25 Mb
0 Mb
10 Mb
20 Mb
CM009898.1
CM009900.1
CM009902.1
CM009904.1
JAGHLR010000001.1
JAGHLR010000011.1
JAGHLR010000004.1

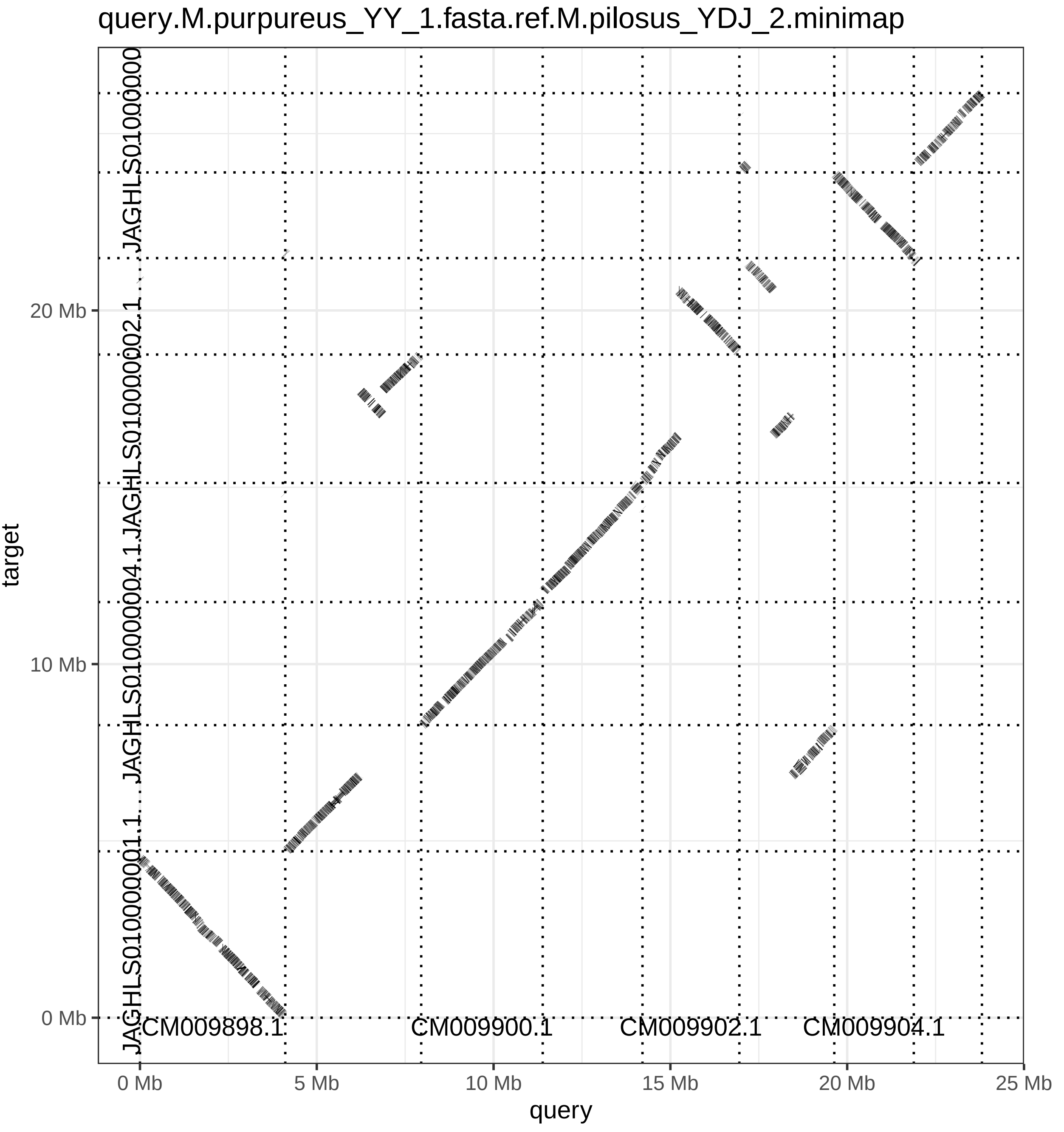
query.M.purpureus_YY_1.fasta.ref.M.pilosus_YDJ_2.minimap
target
20 Mb
10 Mb
0 Mb
JAGHLS010000001.1
JAGHLS010000004.1
JAGHLS010000002.1
CM009898.1
CM009900.1
CM009902.1
CM009904.1
0 Mb
5 Mb
10 Mb
15 Mb
20 Mb
25 Mb
query

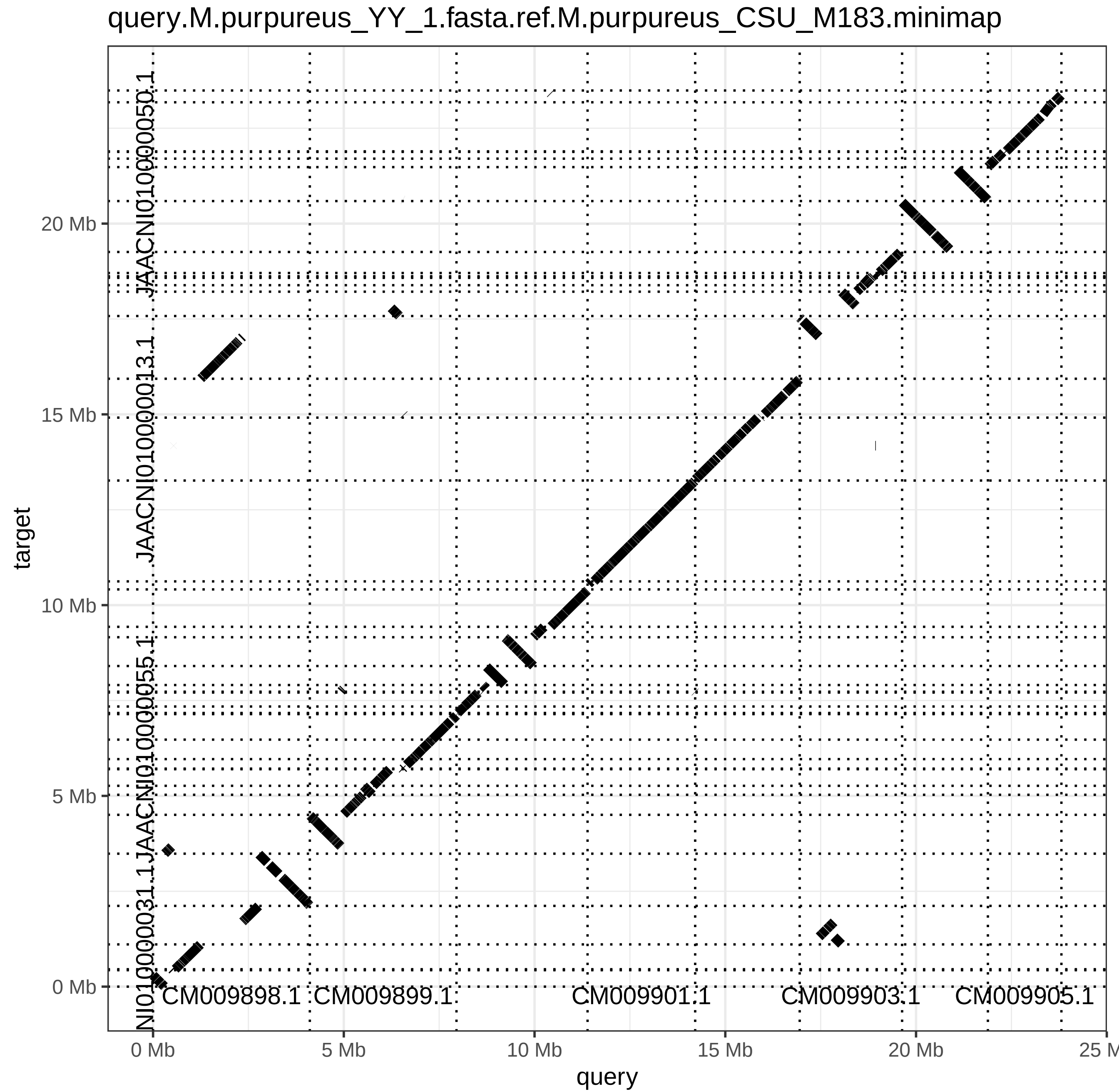

query.M.purpureus_YY_1.fasta.ref.M.purpureus_CSU_M183.minimap
target
query
0 Mb
5 Mb
10 Mb
15 Mb
20 Mb
25 Mb
JAACNI010000050.1
JAACNI010000013.1
JAACNI010000055.1
JAACNI010000031.1
CM009898.1
CM009899.1
CM009901.1
CM009903.1
CM009905.1

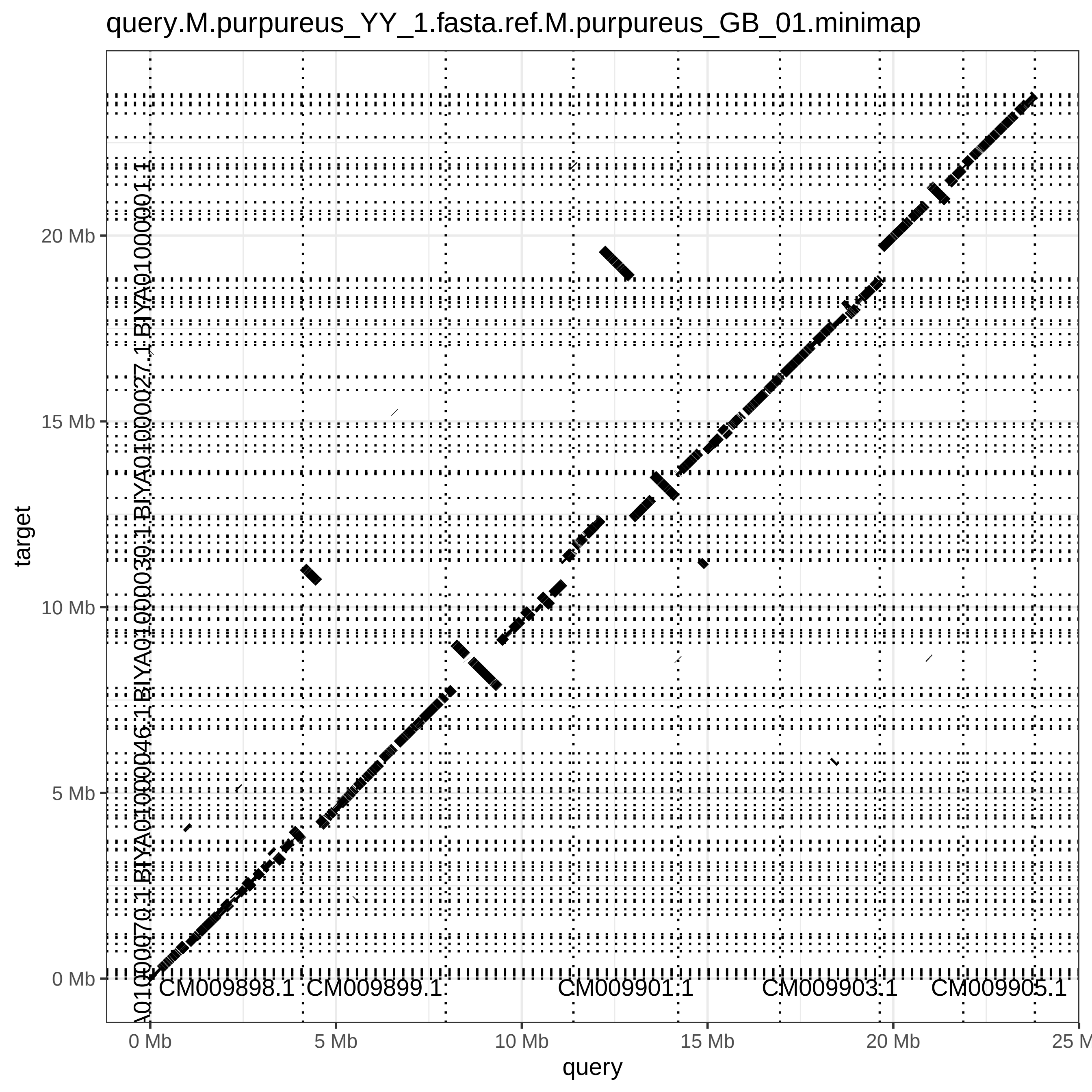
query.M.purpureus_YY_1.fasta.ref.M.purpureus_GB_01.minimap
target
query
0 Mb
5 Mb
10 Mb
15 Mb
20 Mb
25 Mb
CM009898.1
CM009899.1
CM009901.1
CM009903.1
CM009905.1

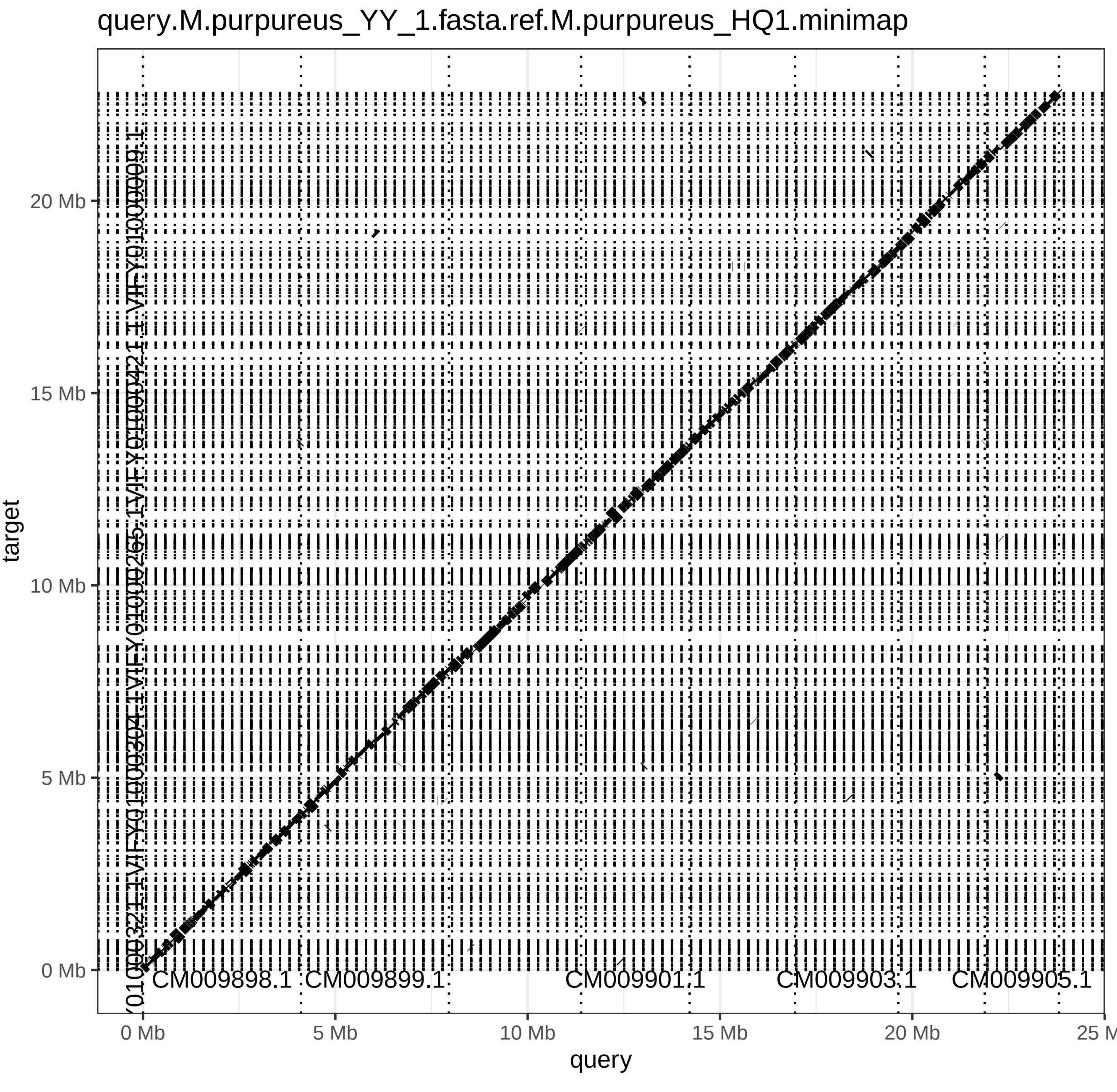
query.M.purpureus_YY_1.fasta.ref.M.purpureus_HQ1.minimap
target
20 Mb
15 Mb
10 Mb
5 Mb
0 Mb
CM009898.1
CM009899.1
CM009901.1
CM009903.1
CM009905.1
0 Mb
5 Mb
10 Mb
15 Mb
20 Mb
query

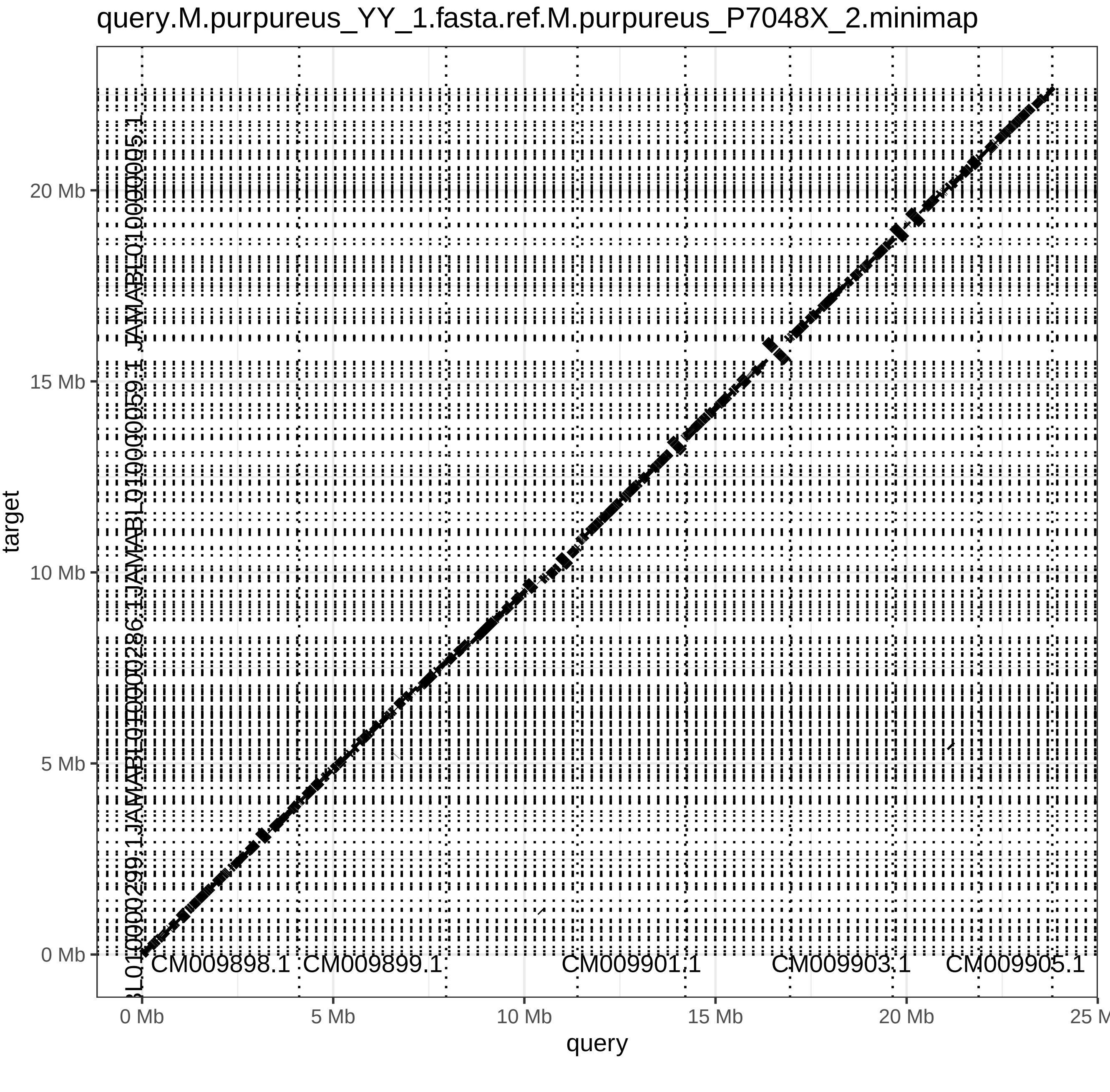

query.M.purpureus_YY_1.fasta.ref.M.purpureus_P7048X_2.minimap
target
query
0 Mb
5 Mb
10 Mb
15 Mb
20 Mb
25 Mb
CM009898.1
CM009899.1
CM009901.1
CM009903.1
CM009905.1

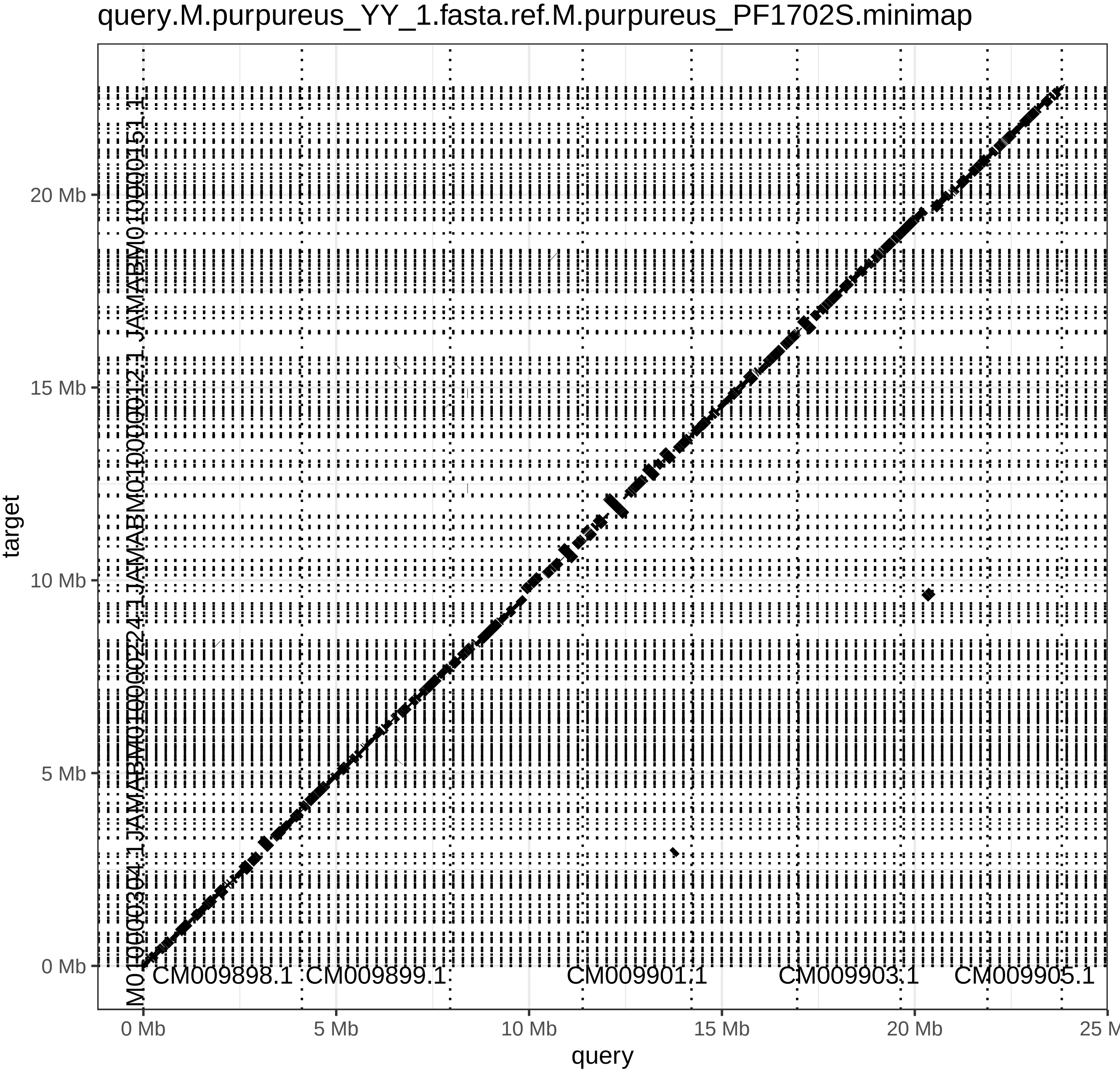
query.M.purpureus_YY_1.fasta.ref.M.purpureus_PF1702S.minimap
target
query
0 Mb
5 Mb
10 Mb
15 Mb
20 Mb
25 Mb
CM009898.1
CM009899.1
CM009901.1
CM009903.1
CM009905.1

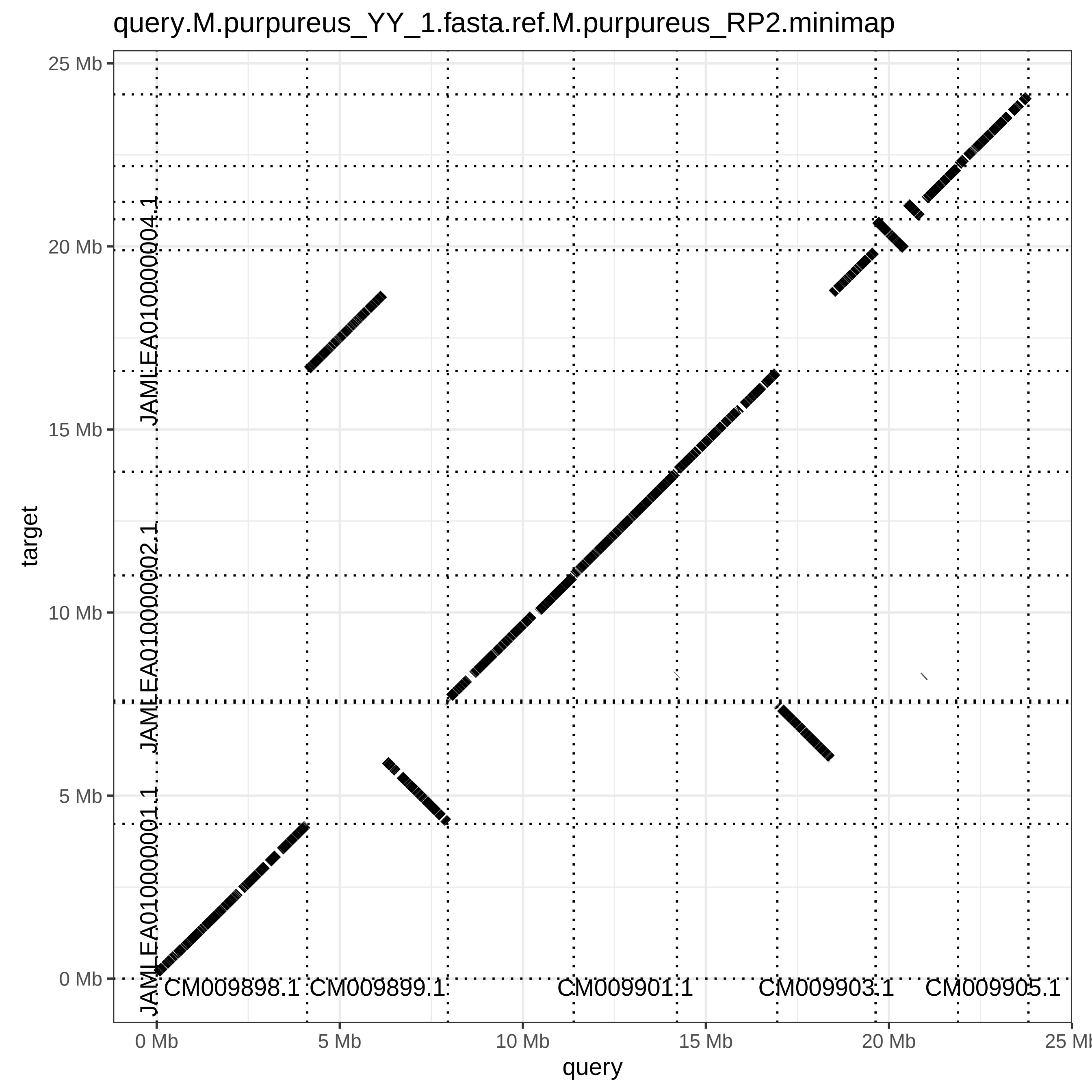

query.M.purpureus_YY_1.fasta.ref.M.purpureus_RP2.minimap
target
query
25 Mb
20 Mb
15 Mb
10 Mb
5 Mb
0 Mb
0 Mb
5 Mb
10 Mb
15 Mb
20 Mb
25 Mb
JAMLEA010000001.1
JAMLEA010000002.1
JAMLEA010000004.1
CM009898.1
CM009899.1
CM009901.1
CM009903.1
CM009905.1

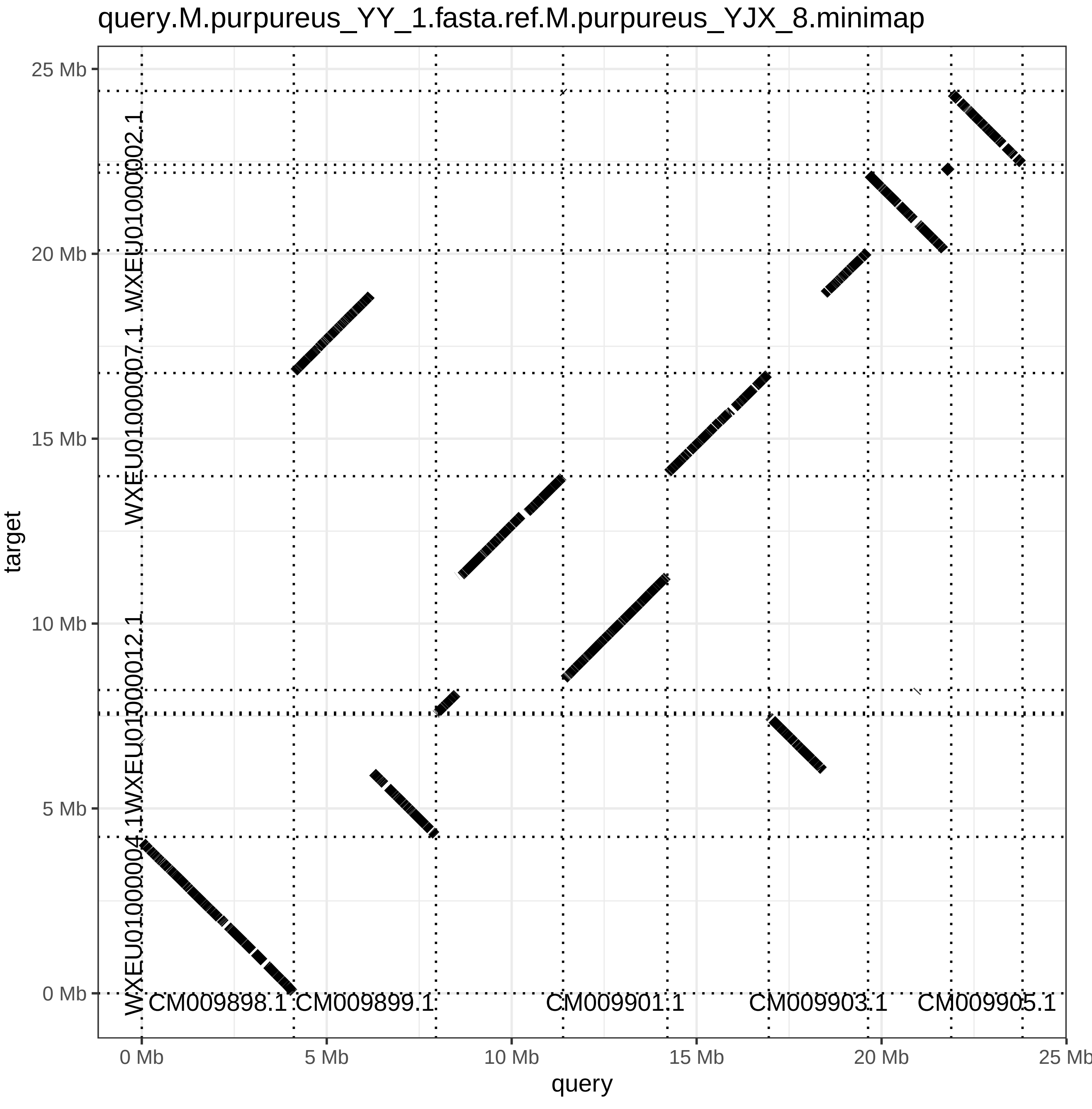
query.M.purpureus_YY_1.fasta.ref.M.purpureus_YJX_8.minimap
target
query
25 Mb
20 Mb
15 Mb
10 Mb
5 Mb
0 Mb
0 Mb
5 Mb
10 Mb
15 Mb
20 Mb
25 Mb
WXEU01000002.1
WXEU01000007.1
WXEU01000012.1
WXEU01000004.1
CM009898.1
CM009899.1
CM009901.1
CM009903.1
CM009905.1

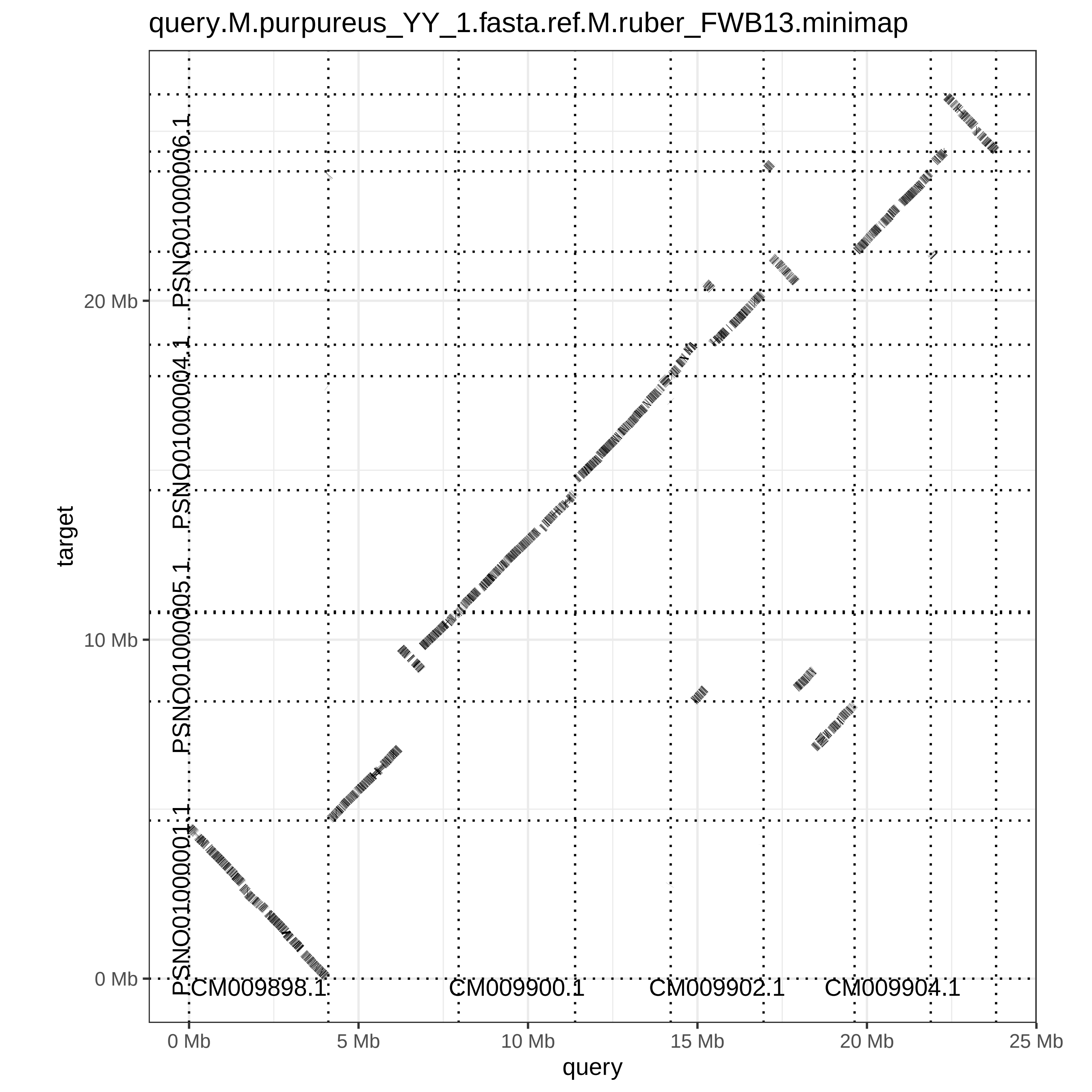
query.M.purpureus_YY_1.fasta.ref.M.ruber_FWB13.minimap
target
query
PSNO01000001.1
PSNO01000005.1
PSNO01000004.1
PSNO01000006.1
CM009898.1
CM009900.1
CM009902.1
CM009904.1
0 Mb
10 Mb
20 Mb
0 Mb
5 Mb
10 Mb
15 Mb
20 Mb
25 Mb

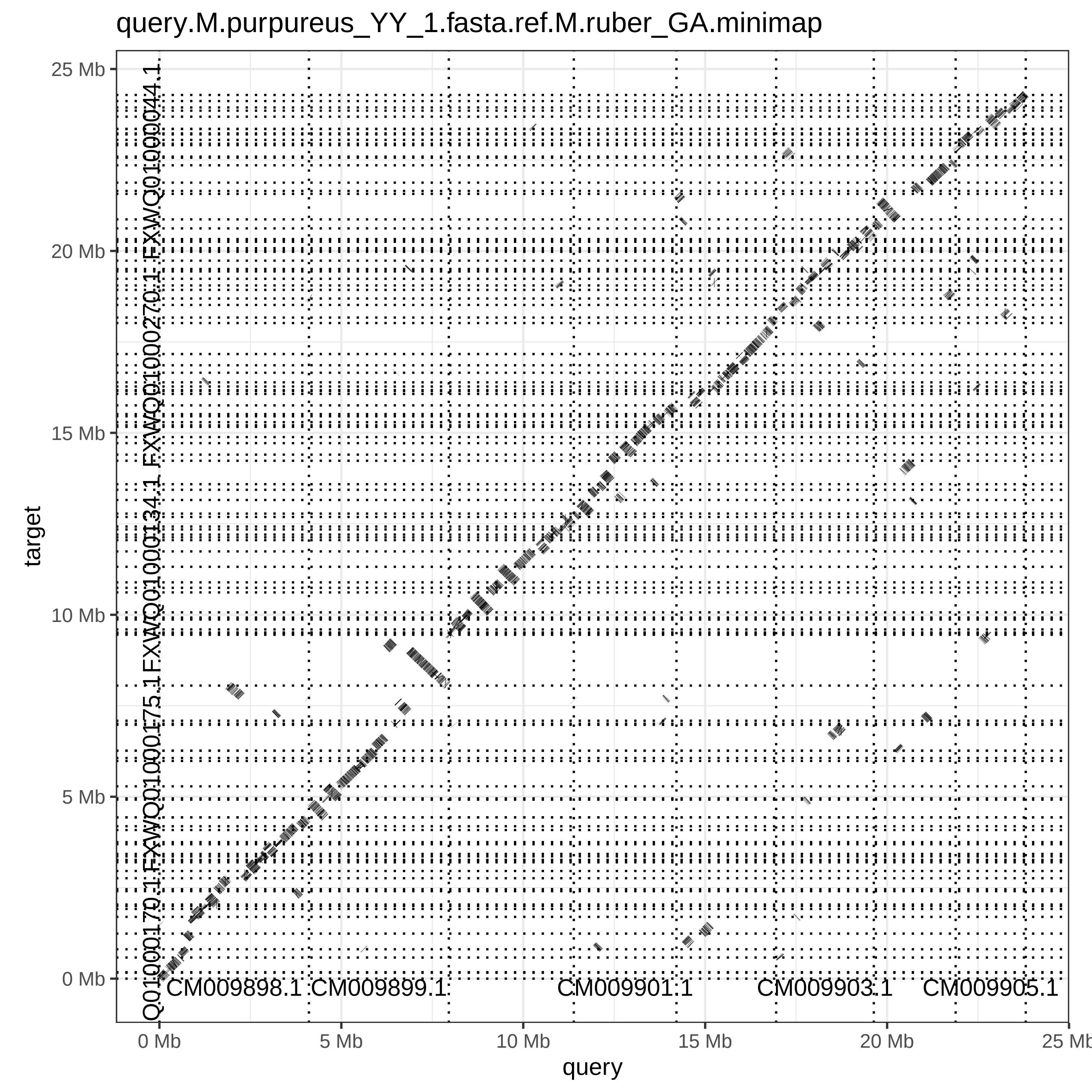
query.M.purpureus_YY_1.fasta.ref.M.ruber_GA.minimap
target
query
25 Mb
20 Mb
15 Mb
10 Mb
5 Mb
0 Mb
0 Mb
5 Mb
10 Mb
15 Mb
20 Mb
25 Mb
CM009898.1
CM009899.1
CM009901.1
CM009903.1
CM009905.1
Q01000170.1 FXWQ01000175.1 FXWQ01000134.1 FXWQ01000270.1 FXWQ01000044.1

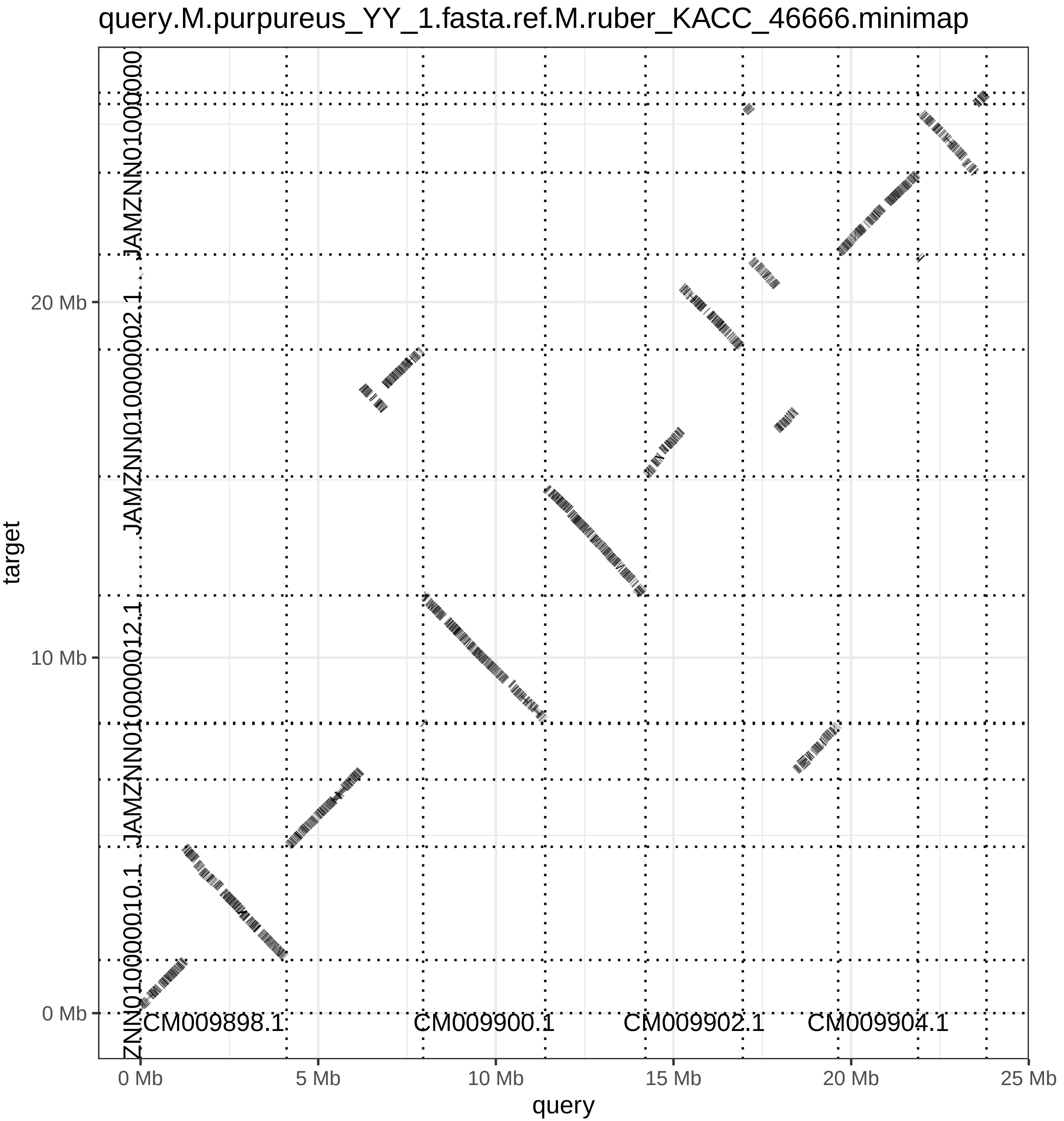
query.M.purpureus_YY_1.fasta.ref.M.ruber_KACC_46666.minimap
target
20 Mb
10 Mb
0 Mb
JAMZNN010000000
JAMZNN010000002.1
JAMZNN010000012.1
ZNN010000010.1
CM009898.1
CM009900.1
CM009902.1
CM009904.1
0 Mb
5 Mb
10 Mb
15 Mb
20 Mb
25 Mb
query

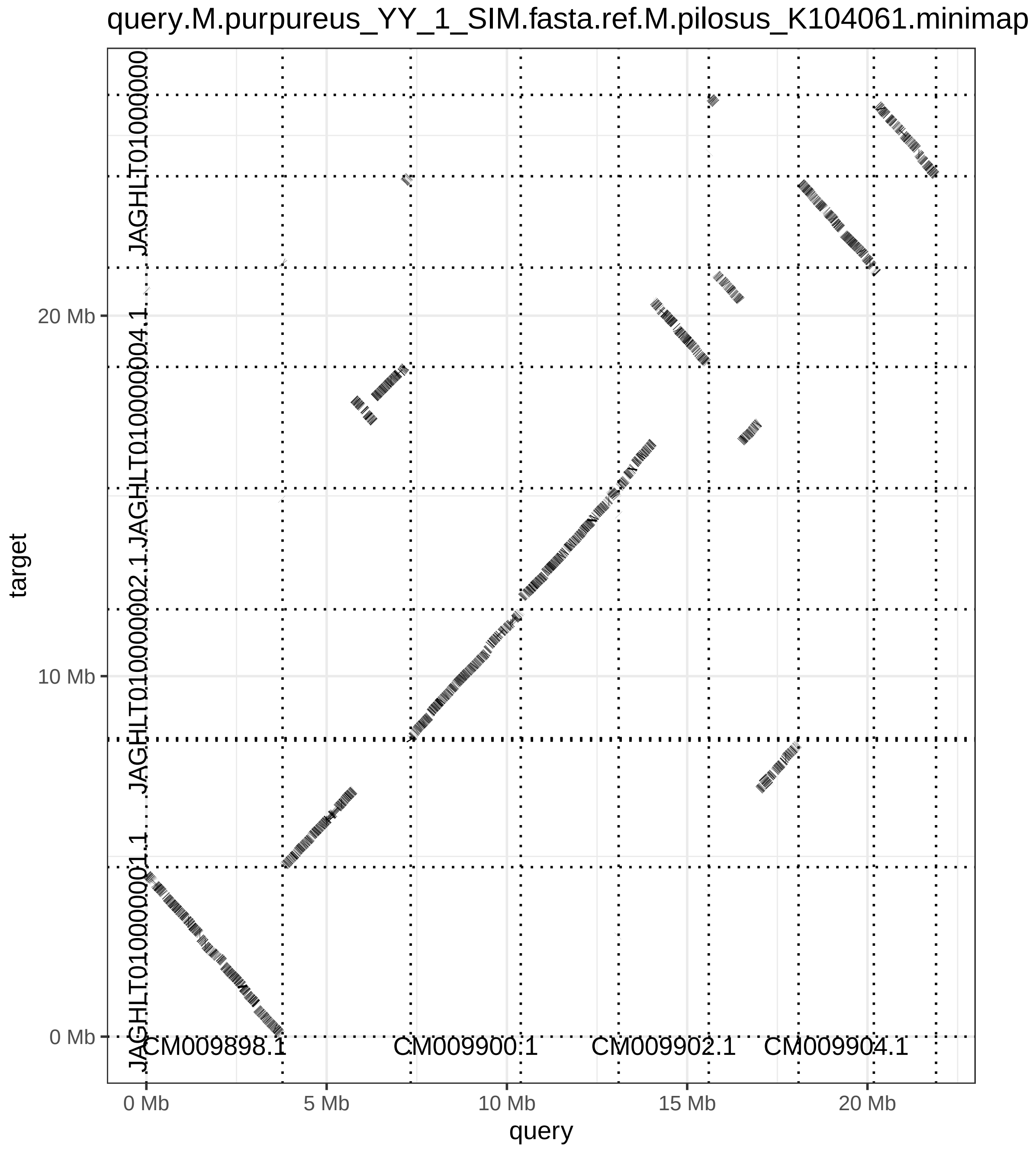

query.M.purpureus_YY_1_SIM.fasta.ref.M.pilosus_K104061.minimap
target
query
0 Mb
10 Mb
20 Mb
0 Mb
5 Mb
10 Mb
15 Mb
20 Mb
CM009898.1
CM009900.1
CM009902.1
CM009904.1
JAGHLT010000001.1
JAGHLT010000002.1
JAGHLT010000004.1
JAGHLT0100000

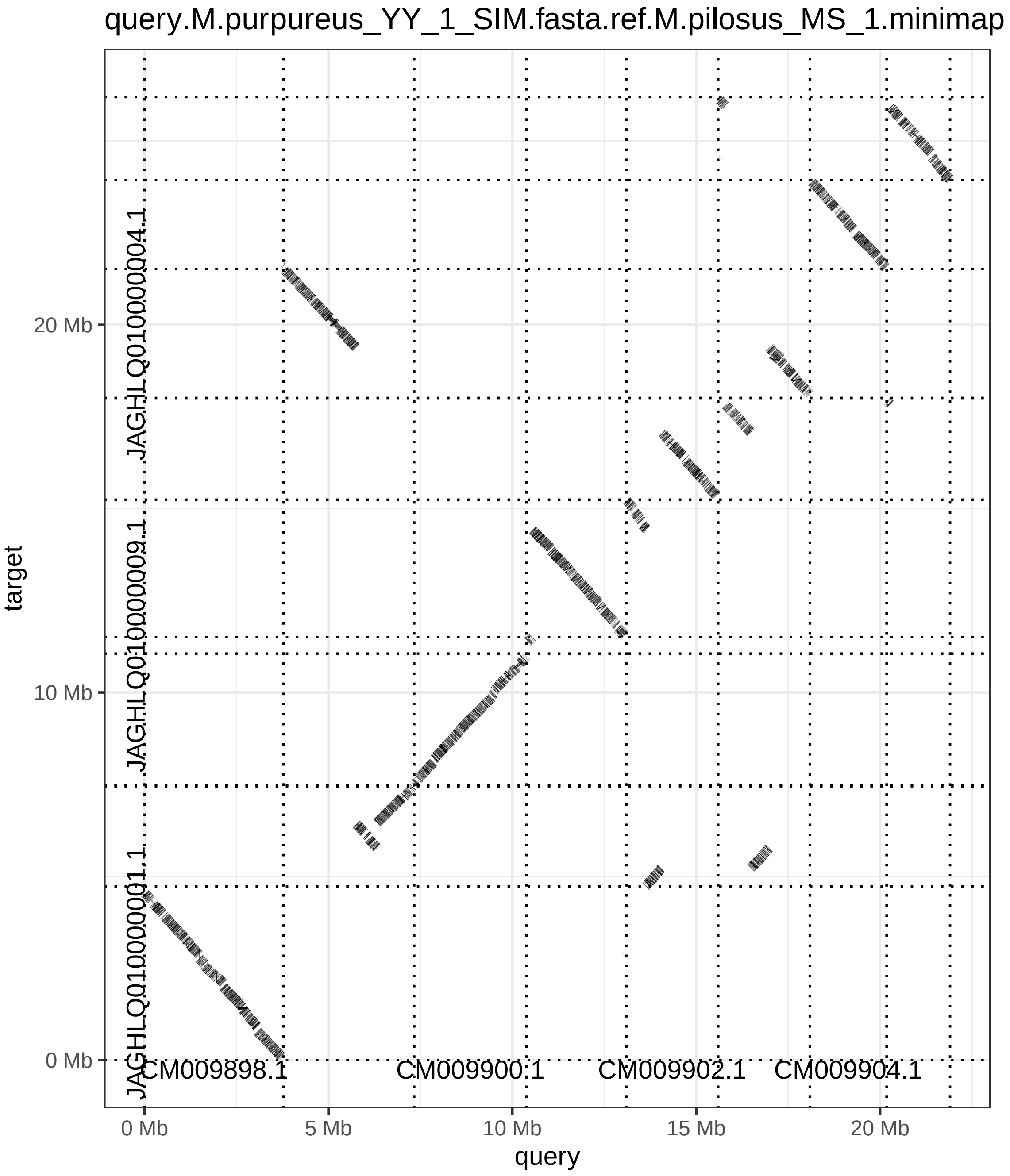
query.M.purpureus_YY_1_SIM.fasta.ref.M.pilosus_MS_1.minimap
target
query
0 Mb
10 Mb
20 Mb
0 Mb
5 Mb
10 Mb
15 Mb
20 Mb
JAGHLQ010000001.1
JAGHLQ010000009.1
JAGHLQ010000004.1
CM009898.1
CM009900.1
CM009902.1
CM009904.1

query.M.purpureus_YY_1_SIM.fasta.ref.M.pilosus_YDJ_1.minimap

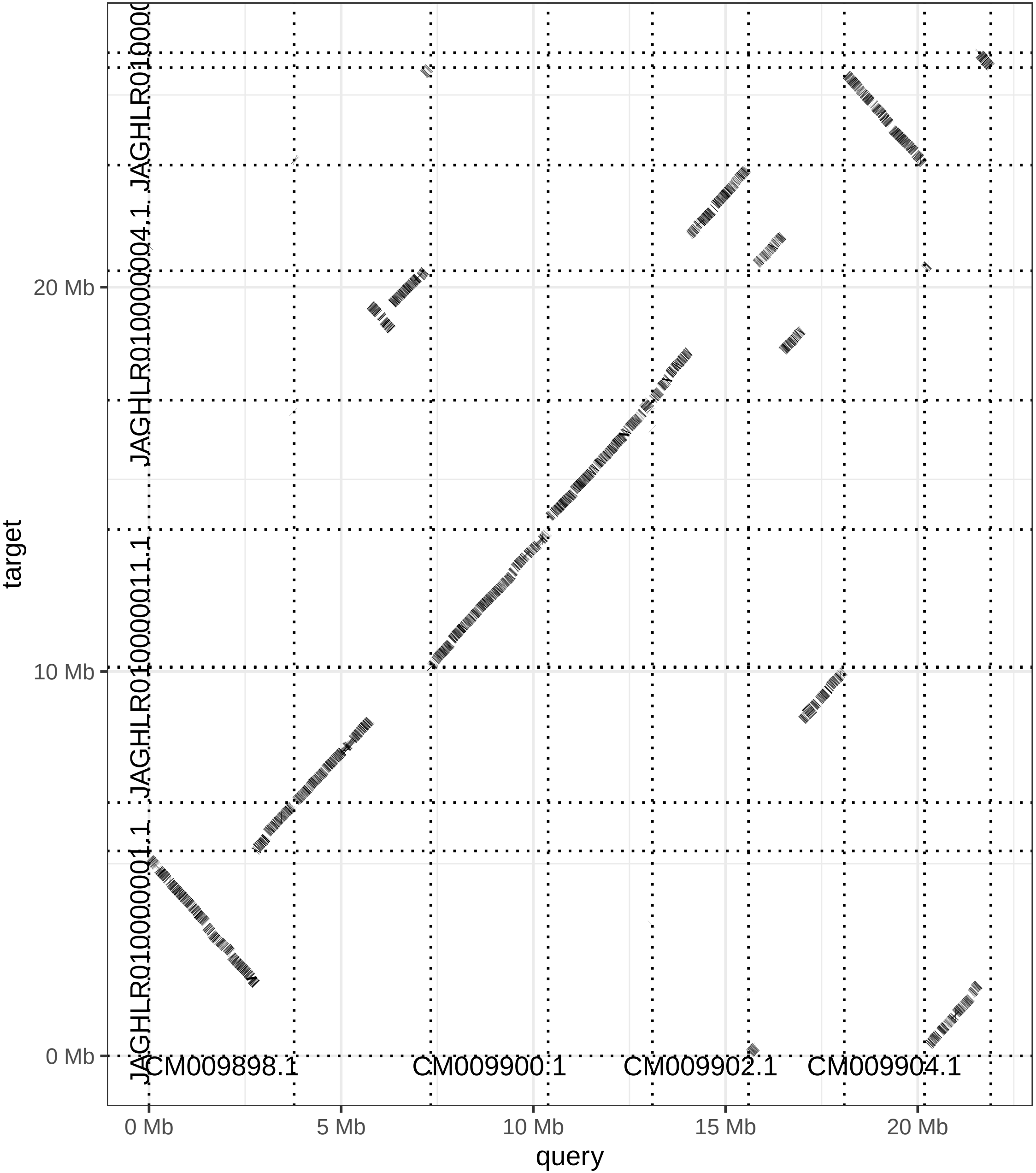

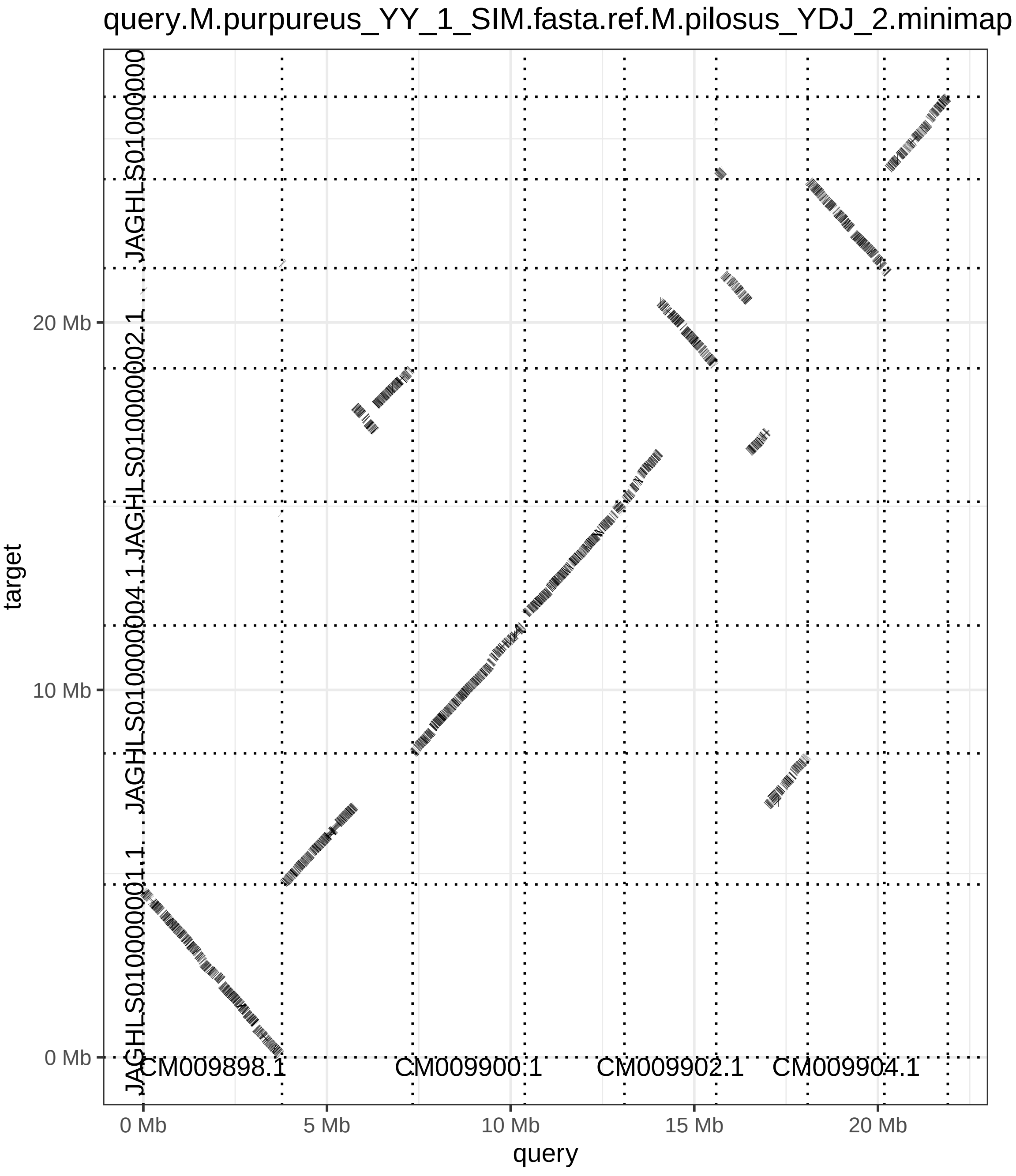
query.M.purpureus_YY_1_SIM.fasta.ref.M.pilosus_YDJ_2.minimap
target
query
0 Mb
10 Mb
20 Mb
5 Mb
15 Mb
CM009898.1
CM009900.1
CM009902.1
CM009904.1
JAGHLS010000001.1
JAGHLS010000004.1
JAGHLS010000002.1

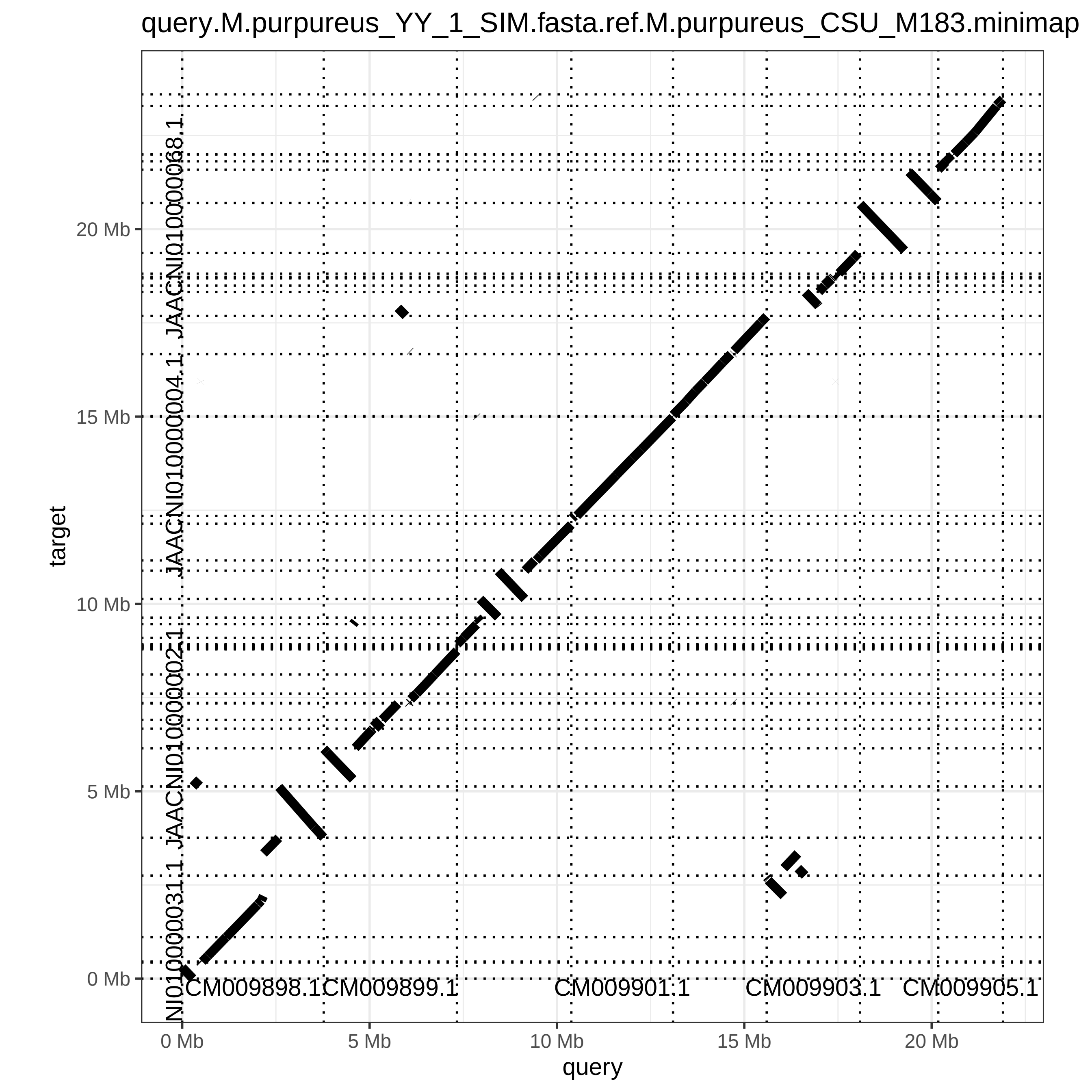

query.M.purpureus_YY_1_SIM.fasta.ref.M.purpureus_CSU_M183.minimap
target
query
0 Mb
5 Mb
10 Mb
15 Mb
20 Mb
CM009898.1
CM009899.1
CM009901.1
CM009903.1
CM009905.1
JAACNI010000068.1
JAACNI010000004.1
JAACNI010000002.1

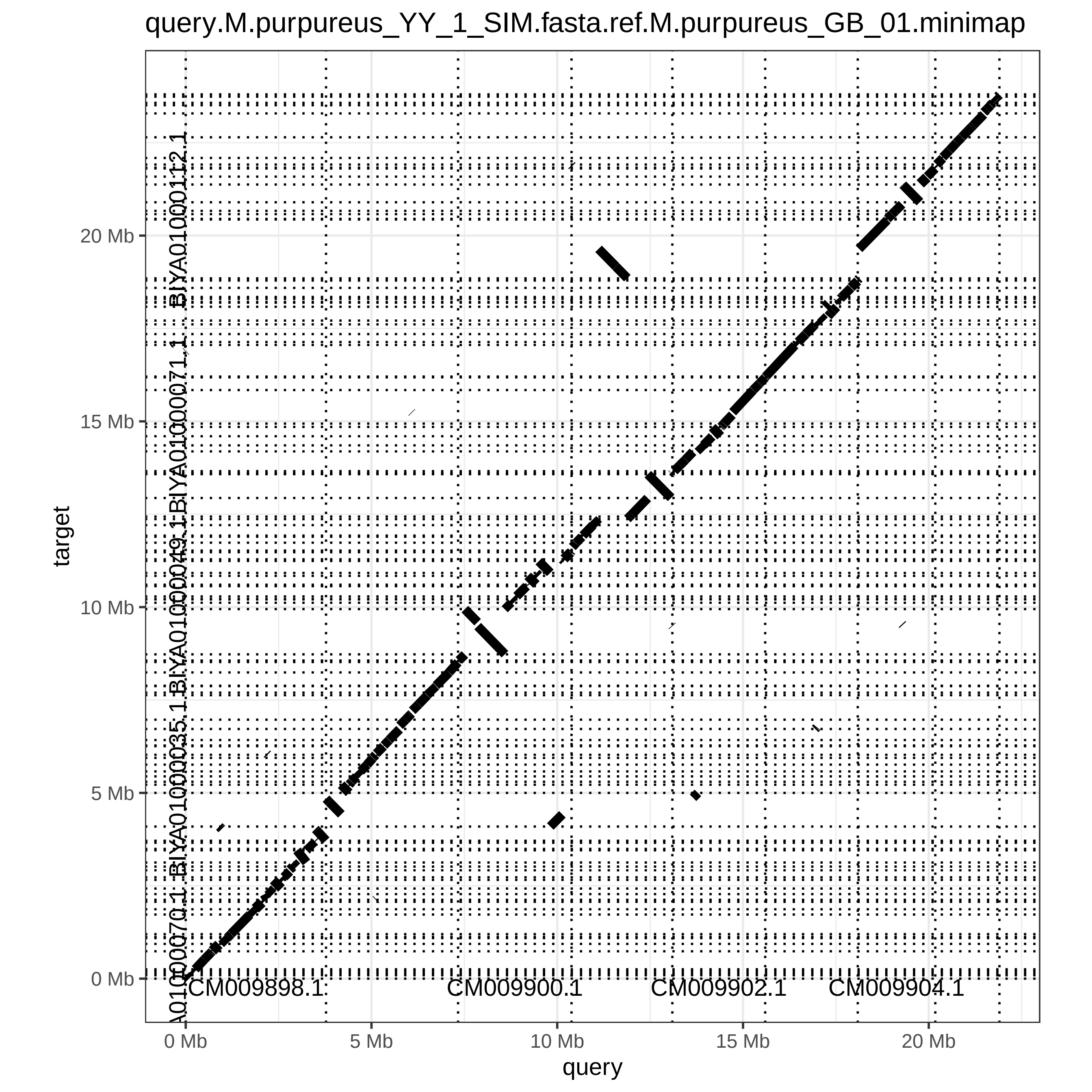

query.M.purpureus_YY_1_SIM.fasta.ref.M.purpureus_GB_01.minimap
target
query
0 Mb
5 Mb
10 Mb
15 Mb
20 Mb
CM009898.1
CM009900.1
CM009902.1
CM009904.1
BIYA010000112.1
BIYA010000071.1
BIYA010000049.1
BIYA010000035.1

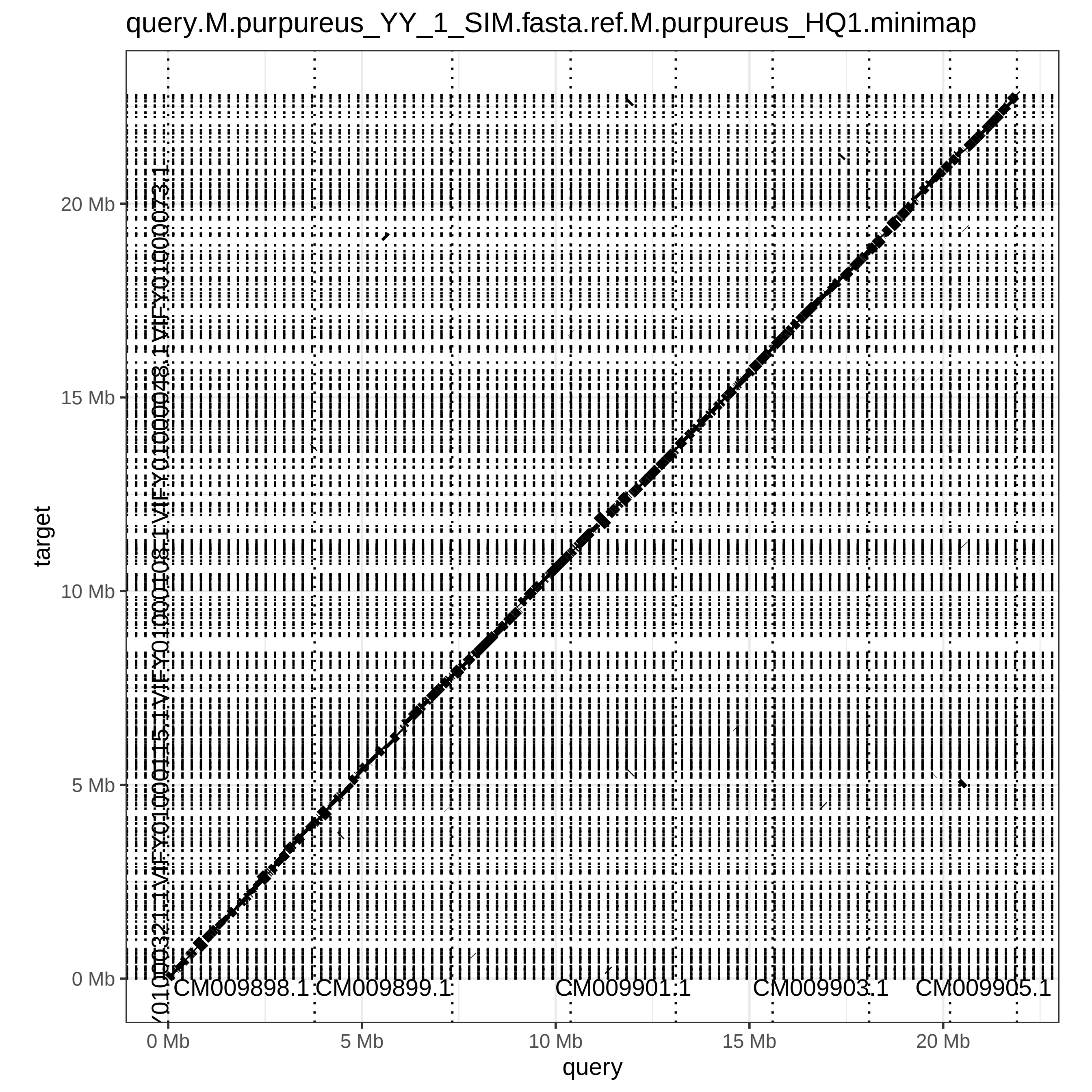
query.M.purpureus_YY_1_SIM.fasta.ref.M.purpureus_HQ1.minimap
target
20 Mb
15 Mb
10 Mb
5 Mb
0 Mb
CM009898.1
CM009899.1
CM009901.1
CM009903.1
CM009905.1
0 Mb
5 Mb
10 Mb
15 Mb
20 Mb
query

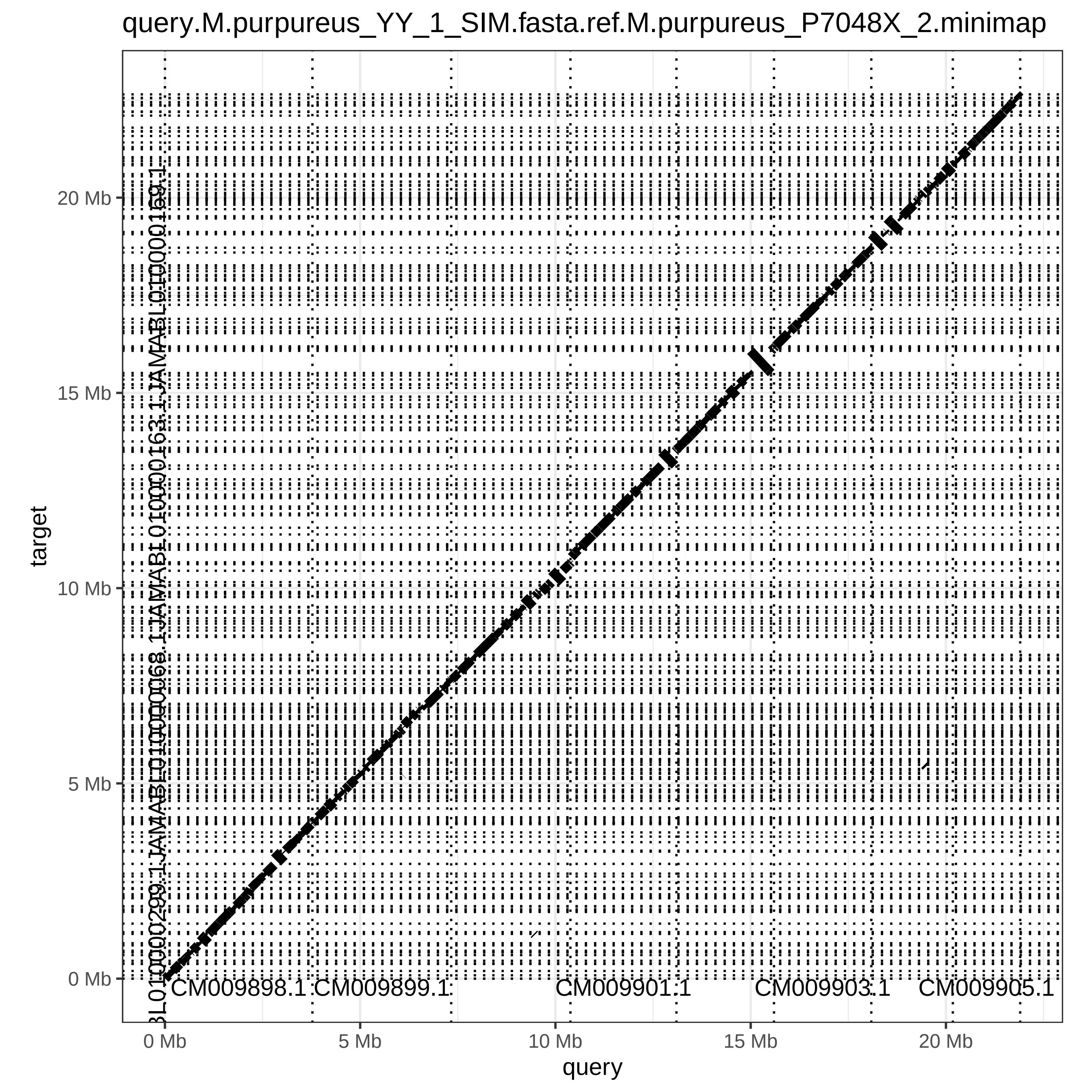
query.M.purpureus_YY_1_SIM.fasta.ref.M.purpureus_P7048X_2.minimap
target
20 Mb
15 Mb
10 Mb
5 Mb
0 Mb
0 Mb
5 Mb
10 Mb
15 Mb
20 Mb
query
CM009898.1
CM009899.1
CM009901.1
CM009903.1
CM009905.1

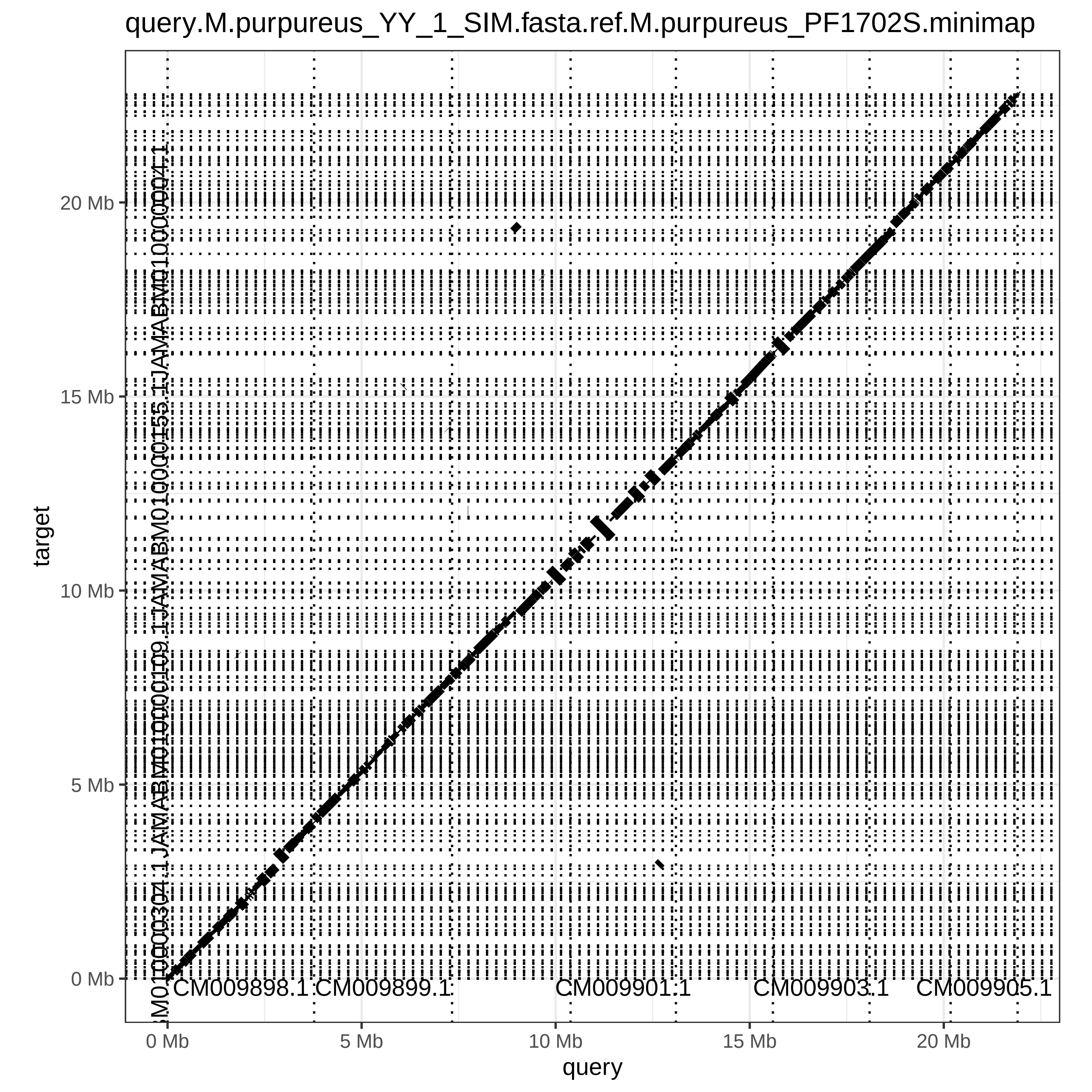
query.M.purpureus_YY_1_SIM.fasta.ref.M.purpureus_PF1702S.minimap
target
20 Mb
15 Mb
10 Mb
5 Mb
0 Mb
query
0 Mb
5 Mb
10 Mb
15 Mb
20 Mb
CM009898.1
CM009899.1
CM009901.1
CM009903.1
CM009905.1

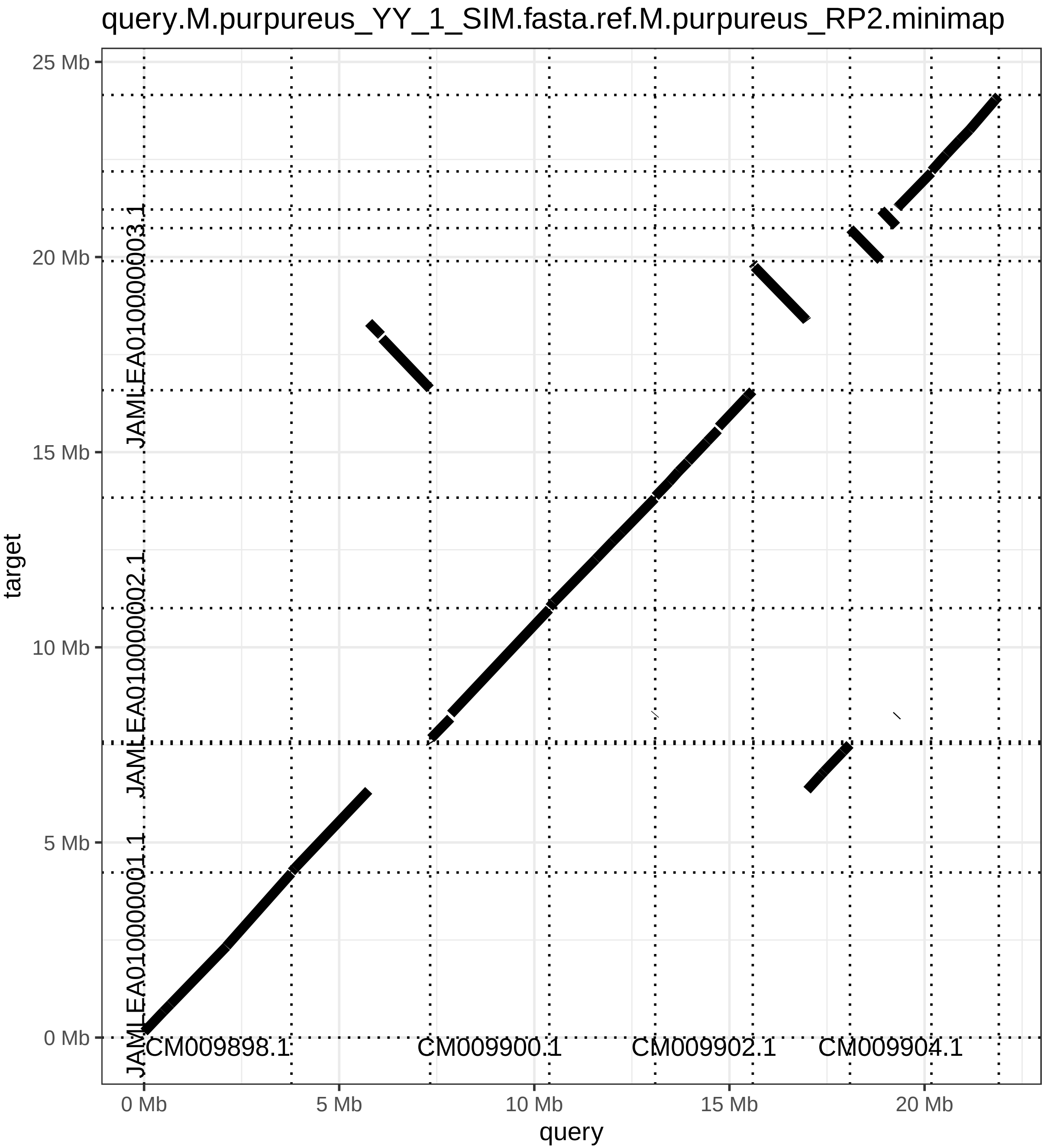

query.M.purpureus_YY_1_SIM.fasta.ref.M.purpureus_RP2.minimap
target
query
25 Mb
20 Mb
15 Mb
10 Mb
5 Mb
0 Mb
0 Mb
5 Mb
10 Mb
15 Mb
20 Mb
JAMLEA010000001.1
JAMLEA010000002.1
JAMLEA010000003.1
CM009898.1
CM009900.1
CM009902.1
CM009904.1

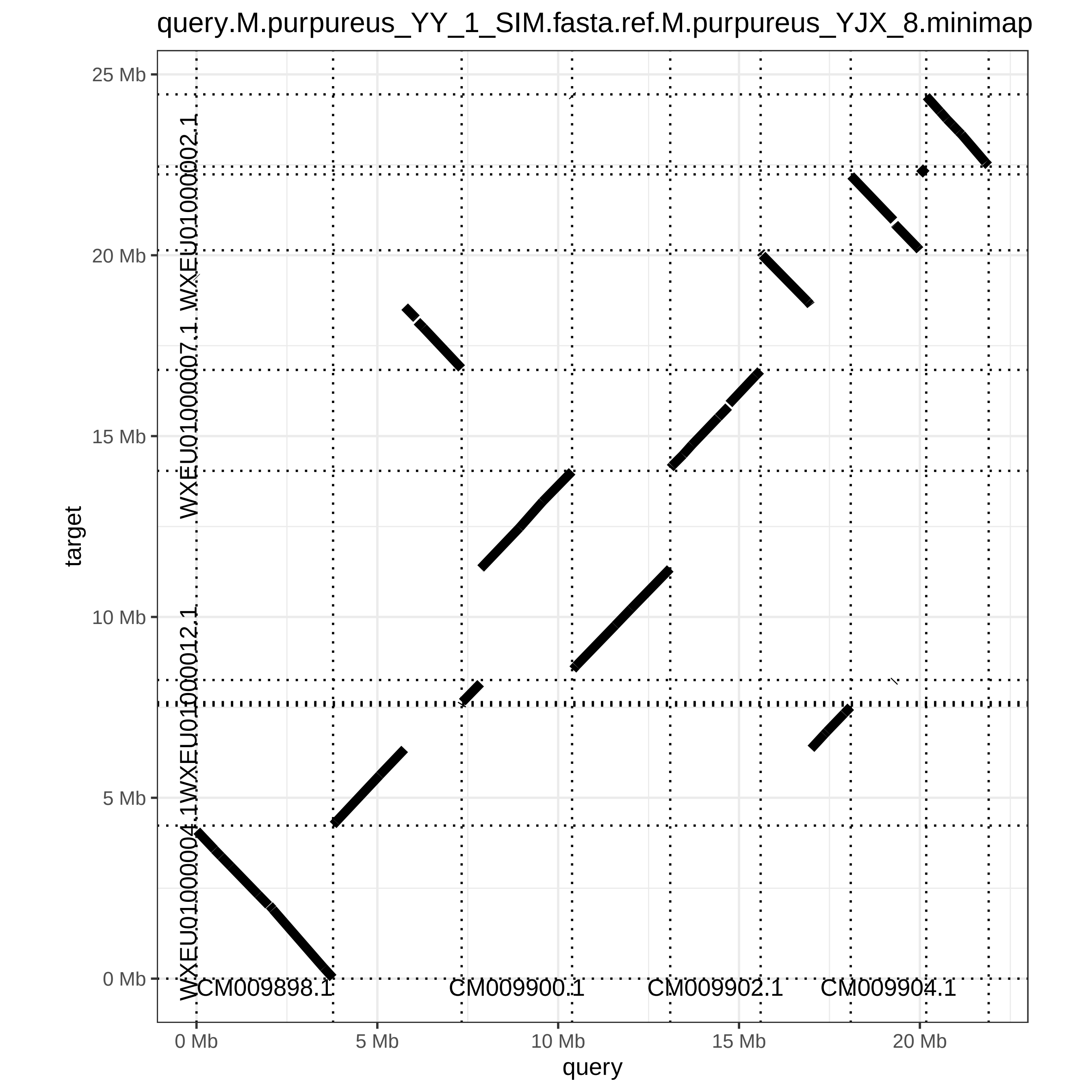
query.M.purpureus_YY_1_SIM.fasta.ref.M.purpureus_YJX_8.minimap
target
25 Mb
20 Mb
15 Mb
10 Mb
5 Mb
0 Mb
WXEU010000002.1
WXEU010000007.1 WXEU010000002.1
WXEU010000012.1
WXEU010000004.1WXEU010000012.1
CM009898.1
CM009900.1
CM009902.1
CM009904.1
0 Mb
5 Mb
10 Mb
15 Mb
20 Mb
query

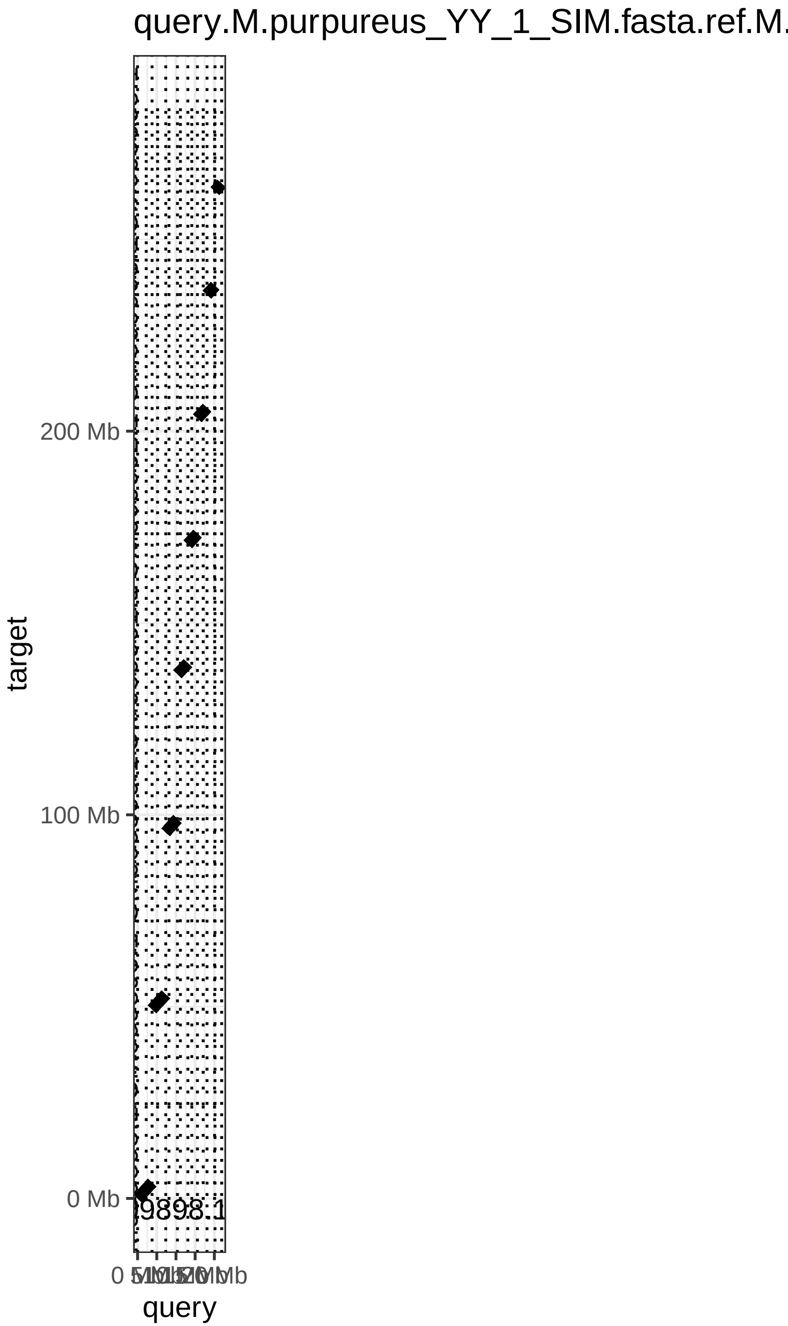

query.M.purpureus_YY_1_SIM.fasta.ref.M.
target
200 Mb
100 Mb
0 Mb
query

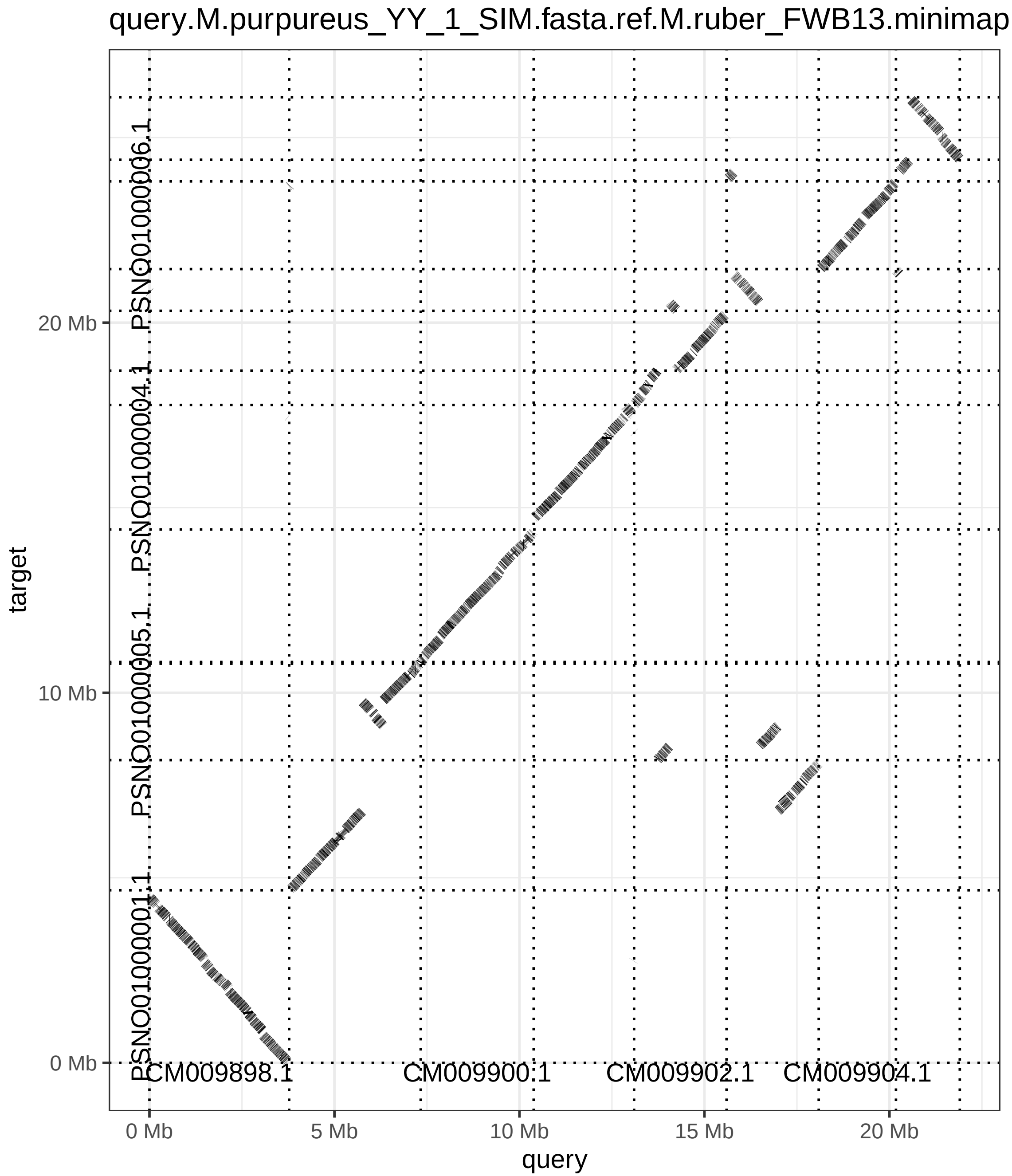
query.M.purpureus_YY_1_SIM.fasta.ref.M.ruber_FWB13.minimap
target
query
0 Mb
10 Mb
20 Mb
0 Mb
5 Mb
10 Mb
15 Mb
20 Mb
PSNO01000001.1
PSNO01000005.1
PSNO01000004.1
PSNO01000006.1
CM009898.1
CM009900.1
CM009902.1
CM009904.1

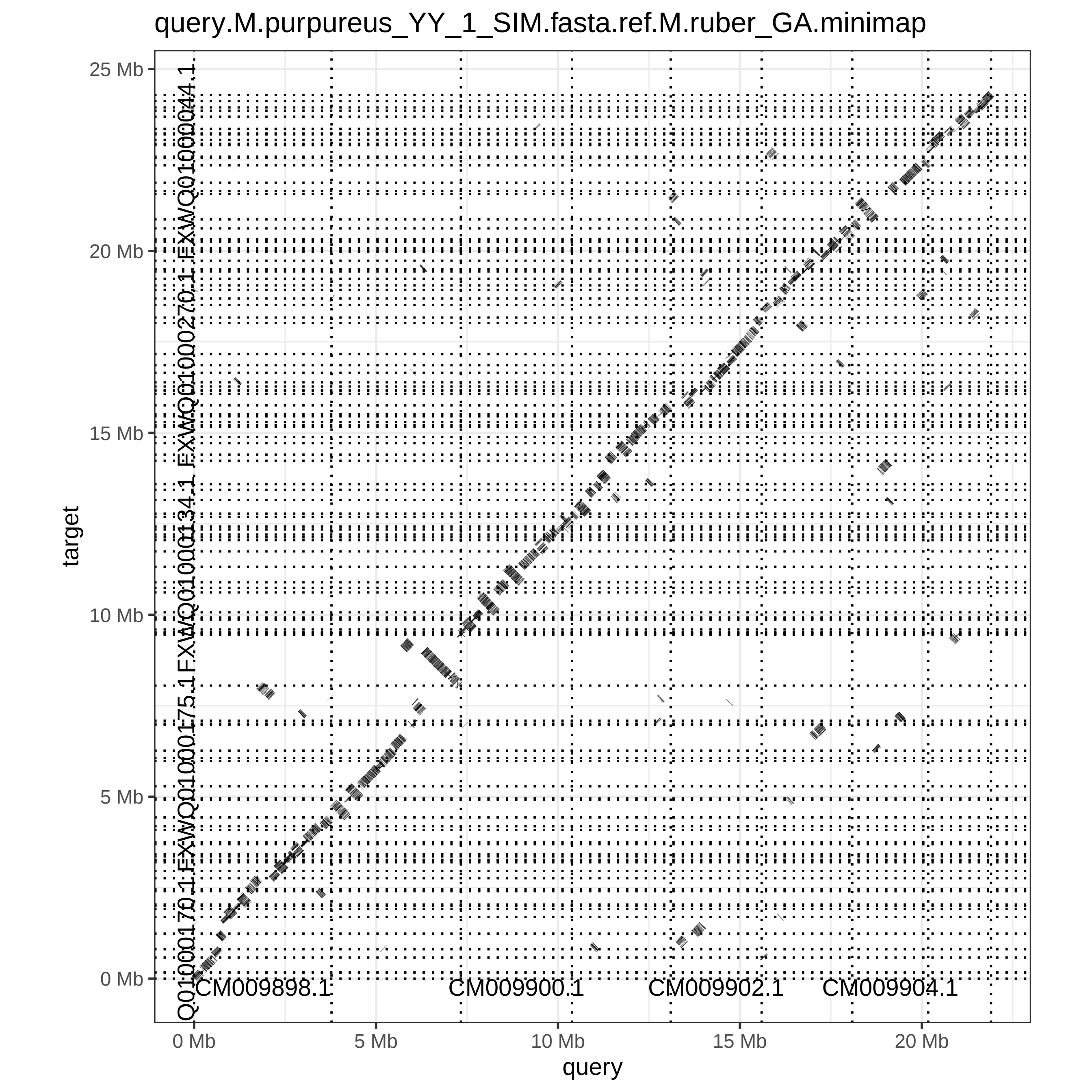
query.M.purpureus_YY_1_SIM.fasta.ref.M.ruber_GA.minimap
target
query
25 Mb
20 Mb
15 Mb
10 Mb
5 Mb
0 Mb
0 Mb
5 Mb
10 Mb
15 Mb
20 Mb
CM009898.1
CM009900.1
CM009902.1
CM009904.1

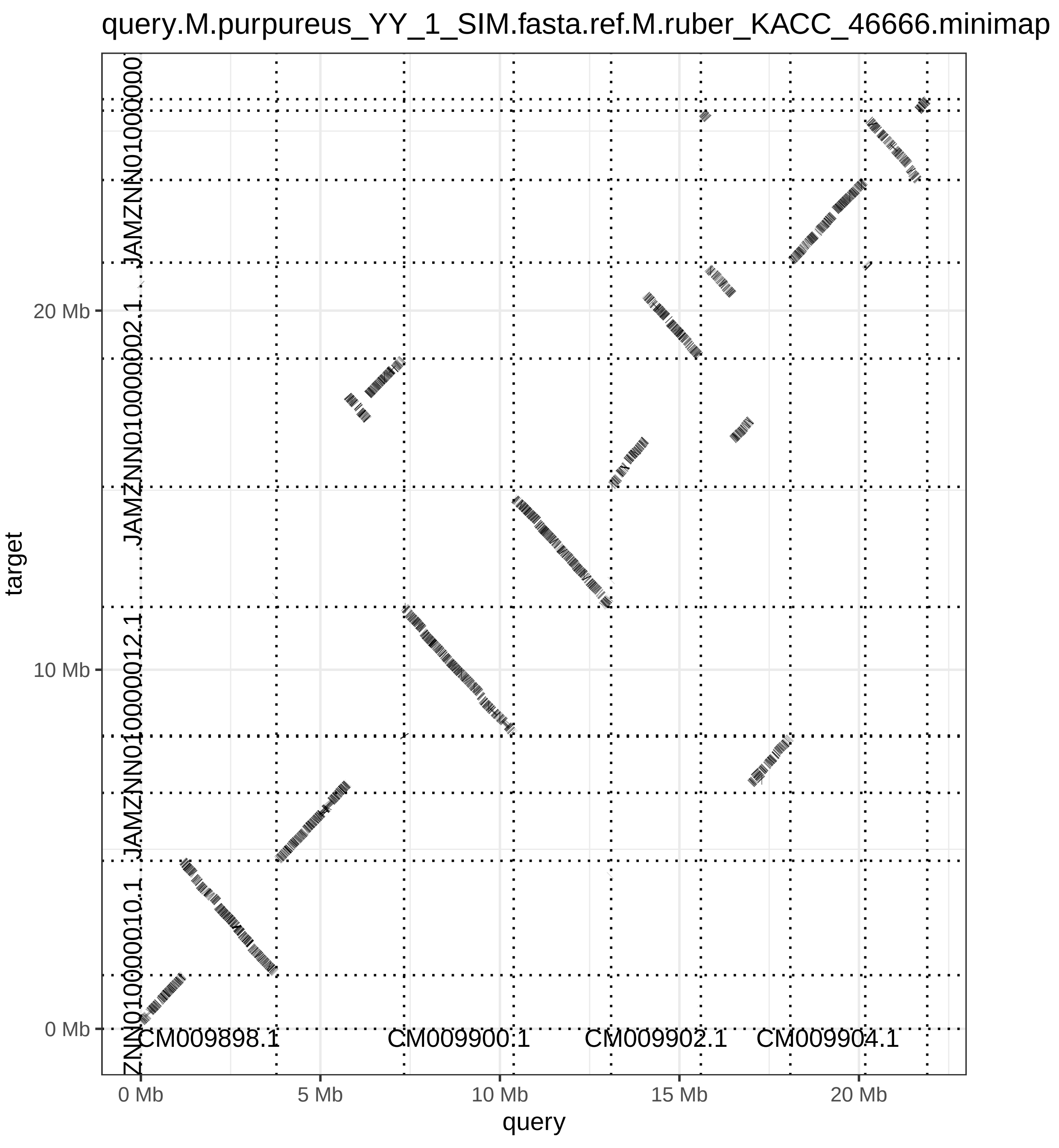
query.M.purpureus_YY_1_SIM.fasta.ref.M.ruber_KACC_46666.minimap
target
query
0 Mb
10 Mb
20 Mb
5 Mb
15 Mb
CM009898.1
CM009900.1
CM009902.1
CM009904.1
JAMZNN010000002.1
JAMZNN010000012.1
JAMZNN010000010.1

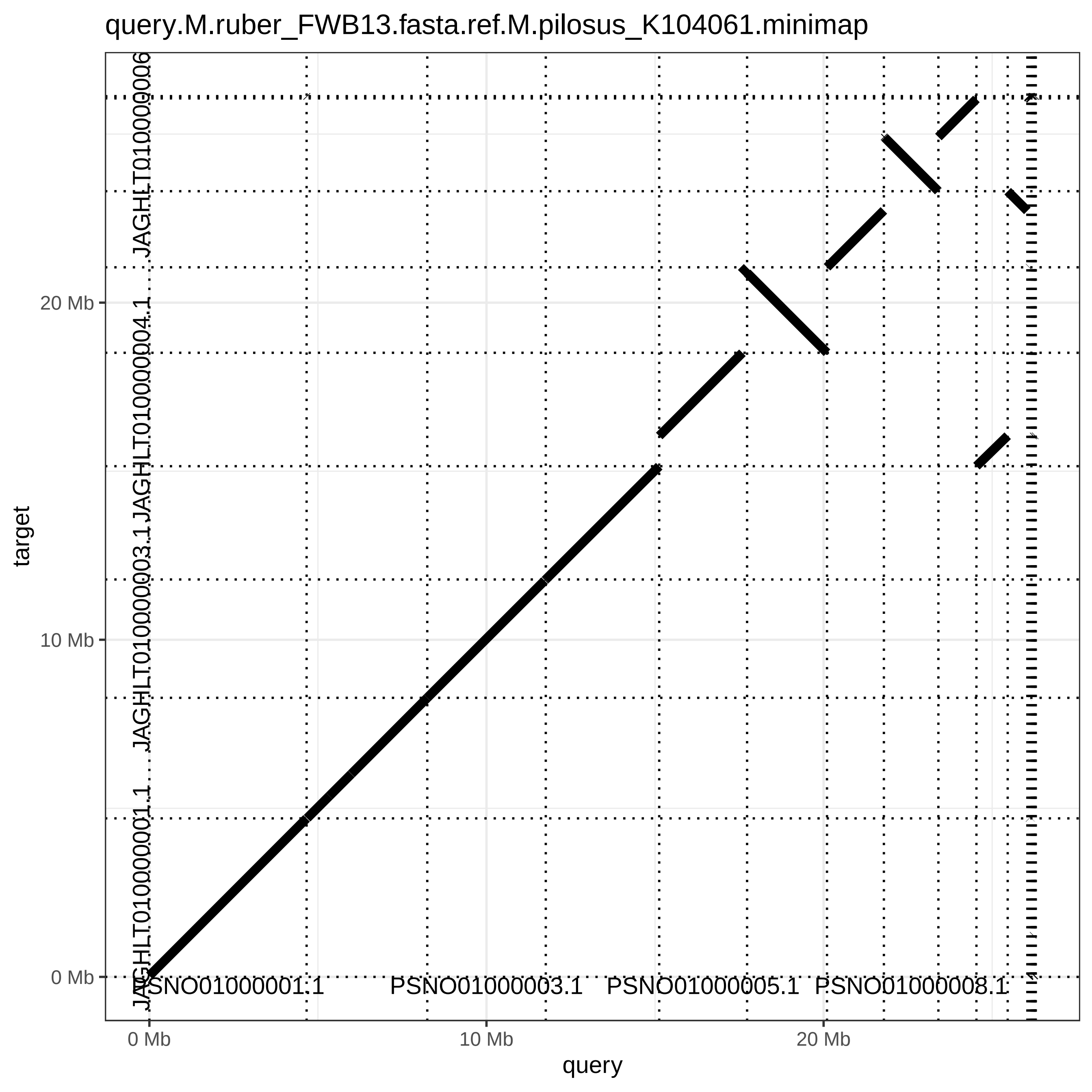
query.M.ruber_FWB13.fasta.ref.M.pilosus_K104061.minimap
target
query
0 Mb
10 Mb
20 Mb
PSNO01000001.1
PSNO01000003.1
PSNO01000005.1
PSNO01000008.1
JAGHLT010000001.1
JAGHLT010000003.1
JAGHLT010000004.1
JAGHLT010000006

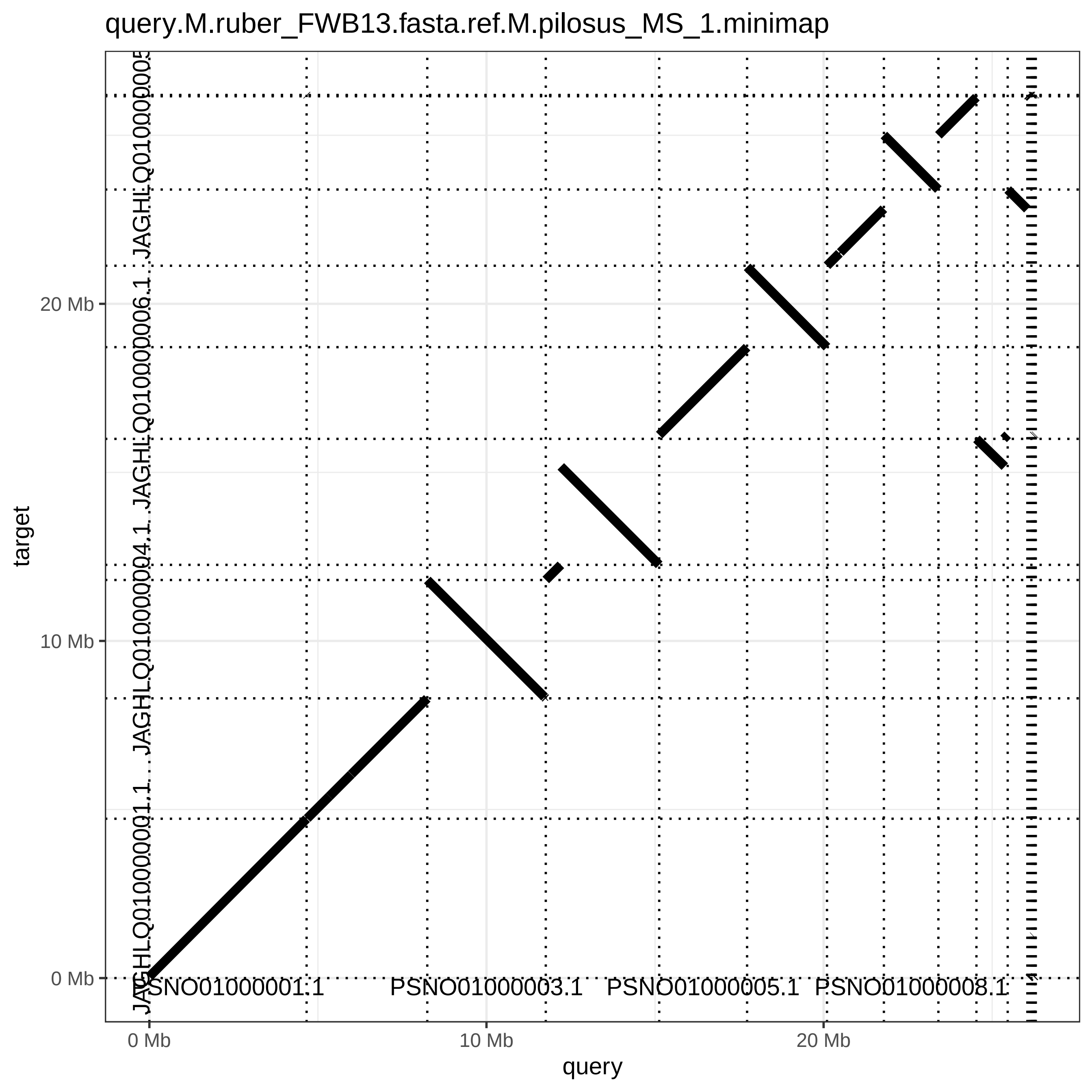

query.M.ruber_FWB13.fasta.ref.M.pilosus_MS_1.minimap
target
query
0 Mb
10 Mb
20 Mb
PSNO01000001.1
PSNO01000003.1
PSNO01000005.1
PSNO01000008.1
JAGHLQ010000001.1
JAGHLQ010000004.1
JAGHLQ010000006.1
JAGHLQ01000005

query.M.ruber_FWB13.fasta.ref.M.pilosus_YDJ_1.minimap

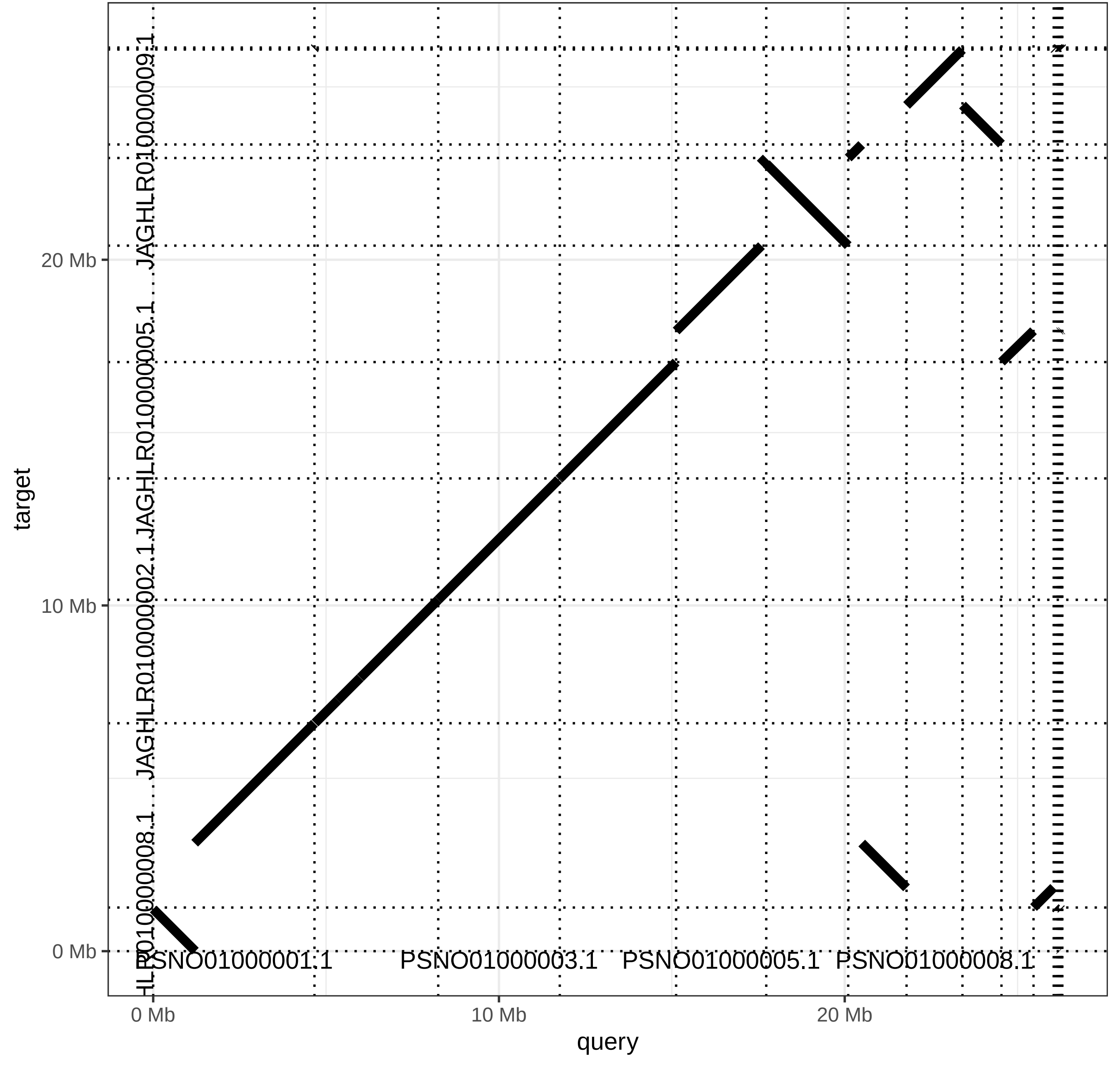

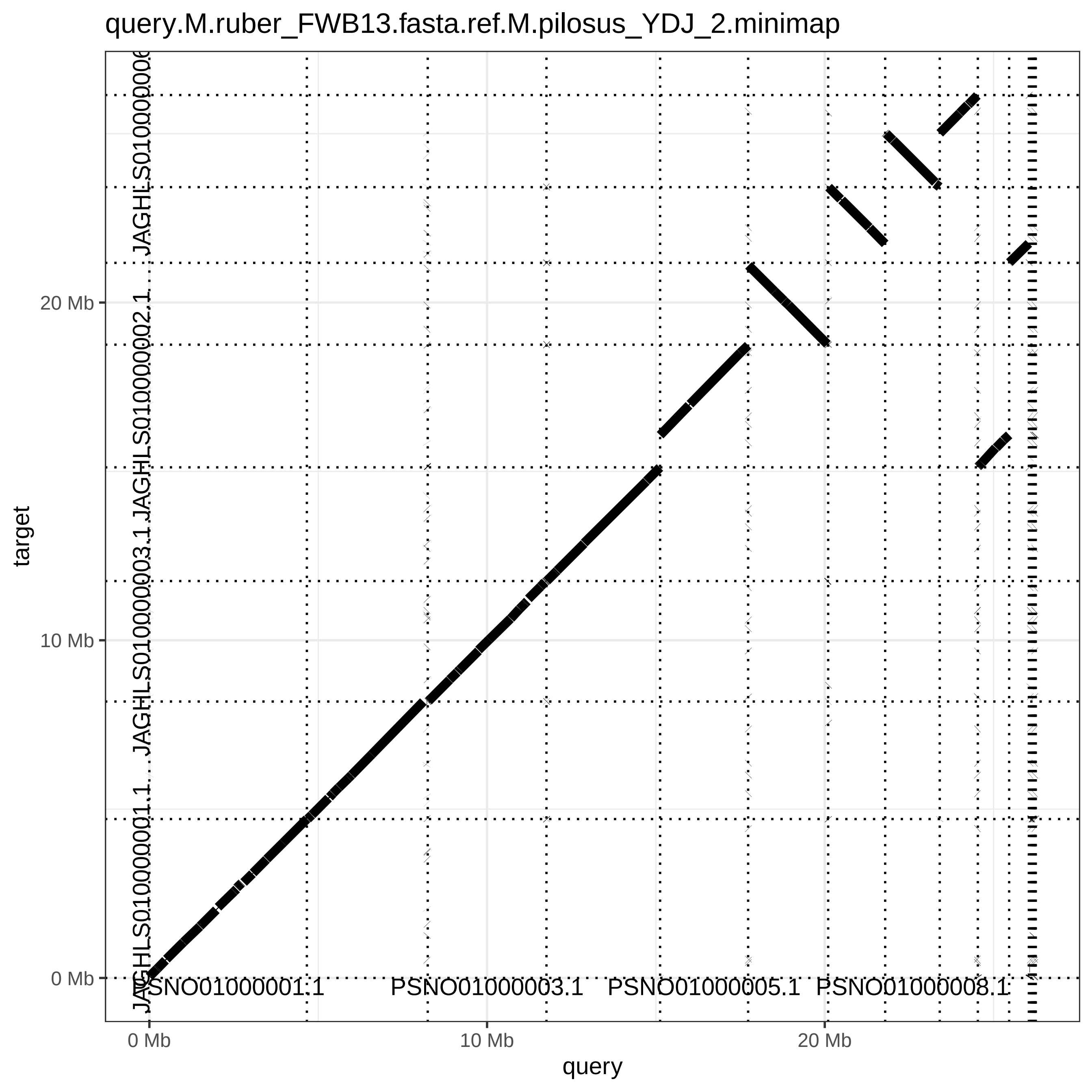

query.M.ruber_FWB13.fasta.ref.M.pilosus_YDJ_2.minimap
target
query
0 Mb
10 Mb
20 Mb
PSNO01000001.1
PSNO01000003.1
PSNO01000005.1
PSNO01000008.1
JAGHLS010000001.1
JAGHLS010000003.1
JAGHLS010000002.1

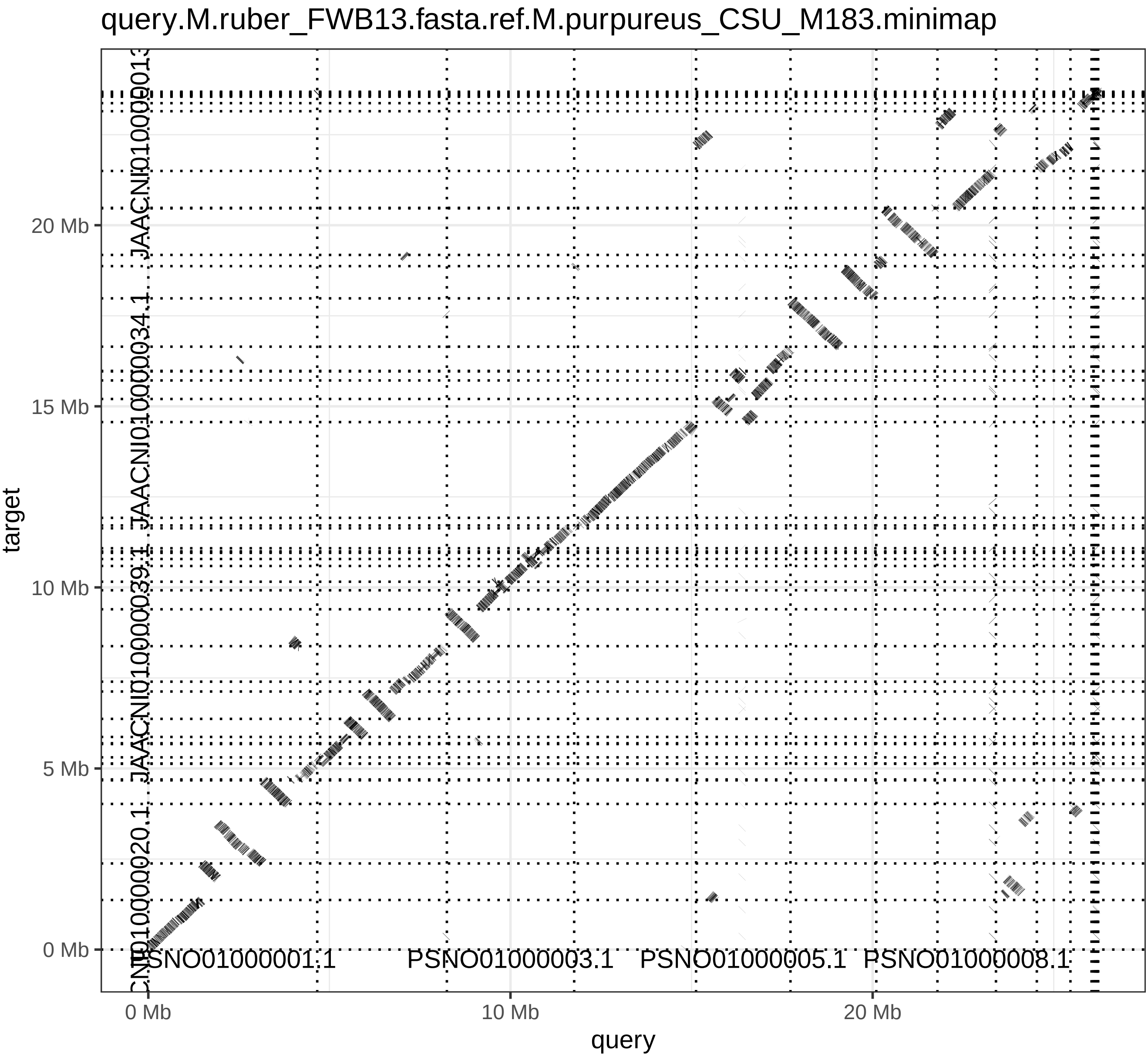
query.M.ruber_FWB13.fasta.ref.M.purpureus_CSU_M183.minimap
target
query
0 Mb
10 Mb
20 Mb
5 Mb
15 Mb
PSNO01000001.1
PSNO01000003.1
PSNO01000005.1
PSNO01000008.1
JAACNI010000020.1
JAACNI010000039.1
JAACNI010000034.1

query.M.ruber_FWB13.fasta.ref.M.purpureus_GB_01.minimap

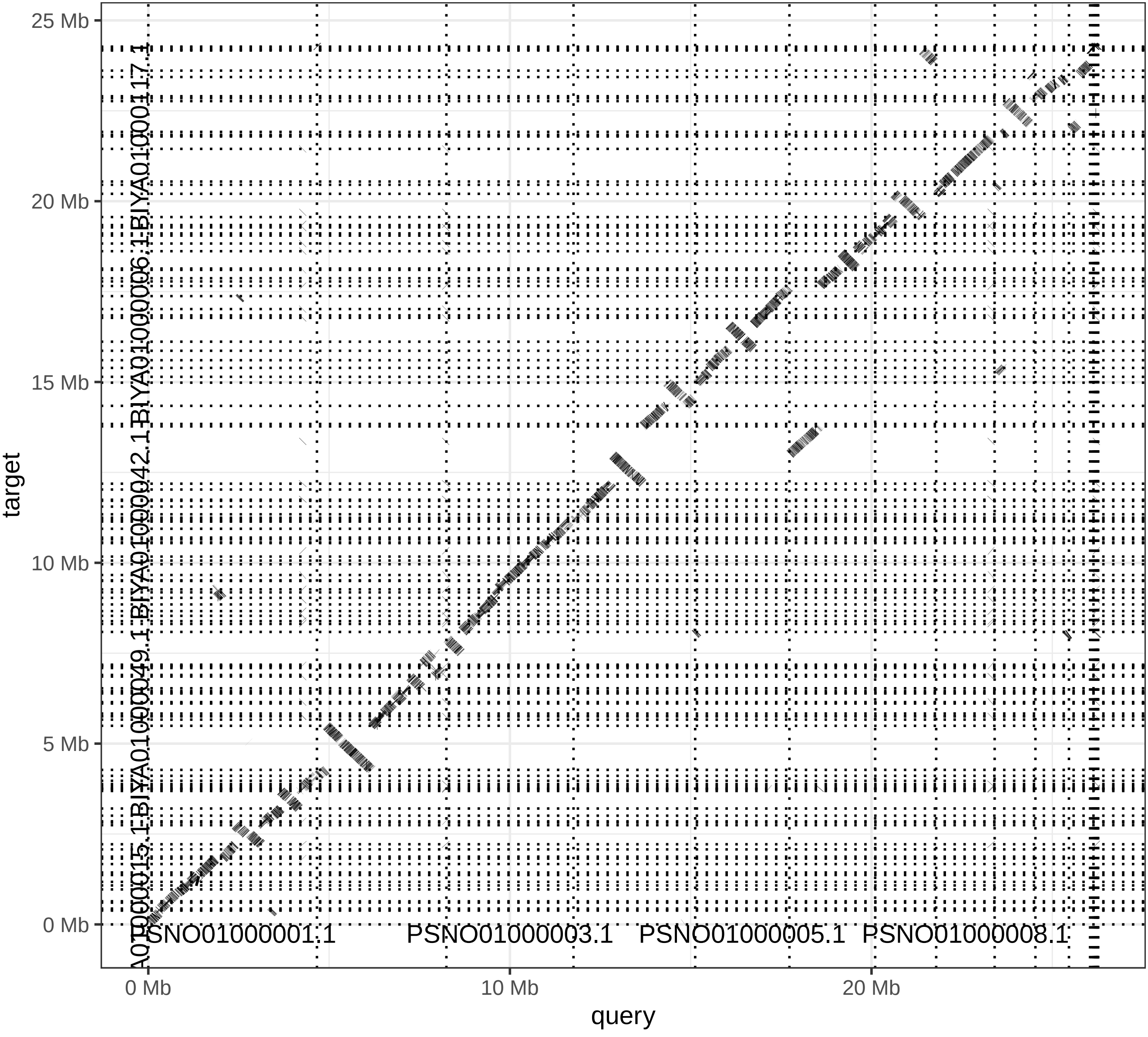

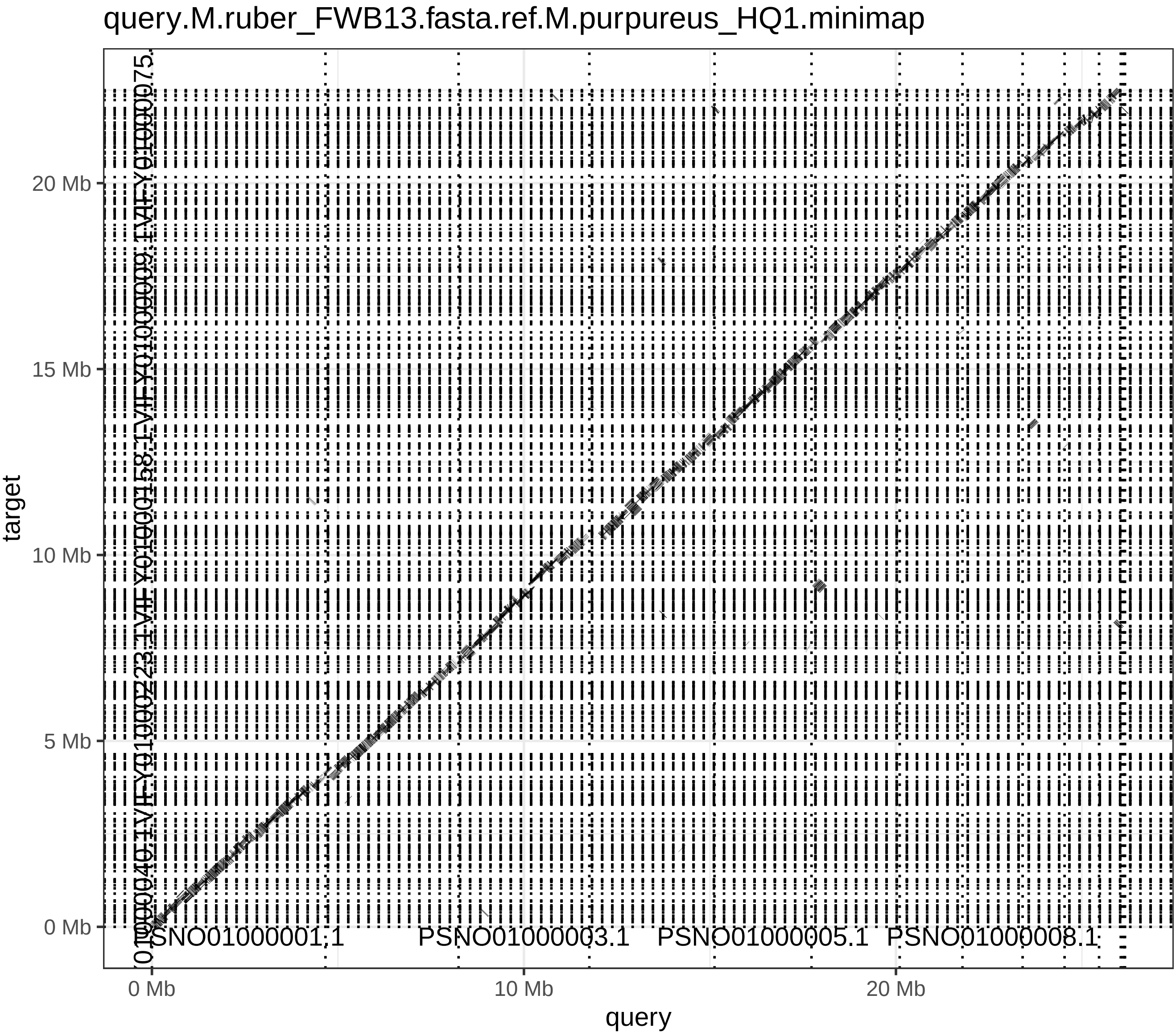
query.M.ruber_FWB13.fasta.ref.M.purpureus_HQ1.minimap
target
20 Mb
15 Mb
10 Mb
5 Mb
0 Mb
PSNO01000001.1
PSNO01000003.1
PSNO01000005.1
PSNO01000008.1
0 Mb
10 Mb
20 Mb
query

query.M.ruber_FWB13.fasta.ref.M.purpureus_P7048X_2.minimap

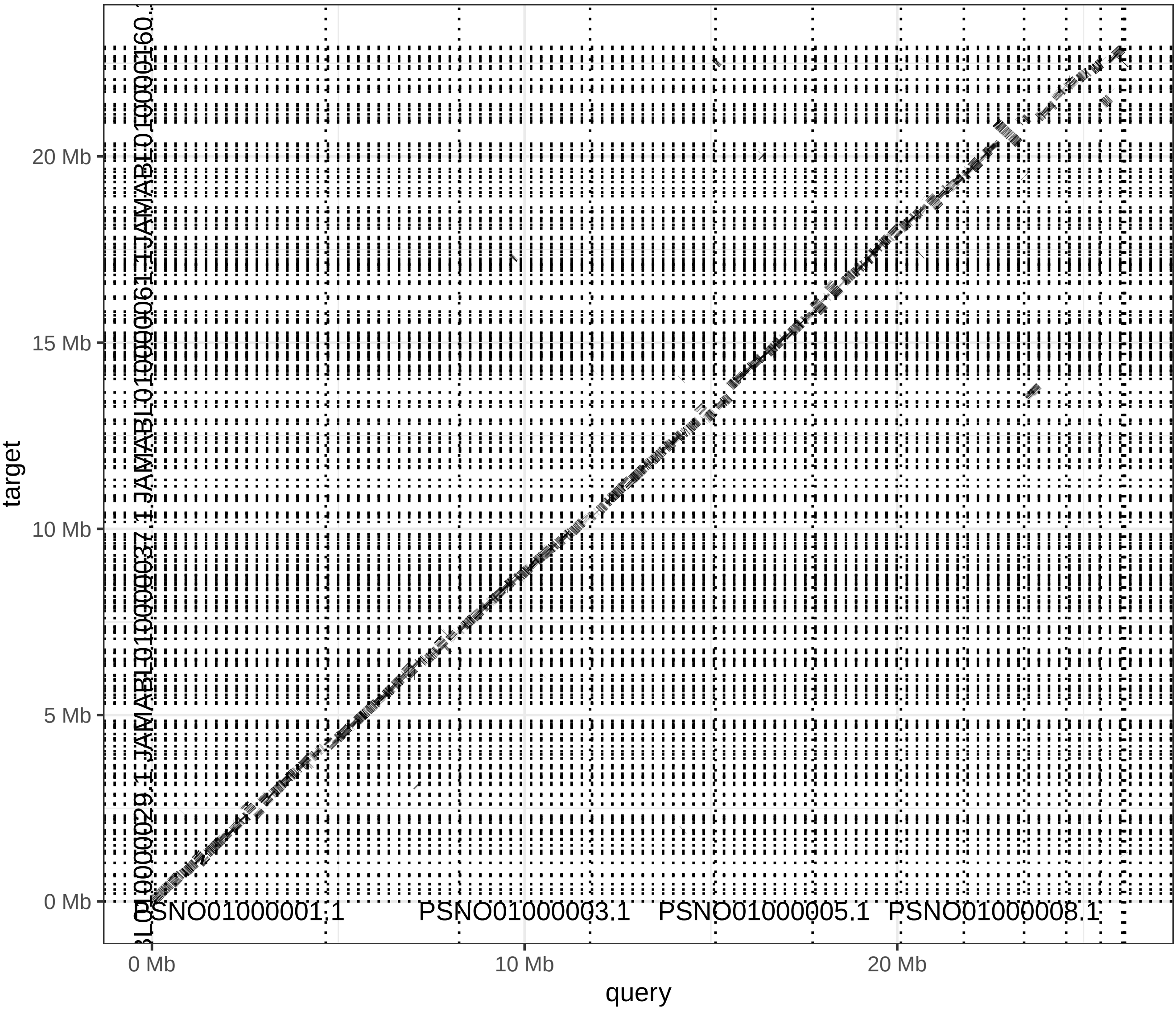

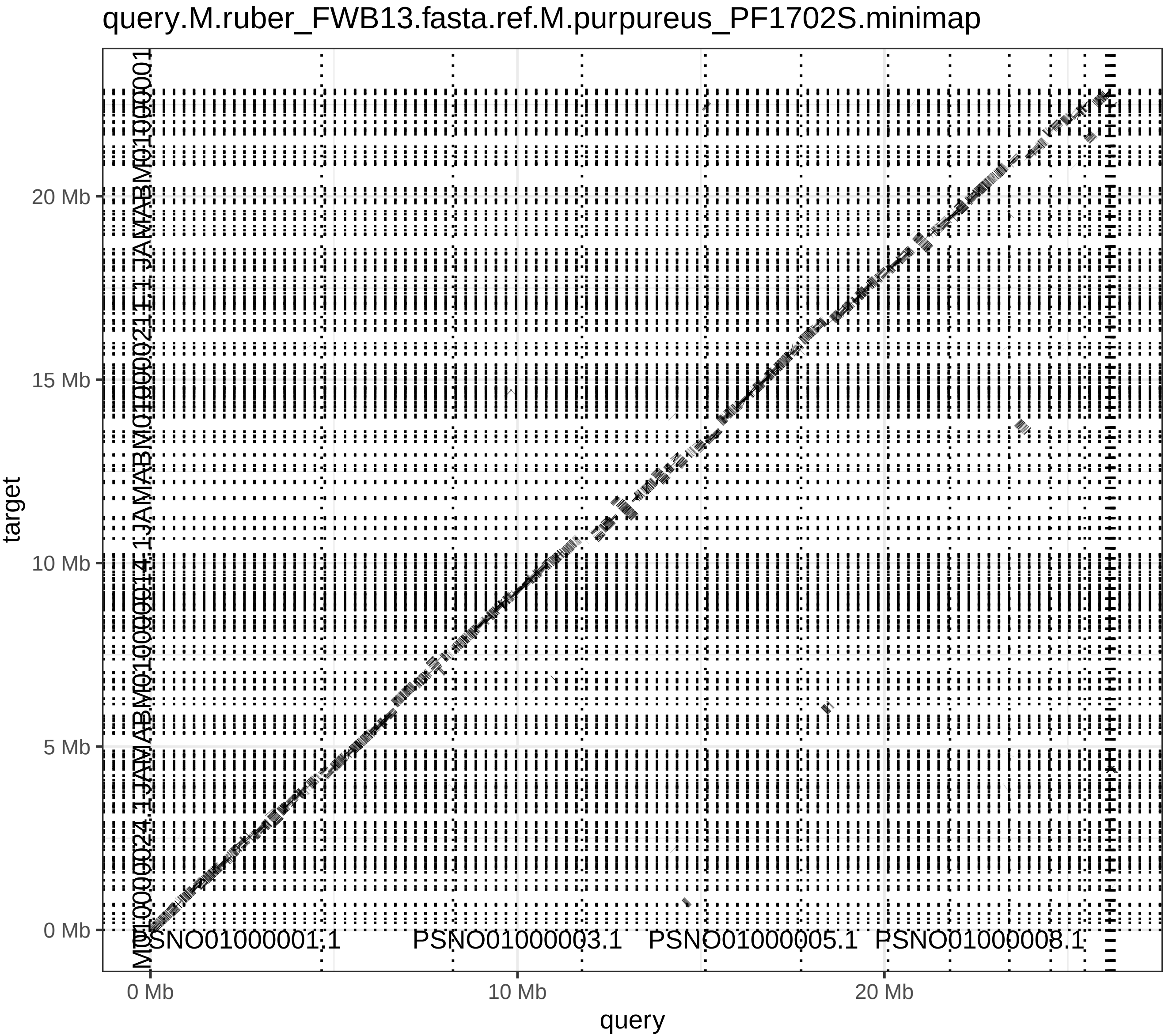

query.M.ruber_FWB13.fasta.ref.M.purpureus_PF1702S.minimap
target
query
0 Mb
5 Mb
10 Mb
15 Mb
20 Mb
0 Mb
10 Mb
20 Mb
PSNO01000001.1
PSNO01000003.1
PSNO01000005.1
PSNO01000008.1

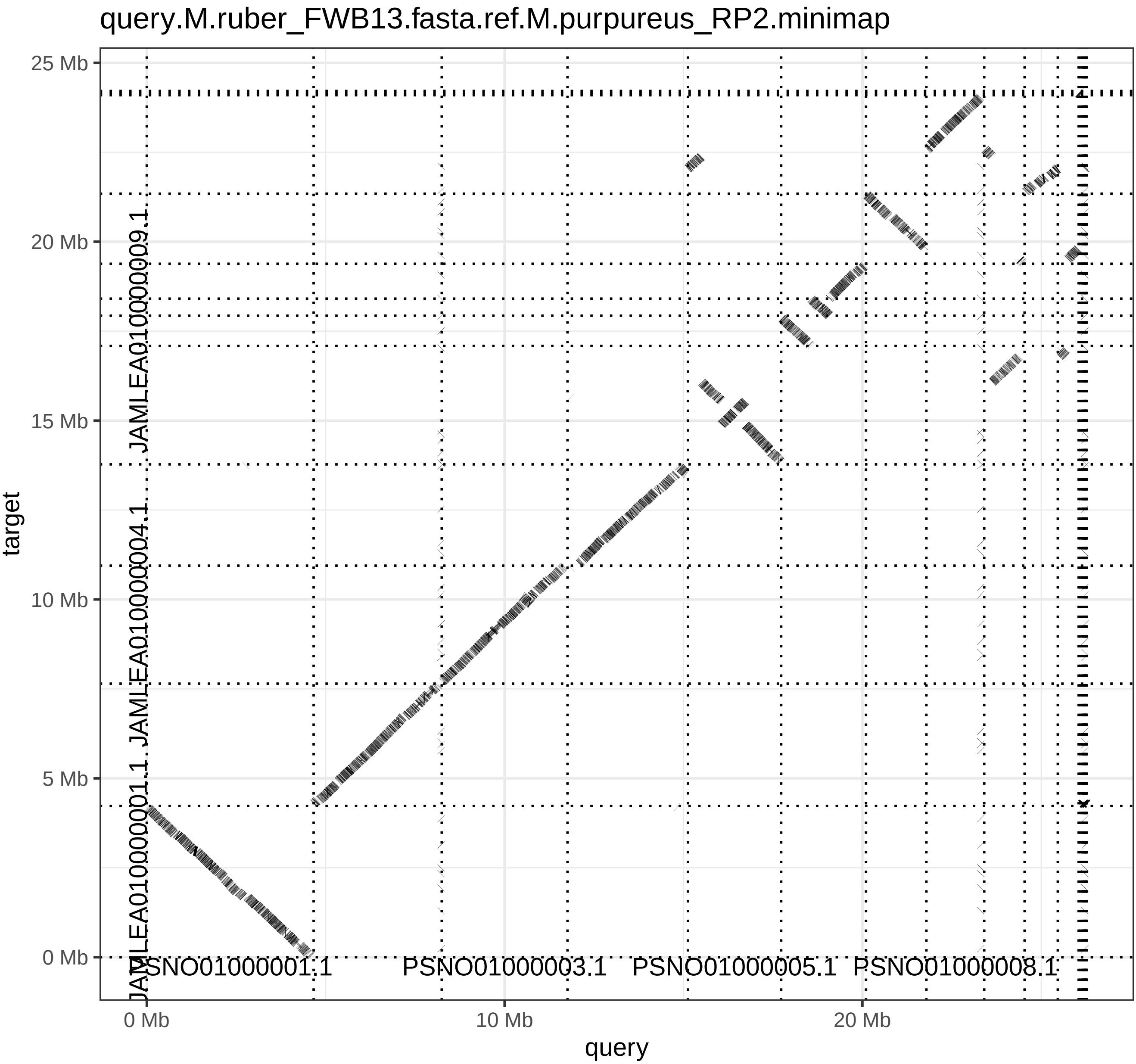
query.M.ruber_FWB13.fasta.ref.M.purpureus_RP2.minimap
25 Mb
20 Mb
15 Mb
10 Mb
5 Mb
0 Mb
target
JAMLEA010000009.1
JAMLEA010000004.1
JAMLEA010000001.1 JAMLEA010000001.1
PSNO01000001.1
PSNO01000003.1
PSNO01000005.1
PSNO01000008.1
0 Mb
10 Mb
20 Mb
query

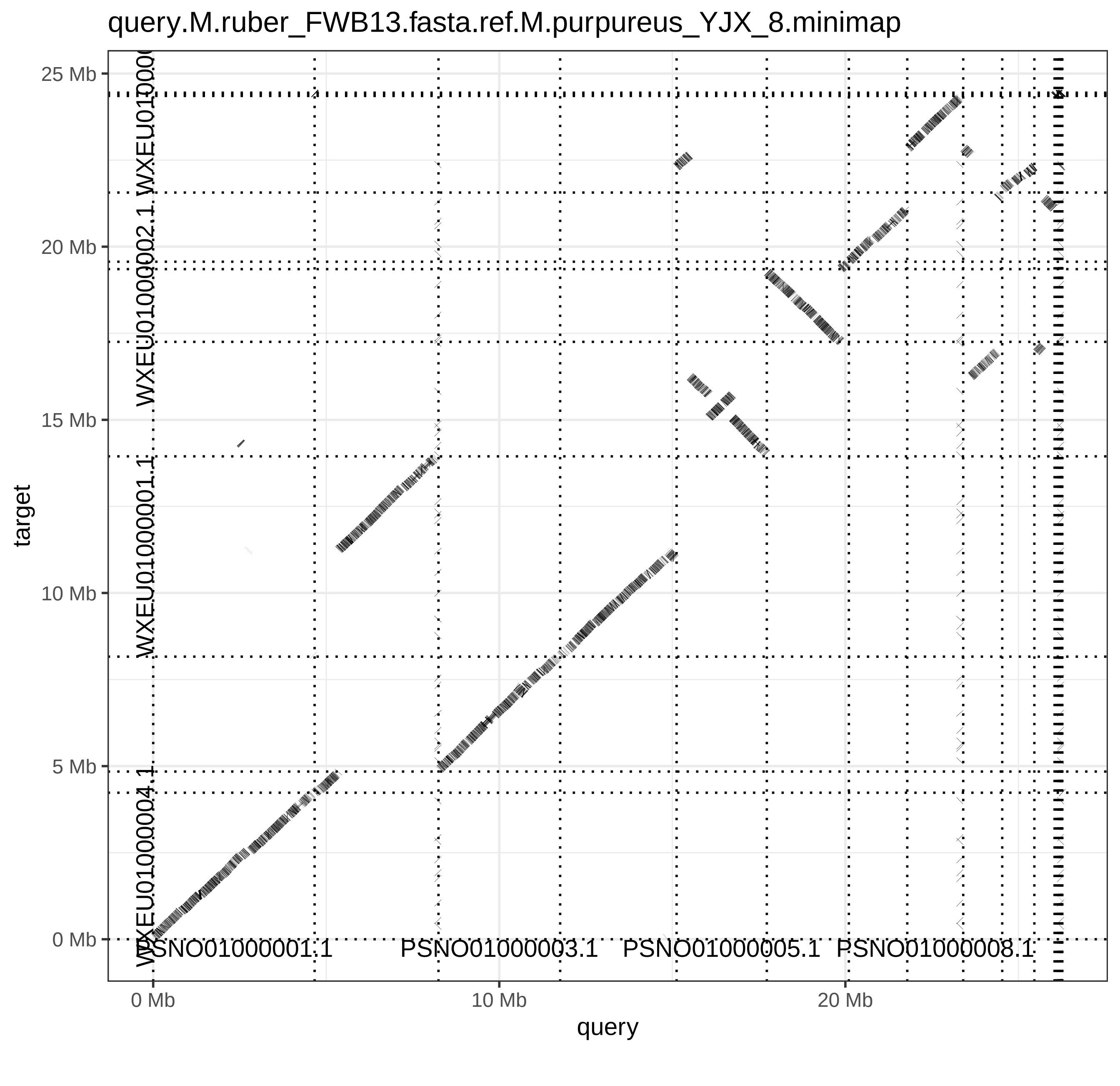

query.M.ruber_FWB13.fasta.ref.M.purpureus_YJX_8.minimap
target
query
25 Mb
20 Mb
15 Mb
10 Mb
5 Mb
0 Mb
0 Mb
10 Mb
20 Mb
WXEU01000002.1
WXEU01000001.1
WXEU01000004.1
PSNO01000001.1
PSNO01000003.1
PSNO01000005.1
PSNO01000008.1

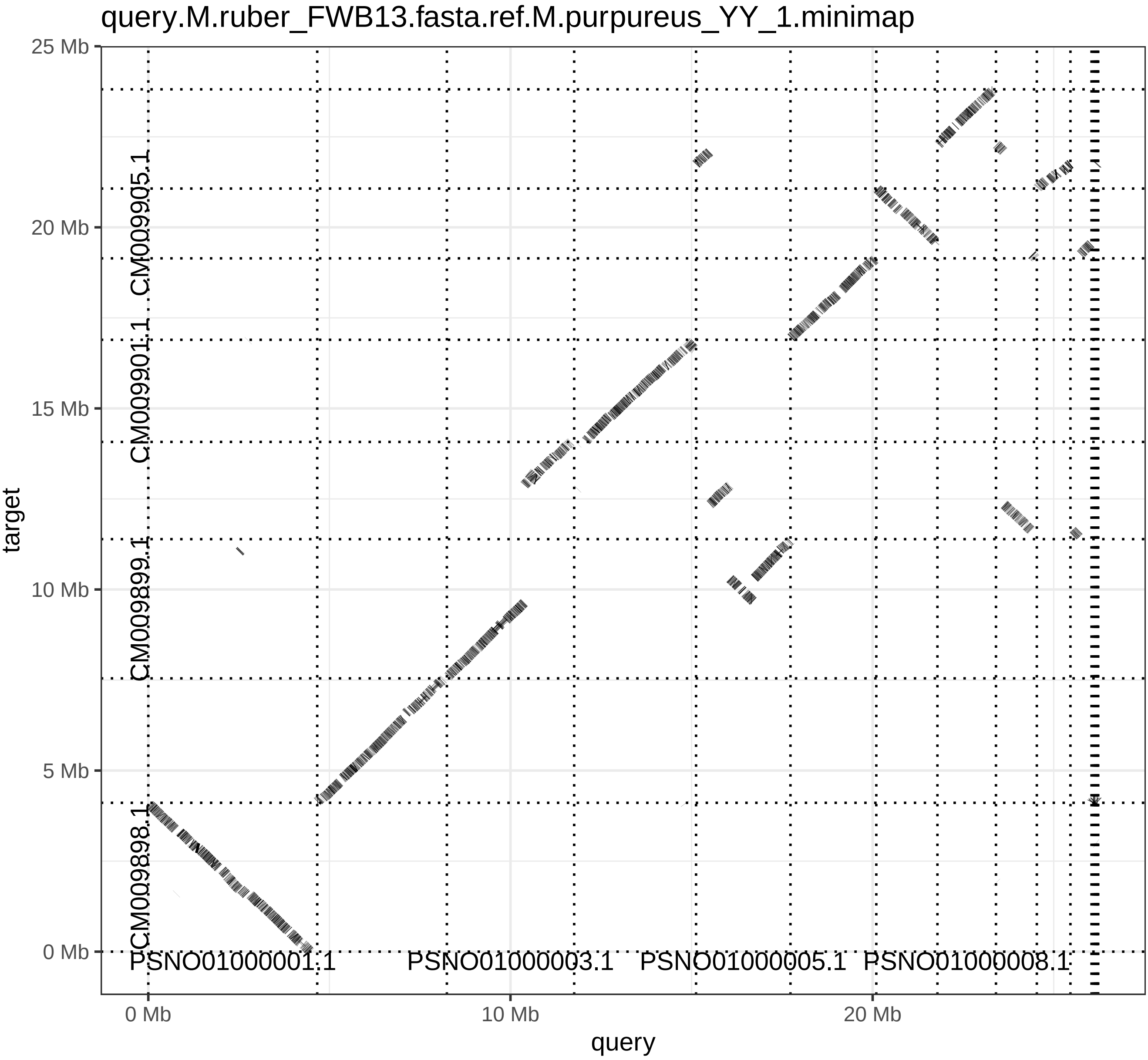

query.M.ruber_FWB13.fasta.ref.M.purpureus_YY_1.minimap
25 Mb
20 Mb
15 Mb
10 Mb
5 Mb
0 Mb
target
CM009905.1
CM009901.1
CM009899.1
CM009898.1
PSNO01000001.1
PSNO01000003.1
PSNO01000005.1
PSNO01000008.1
0 Mb
10 Mb
20 Mb
query

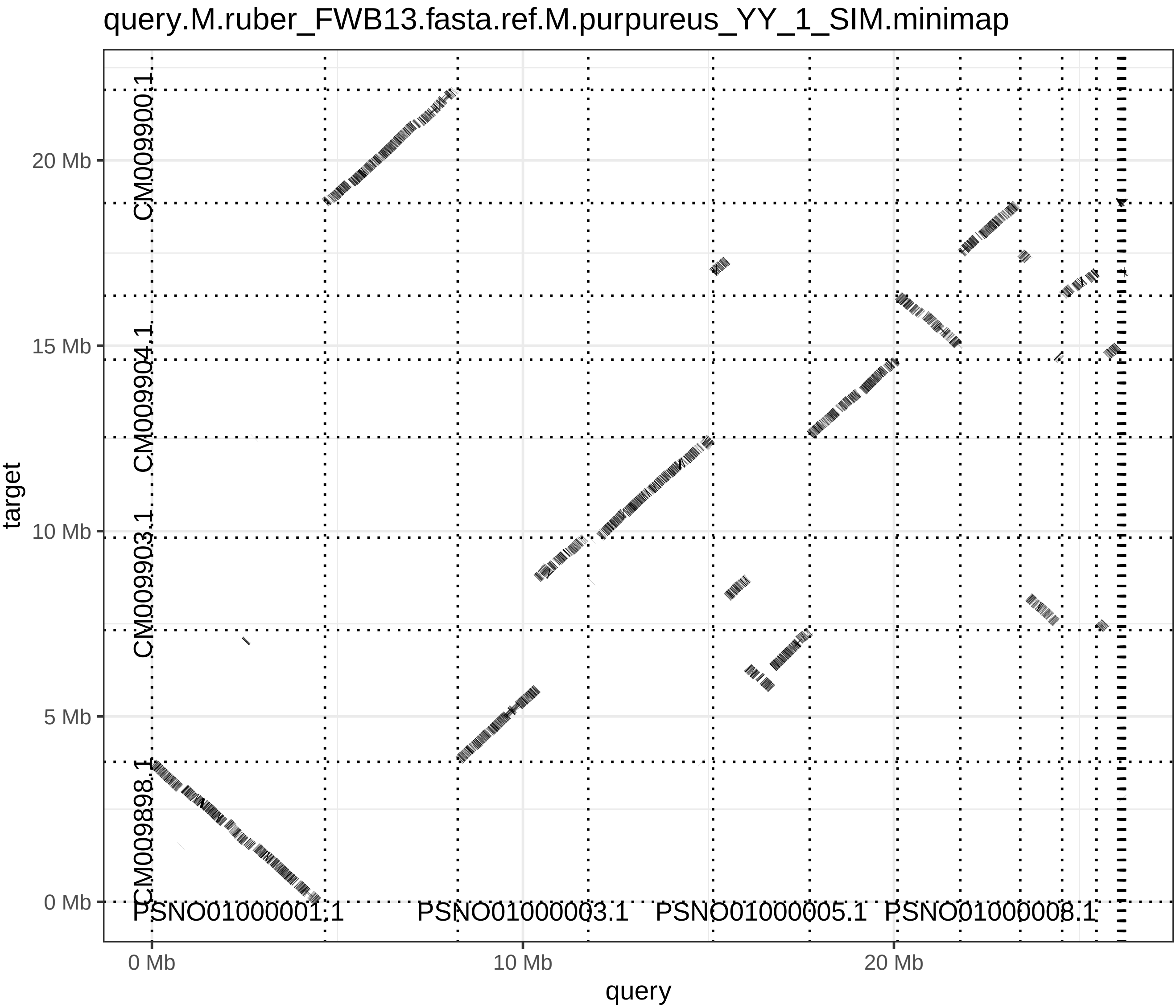
query.M.ruber_FWB13.fasta.ref.M.purpureus_YY_1_SIM.minimap
target
query
20 Mb
15 Mb
10 Mb
5 Mb
0 Mb
0 Mb
10 Mb
20 Mb
CM009900.1
CM009904.1
CM009903.1
CM009898.1
PSNO01000001.1
PSNO01000003.1
PSNO01000005.1
PSNO01000008.1

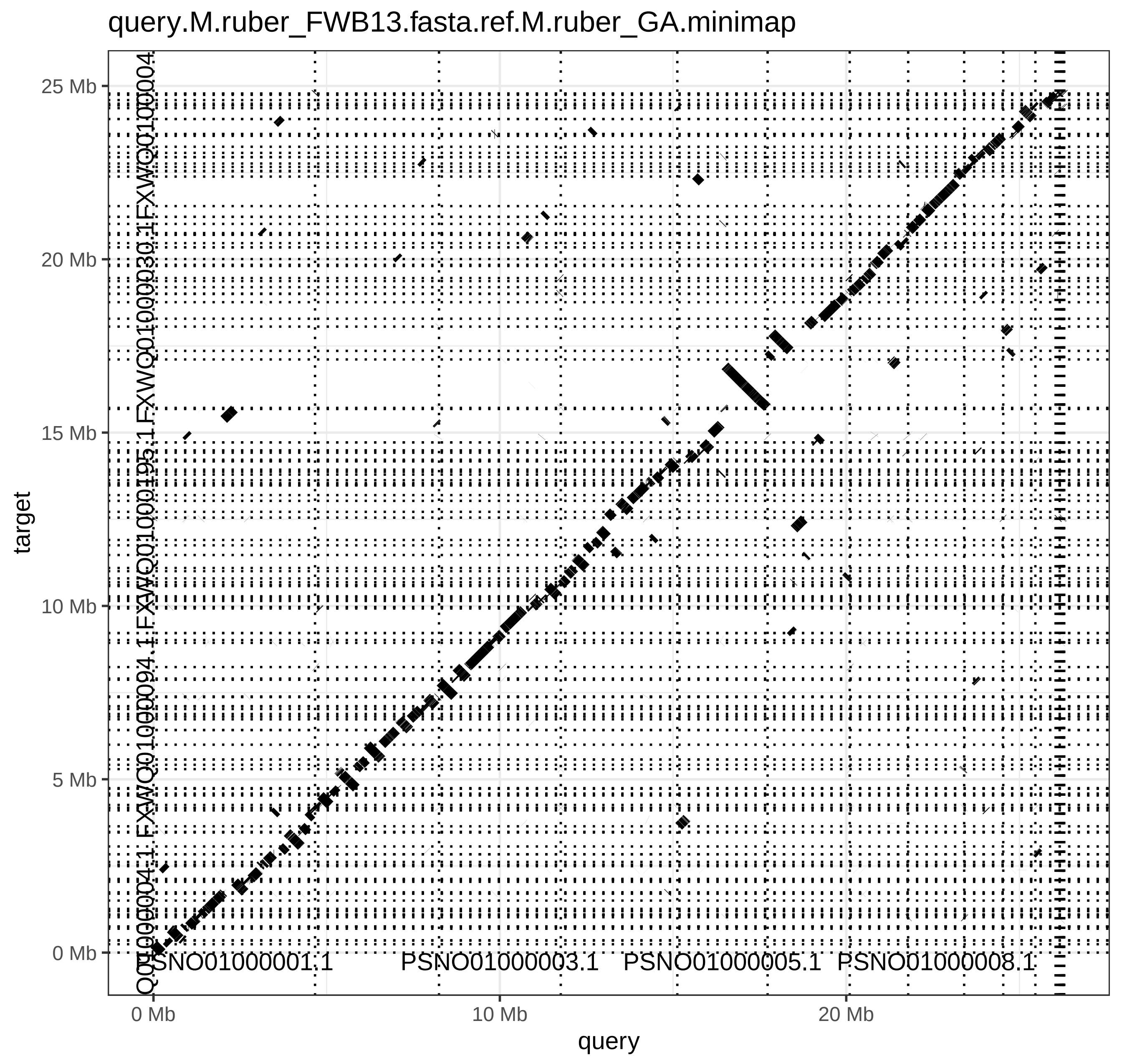

query.M.ruber_FWB13.fasta.ref.M.ruber_GA.minimap
25 Mb
20 Mb
15 Mb
10 Mb
5 Mb
0 Mb
target
0 Mb
10 Mb
20 Mb
query
PSNO01000001.1
PSNO01000003.1
PSNO01000005.1
PSNO01000008.1

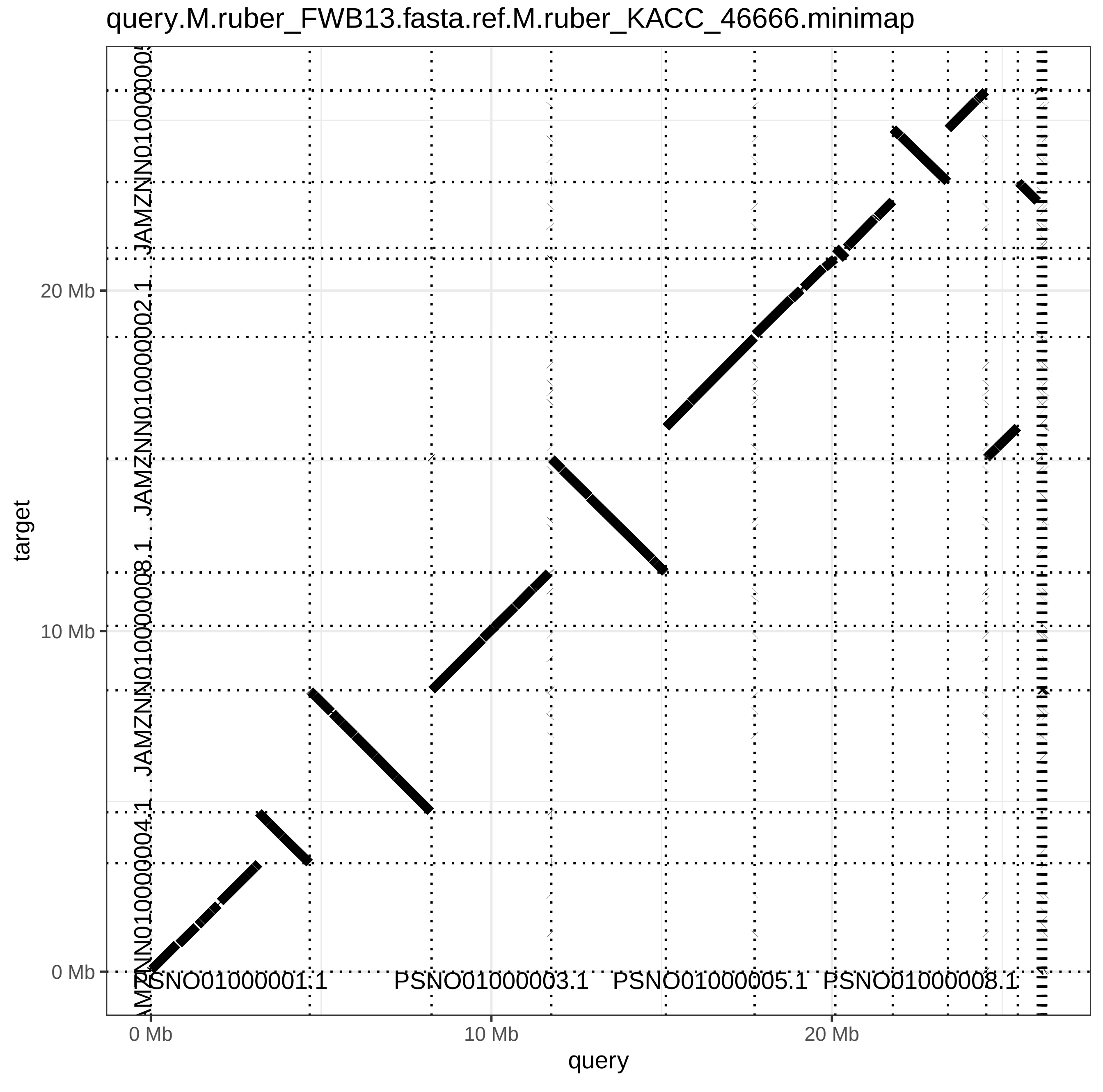
query.M.ruber_FWB13.fasta.ref.M.ruber_KACC_46666.minimap
target
query
20 Mb
10 Mb
0 Mb
0 Mb
10 Mb
20 Mb
PSNO01000001.1
PSNO01000003.1
PSNO01000005.1
PSNO01000008.1
JAMZNN010000004.1
JAMZNN010000008.1
JAMZNN010000002.1

query.M.ruber_GA.fasta.ref.M.pilosus_K104061.minimap

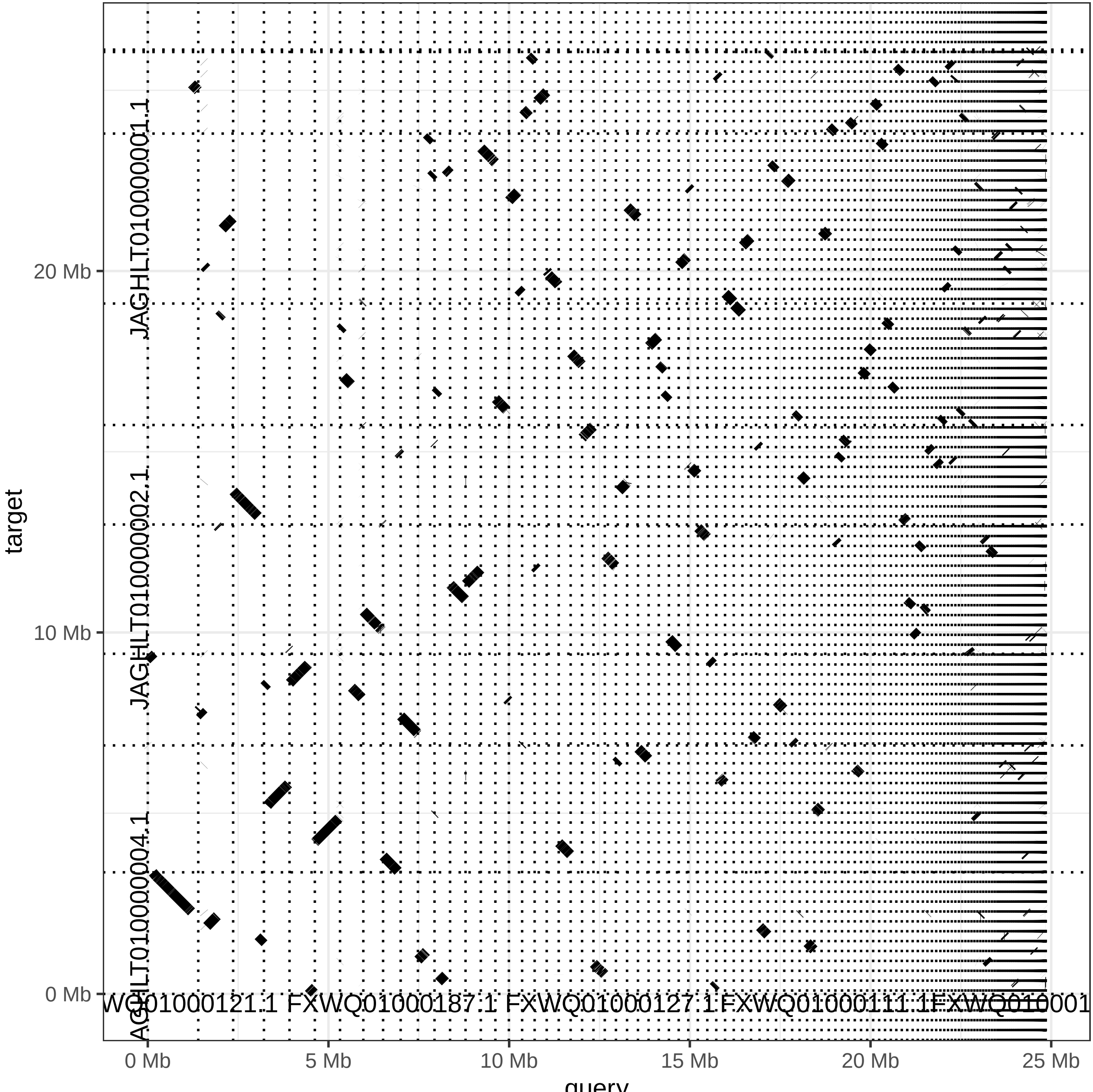

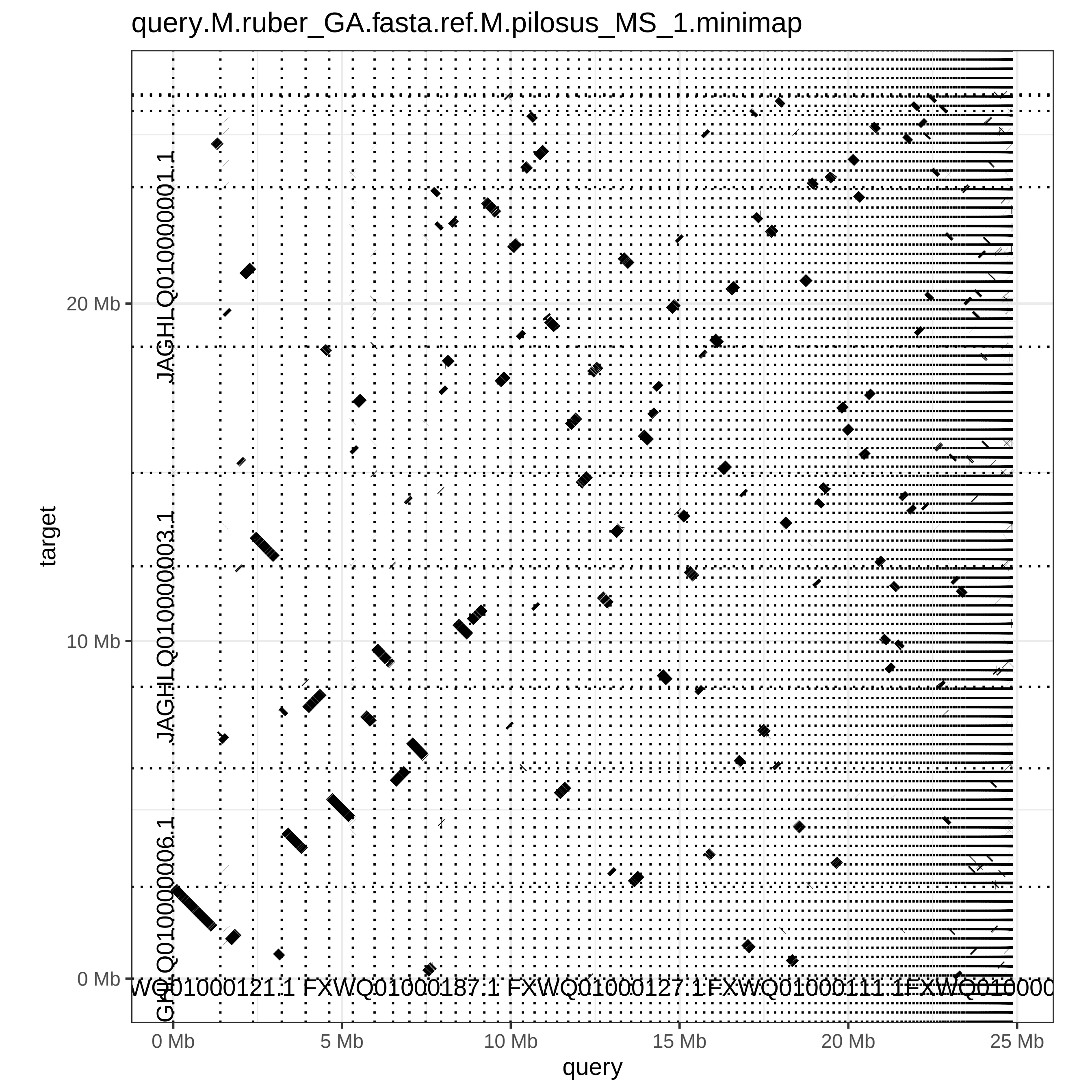

query.M.ruber_GA.fasta.ref.M.pilosus_MS_1.minimap
target
query
20 Mb
10 Mb
0 Mb
0 Mb
5 Mb
10 Mb
15 Mb
20 Mb
25 Mb
JAGHLQ010000001.1
JAGHLQ010000003.1
FXWQ01000187.1
FXWQ01000127.1
FXWQ01000111.1

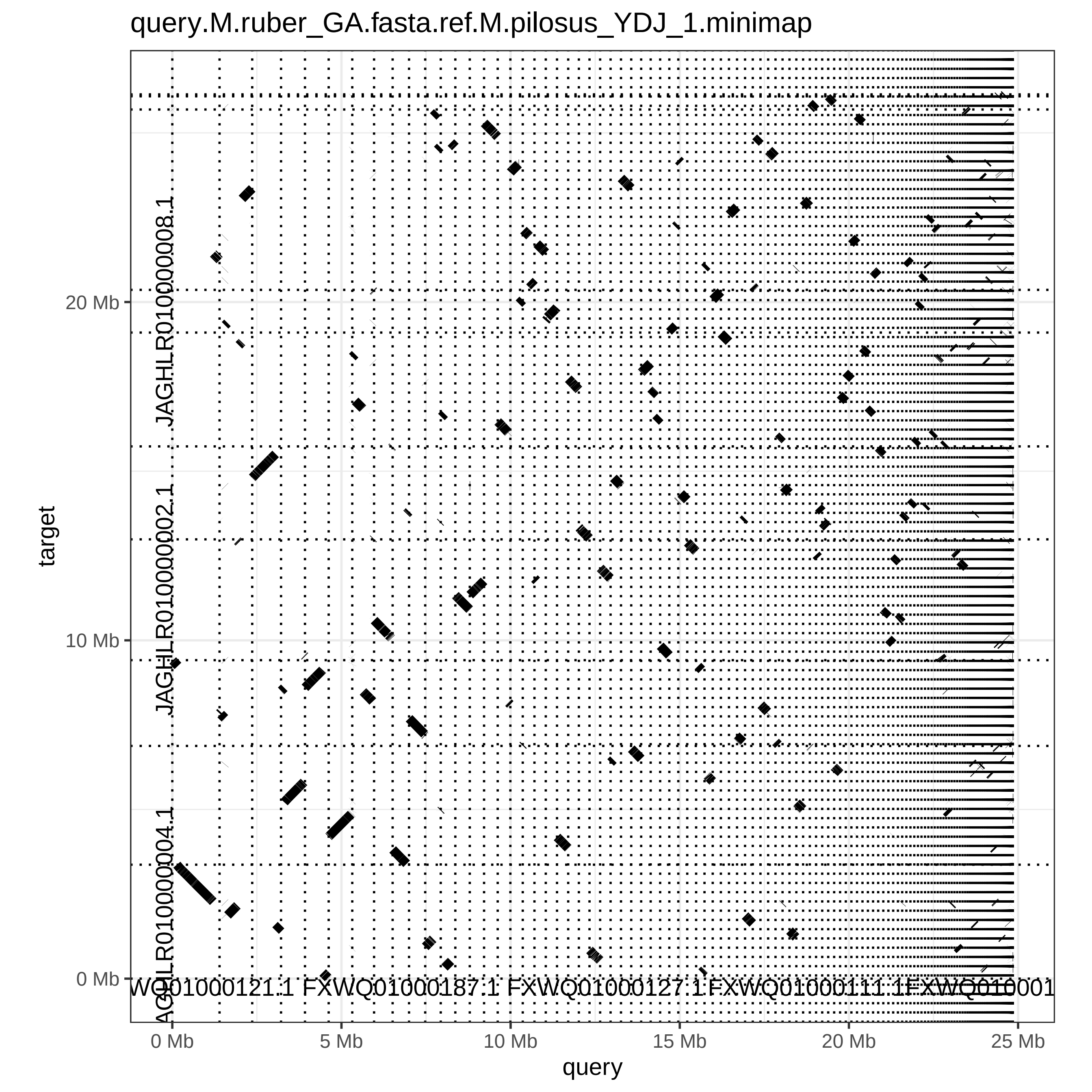

query.M.ruber_GA.fasta.ref.M.pilosus_YDJ_1.minimap
target
JAGHLR010000008.1
JAGHLR010000002.1
JAGHLR010000004.1
20 Mb
10 Mb
0 Mb
WQ01000121.1 FXWQ01000187.1 FXWQ01000127.1 FXWQ01000111.1 FXWQ010001
0 Mb
5 Mb
10 Mb
15 Mb
20 Mb
25 Mb
query

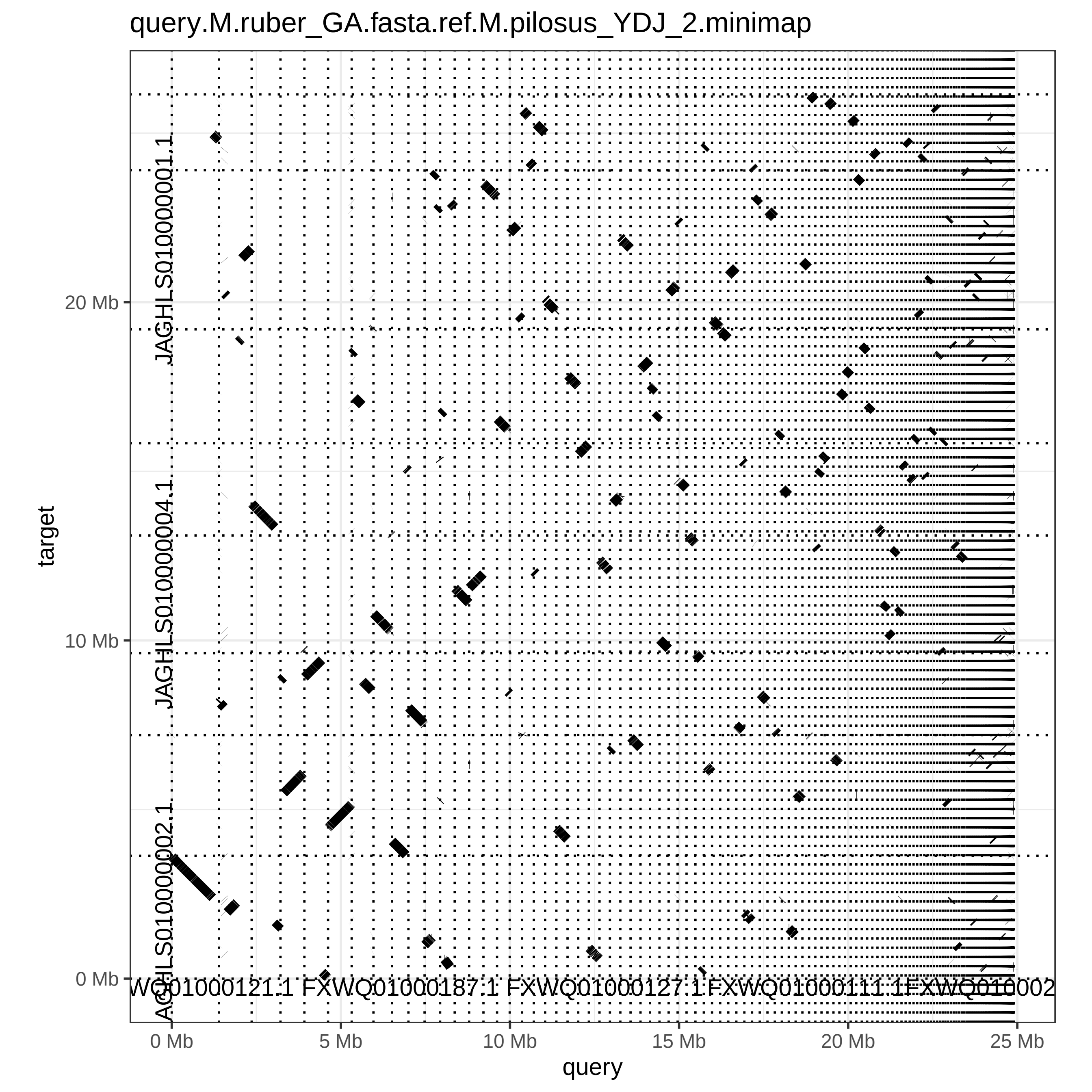

query.M.ruber_GA.fasta.ref.M.pilosus_YDJ_2.minimap
target
query
20 Mb
10 Mb
0 Mb
0 Mb
5 Mb
10 Mb
15 Mb
20 Mb
25 Mb
JAGHLS010000001.1
JAGHLS010000004.1
JAGHLS010000002.1
FXWQ01000187.1
FXWQ01000127.1
FXWQ01000111.1

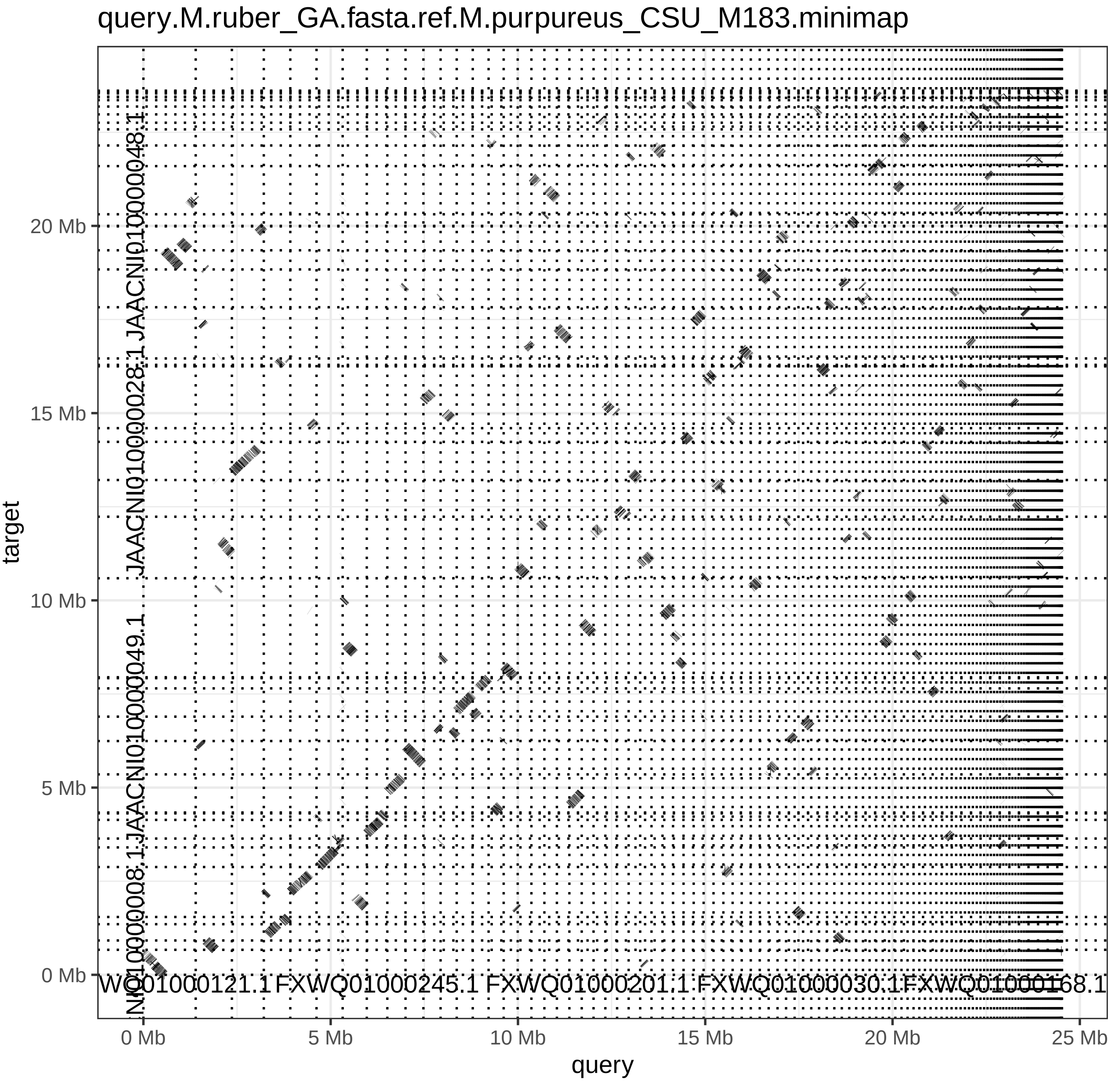
query.M.ruber_GA.fasta.ref.M.purpureus_CSU_M183.minimap
target
query
0 Mb
5 Mb
10 Mb
15 Mb
20 Mb
25 Mb
NI010000008.1
JAACNI010000049.1
JAACNI010000028.1
JAACNI010000048.1
WQ01000121.1
FXWQ01000245.1
FXWQ01000201.1
FXWQ01000030.1
FXWQ01000168.1

query.M.ruber_GA.fasta.ref.M.purpureus_GB_01.minimap

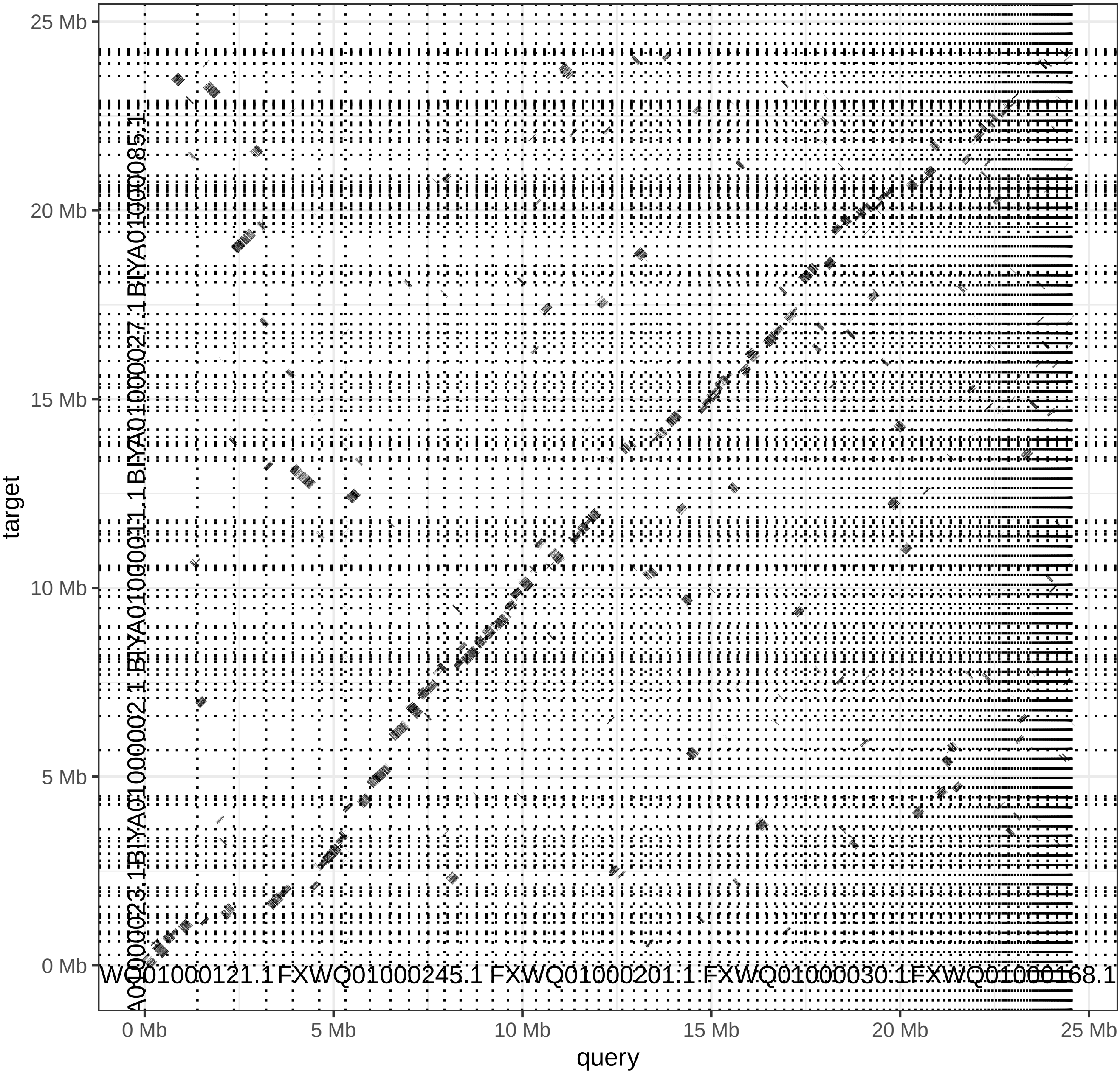

query.M.ruber_GA.fasta.ref.M.purpureus_HQ1.minimap

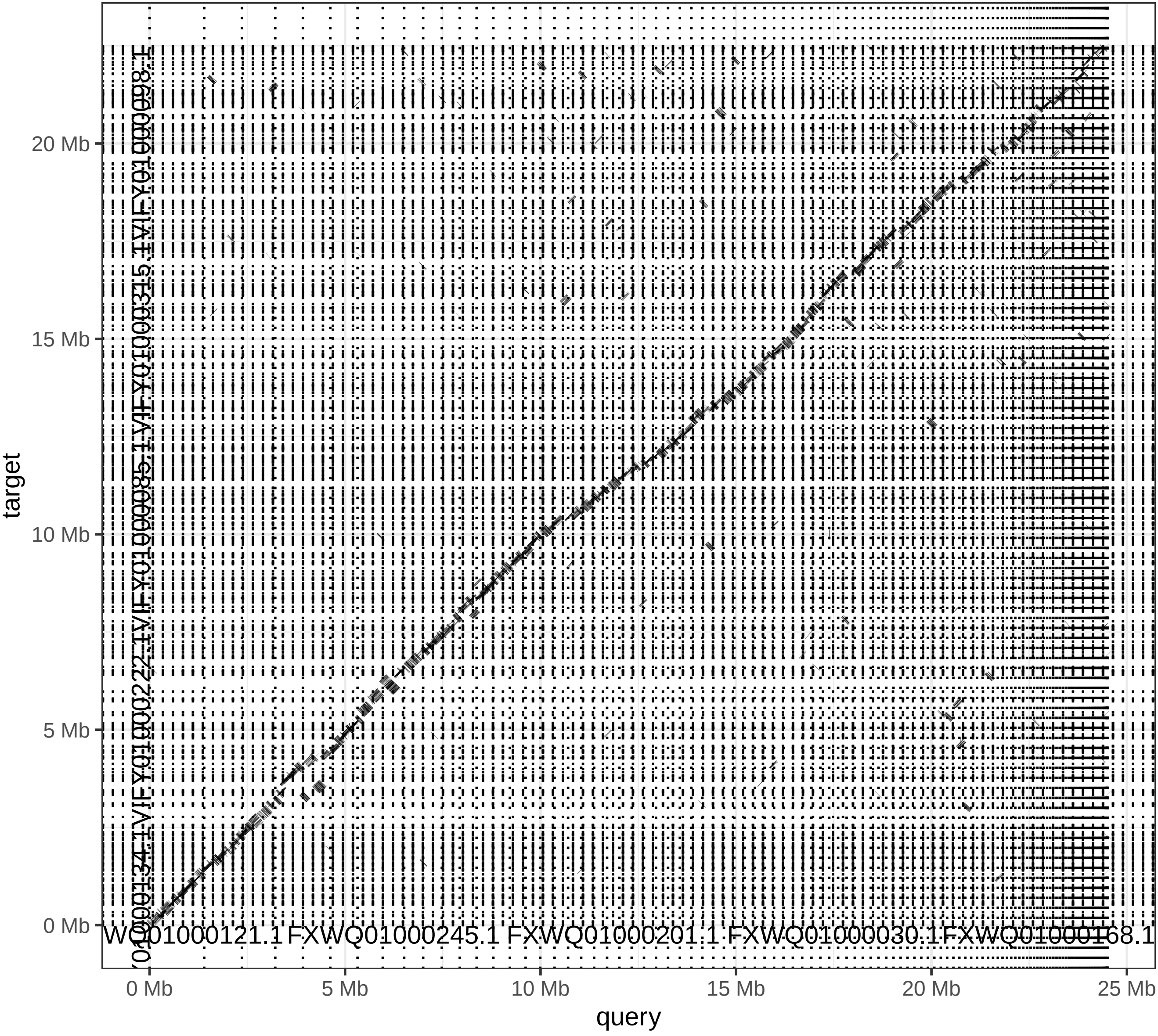

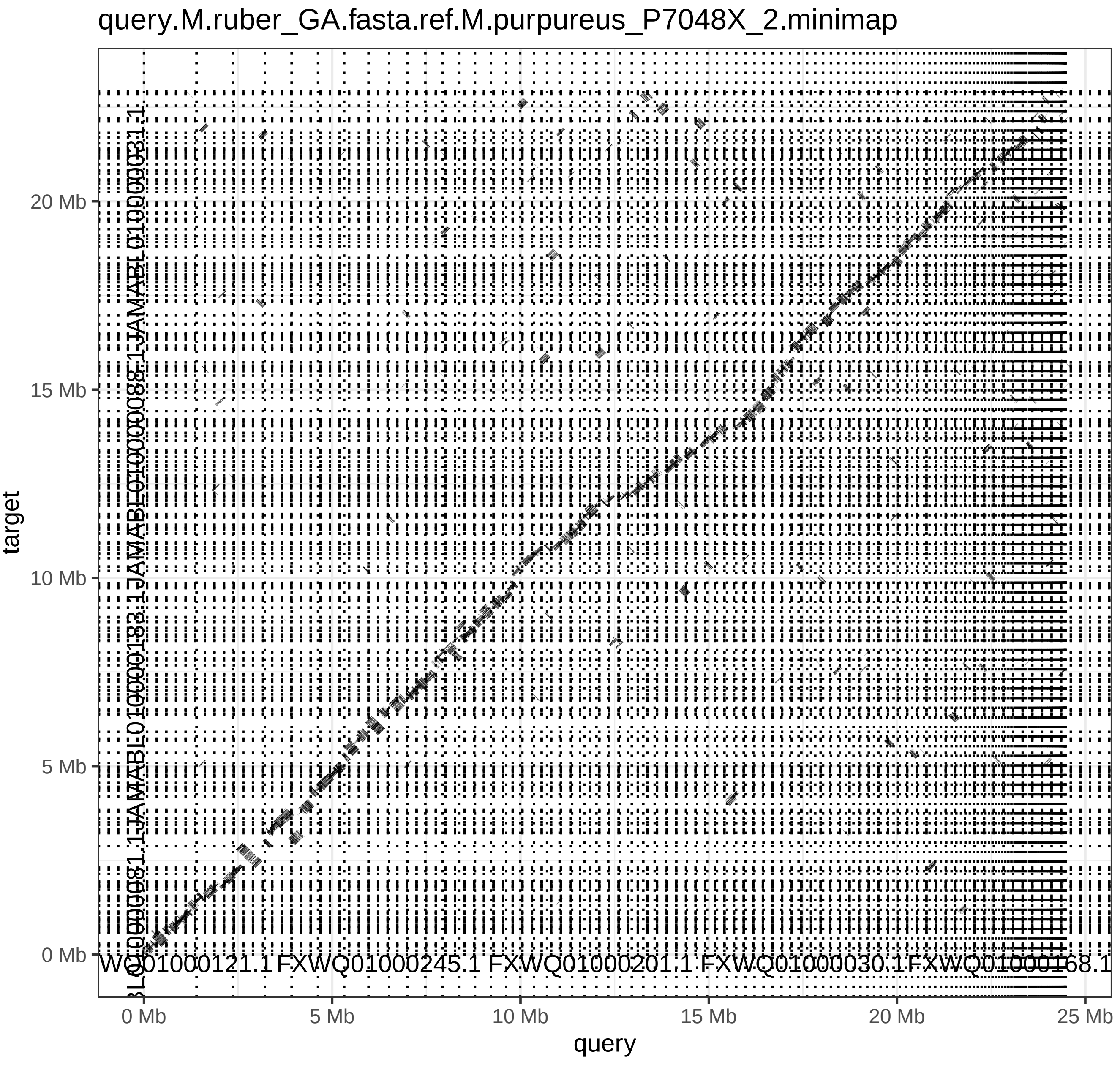
query.M.ruber_GA.fasta.ref.M.purpureus_P7048X_2.minimap
target
20 Mb
15 Mb
10 Mb
5 Mb
0 Mb
FXWQ01000245.1
FXWQ01000201.1
FXWQ01000030.1
FXWQ01000168.1
0 Mb
5 Mb
10 Mb
15 Mb
20 Mb
25 Mb
query

query.M.ruber_GA.fasta.ref.M.purpureus_PF1702S.minimap

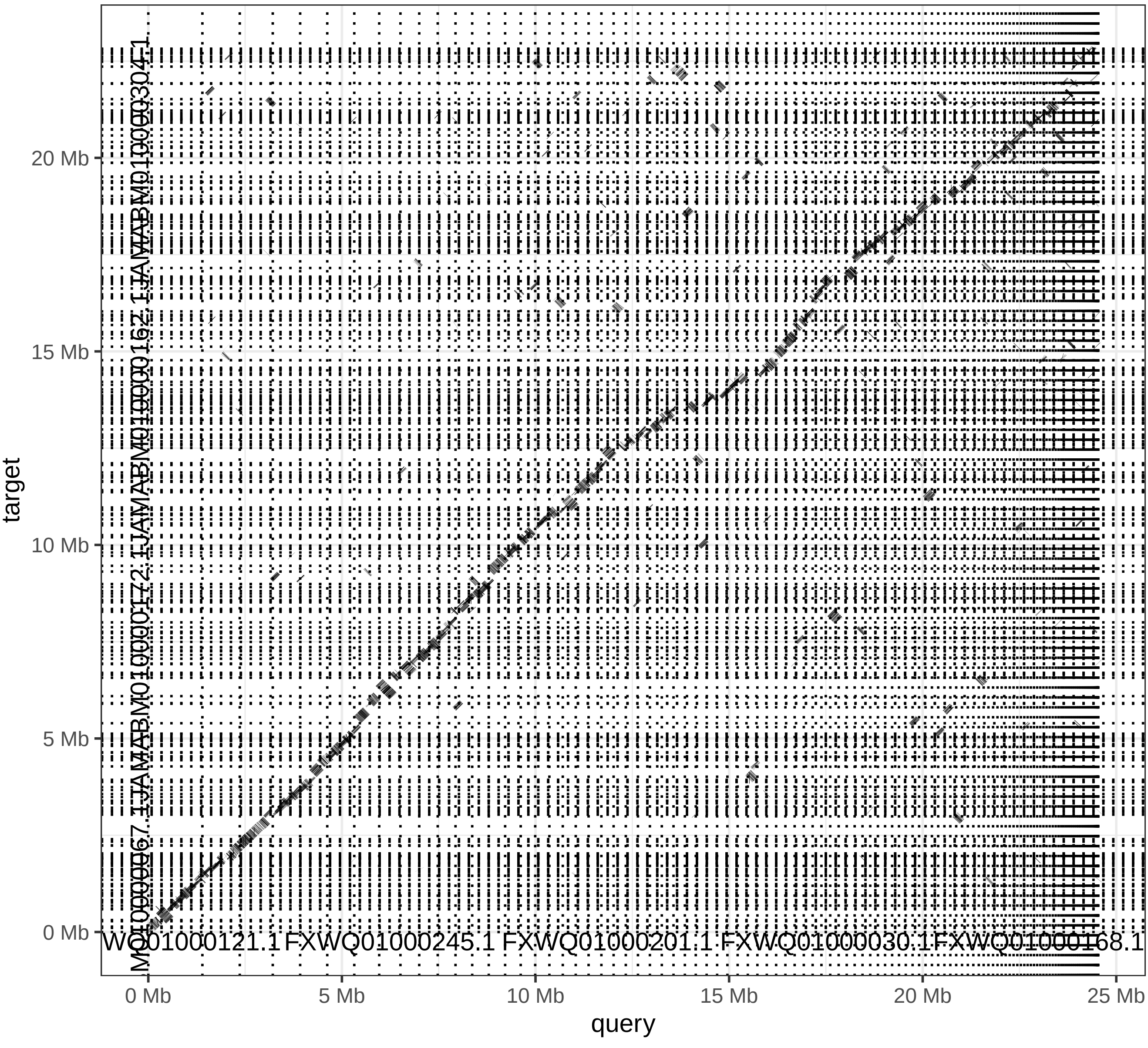

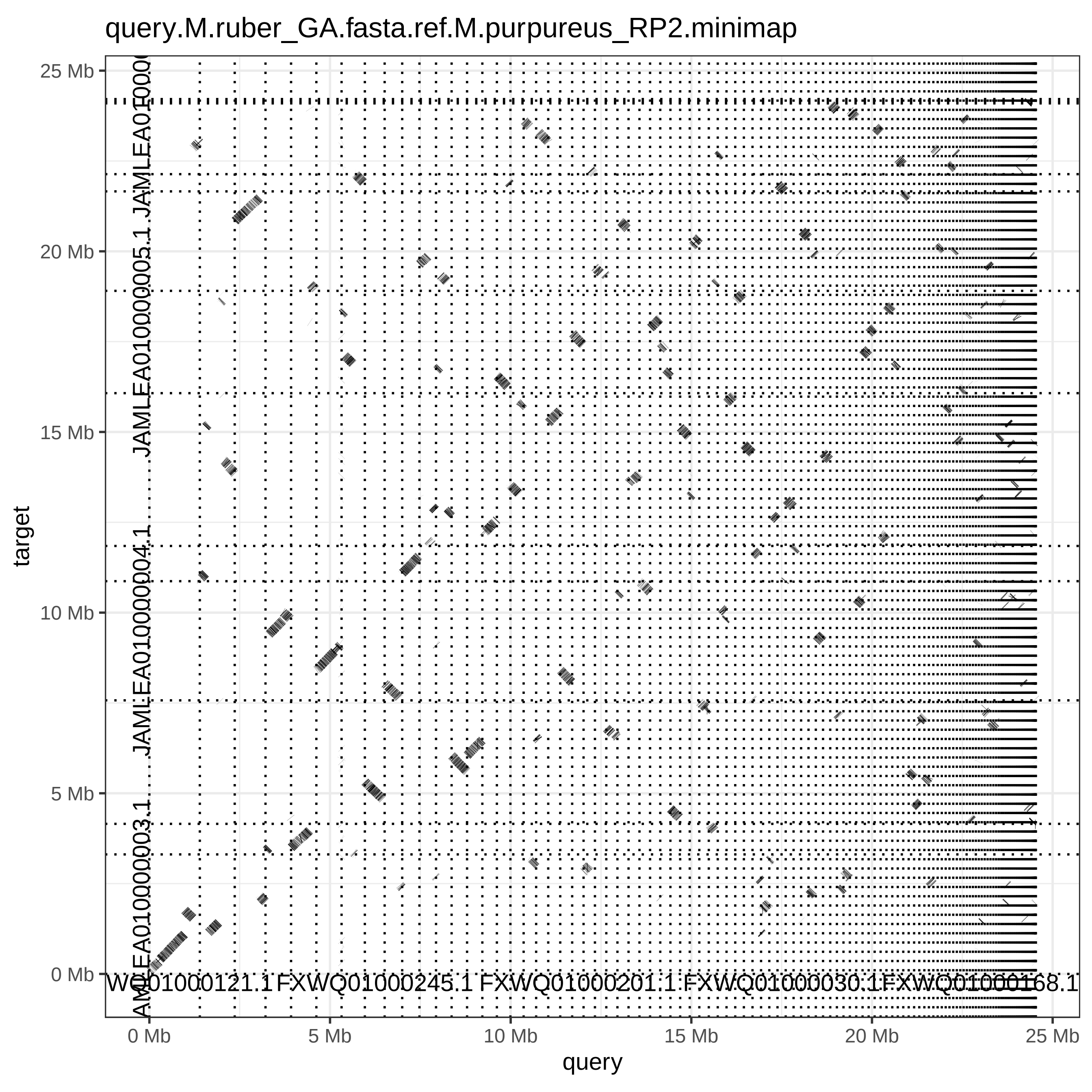

query.M.ruber_GA.fasta.ref.M.purpureus_RP2.minimap
target
query
0 Mb
5 Mb
10 Mb
15 Mb
20 Mb
25 Mb
WQ01000121.1
FXWQ01000245.1
FXWQ01000201.1
FXWQ01000030.1
FXWQ01000168.1
JAMLEA010000003.1
JAMLEA010000004.1
JAMLEA010000005.1

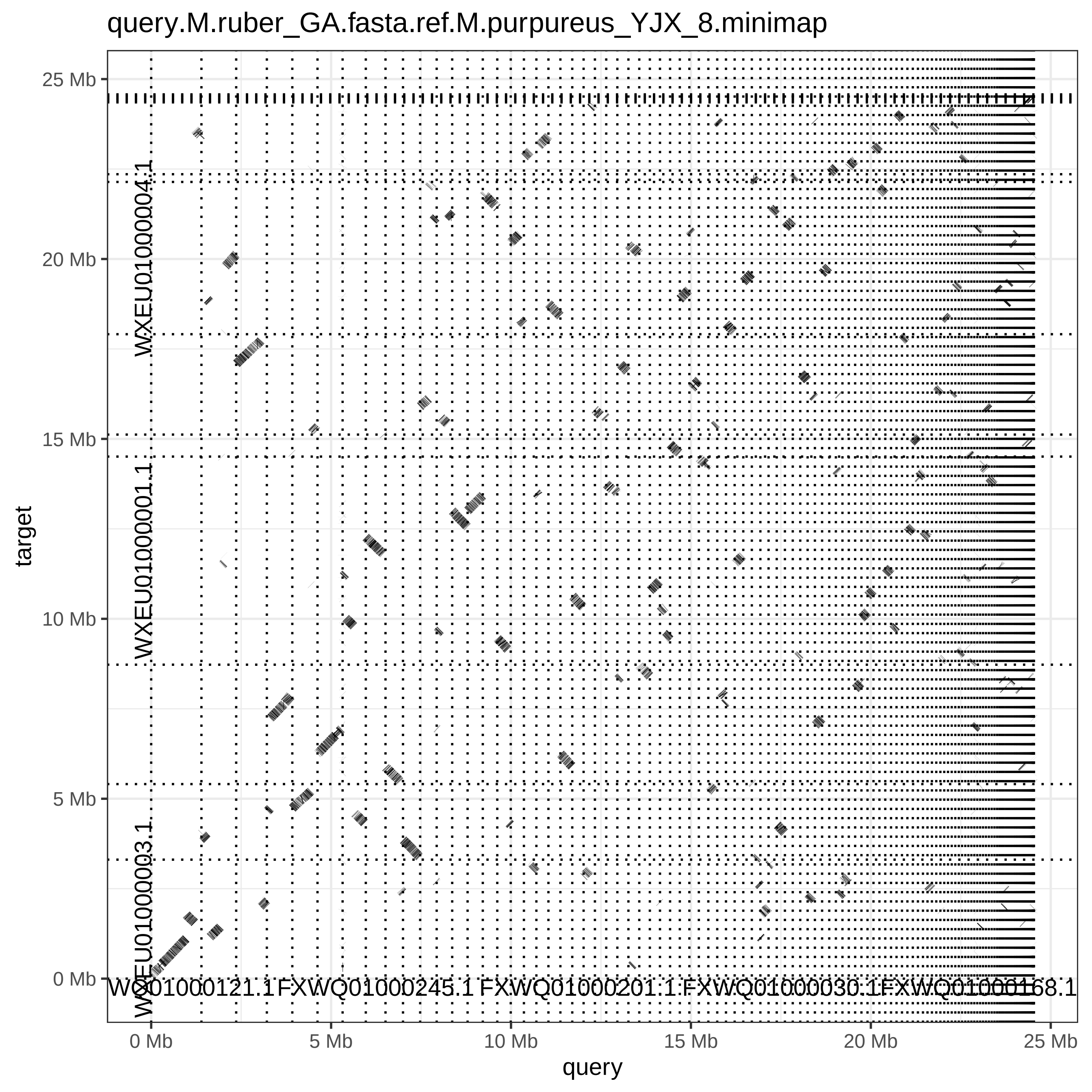
query.M.ruber_GA.fasta.ref.M.purpureus_YJX_8.minimap
target
query
WXEU01000004.1
WXEU01000001.1
WXEU01000003.1
WQ01000121.1
FXWQ01000245.1
FXWQ01000201.1
FXWQ01000030.1
FXWQ01000168.1
0 Mb
5 Mb
10 Mb
15 Mb
20 Mb
25 Mb

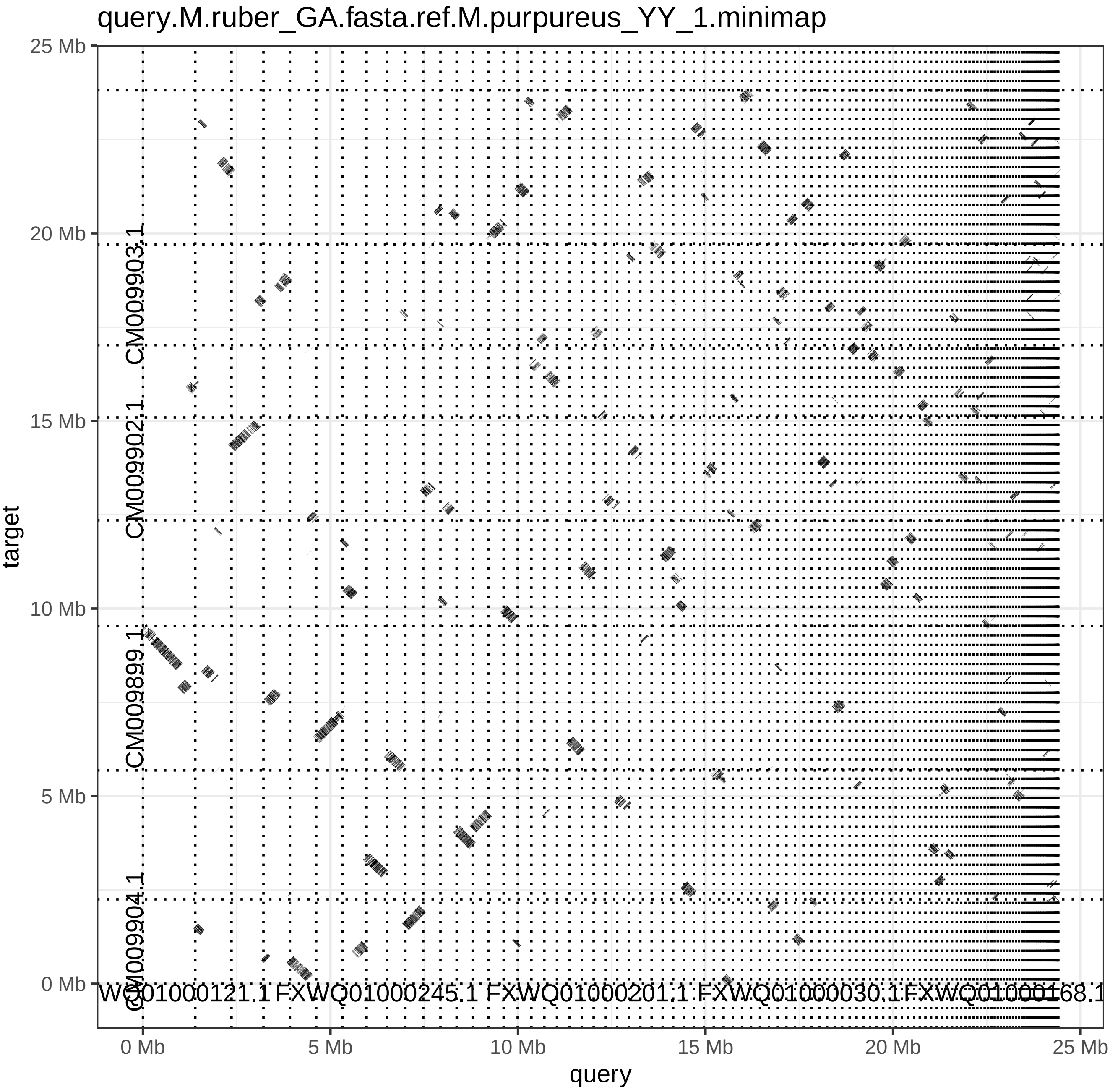
query.M.ruber_GA.fasta.ref.M.purpureus_YY_1.minimap
25 Mb
20 Mb
15 Mb
10 Mb
5 Mb
0 Mb
target
CM009903.1
CM009902.1
CM009899.1
CM009904.1
WQ01000121.1 FXWQ01000245.1 FXWQ01000201.1 FXWQ01000030.1FXWQ01000168.1
0 Mb
5 Mb
10 Mb
15 Mb
20 Mb
25 Mb
query

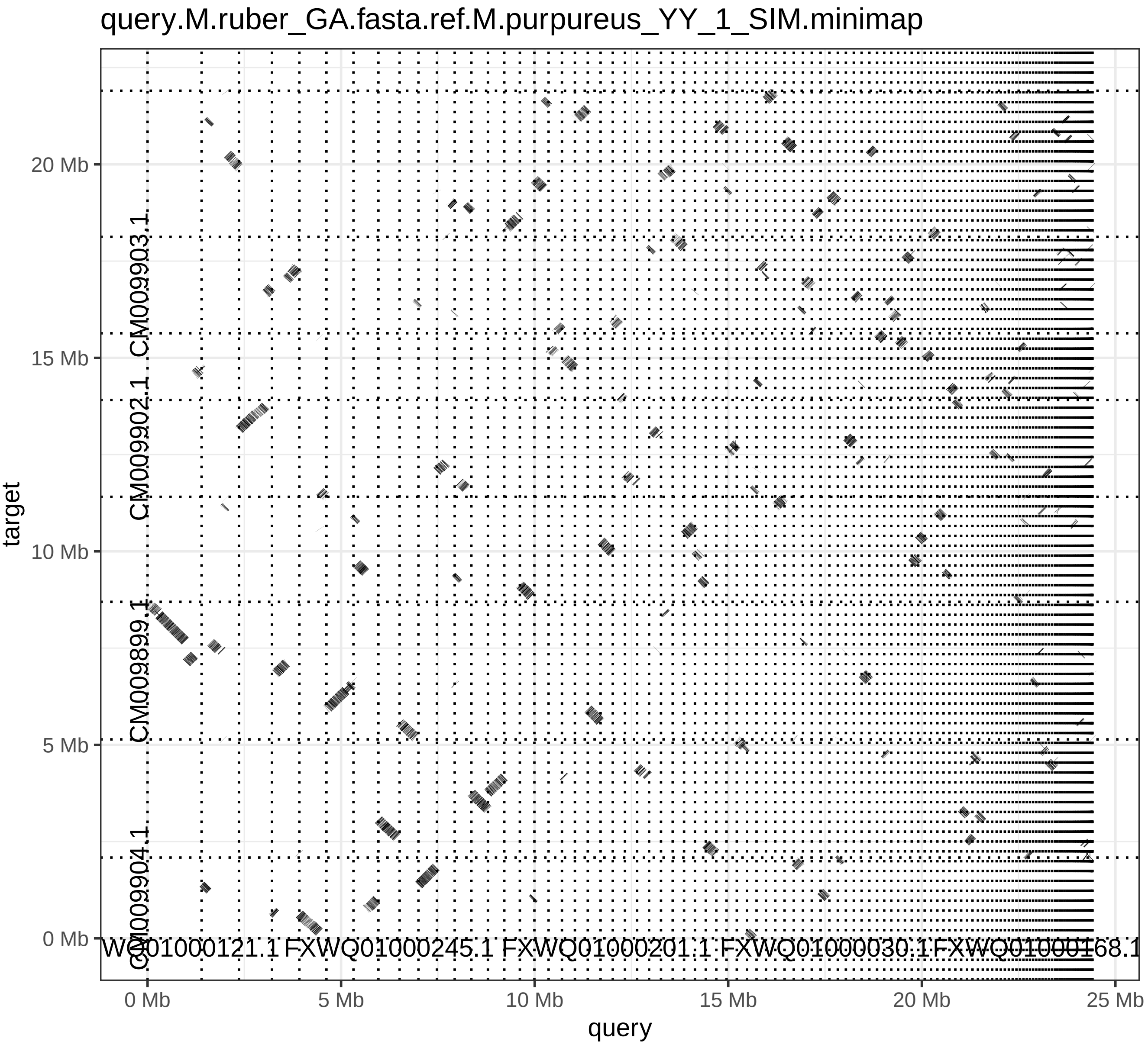

query.M.ruber_GA.fasta.ref.M.purpureus_YY_1_SIM.minimap
target
20 Mb
15 Mb
10 Mb
5 Mb
0 Mb
CM009903.1
CM009902.1
CM009899.1
CM009904.1
WQ01000121.1
FXWQ01000245.1
FXWQ01000201.1
FXWQ01000030.1
FXWQ01000168.1
0 Mb
5 Mb
10 Mb
15 Mb
20 Mb
25 Mb
query

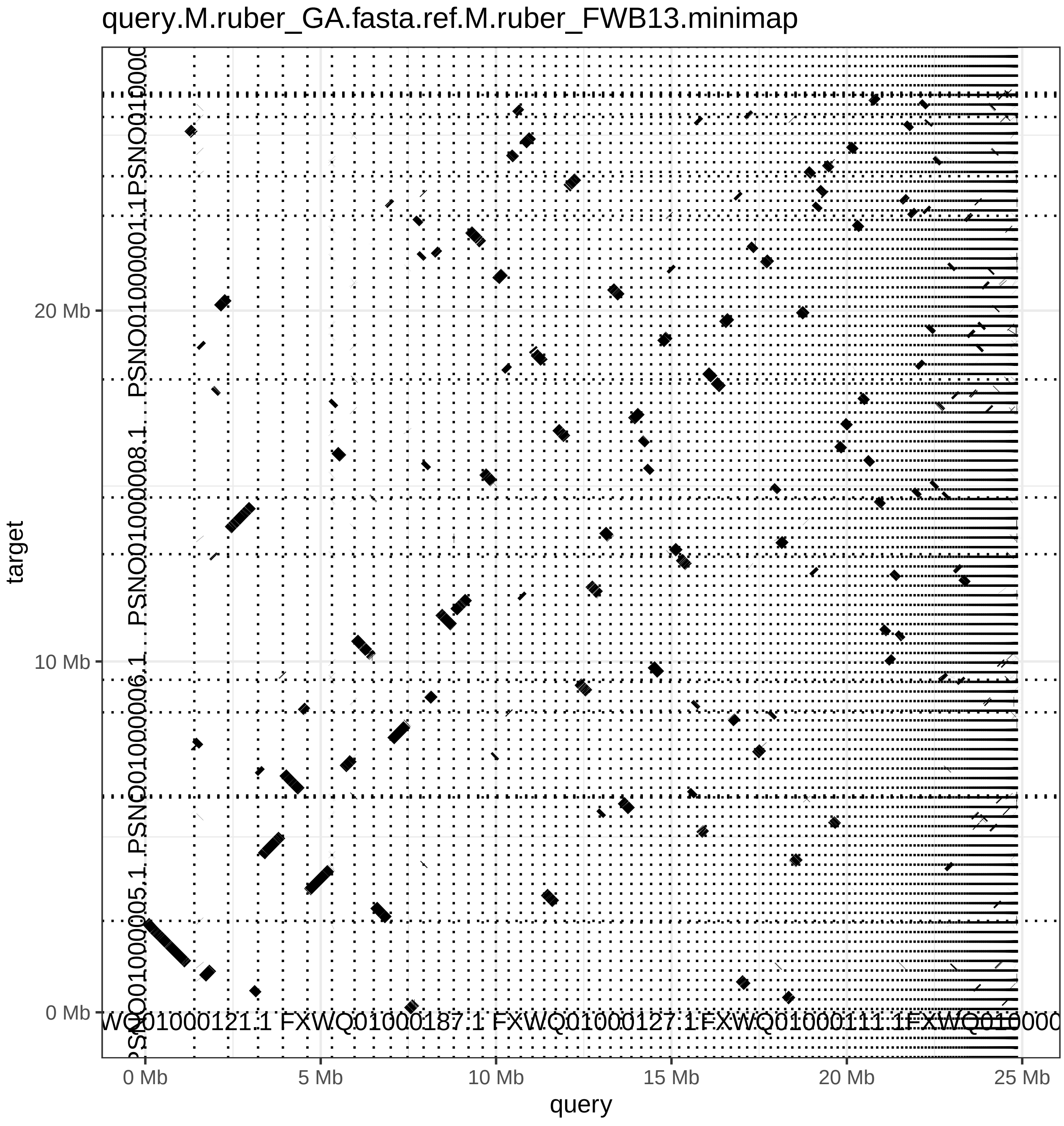
query.M.ruber_GA.fasta.ref.M.ruber_FWB13.minimap
target
query
0 Mb
10 Mb
20 Mb
5 Mb
15 Mb
25 Mb
FXWQ01000187.1
FXWQ01000127.1
PSNO01000008.1
PSNO01000006.1
PSNO01000005.1

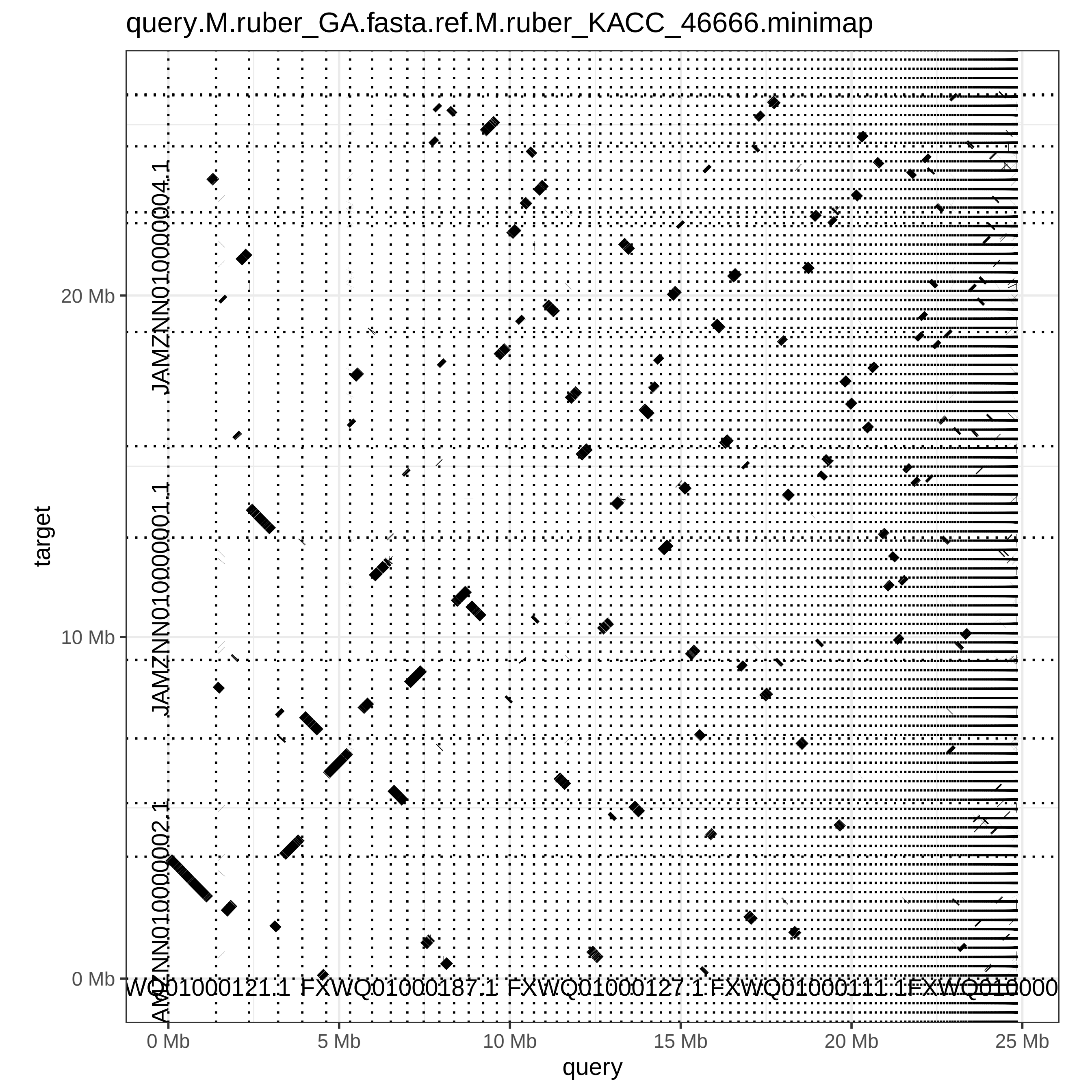
query.M.ruber_GA.fasta.ref.M.ruber_KACC_46666.minimap
target
query
0 Mb
5 Mb
10 Mb
15 Mb
20 Mb
25 Mb
0 Mb
10 Mb
20 Mb
JAMZNN010000004.1
JAMZNN010000001.1
JAMZNN010000002.1
FXWQ01000121.1
FXWQ01000187.1
FXWQ01000127.1
FXWQ01000111.1

query.M.ruber_KACC_46666.fasta.ref.M.pilosus_K104061.minimap

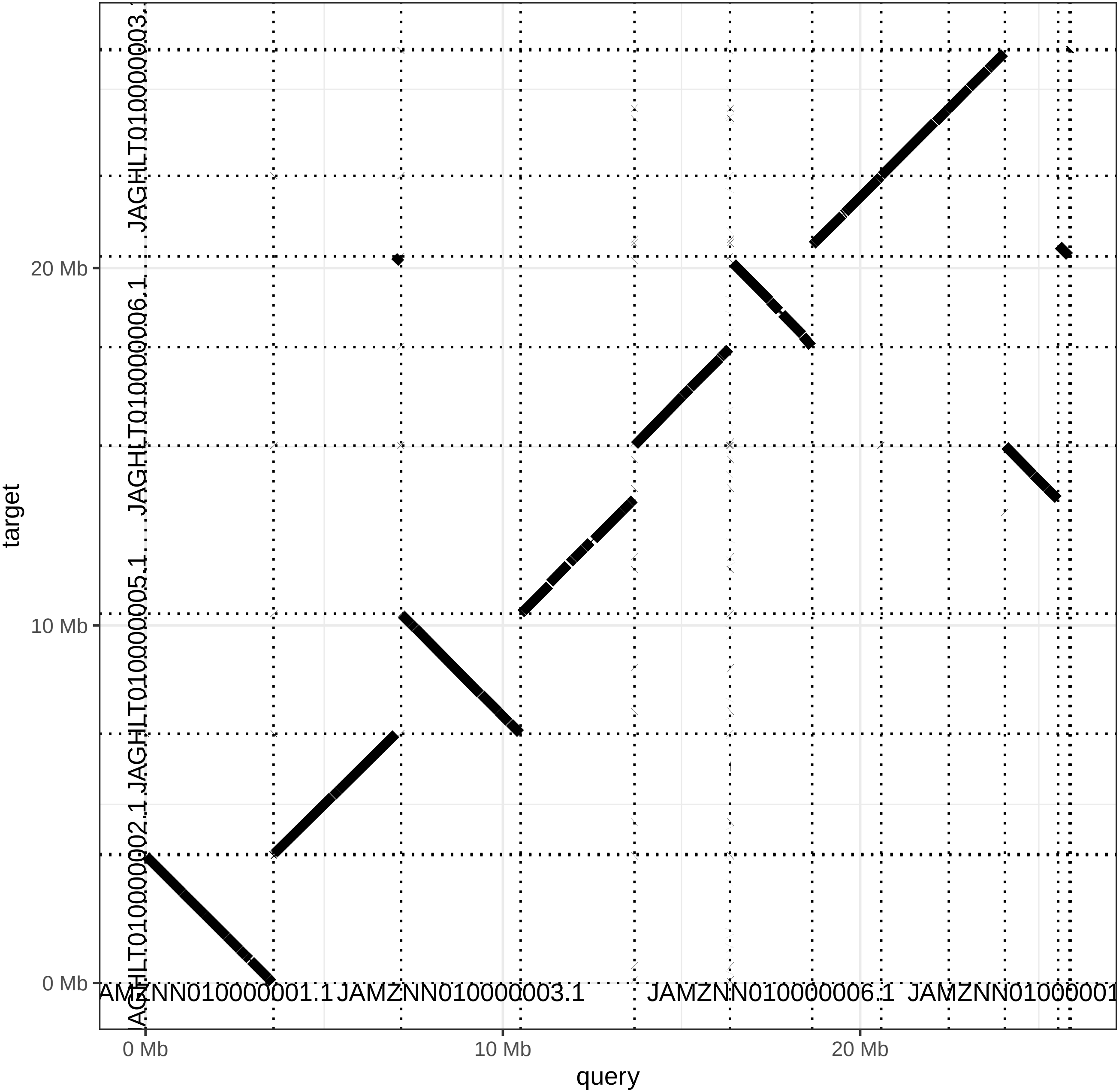

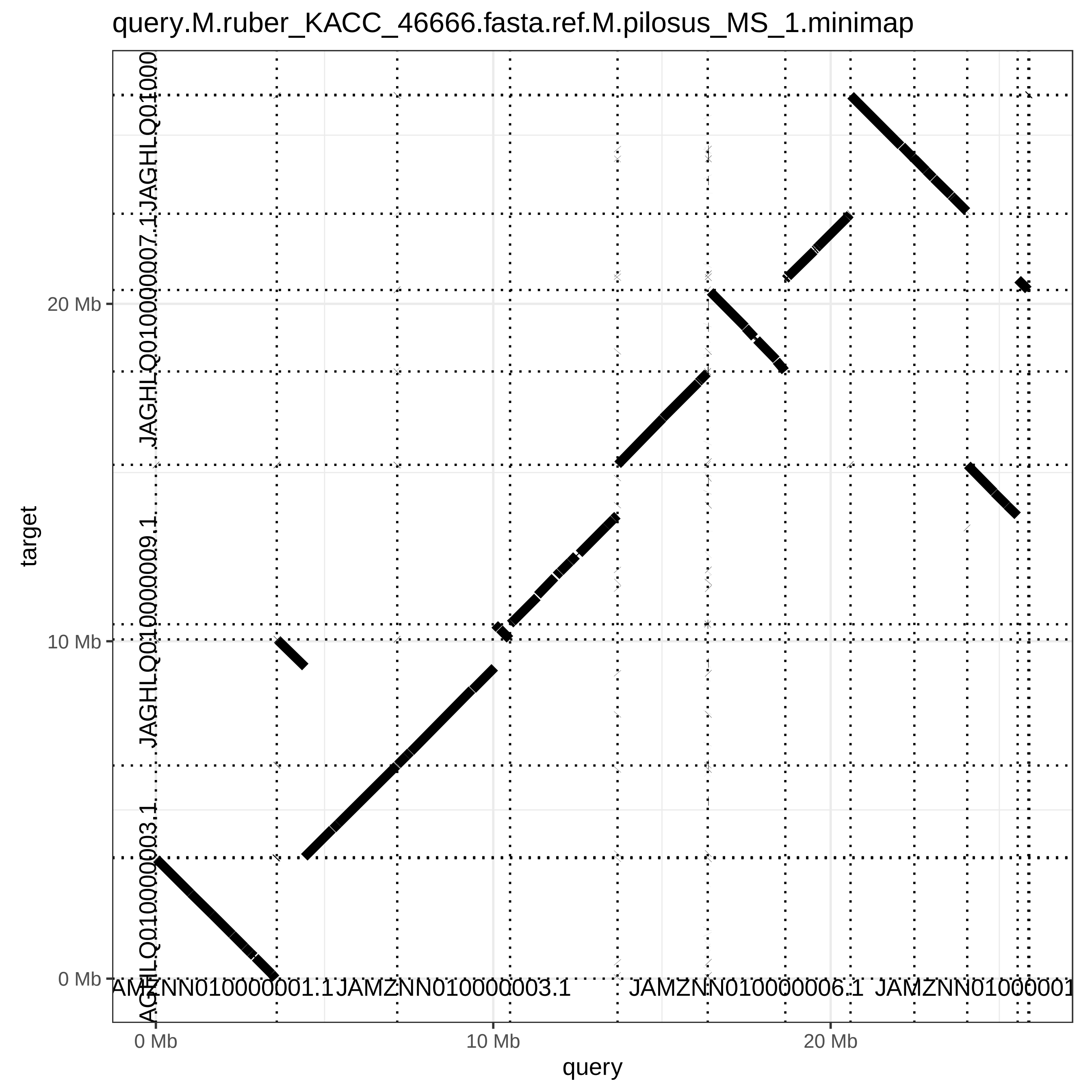
query.M.ruber_KACC_46666.fasta.ref.M.pilosus_MS_1.minimap
target
query
0 Mb
10 Mb
20 Mb
JAGHLQ010000007.1
JAGHLQ010000009.1
AGHLQ010000003.1
JAGHLQ01000
AMZNN010000001.1
JAMZNN010000003.1
JAMZNN010000006.1
JAMZNN01000001

query.M.ruber_KACC_46666.fasta.ref.M.pilosus_YDJ_1.minimap

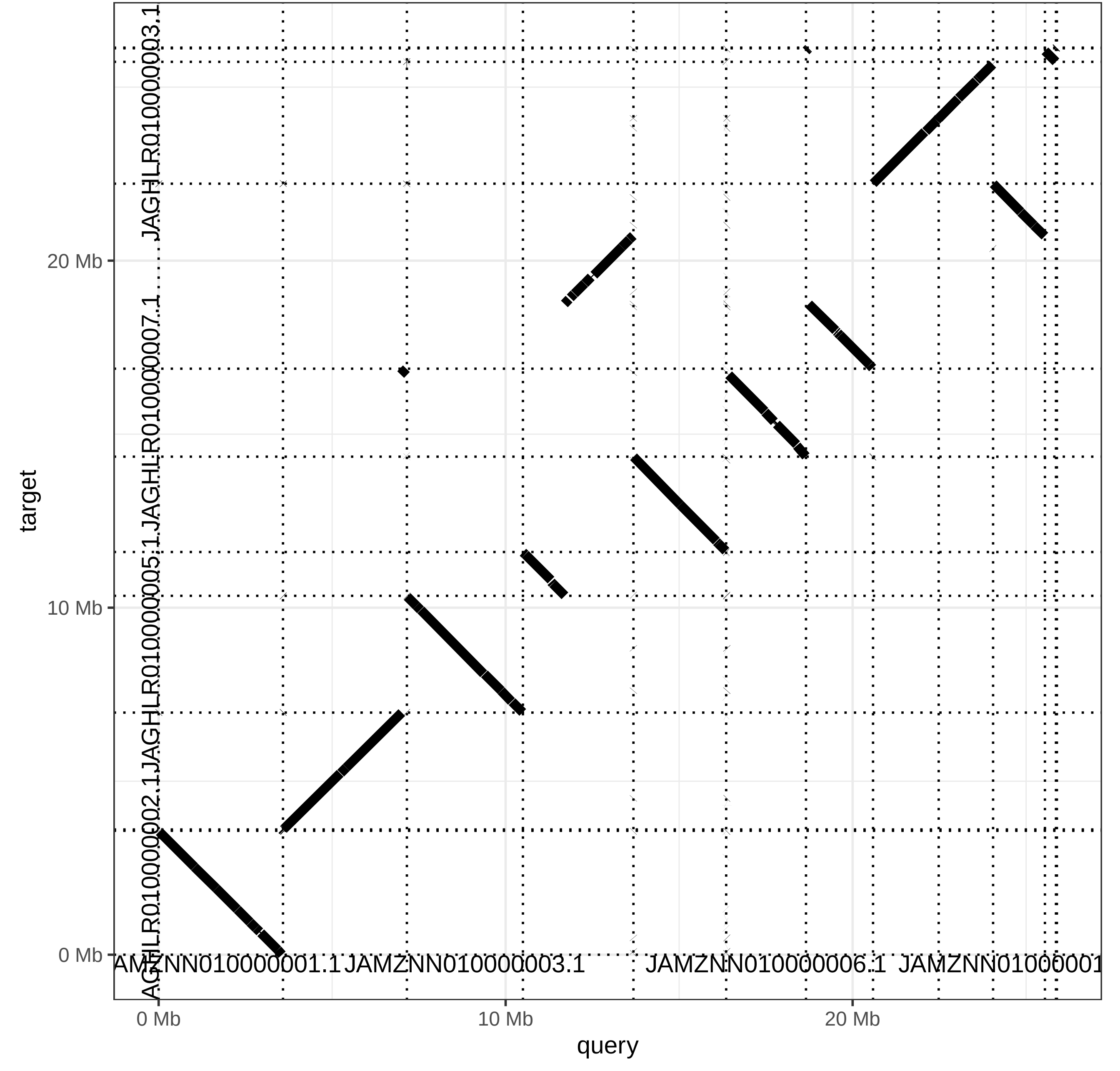

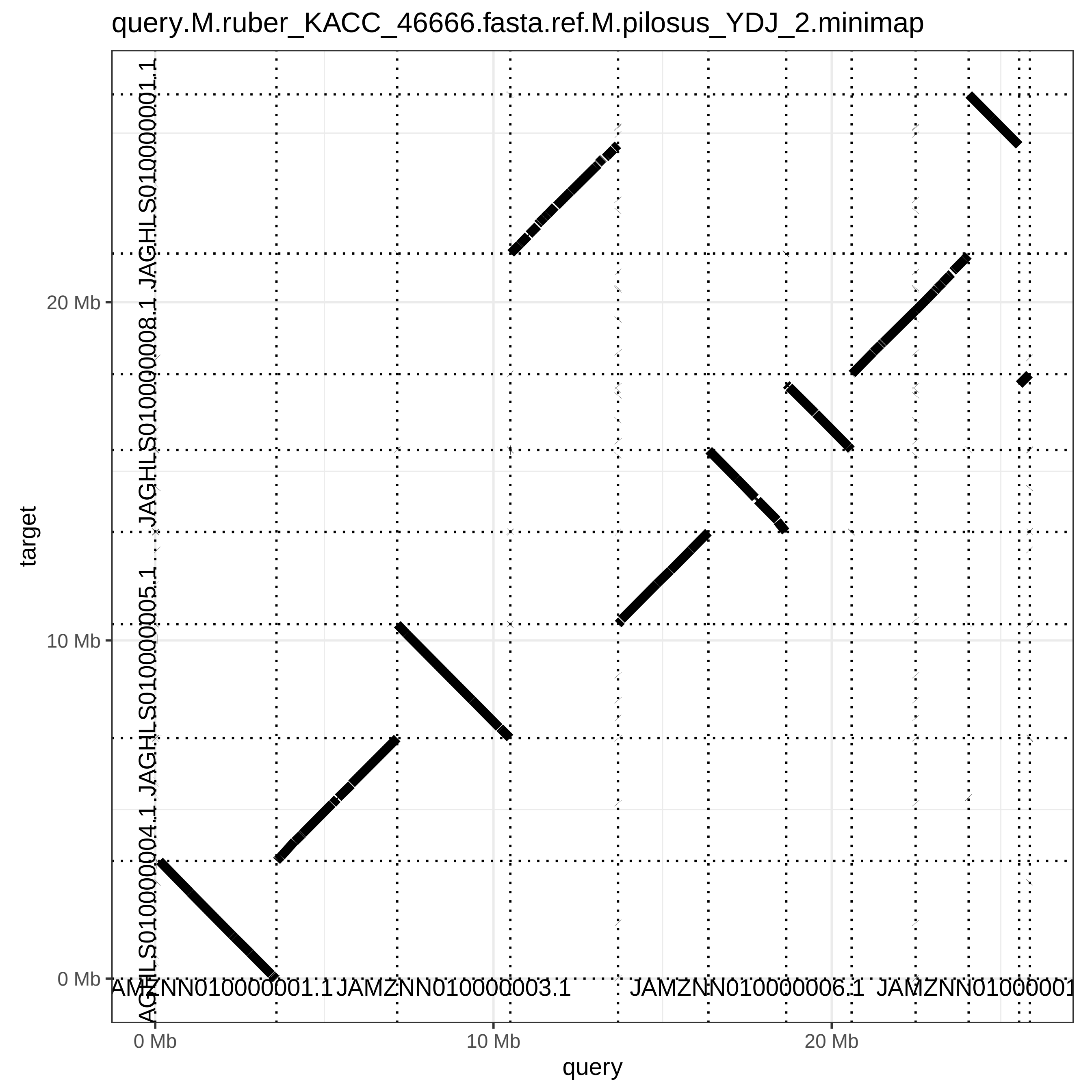

query.M.ruber_KACC_46666.fasta.ref.M.pilosus_YDJ_2.minimap
target
query
0 Mb
10 Mb
20 Mb
JAMZNN010000001.1
JAMZNN010000003.1
JAMZNN010000006.1
JAMZNN01000001
JAGHLS010000001.1
JAGHLS010000008.1
JAGHLS010000005.1
JAGHLS010000004.1

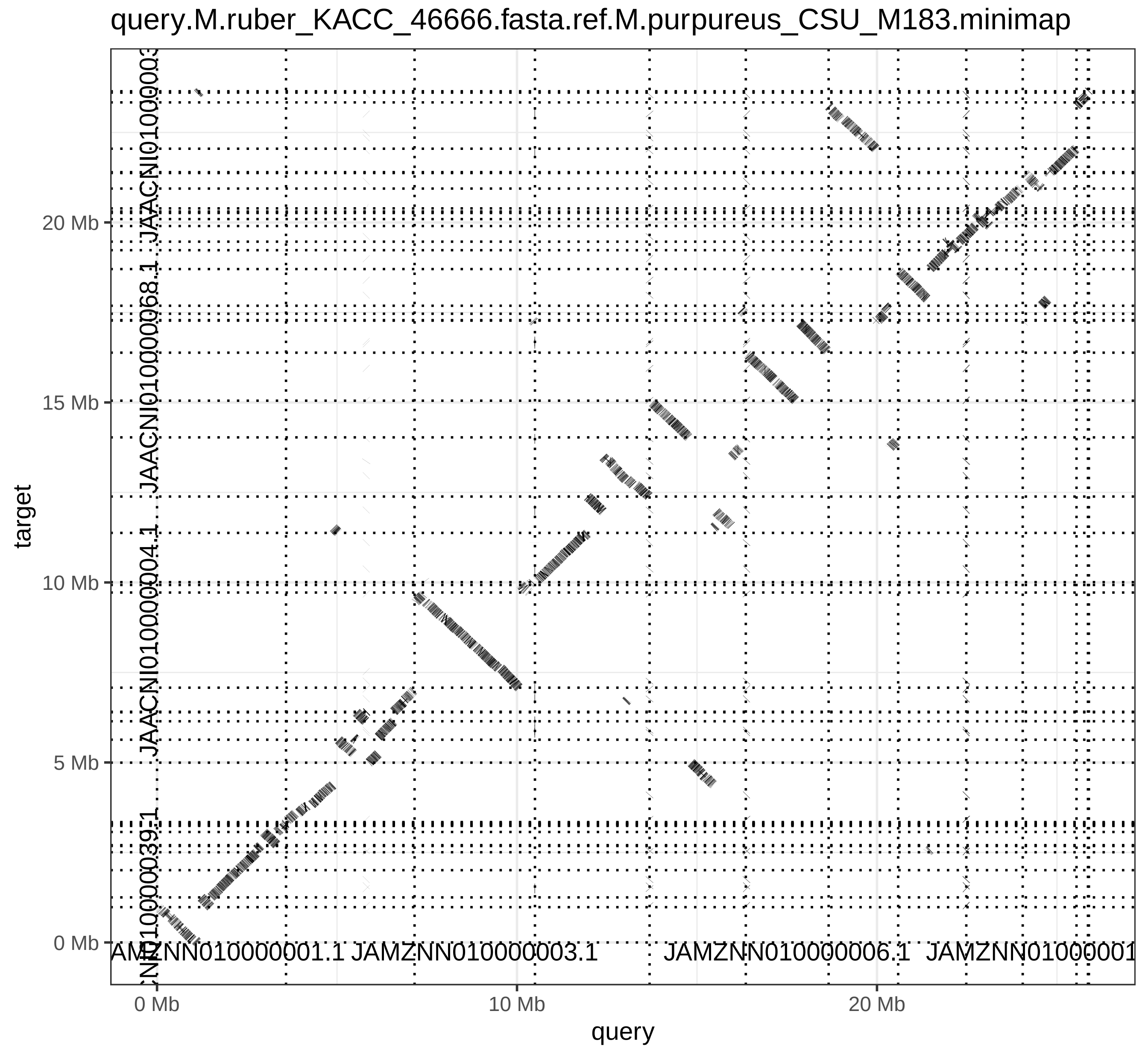
query.M.ruber_KACC_46666.fasta.ref.M.purpureus_CSU_M183.minimap
target
query
0 Mb
5 Mb
10 Mb
15 Mb
20 Mb
0 Mb
10 Mb
20 Mb
AMZNN010000001.1
JAMZNN010000003.1
JAMZNN010000006.1
JAMZNN01000001
JAACNI010000068.1
JAACNI010000004.1
CNI010000039.1

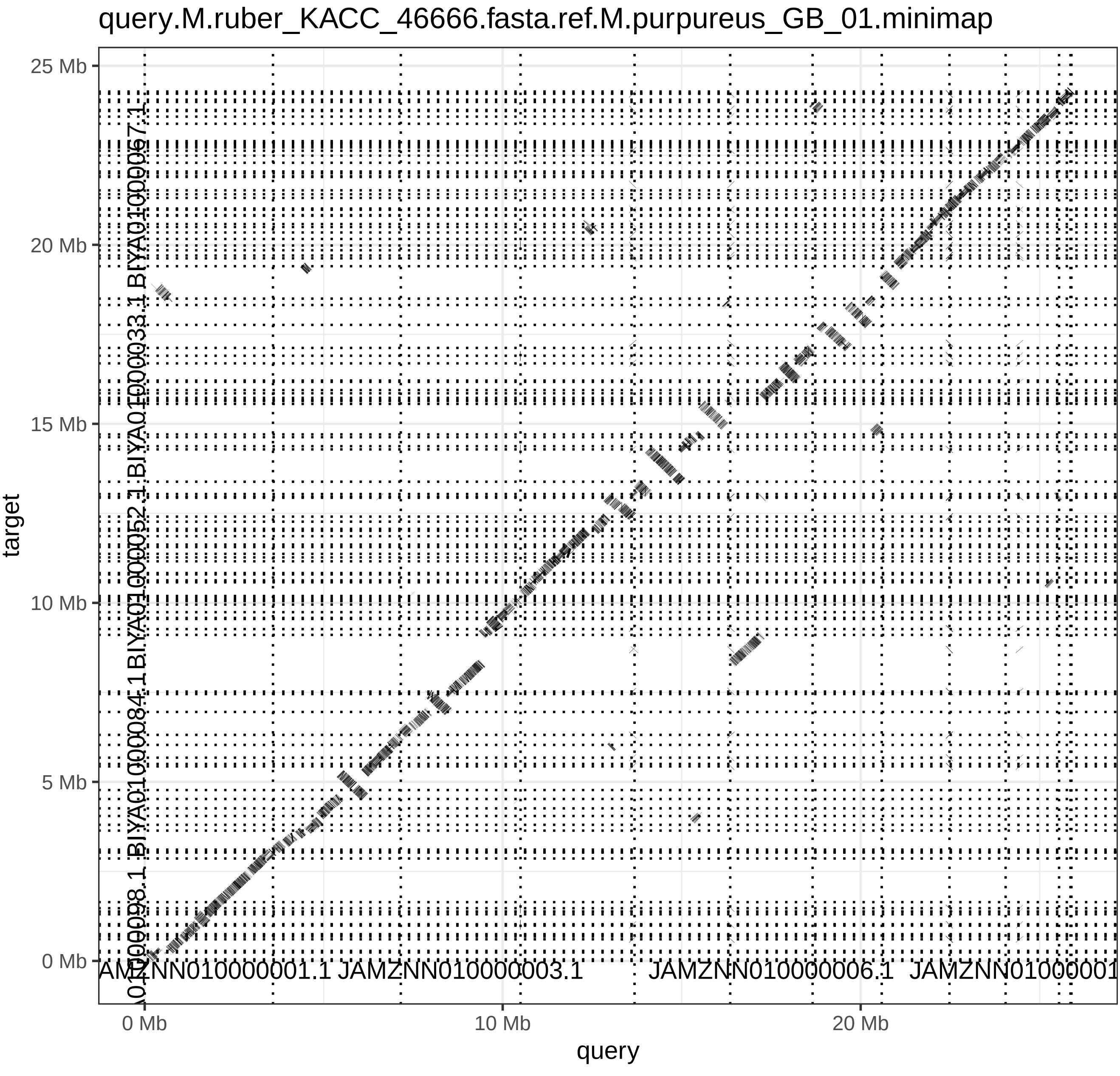
query.M.ruber_KACC_46666.fasta.ref.M.purpureus_GB_01.minimap
25 Mb
20 Mb
15 Mb
10 Mb
5 Mb
0 Mb
target
0 Mb
10 Mb
20 Mb
query
JAMZNN010000003.1
JAMZNN010000006.1

query.M.ruber_KACC_46666.fasta.ref.M.purpureus_HQ1.minimap

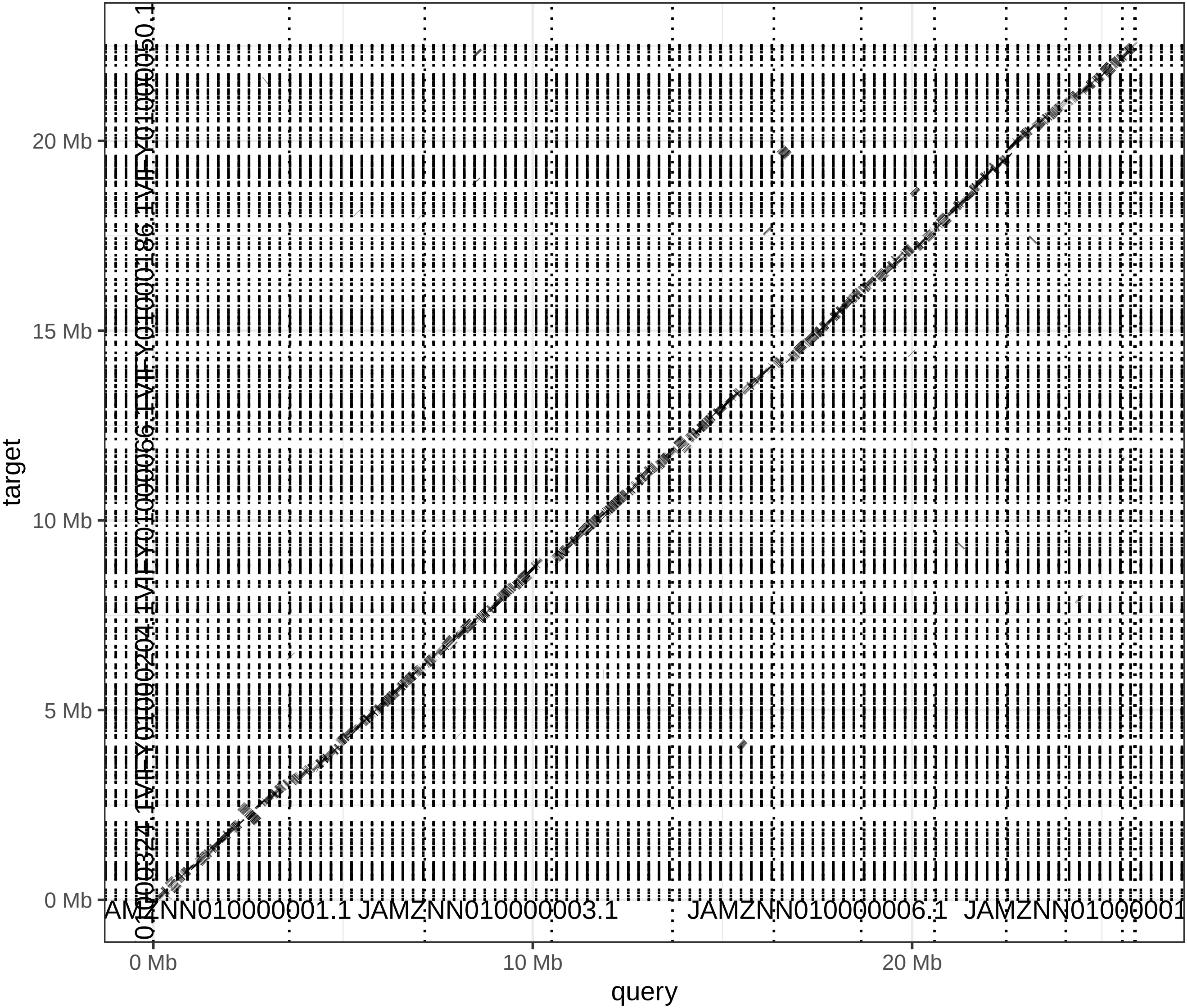

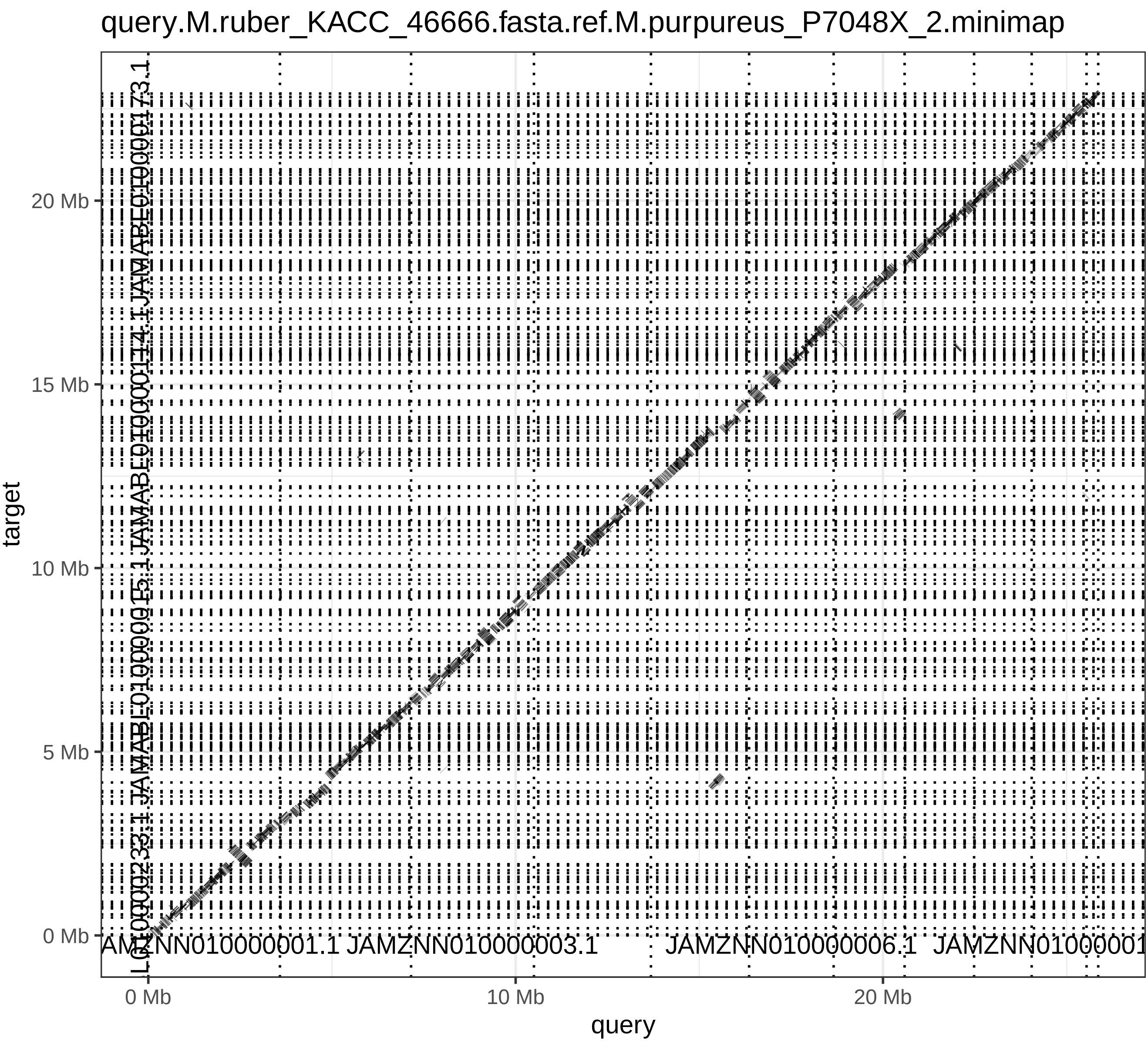

query.M.ruber_KACC_46666.fasta.ref.M.purpureus_P7048X_2.minimap
target
query
0 Mb
5 Mb
10 Mb
15 Mb
20 Mb
0 Mb
10 Mb
20 Mb
JAMZNN010000003.1
JAMZNN010000006.1

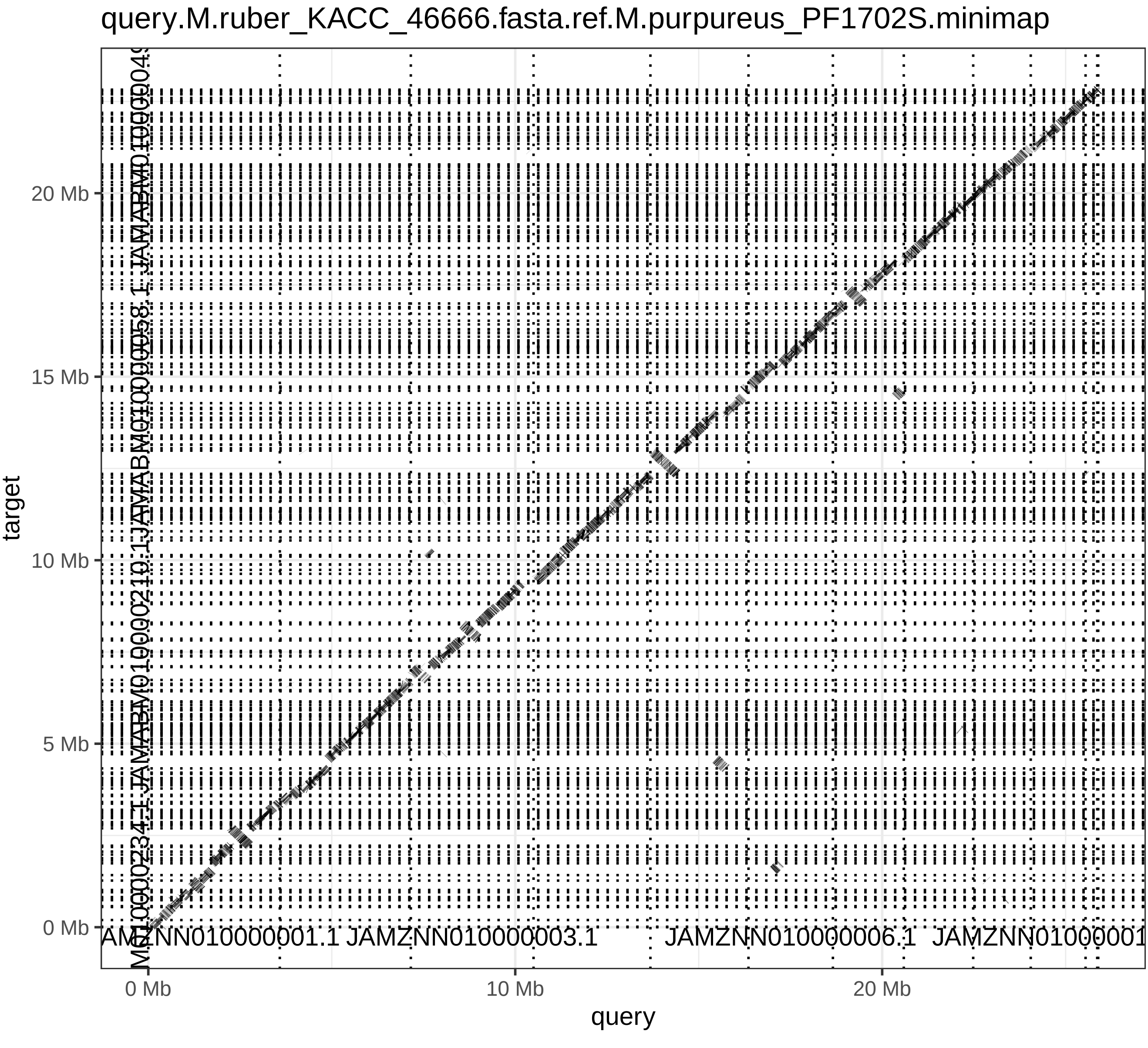
query.M.ruber_KACC_46666.fasta.ref.M.purpureus_PF1702S.minimap
target
20 Mb
15 Mb
10 Mb
5 Mb
0 Mb
JAMZNN010000001.1
JAMZNN010000003.1
JAMZNN010000006.1
0 Mb
10 Mb
20 Mb
query

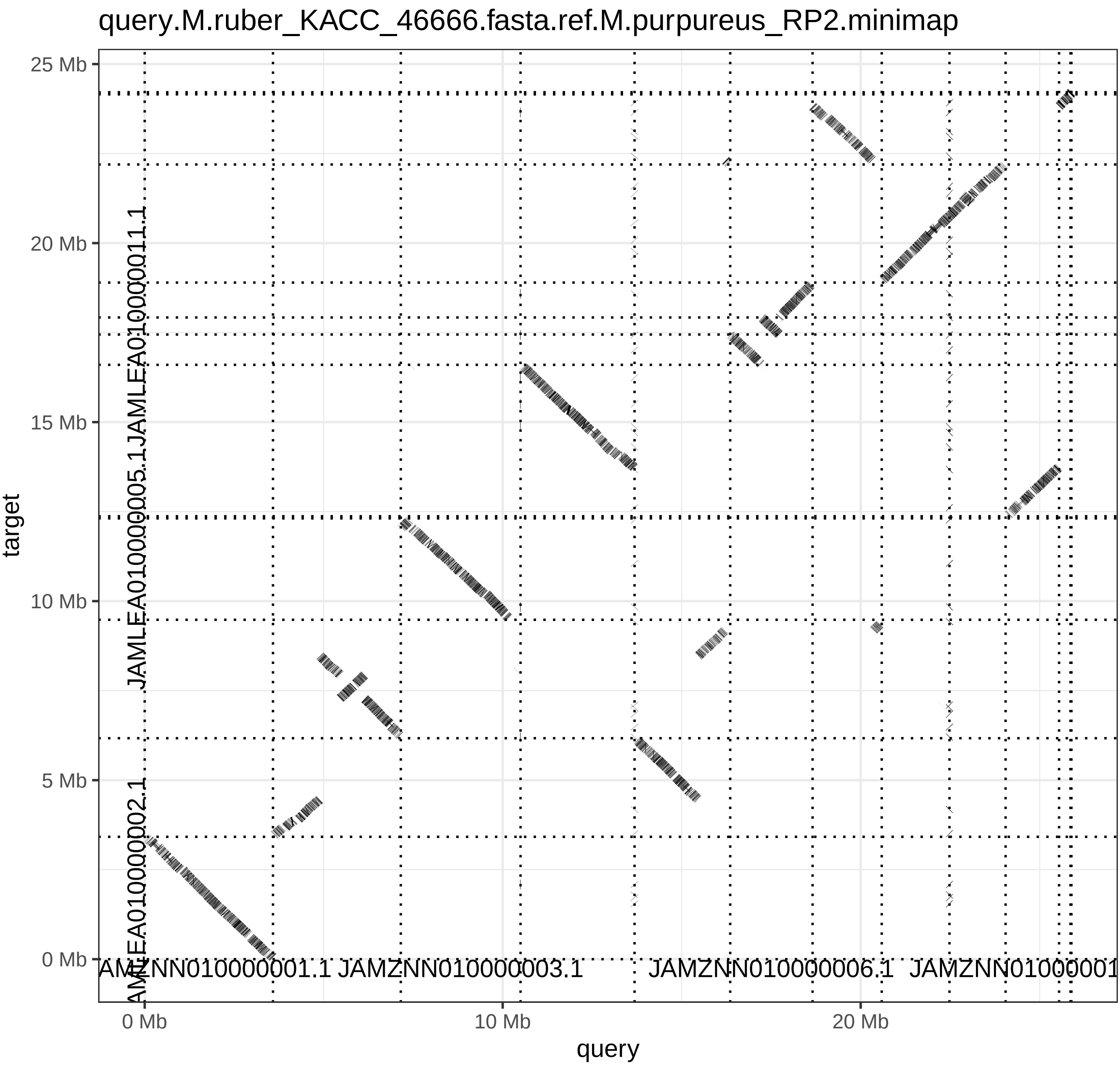

query.M.ruber_KACC_46666.fasta.ref.M.purpureus_RP2.minimap
25 Mb
20 Mb
15 Mb
10 Mb
5 Mb
0 Mb
target
JAMLEA010000011.1
JAMLEA010000005.1
JAMLEA010000002.1
JAMZNN010000001.1
JAMZNN010000003.1
JAMZNN010000006.1
0 Mb
10 Mb
20 Mb
query

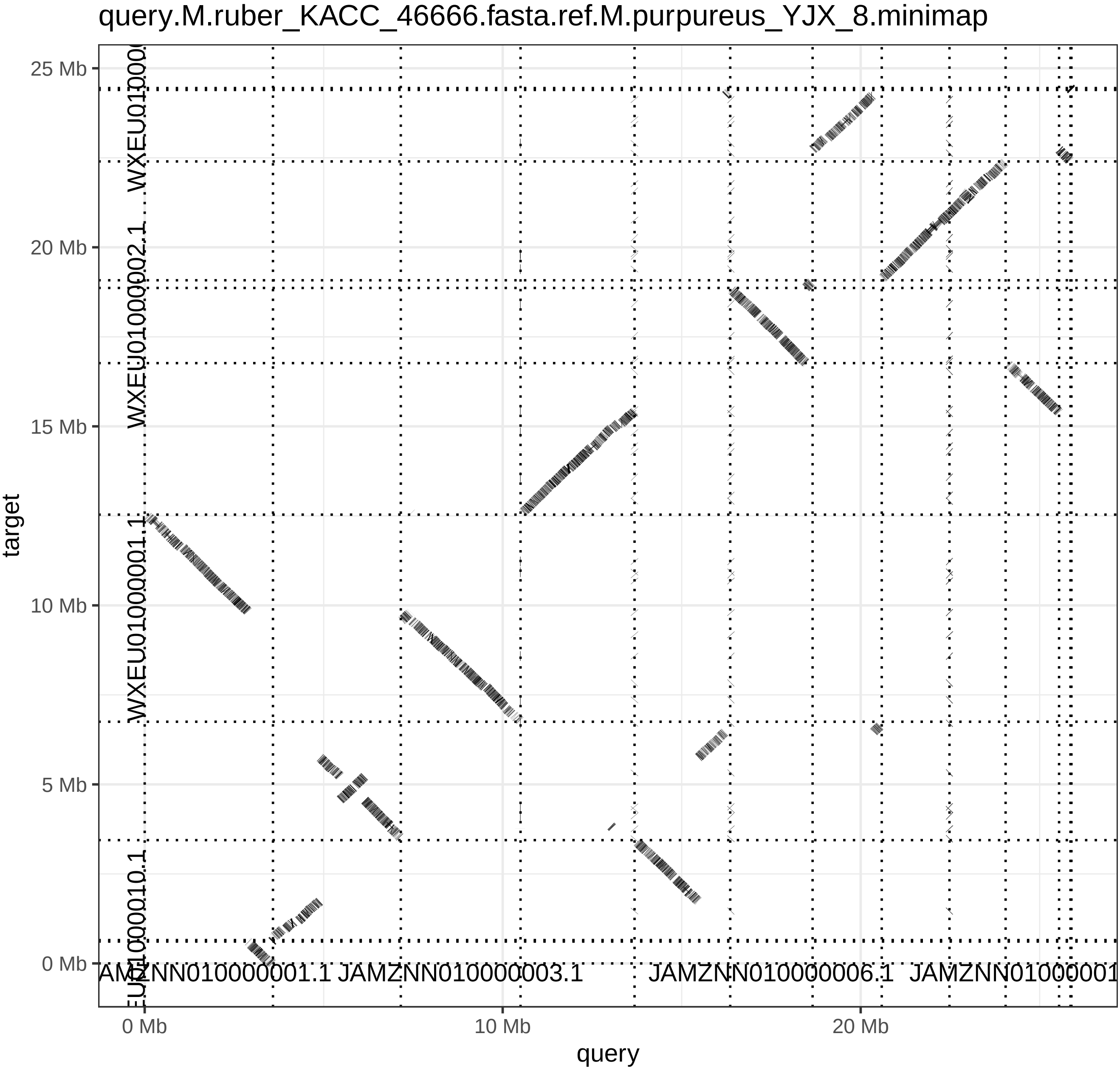

query.M.ruber_KACC_46666.fasta.ref.M.purpureus_YJX_8.minimap
target
query
25 Mb
20 Mb
15 Mb
10 Mb
5 Mb
0 Mb
0 Mb
10 Mb
20 Mb
WXEU010000001.1
WXEU010000002.1
AMZNN010000001.1
JAMZNN010000003.1
JAMZNN010000006.1

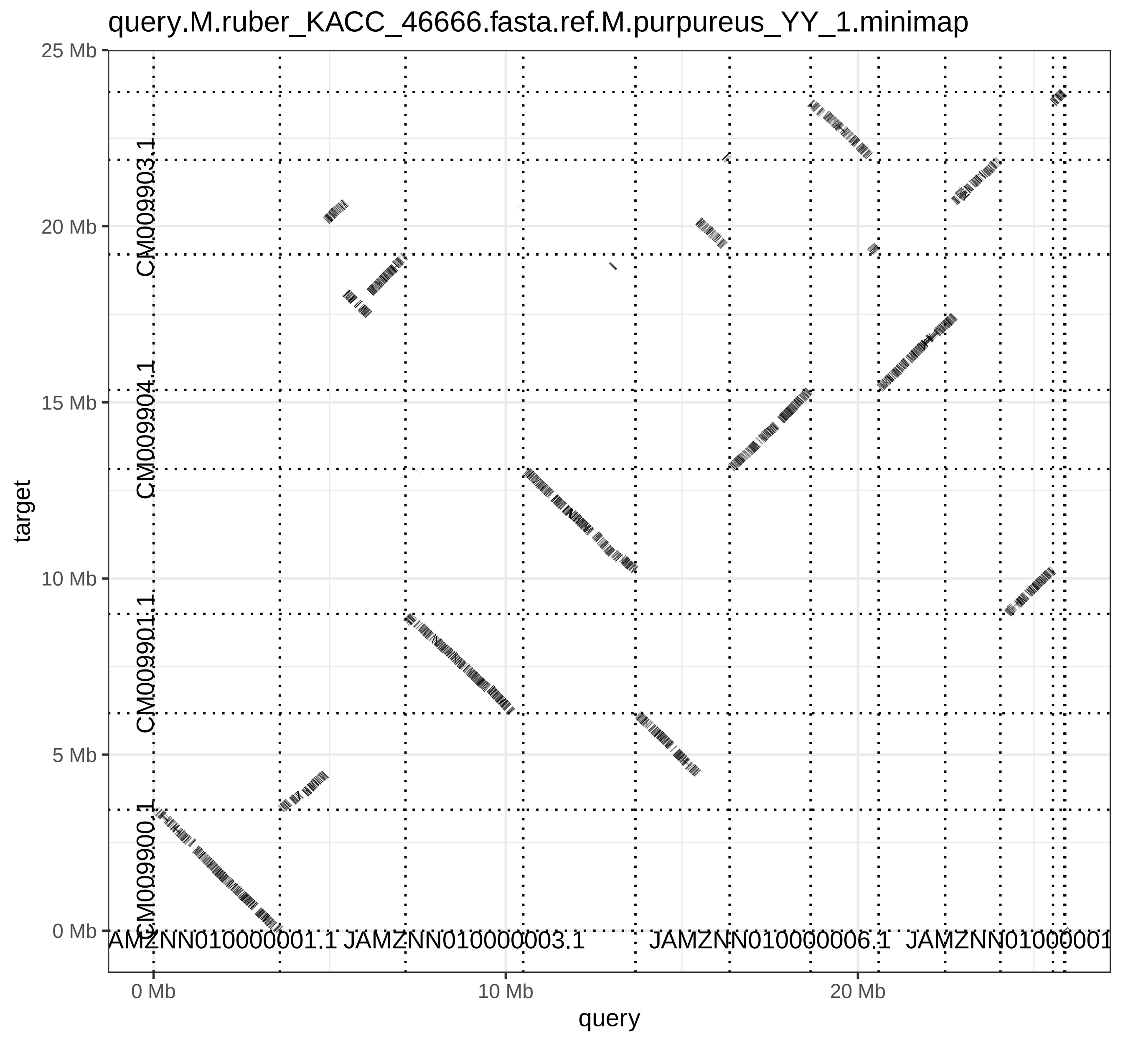
query.M.ruber_KACC_46666.fasta.ref.M.purpureus_YY_1.minimap
target
query
25 Mb
20 Mb
15 Mb
10 Mb
5 Mb
0 Mb
0 Mb
10 Mb
20 Mb
CM009903.1
CM009904.1
CM009901.1
CM009900.1
AMZNN010000001.1
JAMZNN010000003.1
JAMZNN010000006.1
JAMZNN01000001

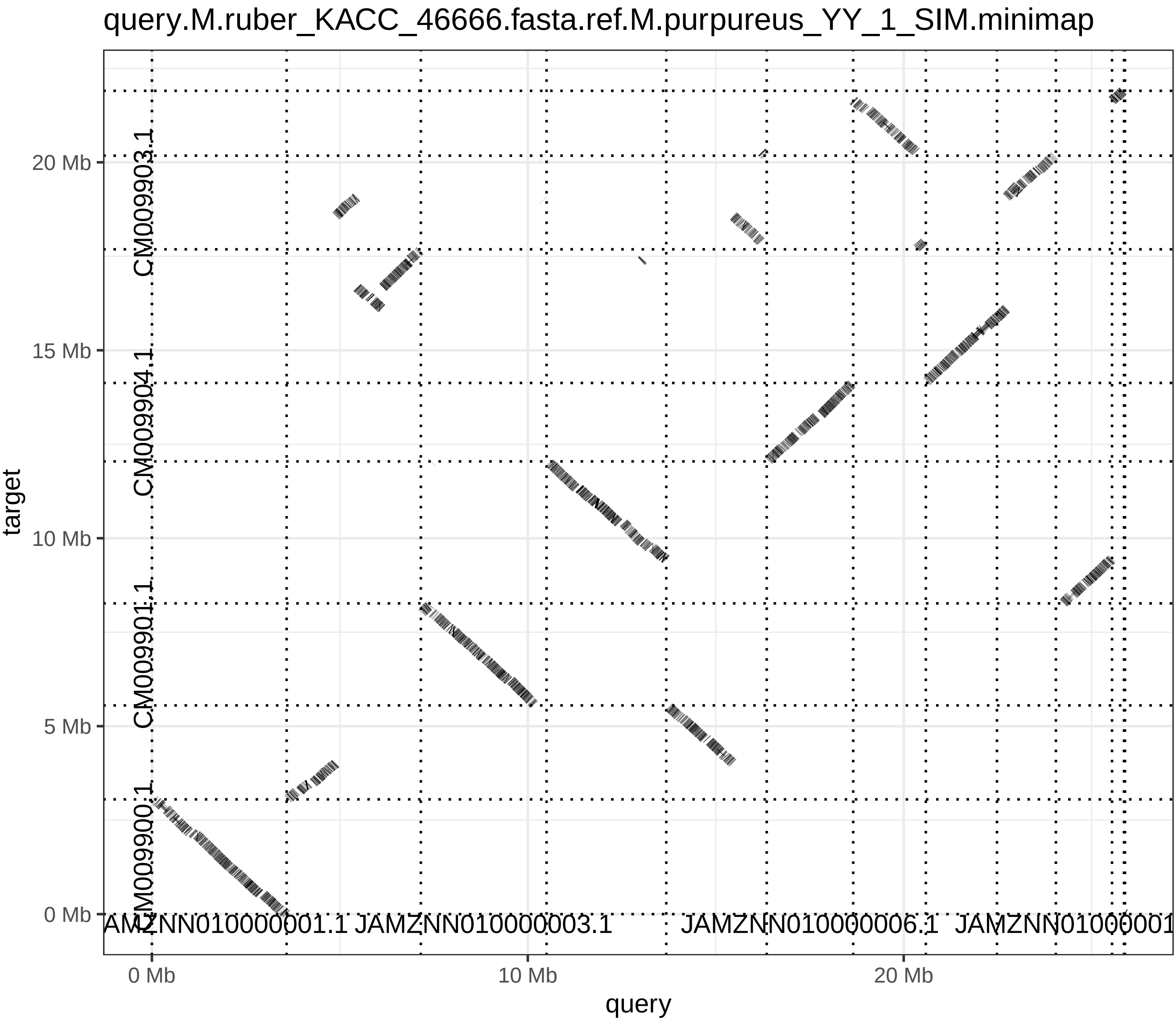
query.M.ruber_KACC_46666.fasta.ref.M.purpureus_YY_1_SIM.minimap
target
query
0 Mb
5 Mb
10 Mb
15 Mb
20 Mb
0 Mb
10 Mb
20 Mb
CM009903.1
CM009904.1
CM009901.1
JAMZNN010000001.1
JAMZNN010000003.1
JAMZNN010000006.1

query.M.ruber_KACC_46666.fasta.ref.M.ruber_FWB13.minimap

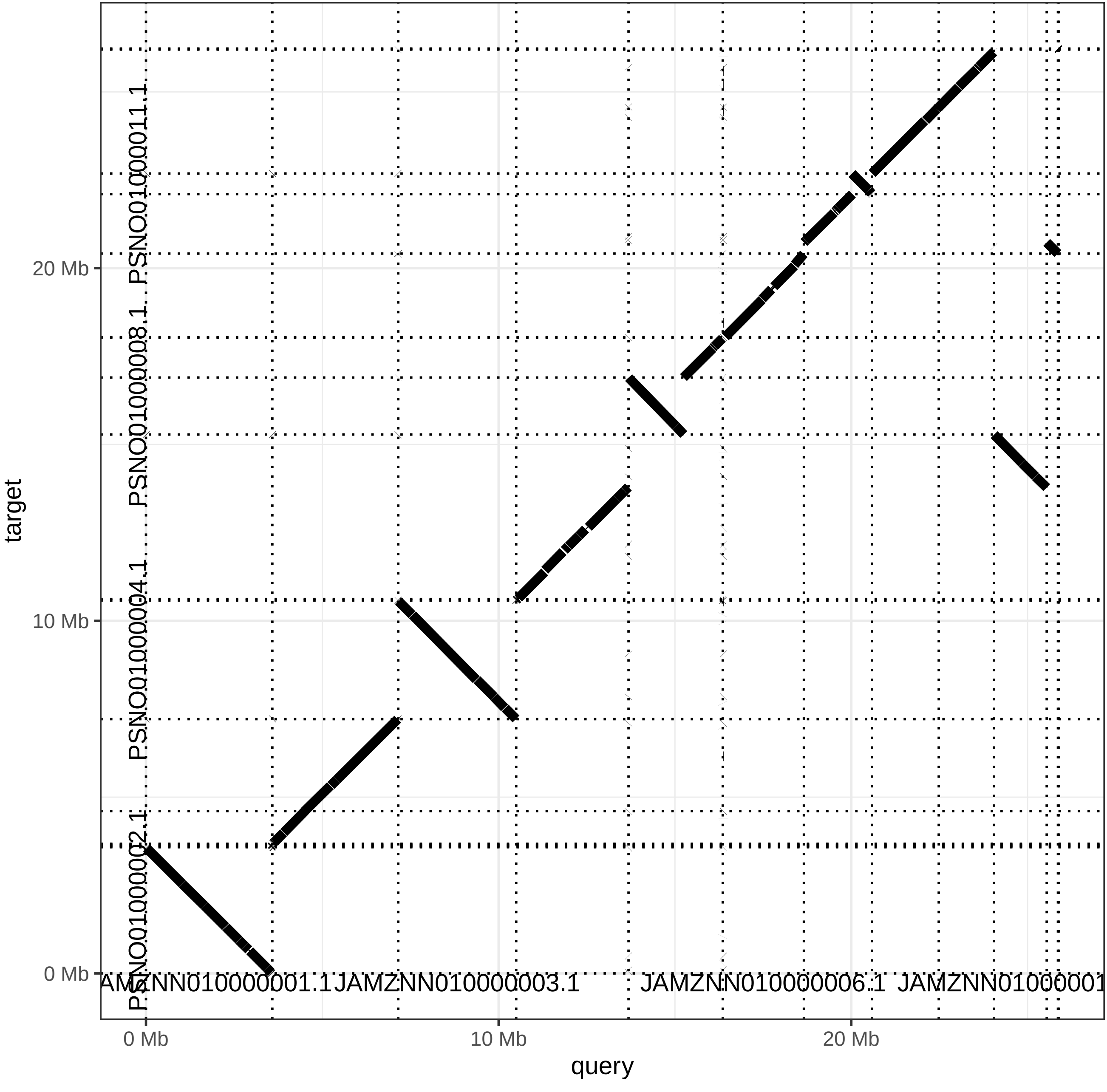

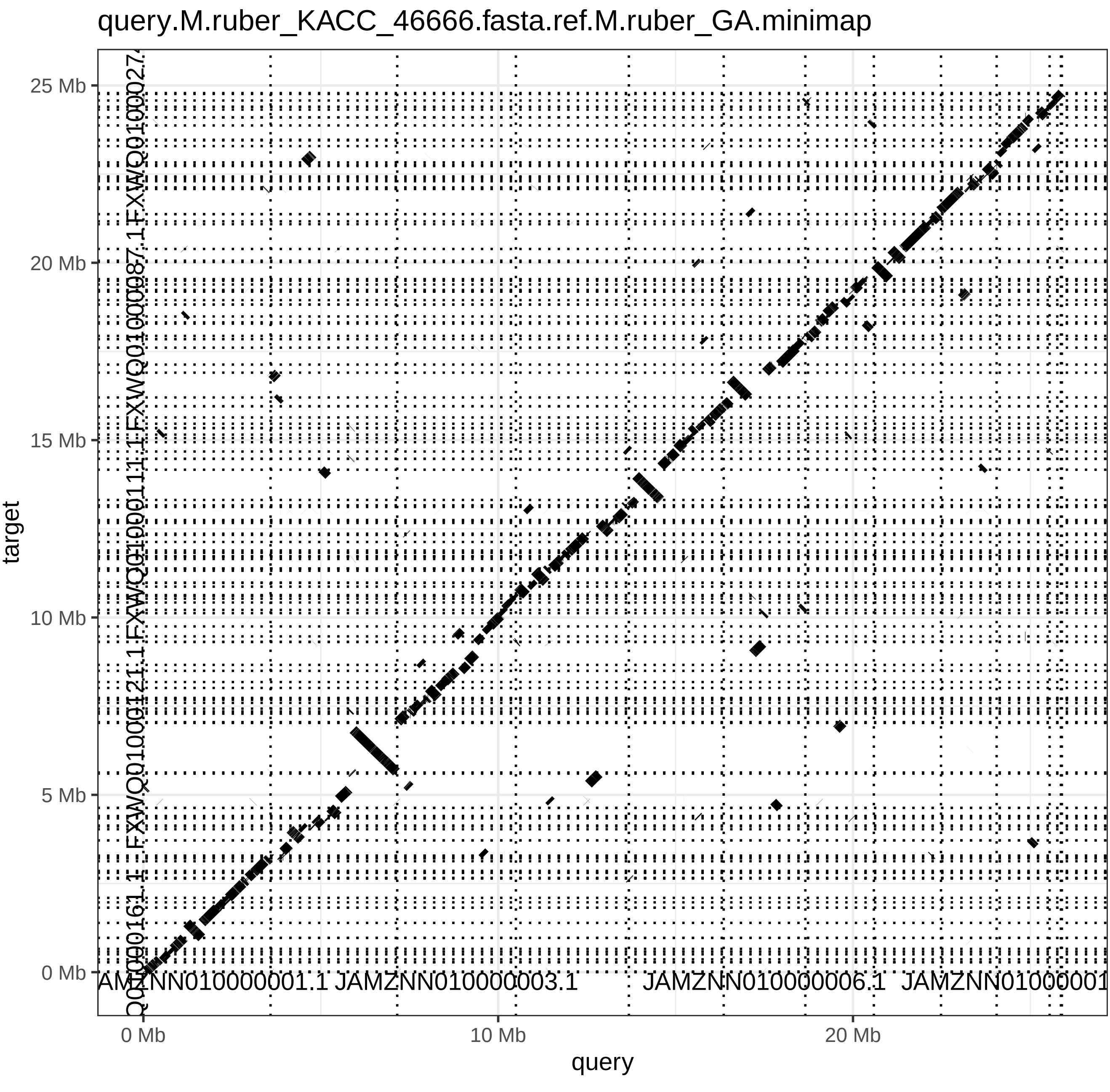

query.M.ruber_KACC_46666.fasta.ref.M.ruber_GA.minimap
target
query
25 Mb
20 Mb
15 Mb
10 Mb
5 Mb
0 Mb
0 Mb
10 Mb
20 Mb
AMZNN010000001.1
JAMZNN010000003.1
JAMZNN010000006.1
JAMZNN01000001
FXWQ0100027.1
FXWQ01000087.1
FXWQ01000111.1
FXWQ01000121.1
FXWQ01000161.1
